# The acylative kinetic resolution of 1,2-azaborine naphthols

**DOI:** 10.1039/d6sc02046a

**Published:** 2026-04-09

**Authors:** Martha I. Prindl, Aidan P. McKay, David B. Cordes, Andrew D. Smith

**Affiliations:** a EaStCHEM, School of Chemistry, University of St Andrews St Andrews KY16 9ST Fife UK ads10@st-andrews.ac.uk

## Abstract

The enantioselective synthesis of atropisomeric molecules containing stereogenic axes is becoming increasingly important due to their growing incorporation within medicinally relevant compounds. Organocatalytic routes to selectively prepare highly enantioenriched stereogenic axes containing a carbon(sp^2^)–boron(sp^2^) bond remain underdeveloped due to the inherent challenge of the longer C–B bond length (1.58 Å) compared to its C(sp^2^)–C(sp^2^) counterpart (1.49 Å). This manuscript showcases the development of an isothiourea catalysed acylative kinetic resolution of 1,2-azaborine frameworks to prepare configurationally stable carbon-boron stereogenic axes with good to excellent stereocontrol (24 examples, selectivity factors up to >200). The scope and limitations of this process have been investigated, with product derivatisation and racemisation studies providing insight into the configurational stability of these species and the association between boron hybridisation and atropisomeric stability. Building on insight gained from these studies preliminary proof of principle investigations concerning an acylative dynamic kinetic resolution in this system has been demonstrated.

## Abstract 2

The acylative kinetic resolution of 1,2-azaborines with excellent stereocontrol (24 examples, selectivity factors up to >200) is reported. Racemisation studies probe their configurational stability leading to proof of principle DKR studies.

## Graphical abstract

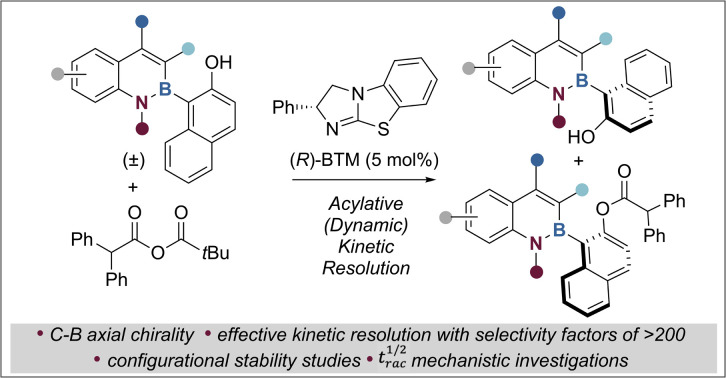

## Introduction

**Fig. 1:**
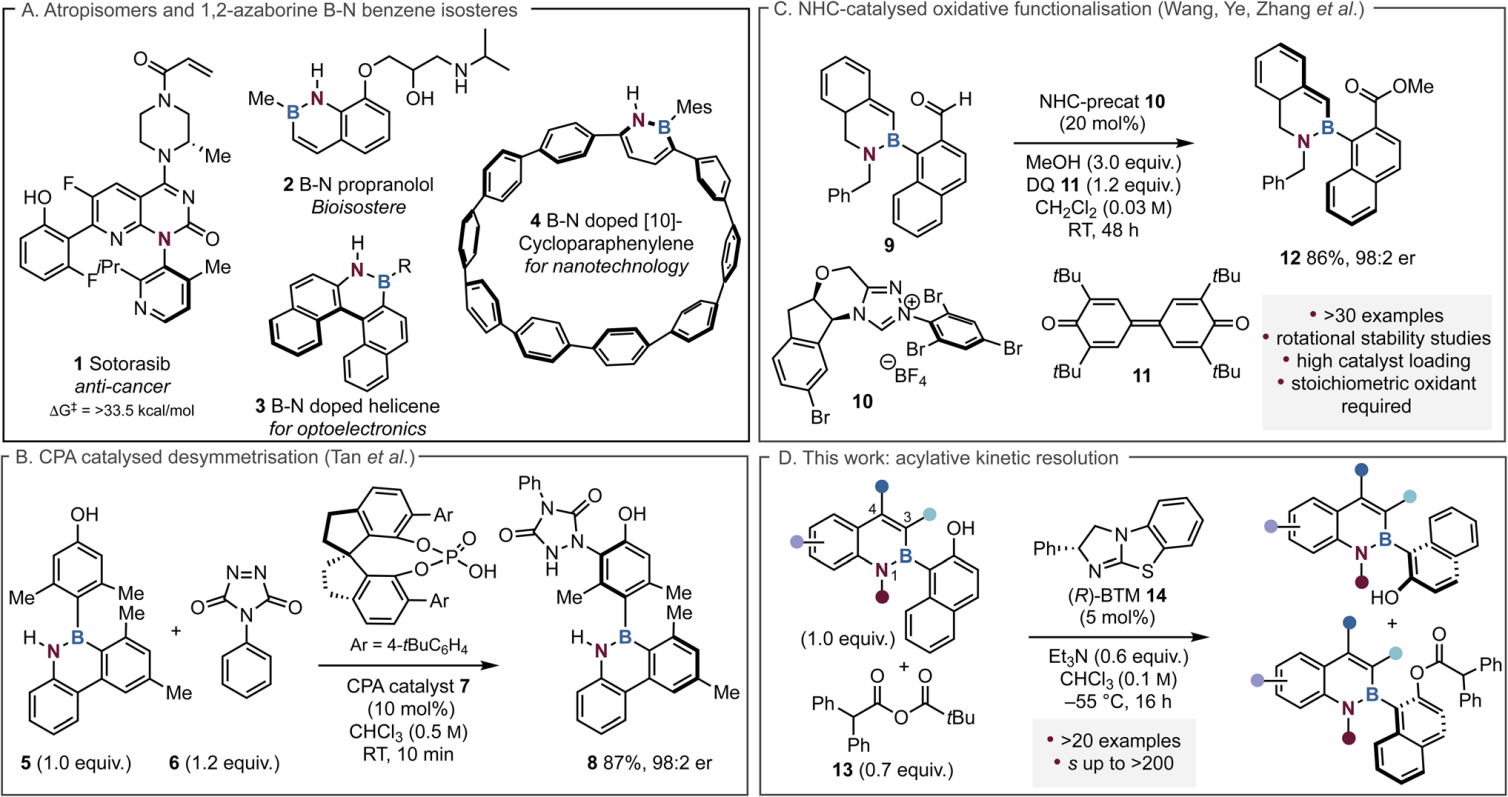
Applications, previous and current work on organocatalytic atropisomeric 1,2-azaborine scaffolds.

Atropisomerism is a fundamental chiral element defined as “stereoisomerism arising from restricted rotation around a single bond”.^1–3^ While configurationally stable chiral atropisomers were first described in 1922,^4^ Oki first defined a practical definition of atropisomers as having a half-life of greater than 1000 s (17 min) in 1983.^5^ A more sophisticated classification system was introduced by LaPlant *et al.*,^6,7^ organising atropisomers into three classes based on their racemisation half-lives and the resultant practical implications. For example, class 1 atropisomers (rotamers) have too short a lifetime to be isolated as distinct species (*t*^1/2^_rac_ < 50 s, Δ*G*^‡^_298_ < 20 kcal mol^−1^) while class 2 atropisomers can be isolated but racemise quickly (50 s < *t*^1/2^_rac_ < 430 d, Δ*G*^‡^_298_ = 20–28 kcal mol^−1^). Class 3 atropisomers (*t*^1/2^_rac_ > 430 d, Δ*G*^‡^_298_ > 28 kcal mol^−1^) are considered configurationally stable and generally amenable for further applications and as such are important components in catalysts, pharmaceuticals, natural products and functional molecules such as photoswitches and chiral recognition reagents.^2,8,9^ For instance, there are currently four FDA approved drugs that are stable atropisomers with the anti-cancer KRAS inhibitor Sotorasib 1 which contains a highly stable C–N axis a representative example^10–14^ (Fig. 1A). Furthermore, it is estimated that ∼30% of small drug molecules approved since 2011 are class 1 atropisomers containing at least one unrestricted stereogenic axis.^9,15^ Commonly studied biaryl or heteroaryl atropisomeric systems incorporate C–C, C–N, C–S and C–O linkages, with methods for their selective preparation alongside their configurational stability having been well explored.^16–20^

To date limited synthetic routes to atropisomeric species that contain a C–B stereogenic axis have been developed, primarily due to the inherent challenge of a lower barrier to rotation that is enabled by a longer C(sp^2^)–B(sp^2^) bond (1.58 Å) compared to a C(sp^2^)–C(sp^2^) bond (1.49 Å).^21,22^ 1,2-Azaborines are a distinctive class of boron-containing molecules that replace a C–C bond with a B–N bond, rendering them benzene isosteres.^23–25^ As such, applications have mainly focused on their potential as bioisosteres such as 2, along with their unique photophysical or electrochemical properties within molecules, including doped helicine motifs 3 and cycloparaphenylenes (CPPs) 4 (Fig. 1A).^26–37^ A handful of examples exemplified by Song and co-workers, have employed transition metal catalysts to develop highly enantioselective methods to generate atropisomeric C–B azaborine axes^38–44^ but notably only limited examples employ organocatalytic methods. The first state-of-the art organocatalytic approach was developed by Tan and co-workers in 2021, who used a chiral phosphoric acid (CPA) catalyst 7 to desymmetrise azaborine 5 to generate products such as 8 with excellent enantiocontrol (Fig. 1B).^45^ In an alternative dynamic kinetic resolution (DKR) approach, Zhang and co-workers employed an N-heterocyclic carbene (NHC) catalysed oxidative esterification process to resolve a wide range of azaborine scaffolds, giving the corresponding products in up to 99 : 1 er (Fig. 1C).^46^ Although effective, this approach requires high catalyst loadings of the NHC catalyst (20 mol%) as well as a stoichiometric amount of the oxidant 3,3′,5,5′-tetra-*tert*-butyldiphenoquinone (DQ).

The use of Lewis basic isothiourea catalysts within enantioselective acylation processes has been well established since Birman's founding work that demonstrated the effective kinetic resolution of secondary alcohols *via* selective benzotetramisole (BTM) 14 catalysed acyl transfer.^47^ Since this initial demonstration, multiple protocols for isothiourea catalysed kinetic resolutions for the preparation of point chiral molecules have been developed, including the resolution of secondary^48,49^ and more challenging tertiary alcohols,^50^ diols^51^ and fluorohydrins.^52^ The application of isothiourea catalysts for the generation of enantioenriched materials containing C–C, C–N or N–N stereogenic axes has previously been explored, including within a thia-Michael addition cyclisation,^53^ the *N*-acylation of sulfonamides^54^ and *N*-acylaminoindoles,^55^ and the resolution of biaryl diol species.^56,57^ At the outset of this project the organocatalytic development of a kinetic resolution process to introduce a C–B stereogenic axis *via* isothiourea catalysis had not been studied. In this context, this manuscript describes the effective kinetic resolution of a range of 1,2-azaborine naphthols to afford highly enantioenriched ester and alcohol products. The key role of substitution at C(3)-, C(4)- and N(1)- in determining the selectivity in these processes has been interrogated, with the configurational stability of a range of substrates probed (Fig. 1D). During the finalisation of this manuscript a related but orthogonal acylative kinetic resolution of 1,2-azaborines using an isothiourea catalyst (tetramisole) was developed by the Li group. This elegant work incorporated a C(3)-iodine substituent as a necessary constraint to achieve high enantioinduction using isobutyric anhydride as an acyl donor, and required a high 20 mol% catalyst loading over 48 hours reaction time.^58^ The barrier to rotation in the C(3)-iodoalcohol substrate was calculated to be 36.5 kcal mol^−1^, consistent with significant configurational stability, meaning that a DKR in this system would be unlikely. In comparison, this work employs lower catalyst loadings (5 mol% of (*R*)-14 over 16 hours) and focuses upon alternative less sterically hindered azaborine motifs bearing C(3)-Me or H-substituents. Furthermore, the sterically encumbered diphenylacetic pivalic anhydride was used in this process, delivering access to enantioenriched azaborine substrates not tolerated in the Li group publication. Significantly, mechanistic investigations concerning 1,2-azaborine racemisation and boron hybridisation, coupled with extending this approach to proof of concept acylative DKR processes distinguishes this from Li's work, and further expands the methods of developing enantioenriched C–B axes within azaborine motifs.

## Results and discussion

### Optimisation of the acylative kinetic resolution of a model 1,2-azaborine

**Fig. 2:**
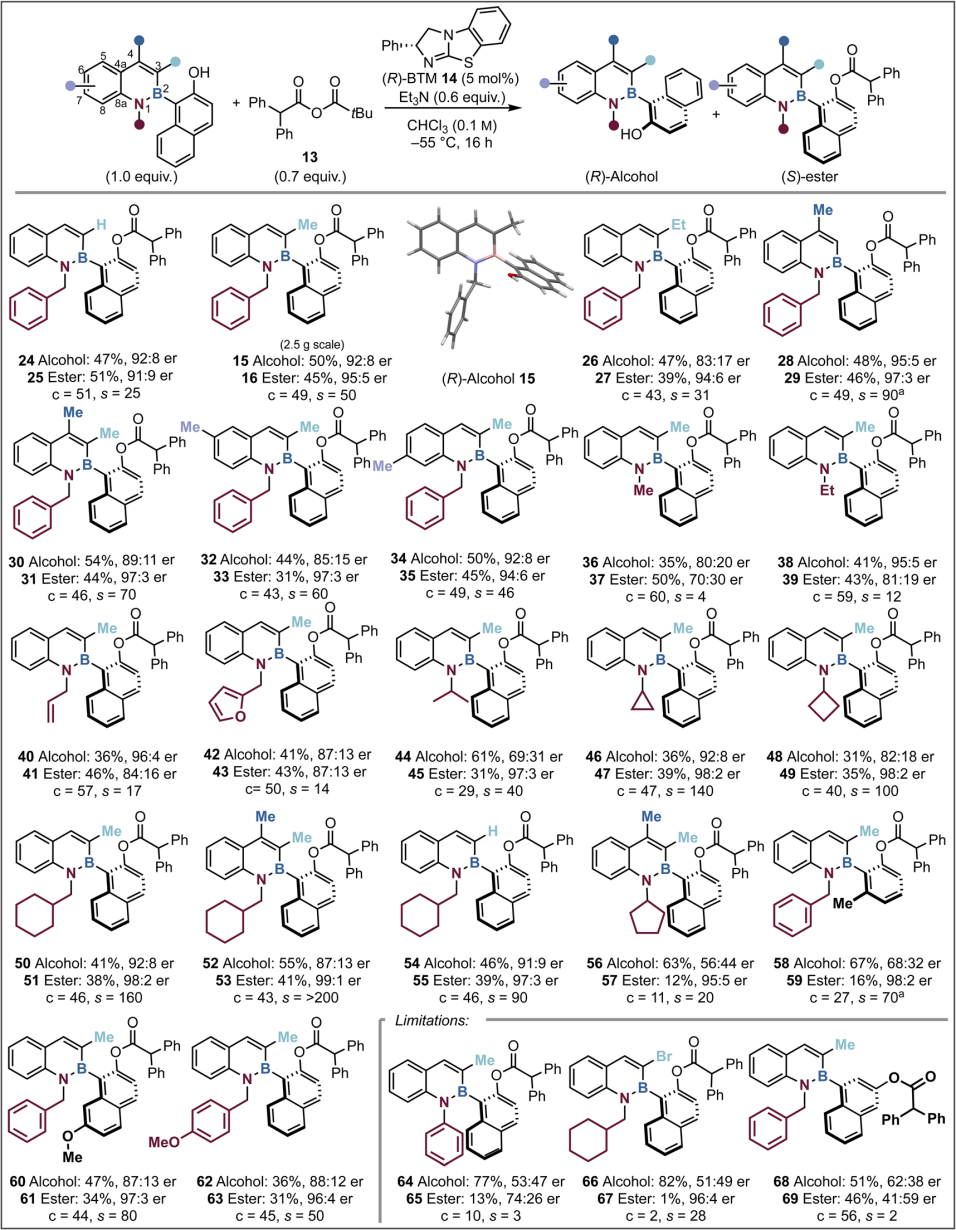
Scope of the reaction. Reactions were performed on a 0.2–0.3 mmol scale. Yields are isolated. Product enantioselectivity determined by HPLC analysis on a chiral stationary phase. (a) Used anhydride 21 to minimise reaction impurities. A reaction using (±)-44 was repeated using 10 mol% (*R*)-14 for 24 h to try and increase conversion, but no improvement to the results was observed. The following equations were used for calculations: c = ee(alcohol)/ee(alcohol) + ee(ester). *s* = ln[(1 − conv)(1 − ee(alcohol))]/(ln[(1 − conv)(1 + ee(alcohol))]. *N.B.* During starting material synthesis the corresponding aminostyrene precursor is isolated as a mixture of *Z*/*E* isomers, but this is inconsequential to the next steps of the azaborine synthesis.

To probe the viability of an acylative kinetic resolution process, initial optimisation investigated 1,2-azaborine substrate 15 bearing an *N*-benzyl and C(3)-Me substituents with diphenylacetic anhydride 21 as model reactants (Table 1). Initial conditions employed 10 mol% of isothiourea (*R*)-BTM 14 with 0.5 equivalents of anhydride 21 and 0.5 equivalents of Et_3_N in CHCl_3_ at room temperature for 16 hours, giving 53% conversion to ester 16 with promising selectivity (*s* = 9, entry 1), giving alcohol 15 (85 : 15 er) and ester 16 (81 : 19 er). Changing the Lewis base catalyst to tetramisole 20 gave reduced conversion and moderate stereoselectivity (entry 2). Further work using (*R*)-BTM sequentially decreased the catalyst loading to 5 and then 1 mol% of 14 (entries 3 and 4) with limited variation in conversion or selectivity. The introduction of isobutyric anhydride 22 with F-BTM 19 afforded poor selectivity (*s* = 3). Lowering the reaction temperature was found to have a significantly beneficial effect upon selectivity (entries 8–10), with optimal selectivity observed at −50 °C (c = 43, *s* = 50, entry 10). Further reducing the temperature to −75 °C afforded no conversion to ester 16 (entries 11–13). Altering the base and anhydride stoichiometry (to 0.6 and 0.7 equivalents respectively), changing to the mixed anhydride 13 and increasing the catalyst loading of 14 up to 5 mol% at −55 °C improved the conversion to 53%, providing optimal KR conditions with high selectivity (*s* = 43, entry 14), giving alcohol 15 (98 : 2 er) and ester 16 (93 : 7 er). Propionic anhydride 23 was also screened but gave reduced stereoselectivity (c = 45, *s* = 3), with the additional steric bulk provided by the diphenyl substitution on 13 being required for optimal selectivity. A 2.5 gram scale-up reaction on the model substrate 15 was performed and pleasingly no degradation in enantiocontrol or conversion was observed, with enantioenriched 15 and 16 obtained with a selectivity factor of 50 and a conversion of 49, corresponding to 50% isolated yield of the alcohol 15 (92 : 8 er), and 45% ester 16 (95 : 5 er, Fig. 2). The absolute (*R*)-configuration within resolved alcohol 15 was unambiguously determined by X-ray crystallographic analysis, with all other alcohols assigned by analogy and allowing the (*S*)-configuration within the ester to be assigned (See SI and Fig. 2).

**Table 1:** Optimisation of the reaction conditions^a^

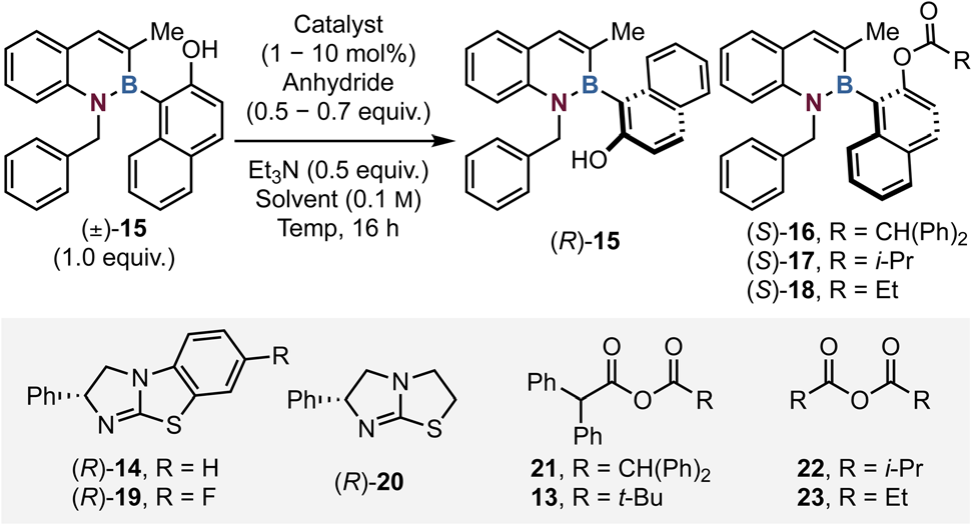							
Entry	Temp. (°C)	Catalyst (mol%)	Solvent (0.1 M)	Anhydride (equiv.)	Conversion (c)	Selectivity factor (*s*)	Er^b^
1	RT	(*R*)-BTM 14 (10)	CHCl_3_	21 (0.5)	53	9	85 : 15/81 : 19
2	RT	(*R*)-TM 20 (10)	CHCl_3_	21 (0.5)	39	8	72 : 28/84 : 16
3	RT	(*R*)-BTM 14 (5)	CHCl_3_	21 (0.5)	44	10	78 : 22/85 : 15
4	RT	(*R*)-BTM 14 (1)	CHCl_3_	21 (0.5)	50	10	83 : 17/83 : 17
5	RT	(*R*)-F-BTM 19 (1)	CHCl_3_	21 (0.5)	43	12	78 : 22/87 : 13
6	RT	(*R*)-F-BTM 19 (1)	CHCl_3_	22 (0.5)	52	3	71 : 29/69 : 31
7	RT	NONE	CHCl_3_	21 (0.5)	N.A^c^	—	—
8	0	(*R*)-BTM 14 (1)	CHCl_3_	21 (0.5)	47	14	84 : 16/88 : 12
9	−20	(*R*)-BTM 14 (1)	CHCl_3_	21 (0.5)	44	34	84 : 16/94 : 6
10	−50	(*R*)-BTM 14 (1)	CHCl_3_	21 (0.5)	43	50	85 : 15/96 : 4
11^d^	−75	(*R*)-BTM 14 (1)	Et_2_O	21 (0.5)	N.R	—	—
12^d^	−75	(*R*)-BTM 14 (1)	PhMe	21 (0.5)	N.R	—	—
13^d^	−75	(*R*)-BTM 14 (1)	CH_2_Cl_2_	21 (0.5)	N.R	—	—
14^e^	−55	(*R*)-BTM 14 (5)	CHCl_3_	13 (0.7)	53	43	98 : 2/93 : 7
15^e^	−55	(*R*)-BTM 14 (5)	CHCl_3_	23 (0.7)	45	3	67 : 33/70 : 30

a All reactions were performed on a 0.1 mmol scale.

b Ratio of alcohol/ester er, determined by HPLC analysis on a chiral stationary phase.

c ∼7% ester observed.

d Reacted for 24 hours.

e Using 0.6 equiv. of Et_3_N. Following literature, *s* values under 50 are reported to nearest integer, above 50 to the nearest 10 and for very high *s* factor values > 200 is used.^59^ The following equations were used for calculations: c = ee(alcohol)/ee(alcohol)+ee(ester). *s* = ln[(1 − conv)(1 − ee(alcohol))]/(ln[(1 − conv)(1 + ee(alcohol))].

### Scope and limitations of the acylative kinetic resolution

With the optimal conditions in hand the scope and limitations of the kinetic resolution process were probed. Variation at the C(3)-position was first considered as this was predicted to significantly change the steric environment around the C–B stereogenic axis which could directly alter both selectivity and conversion. Introduction of a C(3)-H or C(3)-Et substituent was readily tolerated, giving alcohol 24 and ester 25 (C(3)-H, c = 51, *s* = 25, er_ester_ = 91 : 9) and alcohol 26 and ester 27 (C(3)-Et, c = 43, *s* = 31, er_ester_ = 94 : 6) with good but slightly diminished selectivity relative to the model substrate.

Kinetic resolution of alcohols 24 (*s* = 25) and 26 (*s* = 31) proceeded with higher selectivity with anhydride 13, compared to Li's work (for 24 (*s* = 7) and 26 (*s* = 6) respectively) using anhydride 22, highlighting the substrate class orthogonality.^58^ Variation to a C(4)-Me substituent provided higher enantioselectivity than the model system, giving alcohol 28 and ester 29 (C(4)-Me, c = 49, *s* = 90, er_ester_ = 97 : 3), which is postulated to arise as a consequence of a “buttressing effect”, that harnesses the C(4)-Me interaction with the *ortho*-C(3)-H to hinder axis rotation.^60,61^ The incorporation of methyl substitution at both C(3) and C(4) was also tolerated, giving 30 and 31 with excellent levels of selectivity in the acylative kinetic resolution (c = 46, *s* = 70, er_ester_ = 97 : 3). The addition of a methyl substituent to the C(6)- or C(7)-position gave similar results to that observed within the model substrate. These reactions afforded alcohol 32 and ester 33 (C(6)-Me, c = 43, *s* = 60, er_ester_ = 97 : 3) as well as alcohol 34 and ester 35 (C(7)-Me, c = 49, *s* = 46, er_ester_ = 94 : 6) respectively.

Subsequent investigations focused upon the impact of changing the *N*(1)-substituent within the azaborine as this was also predicted to significantly impact the observed selectivity within the system. With a C(3)-Me substituent, sequential variation from an *N*(1)-Me (c = 60, *s* = 4, er_ester_ = 70 : 30), to *N*(1)-Et (c = 59, *s* = 12, er_ester_ = 81 : 19) to *N*(1)-allyl substitution (c = 57, *s* = 17, er_ester_ = 84 : 16) led to a sequential increase in stereoselectivity. In the latter two cases, allowing the reactions to proceed to >50% conversion allowed access to highly enantioenriched alcohols *N*(1)-Et 38 (41%, 95 : 5 er) and *N*(1)-allyl 40 (36%, 96 : 4 er). Compared to the model substrate, incorporation of a *N*(1)-furanylmethyl substituent led to reduced selectivity (c = 50, *s* = 14, er_ester_ = 87 : 13). Incorporation of a branched *N*(1)-*i*-Pr substituent led to higher selectivity but reduced conversion (c = 29, *s* = 40) giving ester 45 with high enantiocontrol (31%, 97 : 3 er). Building on this observation the introduction of branched but conformationally constrained *N*(1)-cycloalkyl groups was considered, with *N*(1)-cyclopropyl substitution affording significantly improved selectivity (c = 47, *s* = 140), giving good yields of highly enantioenriched ester 47 (39%, 98 : 2 er) and alcohol 46 (36%, 92 : 8 er). Likewise, variation to a *N*(1)-cyclobutyl group on 48 afforded excellent selectivity (*s* = 100, er_ester_ = 98 : 2). Extension of this approach to the β-branched *N*(1)-cyclohexylmethyl substituent led to good conversion and excellent stereoselectivity (c = 46, *s* = 160), giving good yields of highly enantioenriched ester 51 (38%, 98 : 2 er) and alcohol 50 (41%, 92 : 8 er). Further developments combined methyl substitution at both C(3)- and C(4)- with an *N*(1)-cyclohexylmethyl substituent leading to excellent selectivity (c = 43, *s* = >200), giving alcohol 52 (55%, 87 : 13 er) and ester 53 (41%, 99 : 1 er). Further use of an *N*(1)-cyclohexylmethyl substituent with C(3)-H substitution also led to kinetic resolution with high selectivity (c = 46, *s* = 90), giving good yields of highly enantioenriched ester 55 (39%, 97 : 3 er) and alcohol 54 (46%, 91 : 9 er). Disappointingly, the introduction of an *N*(1)-cyclopentyl substituent with methyl substitution at both C(3) and C(4) gave poor reactivity and reduced stereoselectivity (c = 11, *s* = 20). The effect of changing the naphthol scaffold to a 2,6-disubstituted phenol on 58 was tested, giving reduced conversion but high selectivity (c = 27, *s* = 70) affording ester 59 in low yield but with high enantiocontrol (16%, 98 : 2 er). Further developing the napthol ring to an OMe-substituted variant was also well tolerated (c = 44, *s* = 80), giving enantioenriched ester 61 (34%, 97 : 3 er) and alcohol 60 (47%, 87 : 13 er). Comparative to the model substrate, the introduction of a *N*(1)-paramethoxybenzyl group on alcohol 62 was also readily tolerated, (c = 45, *s* = 50, er_ester_ = 96 : 4). Several limitations within this system were also identified. For example, introduction of a bulky *N*(1)-phenyl substituent on 64 led to significantly reduced substrate reactivity, giving poor conversion (c = 10) and selectivity (*s* = 3). Incorporation of a C(3)-bromine substituent also led to very poor conversion (c = 2), presumably reflecting the significant steric effect of incorporating bromine substitution at this position, coupled with the use of a sterically encumbered acylating reagent. This limitation highlights the orthogonality in acylating reagent design with Li's work requiring a C(3)-halogen for high enantiodiscrimination while using isobutyric anhydride 22.^58^ Attempts to extend this process to selective acylation of a *meta*-substituted naphthol led to good reaction conversion (c = 56) but with very poor selectivity (*s* = 2), which is postulated to be due to the greater distance from the alcohol to the proposed recognition motif that dictates stereoselectivity.

### Quantifying the configurational stability of azaborines

To understand the configurational stability profile of a number of model azaborine substrates, the racemisation half-life (and barrier to rotation) of a series of enantioenriched alcohols and ester derivatives was investigated.^62^ Heating an enantioenriched substrate of known enantiopurity in xylene to a defined temperature and monitoring the degradation of enantiomeric excess over time was used for this purpose. Due to observed but inconsistent decomposition, a different temperature was used for each substrate. As a representative example, the barrier to rotation for model C(3)-Me substituted alcohol 15 was determined (*t*^1/2^_rac_ = 0.27 h at 404 K, Δ*G*^‡^_404_ = 30.3 kcal mol^−1^). Assuming that Δ*G*^‡^ is invariant with temperature,^62^ this corresponds to a racemisation half-life at room temperature of ∼27 years. This level of configurational stability defines 15 as a class 3 atropisomer.^9^ The barrier to rotation along the C–B axis of the corresponding C(3)-H substituted alcohol 24 was calculated to be substantially lower than the C(3)-Me analogue (*t*^1/2^_rac_ =12.1 h at 314 K, Δ*G*^‡^_314_ = 25.8 kcal mol^−1^), defining 24 as a class 2 atropisomer. This is consistent with the steric nature of the C(3)-substituent having a significant effect upon configurational lability. Extension of this approach to the corresponding C(3)-Me 16 and C(3)-H 25 substituted esters was also investigated. The same trend was observed, with the rotational barrier for C(3)-Me substituted ester 16 (*t*^1/2^_rac_ = 280 h at 353 K, Δ*G*^‡^_353_ = 31.2 kcal mol^−1^) being significantly larger than the C(3)-H analogue 25 (*t*^1/2^_rac_ = 1.3 h at 382 K, Δ*G*^‡^_382_ = 29.8 kcal mol^−1^). This also indicates that the ester products show significantly enhanced configurational stability compared to the respective alcohols, again consistent with the increased size of the ester in comparison to the alcohol leading to a higher barrier to rotation.

**Fig. 3:**
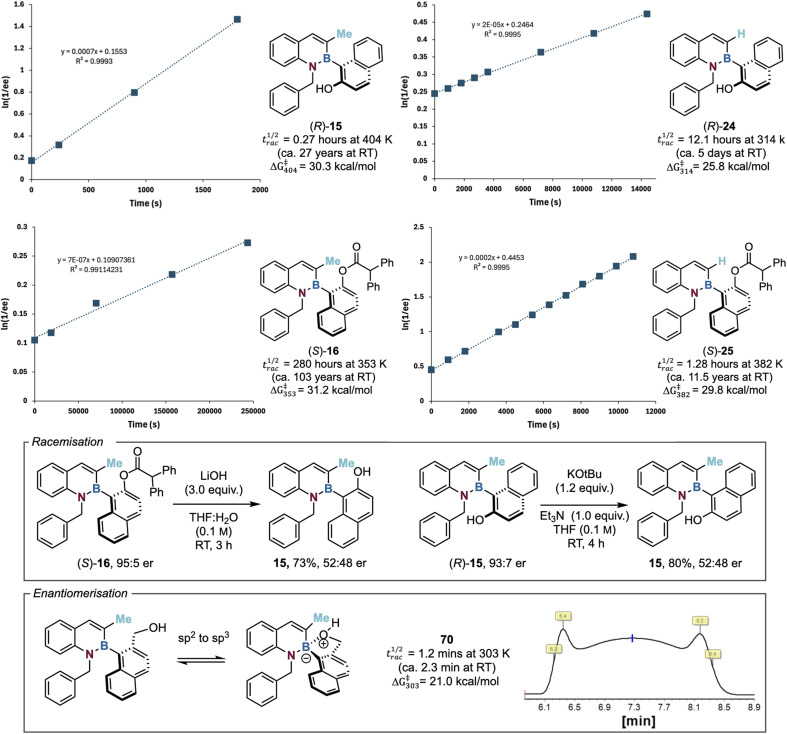
Quantifying the configurational stability of the 1,2-azaborines. With respect to half-life of racemisation calculations, RT = 298 K. *N.B*. The barrier to rotation for alcohol 24 was determined in good agreement with that from Li's group (26.1 kcal mol^−1^).^58^

Further studies noted that upon hydrolysis of enantioenriched ester 16 (95 : 5 er) using 3.0 equivalents of LiOH, while this afforded the alcohol 15 in good yield (73%), it was essentially racemic (52 : 48 er). It is postulated that racemisation of the alcohol occurs through reversible hydroxide anion addition to the boron atom, generating an sp^3^ boron centre which significantly lowers the barrier to rotation around the C–B bond, resulting in racemisation.^38^ Consistent with this proposal, racemisation of enantioenriched alcohol (*R*)-15 (93 : 7 er) was observed over 4 h upon treatment with KO*t*Bu (1.2 equiv.) and Et_3_N (1.0 equiv.) in THF (Fig. 3). Intrigued by this effect, the introduction of a hydroxymethyl substituent within azaborine 70 was investigated. Interestingly, enantiomer interconversion through dynamic HPLC at 303 K was observed for 70 through a diagnostic “Batman” peak shape, indicative of enantiomerisation occurring at the HPLC timescale. Using the DCXplorer software developed by the Trapp group, the barrier to rotation within azaborine 70 was readily determined (*t*^1/2^_rac_ = 1.23 min at 303 K, Δ*G*^‡^_303_ = 21.0 kcal mol^−1^), indicating a significantly lower energy barrier to rotation than the other substrates.^63,64^ While reversible intramolecular nucleophilic addition of the alcohol to the pendant boron atom to form an sp^3^-boron species (resulting in C–B bond elongation) is reasonable, the origin of the observed remarkable effect on configurational lability is unclear. It is speculated that the lability of the azaborine heterocycle is affected by this bond elongation, potentially allowing reversible ring-opening upon coordination, leading to enantiomerisation. The scope and limitations of this observation, as well as further studies concerning the mechanism of this process, is currently under investigation within this laboratory.

### Towards an acylative dynamic kinetic resolution process

**Fig. 4:**
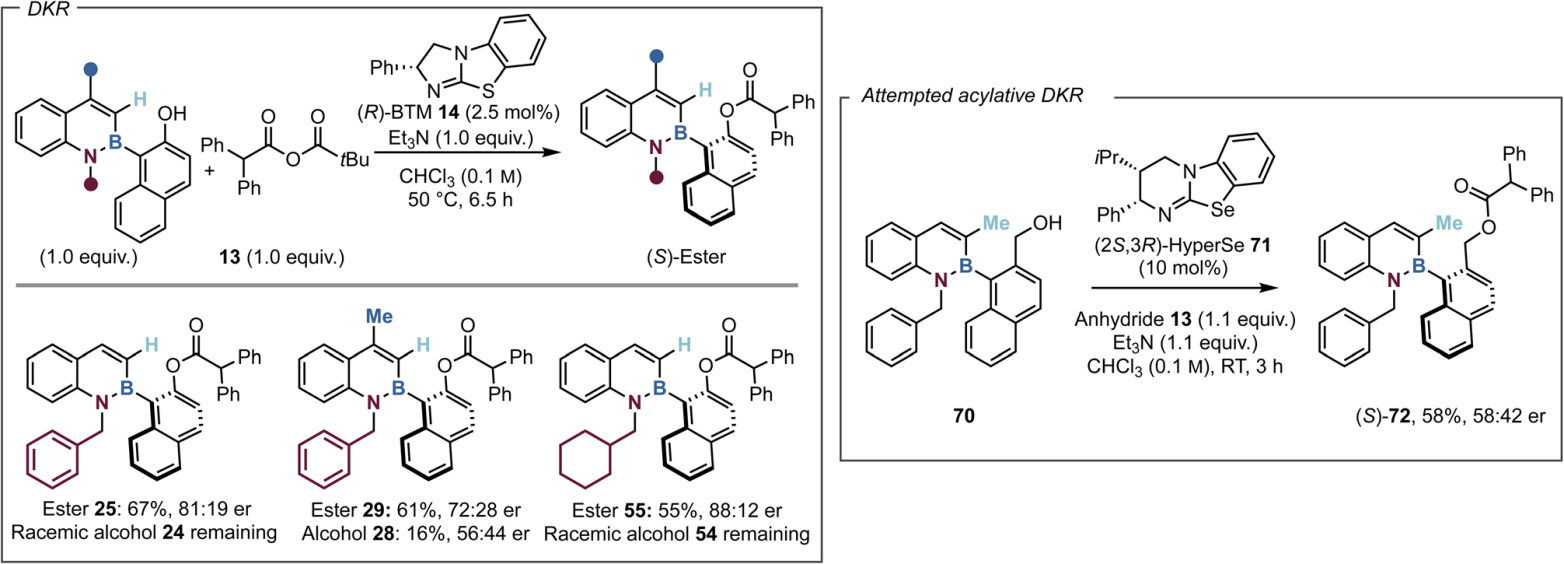
All reactions were performed on a 0.2 mmol scale. Isolated yields are reported. Product enantioselectivity was determined by HPLC analysis on a chiral stationary phase.

Since the barrier to rotation of the C(3)-H substituted alcohol 24 (Δ*G*^‡^_314_ = 25.8 kcal mol^−1^) was calculated to be substantially lower than the corresponding C(3)-Me substituted analogue 15 (Δ*G*^‡^_404_ = 30.3 kcal mol^−1^), it was considered that 24 might undergo a DKR process. This was explored through heating 1.0 equivalents of azaborine 24 and 1.0 equivalents of anhydride 13 with a low catalyst loading of 14 (2.5 mol%) at 50 °C for 6.5 hours. Pleasingly this generated a 67% isolated yield of 25 with reasonable levels of enantioselectivity (81 : 19 er), with the remaining yield attributed to racemic alcohol 24, supporting the dynamic nature of this process (Fig. 4). To clarify that the moderate selectivity observed (81 : 19 er) was not an artifact of ester racemisation, a control study was performed where enantioenriched ester (*S*)-25 was heated without catalyst to 50 °C in CHCl_3_. After 6.5 h, no degradation of enantiopurity was observed, indicating the lower selectivity is not due to *in situ* racemisation. An extensive optimisation process followed, including the screening of a variety of organic and inorganic bases, however no improvement in selectivity or acylation was observed (see SI for further details). The lower yield can be partly attributed to degradation of the azaborine starting material and product upon heating. Azaborines 28 and 54 that worked well within the KR process, were then introduced into the DKR. Disappointingly, the introduction of a C(4)-Me substituent did not improve the results, affording ester 29 in 61% yield and reduced enantioselectivity (72 : 28 er), with the remaining alcohol showing some but poor enantioenrichment (56 : 44 er) after 6.5 h. It is postulated the reduced enantioenrichment of 29 is related to the “buttressing effect” with C(3)-Me substitution that hinders rotation around the B–C axis and disfavours a dynamic process. Variation in the substitution from a *N*(1)-benzyl to a *N*(1)-cyclohexylmethyl group on 55 afforded a mediocre yield (55%), but with greatly improved enantioselectivity (88 : 12 er) while the remaining alcohol 54 was racemic. It is postulated that the moderate enantioselectivity observed within the dynamic process is due to the use of the higher reaction temperature required for alcohol racemisation. The azaborine kinetic resolution optimisation and previous work within the Smith group has observed improved selectivity upon reduced temperatures.^57^ Extension of this approach to the hydroxymethyl substituted analogue 70 that shows dynamic enantiomerization on the HPLC timescale was next attempted. Screening of a number of isothiourea catalysts showed that the Lewis base isoselenourea HyperSe 71 gave the best but still poor enantiocontrol in this transformation (Fig. 4). The enantiomeric ratio of product 72 did not deteriorate over 5 days of storage in solution and so it is assumed the poor enantioinduction is not a consequence of ester racemisation.

### Proposed mechanism and stereochemical model

**Fig. 5:**
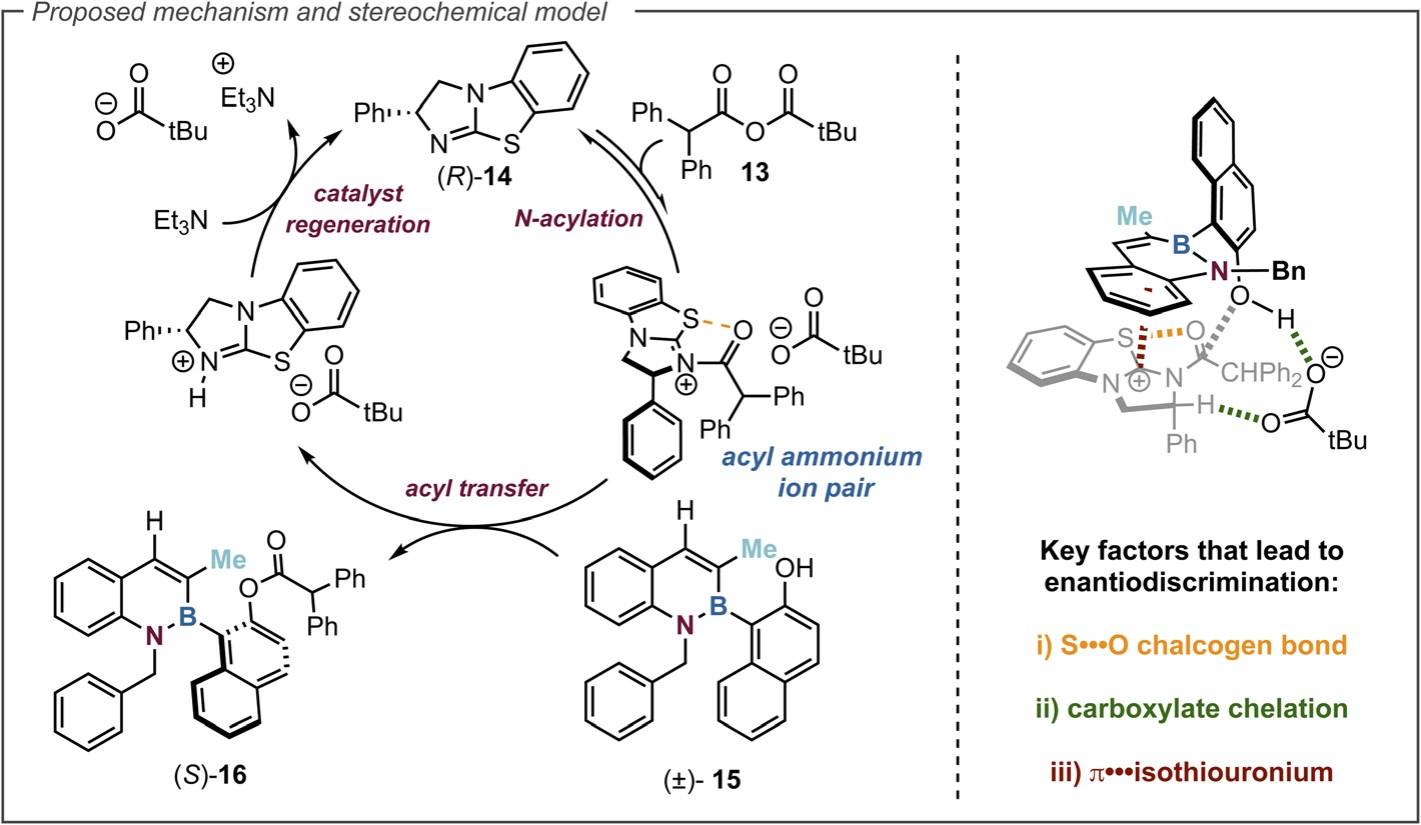
Proposed mechanism and simplified stereochemical rationale.

The proposed catalytic cycle for this transformation is set out in Fig. 5. Initially, (*R*)-BTM 14 is acylated with anhydride 13 leading to an acyl ammonium ion pair. The (*S*)-enantiomer of alcohol 15 preferentially reacts with this acyl ammonium ion pair in the stereodetermining step to afford the ester (*S*)-16, leading to enantioenrichment in the slower reacting (*R*)-15 azaborine alcohol. The resulting ammonium ion pair can be deprotonated by Et_3_N to regenerate the catalyst for turnover. By analogy to previous computational studies the observed selectivity in this acylation can be rationalised using the proposed transition state assembly in Fig. 5. Within this model it is assumed that the key factors that lead to enantiodiscrimination require a 1,5-chalcogen bonding 
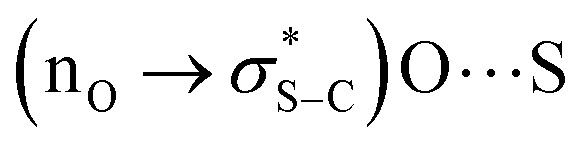
 interaction between the acyl oxygen and the isothiourea catalyst sulfur that acts as a conformational lock.^65–67^ The carboxylate counterion is considered to activate the naphthol towards acylation by deprotonation, while simultaneously participating in non-classical H-bonding to the acylated isothiouronium ion benzylic C–H bond.^68–74^ To deliver high enantioselectivity, a donor substrate motif is needed to promote enantiorecognition through interaction with the positively charged acylated isothiouronium intermediate. A number of enantiorecognition motifs have been employed and recognised in isothiourea-catalysed acylations that include aryl,^48,75–78^ heteroaryl,^79^ alkenyl,^78^ alkynyl,^75^ heteroatom,^80^ C=O,^81,82^ CF_2_,^52^ and P=O substituents.^83^ In this case, utilising the benzofused aromatic substituent to participate in this capacity *via* a π-isothiouronium ion interaction (highlighted in red) leads to the observed selectivity for preferential acylation of the (*S*)-enantiomer.

## Conclusion

In conclusion, an isothiourea catalysed kinetic resolution of 1,2-azaborine substrates has been developed, with selectivity factors of up to >200 observed. Variation of the substitution within the azaborine at C(3)-, C(4)- or N(1)- that are in close proximity to the C–B axis directly impact the observed selectivity of acylation. The enantioenriched azaborines can be readily racemised through heating, or *via* the reversible formation of a tetracoordinate boron ate species. Using this knowledge, a moderately selective acylative dynamic kinetic resolution has been developed.

## Author contributions

M. I. P. and A. D. S. conceived the project; M. I. P. and A. D. S. designed the synthetic experiments; M. I. P. carried out all synthetic experimental studies and analysed the reactions. D. B. C. and A. P. M. carried out single crystal X-ray analysis. M. I. P. and A. D. S. wrote the manuscript. All other correspondence should be addressed to A. D. S.

## Conflicts of interest

The authors declare no competing interests.

## Supplementary Material

SC-OLF-D6SC02046A-s001

SC-OLF-D6SC02046A-s002

## Data Availability

CCDC 2536174 (compound (*R*)-15) contains the supplementary crystallographic data for this paper.^108^ All data (experimental procedures and characterization data) that support the findings of this study are available within the article and its supplementary information (SI). The data underpinning this manuscript is available from the University of St Andrews Research Portal, Pure ID: 333325369 and can be accessed at https://doi.org/10.17630/e86c806f-4b19-4e4b-8842-1430c237adfa. References 84–107 were cited in the SI. Supplementary information is available. See DOI: https://doi.org/10.1039/d6sc02046a.
