# Supplementary material for: The acylative kinetic resolution of 1,2-azaborine naphthols
Source: Chem Sci. 2026 Apr 9;17(23):11412–21. doi: 10.1039/d6sc02046a (PMC13140100; doi:10.1039/d6sc02046a)
Supplement: SC-017-D6SC02046A-s001 [file SC-017-D6SC02046A-s001.pdf]

Supplementary Information (SI) for Chemical Science.
This journal is © The Royal Society of Chemistry 2026

Supporting Information

The Acylative Kinetic Resolution of 1,2-Azaborine Naphthols

Martha I. Prindl,[a] Aidan P. McKay,[a] David B. Cordes,[a] Andrew D. Smith[a]*

[a] School of Chemistry, University of St Andrews, St Andrews, Fife, UK, KY16 9ST

E-mail: ads10@st-andrews.ac.uk

1. General Experimental 3
1.1. Selectivity factor and conversion calculations 6
2. Kinetic Resolution Optimisation 7
2.1. Solvent optimisation 7
2.2. Catalyst optimisation 8
2.3. Base optimisation 9
2.4. Temperature optimisation 10
2.5. Stoichiometry optimisation 11
3. Homologated Alcohol Optimisation 12
3.1. Catalyst and temperature optimisation 12
3.2. Base and solvent optimisation 13
4. Dynamic Kinetic Resolution Optimisation 14
4.1. Catalyst optimisation 14
4.2. Solvent and base optimisation 15
5. General Procedures 16
6. Compound Synthesis 20
6.1. Starting material synthesis 20
6.1.1. Anhydride synthesis 20
6.1.2. Styrene Synthesis 21
6.1.3. Aminostyrene synthesis 25
6.1.4. Naphthol synthesis 38
6.1.5. Alcohol synthesis 39
7. Kinetic Resolution data 55
7.1. Experimental data and HPLC traces 55
7.2. Racemisations 91
7.3. Homologated alcohol substrate 93
7.4. Dynamic Kinetic Resolution data 95
8. Half-life of racemisation studies 98
9. Crystallographic Analysis 101
10. NMR data 102
11. References 307

1. General Experimental

Reactions involving moisture sensitive reagents were carried out in flame-dried glassware under an inert atmosphere (N_2) using standard vacuum line techniques. Anhydrous solvents (tetrahydrofuran, toluene, diethyl ether, dichloromethane) were obtained after passing through an alumina column (Mbraun SPS-800). Petrol is defined as petroleum ether 40–60 °C. All other solvents and commercial reagents were used as received without further purification.

Room temperature (RT) refers to 15–25 °C. Temperatures of 0 °C were obtained using an ice/water bath. Temperature of below 0 °C were obtained using a cryostat with ethyl acetate or acetone as solvent. Reactions involving heating were performed using DrySyn blocks and a contact thermocouple.

Under reduced pressure refers to the use of either a Büchi Rotavapor R-200 with a Büchi V-491 heating bath and Büchi V-800 vacuum controller, a Büchi Rotavapor R-210 with a Büchi V-491 heating bath and Büchi V-850 vacuum controller, a Heidolph Laborota 4001 with vacuum controller, an IKA RV10 rotary evaporator with a IKA HB10 heating bath and ILMVAC vacuum controller, or an IKA RV10 rotary evaporator with a IKA HB10 heating bath and Vacuubrand CVC3000 vacuum controller. Rotary evaporator condensers are fitted to Julabo FL601 Recirculating Coolers filled with ethylene glycol and set to –6 °C.

Analytical thin layer chromatography was performed on pre-coated aluminium plates (Kieselgel 60 F254 silica) purchased from Merck and visualisation was achieved using ultraviolet light (254 nm) and/or staining with aqueous $KMnO_4$ solution followed by heating.

Manual column chromatography was performed in glass columns fitted with porosity 3 sintered discs over Kieselgel 60 silica using the solvent system stated. Automated chromatography was performed on a Biotage® Selekt™ Four running Biotage OS578 with a UV/Vis detector using the method stated and cartridges filled with Kieselgel 60 silica. Boron-capped silica was synthesized in the lab following literature and employed within purification when required to minimise product degradation.[84]

Melting points were recorded on an Electrothermal 9100 melting point apparatus and are not corrected, (dec) refers to decomposition.

Optical rotations were measured on a Perkin Elmer Precisely/Model-341 polarimeter operating at the sodium D line with a 100 mm path cell at 20 °C.

HPLC analyses were obtained on either a Shimadzu HPLC consisting of a DGU-20A5 degassing unit, LC-20AT liquid chromatography pump, SIL-20AHT autosampler, CMB-20A communications bus module, SPD-M20A diode array detector and a CTO-20A column oven or a Shimadzu HPLC consisting of a DGU-20A5R degassing unit, LC-20AD liquid chromatography pump, SIL-20AHT autosampler, SPD-20A UV/Vis detector and a CTO-20A column oven. Separation was achieved using either a DAICEL CHIRALCEL OD-H column or DAICEL CHIRALPAK AD-H, IA, IB, and IC columns using the method stated. HPLC traces of enantiomerically enriched compounds were compared with spectra of an authentic racemic sample prepared from a corresponding reaction using DMAP (10 mol%, see general procedure **D**). It was observed that sample concentration affected the peak shape, with lower sample concentrations, employed *via* smaller injection volumes, improving peak shape consistency for the ester and alcohol azaborine samples (seen in Figure 1).

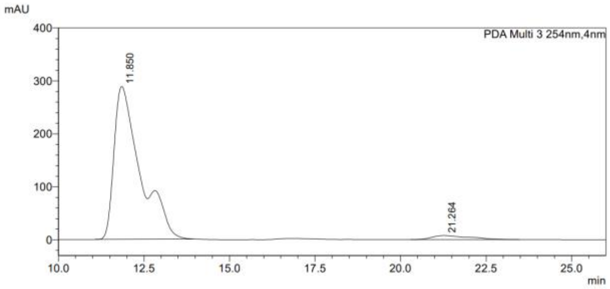

<Peak Table>

PDA Ch3 254nm

Peak#	Ret. Time	Area%
1	11.850	96.353
2	21.264	3.647
Total		100.000

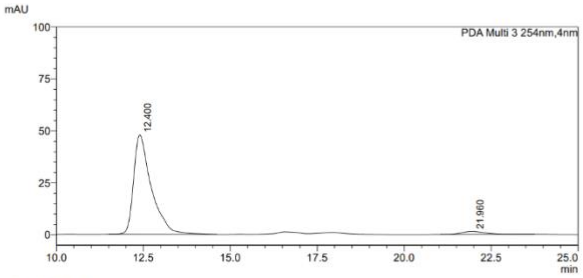

<Peak Table>

PDA Ch3 254nm

Peak#	Ret. Time	Area%
1	12.400	96.301
2	21.960	3.699
Total		100.000

Figure 1: Example of HPLC injection volume affecting peak shape. Left hand side = 10 uL; right hand side = 3 uL. (same sample).

Infrared spectra were recorded on a Shimadzu IRAffinity^{-1} Fourier transform IR spectrophotometer fitted with a Specac Quest ATR accessory (diamond puck). Spectra were recorded of either thin films or solids, with characteristic absorption wavenumbers (ν_{max}) reported in cm^{-1}.

^{1}H, ^{13}C{^{1}H}, and ^{11}B{^{1}H} NMR spectra were acquired on either a Bruker AV400 with a BBFO probe (^{1}H 400 MHz; ^{13}C{^{1}H} 101 MHz), a Bruker AVII 400 with a BBFO probe (^{1}H 400 MHz; ^{13}C{^{1}H} 101 MHz; ^{11}B{^{1}H} 128 MHz), a Bruker AVIII-HD 500 with a SmartProbe BBFO+ probe (^{1}H 500 MHz, ^{13}C{^{1}H} 126 MHz), a Bruker AVIII 500 with a CryoProbe Prodigy BBO probe (^{1}H 500 MHz, ^{13}C{^{1}H} 126 MHz, ^{11}B{^{1}H} 160 MHz), or a Bruker AVIII-HD 700 with a CryoProbe Prodigy TCI probe (^{1}H 700 MHz) in the deuterated solvent stated. Deuterated solvents were purchased and used as received. All chemical shifts are quoted in parts per million (ppm) relative to the residual solvent peak. All coupling constants *J* are quoted

in Hz. Multiplicities are indicated as s (singlet), d (doublet), t (triplet), q (quartet), m (multiplet), and multiples thereof. The abbreviation Ar denotes aromatic, app denotes apparent and br denotes broad. NMR peak assignments were confirmed using 2D ^{1}H correlated spectroscopy (COSY), 2D ^{1}H–^{13}C heteronuclear multiple-bond correlation spectroscopy (HMBC), and 2D ^{1}H–^{13}C heteronuclear single quantum coherence (HSQC) where necessary. Carbon signals of a quaternary carbon directly connected to a boron atom are frequently not observed in $^{13}C\{^{1}H\}$ NMR due to the slow relaxation time. Compounds **15–18, 24, 26, 27, 30–32, 34, 36–42, 46–52, 55, 56, 58, 60, 62, 64, 66, 67, 68** observe no quaternary carbons connected to the boron atom. Compounds **25**, **28, 29, 33, 45, 61, 63, 65, 70** observe one quaternary carbon. $^{11}B\{^{1}H\}$ recorded for ester products often required extended scans (*ca*. 500) to observe a signal. $^{11}B\{^{1}H\}$ recorded at 160 MHz required correction due to the broad signal observed as a result of the NMR tube borosilicate glass. The spectra measured at 160 MHz shown in this experimental section were corrected using the 'Arithmetic' Mestrenova function (an example of this is shown below in Figure 2).

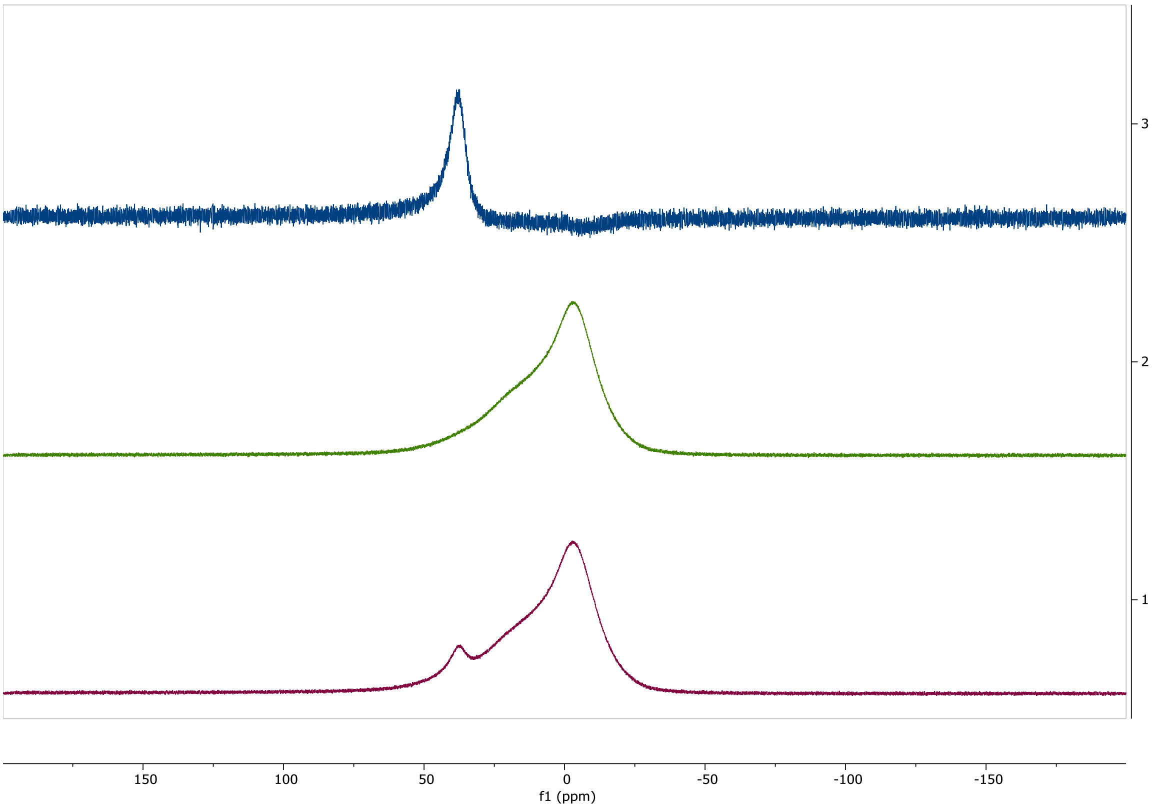

Figure 2: Example of $^{11}B\{^{1}H\}$ 160 MHz spectra, with removal of borosilicate background. 1) spectrum of sample in $CHCl_3$. 2) spectrum of just $CHCl_3$ showing NMR tube background. 3) sample minus NMR tube background

Mass spectrometry (m/z) data were acquired either by electrospray ionisation (ESI) or electron ionisation (EI) at the University of St Andrews Mass Spectrometry Facility or at the University of Edinburgh Mass Spectrometry Facility.

All azaborine materials were stored at –10 °C to avoid degradation.

1.1. Selectivity factor and conversion calculations

The selectivity factors (*s*) and conversions (c) were calculated using the equations below, with the enantiomeric excesses (ee) determined by HPLC analysis on a chiral stationary phase. Reference 85 provides the derivation and alternative forms of these equations.[85]

Equation 1: $s = \frac{ln[(1-conv)(1-ee_{alcohol})]}{ln[(1-conv)(1+ee_{alcohol})]}$

Equation 2: $c = \frac{ee_{alcohol}}{ee_{alcohol}+ee_{ester}}$

2. Kinetic Resolution Optimisation

2.1. Solvent optimisation

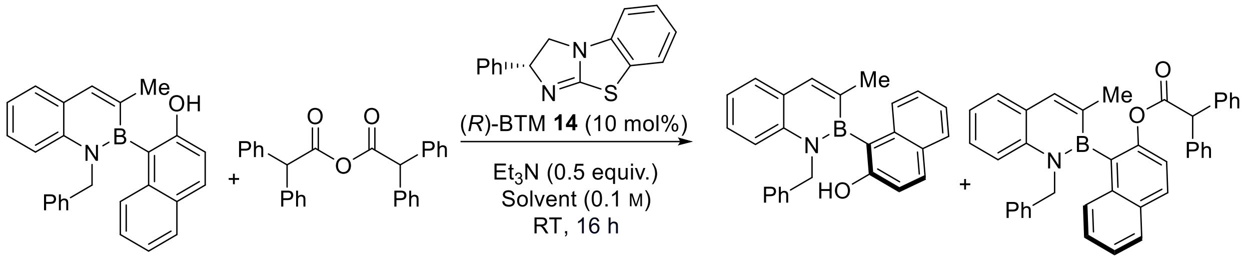

Entry	Solvent	**(*R*)-Alcohol 15 NMRY (%)**	**(*R*)-Alcohol 15 er**	**(*S*)-Ester 16 NMRY (%)**	**(*S*)-Ester 16 er**	**Conversion**	**Selectivity factor**
1	$CHCl_3$	43	85:15	53	81:19	53	9
2	Et_2O	50	93:7	50	73:27	65	7
3	PhMe	57	67:33	33	86:14	32	9
4	CH_2Cl_2	46	78:22	49	78:22	50	6
5	EtOAc	40	73:27	46	72:28	51	4
6	THF	50	66:34	50	67:33	49	3
7	MeCN	42	79:21	51	79:21	50	7
8	Acetone	43	75:25	45	77:23	48	5
9	DCE	44	74:26	41	80:20	44	6
10	DMF	55	74:26	42	78:22	46	6

Table 1: Solvent optimisation. On a 0.2 mmol scale. Yields are NMR yields, determined by 1,4-dinitrobenzene as the internal standard. Enantiomeric ratios determined by HPLC with a chiral stationary phase.

2.2. Catalyst optimisation

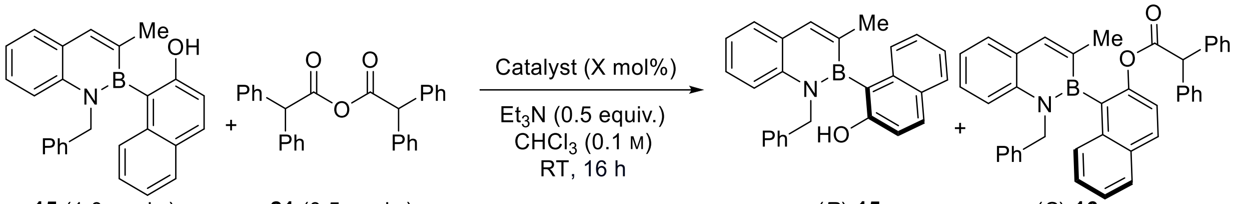

15 (1.0 equiv.) **21** (0.5 equiv.) (*R*)-**15** (*S*)-**16**

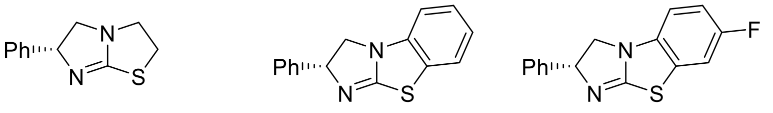

(*R*)-TM **20** (*R*)-BTM **14** (*R*)-F-BTM **19**

Entry	Catalyst (mol%)	**(*R*)-Alcohol 15 NMRY (%)**	**(*R*)-Alcohol 15 er**	**(*S*)-Ester 16 NMRY (%)**	**(*S*)-Ester 16 er**	**Conversion**	**Selectivity factor**
1	(*R*)-**14** (10)	43	85:15	53	81:19	53	9
2	(*R*)-**20** (10)	62	72:28	41	84:16	39	8
3	(*R*)-**14** (5)	56	78:22	49	85:15	44	10
4	(*R*)-**14** (2.5)	53	78:22	49	84:16	45	9
5	(*R*)-**14** (1.0)	51	83:17	49	83:17	50	10
6	(*R*)-**14** (0.1)	79	54:46	15	77:23	13	4
7	(*R*)-**19** (5)	65	82:18	38	85:15	48	11
8	(*R*)-**19** (2.5)	55	80:20	46	87:13	45	12
9	(*R*)-**19** (1.0)	45	78:22	37	87:13	43	12
10[a]	None	100	-	N.R	-	-	-
11[b]	None	90	-	7	-	-	-

Table 2: Catalyst optimisation. On a 0.2 mmol scale. Yields are NMR yields, determined by 1,4-dinitrobenzene as the internal standard. Enantiomeric ratios determined by HPLC with a chiral stationary phase. a) without base. b) with base.

2.3. Base optimisation

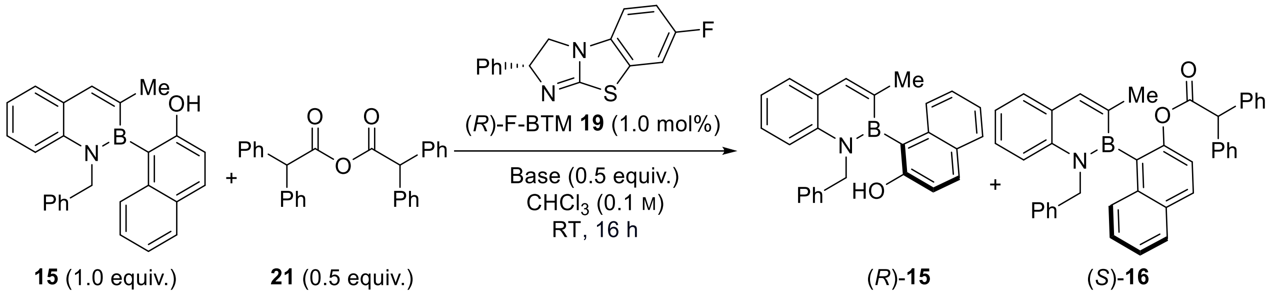

Entry	Base	(*R*)-Alcohol 15 NMRY (%)	(*R*)-Alcohol 15 er	(*S*)-Ester 16 yield NMRY (%)	(*S*)-Ester 16 er	Conversion	Selectivity factor
1	Et_3N	45	78:22	37	87:13	43	12
2	Pyridine	50	56:44	50	57:43	46	2
3	DIPEA	50	73:27	35	88:12	38	12
4	Na_2CO_3	85	51:49	4	78:22	3	4
5	DBU	45	55:45	46	45:55	50	2
6	TMP	50	72:28	40	81:19	42	7
7	NaOAc	96	52:48	5	92:8	5	12
8	Quinaldine	74	52:48	14	N/A	N/A	N/A
9	DABCO	55	65:35	40	76:24	37	4
10	Lutidine	76	52:48	10	75:25	N/A	N/A

Table 3: Base optimisation. On a 0.2 mmol scale. Yields are NMR yields, determined by 1,4-dinitrobenzene as the internal standard. Enantiomeric ratios determined by HPLC with a chiral stationary phase.

2.4. Temperature optimisation

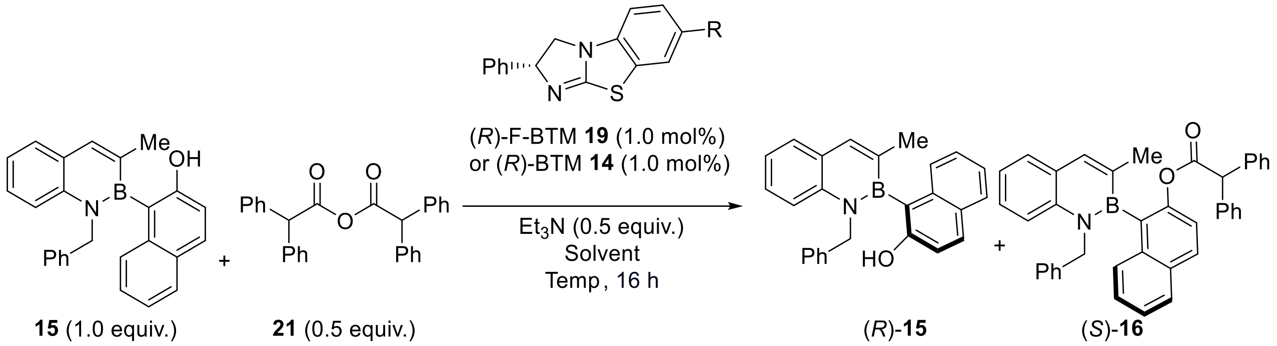

Entry	Catalyst	Temp (°C)	Solvent (M)	(*R*)-Alcohol 15 NMRY (%)	(*R*)-Alcohol 15 er	(*S*)-Ester 16 NMRY (%)	(*S*)-Ester 16 er	Conversion	Selectivity factor
1	(*R*)-**19**	0	$CHCl_3$ (0.1)	50	80:20	38	89:11	43	17
2	(*R*)-**14**	0	$CHCl_3$ (0.1)	50	84:16	45	88:12	47	14
3	(*R*)-**19**	–20	$CHCl_3$ (0.1)	50	84:16	41	95:5	43	38
4	(*R*)-**14**	–20	$CHCl_3$ (0.1)	51	84:16	42	94:6	44	34
5	(*R*)-**19**	–50	$CHCl_3$ (0.1)	70	71:29	25	97:3	31	48
6	(*R*)-**14**	–50	$CHCl_3$ (0.1)	50	85:15	40	96:4	43	53
7	(*R*)-**14**	–75	CH_2Cl_2 (0.1)	N.R	-	-	-	-	-
8[a]	(*R*)-**14**	–75	Et_2O (0.1)	N.R	-	-	-	-	-
9[a]	(*R*)-**14**	–75	PhMe (0.1)	N.R	-	-	-	-	-
10[a]	(*R*)-**14**	–75	Et_2O : $CHCl_3$ (0.2 : 0.2)	90	51:49	8	N/A	N/A	N/A
11	(*R*)-**14**	–55	$CHCl_3$ (0.1)	50	79:21	40	97:3	39	68
12	(*R*)-**14**	–55	$CHCl_3$ (0.05)	60	78:22	42	94:6	39	28
13	(*R*)-**14**	–55	$CHCl_3$ (0.2)	73	74:26	35	97:3	34	54

Table 4: Temperature optimisation. On a 0.2 mmol scale. Yields are NMRY determined by 1,4-dinitrobenzene as the internal standard. Enantiomeric ratio determined on HPLC with chiral stationary phase. a) reacted for 24 h.

2.5. Stoichiometry optimisation

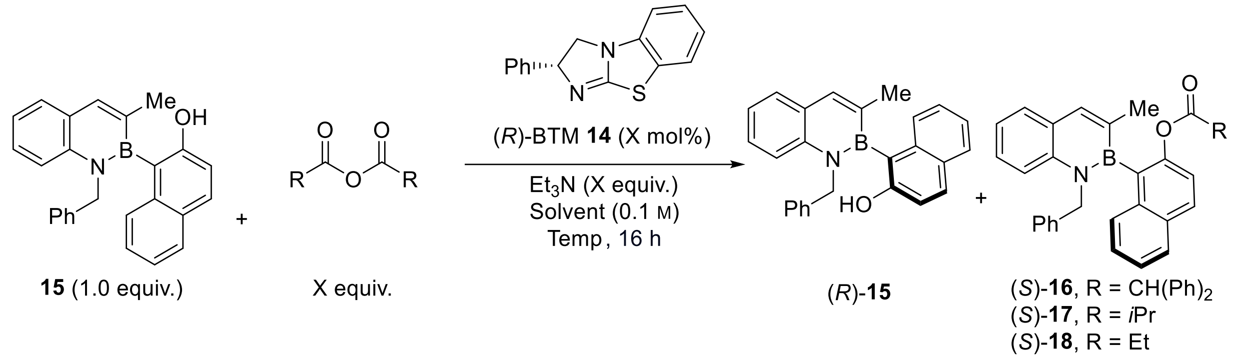

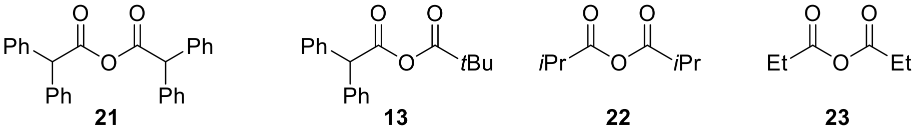

Entry	Anhydride (equiv.)	Temp (°C)	(*R*)-14 (mol%)	Base (equiv.)	(*R*)-Alcohol 15 NMRY (%)	(*R*)-Alcohol 15 er	(*S*)-Ester yield (%)	(*S*)-Ester er	Conversion	Selectivity factor
1	**22** (0.5)	RT	1.0[a]	0.5	(58)	71:29	(48)	69:31	52	3
2	**21** (0.5)	–55	1.0	0.5	50	79:21	40	97:3	39	68
3	**21** (0.6)	–55	1.0	0.6	74	70:30	32	98:2	30	69
4	**21**(0.5)	–55	1.0	0.3	66	74:26	33	97:3	34	58
5	**21** (0.5)	–55	2.5	0.5	55	85:15	45	97:3	43	62
6	**21** (0.5)	–55	5.0	0.5	50	85:15	40	97:3	42	68
7	**21** (0.7)	–55	5.0	0.5	60	80:20	40	98:2	39	78
8	**13** (0.7)	–55	5.0	0.5	57	77:23	40	98:2	36	87
9	**13** (0.7)	–55	10.0	0.5	50	82:18	40	97:2	41	54
10	**13** (0.8)	–55	5.0	0.5	60	86:14	45	96:4	44	57
11	**13** (0.8)	–55	5.0	0.7	40	98:2	55	90:10	55	35
12	**13** (0.7)	–55	5.0	0.6	66	90:10	50	95:5	47	55
**13[b]**	**13 (0.7)**	**–55**	**5.0**	**0.6**	**(37)**	**98:2**	**(34)**	**93:7**	**53**	**43**
14	**23** (0.7)	–55	5.0	0.6	52	67:33	33	70:30	45	3
15	**13** (0.7)	–40	5.0	0.6	50	91:9	49	94:6	48	44
16	**13** (0.7)	–40	2.5	0.6	48	93:7	50	93:7	50	37

*Table 5: Stoichiometry optimisation. Yields are NMRY determined by 1,4-dinitrobenzene as the internal standard, with isolated yields in parentheses. Enantiomeric ratio determined on HPLC with chiral stationary phase a) Using (R)-*F-BTM **19** *b) performed on a 0.4 mmol scale with isolated yields provided.*

3. Homologated Alcohol Optimisation

3.1. Catalyst and temperature optimisation

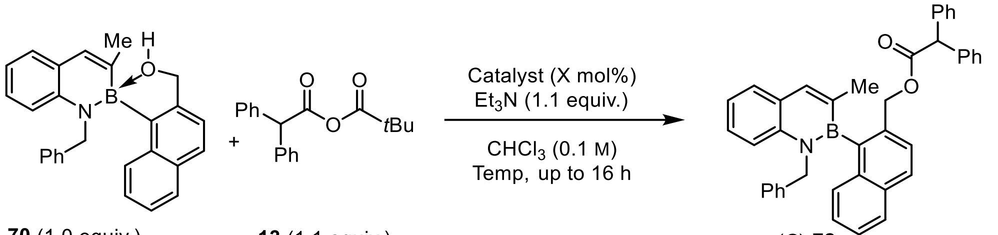

70 (1.0 equiv.) **13** (1.1 equiv.) (*S*)-**72**

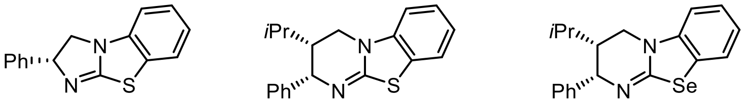

(*R*)-BTM **14** (2*S*,3*R*)-HyperBTM **S1** (2*S*,3*R*)-HyperSe **71**

Entry	Catalyst (mol%)	Temp (°C)	Time (h)	(*S*)-Ester 72 NMRY (%)	(*S*)-Ester 72 er
1	(*R*)-**14** (5)	RT	4	(34)	51:49
2	(2*S*,3*R*)-**S1** (10)	RT	1.5	(45)	55:45
3	(2*S*,3*R*)-**71** (10)	RT	1.5	(63)	57:43
4	(2*S*,3*R*)-**71** (10)	–55	16	76	54:46
5	(2*S*,3*R*)-**71** (5)	–55	16	42	54:46
6	(2*S*,3*R*)-**71** (10)	55	1	60	47:43
7	None	RT	6	N.R.	N/A

Table 6: Catalyst and temperature optimisation. Yields are NMRY determined by 1,4-dinitrobenzene as the internal standard, with isolated yields in parentheses. Enantiomeric ratio determined on HPLC with chiral stationary phase.

3.2. Base and solvent optimisation

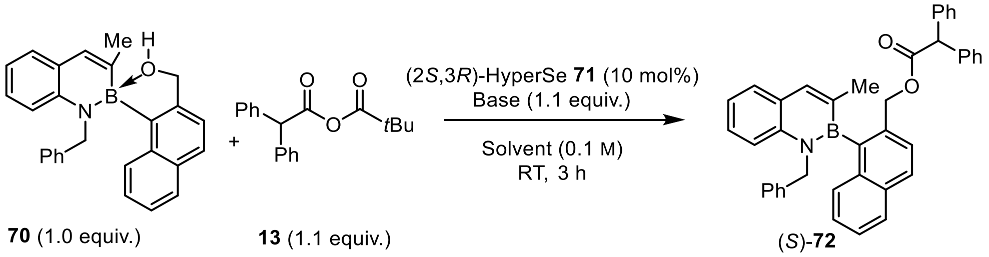

Entry	Base	Solvent (0.1 M)	(*S*)-Ester 72 NMRY (%)	(*S*)-Ester 72 er
1	Et_3N	$CHCl_3$	(63)	57:43
2	2,6-lutidine	$CHCl_3$	6	57:43
3	DBU	$CHCl_3$	59	58:42
4	$NaHCO_3$	$CHCl_3$	78	56:44
5	None	$CHCl_3$	71	55:45
6	Et_3N	PhMe	(59)	57:43
7	Et_3N	DMM	92	56:44
8	Et_3N	EtOAc	84	55:45
9	Et_3N	THF	93	56:44
10	Et_3N	Et_2O	93	55:45
11	Et_3N	MeCN	84	55:45

Table 7: Base and solvent optimisation. Yields are NMRY determined by 1,4-dinitrobenzene as the internal standard, with isolated yields in parentheses. Enantiomeric ratio determined on HPLC with chiral stationary phase.

4. Dynamic Kinetic Resolution Optimisation

4.1. Catalyst optimisation

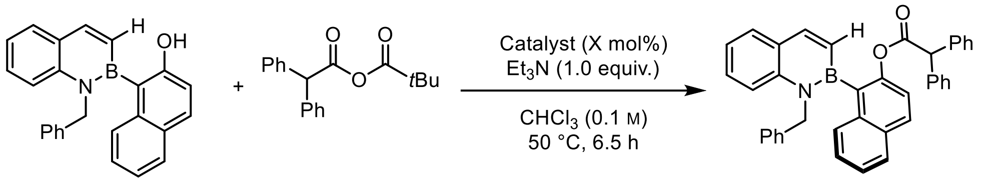

24 (1.0 equiv.) **13** (1.0 equiv.) (*S*)-**25**

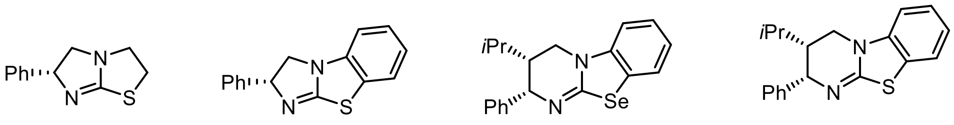

(*R*)-TM **20** (*R*)-BTM **14** (2*S*,3*R*)-HyperSe **71** (2*S*,3*R*)-HyperBTM **S1**

Entry	Temp (°C)	Catalyst (mol%)	(*S*)-Ester 25 NMRY (%)	(*S*)-Ester 25 er
1	50	(*R*)-**14** (5.0)	73	83:17
2	50	(*R*)-**14** (2.5)	70	83:17
3	50	(*R*)-**14** (1.0)	65	79:21
4	50	(R)-**20** (2.5)	63	76:24
5	50	(2*S*,3*R*)-**71** (2.5)	77	72:28
6	50	(2*R*,3*S*)-**S1** (2.5)	65	72:29
7	57	(*R*)-**14** (2.5)	75	78:22
8	40	(*R*)-**14** (2.5)	71	75:25
9[a]	50	(*R*)-**14** (2.5)	77	78:22

Table 8: Catalyst optimisation. Yields are NMRY determined by 1,4-dinitrobenzene as the internal standard. Enantiomeric ratio determined on HPLC with chiral stationary phase a) Using 1.1 equiv. anhydride.

4.2. Solvent and base optimisation

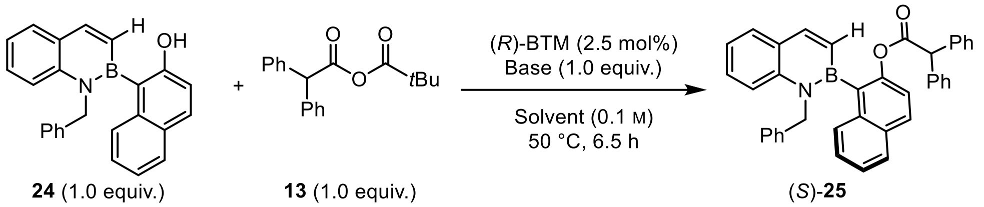

Entry	Solvent[a]	Base	(*S*)-Ester 25 NMRY (%)	(*S*)-Ester 25 er
1	THF	Et_3N	44	79:21
2	EtOAc	Et_3N	70	72:28
3	PhMe	Et_3N	69	78:22
4	MeCN	Et_3N	82	55:45
5	1,4-dioxane	Et_3N	25	81:19
6	Acetone	Et_3N	58	72:28
7	$CHCl_3$ (0.2)	Et_3N	75	79:21
8	$CHCl_3$ (0.05)	Et_3N	67	83:17
9	$CHCl_3$	DIPEA	65	82:18
10	$CHCl_3$	Pyridine	53	77:23
11	$CHCl_3$	Na_2CO_3	50	85:15
12	$CHCl_3$	DBU	88	51:49
13	$CHCl_3$	DIPA	74	83:17
14	$CHCl_3$	TMP	78	78:22
15	$CHCl_3$	DABCO	77	79:21
16[b]	$CHCl_3$	Et_3N	84	79:21

Table 9: Solvent and base optimisation. Yields are NMRY determined by 1,4-dinitrobenzene as the internal standard. Enantiomeric ratio determined on HPLC with chiral stationary phase. a) Unless otherwise specified, using 0.1 M solvent. b) Using 1.3 equiv. anhydride, for 16 h.

5. General Procedures

General Procedure **A**: Wittig reaction - synthesis of bromostyrenes

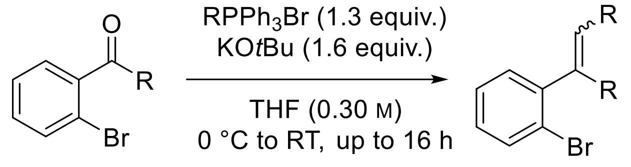

Following literature[86] and under nitrogen, the appropriate alkyl triphenylphosphonium bromide (1.3 equiv.) and KO*t*Bu (1.6 equiv.) were stirred in THF (0.30 M) for 2 h. The solution was cooled to 0 °C in an ice bath and the appropriate carbonyl (1.0 equiv.) was added dropwise, then the mixture was warmed to RT and stirred for up to 16 h. The crude mixture was concentrated under reduced pressure to remove THF and the concentrate was stirred in pentane for 30 mins. The mixture was then filtered with pentane and the filtrate was concentrated under reduced pressure to afford the crude product which was purified as specified to give an unresolved mixture of *Z* and *E* isomers.

*Note the isomer mixture is inconsequential to General Procedure **B***

General Procedure **B**: Buchwald Hartwig

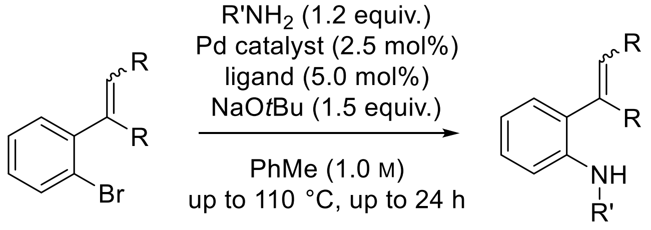

Following literature[86] and under nitrogen, a palladium catalyst (5.0 mol%), a ligand (2.5 mol%) and NaO*t*Bu (1.5 equiv.) were backfilled and stirred in PhMe (1.0 M). The appropriate bromostyrene (1.0 equiv.) followed by the appropriate primary amine (1.2 equiv.) were added dropwise and the mixture was sparged with nitrogen for 15 mins. The solution was then heated to the specified temperature and stirred for up to 24 h, then cooled to RT and filtered with Et_2O through a celite pad. The filtrate was concentrated under reduced pressure to afford the crude product which was purified as specified to give an unresolved mixture of *Z* and *E* isomers.

*Note the isomer mixture is inconsequential to General Procedure **C***

General Procedure **C**: Dewar synthesis

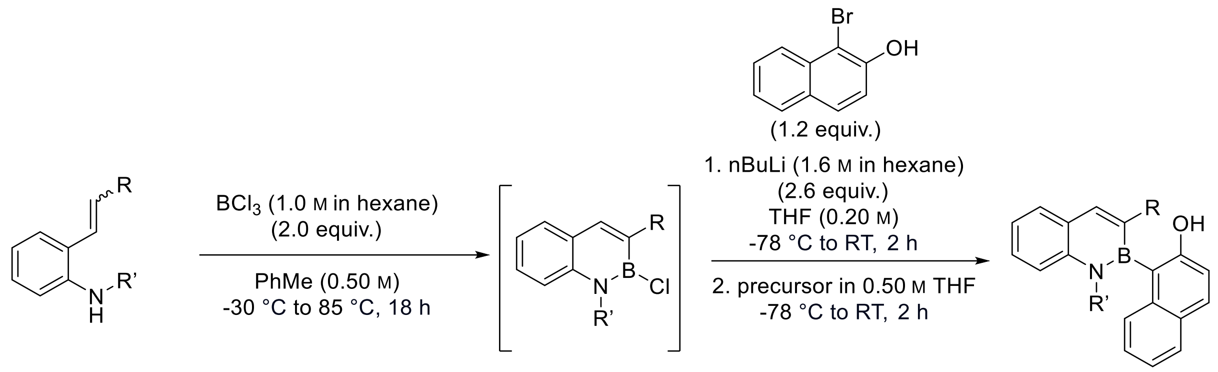

Following literature[38] and under nitrogen, the appropriate aminostyrene (1.0 equiv.) was stirred in PhMe (0.50 M) and set to – 30 °C. BCl_3 (1.0 M in hexane, 2.0 equiv.) was added dropwise and the mixture was allowed to warm to RT and stirred for 1 h, then heated to 85 °C for 17 h. The mixture was then cooled to RT and concentrated under reduced pressure to afford the intermediate chlorinated species which was diluted with THF (0.50 M) and used immediately in the next step.

Under nitrogen, the appropriate 1-bromo-2-naphol species (1.1 equiv.) was dissolved in THF (0.20 M) and set to –78 °C. nBuLi (1.6 M in hexane or 2.1 M in hexane, 2.6 equiv.) was added dropwise and the mixture was stirred at –78 °C for 1 h, then allowed to warm to RT. The solution was then cooled back to –78 °C and the intermediate chlorinated species in THF (0.50 M) was added dropwise with stirring. The solution was stirred at –78 °C for 1 h, then allowed to warm to RT and stirred for a further 1 h, then quenched with aq. NH_4Cl and concentrated under reduced pressure to remove THF. The solution was then diluted with EtOAc and the organic layer was extracted, dried (Na_2SO_4), and concentrated under reduced pressure to afford the crude product which was purified as specified.

Note the azaborine synthesis often obtained products in modest yields due to formation of multiple side products, including 2-napthol. Often two purifications were required to obtain pure azaborine material. Furthermore, the BCl_3 step requires a minimum of 85 ℃ for efficient cyclisation and BCl_3 in hexane solution rather than in CH_2Cl_2 is recommended for this.

General Procedure **D**: Racemic kinetic resolution catalysis

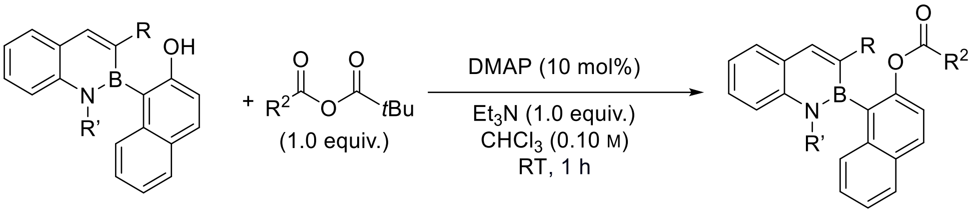

The appropriate azaborine (1.0 equiv.), anhydride (1.0 equiv.), with DAMP (10 mol%) and Et_3N (1.0 equiv.) were stirred in $CHCl_3$ (0.10 M) at RT for 1 h. The mixture was diluted with aq. HCl (1.0 M) and the organic layer was extracted with $CHCl_3$, dried (Na_2SO_4) and concentrated under reduced pressure to afford the crude product which was purified by Biotage® Selekt™.

General Procedure **E**: Enantioselective kinetic resolution catalysis

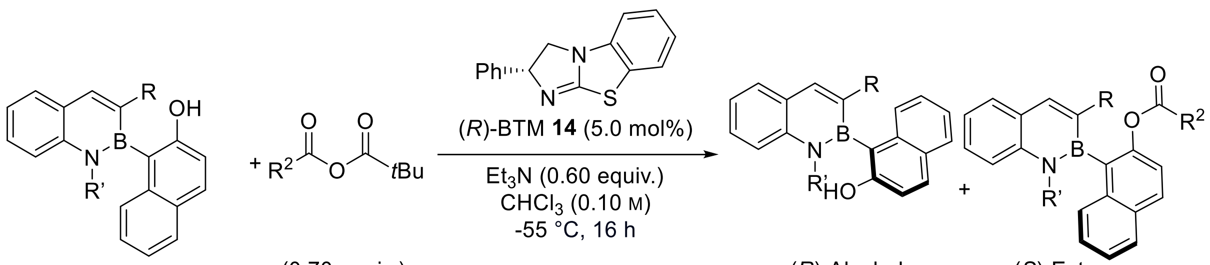

(0.70 equiv.) (*R*)-Alcohol (*S*)-Ester

The appropriate azaborine (1.0 equiv.) and anhydride (0.70 equiv.) in $CHCl_3$ (0.20 M) were cooled in a Schlenk tube to – 55° C. (*R*)-BTM **14** (5.0 mol%) and Et_3N (0.60 equiv.) in $CHCl_3$ (0.20 M) were cooled in a vial to – 55° C then added to the azaborine mixture. The solution was stirred at – 55° C for 16 h, then quenched with HCl (3.0 M in MeOH or 2.0 N in Et_2O, followed by 1.0 M in H_2O), concentrated under reduced pressure to remove $CHCl_3$ and extracted with EtOAc. The organic layer was dried (Na_2SO_4) and concentrated under reduced pressure to afford the crude product which was purified as specified to afford the alcohol and ester products.

General Procedure **F**: Enantioselective dynamic kinetic resolution catalysis

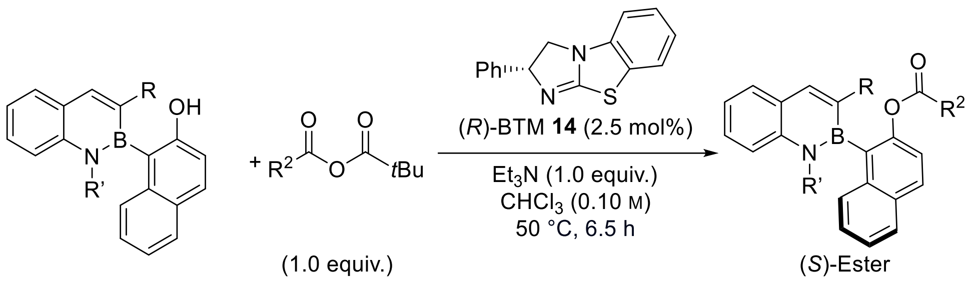

The appropriate azaborine (1.0 equiv.) and anhydride (1.0 equiv.) with (*R*)-BTM **14** (2.5 mol%) and Et_3N (1.0 equiv.) were stirred in $CHCl_3$ (0.10 M) at 50 °C for 6.5 h, then quenched with aq. HCl (1.0 M). The organic layer was extracted with $CHCl_3$, dried (Na_2SO_4) and concentrated under reduced pressure to afford the crude product which was purified as specified.

6. Compound Synthesis

6.1. Starting material synthesis

6.1.1. Anhydride synthesis

2,2-diphenylacetic anhydride **21**

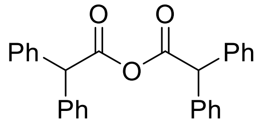

Following literature[87] and under nitrogen, DCC (2.1 g, 10 mmol, 1.0 equiv.) was dissolved in CH_2Cl_2 (63 mL). Diphenyl acetic acid (4.2 g, 20 mmol, 2.0 equiv.) was added and the mixture was stirred at RT for 2 h, then concentrated under reduced pressure to remove CH_2Cl_2. The resulting residue was dissolved in Petrol : EtOAc (70:30 mL), filtered and the filtrate was washed with $NaHCO_3$, brine, dried (Na_2SO_4) and concentrated under reduced pressure to afford the title compound **21** (3.1 g, 75%) as a white solid with all spectroscopic data in accordance with the literature. **mp** 95–98 °C {Lit.[87] 87–89 °C}; **IR** ν_{max} (film) 2936, 1800, 1454, 1059; **^{1}H NMR** (400 MHz, $CDCl_3$) δ_H: 5.03 (2H, s), 7.16–7.18 (8H, m), 7.27–7.30 (12H, m).

2,2-diphenylacetic pivalic anhydride **13**

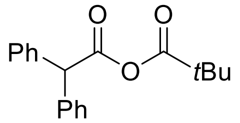

Following literature[88] and under nitrogen, diphenyl acetic acid (1.1 g, 5.0 mmol, 1.0 equiv.) and Et_3N (0.91 mL, 6.5 mmol, 1.3 equiv.) were dissolved in CH_2Cl_2 (17 mL) and set to 0 °C. Pivaloyl chloride (0.74 mL, 6.0 mmol, 1.2 equiv.) was added dropwise and the reaction was stirred at 0 °C for 1 h. The mixture was diluted with Et_2O, filtered and the filtrate was concentrated under reduced pressure to afford the title compound **13** (1.3 g, 86%) an off-white solid with all spectroscopic data in accordance with the literature.[56] **mp** 68–71 °C; **IR** ν_{max} (solid) 2978, 1800, 1738, 1454, 1042; **^{1}H NMR** (400 MHz, $CDCl_3$) δ_H: 1.12 (9H, s), 5.13 (1H, s), 7.27–7.37 (10H, m).

6.1.2. Styrene Synthesis

1-bromo-2-vinylbenzene **S2**

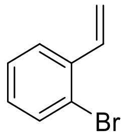

Following general procedure **A**, bromo(methyl)triphenyl-λ^5-phosphane (4.6 g, 13.0 mmol), KO*t*Bu (1.7 g, 15 mmol) in THF (33 mL) for 2 h, then 2-bromobenzaldehyde (1.2 mL, 10 mmol) for 20 h gave, after purification by Biotage® Selekt™ (Sfär HC 100 g, 180 mL.min^{-1}, Hexane [100% 15 CV]) the title compound **S2** (0.95 g, 52%) as a colourless oil with all spectroscopic data in accordance with the literature.[89] **IR** ν_{max} (film) 2924, 1466, 1026, 760; **^{1}H NMR** (500 MHz, $CDCl_3$) δ_H: 5.37 (1H, d, *J* 11.0), 5.70 (1H, d, *J* 17.4), 7.06 (1H, dd, *J* 17.4, 11.0), 7.11 (1H, dt, *J* 7.5, 1.5), 7.28 (1H, t, *J* 7.6), 7.55 (2H, app d, *J* 8.0).

1-bromo-2-(prop-1-en-1-yl)benzene **S3**

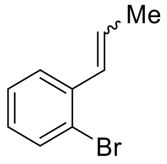

Following general procedure **A**, ethyltriphenylphosphonium bromide (7.4 g, 20 mmol), KO*t*Bu (2.6 g, 23 mmol) in THF (50 mL) for 2 h, then 2-bromobenzaldehyde (1.8 mL, 15 mmol) for 16 h gave, after purification by Biotage® Selekt™ (Sfär HC 100 g, 300 mL.min^{-1}, Hexane [100% 15 CV]) the title compound **S3** (3.5 g, *quant.*) as a colourless oil in a 9:1 mixture of (*E*) : (*Z*) isomers with all spectroscopic data in accordance with the literature.[90] **IR** ν_{max} (film) 2909, 1466, 1020, 961, 743, 667; **^{1}H NMR** ($CDCl_3$, 400 MHz) [*E* isomer] δ_H: 1.93 (3H, dd, *J* 6.7, 1.7), 6.19 (1H, qd, *J* 15.6, 6.7), 6.73 (1H, app dd, *J* 15.6, 1.7), 7.05 (1H, td, *J* 7.7, 1.7), 7.22–7.26 (1H, m), 7.47 (1H, dd, *J* 7.8, 1.7), 7.52 (1H, dd, *J* 8.0, 1.2); [*Z* isomer] δ_H: 1.79 (3H, dd, *J* 7.1, 1.8), 5.90 (1H, dq, *J* 11.5, 7.1), 6.48 (1H, app dd, *J* 11.5, 1.7), 7.59 (1H, dd, *J* 8.0, 0.9).

Note the missing Z isomer signals are hidden underneath E isomer signals

1-bromo-2-(but-1-en-1-yl)benzene **S4**

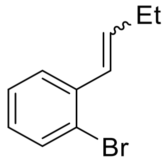

Following general procedure **A**, propyltriphenylphosphonium bromide (15.4 g, 40.0 mmol), KO*t*Bu (5.22 g, 47.0 mmol) in THF (100 mL) for 2 h, then 2-bromobenzaldehyde (3.50 mL, 30.0 mmol), for 17 h gave, after purification by Biotage® Selekt™ (Sfär HC 200 g, 300 mL.min^{-1}, Petrol [100% 10 CV]) the title compound **S4** (6.36, *quant.*)* as a colourless oil in a 7:3 mixture of (*Z*) : (*E*) isomers. **IR** ν_{max} (neat) 2928, 1466, 1433, 1022, 962, 748; **^{1}H NMR** (400 MHz, $CDCl_3$) [*Z* isomer] δ_H: 1.06 (3H, t, *J* 7.5), 2.22 (2H, pd, *J* 7.5, 1.7), 5.79 (1H, dt, *J* 11.4, 7.4), 6.45 (1H, dt, *J* 11.5, 1.7), 7.09–7.13 (1H, m), 7.23–7.31 (2H, m), 7.60 (1H, d, *J* 7.7); [*E* isomer] δ_H: 1.14 (3H, t, *J* 7.5), 2.26–2.34 (2H, m), 6.23 (1H, dt, *J* 15.7, 6.5), 6.74 (1H, dt, *J* 15.7, 1.7), 7.04–7.07 (1H, app m), 7.23–7.31 (2H, m), 7.50–7.55 (1H, m); **^{13}C{^{1}H} NMR** (101 MHz, $CDCl_3$) [*Z* isomer] δ_C: 14.4, 21.9, 124.2, 126.9, 128.0, 128.3, 130.7, 132.7, 135.8, 137.8; [*E* isomer] δ_C: 13.7, 26.3, 123.3, 127.4, 128.0, 128.2, 130.5, 132.8, 135.9, 137.8; **HRMS** (EI)$^+$ $C_{10}H_{11}{}^{79}Br$ [M]$^+$ found 210.0035, requires 210.0039 (–1.64 ppm).

*Despite repeat purifications including chromatography and distillation, the product is slightly impure.

Note the missing E isomer signals are hidden underneath Z isomer signals

1-bromo-2-(prop-1-en-2-yl)benzene **S5**

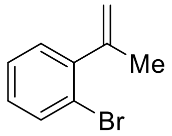

Following general procedure **A**, methyltriphenylphosphonium bromide (9.5 g, 27 mmol), KO*t*Bu (3.5 g, 31 mmol) in THF (70 mL) for 2 h, then 1-(2-bromophenyl)ethan-1-one (2.7 mL, 20 mmol) for 24 h gave, after purification by Biotage® Selekt™ (Sfär HC 200 g, 200 mL.min^{-1}, Petrol [100% 10 CV]), the title compound **S5** (1.4 g, 37%) as a colourless oil with all spectroscopic data in accordance with the literature.[91] **IR** ν_{max} (film) 3082, 2970, 1470, 1026, 903; **^{1}H NMR** (400 MHz, $CDCl_3$)δ_H: 2.10 (3H, dd, *J* 1.6, 0.9), 4.94 (1H, dd, *J* 1.9, 0.9), 5.23 (1H, p, *J* 1.6), 7.11 (1H, ddd, *J* 7.9, 7.2, 1.9), 7.20 (1H, dd, *J* 7.6, 1.9), 7.25–7.29 (1H, app m), 7.55 (1H, dd, *J* 8.0, 1.2).

1-bromo-2-(but-2-en-2-yl)benzene **S6**

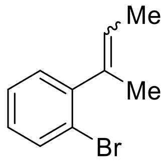

Following general procedure **A**, ethyltriphenylphosphonium bromide (10 g, 27 mmol), KO*t*Bu (3.5, 31 mmol) in THF (70 mL) for 2 h, then 2-bromoacetophenone (2.7 mL, 20 mmol) for 16 h gave, after purification by Biotage® Selekt™ (Sfär HC 200 g, 300 mL.min^{-1}, Hexane [100% 10 CV]) the title compound **S6** (3.9 g, 93%) as a colourless oil in a 4:1 mixture of (*E*) : (*Z*) isomers. **IR** ν_{max} (neat): 2978, 1470, 1439, 1022, 754; **^{1}H NMR** (400 MHz, $CDCl_3$) [*E* isomer] δ_H: 1.40 (3H, dq, *J* 6.8, 1.6), 1.97 (3H, p, *J* 1.6), 5.60 (1H, qq, *J* 6.8, 1.6), 7.08–7.13 (2H, m), 7.29 (1H, td, *J* 7.4, 1.2), 7.57–7.59 (1H, m); [*Z* isomer] δ_H: 1.77 (3H, dq, *J* 6.8, 1.1), 5.45 (1H, qq, *J* 6.8, 1.5), 7.13–7.16 (2H, m), 7.23 (1H, dd, *J* 7.4, 1.3), 7.53 (1H, dd, *J* 8.0, 1.3); **^{13}C{^{1}H} NMR** (101 MHz, $CDCl_3$) [*E* isomer] δ_C: 14.7, 24.5, 122.7, 123.3, 127.5, 128.2, 130.1, 132.8, 137.0, 143.2; [*Z* isomer] δ_C: 14.0, 17.4, 122.5, 125.2, 127.3, 128.0, 130.0; **HRMS** (EI)$^+$ $C_{10}H_{11}{}^{79}Br$ [M]$^+$ found 210.0034, requires 210.0039 (–2.06 ppm).

Note the missing Z isomer signals are hidden underneath E isomer signals

1-bromo-2-(prop-1-en-2-yl)benzene **S7**

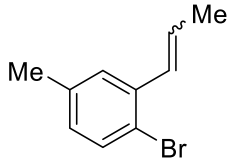

Following general procedure **A**, ethyltriphenylphosphonium bromide (12 g, 33 mmol), KO*t*Bu (4.4 g, 39 mmol), in THF (85 mL) for 2 h, then 2-bromo-5-methylbenzaldehyde (5.0 g, 25 mmol) for 5 h gave, after purification by Biotage® Selekt™ (Sfär HC 200 g, 200 mL.min^{-1}, Petrol : EtOAc, [100:0 to 95:5 15 CV]), the title compound **S7** (4.8 g, 91%) as a colourless oil in a 7:3 mixture of (*E*) : (*Z*) isomers with all spectroscopic data in accordance with the literature.[92] **IR** ν_{max} (film) 3036, 2914, 1653, 1466, 961; **^{1}H NMR** (400 MHz, $CDCl_3$) [*E* isomer] δ_H: 1.94 (3H, dd, *J* 6.7, 1.8), 2.31 (3H, s), 6.19 (1H, dq, *J* 15.6, 6.7), 6.72 (1H, dq, *J* 15.7, 1.8), 6.88 (1H, dd, *J* 8.2, 2.2), 7.30 (1H, d, *J* 2.2), 7.40 (1H, d, *J* 8.1); [*Z* isomer] δ_H: 1.81 (3H, dd, *J* 7.1), 2.33 (3H, s), 5.89 (1H, dq, *J* 11.4, 7.0), 6.47 (1H, dq, *J* 11.5, 2.0), 6.93 (1H, dd, *J* 8.2, 2.3), 7.13 (1H, d, *J* 2.3), 7.47 (1H, d, *J* 8.1).

2-bromo-4-methyl-1-(prop-1-en-1-yl)benzene **S8**

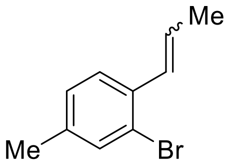

Following general procedure **A**, ethyltriphenylphosphonium bromide (10 g, 27 mmol), KO*t*Bu (3.5 g, 31 mmol) in THF (70 mL) for 2 h, then 2-bromo-4-methylbenzaldehyde (4.0 g, 20 mmol) for 16 h gave, after purification by Biotage® Selekt™ (Sfär HC 200 g, 300 mL.min^{-1}, Petrol [100% 10 CV]), the title compound **S8** (3.7 g, 85%) as a pale yellow oil in ~ a 1:1 mixture of (*Z*) : (*E*) isomers with all spectroscopic data in accordance with the literature.[93] **IR** ν_{max} (film) 2913, 1485, 1215, 1038; **^{1}H NMR** (400 MHz, $CDCl_3$) [*Z* isomer] δ_H: 1.78 (3H, dd, *J* 7.1, 1.8), 2.33 (3H, s), 5.86 (1H, dq, *J* 11.4, 7.1), 6.45 (1H, dd, *J* 11.5, 2.0), 7.03–7.09 (1H, m), 7.36 (1H, d, *J* 4.5), 7.35 (1H, s); [*E* isomer] δ_H: 1.91 (3H, dd, *J* 6.7, 1.8), 2.30 (3H, s), 6.14 (1H, dq, *J* 15.6, 6.7), 6.69 (1H, dq, *J* 15.6, 1.9), 7.19 (1H, d, *J* 7.8), 7.42 (1H, s).

Note the missing E isomer signals are hidden underneath Z isomer signals

6.1.3. Aminostyrene synthesis

N-benzyl-2-vinylaniline **S9**

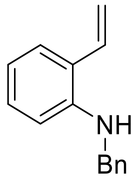

Following general procedure **B**, Pd(μ-Cl) dimer (7.1 mg, 0.019 mmol), John Phos (11.6 mg, 0.039 mmol), NaO*t*Bu (0.52 g, 5.5 mmol), 1-bromo-2-vinylbenzene **S2** (0.71 g, 3.9 mmol) and benzylamine (0.50 g, 4.7 mmol) in PhMe (8 mL) at 110 °C for 26 h gave, after purification by Biotage® Selekt™ (Sfär HC 50 g, 120 mL.min^{-1}, Hexane : EtOAc [100:0 to 85:15 20 CV, 85:15 to 70:30 10 CV]), the title compound **S9** (0.44 g, 54%) as a colourless oil with all the spectroscopic data in accordance with the literature.[86] **IR** ν_{max} (film) 3439, 3028, 1601, 1261; **^{1}H NMR** (500 MHz, $CDCl_3$) δ_H: 4.20 (1H, br s), 4.37 (2H, d *J* 4.6), 5.32 (1H, dd *J* 11.0, 1.6), 5.63 (1H, d *J* 17.3, 1.6), 6.65 (1H, d, *J* 8.2), 6.75 (1H, t, *J* 7.2), 6.80 (1H, dd, *J* 17.3, 11.0), 7.16 (1H, td, *J* 8.1, 1.6), 7.27–7.31 (2H, m), 7.34–7.40 (4H, m).

N-benzyl-2-(prop-1-en-1-yl)aniline **S10**

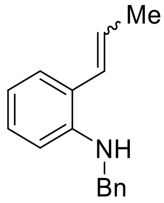

Following general procedure **B**, $Pd(OAc)_2$ (84.2 mg, 0.38 mmol), (±)-BINAP (0.47 g, 0.75 mmol), NaO*t*Bu (2.2 g, 19 mmol) 1-bromo-2-(prop-1-en-1-yl)benzene **S3** (2.9 g, 15 mmol) and benzylamine (2.0 mL, 18 mmol) in PhMe (15 mL) at 110 °C for 5 h gave, after purification by Biotage® Selekt™ (Sfär HC 100 g, 267 mL.min^{-1}, Hexane: EtOAc [100: 0 to 95:5 10 CV, 95:5 to 90:10 10 CV]), the title compound **S10** (1.2 g, 36%) as a yellow oil in a 10:1 mixture of (*E*) : (*Z*) isomers with all spectroscopic data in accordance with the literature.[94] **IR** ν_{max} (film) 3431, 2913, 1599, 1450, 960, 731; **^{1}H NMR** (400 MHz, $CDCl_3$) [*E* isomer] δ_H: 1.91 (3H, dd, *J* 6.6, 1.7), 4.16 (1H, br s), 4.38 (2H, d, *J* 5.51), 6.10 (1H, dq, *J* 15.5, 6.6), 6.42–6.47 (1H, m), 6.63 (1H, d, *J* 8.1), 6.71–6.75 (1H, m), 7.13 (1H, app t, *J* 8.5), 7.23 (1H, dd, *J* 7.5, 1.4), 7.29–7.33 (1H, m), 7.36–7.43 (4H, m); [*Z* isomer] δ_H: 1.77 (3H, dd, *J* 6.9, 1.8), 5.91 (1H, dq, *J* 11.2, 6.9), 6.33 (1H, app d, *J* 11.3).

Note the missing Z isomer signals are hidden underneath E isomer signals

N-benzyl-2-(but-1-en-1-yl)aniline **S11**

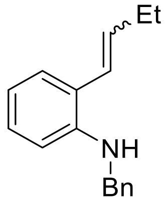

Following general procedure **B**, Pd(OAc)$_2$ (0.14 g, 0.63 mmol), (±)-BINAP (0.78 g, 1.3 mmol), NaO*t*Bu (4.3 g, 38 mmol), 1-bromo-2-(but-1-en-1-yl)benzene **S4** (5.3 g, 25 mmol) and benzylamine (3.3 mL, 30 mmol) in PhMe (25 mL) at 110 °C for 24 h gave, after purification by Biotage® Selekt™ (Sfär HC 200 g, 250 mL.min^{-1}, Petrol : EtOAc [100:0 to 90:10 10 CV, 90:10 to 75:25 10 CV, 75:25 to 50:50 10 CV]), the title compound **S11** (4.2 g, 70%)* as a yellow oil in a 3:1 mixture of (*Z*) : (*E*) isomers. **IR** ν_{max} (film) 3428, 2963, 1601, 1506, 1452, 748; **^{1}H NMR** (400 MHz, $CDCl_3$) [*Z* isomer] δ_H: 1.01 (3H, t, *J* 7.5), 2.18 (2H, pd, *J* 7.5, 1.6), 4.13 (1H, br s), 4.37 (2H, app d, *J* 5.5), 5.79 (1H, dt, *J* 11.2, 7.3), 6.26 (1H, d, *J* 11.2), 6.64 (1H, d, *J* 8.1), 6.72 (1H, td, *J* 6.6, 0.8), 7.07 (1H, d, *J* 7.4), 7.13 (1H, d, *J* 8.3), 7.27–7.32 (1H, m), 7.32–7.42 (4H, m); [*E* isomer] δ_H: 1.10 (3H, t, *J* 7.5), 2.21–2.29 (2H, m), 4.17 (1H, br s), 6.12 (1H, dt, *J* 15.6, 6.6), 6.42 (1H, d, *J* 15.6), 7.24 (1H, dd, *J* 7.6, 1.5); **^{13}C{^{1}H} NMR** (126 MHz, $CDCl_3$) [*Z* isomer] δ_C: 14.4, 22.2, 48.4, 110.3, 116.8, 123.2, 124.4, 127.3, 127.6, 128.2, 128.7, 129.6, 137.1, 139.6, 145.5; [*E* isomer] δ_C: 14.0, 26.6, 48.5, 110.9, 117.6, 124.5, 124.6, 127.3, 127.6, 135.7, 145.0; **HRMS** (ESI)$^+$ $C_{17}H_{20}N$ [M+H]$^+$ found 238.1588, requires 238.1590 (–1.05 ppm).

* Despite repeat purifications, the product is slightly impure.

Note the missing E isomer signals are hidden underneath Z isomer signals

N-benzyl-2-(prop-1-en-2-yl)aniline **S12**

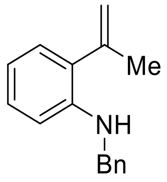

Following general procedure **B**, Pd(OAc)$_2$ (42.1 mg, 0.13 mmol), (±)-BINAP (0.20 g, 0.25 mmol), NaO*t*Bu (1.1 g, 7.5 mmol), 1-bromo-2-(prop-1-en-2-yl)benzene **S5** (1.1 g, 5.0 mmol) and benzylamine (0.70 mL, 6.0 mmol) in PhMe (5 mL) for 5 h gave, after purification by Biotage® Selekt™ (Sfär HC 50 g, 120 mL.min^{-1}, Petrol : EtOAc, [100:0 to 90:10 10 CV, 90:10 to 75:25 10 CV]), the title compound **S12** (0.70 g, 60%) as a pale yellow oil. **IR** ν_{max} (film) 3424, 3028, 2845, 1599, 1504, 1433, 1294, 903; **^{1}H NMR** (400 MHz, $CDCl_3$) δ_H: 2.09 (3H, t, *J* 1.2), 4.36 (2H, d, *J* 5.6), 4.54 (1H, t, *J* 5.6), 5.09 (1H, dd, *J* 2.3, 1.0), 5.30 (1H, app t, *J* 1.9), 6.62 (1H, dd, *J* 8.3, 1.0), 6.71 (1H, td, *J* 7.4, 1.1), 7.05 (1H, dd, *J* 7.5,

1.6), 7.12 (1H, td, *J* 7.6, 1.6), 7.27–7.31 (1H, m), 7.33–7.36 (4H, m); **^{13}C{^{1}H} NMR** (126 MHz, $CDCl_3$) δ_C: 24.2, 48.4, 110.7, 116.0, 116.9, 127.2, 127.4, 128.0, 128.3, 128.8, 129.5, 139.7, 143.7, 144.3; **HRMS** $(ESI)^+$ $C_{16}H_{18}N$ $[M+H]^+$ found 224.1431, requires 224.1434 (–1.12 ppm).

N-benzyl-2-(but-2-en-2-yl)aniline **S13**

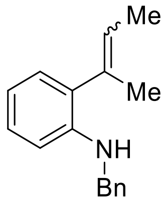

Following general procedure **B**, $Pd(OAc)_2$ (84.2 mg, 0.38 mmol), (±)-BINAP (0.47 g, 0.75 mmol), Na*Ot*Bu (2.2 g, 23 mmol), 1-bromo-2-(but-2-en-2-yl)benzene **S6** (3.2 g, 15 mmol), benzylamine (2.0 mL, 18 mmol) in PhMe (15 mL) at 110 °C for 24 hours gave, after purification by Biotage® Selekt™ (Sfär HC 100 g, 200 mL.min^{-1}, Cyclohexane : EtOAc [100:0 to 90:10 10 CV, 90:10 to 75:25 10 CV, 75:25 to 50:50 10 CV]), the title compound **S13** (2.9 g, 80%) as an orange oil in a 86:14 mixture of (*E*) : (*Z*) isomers. **IR** ν_{max} (film) 3422, 2911, 1578, 1504, 1319, 1028; **^{1}H NMR** (400 MHz, $CDCl_3$) [*E* isomer] δ_H: 1.49 (3H, dq, *J* 6.6, 1.6), 1.97 (3H, dq, *J* 3.2, 1.6), 4.28 (1H, br s), 4.37 (2H, s), 5.68 (1H, app dddt, *J* 8.2, 6.7, 5.2, 1.5), 6.63 (1H, d, *J* 8.2), 6.73 (1H, t, *J* 7.4), 6.96 (1H, app d, *J* 7.4), 7.13 (1H, t, *J* 7.7), 7.27–7.31 (1H, m), 7.35–7.37 (4H, m); [*Z* isomer] δ_H: 1.78 (3H, app d, *J* 6.8), 1.96–1.99 (3H, app m), 4.38 (1H, s), 5.59 (1H, dddt, *J* 8.4, 6.8, 5.1, 1.6), 6.99 (1H, d, *J* 7.5); **^{13}C{^{1}H} NMR** (101 MHz, $CDCl_3$) [*E* isomer] δ_C: 14.9, 24.9, 48.3, 110.3, 117.0, 124.2, 127.2, 127.4, 127.6, 127.9, 128.4, 128.7, 134.5, 140.0, 144.3; [*Z* isomer]; 14.1, 17.3, 48.3, 110.6, 125.1, 127.3, 127.8, 128.6; **HRMS** $(ESI)^+$ $C_{17}H_{20}N$ $[M+H]^+$ found 238.1587, requires 238.1590 (–1.2 ppm).

Note the missing Z isomer signals are hidden underneath E isomer signals

N-benzyl-4-methyl-2-(prop-1-en-1-yl)aniline **S14**

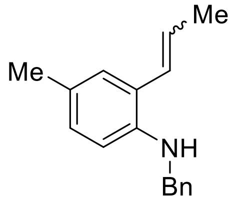

Following general procedure **B**, $Pd(OAc_2)$ (0.12 g, 0.5 mmol), (±)-BINAP (0.70 g, 1.1 mmol), Na*Ot*Bu (3.1 g, 32 mmol), 1-bromo-4-methyl-2-(prop-1-en-1-yl)benzene **S7** (4.5 g, 21 mmol), benzylamine (2.8 mL, 26 mmol) in PhMe (21 mL) at 110 °C for 4 h gave, after purification by Biotage® Selekt™ (Sfär HC 100 g, 200 mL.min^{-1}, Petrol : EtOAc [100:0

to 90:10 10 CV, 90:10 to 75:25 10 CV]), the title compound **S14** (3.8 g, 76%) as a yellow oil in ~6:4 mixture of (*E*) : (*Z*) isomers. **IR** ν_{max} (film) 3424, 3013, 2913, 1508, 1452, 1252, 966; **^{1}H NMR** (400 MHz, $CDCl_3$) [*E* isomer] δ_H: 1.94 (3H, dd, *J* 6.6, 1.8), 2.30 (3H, s), 4.06 (1H, br s), 4.38 (2H, s), 6.13 (1H, dq, *J* 15.5, 6.6), 6.48 (1H, dt, *J* 15.5, 1.8), 6.58–6.62 (1H, m), 6.96–7.01 (2H, m), 7.32–7.46 (5H, m); [*Z* isomer] δ_H: 1.81 (3H, dd, *J* 6.9, 1.8), 2.31 (3H, s), 4.06 (1H, br s), 4.38 (2H, s), 5.92 (1H, dq, *J* 11.2, 7.9), 6.36 (1H, dd, *J* 11.3, 2.0), 7.11 (1H, d, *J* 2.1); **^{13}C{^{1}H} NMR** (126 MHz, $CDCl_3$) [*E* isomer] δ_C: 19.1, 20.5, 48.7, 111.1, 124.7, 126.6, 126.8, 127.3, 127.6, 128.2, 128.6, 128.7, 130.2, 139.8, 142.7; [*Z* isomer] δ_C: 14.8, 20.6, 48.6, 110.6, 122.9, 125.8, 126.1, 127.2, 127.5, 128.3, 128.6, 128.7, 129.2, 139.9, 143.3; **HRMS** (ESI)$^+$ $C_{17}H_{20}N$ [M+H]$^+$ found 238.1588, requires 238.1590 (–1.09 ppm).

Note the missing Z isomer signals are hidden underneath E isomer signals

N-benzyl-5-methyl-2-(prop-1-en-1-yl)aniline **S15**

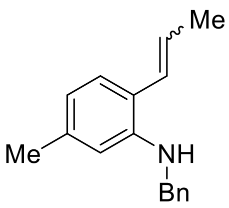

Following general procedure **B**, $Pd(OAc)_2$ (42.1 mg, 0.13 mmol), (±)-BINAP (0.20 g, 0.25 mmol), NaO*t*Bu (1.1 g, 7.5 mmol), 2-bromo-4-methyl-1-(prop-1-en-1-yl)benzene **S8** (1.1 g, 5.0 mmol), benzylamine (0.70 mL, 6.0 mmol) in PhMe (5 mL) at 110 °C for 4 h gave, after purification by Biotage® Selekt™ (Sfär HC 100 g, 180 mL.min^{-1} ,Petrol : EtOAc, [100:0 to 90:10 10 CV, 90:10 to 75:25 10 CV]), the title compound **S15** (1.0 g, 81%) as a yellow oil in a 4:1 mixture of (*Z*) : (*E*) isomers. **IR** ν_{max} (film) 3418, 2914, 1611, 1515, 1452, 1028, 816; **^{1}H NMR** (400 MHz, $CDCl_3$) [*Z* isomer] δ_H: 1.74 (3H, dd, *J* 6.9, 1.8), 2.29 (3H, s), 4.03 (1H, br s), 4.35 (2H, d, *J* 5.0), 5.84 (1H, dq, *J* 11.1, 6.9), 6.27 (1H, app d, *J* 11.0), 6.49 (1H, s), 6.55 (1H, d, *J* 7.5), 6.97 (1H, d, *J* 7.6), 7.33–7.41 (5H, m); [*E* isomer] δ_H: 1.87 (3H, dd, *J* 6.6, 1.7), 2.27 (3H, s), 4.08 (1H, br s), 6.03 (1H, dq, *J* 15.5, 6.6), 6.38 (1H, dd, *J* 15.4, 2.0), 6.46 (1H, s), 7.11 (1H, d, *J* 7.6), 7.27–7.31 (5H, m); **^{13}C{^{1}H} NMR** (126 MHz, $CDCl_3$) [*Z* isomer] δ_C: 14.7, 21.9, 48.4, 111.2, 117.6, 120.1, 125.9, 127.3, 127.7, 128.8, 129.1, 129.5, 138.0, 139.8, 145.6; [*E* isomer] δ_C: 19.0, 21.8, 48.6, 111.26, 118.4, 122.0, 126.6, 127.4, 127.5, 127.8, 127.9, 128.8, 144.9; **HRMS** (ESI)$^+$ $C_{17}H_{20}N$ [M+H]$^+$ found 238.1587, requires 238.1590 (–1.24 ppm).

Note the missing E isomer signals are hidden underneath Z isomer signals

N-methyl-2-(prop-1-en-1-yl)aniline **S16**

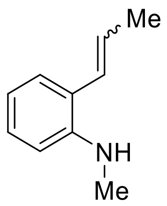

Following general procedure **B**, $Pd(OAc)_2$ (84.2 mg, 0.38 mmol), (±)-BINAP (0.47 g, 0.75 mmol), Na*Ot*Bu (2.2 g, 23 mmol), 1-bromo-2-(prop-1-en-1-yl)benzene **S3** (3.0 g, 15 mmol) and methylamine (2.0 M in THF, 15 mL, 30 mmol, 2.0 equiv.) in PhMe (15 mL) at 35 °C for 24 h gave, after purification by Biotage® Selekt™ (Sfär HC 100 g, 150 mL.min^{-1}, Petrol : EtOAc [100:0 to 75:25 15 CV, 75:25 to 50:50 10 CV]) the title compound **S16** (0.42 g, 19%) as a pale brown oil in a 4:1 mixture of (*E*) : (*Z*) isomers with all spectroscopic data in accordance with the literature.[95] **IR** ν_{max} (neat) 3435, 2912, 1601, 1504, 1165, 968; **^{1}H NMR** (400 MHz, $CDCl_3$) [*E* isomer] δ_H: 1.91 (3H, dd, *J* 6.6, 1.8), 2.88 (3H, s), 3.82 (1H, br s), 6.06 (1H, dq, *J* 15.5, 6.6), 6.40 (1H, dq, *J* 15.5, 2.0), 6.63 (1H, d, *J* 7.7), 6.71 (1H, td, *J* 7.5, 1.2), 7.19 (2H, app d, *J* 7.6); [*Z* isomer] δ_H: 1.74 (3H, dd, *J* 6.9, 1.8), 5.90 (1H, dq, *J* 11.2, 6.9), 6.25–6.29 (1H, app m).

Note the missing Z isomer signals are hidden underneath E isomer signals

N-ethyl-2-(prop-1-en-1-yl)aniline **S17**

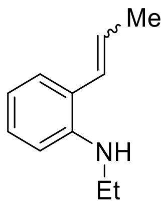

Following general procedure **B**, $Pd(OAc)_2$ (84.2 mg, 0.38 mmol), (±)-BINAP (0.47 g, 0.75 mmol), Na*Ot*Bu (2.2 g, 23 mmol), 1-bromo-2-(prop-1-en-1-yl)benzene **S3** (3.0 g, 15 mmol) and ethylamine (2.0 M in THF, 18 mL, 36 mmol, 2.4 equiv.) in PhMe (15 mL) at 60 °C for 24 h gave, after purification by Biotage® Selekt™ (Sfär HC 100 g, 200 mL.min^{-1}, Petrol : EtOAc [100: 0 to 90:10 15 CV, 90:10 to 75:25 10 CV]) the title compound **S17** (1.3 g, 52%) as a pale yellow oil in a 5:4 mixture of (*E*) : (*Z*) isomers with all spectroscopic data in accordance with the literature.[95] **IR** ν_{max} (film) 3420, 2967, 1601, 1504, 1454, 1315, 1258, 968; **^{1}H NMR** (400 MHz, $CDCl_3$) [*E* isomer] δ_H: 1.31 (3H, t, *J* 7.1), 1.91 (3H, dd, *J* 6.6, 1.8), 3.15–3.22 (2H, m), 3.65 (1H, br s), 6.06 (1H, dq, *J* 15.5, 6.6), 6.40 (1H, dt, *J*, 15.5, 1.8), 6.61–6.72 (2H, m), 7.13–7.15 (1H, m), 7.17–7.20 (1H, m); [*Z* isomer] δ_H: 1.28 (3H, t, *J* 7.1), 1.72 (3H, dd, *J* 6.9, 1.8), 3.60 (1H, br s), 5.89 (1H, dq, *J* 11.2, 6.9), 6.27 (1H, dd, *J* 11.3, 2.0), 7.05 (1H, dt, *J* 7.4, 1.2).

Note the missing Z isomer signals are hidden underneath E isomer signals

N-allyl-2-(propyl-1-en-1-yl)aniline **S18**

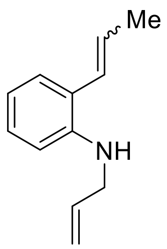

Following general procedure **B**, $Pd(OAc)_2$ (84.2 mg, 0.38 mmol), (±)-BINAP (0.47 g, 0.75 mmol), NaO*t*Bu (2.2 g, 23 mmol), 1-bromo-2-(prop-1-en-1-yl)benzene **S3** (3.0 g, 15 mmol) and benzylamine (1.0 mL, 18 mmol) in PhMe (15 mL) at 60°C for 18 h gave, after purification by Biotage® Selekt™ (Sfär HC 100 g, 180 mL.min^{-1}, Petrol : EtOAc [100:0 to 90:10 10 CV, 90:10 to 85:15 10 CV]), the title compound **S18** (2.6 g, 73%) as a yellow oil in a ~6:4 ratio of (*Z*) : (*E*) isomers. **IR** ν_{max} (film) 3422, 3007, 2913, 1601, 1503, 1312, 918; **^{1}H NMR** (400 MHz, $CDCl_3$) [*Z* isomer] δ_H: 1.74 (3H, dt, *J* 6.9, 1.5), 3.81–3.90 (3H, m), 5.18 (1H, ddq, *J* 10.2, 7.2, 1.4), 5.29 (1H, ddq, *J* 17.2, 8.8, 1.5), 5.87–6.11 (2H, m), 6.30 (1H, d, *J* 9.2), 6.63 (1H, t, *J* 8.4), 6.71 (1H, t, *J* 7.4), 7.06 (1H, dd, *J* 7.4), 7.19 (1H, m); [*E* isomer] δ_H: 1.91 (3H, dt, *J* 6.6, 1.4), 6.42 (1H, dd, *J* 15.5, 2.0), 7.12–7.16 (2H, m); **^{13}C{^{1}H} NMR** (101 MHz, $CDCl_3$) [*Z* isomer] δ_C: 14.7, 46.6, 110.4, 116.2, 116.7, 122.9, 126.1, 128.2, 129.4, 129.7, 135.6, 145.5; [*E* isomer] δ_C: 19.1, 46.7, 110.9, 116.4, 117.6, 124.9, 126.8, 127.6, 128.2, 128.6, 144.9; **HRMS** (ESI)$^+$ $C_{12}H_{16}N$ [M+H]$^+$ found 174.1278, requires 174.1277 (+0.50 ppm).

Note the missing E isomer signals are hidden underneath Z isomer signals

N-(furan-2-ylmethyl)-2-(prop-1-en-1-yl)aniline **S19**

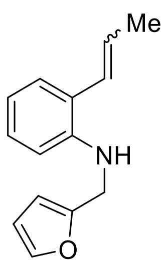

Following general procedure **B**, $Pd(OAc)_2$ (84.2 mg, 0.38 mmol), (±)-BINAP (0.47 g, 0.75 mmol), NaO*t*Bu (2.2 g, 23 mmol), 1-bromo-2-(prop-1-en-1-yl)benzene **S3** (2.9 g, 15 mmol) and furfurylamine (1.7 mL, 18 mmol) in PhMe (15 mL) at 110 °C for 18 h gave, after purification by Biotage® Selekt™ (100 g, 180 mL.min^{-1}, Petrol : EtOAc, [100:0 to 90:10 10 CV, 90:10 to 75:25 10 CV]), the title compound **S19** (2.6 g, 81%) as a yellow oil in a 9:1

mixture of (*Z*) : (*E*) isomers. **IR** ν_{max} (film) 3435, 2913, 1601, 1504, 1310, 1146, 1013, 968; **^{1}H NMR** (400 MHz, $CDCl_3$) [*Z* isomer] δ_H: 1.92 (3H, dq, *J* 6.6, 1.8), 4.14 (1H, br s), 4.36 (2H, d, *J* 5.6), 6.04–6.13 (1H, m), 6.27 (1H, app t, *J* 2.7), 6.35 (1H, td, *J* 3.3, 1.8), 6.43 (1H, dt, *J* 15.5, 2.1), 6.70–6.78 (2H, m), 7.16 (1H, tq, *J* 7.8, 1.0), 7.22 (1H, dt, J 7.4, 2.2), 7.40 (1H, dt, *J* 7.2, 1.0); [*E* isomer] δ_H: 1.74 (3H, ddd, *J* 6.7, 4.3, 1.8), 5.88–5.97 (1H, m), 7.96–7.11 (1H, m), 7.38–7.39 (1H, m); **^{13}C{^{1}H} NMR** (126 MHz, $CDCl_3$) [*Z* isomer] δ_C: 19.0, 41.7, 107.1, 110.5, 111.0, 118.1, 125.2, 126.7, 127.6, 128.2, 128.8, 142.1, 144.5, 152.9; [*E* isomer] δ_C: 14.6, 41.6, 107.0, 110.5, 117.2, 125.9, 129.6, 129.7, 142.0, 145.1; **HRMS** (ESI)$^+$ $C_{14}H_{16}NO$ [M+H]$^+$ found 214.1226, requires 214.1226 (–0.40 ppm).

Note the missing E isomer signals are hidden underneath Z isomer signals

(*E*)-isopropyl-2-(prop-1-en-1-yl)aniline **S20**

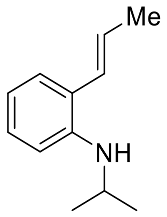

Following general procedure **B**, $Pd(OAc)_2$ (84.2 mg, 0.38 mmol), (±)-BINAP (0.47 g, 0.75 mmol), NaO*t*Bu (2.2 g, 23 mmol), 1-bromo-2-(prop-1-en-1-yl)benzene **S3** (3.0 mL, 15 mmol) and isopropylamine (1.5 mL, 18 mmol) in PhMe (15 mL) at 60 °C for 24 h gave, after purification by Biotage® Selekt™ (Sfär HC 100 g, 250 mL.min^{-1}, Petrol : EtOAc [100:0 5 CV, 100:0 to 90:10 5 CV, 90:10 to 75:25 10 CV]), the title compound **S20** (1.2 g, 40 %) as a yellow oil. **IR** ν_{max} (film) 3420, 2963, 1601, 1501, 1175; **^{1}H NMR** (400 MHz, $CDCl_3$) δ_H: 1.24 (6H, d, *J* 6.2), 1.91 (3H, dd, *J* 6.6, 1.7), 3.57 (1H, br s), 3.66 (1H, app dq, *J* 12.5, 6.3), 6.04 (1H, dq, *J* 15.4, 6.6), 6.36 (1H, app dd, *J* 15.5, 1.5), 6.62–6.68 (2H, m), 7.13 (1H, td, *J* 8.2, 1.6), 7.18 (1H, dd, *J* 7.5, 1.6); **^{13}C{^{1}H} NMR** (101 MHz, $CDCl_3$) δ_C: 19.1, 23.3, 44.3, 111.2, 116.9, 124.6, 126.9, 127.8, 128.2, 128.3, 144.4; **HRMS** (ESI)$^+$ $C_{12}H_{18}N$ [M+H]$^+$ found 176.1433, requires 176.1434 (–0.29 ppm).

N-cyclopropyl-2-(prop-1-en-1-yl)aniline **S21**

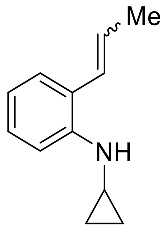

Following general procedure **B**, $Pd(OAc)_2$ (84.2 mg, 0.38 mmol), (±)-BINAP (0.47 g, 0.75 mmol), NaO*t*Bu (2.2 g, 23 mmol), 1-bromo-2-(prop-1-en-1-yl)benzene **S2** (3.0 g, 15 mmol) and cyclopropanamine (2.1 mL, 30 mmol, 2.0 equiv.) in PhMe (15 mL) at 65 °C for 24 h gave, after purification by Biotage® Selekt™ (Sfär HC 100 g, 150 mL.min^{-1}, Cyclohexane : EtOAc [100:0 to 75:25 10 CV, 75:25 to 50:50 10 CV]), the title compound **S21** (1.2 g, 45 %) as a pale orange oil in ~10:1 mixture of (*E*) : (*Z*) isomers. **IR** ν_{max} (film): 3404, 3009, 2969, 2959, 1601, 1497, 1364, 1301, 966; **^{1}H NMR** (400 MHz, $CDCl_3$) [*E* isomer] δ_H: 0.55 (2H, app dq, *J* 5.5, 4.8, 2.9), 0.76 (2H, tdd, *J* 6.3, 4.5, 1.5), 1.90 (3H, dt, *J* 6.6, 1.9), 2.43 (1H, ttd, *J* 6.9, 3,5, 1.7), 4.28 (1H, br s), 6.04 (1H, dqd, *J* 15.1, 6.5, 1.8), 6.33 (1H, dt, *J* 15.5, 1.9), 6.74 (1H, ddd, *J* 7.8, 6.7, 1.7), 7.05–7.08 (1H, m), 7.18 (2H, td, *J* 7.2, 3.5); [*Z* isomer] δ_H: 1.45 (2H, d, *J* 2.0), 1.72 (3H, dt, *J* 7.0, 2.0), 4.19 (1H, br s), 5.87 (1H, dddd, *J* 13.7, 11.3, 7.0, 1.7), 6.22 (1H, d, *J* 11.4);**^{13}C{^{1}H} NMR** (126 MHz, $CDCl_3$) [*E* isomer] δ_C: 7.6, 19.0, 25.4, 111.8, 117.2, 124.3, 126.7, 127.4, 128.1,128.4, 145.6; [*Z* isomer] δ_C: 14.6, 25.3, 27.1, 111.4, 116.9, 126.0, 129.1, 129.5; **HRMS** (ESI)$^+$ $C_{12}H_{16}N$ [M+H]$^+$ found 174.1276, requires 174.1277 (–0.78 ppm).

Note the missing Z isomer signals are hidden underneath E isomer signals

N-cyclobutyl-2-(prop-1-en-1-yl)aniline **S22**

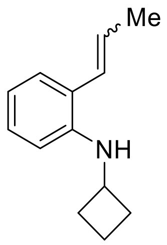

Following general procedure **B**, $Pd(OAc)_2$ (84.2 mg, 0.38 mmol), (±)-BINAP (0.47 g, 0.75 mmol), NaO*t*Bu (2.2 g, 23 mmol), 1-bromo-2-(prop-1-en-1-yl)benzene **S3** (3.0 g, 15 mmol) and cyclobutylamine (1.9 mL, 23 mmol, 1.5 equiv.) in PhMe (15 mL) at 75 °C for 18 h gave, after purification by Biotage® Selekt™ (Sfär HC 100 g, 200 mL.min^{-1}, Petrol : EtOAc [100:0 to 90:10 10 CV]), the title compound **S22** (1.6 g, 56%) as a yellow oil in a 3:1 mixture of (*Z*) : (*E*) isomers. **IR** ν_{max} (film) 3412, 2934, 1601, 1501, 1312, 1169; **^{1}H NMR** (400 MHz, $CDCl_3$) [*Z* isomer] δ_H: 1.74 (3H, dd, *J* 6.9, 1.9), 1.78–1.89 (4H, m), 2.40–2.50

(2H, m), 3.86–3.97 (2H, m), 5.89 (1H, dq, *J* 11.1, 6.9), 6.27 (1H, dt, *J* 11.2, 2.1), 6.55 (1H, d, *J* 8.1), 6.69 (1H, tt, *J* 7.5, 1.6), 7.05 (1H, d, *J* 7.5), 7.09–7.20 (1H, m); [*E* isomer] δ_H: 1.74 (3H, app dt, *J* 11.2, 2.1), 2.40–2.56 (2H, m), 6.06 (1H, dq, *J* 15.4, 6.5), 6.39 (1H, dd, *J* 15.5, 2.2), 6.52 (1H, d, *J* 7.6); **^{13}C{^{1}H} NMR** (101 MHz, $CDCl_3$) [*Z* isomer] δ_C: 14.7, 15.4, 31.5, 49.0, 110.7, 116.5, 122.6, 126.1, 128.2, 129.1, 129.7, 144.7; [*E* isomer] δ_C: 15.5, 19.1, 49.2, 111.2, 117.4, 126.8, 127.5, 128.2, 128.3, 144.1; **HRMS** (ESI)$^+$ $C_{13}H_{18}N$ [M+H]$^+$ found 188.1433, requires 188.1434 (–0.28 ppm).

Note the missing E isomer signals are hidden underneath Z isomer signals

N-(cyclohexylmethyl)-2-(prop-1-en-1-yl)aniline **S23**

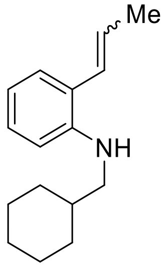

Following general procedure **B**, $Pd(OAc)_2$ (66.2 mg, 0.30 mmol), (±)-BINAP (0.40 g, 0.60 mmol), NaO*t*Bu (1.7 g, 18 mmol), 1-bromo-2-(prop-1-en-1-yl)benzene **S3** (2.3 g, 12 mmol) and cyclohexylmethanamine (1.8 mL, 14 mmol) in PhMe (12 mL) at 110 °C for 18 h gave, after purification by Biotage® Selekt™ (Sfär HC 100 g, 150 mL.min^{-1}, Petrol : EtOAc [100:0 to 90:10 10 CV, 90:10 to 75:25 10 CV]), the title compound **S23** (1.7 g, 62%) as a colourless oil in a 1:1 mixture of (*Z*) : (*E*) isomers. **IR** ν_{max} (film) 3428, 3013, 2920, 2851, 1601, 1506, 1319, 968; **^{1}H NMR** (400 MHz, $CDCl_3$) [*Z* isomer] δ_H: 0.94–1.05 (2H, m), 1.13–1.33 (3H, m), 1.55–1.65 (1H, m), 1.65–1.86 (8H, m), 2.97 (2H, t, *J* 5.9), 3.82 (1H s), 5.89 (1H, dq, *J* 10.9, 6.9), 6.26 (1H, d, *J* 10.6), 6.66 (2H, td, *J* 7.4, 1.5), 7.03 (1H, d, *J* 7.3), 7.11–7.17 (1H, m); [*E* isomer] δ_H: 0.94–1.05 (2H, m), 1.13–1.33 (3H, m), 1.55–1.65 (1H, m), 1.65–1.86 (5H, m), 1.90–1.91 (3H, m), 2.97 (2H, t, *J* 5.9), 3.77 (1H, s), 6.05 (1H, dq, *J* 14.7, 6.6), 6.38 (1H, d, *J* 15.4), 6.61 (2H, t, *J* 8.7), 7.11–7.17 (2H, m); **^{13}C{^{1}H} NMR** (101 MHz, $CDCl_3$) [*Z* isomer] δ_C: 14.7, 26.1, 31.5, 37.6, 50.7, 116.0, 116.9, 122.6, 126.1, 128.2, 129.3, 129.6, 145.4; [*E* isomer] δ_C: 19.1, 26.8, 31.6, 37.7, 50.8, 110.0, 110.4, 124.4, 126.9, 127.6, 128.2, 128.4, 146.0; **HRMS** (ESI)$^+$ $C_{16}H_{24}N$ [M+H]$^+$ found 230.1901, requires 230.1093 (–0.93 ppm).

N-2-(but-2-en-2-yl)-N-(cyclohexylmethyl)aniline **S24**

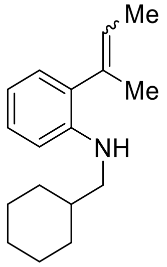

Following general procedure **B**, $Pd(OAc)_2$ (84.2 mg, 0.38 mmol), (±)-BINAP (0.47 g, 0.75 mmol), NaO*t*Bu (2.2 g, 23 mmol), 1-bromo-2-(but-2-en-2-yl)benzene **S6** (3.2 g, 15 mmol), cyclohexylmethanamine (2.3 mL, 18 mmol) in PhMe (15 mL) at 110 °C for 16 h gave, after purification by Biotage® Selekt™ (Sfär HC 100 g, 180 mL.min^{-1}, Petrol : EtOAc, [100:0 5 CV, 100:0 to 90:10 10 CV]), the title compound **S24** (2.6 g, 71%) as a colourless oil in ~8.5 : 1.5 mixture of (*Z*) : (*E*) isomers. **IR** ν_{max} (film) 3420, 2920, 2851, 1601, 1506, 1319; **^{1}H NMR** (500 MHz, $CDCl_3$) [*Z* isomer] δ_H: 1.00 (2H, dq, *J* 12.1, 3.1), 1.17–1.33 (3H, m), 1.46 (3H, dd, *J* 6.7), 1.57–1.65 (1H, m), 1.69–1.73 (1H, m), 1.75–1.82 (4H, m), 1.97 (3H, t, *J* 1.6), 3.00 (2H, br s), 3.94 (1H, br s), 5.70 (1H, dddd, *J* 8.2, 6.7, 5.2, 1.5), 6.61–6.67 (1H, app m), 6.70 (1H, td, *J* 7.4, 1.1), 6.93 (1H, dd, *J* 7.4, 1.7), 7.15–7.19 (1H, m); [*E* isomer] δ_H: 1.95–1.96 (3H, m), 4.00 (1H, br s), 5.56 (1H, dddd, *J* 8.3, 6.7, 5.3, 1.6), 6.97 (1H, dd, *J* 7.4, 1.7), 7.13–7.16 (1H, m); **^{13}C{^{1}H} NMR** (126 MHz, $CDCl_3$) [*Z* isomer] δ_C: 14.8, 24.8, 26.2, 26.7, 31.5, 37.7, 50.7, 109.8, 116.2, 124.0, 127.3, 127.9, 128.3, 134.6, 144.7; [*E* isomer] δ_C: 14.1, 17.3, 37.5, 50.7, 110.1, 116.2, 124.8, 127.8, 128.5, 131.6, 134.6, 145.1; **HRMS** (ESI)$^+$ $C_{17}H_{26}H$ $[M+H]^+$ found 244.2055, requires 244. 2060 (–1.79 ppm).

Note the missing E isomer signals are hidden underneath Z isomer signals

N-(cyclohexylmethyl)-2-vinylaniline **S25**

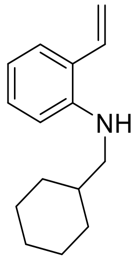

Following general procedure **B**, $Pd(OAC)_2$ (84.2 mg, 0.38 mmol), (±)-BINAP (0.47 g, 0.75 mmol), NaO*t*Bu (2.2 g, 23 mmol), 1-bromo-2-vinylbenzene **S2** (1.9 mL, 15 mmol) and cyclohexylmethylamine (2.3 mL, 18 mmol) in PhMe (15 mL) at 110 °C for 7 h gave, after purification by Biotage® Selekt™ (Sfär HC 100 g, 180 mL.min^{-1},Petrol : EtOAc [100:0 to 90:10 10 CV, 90:10 to 72:25 10 CV]), the title compound **S25** (2.0 g, 61%) as a colourless oil. **IR** ν_{max} (film) 3435, 3080, 2920, 2849, 1603, 1506, 1447, 1310, 1258, 907; **^{1}H NMR**

(500 MHz, $CDCl_3$) δ_H: 1.03 (2H, dq, *J* 12.1, 3.4), 1.18–1.34 (3H, m), 1.60–1.68 (1H, m), 1.68–1.71 (1H, m), 1.78 (2H, dt, *J* 11.9, 3.0), 1.85–1.88 (2H, m), 3.01 (2H, d, *J* 6.7), 3.89 (1H, br s), 5.34 (1H, dd, *J* 11.0, 1.6), 5.63 (1H, dd, *J* 17.3, 1.6), 6.65 (1H, dd, *J* 8.2, 1.1), 6.72 (1H, td, *J* 7.4, 1.1), 6.78 (1H, dd, *J* 17.3, 11.0), 7.20 (1H, td, *J* 7.7, 1.6), 7.26–7.28 (1H, m); **$^{13}C\{^1H\}$ NMR** (126 MHz, $CDCl_3$) δ_C: 26.1, 26.7, 31.5, 37.6, 50.8, 110.7, 116.2, 116.9, 124.1, 127.6, 129.1, 133.2, 145.7; **HRMS** $(ESI)^+$ $C_{15}H_{22}N$ $[M+H]^+$ found 216.1744, requires 216.1747 (–1.23 ppm).

N-2-(but-2-en-2-yl)-N-cyclopentylaniline **S26**

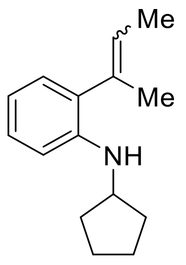

Following general procedure **B**, $Pd(OAc)_2$ (56.1 mg, 0.25 mmol), (±)-BINAP (0.30 g, 0.50 mmol), NaO*t*Bu (1.4 g, 15 mmol), 1-bromo-2-(but-2-en-2-yl)benzene **S6** (2.1 g, 10 mmol), cyclopentylamine (1.0 g, 12 mmol), in PhMe (10 mL) for 18 h gave, after purification by Biotage® Selekt™ (Sfär HC 100 g, 150 mL.min^{-1}, Petrol : EtOAc [100:0 to 90:10 10 CV, 90:10 to 75:25 10 CV]), the title compound **S26** (1.7 g, 79%) as a pale yellow oil in ~8:2 mixture of (*Z*) : (*E*) isomers. **IR** ν_{max} (film) 3408, 2957, 1601, 1504, 1307, 1028; **^1H NMR** (400 MHz, $CDCl_3$) [*Z* isomer] δ_H: 1.44 (3H, dq, *J* 6.7, 1.5), 1.44–1.52 (2H, m), 1.60–1.70 (4H, m), 1.99–2.05 (5H, m), 3.78–3.87 (2H, m), 5.66 (1H, dq, *J* 6.7, 1.5), 6.66–6.70 (2H, m), 6.91 (1H, dd, *J* 7.6, 1.7), 7.13–7.17 (1H, m); [*E* isomer] δ_H: 1.79 (1H, dq, *J* 6.8, 1.1), 1.93 (3H, app d, *J* 1.3), 5.53 (1H, dddd, *J* 8.3, 6.8, 5.3, 1.5), 6.94–6.97 (1H, m); **$^{13}C\{^1H\}$ NMR** (126 MHz, $CDCl_3$) [*Z* isomer] δ_C: 14.7, 24.1, 24.8, 33.6, 33.7, 54.5, 110.8, 116.3, 123.7, 127.4, 127.8, 128.3, 134.6, 144.0; [*E* isomer] δ_C: 14.1, 17.2, 24.1, 54.8, 111.3, 116.4, 124.9, 127.7, 128.5; **HRMS** $(ESI)^+$ $C_{15}H_{22}N$ $[M+H]^+$ found 216.1745, requires 216.1747 (–0.81 ppm).

Note the missing E isomer signals are hidden underneath Z isomer signals

N-(4-methoxybenzyl)-2-(prop-1-en-1-yl)aniline **S27**

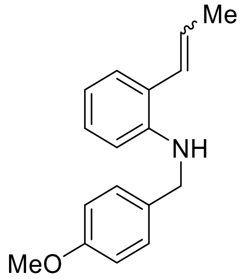

Following general procedure **B**, Pd(OAc)$_2$ (112.3 mg, 0.50 mmol), (±)-BINAP (0.62 g, 1.0 mmol), NaO*t*Bu (1.5 g, 30 mmol), *N*-1-bromo-2-(prop-1-en-1-yl)benzene **S3** (3.9 g, 20 mmol) and 4-(methoxyphenyl)methanamine (3.3 g, 24 mmol) in PhMe (20 mL) at 110 °C for 4 h gave, after purification by Biotage® Selekt™ (Sfär HC 200 g, 180 mL.min^{-1}, Petrol : EtOAc [100:0 to 90:10 10 CV, 90:10 to 75:25 10 CV]), the title compound **S27** (4.3 g, 84%) as a pale yellow oil in ~ 1.1:1 mixture of (*Z*) : (*E*) isomers. **IR** ν_{max} (film) 3418, 1508, 1244, 744; **^{1}H NMR** (400 MHz, CDCl$_3$) [*Z* isomer] δ_H: 1.75 (3H, ddt, *J* 6.8, 3.3, 1.5), 3.81–3.82 (3H, m), 4.04 (1H, br s), 4.29 (2H, s), 5.89–5.92 (1H, m), 6.29 (1H, d, *J* 11.1), 6.65 (2H, tt, *J* 8.3, 2.5), 6.90 (4H, ddt, *J* 8.5, 5.6, 2.4), 7.28–7.34 (2H, m); [*E* isomer] δ_H: 1.87–1.90 (3H, m), 3.81–3.82 (3H, m), 4.04 (1H, br s), 4.29 (2H, s), 6.02–6.12 (1H, m), 6.41 (1H, d, *J* 15.7), 6.69–6.74 (2H, m), 7.07–7.28 (4H, m), 7.28–7.34 (2H, m); **^{13}C{^{1}H} NMR** (126 MHz, CDCl$_3$) [*Z* isomer] δ_C: 14.7, 47.9, 55.4, 110.4, 114.2, 116.7, 122.8, 126.1, 127.5, 128.3, 128.9, 129.4, 131.6, 145.0, 159.0; [*E* isomer] δ_C: 19.1, 48.0, 110.8, 117.6, 124.7, 126.8, 128.2, 128.6, 129.0, 129.6, 131.5, 145.6; **HRMS** (ESI)$^+$ $C_{17}H_{19}NONa$ [M+Na]$^+$ found 276.1358, requires 276.1359 (–0.38 ppm).

Note the missing E isomer signals are hidden underneath Z isomer signals

N-phenyl-2-(prop-1-en-1-yl)aniline **S28**

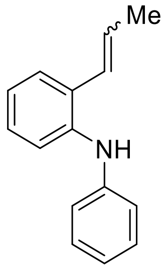

Following general procedure **B**, Pd(OAc)$_2$ (84.2 mg, 0.38 mmol), (±)-BINAP (0.47 g, 0.75 mmol), NaO*t*Bu (2.2 g, 23 mmol), 1-bromo-2-(prop-1-en-1-yl)benzene **S3** (3.0 g, 15 mmol) and aniline (1.7 mL, 1.7 mmol) in PhMe (15 mL) at 110 °C for 23 h gave, after purification by Biotage® Selekt™ (Sfär HC 100 g, 200 mL.min^{-1}, Petrol : EtOAc [100:0 to 75:25 10 CV, 75:25 to 50:50 10 CV]), the title compound **S28** (1.8, 57%) as a pale yellow

oil in a 9:1 mixture of (*E*) : (*Z*) isomers with all spectroscopic data in accordance with the literature.[96] **IR** ν_{max} (film) 3999, 3019, 2911, 1593, 1497, 1300, 968; **^{1}H NMR** (400 MHz, $CDCl_3$) [*E* isomer] δ_H: 1.91 (3H, dd, *J* 6.6, 1.8), 5.52 (1H, br s), 6.17 (1H, dq, *J* 15.6, 6.6), 6.55 (1H, dq, *J* 15.6, 1.8), 6.90 (1H, app t, *J* 7.3), 6.98 (3H, app d, *J* 8.6), 7.16 (1H, td, *J* 8.2, 1.4), 7.26 (3H, app t, *J* 7.9), 7.41 (1H, dd, *J* 7.7, 1.6); [*Z* isomer] δ_H: 1.78 (3H, dd, *J* 7.0, 1.8), 5.60 (1H, br s), 5.95 (1H, dq, *J* 11.3, 7.0), 6.39 (1H, dd, *J* 11.4, 1.9), 7.07 (2H, app d, *J* 7.6), 7.33 (1H, dd, *J* 8.2, 1.2).

Note the missing Z isomer signals are hidden underneath E isomer signals

6.1.4. Naphthol synthesis

1-bromo-7-methoxynaphthalen-2-ol **S29**

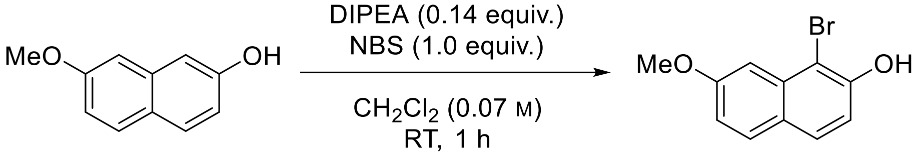

Following literature[97] and under nitrogen, 7-methoxy-2-naphthol (1.0 g, 5.8 mmol) and diisopropylethylamine (81 µL, 0.80 mmol) were stirred in CH_2Cl_2 (20 mL). NBS (1.0 g, 5.8 mmol) in CH_2Cl_2 (60 mL) was added dropwise over 30 mins, and the reaction was stirred at RT for 1 h, then poured into water (100 mL) and quenched with 12 M aq. HCl to pH 1. The mixture was diluted with CH_2Cl_2, then the organic layer was extracted, dried (Na_2SO_4) and concentrated under reduced pressure to afford the crude which was purified by Biotage® Selekt™ (Sfär HC 100 g, 250 mL.min^{-1}, Petrol : EtOAc [95:5 to 90:10 5 CV, 90:10 to 75:25 10 CV]), to give the title compound **S29** (1.3 g, 87%) as a pale pink solid with all spectroscopic data in accordance with the literature.[97] **mp** 100–102 °C {Lit.[98] 92–94 °C}; **IR** ν_{max} (film) 3414, 2970, 1618, 1377, 1159, 1140, 835; **^{1}H NMR** (400 MHz, $CDCl_3$) δ_H: 3.97 (3H, s), 5.89 (1H, s), 7.04 (1H, dd, *J* 8.9, 2.5), 7.12 (1H, d, *J* 8.8), 7.32 (1H, d, *J* 2.5), 7.66 (2H, app dd, *J* 8.8, 7.4).

(1-bromonaphthalen-2-yl)methanol **S30**

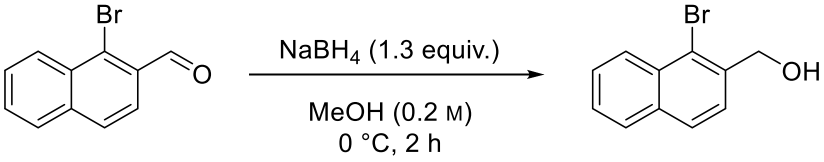

Following literature[99] and under nitrogen, 1-bromo-2-naphthaldehyde (1.2 g, 5.0 mmol, 1.0 equiv.) and MeOH (25 mL) were set to 0 °C. $NaBH_4$ (0.24 g, 6.3 mmol, 1.3 equiv.) was added portionwise and the mixture was stirred at 0 °C for 2 h, then quenched with aq. NH_4Cl and concentrated under reduced pressure to remove MeOH. The crude solution was diluted with EtOAc, the organic phases extracted, dried (Na_2SO_4) and concentrated under reduced pressure to afford the title compound **S30** (1.1 g, 94%) as a pale orange solid which was used without further purification and with all spectroscopic data in accordance with the literature.[100] **mp** 103–105 °C {Lit.[100] 101–102 °C}; **IR** ν_{max} (film) 3246, 3067, 2853, 1503, 1321, 1063; **^{1}H NMR** (500 MHz, $CDCl_3$) δ_H: 2.12 (1H, br s), 5.00 (2H, s), 7.53 (1H, ddd, *J* 8.1, 6.8, 1.2), 7.59–7.64 (2H, m), 7.83–7.85 (2H, m), 8.31 (1H, dd, *J* 8.5, 1.2).

6.1.5. Alcohol synthesis

1-(1-benzylbenzo[e][1,2]azaborinin-2(1H)-yl)naphthalen-2-ol **24**

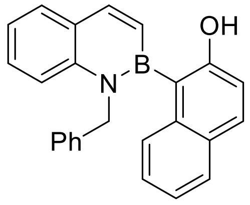

Following general procedure **C**, *N*-benzyl-2-vinylaniline **S9** (3.3 g, 9.0 mmol) and BCl_3 (1.0 M in hexane, 18 mL, 18 mmol), in PhMe (18 mL) for 18 h gave the chlorinated intermediate. 1-bromonaphthalen-2-ol (2.2 g, 9.9 mmol) with nBuLi (2.1 M in hexane, 10 mL, 24 mmol) in THF (50 mL) for 2 h, then the chlorinated intermediate in THF (18 mL) for 2 h gave, after purification by Biotage® Selekt™ (Sfär HC 100 g, 180 mL.min^{-1}, Petrol : CH_2Cl_2 [90:10 to 70:30 10 CV, 70:30 to 50:50 10 CV]), the title compound **24** (1.4 g, 42%) as an off-white solid. **mp** 165–167 °C; **IR** ν_{max} (solid) 3536, 3028, 1591, 1227, 733; **^{1}H NMR** (700 MHz, $CDCl_3$) δ_H: 4.82 (1H, s), 5.34 (1H, d, *J* 16.4), 5.37 (1H, d, *J* 16.4), 7.07 (3H, app d, *J* 8.7), 7.11–7.14 (2H, m) 7.18 (2H, t, *J* 7.5), 7.27 (1H, t, *J* 7.4), 7.30–7.34 (2H, m), 7.41 (1H, dt, *J* 5.8, 1.4), 7.50 (1H, d, *J* 8.6), 7.56 (1H, app d, *J* 7.4), 7.76 (1H, d, *J* 8.8), 7.80 (2H, app t, *J* 8.8) 8.23 (1H, d, *J* 11.2); **^{13}C{^{1}H} NMR** (176 MHz, $CDCl_3$) δ_C: 53.0, 117.4,117.8, 121.5, 123.3, 126.2, 126.3, 126.9, 127.5, 127.9, 128.5, 128.7, 129.2, 130.2, 130.7, 131.5, 136.5, 138.8, 141.7, 146.2, 154.7; **^{11}B{^{1}H} NMR** (128 MHz, $CDCl_3$) δ_B: 38.95 (br s); **HRMS** (ESI)$^+$ $C_{25}H_{20}BNONa$ $[M+Na]^+$ found 384.1529, requires 384.1530 (–0.38 ppm).

1-(1-benzyl-3-methylbenzo[*e*][1,2]azaborinin-2(1*H*)-yl)naphthalen-2-ol **15**

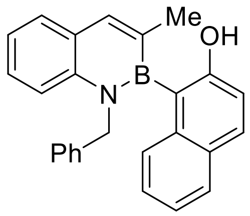

Following general procedure **C**, *N*-benzyl-2-(prop-1-en-1-yl)aniline **S10** (1.2 g, 5.3 mmol) and BCl_3 (1.0 M in hexane, 10.6 mL, 11 mmol) in PhMe (11 mL) for 18 h gave the chlorinated intermediate. 1-bromonaphthalen-2-ol (1.3 g, 5.8 mmol) and nBuLi (1.7 M in hexane, 8.4 mL, 14 mmol) in THF (30 mL) for 2 h, then the chlorinated intermediate in THF (11 mL) for 2 h gave, after purification by Biotage® Selekt™ (Sfär HC 100g, 150mL.min^{-1}, Hexane : CH_2Cl_2 [100:0 to 50:50 20 CV, 50:50 to 0:100 20 CV]), the title compound **15** (1.1 g, 53%) as a pale yellow amorphous solid. **IR** ν_{max} (film) 3543, 2953, 1611, 1227, 907, 729; **^{1}H NMR** (400 MHz, $CDCl_3$) δ_H: 2.08 (3H, d, *J* 1.3), 4.65 (1H, s), 5.29 (2H, d, *J* 2.2), 7.04 (1H, d, *J* 8.8), 7.07 (2H, d, *J* 6.7), 7.10–7.20 (3H, m), 7.22–7.26 (1H, m), 7.29–7.36 (3H, m), 7.41–7.43 (2H, m), 7.73 (1H, dd, *J* 7.8, 1.7), 7.75 (1H, d, *J* 8.7), 7.79–7.82 (1H, m), 7.97 (1H, s); **^{13}C{^{1}H}**

NMR (126 MHz, $CDCl_3$) δ_C: 21.8, 53.2, 117.1, 117.7, 121.5, 123.4, 126.2, 126.3, 126.8, 127.1, 127.5, 127.7, 128.5, 128.6, 129.2, 129.7, 130.0, 136.3, 139.0, 140.4, 142.6, 154.3; **^{11}B{^{1}H}** (128 MHz, $CDCl_3$) δ_B: 40.33 (br s); **HRMS** (ESI$^+$) $C_{26}H_{22}BNONa$ [M+Na]$^+$ found 398.1688, requires 398.1687 (+0.2 ppm).

1-(1-benzyl-3-ethylbenzo[*e*][1,2]azaborinin-2(1*H*)-yl)naphthalen-2-ol **26**

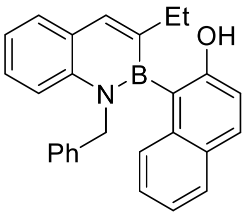

Following general procedure **C**, *N*-benzyl-2-(but-1-en-1-yl)aniline **S11** (3.2 g, 14 mmol) and BCl_3 (1.0 M in hexane, 27 mL, 27 mmol) in THF (27 mL) for 18 h gave the chlorinated intermediate. 1-bromonaphthalen-2-ol (3.3 g, 15 mmol) and nBuLi (2.3 M in hexane, 16 mL, 36 mmol) in THF (75 mL) for 2 h, then the chlorinated intermediate in THF (27 mL) for 16 h gave, after purification by Biotage® Selekt™ (Sfär HC 100 g, 267 mL.min^{-1}, Petrol : CH_2Cl_2 [90:10 to 70:30 10 CV, 70:30 to 50:50 10 CV]), the title compound **26** (0.99 g, 17%) as a pale yellow amorphous solid. **IR** ν_{max} (film) 3534, 2959, 1609, 1452, 1331, 1227, 906; **^{1}H NMR** (400 MHz, $CDCl_3$) δ_H: 1.01 (3H, t, *J* 7.5), 2.42 (2H, q, *J* 7.5), 4.61 (1H, s), 5.24–5.33 (2H, m), 7.04 (1H, d, *J* 8.8), 7.07 (2H, app dd, *J* 6.7, 1.7), 7.12–7.20 (3H, m), 7.24–7.36 (4H, m), 7.42–7.44 (2H, m), 7.75–7.82 (3H, m), 7.98 (1H, s); **^{13}C{^{1}H} NMR** (126 MHz, $CDCl_3$) δ_C: 14.9, 28.3, 53.1, 117.1, 117.7, 121.5, 123.4, 126.2, 126.2, 126.8, 127.3, 127.6, 127.8, 128.5, 128.6, 129.2, 129.9, 130.0, 136.5, 139.0, 140.5, 141.0, 154.4; **^{11}B{^{1}H} NMR** (128 MHz, $CDCl_3$) δ_B: 38.93 (br s); **HRMS** (ESI$^+$) $C_{27}H_{24}BNONa$ [M+Na]$^+$ found 412.1841, requires 412.1843 (–0.59 ppm).

1-(1-benzyl-4-methylbenzo[e][1,2]azaborinin-2(1H)-yl)naphthalen-2-ol **28**

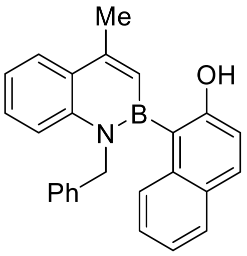

Following general procedure **C**, *N*-benzyl-2-(prop-1-en-2-yl)aniline **S12** (0.80 g, 3.5 mmol) and BCl_3 (1.0 M in hexane, 7.1 mL, 7.1 mmol) in PhMe (7 mL) for 18 h gave the chlorinated intermediate. 1-bromonaphthalen-2-ol (0.9 g, 3.9 mmol), nBuLi (2.1 M in hexane, 4.3 mL, 9.2 mmol) in THF (19 mL) for 2 h, then the chlorinated intermediate in THF (7 mL) for 2 h

gave, after purification by Biotage® Selekt™ (Sfär HC 100 g, 150 mL.min^{-1}, Petrol : EtOAc, [100:0 to 90:10 7 CV, 90:10 to 75:25 8 CV]), the title compound **28** (0.51 g, 39%) as an off-white amorphous solid. **IR** ν_{max} (film) 3537, 3055, 2978, 1593, 1231, 1169, 908; **^{1}H NMR** (400 MHz, $CDCl_3$) δ_H: 2.72 (3H, d, *J* 1.1), 4.87 (1H, s), 5.33 (2H, dd, *J* 15.8, 2.6), 6.97 (1H, s), 7.06–7.09 (3H, m), 7.11–7.14 (1H, m), 7.16–7.20 (2H, m), 7.27–7.35 (3H, m), 7.41 (1H, ddd, *J* 8.6, 7.0, 1.6), 7.52 (1H, dd, *J* 8.6, 1.2), 7.56–7.60 (1H, m), 7.76 (1H, d, *J* 8.5), 7.78–7.81 (1H, m), 8.00 (1H, dd, *J* 8.1, 1,6); **^{13}C{^{1}H} NMR** (126 MHz, $CDCl_3$) δ_C: 23.3, 53.0, 117.8, 121.3, 123.3, 126.2, 126.2, 126.6, 126.8, 127.6, 127.9, 128.4, 128.5, 128.6, 129.2, 130.1, 131.8 (br s), 136.6, 139.0, 141.8, 152.5, 154.7; **^{11}B{^{1}H} NMR** (128 MHz, $CDCl_3$) δ_B: 36.97 (br s); **HMRS** (ESI)$^+$ $C_{26}H_{22}BNONa$ [M+Na]$^+$ found 398.1686, requires 398.1687 (–0.10 ppm).

1-(1-benzyl-3,4-dimethylbenzo[*e*][1,2]azaborinin-2(1*H*)-yl)naphthalen-2-ol **30**

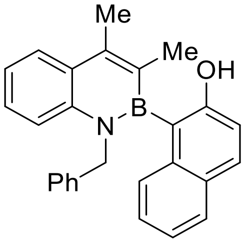

Following general procedure **C**, *N*-benzyl-2-(but-2-en-2-yl)aniline **S13** (2.3 g, 9.6 mmol) and BCl_3 (1.0 M in hexane, 19 mL, 19 mmol) in PhMe (19 mL) for 18 h gave the chlorinated intermediate. 1-bromonaphthalen-2-ol (2.4 g, 11 mmol), nBuLi (2.3 M in hexane, 11 mL, 25 mmol) in THF (53 mL) for 2 h, then the chlorinated intermediate in THF (19 mL) for 3 h gave, after purification by Biotage® Selekt™ (Sfär HC 100 g, 200 mL.min^{-1}, Petrol : EtOAc [90:10 to 70:30 10 CV, 70:30 to 50:50 10 CV, 50:50 to 0:100 10 CV]), the title compound **30** (0.62 g, 17%) as a white amorphous solid. **IR** ν_{max} (film) 3526, 2918, 1585, 1225, 810; **^{1}H NMR** (400 MHz, $CDCl_3$) δ_H: 2.03 (3H, s), 2.63 (3H, s), 4.62 (1H, s), 5.26 (2H, s), 7.05 (3H, d, *J* 8.7), 7.09–7.19 (3H, m), 7.26–7.36 (4H, m), 7.41–7.43 (1H, m), 7.45 (1H, dd, *J* 8.4, 1.4), 7.75 (1H, d, *J* 8.8), 7.78–7.82 (1H, m), 8.07 (1H, dd, *J* 8.1, 1.7); **^{13}C{^{1}H} NMR** (126 MHz, $CDCl_3$) δ_C: 16.0, 18.4, 53.4, 117.5, 117.7, 121.3, 123.3, 125.9, 126.1, 126.2, 126.7, 127.1, 127.3, 128.2, 128.4, 128.6, 129.2, 129.8, 136.5, 139.1, 140.4, 146.9, 154.3; **^{11}B{^{1}H} NMR** (128 MHz, $CDCl_3$) δ_B: 39.92 (br s); **HRMS** (ESI$^+$) $C_{27}H_{24}BNONa$ [M+Na]$^+$ found 412.1842, requires 412.1843 (–0.25 ppm).

1-(1-benzyl-3,6-dimethylbenzo[e][1,2]azaborinin-2(1H)-yl)naphthalen-2-ol **32**

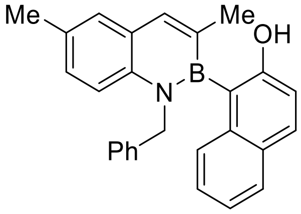

Following general procedure **C**, *N*-benzyl-4-methyl-2-(prop-1-en-1-yl)aniline **S14** (2.4 g, 10 mmol) and BCl_3 (1.0 M in hexane, 20 mL, 20 mmol), in PhMe (20 mL) for 18 h gave the chlorinated intermediate. 1-bromonaphthalen-2-ol (2.5 g, 11 mmol), nBuLi (2.1 M in hexane, 12 mL, 26 mmol) in THF (55 mL) for 2 h, then the chlorinated intermediate in THF (20 mL), for 1.5 h gave, after purification by Biotage® Selekt™ (Sfär HC 100 g, 200 mL.min^{-1}, Petrol : CH_2Cl_2, [90:10 to 75:25 10 CV, 75:25 to 50:50 10 CV]), the title compound **32** (2.7 g, 70%) as an off-white amorphous solid. **IR** ν_{max} (film) 3536, 2918, 1595, 1423, 1233, 905; **^{1}H NMR** (400 MHz, $CDCl_3$) δ_H: 2.06 (3H, s), 2.44 (3H, s), 4.61 (1H, app d, *J* 6.9), 5.22–5.30 (2H, m), 7.04–7.07 (3H, m), 7.10–7.19 (4H, m), 7.29–7.31 (3H, m), 7.42 (1H, t, *J* 4.7), 7.51 (1H, s), 7.75 (1H, d, *J* 8.7), 7.80 (1H, dd, *J* 7.3, 2.3), 7.90 (1H, s); **^{13}C{^{1}H} NMR** (126 MHz, $CDCl_3$) δ_C: 20.8, 21.8, 53.2, 116.9, 117.7, 123.3, 126.2, 126.3, 126.8, 127.1, 127.7, 128.5, 128.6, 128.8, 129.2, 129.5, 129.9, 130.8, 136.4, 138.5, 139.0, 142.4, 154.3; **^{11}B{^{1}H} NMR** (128 MHz, $CDCl_3$) δ_B: 42.37 (br s); **HRMS** (ESI)$^+$ $C_{27}H_{24}BNONa$ $[M+Na]^+$ found 412.1837, requires 412.1843 (–1.46 ppm).

1-(1-benzyl-3,7-dimethylbenzo[e][1,2]azaborinin-2(1H)-yl)naphthalen-2-ol **34**

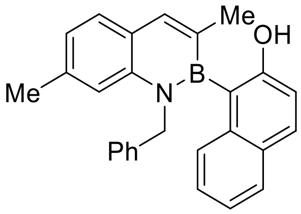

Following general procedure **C**, *N*-benzyl-5-methyl-2-(prop-1-en-1-yl)aniline **S15** (0.95 g, 4.0 mmol) and BCl_3 (1.0 M in hexane, 8.0 mL, 8.0 mmol) in PhMe (8 mL) for 18 h gave the chlorinated intermediate. 1-bromonaphthalen-2-ol (0.98 g, 4.4 mmol), nBuli (2.3 M in hexane, 5.0 mL, 11 mmol) in THF (22 mL) for 2 h, then the chlorinated intermediate in THF (8 mL) for 1 h gave, after purification by Biotage® Selekt™ (Sfär HC 100 g, 180 mL.min^{-1} Petrol : CH_2Cl_2, [85:15 to 70:30 8 CV, 70:30 to 50:50 10 CV]), the title compound **34** (0.73 g, 47%) as a pale yellow amorphous solid. **IR** ν_{max} (film) 3543, 2920, 1601, 1506, 1227, 1142, 905; **^{1}H NMR** (400 MHz, $CDCl_3$) δ_H: 2.05 (3H, d, *J* 1.4), 2.37 (3H, s), 4.58 (1H, s), 5.26 (2H, s), 7.03–7.09 (4H, m), 7.10–7.20 (3H, m), 7.25–7.26 (1H, m), 7.28–7.31 (2H, m), 7.39–7.42 (1H, m), 7.61 (1H, d, *J* 7.9), 7.75 (1H, d, *J* 8.8), 7.79–7.81 (1H, m), 7.93 (1H, s);

^{13}**C{^{1}H} NMR** (126 MHz, $CDCl_3$) δ_C: 21.7, 22.2, 53.1, 117.2, 117.7, 123.0, 123.3, 125.5, 126.2, 126.2, 126.8, 127.1, 128.5, 128.6, 129.2, 129.5, 129.9, 136.3, 137.6, 139.1, 140.6, 142.5, 154.3; ^{11}**B{^{1}H} NMR** (128 MHz, $CDCl_3$) δ_B: 40.49 (br s); **HRMS** (ESI)$^+$ $C_{27}H_{24}BNONa$ $[M+Na]^+$ found 412.1840, requires 412.1843 (–0.73 ppm).

1-(1,3-dimethylbenzo[*e*][1,2]azaborinin-2(1*H*)-yl)naphthalen-2-ol **36**

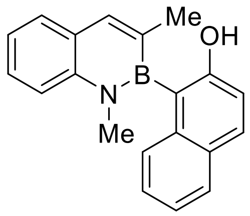

Following general procedure **C**, *N*-methyl-2-(prop-1-en-1-yl)aniline **S16** (0.40 g, 2.8 mmol) and BCl_3 (1.0 M in hexane, 5.7 mL, 5.7 mmol), in PhMe (6 mL) for 16 h gave the chlorinated intermediate. 1-bromonaphthalen-2-ol (0.70 g, 3.1 mmol) and nBuLi (2.3 M in hexane, 3.6 mL, 7.4 mmol) in THF (15 mL) for 2 hours, then the chlorinated intermediate in THF (6 mL) for 3 h gave, after purification by Biotage® Selekt™ (Sfär HC 50 g, 150 mL.min^{-1}, Petrol : CH_2Cl_2 [75:25 to 50:50 10 CV, 50:50 to 30:70 15 CV]), the title compound **36** (0.40 g, 47%) as a pale yellow amorphous solid. **IR** ν_{max} (film) 3528, 2955, 1611, 1337, 1223, 907, 812; ^{1}**H NMR** (400 MHz, $CDCl_3$) δ_H: 2.05 (3H, s), 3.58 (3H, s), 4.73 (1H, br s), 7.17 (1H, d, *J* 8.8), 7.30–7.36 (4H, m), 7.56 (1H, ddd, *J* 8.6, 6.9, 1.6), 7.65 (1H, d, *J* 8.6), 7.74 (1H, dd, *J* 7.8, 1.6), 7.85 (2H, t, *J* 8.7), 7.92 (1H, s); ^{13}**C{^{1}H} NMR** (126 MHz, $CDCl_3$) δ_C: 21.7, 37.0, 115.1, 117.6, 121.6, 123.3, 126.3, 127.2, 127.3, 127.7, 128.6, 129.2, 129.6, 130.0, 136.4, 141.5, 142.2, 154.2; ^{11}**B{^{1}H} NMR** (128 MHz, $CDCl_3$) δ_B: 37.54 (br s); **HRMS** (ESI$^+$) $C_{20}H_{18}BNONa$ $[M+Na]^+$ found 322.1373, requires 322.1374 (–0.17 ppm).

1-(1-ethyl-3-methylbenzo[*e*][1,2]azaborinin-2(1*H*)-yl)naphthalen-2-ol **38**

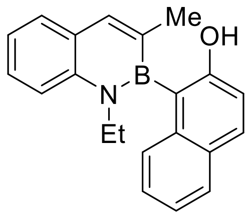

Following general procedure **C**, *N*-ethyl-2-(prop-1-en-1-yl)aniline **S17** (1.2 g, 7.3 mmol) and BCl_3 (1.0 M in hexane, 15 mL, 15 mmol), in PhMe (15 mL) for 18 h gave the chlorinated intermediate. 1-bromonaphthalen-2-ol (1.8 g, 8.1 mmol), nBuLi (2.3 M in hexane, 8.4 mL) and THF (41 mL) for 2 h, then the chlorinated intermediate in THF (15 mL) for 2 h gave, after purification by Biotage® Selekt™ (Sfär HC 100g, 250 mL.min^{-1}, Petrol : CH_2Cl_2 [20:80 to 50:50 10 CV, 50:50 to 30:70 10 CV]), the title compound **38** (1.4 g, 59%) as a white

amorphous solid. **IR** ν_{max} (film) 3528, 2974, 1614, 1375, 1246, 906, 812; **^{1}H NMR** (400 MHz, $CDCl_3$) δ_H: 1.24 (3H, t, *J* 7.1), 2.00 (3H, s), 4.07 (2H, app dp, *J* 18.9, 7.0), 4.63 (1H, s), 7.17 (1H, d, *J* 8.8), 7.28–7.36 (4H, m), 7.53 (1H, ddd, *J* 8.6, 7.0, 1.7), 7.65 (1H, d, *J* 8.6), 7.73 (1H, dd, *J* 7.8, 1.6), 7.83 (1H, d, *J* 8.8), 7.85 (1H, d, *J* 7.3), 7.90 (1H, s); **^{13}C{^{1}H} NMR** (126 MHz, $CDCl_3$) δ_C: 15.6, 21.6, 43.8, 115.4, 117.6, 121.3, 123.4, 126.2, 127.1, 127.6, 127.8, 128.5, 129.2, 129.8, 130.0, 136.3, 140.1, 142.3, 153.8; **^{11}B{^{1}H} NMR** (160 MHz, $CDCl_3$) δ_B: 38.20 (br s); **HRMS** (ESI$^+$) $C_{21}H_{20}BNONa$ [M+Na]$^+$ found 336.1532, requires 336.1530 (+0.48 ppm).

1-(1-allyl-3-methylbenzo[e][1,2]azaborinin-2(1H)-yl)naphthalen-2-ol **40**

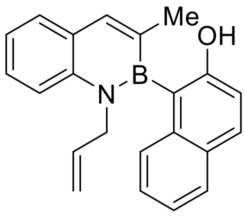

Following general procedure **C**, *N*-allyl-2-(prop-1-en-1-yl)aniline **S18** (1.0 g, 6.0 mmol) and BCl_3 (1.0 M in hexane, 12 mL, 12 mmol) in PhMe (12 mL) for 18 h gave the chlorinated intermediate. 1-bromonaphthalen-2-ol (1.5 g, 6.6 mmol), nBuLi (2.3 M in hexane, 6.8 mL, 16 mmol) in THF (33 mL) for 2 h, then the chlorinated intermediate in THF (12 mL) for 2 h gave, after purification by Biotage® Selekt™ (Sfär HC 100 g, 200 mL.min^{-1}, Petrol : EtOAc [100:0 to 90:10 10 CV, 90:10 to 70:30 15 CV]), the title compound **40** (0.50 g, 24%) as a pale yellow amorphous solid. **IR** ν_{max} (film) 3539, 2953, 1611, 1506, 1331, 1225, 907; **^{1}H NMR** (400 MHz, $CDCl_3$) δ_H: 2.00 (3H, d, *J* 1.3), 4.61 (1H, br s), 4.66 (2H, ddd, *J* 7.0, 5.1, 1.9), 4.93 (1H, dq, *J* 17.3, 1.7), 5.05 (1H, dq, *J* 10.5, 1.6), 5.90 (1H, ddt, *J* 17.4, 10.2, 5.0), 7.15 (1H, d, *J* 8.8), 7.27–7.35 (4H, m), 7.49 (1H, ddd, *J* 8.6, 7.1, 1.7), 7.60 (1H, d, *J* 8.6), 7.72 (1H, dd, *J* 7.8, 1.6), 7.81 (1H, d, *J* 9.0), 7.84 (1H, dd, *J* 7.3, 1.0), 7.92 (1H, s); **^{13}C{^{1}H} NMR** (101 MHz, $CDCl_3$) δ_C: 21.7, 51.7, 116.4, 116.4, 117.8, 121.6, 123.4, 126.3, 127.2, 127.5, 127.6, 128.6, 129.3, 129.8, 129.9, 135.5, 136.3, 140.4, 142.5, 154.1; **^{11}B{^{1}H} NMR** (128 MHz, $CDCl_3$) δ_B: 37.98 (br s); **HRMS** (ESI)$^+$ $C_{22}H_{20}BNONa$ [M+Na]$^+$ found 348.1529, requires 348.1530 (–0.21 ppm).

1-(1-(furan-2-ylmethyl)-3-methylbenzo[e][1,2]azaborinin-2(1H)-yl)naphthalen-2-ol **42**

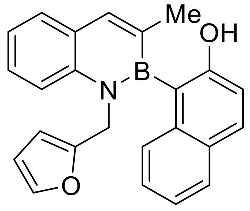

Following general procedure **C**, *N*-(furan-2-ylmethyl)-2-(prop-1-en-1-yl)aniline **S19** (1.5 mL, 7.0 mmol) and BCl_3 (1.0 M in hexane, 14 mL, 14 mmol) in PhMe (14 mL) for 18 h gave the chlorinated intermediate. 1-bromonaphthalen-2-ol (1.7 g, 7.7 mmol), nBuLi (2.1 M in hexane, 8.7 mL, 19 mmol), in THF (40 mL) for 2 h, then the chlorinated intermediate in THF (14 mL) for 2 h gave, after purification by Biotage® Selekt™ (Sfär HC 100 g, 180 mL.min^{-1} ,Petrol : EtOAc [100:0 to 90:10 15 CV, 90:10 to 75:25 10 CV]), the title compound **42** (0.50 g, 19%) as a white amorphous solid. **IR** ν_{max} (film) 3536, 2951, 1611, 1504, 1335, 1225, 907; **^{1}H NMR** (500 MHz, $CDCl_3$) δ_H: 2.01 (3H, d, *J* 1.3), 4.94 (1H, s), 5.22 (2H, s), 6.00 (1H, d, *J* 3.6), 6.19 (1H, dd, *J* 3.3, 1.8), 7.18 (1H, d, *J* 8.8), 7.22 (1H, d, *J* 1.8), 7.27–7.35 (4H, m), 7.46 (1H, ddd, *J* 8.7, 7.1, 1.7), 7.72 (2H, d, *J* 9.3), 7.83 (2H, app dd, *J* 9.6, 8.6), 7.92 (1H, s); **^{13}C{^{1}H} NMR** (126 MHz, $CDCl_3$) δ_C: 21.7, 46.5, 107.6, 110.5, 115.0, 118.1, 121.8, 123.4, 126.3, 127.0, 127.6, 127.7, 128.5, 129.3, 129.8, 130.1, 136.2, 140.3, 141.6, 142.7, 152.1, 154.5; **^{11}B{^{1}H} NMR** (128 MHz, $CDCl_3$) δ_B: 39.96 (br s); **HRMS** (ESI)$^+$ $C_{24}H_{20}BNO_2Na$ $[M+Na]^+$ found 388.1476, requires 388.1477 (–0.19 ppm).

1-(1-isopropyl-3-methylbenzo[*e*][1,2]azaborinin-2(1*H*)-yl)naphthalen-2-ol **44**

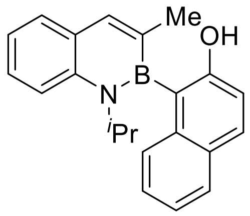

Following general procedure **C**, (*E*)-isopropyl-2-(prop-1-en-1-yl)aniline **S20** (0.40 g, 2.3 mmol) and BCl_3 (1.0 M in hexane, 4.6 mL, 4.6 mmol) in PhMe (5 mL) for 18 hours, gave the chlorinated intermediate.1-bromonaphthalen-2-ol (0.57 g, 2.5 mmol), nBuLi (1.7 M in hexane, 3.7 mL, 6.1 mmol) and THF (13) for 2 h, then the chlorinated intermediate in THF (5 mL) for 2 h gave, after purification by Biotage® Selekt™ (Sfär HC 50 g, 200 mL.min^{-1}, Petrol : CH_2Cl_2 [70:30 to 30:70 10 CV, 30:70 to 0:100 10 CV]), the title compound **44** (0.20 g, 26%) as a pale yellow amorphous solid. **IR** ν_{max} (film) 3537, 2932, 1612, 1300, 1258, 907; **^{1}H NMR** (400 MHz, $CDCl_3$) δ_H: 1.55 (3H, d, *J* 7.2), 1.67 (3H, d, *J* 7.2), 1.92 (3H, s), 4.56 (1H, s), 4.84 (1H, app p, *J* 7.2), 7.16 (1H, d, *J* 8.8), 7.25–7.37 (4H, m), 7.47 (1H, t, *J* 7.1), 7.72 (1H, dd, *J* 7.8, 1.8), 7.81 (1H, d, *J* 8.8), 7.83–7.86 (2H, m), 7.92 (1H, d, *J* 8.7); **^{13}C{^{1}H} NMR** (101 MHz, $CDCl_3$) δ_C: 21.7, 21.9, 22.2, 53.5, 117.6, 118.8, 121.1, 123.4,

126.2, 126.4, 127.2, 128.4, 128.8 (br s), 129.2, 129.6, 130.5, 136.2, 139.6 (br s), 142.6, 153.6; **^{11}B{^{1}H} NMR** (128 MHz, $CDCl_3$) δ_B: 37.72 (br s); **HRMS** (ESI$^+$) $C_{22}H_{22}BNONa$ [M+Na]$^+$ found 350.1687, requires 350.1687 (±0.0 ppm).

1-(1-cyclopropyl-3-methylbenzo[*e*][1,2]azaborinin-2(1*H*)-yl)naphthalen-2-ol **46**

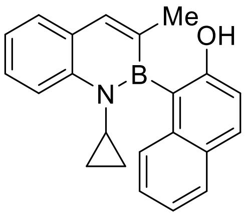

Following general procedure **C**, *N*-cyclopropyl-2-(prop-1-en-1-yl)aniline **S21** (0.95 g, 5.5 mmol) and BCl_3 (1.0 M in hexane, 11 mL, 11 mmol) in PhMe (11 mL) for 16 h gave the chlorinated intermediate. 1-bromonaphthalen-2-ol (1.4 g, 6.1 mmol) and nBuLi (2.3 M in hexane, 6.3 mL, 15 mmol) in THF (30 mL) for 2 h, then the chlorinated intermediate in THF (11 mL) for 16 h gave, after purification by Biotage® Selekt™ (Sfär HC 100 g, 200 mL.min^{-1}, Petrol : EtOAc [80:20 to 70:30 5 CV, 70:30 to 50:50 15 CV]), the title compound **46** (0.33 g, 18%) as an off-white amorphous solid. **IR** ν_{max} (film) 3402, 2916, 1611, 1429, 1321, 808; **^{1}H NMR** (400 MHz, $CDCl_3$) δ_H: 0.51–0.74 (4H, m), 2.04 (3H, d, *J* 1.3), 3.18 (1H, app ddd, *J* 11.2, 7.0, 4.3), 4.72 (1H, s), 7.13 (1H, d, *J* 8.8), 7.26–7.37 (4H, m), 7.54 (1H, ddd, *J* 8.7, 7.1, 1.7), 7.69 (1H, dd, *J* 7.7, 1.6), 7.81 (1H, d, *J* 8.8), 7.83–7.85 (1H, m), 7.87 (1H, s), 8.18 (1H, d, *J* 8.5); **^{13}C{^{1}H} NMR** (126 MHz, $CDCl_3$) δ_C: 10.1, 21.5, 31.8, 117.0, 117.4, 121.6, 123.1, 126.2, 127.1, 127.2, 127.3, 128.5, 129.1, 129.5, 129.7, 136.2, 142.4, 142.6, 153.5; **^{11}B{^{1}H} NMR** (160 MHz, $CDCl_3$) δ_B: 39.58 (br s); **HRMS** (ESI$^+$) $C_{22}H_{20}BNONa$ [M+Na]$^+$ found 348.1530, requires 348.1530 (–0.11 ppm).

1-(1-cyclobutyl-3-methylbenzo[e][1,2]azaborinin-2(1H)-yl)naphthalen-2-ol **48**

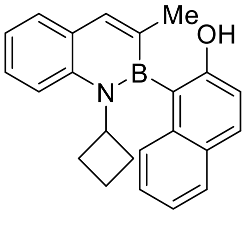

Following general procedure **C**, *N*-cyclobutyl-2-(prop-1-en-1-yl)aniline **S22** (1.4 g, 7.5 mmol) and BCl_3 (1.0 M in hexane, 15 mL, 15 mmol) in PhMe (15 mL) for 18 h gave the chlorinated intermediate. 1-bromonaphthalen-2-ol (1.8 g, 8.3 mmol) and nBuLi (2.3 M in hexane, 8.5 mL, 20 mmol) in THF (41 mL) for 2 h, then the chlorinated intermediate in THF

(15 mL) for 16 h gave, after purification by Biotage® Selekt™ (Sfär HC 100 g, 250 mL.min^{-1}, Petrol : CH_2Cl_2 [90:10 to 75:25 10 CV, 75:25 to 50:50 10 CV, 50:50 to 30:70 10 CV]), the title compound **48** (0.40 g, 17%) as a pale yellow amorphous solid. **IR** ν_{max} (film) 3536, 2951, 1611, 1429, 1333, 1254, 907; **^{1}H NMR** (400 MHz, $CDCl_3$) δ_H: 1.46–1.57 (2H, m), 1.95 (3H, d, *J* 1.3), 1.99–2.06 (1H, m), 2.15–2.28 (3H, m), 4.71 (1H, s), 4.91 (1H, tt, *J* 9.3, 7.0), 7.12 (1H, d, *J* 8.8), 7.26–7.37 (4H, m), 7.49 (1H, ddd, *J* 8.7, 7.1, 1.7), 7.70 (1H, dd, *J* 7.8, 1.7), 7.76 (1H, d, *J* 8.6), 7.81–7.84 (3H, m); **^{13}C{^{1}H} NMR** (101 MHz, $CDCl_3$) δ_C: 15.3, 21.5, 32.3, 56.7, 117.4, 121.4, 123.2, 126.3, 126.8, 127.5, 128.0, 128.5, 129.0, 129.8, 136.4, 140.4, 142.4, 153.6; **^{11}B{^{1}H} NMR** (128 MHz, $CDCl_3$) δ_B: 38.30 (br s); **HRMS** (ESI)$^+$ $C_{23}H_{22}BNONa$ [M+Na]$^+$ found 362.1687, requires 362.1687 (+0.13 ppm).

1-(1-(cyclohexylmethyl)-3-methylbenzo[e][1,2]azaborinin-2(1H)-yl)naphthalen-2-ol **50**

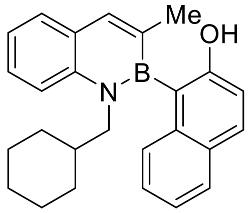

Following general procedure **C**, *N*-(cyclohexylmethyl)-2-(prop-1-en-1-yl)aniline **S23** (1.4 g, 6.1 mmol) and BCl_3 (1.0 M in hexane, 12 mL, 12 mmol) in PhMe (13 mL) for 18 h gave the chlorinated intermediate. 1-bromonaphthalen-2-ol (1.5 g, 6.7 mmol) and nBuLi (2.3 M in hexane, 7.0 mL, 16 mmol), in THF (34 mL) for 2 h, then the chlorinated intermediate in THF (13 mL) for 2 h gave, after purification by Biotage® Selekt™ (Sfär HC 100 g, 250 mL.min^{-1}, Petrol : CH_2Cl_2 [80:20 to 50:50 10 CV, 50:50 to 30:70 10 CV]) the title compound **50** (1.4 g, 60%) as a white amorphous solid. **IR** ν_{max} (film) 3543, 3055, 2922, 1609, 1443, 1333, 1225, 906; **^{1}H NMR** (400 MHz, $CDCl_3$) δ_H: 0.51–0.73 (2H, m), 0.87–1.07 (3H, m), 1.47–1.60 (5H, m), 1.81–1.91 (1H, m), 2.03 (3H, d, *J* 1.3), 3.84 (1H, dd, *J* 13.9, 7.6), 3.97 (1H, dd, *J* 13.9, 5.9), 4.73 (1H, s), 7.16 (1H, d, *J* 8.8), 7.28–7.36 (4H, m), 7.52 (1H, ddd, *J* 8.6, 7.0, 1.7), 7.60 (1H, d, *J* 8.5), 7.72 (1H, dd, *J* 7.8, 1.6), 7.83 (1H, d, *J* 8.7), 7.83–7.85 (1H, m), 7.89 (1H, s); **^{13}C{^{1}H} NMR** (101 MHz, $CDCl_3$) δ_C: 21.7, 26.0, 26.3, 30.8, 31.3, 36.8, 55.4, 116.3, 117.6, 121.3, 123.2, 126.1, 127.4, 127.4, 127.8, 128.5, 129.2, 130.0, 130.1, 136.2, 140.6, 142.6, 154.2; **^{11}B{^{1}H} NMR** (128 MHz, $CDCl_3$) δ_B: 39.25 (br s); **HRMS** (ESI)$^+$ $C_{26}H_{28}BNONa$ [M+Na]$^+$ found 404.2152, requires 404.2156 (–0.99 ppm).

1-(1-(cyclohexylmethyl)-3,4-dimethylbenzo[e][1,2]azaborinin-2(1H)-yl)naphthalen-2-ol **52**

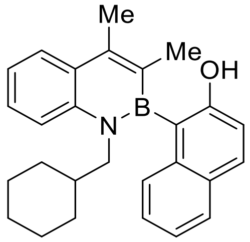

Following general procedure **C**, 2-(but-2-en-2-yl)-*N*-(cyclohexylmethyl)aniline **S24** (1.9 g, 7.9 mmol) and BCl_3 (1.0 M in hexane, 16 mL, 16 mmol) in PhMe (16 mL) for 18 h gave the chlorinated intermediate. 1-bromonaphthalen-2-ol (1.9 g, 8.7 mmol), nBuLi (2.1 M in hexane, 9.8 mL, 21 mmol) in THF (44 mL) for 2 h, then the chlorinated intermediate in THF (16 mL) for 1.5 h gave, after purification by Biotage® Selekt™ (Sfär HC 100 g, 180 mL.min^{-1}, Petrol : CH_2Cl_2 [80:20 to 50:50 15 CV, 50:50 to 30:70 10 CV]), the title compound **52** (1.7 g, 53%) as a white amorphous solid. **IR** ν_{max} (film) 3543, 2922, 2815, 1587, 1331, 1128, 908; **^{1}H NMR** (400 MHz, $CDCl_3$) δ_H: 0.52–0.61 (1H, m), 0.63–0.70 (1H, m), 0.84–1.04 (3H, m), 1.46–1.55 (5H, m), 1.80–1.88 (1H, m), 1.99 (3H, s), 2.58 (3H, s), 3.80 (1H, dd, *J* 13.8, 7.6), 3.94 (1H, dd, *J* 14.0, 5.8), 4.71 (1H, s), 7.17 (1H, d, *J* 8.8), 7.27–7.36 (4H, m), 7.53 (1H, ddd, *J* 8.5, 6.9, 1.6), 7.63 (1H, dd, *J* 8.6, 1.2), 7.81–7.85 (2H, m), 8.07 (1H, dd, *J* 8.2, 1.6); **^{13}C{^{1}H} NMR** (101 MHz, $CDCl_3$) δ_C: 16.0, 18.4, 26.0, 26.3, 30.8, 31.3, 36.8, 55.5, 116.7, 117.5, 121.1, 123.2, 126.1, 126.1, 127.0, 127.6, 128.3, 128.4, 129.2, 130.0, 136.4, 140.5, 146.8, 154.3; **^{11}B{^{1}H} NMR** (128 MHz, $CDCl_3$) δ_B: 40.27 (br s); **HRMS** (ESI)$^+$ $C_{27}H_{30}BNONa$ [M+Na]$^+$ found 418.2307, requires 418.2313 (–1.45 ppm).

1-(1-(cyclohexylmethyl)benzo[e][1,2]azaborinin-2(1H)-yl)naphthalen-2-ol **54**

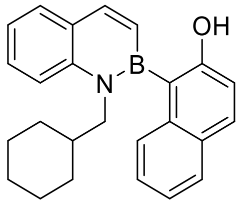

Following general procedure **C**, *N*-(cyclohexylmethyl)-2-vinylaniline **S25** (1.7 g, 7.9 mmol) and BCl_3 (1.0 M in hexane, 16 mL, 16 mmol) in PhMe (16 mL) for 18 h gave the chlorinated intermediate. 1-bromonaphthalen-2-ol (1.9 g, 8.6 mmol), nBuLi (2.1 M in hexane, 9.8 mL, 21 mmol) in THF (44 mL) for 2 h, then the chlorinated intermediate in THF (16 mL) for 3 h gave, after purification by Biotage® Selekt™ (Sfär HC 100 g, 200 mL.min^{-1}, Petrol : CH_2Cl_2, [90:10 to 70:30 10 CV, 70:30 to 50:50 10 CV]), the title compound **54** (0.50 g, 17%) as a white amorphous solid. **IR** ν_{max} (film) 3539, 2922, 1589, 1333, 1130, 905; **^{1}H NMR** (500 MHz, $CDCl_3$) δ_H: 0.55 (1H, q, *J* 10.7), 0.62–0.72 (1H, m), 0.87–1.07 (3H, m), 1.47–1.59

(5H, m), 1.81–1.90 (1H, m), 3.96 (1H, dd, *J* 13.9, 7.4), 4.06 (1H, dd, *J* 13.8, 6.2), 4.99 (1H, s), 7.05 (1H, d, *J* 11.2), 7.18 (1H, d, *J* 8.8), 7.33–7.35 (3H, m), 7.52–7.55 (1H, m), 7.61 (1H, ddd, *J* 8.6, 6.9, 1.7), 7.68 (1H, d, *J* 8.6), 7.79 (1H, dd, *J* 7.8, 1.7), 7.83–7.85 (2H, m), 8.15 (1H, d, *J* 11.2); **^{13}C{^{1}H} NMR** (126 MHz, $CDCl_3$) δ_C: 25.9, 25.9, 26.2, 30.7, 31.1, 36.5, 55.3, 116.6, 117.7, 121.3, 123.2, 126.2, 127.6, 128.0, 128.5, 128.6, 129.2, 130.4, 131.0, 131.4 (br s), 136.3, 141.8, 145.9, 154.7; **^{11}B{^{1}H} NMR** (128 MHz, $CDCl_3$) δ_B: 38.13 (br s); **HRMS** (ESI)$^+$ $C_{25}H_{26}BNONa$ [M+Na]$^+$ found 390.1998, requires 390.2000 (–0.48 ppm).

1-(1-cyclopentyl-3,4-dimethylbenzo[e][1,2]azaborinin-2(1H)-yl)naphthalen-2-ol **56**

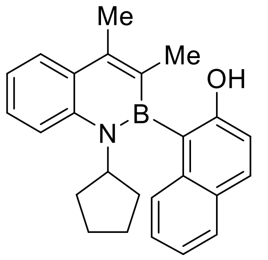

Following general procedure **C**, 2-(but-2-en-2-yl)-*N*-cyclopentylaniline **S26** (1.4 g, 6.5 mmol) and BCl_3 (1.0 M in hexane, 13 mL, 13 mmol) in PhMe (13 mL) for 18 h gave the chlorinated intermediate. 1-bromonaphthalen-2-ol (1.6 g, 7.2 mmol), nBuLi (2.1 M in hexane, 8.1 mL, 17 mmol) in THF (36 mL) for 2 h , then the chlorinated intermediate in THF (13 mL) for 2 h gave, after purification by Biotage® Selekt™ (Sfär HC 100 g, 150 mL.min^{-1}, Petrol : CH_2Cl_2 [90:10 to 70:30 10 CV, 70:30 to 50:50 10 CV]), the title compound **56** (0.60 g, 25%) as an off-white amorphous solid. **IR** ν_{max} (film) 3539, 2955, 1589, 1314, 1180, 908; **^{1}H NMR** (400 MHz, $CDCl_3$) δ_H: 1.46–1.55 (2H, m), 1.62–1.69 (1H, m), 1.86–2.01 (6H, m), 2.37–2.48 (2H, m), 2.56 (3H, s), 4.59 (1H, s), 4.97 (1H, p, *J* 9.5), 7.17 (1H, d, *J* 8.8), 7.27–7.34 (3H, m), 7.37 (1H, dd, *J* 8.1, 0.9), 7.46 (1H, ddd, *J* 8.6, 6.9, 1.7), 7.69 (1H, dd, *J* 8.7, 1.2), 7.81 (1H, d, *J* 8.8), 7.84 (1H, dd, *J* 8.3, 1.4), 8.10 (1H, dd, *J* 8.2, 1.6); **^{13}C{^{1}H} NMR** (101 MHz, $CDCl_3$) δ_C: 16.0, 18.4, 25.7, 25.8, 29.3, 29.8, 63.0, 117.6, 118.6, 120.9, 123.3, 125.8, 126.0, 126.7, 127.5, 128.4, 129.2, 129.2, 129.5, 136.4, 138.6, 146.7, 153.7; **^{11}B{^{1}H} NMR** (160 MHz, $CDCl_3$) δ_B: 39.38 (br s); **HRMS** (ESI)$^+$ $C_{25}H_{26}BNONa$ [M+Na]$^+$ found 390.1994, requires 390.2000 (–1.53 ppm).

2-(1-benzyl-3-methylbenzo[e][1,2]azaborinin-2(1H)-yl)-3-methylphenol **58**

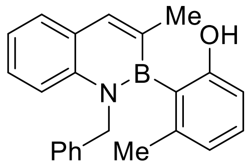

Following general procedure **C**, *N*-benzyl-2-(prop-1-en-1-yl)aniline **S10** (1.1 g, 4.9 mmol) and BCl_3 (1.0 M in hexane, 9.7 mL, 9.7 mmol) in PhMe (10 mL) for 18 h gave the chlorinated intermediate. 2-bromo-3-methylphenol (1.0 g, 5.3 mmol), nBuLi (2.1 M in hexane, 6.0 mL, 13 mmol) in THF (27 mL) for 2 h, then the chlorinated intermediate in THF (10 mL) for 2.5 h gave, after purification by Biotage® Selekt™ (Sfär HC 100 g, 220 mL.min^{-1}, Petrol : EtOAc, [100:0 to 90:10 10 CV, 90:10 to 75:25 10 CV]), the title compound **58** (0.60 g, 36%) as a white solid. **mp** 145–147 °C; **IR** ν_{max} (film) 3536, 2953, 1607, 1451, 1375, 1242, 905; **^{1}H NMR** (400 MHz, $CDCl_3$) δ_H: 2.10 (3H, s), 2.13 (3H, d, *J* 1.3), 4.40 (1H, s), 5.26 (1H, d, *J* 16.4), 5.34 (1H, d, *J* 16.4), 6.59 (1H, dd, *J* 8.0, 0.9), 6.81 (1H, dt, *J* 7.5, 0.8), 7.09–7.13 (3H, m), 7.14–7.24 (4H, m), 7.29 (1H, ddd, *J* 8.6, 7.0, 1.7), 7.36 (1H, d, *J* 8.5), 7.68 (1H, dd, *J* 7.7, 1.6), 7.90 (1H, s); **^{13}C{^{1}H} NMR** (126 MHz, $CDCl_3$) δ_C: 21.8, 22.1, 52.8, 112.0, 117.0, 121.3, 122.2, 126.2, 126.8, 127.3, 127.5, 128.6, 128.9, 129.6, 139.0, 140.3, 141.1, 142.2, 156.4; **^{11}B{^{1}H} NMR** (128 MHz, $CDCl_3$) δ_B: 38.99 (br s); **HRMS** (ESI)$^+$ $C_{23}H_{22}BNONa$ [M+Na]$^+$ found 362.1683, requires 362.1687 (–0.98 ppm).

1-(1-benzyl-3-methylbenzo[e][1,2]azaborinin-2(1H)-yl)-7-methoxynaphthalen-2-ol **60**

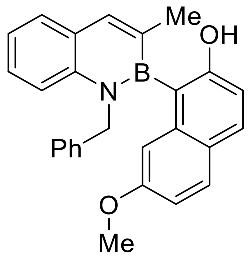

Following general procedure **C**, *N*-benzyl-2-(prop-1-en-1-yl)aniline **S10** (1.0 g, 4.5 mmol) and BCl_3 (1.0 M in hexane, 9.1 mL, 9.1 mmol) in PhMe (9 mL) for 18 h gave the chlorinated intermediate. 1-bromo-7-methoxynaphthalen-2-ol **S29** (1.3 g, 5.0 mmol), nBuLi (2.1 M in hexane, 5.6 mL, 12 mmol) in THF (25 mL) for 2 h, then the chlorinated intermediate in THF (9 mL) for 2 h gave, after purification by Biotage® Selekt™ (Sfär HC 100 g, 120 mL.min^{-1}, Petrol : EtOAc, [100:0 to 90:10 10 CV, 90:10 to 75:25 10 CV]), the title compound **60** (0.90 g, 51%) as an off-white amorphous solid. **IR** ν_{max} (film) 3283, 2974, 1611, 1508, 1217, 1032; **^{1}H NMR** (400 MHz, $CDCl_3$) δ_H: 2.09 (3H, s), 3.68 (3H, s), 4.54 (1H, s), 5.33 (2H, s), 6.72 (1H, d, *J* 2.5), 6.89 (1H, d, *J* 8.7), 6.98 (1H, dd, *J* 8.9, 2.5), 7.06–7.19 (5H, m), 7.22–7.26 (1H, m), 7.33 (1H, ddd, *J* 8.6, 7.0, 1.7), 7.42 (1H, d, *J* 8.5), 7.68 (2H, app t, *J* 8.9), 7.72 (1H, dd, *J* 7.8, 1.6), 7.96 (1H, s); **^{13}C{^{1}H} NMR** (126 MHz, $CDCl_3$) δ_C: 21.8, 53.1, 55.3,

105.9, 115.2, 115.5, 117.1, 121.5, 124.6, 126.2, 126.8, 127.4, 127.8, 128.6, 129.7, 129.7, 129.9, 137.7, 139.0, 140.5, 142.6, 154.9, 158.1; **^{11}B{^{1}H} NMR** (128 MHz, $CDCl_3$) δ_B: 40.90 (br s); **HRMS** (ESI)$^+$ $C_{27}H_{24}BNO_2Na$ [M+Na]$^+$ found 428.1790, requires 428.1792 (–0.43 ppm).

1-(1-(4-methoxybenzyl)-3-methylbenzo[e][1,2]azaborinin-2(1H)-yl)naphthalen-2-ol **62**

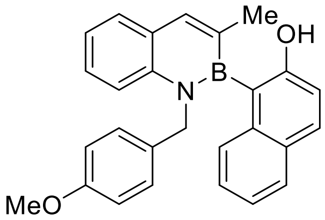

Following general procedure **C**, *N*-(4-methoxybenzyl)-2-(prop-1-en-1-yl)aniline **S27** (2.4 g, 9.4 mmol) and BCl_3 (1.0 M in CH_2Cl_2, 19 mL, 19 mmol) in PhMe (19 mL) for 16 h gave the chlorinated intermediate. 1-bromonaphthalen-2-ol (2.3 g, 10.3 mmol), nBuLi (2.1 M in hexane, 12 mL, 25 mmol) in THF (52 mL) for 2 h, then the chlorinated intermediate in THF (19 mL) for 3 h gave, after purification by Biotage® Selekt™ (Sfär HC 50 g, 150 mL.min^{-1}, Petrol : CH_2Cl_2 [75:25 to 50:50 10 CV, 50:50 to 30:70 15 CV]), the title compound **62** (0.88 g, 23%) as a pale brown amorphous solid. **IR** ν_{max} (film) 3387, 2955, 1611, 1510, 1248, 746; **^{1}H NMR** (400 MHz, $CDCl_3$) δ_H: 2.06 (3H, d, *J* 1.3), 3.70 (3H, s), 4.66 (1H, s), 5.21 (2H, d, *J* 1.8), 6.68–6.72 (2H, m), 6.97–6.99 (2H, m), 7.05 (1H, d, *J* 8.8), 7.21–7.25 (1H, m), 7.29–7.37 (3H, m), 7.39–7.45 (2H, m), 7.71 (1H, dd, *J* 7.7, 1.7), 7.75 (1H, d, *J* 8.8), 7.79–7.81 (1H, m), 7.95 (1H, s); **^{13}C{^{1}H} NMR** (126 MHz, $CDCl_3$) δ_C: 21.8, 52.6, 55.3, 114.0, 117.1, 117.7, 121.5, 123.4, 126.3, 127.1, 127.3, 127.5, 127.8, 128.5, 129.2, 129.7, 129.9, 131.0, 136.3, 140.5, 142.5, 154.3, 158.4; **^{11}B{^{1}H} NMR** (160 MHz, $CDCl_3$) δ_B: 38.28 (br s); **HRMS** (ESI)$^+$ $C_{27}H_{24}BNO_2Na$ [M+Na]$^+$ found 428.1789, requires 428.1792 (–0.71 ppm).

1-(3-methyl-1-phenylbenzo[*e*][1,2]azaborinin-2(1*H*)-yl)naphthalen-2-ol **64**

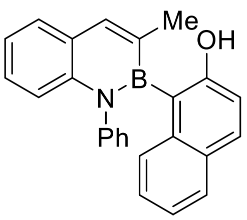

Following general procedure **C**, *N*-phenyl-2-(prop-1-en-1-yl)aniline **S28** (1.4 g, 6.5 mmol) and BCl_3 (1.0 M in hexane, 13 mL, 13 mmol) in PhMe (13 mL) for 18 h gave the chlorinated intermediate. 1-bromonaphthalen-2-ol (16 g, 7.2 mmol), nBuLi (2.3 M in hexane, 7.4 mL, 17 mmol) in THF (36 mL) for 2 h, then the chlorinated intermediate in THF (13 mL) for 4 h gave, after purification by Biotage® Selekt™ (Sfär HC 100g, 180 mL.min^{-1}, Petrol : CH_2Cl_2

[80:20 to 50:50 10 CV, 50:50 to 30:70 10 CV, 30:70 to 0:100 5 CV]), the title compound **64** (0.53 g, 22%) as a pale yellow amorphous solid. **IR** ν_{max} (film) 3537, 3057, 1595, 1491, 1294, 907; **^{1}H NMR** (400 MHz, $CDCl_3$) δ_H: 2.07 (3H, s), 4.50 (1H, s), 6.85 (1H, d, *J* 8.8), 6.89 (1H, d, *J* 7.0), 6.97 (2H, d, *J* 4.6), 7.07 (1H, dt, *J* 9.9, 4.1), 7.21–7.33 (6H, m), 7.40 (1H, d, *J* 8.1), 7.56 (1H, d, *J* 8.8), 7.67 (1H, d, *J* 7.4), 7.74 (1H, dd *J* 7.1, 2.3), 7.99 (1H, s); **^{13}C{^{1}H} NMR** (126 MHz, $CDCl_3$) δ_C: 21.7, 117.1, 117.6, 121.7, 123.0, 125.8, 126.7, 127.0, 127.3, 127.7, 127.8, 128.2, 128.7, 128.7, 129.2, 129.5, 136.3, 141.6, 142.3, 143.9, 153.2; **^{11}B{^{1}H} NMR** (128 MHz, $CDCl_3$) δ_B: 39.31 (br s); **HRMS** (ESI$^+$) $C_{25}H_{20}BNONa$ $[M+Na]^+$ found 384.1530, requires 384.1530 (–0.01 ppm).

1-(3-bromo-1-(cyclohexylmethyl)benzo[e][1,2]azaborinin-2(1H)-yl)naphthalen-2-ol **66**

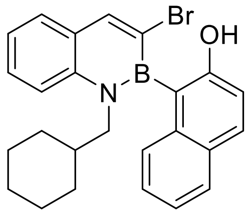

Following literature[101] and under nitrogen, 1-(1-(cyclohexylmethyl)benzo[e][1,2]azaborinin-2(1H)-yl)naphthalen-2-ol **54** (0.28 g, 0.80 mmol, 1.0 equiv.) in CH_2Cl_2 (4 mL) were set to –20 °C. Br_2 (43 µL, 0.88 mmol, 1.1 equiv.) in CH_2Cl_2 (4 mL) was added dropwise over 25 mins, and the solution was stirred at –20 °C for 40 mins, then warmed to RT. The mixture was stirred at RT for 1 h, then quenched dropwise with aq. $Na_2S_2O_4$, diluted with CH_2Cl_2 and the organic layer was extracted, dried (Na_2SO_4) and concentrated under reduced pressure. The crude product was purified by Biotage® Selekt™ (Sfär HC 50 g, 160 mL.min^{-1}, Petrol : CH_2Cl_2 [85:15 to 70:30 10 CV, 70:30 to 40:60 10 CV]), to give the title compound **66** (57.8 mg, 17%) as a white amorphous solid. **IR** ν_{max} (film) 3545, 2922, 1589, 1333, 1140, 907; **^{1}H NMR** (400 MHz, $CDCl_3$) δ_H: 0.54–0.70 (2H, m), 0.90–1.05 (3H, m), 1.48–1.62 (5H, m), 1.77–1.83 (1H, m), 3.83 (1H, dd, *J* 13.9, 8.0), 3.99 (1H, dd, *J* 14.0, 5.5), 4.85 (1H, br s), 7.15 (1H, d, *J* 8.8), 7.31–7.35 (4H, m), 7.61–7.62 (2H, m), 7.71 (1H, d, *J* 7.8), 7.82–7.86 (2H, m), 8.45 (1H, s); **^{13}C{^{1}H} NMR** (126 MHz, $CDCl_3$) δ_C: 25.9, 26.0, 26.2, 30.7, 31.2, 37.0, 56.2, 116.8, 117.9, 122.0, 123.4, 126.4, 127.2, 127.3, 128.6, 128.9, 129.1, 130.2, 130.7, 136.1, 140.8, 146.2, 154.7; **^{11}B{^{1}H} NMR** (128 MHz, $CDCl_3$) δ_B: 36.91 (br s); **HRMS** (ESI)$^+$ $C_{25}H_{25}B^{79}BrNONa$ $[M+Na]^+$ found 468.1098, requires 468.1105 (–1.54 ppm).

4-(1-benzyl-3-methylbenzo[*e*][1,2]azaborinin-2(1*H*)-yl)naphthalen-2-ol **68**

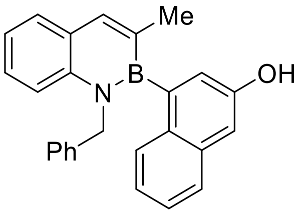

Following general procedure **C**, *N*-benzyl-2-(prop-1-en-1-yl)aniline **S10** (1.3 g, 5.4 mmol) and BCl_3 (1.0 M in hexane, 11 mL, 11 mmol) in PhMe (11 mL) for 18 h gave the chlorinated intermediate. 4-bromonaphthalen-2-ol (1.3 g, 5.9 mmol), nBuLi (2.3 M in hexane, 6.1 mL, 14 mmol) in THF (30 mL) for 2 h, then the chlorinated intermediate in THF (11 mL) for 2 h gave, after purification by Biotage® Selekt™ (Sfär HC 100 g, 120 mL.min^{-1}, Petrol : CH_2Cl_2 [80:20 to 50:50 10 CV, 50:50 to 30:70 10 CV]), the title compound **68** (0.32 g, 16%) as a pale brown amorphous solid. **IR** ν_{max} (film) 3516, 3030, 2951, 1611, 1595, 1236, 1169, 910; **^{1}H NMR** (400 MHz, $CDCl_3$) δ_H: 2.01 (3H, d, *J* 1.3), 4.76 (1H, s), 5.15 (1H, d, *J* 16.6), 5.24 (1H, d, *J* 16.6), 7.00 (1H, s), 7.01 (2H, app d, *J* 2.7), 7.11 (1H, d, *J* 2.6), 7.15–7.25 (5H, m), 7.29–7.35 (2H, m), 7.40 (1H, ddd, *J* 8.2, 6.7, 1.2), 7.50–7.53 (1H, m), 7.71 (2H, d, *J* 8.0), 7.92 (1H, s); **^{13}C{^{1}H} NMR** (126 MHz, $CDCl_3$) δ_C: 22.0, 53.2, 109.2, 117.0, 120.2, 121.5, 123.5, 125.9, 126.3, 126.8, 127.2, 127.5, 127.6, 128.4, 128.7, 129.6, 130.6, 134.8 139.0, 140.1, 142.4, 152.9; **^{11}B{^{1}H}** (128 MHz, $CDCl_3$) δ_B: 38.74 (br s); **HRMS** (ESI$^+$) $C_{26}H_{22}BNONa$ $[M+Na]^+$ found 398.1687, requires 398.1687 (+0.17 ppm).

(1-(1-benzyl-3-methylbenzo[e][1,2]azaborinin-2(1H)-yl)naphthalen-2-yl)methanol **70**

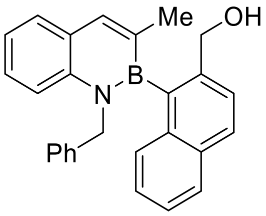

Following general procedure **C**, *N*-benzyl-2-(prop-1-en-1-yl)aniline **S10** (0.95 g, 4.3 mmol), BCl_3 (1.0 M in hexane, 8.5 mL, 8.5 mmol) in PhMe (9.0 mL) for 18 h gave the chlorinated intermediate. (1-bromonaphthalen-2-yl)methanol **S30** (1.1 g, 4.7 mmol), nBuLi (2.1 M in hexane, 5.3 mL, 11 mmol) in THF (23 mL) for 2 h, then the chlorinated intermediate in THF (9 mL) for 2 h gave, after purification by Biotage® Selekt™ (Sfär HC 100 g, 180 mL.min^{-1}, Petrol : CH_2Cl_2 [90:10 to 75:25 10 CV, 75:25 to 30:70 15 CV]), the title compound **70** (0.16 g, 10%) as a pale yellow amorphous solid. **IR** ν_{max} (film) 3348, 2928, 1610, 1348, 1221, 907; **^{1}H NMR** (400 MHz, $CDCl_3$) δ_H: 1.95 (3H, d, *J* 1.3), 4.36 (1H, d, *J* 12.7), 4.54 (1H, dd, *J* 12.7, 4.7), 5.03 (1H, d, *J* 16.9), 5.24 (1H, d, *J* 16.9), 6.91 (2H, d, *J* 8.2), 7.13–7.21 (3H, m), 7.25–7.49 (6H, m), 7.55 (1H, d, *J* 8.5), 7.75 (1H, dd, *J* 7.7, 1.5), 7.87 (2H, t, *J* 8.0), 7.92 (1H, s); **^{13}C{^{1}H} NMR** (126 MHz, $CDCl_3$) δ_C: 22.0, 52.6, 66.0, 116.7, 121.5,

125.7, 125.8, 125.9, 126.0, 127.0, 127.4, 127.6, 128.1, 128.3, 128.5, 128.7, 129.7, 132.8, 134.7, 138.1 (br s), 138.8, 140.4, 140.5, 141.9; **^{11}B{^{1}H} NMR** (128 MHz, $CDCl_3$) δ_B: 39.74 (br s); **HRMS** (ESI)$^+$ $C_{27}H_{24}BNONa$ $[M+Na]^+$ found 412.1840, requires 412.1843 (–0.70 ppm).

7. Kinetic Resolution data

7.1. Experimental data and HPLC traces

(*S*)-1-(1-benzylbenzo[e][1,2]azaborinin-2(1H)-yl)naphthalen-2-yl 2,2-diphenylacetate **25**

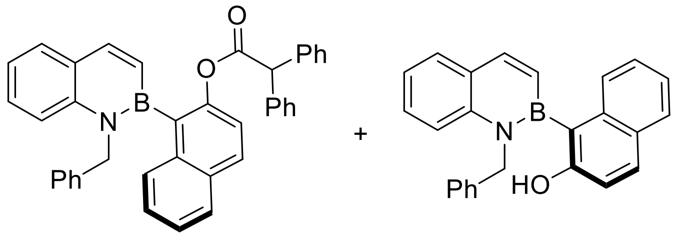

c = 51
s = 25

Following general procedure **E**, 1-(1-benzylbenzo[e][1,2]azaborinin-2(1H)-yl)naphthalen-2-ol **24** (145 mg, 0.40 mmol), 2,2-diphenylacetic pivalic anhydride **13** (82.9 mg, 0.28 mmol), (*R*)-BTM **14** (5.1 mg, 0.020 mmol), Et_3N (33.5 µL, 0.24 mmol) in $CHCl_3$ (4 mL) for 16 h gave, after purification on boron-capped silica by Biotage® Selekt™ (Sfär HC 25 g, 80 mL.min^{-1}, Petrol : EtOAc [100:0 to 90:10 10 CV, 90:10 to 75:25 10 CV]), the title ester **25** (113 mg, 51%) as a white amorphous solid. $[\alpha]_D^{20}$ +3.6 (*c* 1.3, $CHCl_3$); **Chiral HPLC analysis**, Chiralpak IB (99.5:0.5 hexane : IPA, flow rate 1.0 mL.min^{-1}, 254 nm, 30 °C) t_R (*S*): 11.2 min, t_R (*R*): 15.5 min, 91:9 er; **IR** ν_{max} (film) 3028, 1748, 1591, 1452, 1184, 1119, 907; **^{1}H NMR** (400 MHz, $CDCl_3$) δ_H: 4.92 (1H, d, *J* 16.6), 5.05 (1H, s), 5.18 (1H, d, *J* 16.6), 6.89 (2H, app dd, *J* 6.6, 2.9), 6.96 (1H, d, *J* 11.2), 7.04–7.20 (14H, m), 7.28–7.31 (3H, m), 7.38 (2H, app ddd, *J* 10.4, 8.4, 6.9), 7.59 (1H, d, *J* 8.4), 7.77 (1H, d, *J* 7.7), 7.82 (2H, t, *J* 7.8), 8.07 (1H, d, *J* 11.3); **^{13}C{^{1}H} NMR** (126 MHz, $CDCl_3$) δ_C: 53.0, 57.4, 117.5, 121.3, 121.4, 125.4, 126.1, 126.1, 126.7, 127.3, 127.9, 128.5, 128.5, 128.6, 128.7, 128.7, 128.7, 128.7, 129.6, 130.5, 131.3 (br s), 131.7, 136.1, 137.9, 138.2, 138.6, 141.4, 145.4, 149.9, 171.4; **^{11}B{^{1}H} NMR** (128 MHz, $CDCl_3$) δ_B: 36.86 (br s); **HRMS** (ESI)$^+$ $C_{39}H_{30}BNO_2Na$ [M+Na]$^+$ found 578.2255, requires 578.2262 (–1.18 ppm).

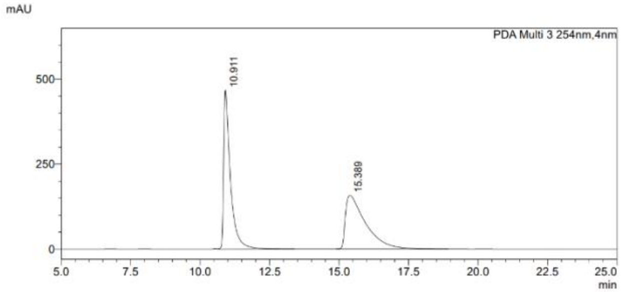

<Peak Table>

PDA Ch3 254nm

Peak#	Ret. Time	Area%
1	10.911	50.067
2	15.389	49.933
Total		100.000

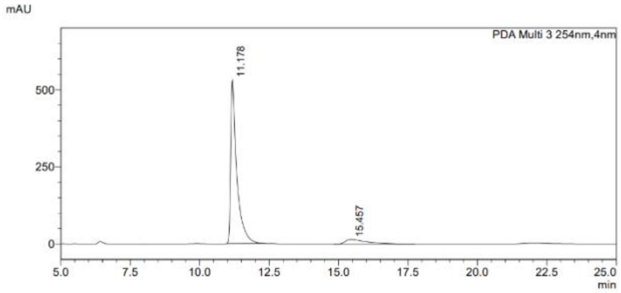

<Peak Table>

PDA Ch3 254nm

Peak#	Ret. Time	Area%
1	11.178	90.548
2	15.457	9.452
Total		100.000

The title alcohol (*R*)-1-(1-benzylbenzo[e][1,2]azaborinin-2(1H)-yl)naphthalen-2-ol **24** (67.2 mg, 47%): $[\alpha]_D^{20}$ +138.5 (*c* 1.7 $CHCl_3$); **Chiral HPLC analysis**, Chiralpak IA (90:10 hexane : IPA, flow rate 1.0 mL.min^{-1}, 254 nm, 30 °C) t_R (*S*): 19.5 min, t_R (*R*): 26.5 min, 8:92 er.

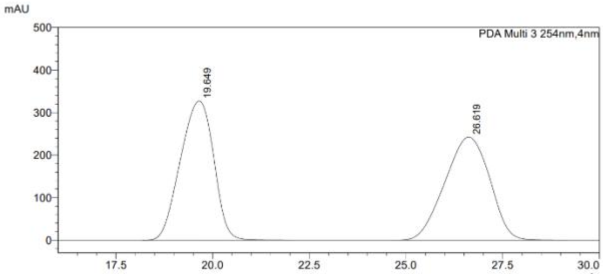

<Peak Table>

PDA Ch3 254nm

Peak#	Ret. Time	Area%
1	19.649	50.058
2	26.619	49.942
Total		100.000

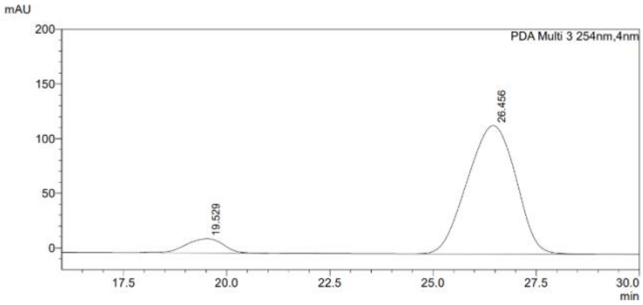

<Peak Table>

PDA Ch3 254nm

Peak#	Ret. Time	Area%
1	19.529	7.728
2	26.456	92.272
Total		100.000

(*S*)-1-(1-benzyl-3-methylbenzo[*e*][1,2]azaborinin-2(1*H*)-yl)naphthalen-2-yl
2,2 diphenylacetate **16**

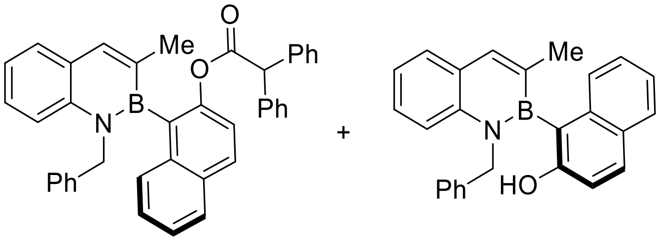

c = 49
s = 50

Following general procedure **E**, 1-(1-benzyl-3-methylbenzo[*e*][1,2]azaborinin-2(1*H*)-yl)naphthalen-2-ol **15** (2.5 g, 6.7 mmol), 2,2-diphenylacetic pivalic anhydride **13** (1.4 g, 4.7 mmol), (*R*)-BTM **14** (84.5 mg, 0.34 mmol), Et_3N (0.56 mL, 4.0 mmol) in $CHCl_3$ (67 mL) for 16 h gave, after purification by Biotage® Selekt™ (Sfär HC 100 g, 180 mL.min^{-1}, Petrol : EtOAc [100:0 to 90:10 10 CV, 90:10 to 75:25 10 CV]), the title ester **16** (1.7 g, 45%) as a white amorphous solid. $[\alpha]_D^{20}$ +25.1 (*c* 0.4, $CHCl_3$); **Chiral HPLC analysis**, Chiralpak IA (99:1 hexane : IPA, flow rate 1.0 mL.min^{-1}, 211 nm, 30 °C) t_R (*R*): 16.5 min, t_R (*S*): 26.0 min, 5:95 er. **IR** ν_{max} (film) 2019, 1751, 1611, 1495, 1192, 1130, 908, 731; **^{1}H NMR** (400 MHz, $CDCl_3$) δ_H: 1.94 (3H, d, *J* 1.1), 4.88 (1H, d, *J* 16.7), 5.04 (1H, s), 5.15 (1H, d, *J* 16.7), 6.86–6.89 (2H, m), 7.04–7.16 (13H, m), 7.24 (2H, app d, *J* 8.9), 7.27–7.34 (3H, m), 7.39 (1H, ddd, *J* 8.1, 6.9, 1.1), 7.50 (1H, d, *J* 8.3), 7.73 (1H, ddd, *J* 7.5, 1.7), 7.83 (3H, app dd, *J* 8.5, 2.7); **^{13}C{^{1}H} NMR** (101 MHz, $CDCl_3$) δ_C : 21.7, 53.1, 57.4, 117.3, 121.3, 121.4, 125.5, 126.1, 126.1, 126.5, 127.3, 127.3, 127.7, 128.3, 128.4, 128.5, 128.6, 128.6, 128.7, 129.4, 129.6, 131.6, 135.8, 137.9, 138.1, 138.7, 140.3, 142.3, 149.7, 171.4; **^{11}B{^{1}H} NMR** (128 MHz, $CDCl_3$) δ_B: 38.45 (br s); **HRMS** (ESI)$^+$ $C_{40}H_{32}BNO_2Na$ [M+Na]$^+$ found 592.2422, requires 592.2418 (+0.68 ppm).

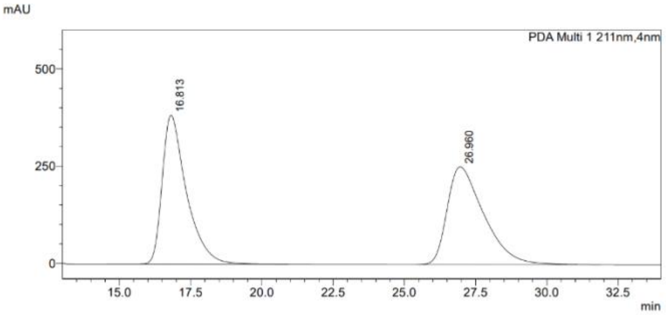

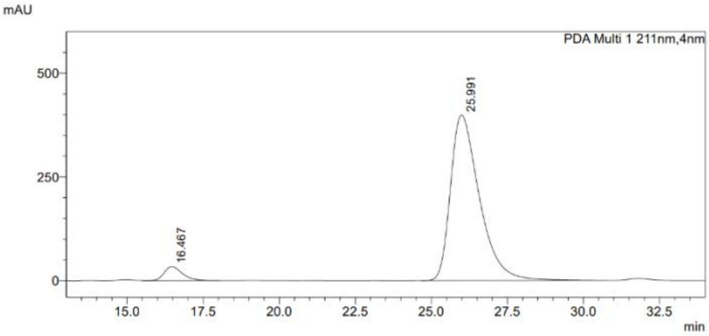

<Peak Table>

PDA Ch1 211nm

Peak#	Ret. Time	Area%
1	16.813	50.169
2	26.960	49.831
Total		100.000

<Peak Table>

PDA Ch1 211nm

Peak#	Ret. Time	Area%
1	16.467	5.138
2	25.991	94.862
Total		100.000

The title alcohol (*R*)-1-(1-benzyl-3-methylbenzo[e][1,2]azaborinin-2(1H)-yl)naphthalen-2-ol **15**: (1.3 g, 50%): $[\alpha]_D^{20}$ +217.0 (*c* 0.4, $CHCl_3$); **Chiral HPLC analysis**, Chiralpak IA (95:5 hexane : IPA, flow rate 1.0 mL.min^{-1}, 211 nm, 30 °C) t_R (*R*): 20.8 min, t_R (*S*): 24.3 min, 92:8 er.

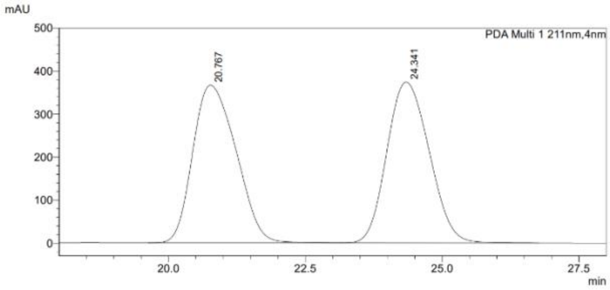

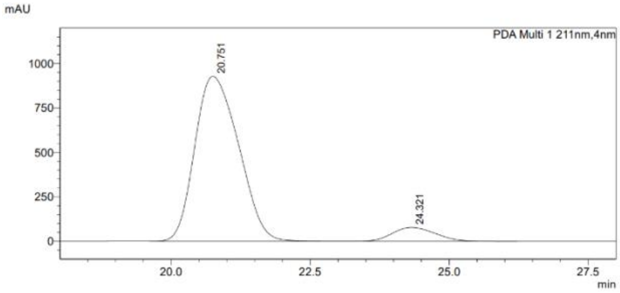

<Peak Table>

PDA Ch1 211nm

Peak#	Ret. Time	Area%
1	20.767	50.048
2	24.341	49.952
Total		100.000

<Peak Table>

PDA Ch1 211nm

Peak#	Ret. Time	Area%
1	20.751	92.366
2	24.321	7.634
Total		100.000

(*S*)-1-(1-benzyl-3-methylbenzo[*e*][1,2]azaborinin-2(1*H*)-yl)naphthalen-2-yl isobutyrate **17**

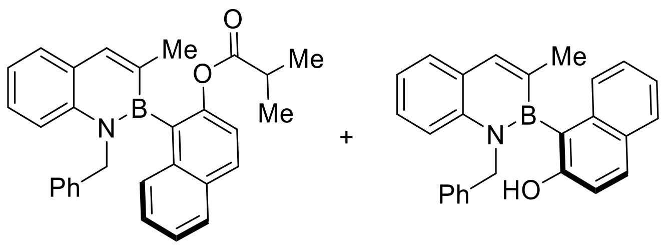

c = 52
s = 3

Following general procedure **E**, 1-(1-benzyl-3-methyl-1,2,4a,5-tetrahydroquinolin-2-yl)naphthalen-2-ol **15** (37.5 mg, 0.10 mmol), isobutyric anhydride **22** (8.3 μL, 0.05 mmol), F-(*R*)-BTM **19** (0.25 mg, 1.00 µmol), Et_3N (7.0 μL, 0.05 mmol) in $CHCl_3$ (1.0 mL) at RT for 16 h gave, after purification by Biotage® Selekt™ (Sfär HC 25 g, 80 mL.min^{-1}, Hexane :

EtOAc [100:0 to 90:10 10 CV, 90:10 to 75:25 10CV]), the title ester **17** (9.0 mg, 40%) as a white amorphous solid. $[\alpha]_D^{20}$ –24.9 (*c* 0.5, $CHCl_3$); **Chiral HPLC analysis**, Chiralcel OD-H (99:1 hexane : IPA, flow rate 1.0 mL.min^{-1}, 211 nm, 30 °C) t_R (*R*): 5.9 min, t_R (*S*): 6.7 min, 69:31 er; **IR** ν_{max} (film) 2972, 1749, 1611, 1454, 1132; **^{1}H NMR** (400 MHz, $CDCl_3$) δ_H: 1.02 (6H, dd, *J* 7.0, 2.8), 1.99 (3H, d, *J* 1.3), 2.55 (1H, app p, *J* 7.0), 5.16 (1H, d, *J* 16.6), 5.26 (1H, d, *J* 16.7), 7.01–7.03 (2H, m), 7.08–7.17 (3H, m), 7.23 (1H, ddd, *J* 8.0, 6.9, 1.3), 7.26–7.38 (4H, m), 7.41 (1H, ddd, *J* 8.1, 6.8, 1.2), 7.51 (1H, dd, *J* 8.2, 1.2), 7.71 (1H, dd, *J* 7.7, 1.3), 7.85–7.90 (3H, m); **^{13}C{^{1}H} NMR** (101 MHz, $CDCl_3$) δ_C: 18.8, 18.9, 21.7, 34.3, 53.2, 117.0, 121.4, 121.7, 125.4, 126.2, 126.7, 127.4, 127.6, 128.1, 128.4, 128.5, 129.3, 129.6, 131.5, 135.8, 138.7, 140.2, 142.1, 149.9, 175.7; **^{11}B{^{1}H} NMR** (128 MHz, $CDCl_3$) δ_B: 40.85 (br s); **HRMS** (ESI)$^+$ $C_{30}H_{28}BNO_2Na$ [M+Na]$^+$ found 468.2105, requires 468.2105 (–0.15 ppm).

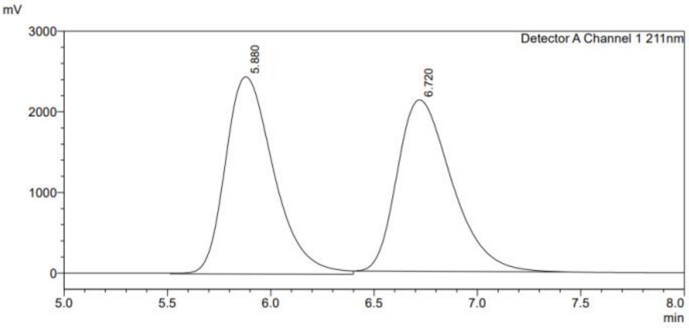

<Peak Table>

Detector A Channel 1 211nm

Peak#	Ret. Time	Area%
1	5.880	50.544
2	6.720	49.456
Total		100.000

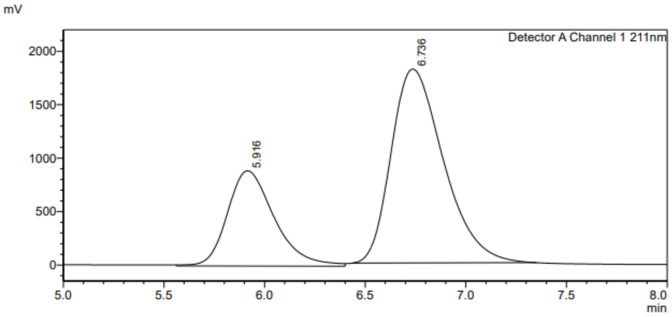

<Peak Table>

Detector A Channel 1 211nm

Peak#	Ret. Time	Area%
1	5.916	30.636
2	6.736	69.364
Total		100.000

The title alcohol (*R*)-1-(1-benzyl-3-methyl-1,2,4a,5-tetrahydroquinolin-2-yl)naphthalen-2-ol **15** (21.8 mg, 58%): $[\alpha]_D^{20}$ +67.5 (*c* 0.5, $CHCl_3$); **Chiral HPLC analysis**, Chiralpak IA (95:5 hexane : IPA, flow rate 1.0 mL.min^{-1}, 254 nm, 30 °C) t_R (*R*): 20.5 min, t_R (*S*): 24.3 min, 71:29 er.

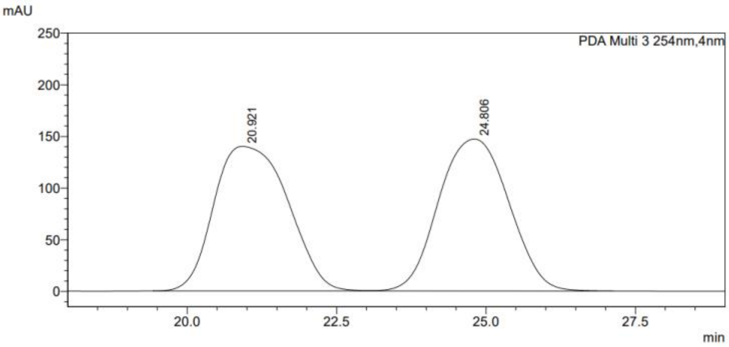

<Peak Table>

PDA Ch3 254nm

Peak#	Ret. Time	Area%
1	20.921	50.128
2	24.806	49.872
Total		100.000

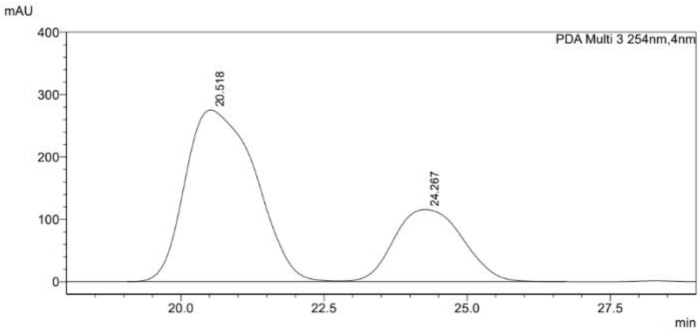

<Peak Table>

PDA Ch3 254nm

Peak#	Ret. Time	Area%
1	20.518	70.845
2	24.267	29.155
Total		100.000

(*S*)-1-(1-benzyl-3-methylbenzo[*e*][1,2]azaborinin-2(1*H*)-yl)naphthalen-2-yl propionate **18**

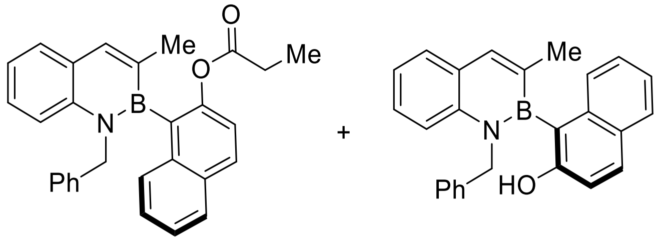

c = 45
s = 3

Following general procedure **E**, 1-(1-benzyl-3-methyl-1,2,4a,5-tetrahydroquinolin-2-yl)naphthalen-2-ol **15** (113 mg, 0.30 mmol), propionic anhydride **23** (26.8 μL, 0.21 mmol), (*R*)-BTM **14** (3.8 mg, 0.015 mmol), Et_3N (25 µL, 0.18 mmol) in $CHCl_3$ (0.5 mL) for 16 h gave, after purification by Biotage® Selekt™ (Sfär HC 25 g, 80 mL.min^{-1}, Hexane : EtOAc [100:0 to 90:10 10 CV, 90:10 to 75:25 10 CV]) the title ester **18** (42.9 mg, 33%) as a white amorphous solid. $[\alpha]_D^{20}$ –41.9 (*c* 0.8, $CHCl_3$); **Chiral HPLC analysis**, Chiralpak ID (95:5 hexane : IPA, flow rate 1.0 mL.min^{-1}, 254 nm, 30 °C) t_R (*R*): 5.8 min, t_R (*S*): 7.7 min, 30:70 er; **IR** ν_{max} (film) 2940, 1751, 1611, 1454, 1348, 1140, 906; **^{1}H NMR** (400 MHz, $CDCl_3$) δ_H: 1.02 (3H, app td, *J* 7.6, 1.7), 2.01 (3H, s), 2.33 (2H, q, *J* 7.6), 5.20 (1H, d, *J* 16.7), 5.27 (1H, d, *J* 16.7), 7.02 (2H, d, *J* 7.6), 7.12–7.18 (3H, m), 7.23–7.26 (1H, app m), 7.29–7.34 (3H, m), 7.37 (1H, t, *J* 8.3), 7.41–7.45 (1H, m), 7.51 (1H, d, *J* 8.3), 7.73 (1H, d, *J* 7.7), 7.87 (2H, dd, *J* 8.5, 4.2), 7.93 (1H, s); **^{13}C{^{1}H} NMR** (126 MHz, $CDCl_3$) δ_C: 9.2, 21.7, 27.9, 53.2, 117.1, 121.4, 121.7, 125.4, 126.1, 126.2, 126.7, 127.4, 127.6, 128.0, 128.4, 128.5, 129.3, 129.6, 131.5, 135.7, 138.7, 140.2, 142.1, 149.9, 173.1; **^{11}B{^{1}H} NMR** (128 MHz, $CDCl_3$) δ_B: 40.38 (br s); **HRMS** (ESI)$^+$ $C_{29}H_{26}BNO_2Na$ [M+Na]$^+$ found 454.1947, requires 454.1949 (–0.42 ppm).

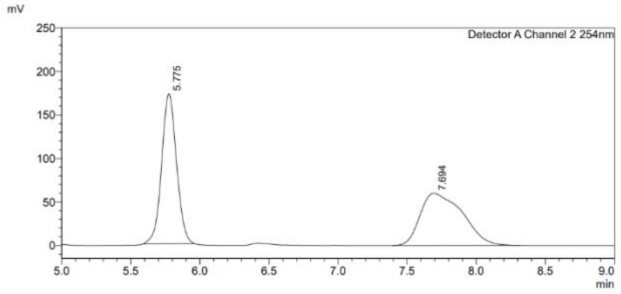

<Peak Table>

Detector A Channel 2 254nm

Peak#	Ret. Time	Area%
1	5.775	50.398
2	7.694	49.602
Total		100.000

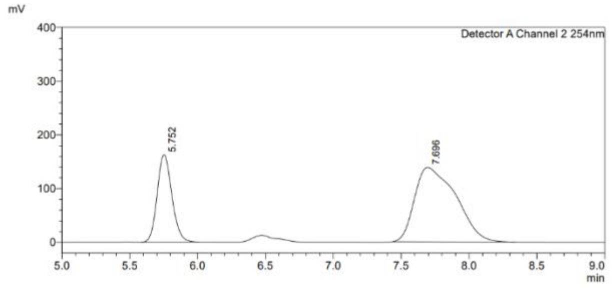

<Peak Table>

Detector A Channel 2 254nm

Peak#	Ret. Time	Area%
1	5.752	29.544
2	7.696	70.456
Total		100.000

The title alcohol (*R*)-1-(1-benzyl-3-methyl-1,2,4a,5-tetrahydroquinolin-2-yl)naphthalen-2-ol **15** (58.3 mg, 52%): $[\alpha]_D^{20}$ +77.8 (*c* 1.1, $CHCl_3$); **Chiral HPLC analysis**, Chiralpak IA (95:5 hexane : IPA, flow rate 1.0 mL.min^{-1}, 220 nm, 30 °C) t_R (*R*): 20.8 min, t_R (*S*): 24.4 min, 67:33 er.

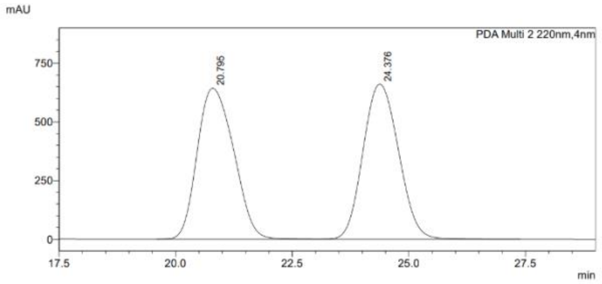

<Peak Table>

PDA Ch2 220nm

Peak#	Ret. Time	Area%
1	20.795	49.950
2	24.376	50.050
Total		100.000

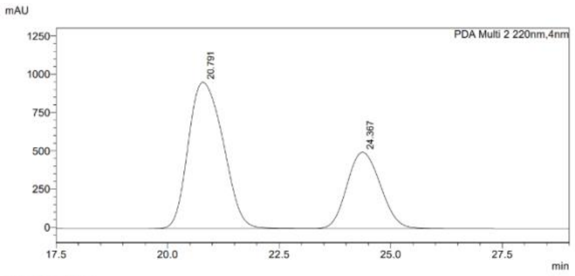

<Peak Table>

PDA Ch2 220nm

Peak#	Ret. Time	Area%
1	20.791	66.567
2	24.367	33.433
Total		100.000

(*S*)-1-(1-benzyl-3-ethylbenzo[*e*][1,2]azaborinin-2(1*H*)-yl)naphthalen-2-yl 2,2 diphenylacetate **27**

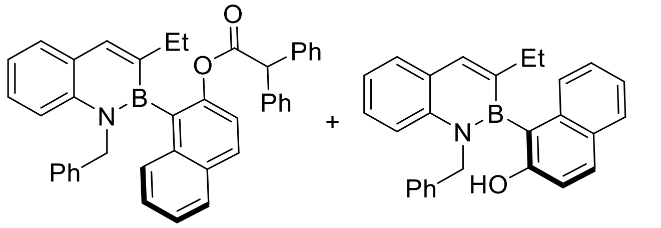

c = 43
s = 31

Following general procedure **E**, 1-(1-benzyl-3-ethylbenzo[*e*][1,2]azaborinin-2(1*H*)-yl)naphthalen-2-ol **26** (117 mg, 0.30 mmol), 2,2-diphenylacetic pivalic anhydride **13** (62.2 g, 0.21 mmol), (*R*)-BTM **14** (3.8 mg, 0.015 mmol), Et_3N (25.2 μL, 0.18 mmol) in $CHCl_3$ (3 mL) for 15 h gave, after purification with boron capped silica by Biotage® Selekt™ (Sfär HC 25 g, 80 mL.min^{-1}, Petrol : EtOAc [100:0 to 90:10 10 CV, 90:10 to 75:25 10 CV]), the title ester **27** (69.1 mg, 39%) as a white amorphous solid. $[\alpha]_D^{20}$ +34.3 (*c* 1.3, $CHCl_3$); **Chiral HPLC analysis**, Chiralpak IA (97:3 hexane : IPA, flow rate 1.0 mL.min^{-1}, 211 nm, 30 °C) t_R (*R*): 10.8 min, t_R(*S*): 16.0 min, 6:94 er; **IR** ν_{max} (film) 2960, 1751, 1610, 1494, 1192, 1130, 1115, 910; **^{1}H NMR** (400 MHz, $CDCl_3$) δ_H: 0.95 (3H, t, *J* 7.4), 2.28 (2H, q, *J* 7.4), 4.91 (1H, d, *J* 16.8), 5.03 (1H, s), 5.16 (1H, d, *J* 16.8), 6.86–6.89 (2H, m), 7.03–7.18 (13H, m), 7.22–7.40 (6H, m), 7.50 (1H, d, *J* 8.3), 7.78 (1H, dd, *J* 7.5, 1.9), 7.83 (2H, d, *J* 8.7), 7.86 (1H, s); **^{13}C{^{1}H} NMR** (101 MHz, $CDCl_3$) δ_C: 14.4, 27.7, 53.1, 57.4, 117.3, 121.3, 121.4, 125.5, 126.0, 126.1, 126.5, 127.3, 127.3, 127.4, 127.8, 128.3, 128.4, 128.6, 128.6, 128.6, 129.4, 129.8, 131.5, 136.0, 137.9, 138.1, 138.7, 139.8, 140.4, 149.8, 171.3; **^{11}B{^{1}H} NMR** (128 MHz, $CDCl_3$) δ_B: 40.48 (br s); **HRMS** (ESI$^+$) $C_{41}H_{35}BNO_2$ [M+H]$^+$ found 584.2751, requires 584.2755 (–0.75 ppm).

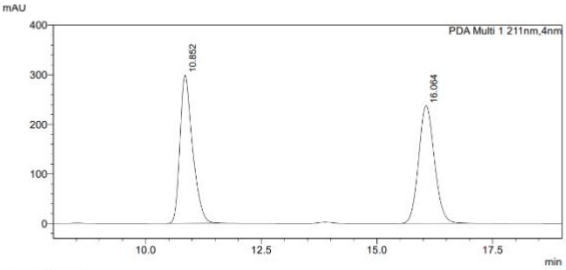

<Peak Table>
PDA Ch1 211nm

Peak#	Ret. Time	Name	Area%
1	10.852		50.080
2	16.064		49.920
Total			100.000

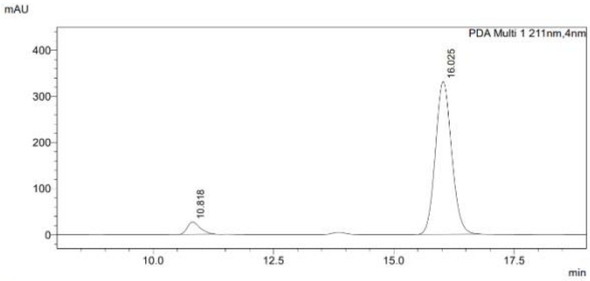

<Peak Table>
PDA Ch1 211nm

Peak#	Ret. Time	Area%
1	10.818	5.959
2	16.025	94.041
Total		100.000

The title alcohol (*R*)-1-(1-benzyl-3-ethylbenzo[e][1,2]azaborinin-2(1H)-yl)naphthalen-2-ol **26**: (54.6 mg, 47%): $[\alpha]_{\mathrm{D}}^{20}$ +165.8 (*c* 1.1, $CHCl_3$); **Chiral HPLC analysis**, Chiralpak AD-H (97:3 hexane : IPA, flow rate 1.0 mL.min^{-1}, 211 nm, 30 °C) t_R (*S*): 36.0 min, t_R (*R*): 45.7 min, 17:83 er.

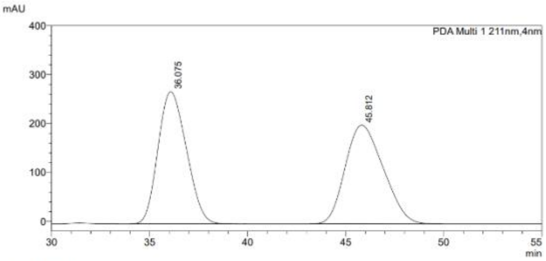

<Peak Table>
PDA Ch1 211nm

Peak#	Ret. Time	Area%
1	36.075	49.975
2	45.812	50.025
Total		100.000

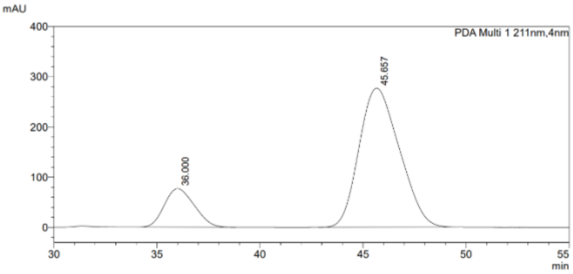

<Peak Table>
PDA Ch1 211nm

Peak#	Ret. Time	Area%
1	36.000	17.071
2	45.657	82.929
Total		100.000

(*S*)-1-(1-benzyl-4-methylbenzo[e][1,2]azaborinin-2(1H)-yl)naphthalen-2-yl
2,2 diphenylacetate **29**

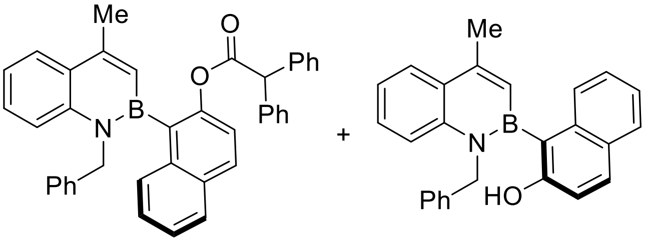

c = 49
s = 90

Following general procedure **E**, 1-(1-benzyl-4-methylbenzo[e][1,2]azaborinin-2(1H)-yl)naphthalen-2-ol **28** (75.1 mg, 0.20 mmol), 2,2-diphenylacetic anhydride **21** (56.9 mg, 0.14 mmol), (*R*)-BTM **14** (2.5 mg, 0.010 mmol), Et_3N (16.7 µL, 0.12 mmol) in $CHCl_3$ (2 mL) for 16 h gave, after purification by Biotage® Selekt™ (Sfär HC 25 g, 80 mL.min^{-1}, Petrol : EtOAc [100:0 to 90:10 12 CV, 90:10 to 75:25 10 CV]), the title ester **29** (52.5 mg, 46%) as a white amorphous solid. $[\alpha]_{D}^{20}$ +11.8 (*c* 0.2, $CHCl_3$); **Chiral HPLC analysis**, Chiralpak IA (99:1 hexane : IPA, flow rate 1.0 mL.min^{-1}, 220 nm, 30 °C) t_R (*R*): 21.8 min, t_R (*S*): 25.5 min, 3:97 er; **IR** ν_{max} (film) 3028, 1749, 1595, 1304, 1123, 907; **^{1}H NMR** (400 MHz, $CDCl_3$) δ_H:

2.67 (3H, s), 4.96 (1H, d, *J* 16.7), 5.10 (1H, s), 5.20 (1H, d, *J* 16.6), 6.85 (1H, s), 6.93–6.95 (2H, m), 7.07–7.22 (14H, m), 7.30–7.43 (5H, m), 7.65 (1H, d, *J* 8.3), 7.84 (2H, dd, *J* 9.1, 7.2), 8.01 (1H, dd, *J* 8.0, 1.6); **^{13}C{^{1}H} NMR** (126 MHz, $CDCl_3$) δ_C: 23.2, 53.0, 57.4, 117.9, 121.2, 121.3, 125.4, 126.0, 126.1, 126.4, 126.6, 127.3, 127.3, 127.8, 128.1, 128.4, 128.6, 128.6, 128.7, 128.8, 129.4, 131.7, 131.7 (br s), 136.1, 138.0, 138.2, 138.8, 141.5, 149.9, 151.2, 171.4; **^{11}B{^{1}H} NMR** (160 MHz, $CDCl_3$) δ_B: 37.57 (br s); **HRMS** (ESI)$^+$ $C_{40}H_{32}BNO_2Na$ $[M+Na]^+$ found 592.2423, requires 592.2419 (+0.75 ppm).

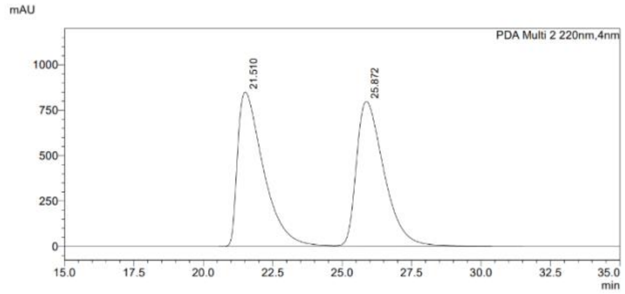

<Peak Table>

PDA Ch2 220nm

Peak#	Ret. Time	Area%
1	21.510	49.946
2	25.872	50.054
Total		100.000

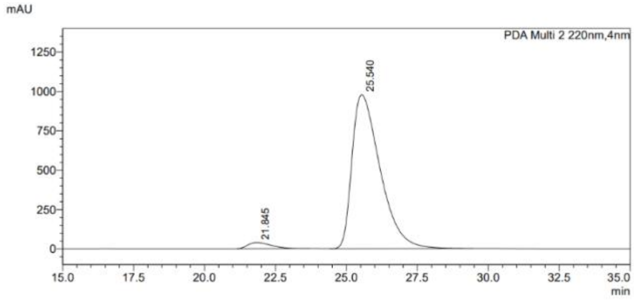

<Peak Table>

PDA Ch2 220nm

Peak#	Ret. Time	Area%
1	21.845	3.152
2	25.540	96.848
Total		100.000

The title alcohol (*R*)-1-(1-benzyl-4-methylbenzo[e][1,2]azaborinin-2(1H)-yl)naphthalen-2-ol **28** (36.0 mg, 48%): $[\alpha]_D^{20}$ +220.6 (*c* 1.7, $CHCl_3$); **Chiral HPLC analysis**, Chiralpak IA (95:5 hexane : IPA, flow rate 1.0 mL.min^{-1}, 254 nm, 30 °C) t_R (*R*):19.3 min, t_R (*S*): 31.8 min, 95:5 er.

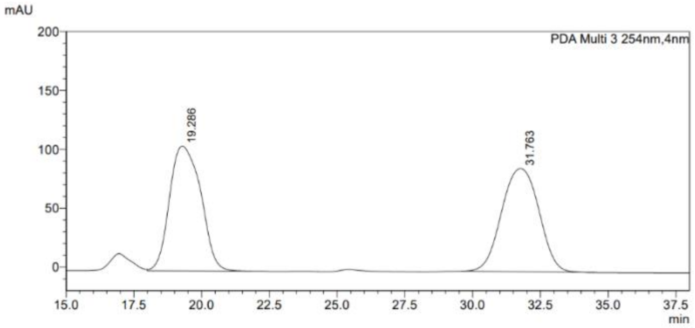

<Peak Table>

PDA Ch3 254nm

Peak#	Ret. Time	Area%
1	19.286	50.242
2	31.763	49.758
Total		100.000

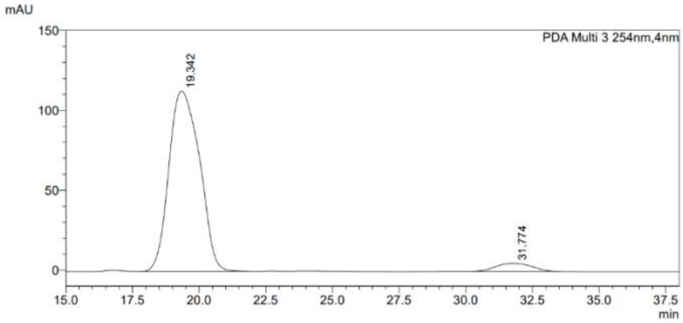

<Peak Table>

PDA Ch3 254nm

Peak#	Ret. Time	Area%
1	19.342	95.026
2	31.774	4.974
Total		100.000

(*S*)-1-(1-benzyl-3,4-dimethylbenzo[*e*][1,2]azaborinin-2(1*H*)-yl)naphthalen-2-yl 2,2 diphenylacetate **31**

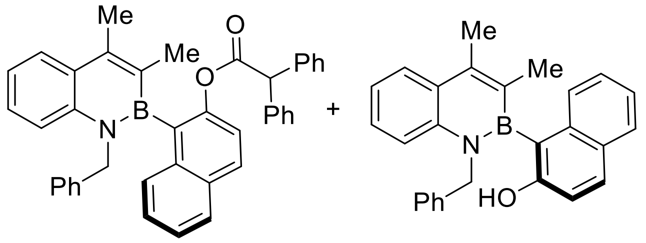

c = 46
s = 70

Following general procedure **E**, 1-(1-benzyl-3,4-dimethylbenzo[*e*][1,2]azaborinin-2(1*H*)-yl)naphthalen-2-ol **30** (117 mg, 0.30 mmol), 2,2-diphenylacetic pivalic anhydride **13** (62.2 mg, 0.21 mmol), (*R*)-BTM **14** (3.8 mg, 0.015 mmol), Et_3N (25.2 μL, 0.18 mmol) in $CHCl_3$ (3 mL) for 15 h gave, after purification on boron capped silica by Biotage® Selekt™ (Sfär HC 25 g, 80 mL.min^{-1}, Petrol : EtOAc [100:0 to 90:10 10 CV, 90:10 to 75:25 10 CV]), the title ester **31** (77.0 mg, 44%) as a white amorphous solid. $[\alpha]_D^{20}$ +19.5 (*c* 1.5, $CHCl_3$); **Chiral HPLC analysis**, Chiralpak IA (97:3 hexane : IPA, flow rate 1.0 mL.min^{-1}, 254 nm, 30 °C) t_R (*R*): 10.8 min, t_R (*S*): 16.0 min, 3:97 er; **IR** ν_{max} (film) 3028, 2924, 1751, 1587, 1192, 1119, 908; **^{1}H NMR** (400 MHz, $CDCl_3$) δ_H: 1.89 (3H, s), 2.59 (3H, s), 4.94 (1H, d, *J* 16.8), 5.04 (1H, s), 5.15 (1H, d, *J* 16.8), 6.89–6.92 (2H, m), 7.05–7.17 (13H, m), 7.24–7.35 (5H, m), 7.40 (1H, ddd, *J* 8.2, 6.8, 1.2), 7.52 (1H, dd, *J* 8.3, 1.1), 7.83 (1H, d, *J* 8.9), 7.85 (1H, d, *J* 8.3), 8.08–8.11 (1H, m); **^{13}C{^{1}H} NMR** (101 MHz, $CDCl_3$) δ_C: 15.8, 18.4, 53.3, 57.3, 117.6, 121.2, 121.3, 125.5, 125.8, 126.0, 126.1, 126.5, 126.9, 127.3, 128.2, 128.3, 128.4, 128.5, 128.5, 128.6, 129.2, 131.6, 135.9, 138.0, 138.1, 138.9, 140.2, 146.1, 149.7, 171.3; **^{11}B{^{1}H} NMR** (128 MHz, $CDCl_3$) δ_B: 40.50 (br s); **HRMS** (ESI$^+$) $C_{41}H_{35}BNO_2$ [M+H]$^+$ found 584.2745, requires 584.2755 (–1.78 ppm).

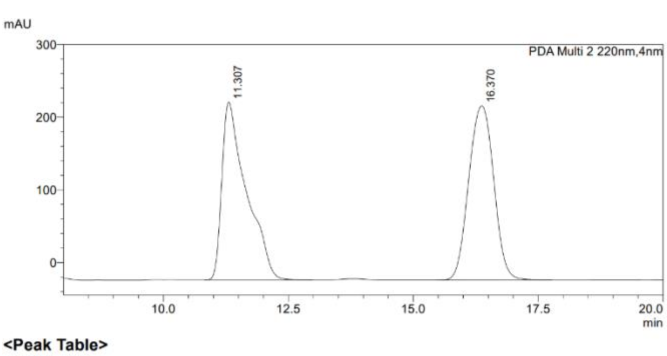

<Peak Table>

PDA Ch2 220nm

Peak#	Ret. Time	Area	Area%
1	11.307	8364742	49.989
2	16.370	8368329	50.011
Total		16733071	100.000

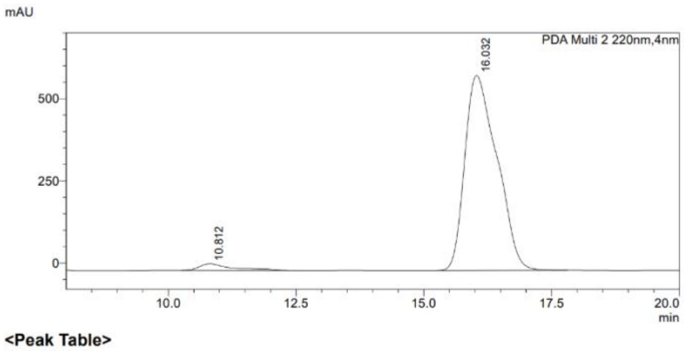

<Peak Table>

PDA Ch2 220nm

Peak#	Ret. Time	Area%
1	10.812	3.408
2	16.032	96.592
Total		100.000

The title alcohol (*R*)-1-(1-benzyl-3,4-dimethylbenzo[e][1,2]azaborinin-2(1H)-yl)naphthalen-2-ol **30** (63.1 mg, 54%): $[\alpha]_D^{20}$ +174.7 (*c* 1.4, $CHCl_3$); **Chiral HPLC analysis**,

Chiralpak IA (95:5 hexane : IPA, flow rate 1.0 mL.min^{-1}, 211 nm, 30 °C) t_R (*R*): 12.6 min, t_R (*S*): 24.5 min, 89:11 er.

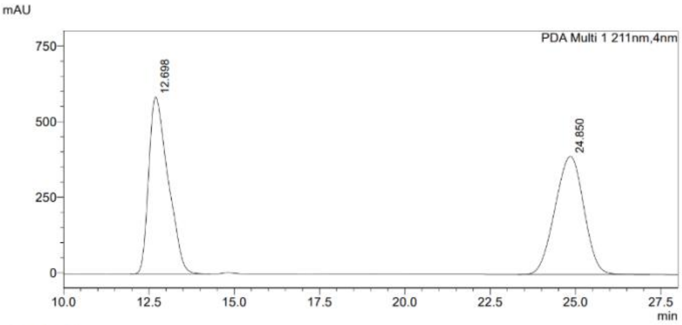

<Peak Table>

PDA Ch1 211nm

Peak#	Ret. Time	Area%
1	12.698	50.130
2	24.850	49.870
Total		100.000

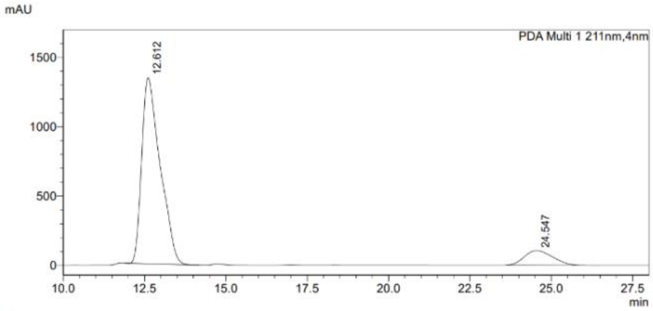

<Peak Table>

PDA Ch1 211nm

Peak#	Ret. Time	Area%
1	12.612	89.259
2	24.547	10.741
Total		100.000

(*S*)-1-(1-benzyl-3,6-dimethylbenzo[e][1,2]azaborinin-2(1H)-yl)naphthalen-2-yl 2,2 diphenylacetate **33**

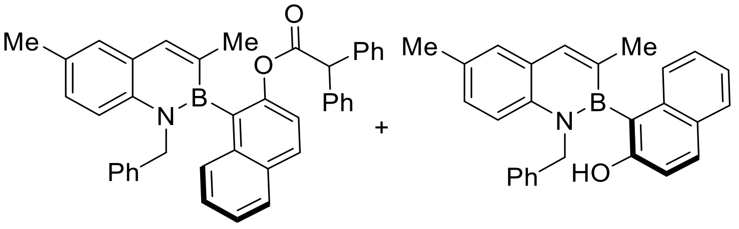

c = 43
s = 60

Following general procedure **E**, 1-(1-benzyl-3,6-dimethylbenzo[e][1,2]azaborinin-2(1H)-yl)naphthalen-2-ol **32** (117 mg, 0.30 mmol), 2,2-diphenylacetic pivalic anhydride **13** (62.2 mg, 0.21 mmol), (*R*)-BTM **14** (1.8 mg, 0.015 mmol), Et_3N (25.2 μL, 0.21 mmol) in $CHCl_3$ (3 mL) for 16 h gave, after purification by Biotage® Selekt™ (Sfär HC 25 g, 160 mL.min^{-1}, Petrol : EtOAc, [100:0 to 90:10 8 CV, 90:10 to 75:25 10 CV]), the title ester **33** (54.4 mg, 31%) as a white amorphous solid. $[a]_D^{20}$ +40.1 (*c* 1.0, $CHCl_3$); **Chiral HPLC analysis**, Chiralpak IA (99:1 hexane : IPA, flow rate 1.0 mL.min^{-1}, 270 nm, 30 °C) t_R (*R*): 20.4 min, t_R (*S*): 25.3 min, 3:97 er; **IR** ν_{max} (film) 3028, 1749, 1494, 1192, 1117, 908; **^{1}H NMR** (400 MHz, $CDCl_3$) δ_H: 1.96 (3H, d, *J* 1.3), 2.51 (3H, s), 4.90 (1H, d, *J* 16.7), 5.06 (1H, s), 5.16 (1H, d, *J* 16.7), 6.89–6.92 (2H, m), 7.06–7.20 (15H, m), 7.26 (1H, t, *J* 4.4), 7.28–7.31 (1H, m), 7.41 (1H, ddd, *J* 8.1, 6.8, 1.2), 7.52–7.55 (2H, m), 7.82–7.86 (3H, m); **^{13}C{^{1}H} NMR** (126 MHz, $CDCl_3$) δ_C: 20.9, 21.8, 53.1, 57.3, 117.2, 121.3, 125.5, 126.1, 126.5, 127.3, 127.3, 127.7, 128.3, 128.3, 128.4, 128.6, 128.6, 128.6, 129.3, 129.4, 130.6, 131.6, 135.8, 137.9, 138.2, 138.3, 138.8, 140.5 (br s), 142.0, 149.7, 171.4; **^{11}B{^{1}H} NMR** (160 MHz, $CDCl_3$) δ_B: 38.37 (br s); **HRMS** (ESI)$^+$ $C_{41}H_{34}BNO_2Na$ [M+Na]$^+$ found 606.2574, requires 606.2575 (–0.14 ppm).

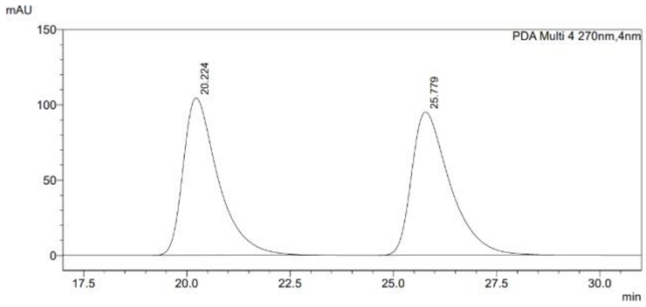

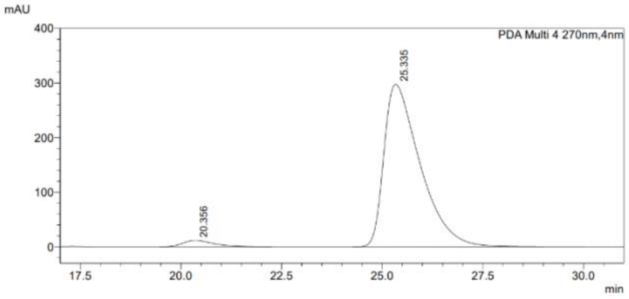

<Peak Table>

PDA Ch4 270nm

Peak#	Ret. Time	Area%
1	20.224	50.047
2	25.779	49.953
Total		100.000

<Peak Table>

PDA Ch4 270nm

Peak#	Ret. Time	Area%
1	20.356	3.285
2	25.335	96.715
Total		100.000

The title alcohol (*R*)-1-(1-benzyl-3,6-dimethylbenzo[e][1,2]azaborinin-2(1H)-yl)naphthalen-2-ol **32** (51.5 mg, 44%): $[\alpha]_D^{20}$ +192.3 (*c* 1.0, $CHCl_3$); **Chiral HPLC analysis**, Chiralpak IA (95:5 hexane : IPA, flow rate 1.0 mL.min^{-1}, 270 nm, 30 °C) t_R (*S*): 30.7 min, t_R (*R*): 42.3 min, 15:85 er.

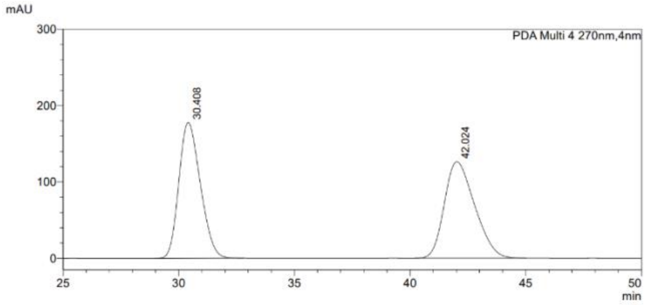

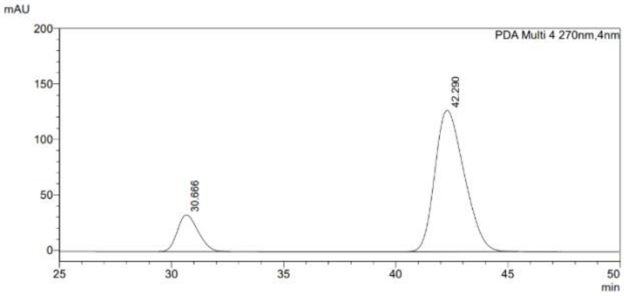

<Peak Table>

PDA Ch4 270nm

Peak#	Ret. Time	Area%
1	30.408	49.998
2	42.024	50.002
Total		100.000

<Peak Table>

PDA Ch4 270nm

Peak#	Ret. Time	Area%
1	30.666	15.441
2	42.290	84.559
Total		100.000

(*S*)-1-(1-benzyl-3,7-dimethylbenzo[e][1,2]azaborinin-2(1H)-yl)naphthalen-2-yl 2,2 diphenylacetate **35**

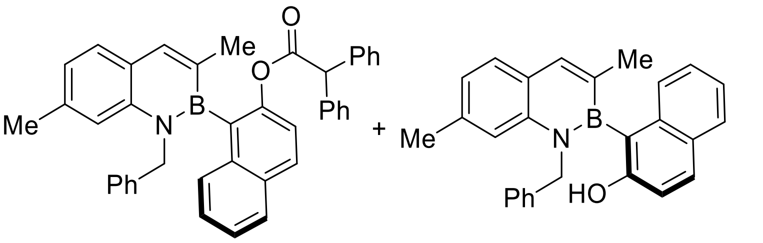

c = 49
s = 46

Following general procedure **E**, 1-(1-benzyl-3,7-dimethylbenzo[e][1,2]azaborinin-2(1H)-yl)naphthalen-2-ol **34** (117 mg, 0.30 mmol), 2,2-diphenylacetic pivalic anhydride **13** (62.2 mg, 0.21 mmol), (*R*)-BTM **14** (3.8 mg, 0.015 mmol), Et_3N (25.2 μL, 0.18 mmol) in $CHCl_3$ (3 mL) for 16 h gave, after purification on boron capped silica on the Biotage® Selekt™ (Sfär HC 25 g, 80 mL.min^{-1}, Petrol : EtOAc [100:0 to 95:5 3 CV, 95:5 to 85:15 12 CV,

85:15 to 75:25 5 CV]), the title ester **35** (79.0 mg, 45%) as a white amorphous solid. $[\alpha]_D^{20}$ +36.2 (*c* 1.4, $CHCl_3$); **Chiral HPLC analysis**, Chiralpak AD-H (99:1 hexane : IPA, flow rate 1.0 mL.min^{-1}, 254 nm, 30 °C) t_R (*R*): 9.8 min, t_R (*S*): 21.6 min, 6:94 er; **IR** ν_{max} (film) 3028, 2916, 1748, 1601, 1452, 1231, 1115, 907; **^{1}H NMR** (400 MHz, $CDCl_3$) δ_H: 1.93 (3H, d, *J* 1.3), 2.38 (3H, s), 4.87 (1H, d, *J* 16.7), 5.04 (1H, s), 5.14 (1H, d, *J* 16.7), 6.88–6.91 (2H, m), 7.05–7.18 (15H, m), 7.24–7.29 (2H, app m), 7.39 (1H, ddd, *J* 8.1, 6.8, 1.2), 7.50 (1H, dd, *J* 8.3, 1.1), 7.64 (1H, d, *J* 7.9), 7.82–7.85 (3H, m); **^{13}C{^{1}H} NMR** (126 MHz, $CDCl_3$) δ_C: 21.6, 22.2, 53.0, 57.3, 117.5, 121.3, 122.8, 125.4, 125.5, 126.1, 126.2, 126.5, 127.2, 127.3, 128.3, 128.4, 128.6, 128.6, 129.3, 129.4, 130.8 (br s), 131.6, 135.8, 137.2, 137.9, 138.2, 138.8, 139.2 (br s), 140.4, 142.1, 149.6, 171.4; **^{11}B{^{1}H}** (160 MHz, $CDCl_3$) δ_B: 38.37 (br s); **HRMS** (ESI)$^+$ $C_{41}H_{34}BNO_2Na$ [M+Na]$^+$ found 606.2577, requires 606.2575 (+0.43 ppm).

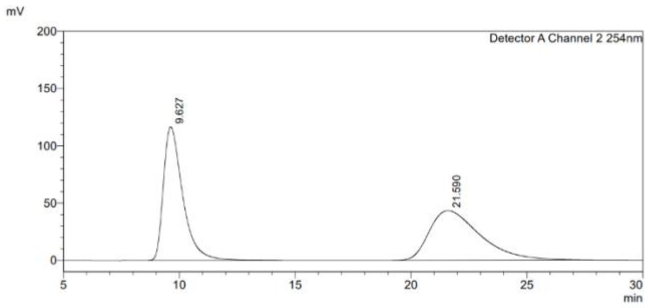

<Peak Table>

Detector A Channel 2 254nm

Peak#	Ret. Time	Area%
1	9.627	50.477
2	21.590	49.523
Total		100.000

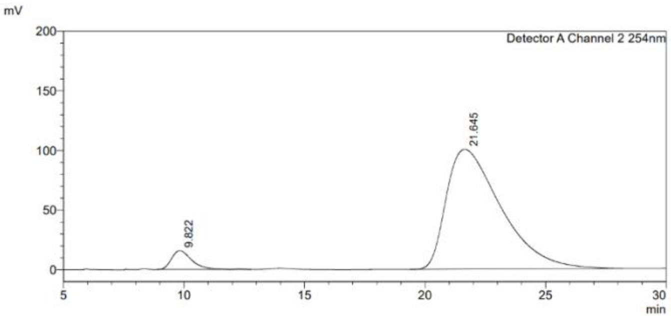

<Peak Table>

Detector A Channel 2 254nm

Peak#	Ret. Time	Area%
1	9.822	5.534
2	21.645	94.466
Total		100.000

The title alcohol (*R*)-1-(1-benzyl-3,7-dimethylbenzo[e][1,2]azaborinin-2(1H)-yl)naphthalen-2-ol **34** (57.9 mg, 50%): $[\alpha]_D^{20}$ +238.8 (*c* 1.1, $CHCl_3$); **Chiral HPLC analysis**, Chiralcel OD-H (99.3:0.7 hexane : IPA, flow rate 1.5 mL.min^{-1}, 211 nm, 30 °C) t_R (*R*): 36.7 min, t_R (*S*): 51.7 min, 92:8 er.

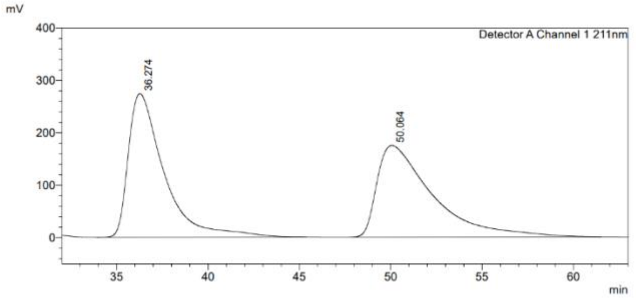

<Peak Table>

Detector A Channel 1 211nm

Peak#	Ret. Time	Area%
1	36.274	50.202
2	50.064	49.798
Total		100.000

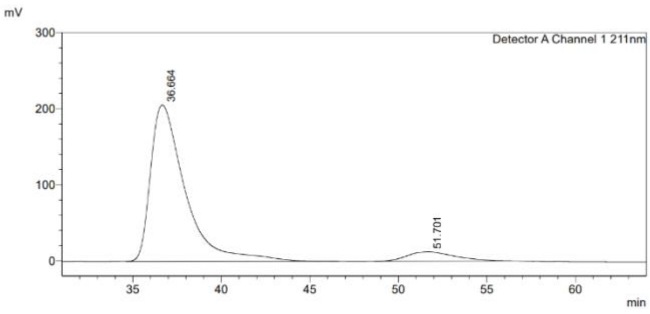

<Peak Table>

Detector A Channel 1 211nm

Peak#	Ret. Time	Area%
1	36.664	92.454
2	51.701	7.546
Total		100.000

(*S*)-1-(1,3-dimethylbenzo[*e*][1,2]azaborinin-2(1*H*)-yl)naphthalen-2-yl 2,2 diphenylacetate **37**

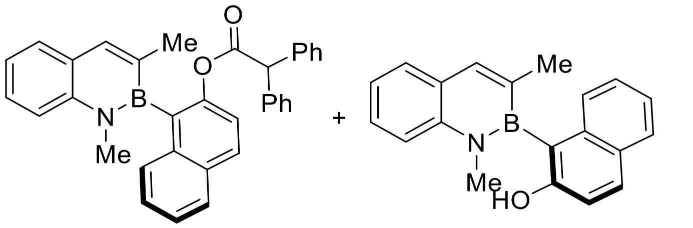

c = 60
s = 4

Following general procedure **E**, 1-(1,3-dimethylbenzo[*e*][1,2]azaborinin-2(1*H*)-yl)naphthalen-2-ol **36** (89.8 mg, 0.30 mmol), 2,2-diphenylacetic pivalic anhydride **13** (62.2 mg, 0.21 mmol), (*R*)-BTM **14** (3.8 mg, 0.015 mmol), Et_3N (25.2 μL, 0.18 mmol) in $CHCl_3$ (3 mL) for 16 h gave, after purification on boron capped silica by Biotage® Selekt™ (Sfär HC 25 g, 80 mL.min^{-1}, Cyclohexane : EtOAc [100:0 to 90:10 10 CV, 90:10 to 75:25 10 CV]), the title ester **37** (74.6 mg, 50%) as a white amorphous solid. $[\alpha]_D^{20}$ –9.6 (*c* 1.3, $CHCl_3$); **Chiral HPLC analysis**, Chiralpak IA (98:2 hexane : IPA, flow rate 1.0 mL.min^{-1}, 254 nm, 30 °C) t_R (*R*): 8.7 min, t_R (*S*): 11.0 min, 30:70 er; **IR** ν_{max} (film) 2936, 1749, 1611, 1494, 1339, 1123, 762; **^{1}H NMR** (400 MHz, $CDCl_3$) δ_H: 1.91 (3H, s), 3.29 (3H, s), 5.01 (1H, s), 6.89 (2H, t, *J* 7.5), 6.95 (2H, d, *J* 7.2), 6.99 (1H, t, *J* 7.2), 7.04–7.13 (5H, m), 7.29 (1H, d, *J* 8.8), 7.35 (2H, m), 7.41 (1H, d, *J* 8.2), 7.46 (2H, app t, *J* 7.7), 7.55 (1H, ddd, *J* 8.5, 7.0, 1.6), 7.72 (1H, dd, *J* 7.8, 1.6), 7.76 (1H, s), 7.90 (1H, d, *J* 8.8), 7.91 (1H, d, *J* 8.1); **^{13}C{^{1}H} NMR** (126 MHz, $CDCl_3$) δ_C: 21.7, 37.0, 57.2, 115.2, 121.3, 121.4, 125.5, 126.4, 127.1, 127.3, 127.4, 127.4, 128.2, 128.4, 128.4, 128.5, 128.6, 128.6, 129.4, 129.5, 131.7, 135.8, 137.9, 138.0, 141.2, 141.5, 149.8, 171.6; **^{11}B{^{1}H} NMR** (128 MHz, $CDCl_3$) δ_B: 38.66 (br s); **HRMS** (ESI$^+$) $C_{34}H_{29}BNO_2$ $[M+H]^+$ found 494.2282, requires 494.2286 (–0.79 ppm).

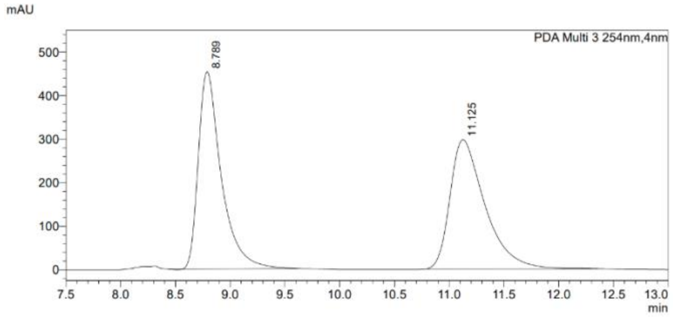

<Peak Table>

PDA Ch3 254nm

Peak#	Ret. Time	Area%
1	8.789	49.916
2	11.125	50.084
Total		100.000

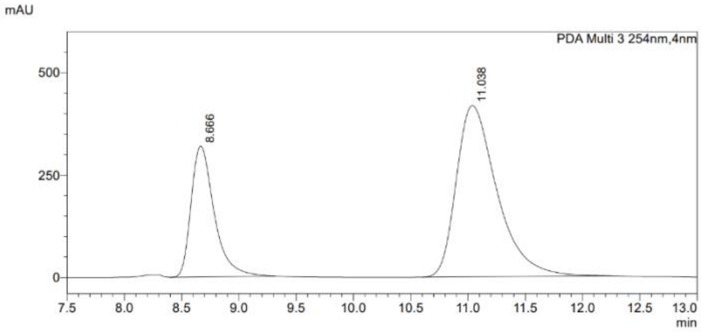

<Peak Table>

PDA Ch3 254nm

Peak#	Ret. Time	Area%
1	8.666	30.243
2	11.038	69.757
Total		100.000

The title alcohol (*R*)-1-(1,3-dimethylbenzo[e][1,2]azaborinin-2(1H)-yl)naphthalen-2-ol **36** (31.7 mg, 35%): $[\alpha]_D^{20}$ +65.0 (*c* 0.6, $CHCl_3$); **Chiral HPLC analysis**, Chiralpak IA (97:3 hexane : IPA, flow rate 1.0 mL.min^{-1}, 254 nm, 30 °C) t_R (*R*): 25.5 min, t_R (*S*): 34.9 min, 80:20 er.

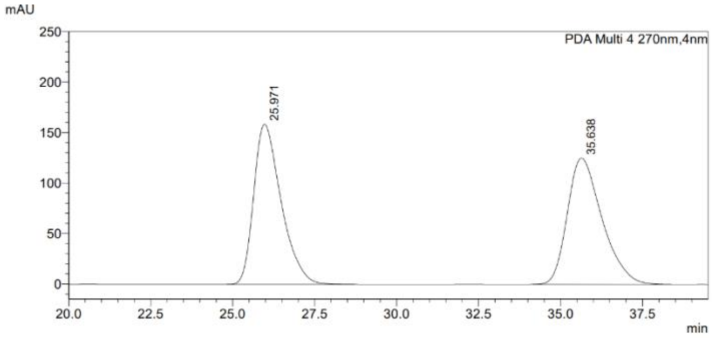

<Peak Table>

PDA Ch4 270nm

Peak#	Ret. Time	Area%
1	25.971	50.103
2	35.638	49.897
Total		100.000

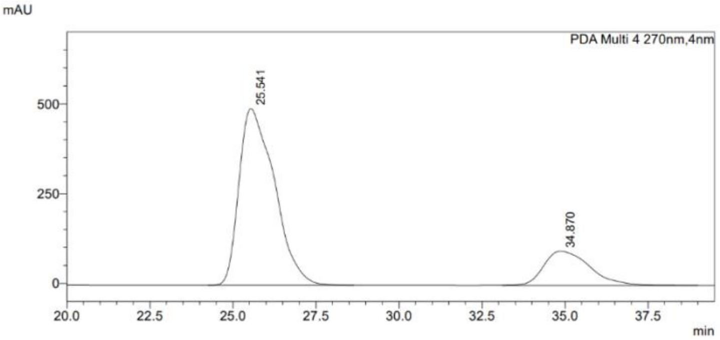

<Peak Table>

PDA Ch4 270nm

Peak#	Ret. Time	Area%
1	25.541	79.951
2	34.870	20.049
Total		100.000

(*S*)-1-(1-ethyl-3-methylbenzo[*e*][1,2]azaborinin-2(1*H*)-yl)naphthalen-2-yl 2,2 diphenylacetate **39**

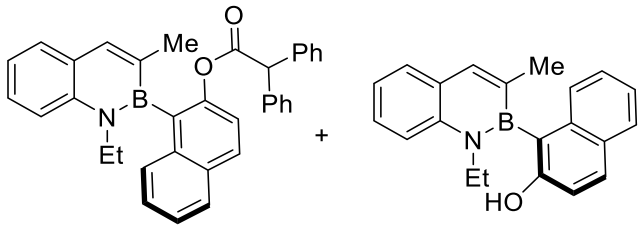

c = 59
s = 12

Following general procedure **E**, 1-(1-ethyl-3-methylbenzo[*e*][1,2]azaborinin-2(1*H*)-yl)naphthalen-2-ol **38** (94.0 mg, 0.30 mmol), 2,2-diphenylacetic pivalic anhydride **13** (62.6 mg, 0.21 mmol), (*R*)-BTM **14** (3.8 mg, 0.015 mmol), Et_3N (25.2 µL, 0.18 mmol) in $CHCl_3$ (3 mL) for 16 h gave, after purification on boron capped silica by Biotage® Selekt™ (Sfär HC 25 g, 120 mL.min^{-1}, Petrol : EtOAc [100:0 to 90:10 10 CV, 90:10 to 75:25 10 CV]), the title ester **39** (68.6 mg, 43%) as a white amorphous solid. $[\alpha]_D^{20}$ −12.6 (*c* 1.3, $CHCl_3$); **Chiral HPLC analysis**, Chiralpak IA (97:3 hexane : IPA, flow rate 1.0 mL.min^{-1}, 254 nm, 30 °C) t_R (*R*): 7.0 min, t_R (*S*): 7.6 min, 19:81 er; **IR** ν_{max} (film) 2974, 1749, 1192, 1125, 906; **^{1}H NMR** (400 MHz, $CDCl_3$) δ_H: 0.97 (3H, t, *J* 7.1), 1.86 (3H, s), 3.69 (1H, dq, *J* 14.2, 7.1), 3.88 (1H, dq, *J* 14.2, 7.1), 5.00 (1H, s), 6.91−6.95 (2H, m), 6.98−7.10 (8H, m), 7.28−7.34 (3H, m), 7.41−7.54 (4H, m), 7.73 (1H, dd, *J* 7.9, 1.6), 7.75 (1H, s), 7.88 (1H, d, *J* 8.8), 7.90 (1H, d, *J* 8.0); **^{13}C{^{1}H} NMR** (126 MHz, $CDCl_3$) δ_C: 14.9, 21.6, 43.8, 57.4, 115.6, 121.1, 121.4, 125.6, 126.2, 127.2, 127.2, 127.3, 127.8, 128.3, 128.4, 128.5, 128.6, 128.6, 129.2, 129.9, 131.7, 135.8, 137.8, 138.0, 139.9, 141.6, 149.5, 171.4; **^{11}B{^{1}H} NMR**

(128 MHz, $CDCl_3$) δ_B: 38.86 (br s), **HRMS** (ESI^+) $C_{35}H_{30}BNO_2Na$ $[M+Na]^+$ found 530.2264, requires 530.2262 (+0.37 ppm).

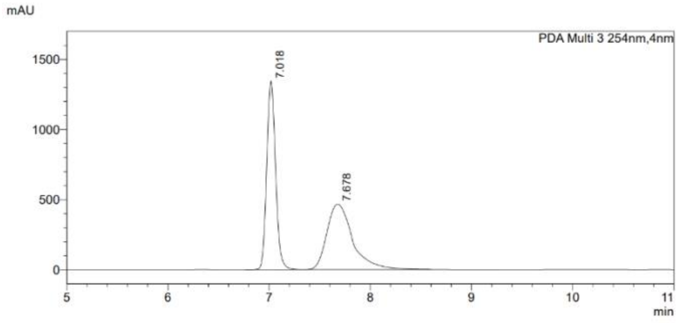

<Peak Table>

PDA Ch3 254nm

Peak#	Ret. Time	Area%
1	7.018	49.953
2	7.678	50.047
Total		100.000

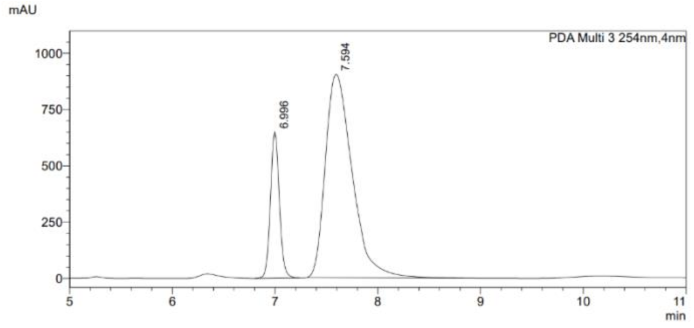

<Peak Table>

PDA Ch3 254nm

Peak#	Ret. Time	Area%
1	6.996	19.196
2	7.594	80.804
Total		100.000

The title alcohol (*R*)-1-(1-ethyl-3-methylbenzo[e][1,2]azaborinin-2(1H)-yl)naphthalen-2-ol **38** (38.7 mg, 41%): $[\alpha]_D^{20}$ +84.2 (*c* 1.7, $CHCl_3$); **Chiral HPLC analysis**, Chiralpak IA (95:5 hexane : IPA, flow rate 1.0 mL.min^{-1}, 254 nm, 30 °C) t_R (*R*): 11.3 min, t_R (*S*): 20.4 min, 95:5 er.

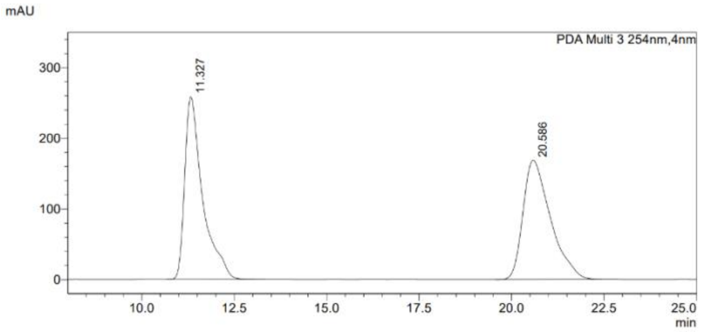

<Peak Table>

PDA Ch3 254nm

Peak#	Ret. Time	Area%
1	11.327	49.903
2	20.586	50.097
Total		100.000

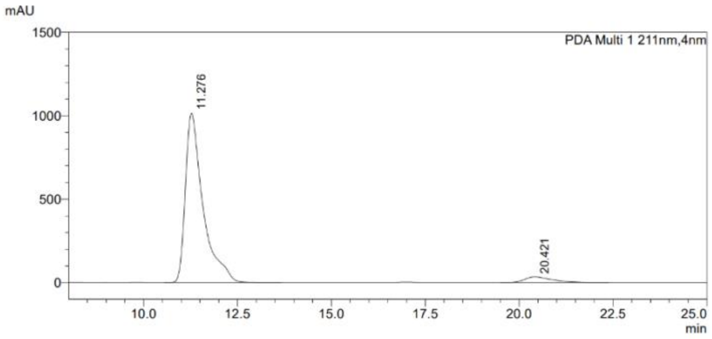

<Peak Table>

PDA Ch1 211nm

Peak#	Ret. Time	Area%
1	11.276	94.967
2	20.421	5.033
Total		100.000

(*S*)-1-(1-allyl-3-methylbenzo[e][1,2]azaborinin-2(1H)-yl)naphthalen-2-yl
2,2 diphenylacetate **41**

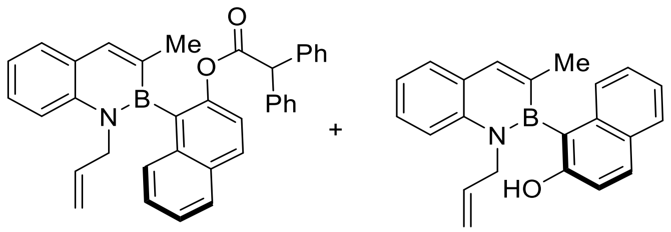

c = 57
s = 17

Following general procedure **E**, 1-(1-allyl-3-methylbenzo[e][1,2]azaborinin-2(1H)-yl)naphthalen-2-ol **40** (97.6 mg, 0.30 mmol), 2,2-diphenylacetic pivalic anhydride **13**

(62.2 mg, 0.21 mmol), (*R*)-BTM **14** (3.8 mg, 0.015 mmol), Et_3N (25.2 μL, 0.18 mmol) in $CHCl_3$ (3 mL) for 16 h gave, after purification on boron capped silica by Biotage® Selekt™ (Sfär HC 25 g, 80 mL.min^{-1}, Petrol : EtOAc [100:0 to 90:10 10 CV, 90:10 to 75:25 10 CV]), the title ester **41** (71.7 mg, 46%) as a white amorphous solid. $[\alpha]_D^{20}$ –5.2 (*c* 1.4, $CHCl_3$); **Chiral HPLC analysis**, Chiralpak IA (97:3 hexane : IPA, flow rate 1.0 mL.min^{-1}, 254 nm, 30 °C) t_R (*R*): 7.0 min, t_R (*S*): 8.4 min, 16:84 er; **IR** ν_{max} (film) 3059, 1749, 1611, 1494, 1227, 1192, 1136; **^{1}H NMR** (400 MHz, $CDCl_3$) δ_H: 1.90 (3H, d, *J* 1.3), 4.28 (1H, ddt, *J* 16.8, 5.2, 1.8), 4.50 (1H, ddt, *J* 16.8, 5.2, 1.9), 4.79 (1H, dd, *J* 17.3, 1.6), 4.87 (1H, dd, *J* 10.5, 1.6), 5.01 (1H, s), 5.55 (1H, ddt, *J* 17.3, 10.4, 5.1), 6.95–6.99 (2H, m), 7.03–7.13 (8H, m), 7.29 (1H, d, *J* 8.9), 7.33 (2H, ddd, *J* 7.8, 2.6, 2.2), 7.43–7.51 (4H, m), 7.73 (1H, dd, *J* 7.7, 1.4), 7.79 (1H, s), 7.88 (1H, d, *J* 8.9), 7.90 (1H, d, *J* 8.2); **^{13}C{^{1}H} NMR** (101 MHz, $CDCl_3$) δ_C: 21.3, 51.9, 57.3, 116.3, 116.6, 121.3, 121.4, 125.6, 126.2, 127.2, 127.3, 127.6, 128.4, 128.5, 128.5, 128.5, 128.6, 128.6, 129.4, 129.6, 131.7, 135.2, 135.8, 137.8, 138.0, 140.3, 141.9, 149.5, 171.4; **^{11}B{^{1}H} NMR** (128 MHz, $CDCl_3$) δ_B: 38.63 (br s); **HRMS** (ESI)$^+$ $C_{36}H_{30}BNO_2Na$ [M+Na]$^+$ found 542.2265, requires 542.2262 (+0.64 ppm).

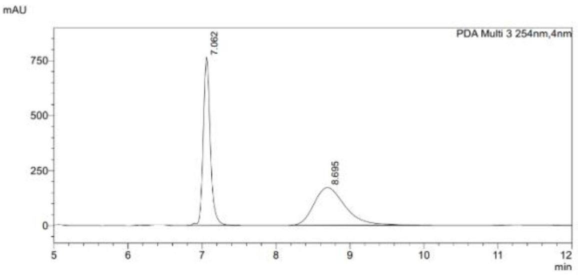

<Peak Table>

PDA Ch3 254nm

Peak#	Ret. Time	Area%
1	7.062	50.095
2	8.695	49.905
Total		100.000

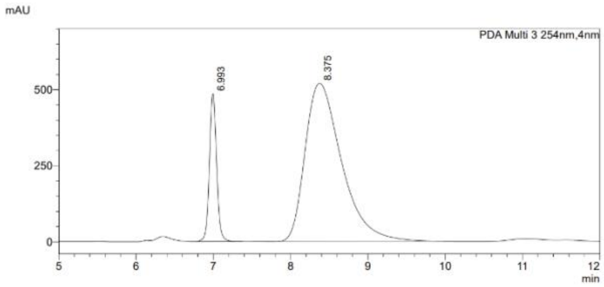

<Peak Table>

PDA Ch3 254nm

Peak#	Ret. Time	Area%
1	6.993	15.630
2	8.375	84.370
Total		100.000

The title alcohol (*R*)-1-(1-allyl-3-methylbenzo[e][1,2]azaborinin-2(1H)-yl)naphthalen-2-ol **40** (34.8 mg, 36%): $[\alpha]_D^{20}$ +97.0 (*c* 1.6, $CHCl_3$); **Chiral HPLC analysis**, Chiralpak IA (95:5 hexane : IPA, flow rate 1.0 mL.min^{-1}, 254 nm, 30 °C) t_R (*R*): 12.4 min, t_R (*S*): 22.0 min, 96:4 er.

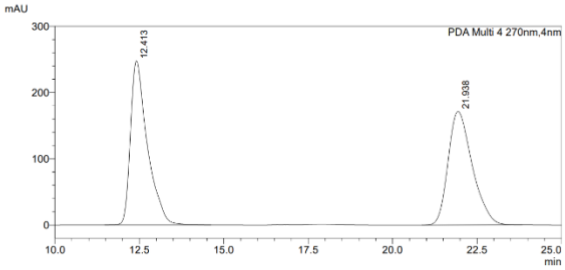

<Peak Table>

PDA Ch4 270nm

Peak#	Ret. Time	Area%
1	12.413	50.368
2	21.938	49.632
Total		100.000

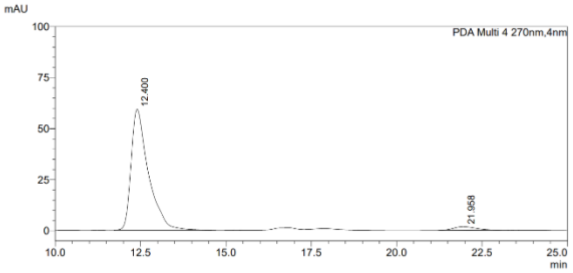

PDA Ch4 270nm

Peak#	Ret. Time	Area%
1	12.400	96.262
2	21.958	3.738
Total		100.000

(*S*)-1-(1-(furan-2-ylmethyl)-3-methylbenzo[e][1,2]azaborinin-2(1H)-yl)naphthalen-2-yl 2,2-diphenylacetate **43**

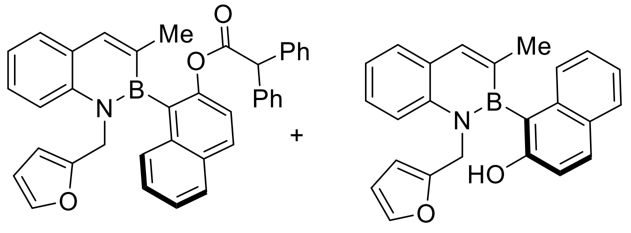

c = 50
s = 14

Following general procedure **E**, 1-(1-(furan-2-ylmethyl)-3-methylbenzo[e][1,2]azaborinin-2(1H)-yl)naphthalen-2-ol **42** (110 mg, 0.30 mmol), 2,2-diphenylacetic pivalic anhydride **13** (62.2 mg, 0.21 mmol), (*R*)-BTM **14** (3.8 mg, 0.015 mmol), Et_3N (25.2 µL, 0.18 mmol) in $CHCl_3$ (3 mL) for 16 h gave, after purification on Biotage® Selekt™ (Sfär HC 25 g, 80 mL.min^{-1}, Petrol : EtOAc [100:0 to 95:5 3 CV, 95:5 to 85:15 15 CV]), the title ester **43** (72.4 mg, 43%) as a white amorphous solid. $[\alpha]_D^{20}$ +12.6 (*c* 1.3, $CHCl_3$); **Chiral HPLC analysis**, Chiralpak ID (99:1 hexane : IPA, flow rate 1.0 mL.min^{-1}, 254 nm, 30 °C) t_R (*R*): 14.1 min, t_R (*S*): 22.4 min, 13:87 er; **IR** ν_{max} (film) 3059, 1748, 1611, 1337, 1192, 1145, 907; **^{1}H NMR** (400 MHz, $CDCl_3$) δ_H: 1.88 (3H, d, *J* 1.3), 4.78 (1H, d, *J* 16.5), 5.00 (1H, s), 5.05 (1H, d, *J* 16.2), 5.61 (1H, d, *J* 2.8), 6.02 (1H, dd, *J* 3.3, 1.8), 6.98–7.12 (11H, m), 7.27–7.33 (3H, m), 7.41–7.48 (3H, m), 7.55 (1H, d, *J* 8.5), 7.71 (1H, dd, *J* 7.8, 1.6), 7.78 (1H, s), 7.88 (2H, dd, *J* 8.6, 1.9); **^{13}C{^{1}H} NMR** (126 MHz, $CDCl_3$) δ_C: 21.6, 46.4, 57.3, 107.4, 110.3, 116.4, 121.2, 121.6, 125.6, 126.1, 127.2, 127.3, 127.3, 127.6, 128.4, 128.5, 128.5, 128.6, 129.6, 129.6, 130.7 (br s), 131.6, 135.8, 137.9, 138.0, 140.3, 140.4 (br s), 141.1, 142.2, 149.6, 152.1, 171.3; **^{11}B{^{1}H} NMR** (128 MHz, $CDCl_3$) δ_B: 40.25 (br s); **HRMS** (ESI)$^+$ $C_{38}H_{30}BNO_3Na$ $[M+Na]^+$ found 582.2207, requires 582.2211 (–0.66 ppm).

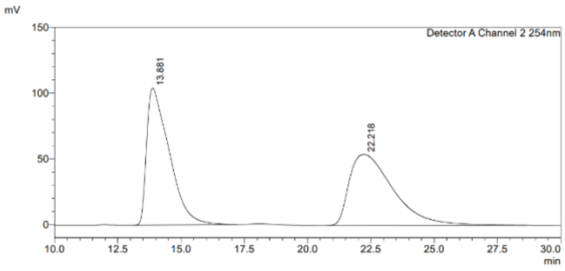

<Peak Table>

Detector A Channel 2 254nm

Peak#	Ret. Time	Area%
1	13.881	49.829
2	22.218	50.171
Total		100.000

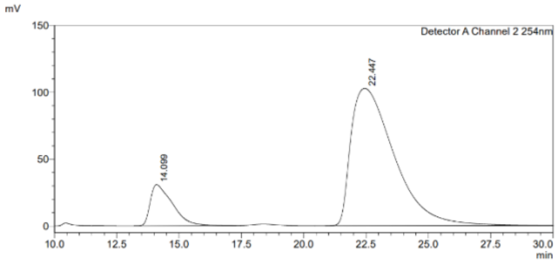

<Peak Table>

Detector A Channel 2 254nm

Peak#	Ret. Time	Area%
1	14.099	13.220
2	22.447	86.780
Total		100.000

The title alcohol (*R*)-1-(1-(furan-2-ylmethyl)-3-methylbenzo[e][1,2]azaborinin-2(1H)-yl)naphthalen-2-ol **42** (44.9 mg, 41%): $[\alpha]_D^{20}$ +156.7 (*c* 0.8, $CHCl_3$); **Chiral HPLC analysis**, Chiralpak ID (98:2 hexane : IPA, flow rate 1.0 mL.min^{-1}, 254 nm, 30 °C) t_R (*R*): 9.1 min, t_R (*S*): 13.9 min, 87:13 er.

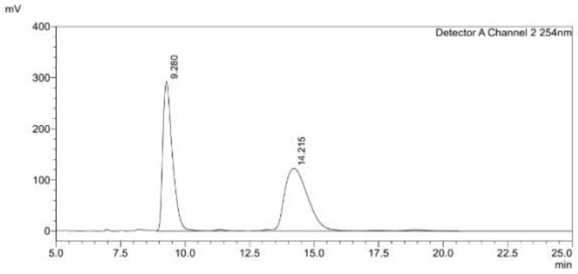

<Peak Table>

Detector A Channel 2 254nm

Peak#	Ret. Time	Area%
1	9.280	49.474
2	14.215	50.526
Total		100.000

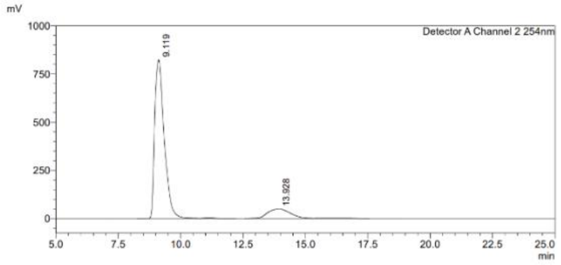

<Peak Table>

Detector A Channel 2 254nm

Peak#	Ret. Time	Area%
1	9.119	86.628
2	13.928	13.372
Total		100.000

(*S*)-1-(1-isopropyl-3-methylbenzo[*e*][1,2]azaborinin-2(1*H*)-yl)naphthalen-2-yl 2,2 diphenylacetate **45**

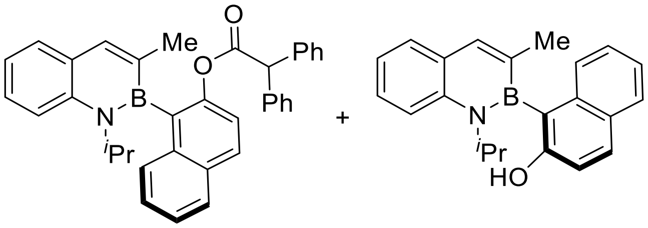

c =29
s = 40

Following general procedure **E**, 1-(1-isopropyl-3-methylbenzo[*e*][1,2]azaborinin-2(1*H*)-yl)naphthalen-2-ol **44** (97.2 mg, 0.30 mmol), 2,2-diphenylacetic pivalic anhydride **13** (62.2 mg, 0.21 mmol), (*R*)-BTM **14** (3.8 mg, 0.015 mmol), Et_3N (25.2 μL, 0.18 mmol) in $CHCl_3$ (3 mL) for 16 h gave, after purification on boron capped silica by Biotage® Selekt™ (Sfär HC 25 g, 80 mL.min^{-1}, Cyclohexane : Petrol [100:0 to 90:10 10 CV, 90:10 to 75:25 10 CV]) the title ester **45** (48.8 mg, 31%) as an off-white amorphous solid. $[\alpha]_D^{20}$ –0.51 (*c* 0.6, $CHCl_3$); **Chiral HPLC analysis**, Chiralpak IA (99.5:0.5 hexane : IPA, flow rate 1.0 mL.min^{-1}, 220 nm, 30 °C) t_R (*R*): 12.2 min, t_R (*S*): 13.9 min, 3:97 er; **IR** ν_{max} (film) 2936, 1749, 1611, 1296, 1121, 972; **^{1}H NMR** (400 MHz, $CDCl_3$) δ_H: 1.24 (3H, d, *J* 7.2), 1.46 (3H, d, *J* 7.2), 1.89 (3H, s), 4.58 (1H, hept, *J* 6.1), 5.08 (1H, s), 6.96–7.01 (4H, m), 7.06–7.14 (6H, m), 7.31 (1H, d, *J* 8.8), 7.34–7.38 (2H,m), 7.46–7.51 (3H, m), 7.77–7.79 (3H, m), 7.90 (1H, d, *J* 9.1), 7.93 (1H, d, *J* 7.7); **^{13}C{^{1}H} NMR** (101 MHz, $CDCl_3$) δ_C: 21.2, 21.7, 21.8, 53.5, 57.4, 118.9, 120.8, 121.5, 125.6, 126.1, 126.2, 127.2, 127.2, 128.2, 128.5, 128.5, 128.6, 128.6, 128.9, 129.0, 130.3, 131.7, 135.7, 137.6, 138.0, 139.1, 140.6 (br s), 142.0, 149.3, 171.4; **^{11}B{^{1}H} NMR** (128 MHz, $CDCl_3$) δ_B: 39.64 (br s); **HRMS** (ESI)$^+$ $C_{36}H_{32}BNO_2Na$ [M+Na]$^+$ found 544.2415, requires 544.2418 (–0.68 ppm).

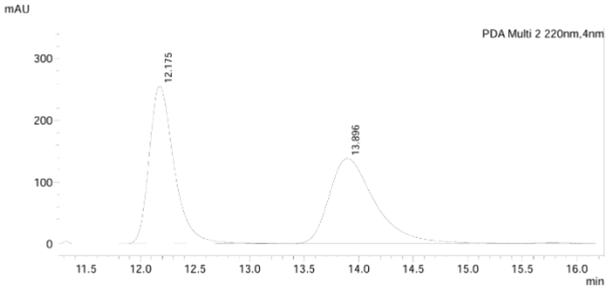

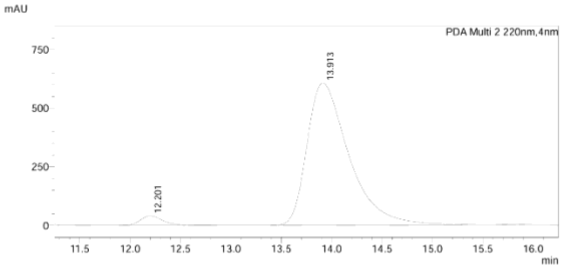

<Peak Table>
PDA Ch2 220nm

Peak#	Ret. Time	Area%
1	12.175	50.160
2	13.896	49.840
Total		100.000

<Peak Table>
PDA Ch2 220nm

Peak#	Ret. Time	Area%
1	12.201	3.478
2	13.913	96.522
Total		100.000

The title alcohol (*R*)-1-(1-isopropyl-3-methylbenzo[e][1,2]azaborinin-2(1H)-yl)naphthalen-2-ol **44** (60.1 mg, 61%): $[a]_D^{20}$ +0.54 (*c* 1.2, $CHCl_3$); **Chiral HPLC analysis**, Chiralpak IA (98:2 hexane : IPA, flow rate 1.0 mL.min^{-1}, 211 nm, 30 °C) t_R (*R*): 20.0 min, t_R (*S*): 24.4 min, 69:31 er.

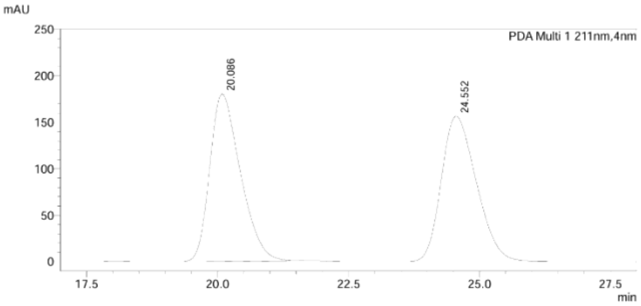

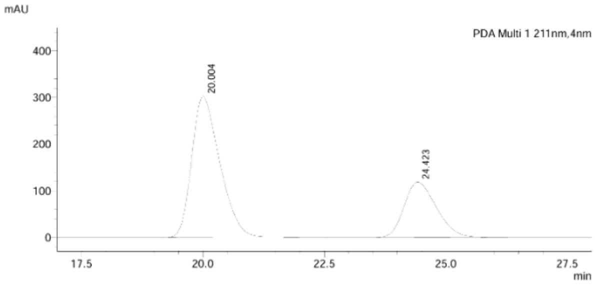

<Peak Table>
PDA Ch1 211nm

Peak#	Ret. Time	Area%
1	20.086	50.165
2	24.552	49.835
Total		100.000

<Peak Table>
PDA Ch1 211nm

Peak#	Ret. Time	Area%
1	20.004	68.825
2	24.423	31.175
Total		100.000

(*S*)-1-(1-cyclopropyl-3-methylbenzo[*e*][1,2]azaborinin-2(1*H*)-yl)naphthalen-2-yl
2,2 diphenylacetate **47**

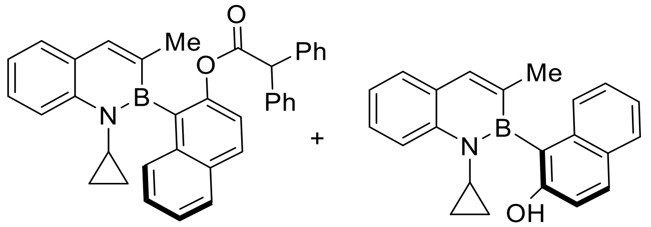

c = 47
s = 140

Following general procedure **E**, 1-(1-cyclopropyl-3-methylbenzo[*e*][1,2]azaborinin-2(1*H*)-yl)naphthalen-2-ol **46** (98.6 mg, 0.30 mmol), 2,2-diphenylacetic pivalic anhydride **13** (62.2 mg, 0.21 mmol), (*R*)-BTM **14** (3.8 mg, 0.015 mmol), Et_3N (25.2 μL, 0.18 mmol) in $CHCl_3$ (3 mL) for 15 h gave, after purification on boron capped silica by Biotage® Selekt™ (Sfär HC 25 g, 80 mL.min^{-1}, Petrol : EtOAc [100:0 to 90:10 10 CV, 90:10 to 75:25 10 CV]), the title ester **47** (60.6 mg, 39%) as a white amorphous solid. $[a]_D^{20}$+42.3 (*c* 1.1, $CHCl_3$); **Chiral HPLC analysis**, Chiralpak AD-H (98:2 hexane : IPA, flow rate 1.0 mL.min^{-1}, 254 nm,

30 °C) t_R (*S*): 9.8 min, t_R (*R*): 10.6 min, 98:2 er; **IR** ν_{max} (film) 3059, 2955, 1748, 1611, 1327, 1115, 907; **^{1}H NMR** (400 MHz, $CDCl_3$) δ_H: 0.20–0.26 (1H, m), 0.43–0.60 (3H, m), 1.88 (3H, d, *J* 1.3), 2.85 (1H, ddd, *J* 7.2, 4.7, 2.6), 5.01 (1H, s), 6.97–7.10 (10H, m), 7.30–7.37 (3H, m), 7.42—7.46 (2H, m), 7.52 (1H, ddd, *J* 8.7, 7.0, 1.7), 7.69 (1H, dd, *J* 7.8, 1.7), 7.74 (1H, s), 7.86–7.91 (2H, m), 8.02 (1H, d, *J* 8.5); **^{13}C{^{1}H} NMR** (126 MHz, $CDCl_3$) δ_C: 9.7, 10.1, 21.5, 32.0, 57.4, 117.2, 121.2, 121.4, 125.3, 126.3, 127.0, 127.2, 127.3, 127.3, 128.4, 128.4, 128.5, 128.5, 128.6, 128.6, 129.0, 129.4, 131.5, 135.7, 137.8, 138.1, 142.0, 142.6, 149.3, 171.3; **^{11}B{^{1}H} NMR** (128 MHz, $CDCl_3$) δ_B: 40.15 (br s); **HRMS** (ESI$^+$) $C_{36}H_{31}BNO_2$ [M+H]$^+$ found 520.2440, requires 520.2442 (–0.46 ppm).

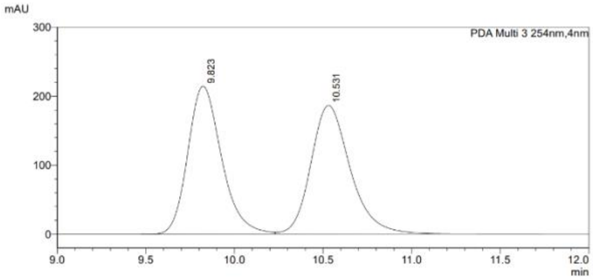

<Peak Table>

PDA Ch3 254nm

Peak#	Ret. Time	Area%
1	9.823	49.686
2	10.531	50.314
Total		100.000

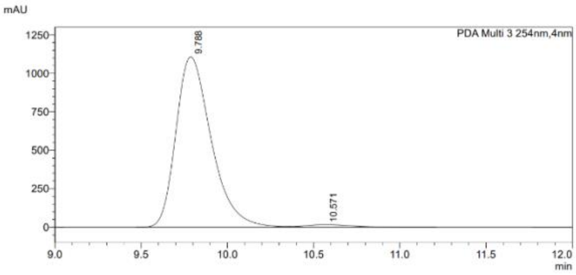

<Peak Table>

PDA Ch3 254nm

Peak#	Ret. Time	Area%
1	9.788	98.076
2	10.571	1.924
Total		100.000

The title alcohol (*R*)-1-(1-cyclopropyl-3-methylbenzo[e][1,2]azaborinin-2(1H)-yl)naphthalen-2-ol **46** (37.5 mg, 36%): $[\alpha]_D^{20}$+86.3 (*c* 1.8, $CHCl_3$); **Chiral HPLC analysis**, Chiralpak IA (97:3 hexane : IPA, flow rate 1.0 mL.min^{-1}, 254 nm, 30 °C) t_R (*R*): 13.2 min, t_R (*S*): 17.0 min, 92:8 er.

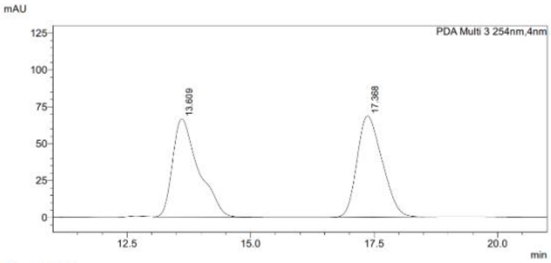

<Peak Table>

PDA Ch3 254nm

Peak#	Ret. Time	Area%
1	13.609	50.178
2	17.368	49.822
Total		100.000

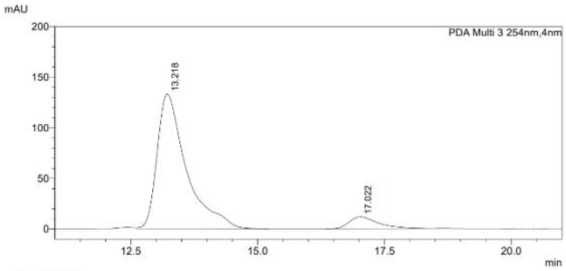

<Peak Table>

PDA Ch3 254nm

Peak#	Ret. Time	Area%
1	13.218	92.060
2	17.022	7.940
Total		100.000

(*S*)-1-(1-cyclobutyl-3-methylbenzo[e][1,2]azaborinin-2(1H)-yl)naphthalen-2-yl 2,2 diphenylacetate **49**

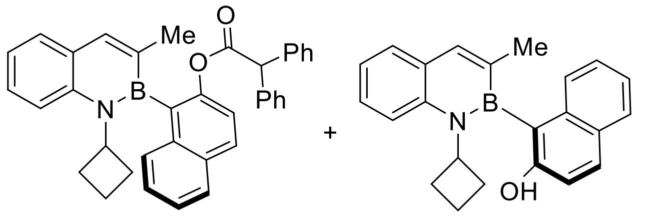

c = 40
s = 100

Following general procedure **E**, 1-(1-cyclobutyl-3-methylbenzo[e][1,2]azaborinin-2(1H)-yl)naphthalen-2-ol **48** (102 mg, 0.30 mmol), 2,2-diphenylacetic pivalic anhydride **13** (62.2 mg, 0.21 mmol), (*R*)-BTM **14** (3.8 mg, 0.015 mmol), Et_3N (25.2 μL, 0.18 mmol) in $CHCl_3$ (3 mL) for 16 h gave, after purification on boron capped silica by Biotage® Selekt™ (Sfär HC 25 g, 80 mL.min^{-1}, Petrol : EtOAc [100:0 to 90:10 15 CV, 90:10 to 75:25 10 CV]), the title ester **49** (56.7 mg, 35%) as a white amorphous solid. $[\alpha]_D^{20}$ –19.0 (*c* 1.1, $CHCl_3$); **Chiral HPLC analysis**, Chiralpak IA (98:2 hexane : IPA, flow rate 1.0 mL.min^{-1}, 270 nm, 30 °C) t_R (*R*): 8.0 min, t_R (*S*): 9.4 min, 2:98 er; **IR** ν_{max} (film) 2951, 1749, 1611, 1495, 1315, 1190, 1117, 907; **^{1}H NMR** (400 MHz, $CDCl_3$) δ_H: 1.39–1.56 (2H, m), 1.80 (3H, s), 1.86–2.03 (2H, m), 2.17 (2H, hept, *J* 10.1), 4.70 (1H, p, *J* 8.7), 5.00 (1H, s), 7.03 (10H, app ddd, *J* 21.1, 13.7, 7.2), 7.27 (1H, d, *J* 8.9), 7.34 (2H, app dt, *J* 15.0, 7.1), 7.42–7.52 (3H, m), 7.70–7.74 (3H, m), 7.87 (1H, d, *J* 8.9), 7.89 (1H, d, *J* 8.0); **^{13}C{^{1}H} NMR** (126 MHz, $CDCl_3$) δ_C: 14.8, 21.6, 31.1, 31.2, 56.7, 57.3, 117.5, 121.2, 121.2, 125.4, 126.3, 126.5, 127.2, 128.2, 128.5, 128.5, 128.6, 128.7, 129.0, 129.9, 131.5, 135.9, 137.9, 138.1, 140.2, 141.8, 149.3, 171.4; **^{11}B{^{1}H} NMR** (128 MHz, $CDCl_3$) δ_B: 37.67 (br s); **HRMS** (ESI)$^+$ $C_{37}H_{32}BNO_2Na$ [M+Na]$^+$ found 556.2422, requires 556.2418 (+0.67 ppm).

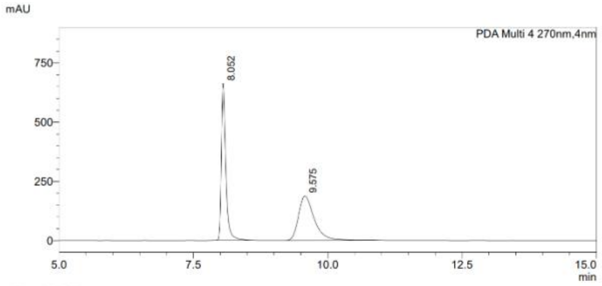

<Peak Table>
PDA Ch4 270nm

Peak#	Ret. Time	Area%
1	8.052	49.929
2	9.575	50.071
Total		100.000

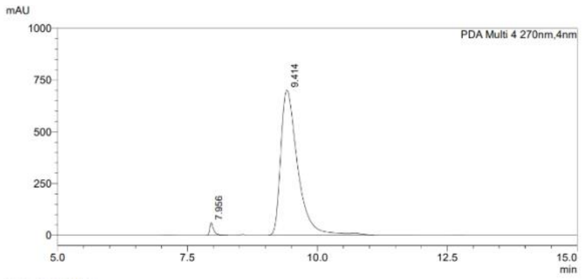

<Peak Table>
PDA Ch4 270nm

Peak#	Ret. Time	Area%
1	7.956	1.945
2	9.414	98.055
Total		100.000

The title alcohol (*R*)-1-(1-cyclobutyl-3-methylbenzo[e][1,2]azaborinin-2(1H)-yl)naphthalen-2-ol **48** (31.3 mg, 31%): $[\alpha]_D^{20}$ +83.1 (*c* 1.4, $CHCl_3$); **Chiral HPLC analysis**, Chiralpak IB (98:2 hexane : IPA, flow rate 1.0 mL.min^{-1}, 254 nm, 30 °C) t_R (*R*): 10.9 min, t_R (*S*): 27.8 min, 82:18 er.

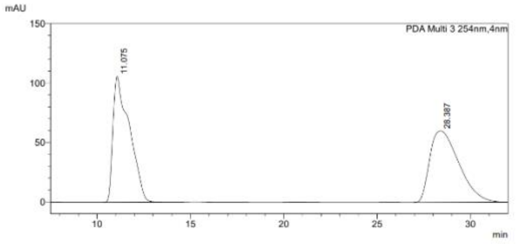

<Peak Table>

PDA Ch3 254nm

Peak#	Ret. Time	Area%
1	11.075	50.038
2	28.387	49.962
Total		100.000

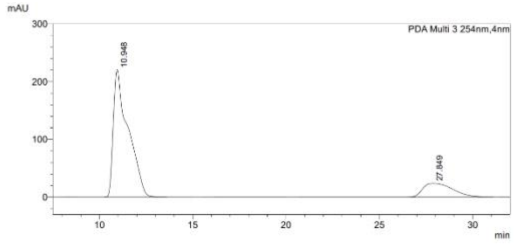

<Peak Table>

PDA Ch3 254nm

Peak#	Ret. Time	Area%
1	10.948	82.192
2	27.849	17.808
Total		100.000

(*S*)-1-(1-(cyclohexylmethyl)-3-methylbenzo[e][1,2]azaborinin-2(1H)-yl)naphthalen-2-yl 2,2-diphenylacetate **51**

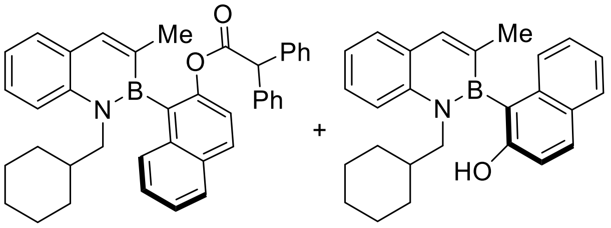

c = 46
s = 160

Following general procedure **E**, 1-(1-(cyclohexylmethyl)-3-methylbenzo[e][1,2]azaborinin-2(1H)-yl)naphthalen-2-ol **50** (114 mg, 0.30 mmol), 2,2-diphenylacetic pivalic anhydride **13** (62.2 mg, 0.21 mmol), (*R*)-BTM **14** (3.8 mg, 0.015 mmol), Et_3N (25.2 µL, 0.18 mmol) in $CHCl_3$ (3 mL) for 16 h gave, after purification by Biotage® Selekt™ (Sfär HC 25 g, 80 mL.min^{-1}, Petrol : EtOAc [100:0 to 90:10 15 CV, 90:10 CV to 72:25 10 CV]), the title ester **51** (64.9 mg, 38%) as a white amorphous solid. $[\alpha]_D^{20}$ +74.4 (*c* 1.3, $CHCl_3$); **Chiral HPLC analysis**, Chiralpak IA (99.5:0.5 hexane : IPA, flow rate 1.0 mL.min^{-1}, 254 nm, 30 °C) t_R (*S*): 9.8 min, t_R (*R*): 10.7 min, 98:2 er; **IR** ν_{max} (film) 3061, 2922, 1751, 1609, 1493, 1340, 1192, 1115, 907; **^{1}H NMR** (400 MHz, $CDCl_3$) δ_H: 0.27–0.35 (1H, m), 0.57–0.67 (1H, m), 0.75–0.95 (3H, m), 1.29–1.45 (5H, m), 1.68–1.76 (1H, m), 1.83 (3H, d, *J* 1.3), 3.52 (1H, dd, *J* 14.1, 5.9), 3.73 (1H, dd, *J* 14.1, 7.6), 4.99 (1H, s), 6.98–7.16 (10H, m), 7.29–7.36 (3H, m), 7.41–7.52 (4H, m), 7.71 (1H, dd, *J* 7.8, 1.6), 7.75 (1H, s), 7.89 (2H, app dd, *J* 8.8, 2.7); **^{13}C{^{1}H} NMR** (101 MHz, $CDCl_3$) δ_C: 21.7, 25.8, 26.0, 26.2, 31.0, 31.3, 37.0, 55.3, 57.4, 116.4, 121.1, 121.1, 125.4, 126.0, 127.0, 127.2, 127.3, 127.8, 128.5, 128.5, 128.5, 128.6, 128.7, 128.8, 129.4, 129.8, 131.6, 135.9, 137.9, 138.1, 140.6, 141.9, 149.5, 171.1; **^{11}B{^{1}H} NMR** (128 MHz, $CDCl_3$) δ_B: 37.19 (br s); **HRMS** (ESI)$^+$ $C_{40}H_{38}BNO_2Na$ [M+Na]$^+$ found 598.2886, requires 598.2888 (–0.32 ppm).

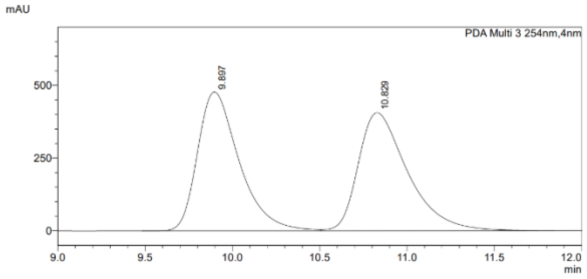

<Peak Table>

PDA Ch3 254nm

Peak#	Ret. Time	Area%
1	9.897	49.756
2	10.829	50.244
Total		100.000

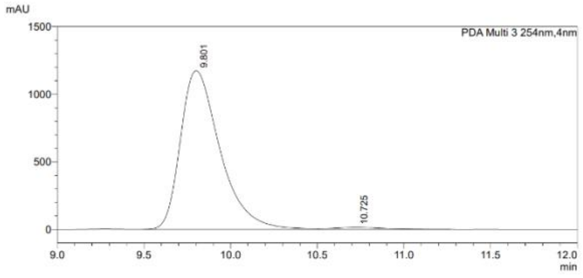

<Peak Table>

PDA Ch3 254nm

Peak#	Ret. Time	Area%
1	9.801	98.344
2	10.725	1.656
Total		100.000

The title alcohol (*R*)-1-(1-(cyclohexylmethyl)-3-methylbenzo[e][1,2]azaborinin-2(1H)-yl)naphthalen-2-ol **50** (46.7 mg, 41%): $[\alpha]_D^{20}$ +171.8 (*c* 0.9, $CHCl_3$); **Chiral HPLC analysis**, Chiralpak IC (98:2 hexane : IPA, flow rate 1.0 mL.min^{-1}, 254 nm, 30 °C) t_R (*S*): 4.0 min, t_R (*R*): 5.3 min, 8:92 er.

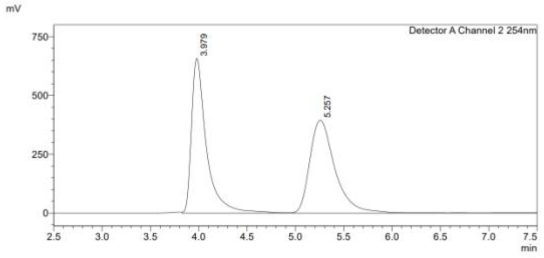

<Peak Table>

Detector A Channel 2 254nm

Peak#	Ret. Time	Area%
1	3.979	49.496
2	5.257	50.504
Total		100.000

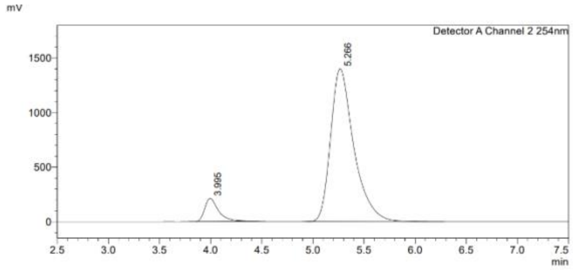

<Peak Table>

Detector A Channel 2 254nm

Peak#	Ret. Time	Area%
1	3.995	8.315
2	5.266	91.685
Total		100.000

(*S*)-1-(1-(cyclohexylmethyl)-3,4-dimethylbenzo[e][1,2]azaborinin-2(1H)-yl)naphthalen-2-yl 2,2-diphenylacetate **53**

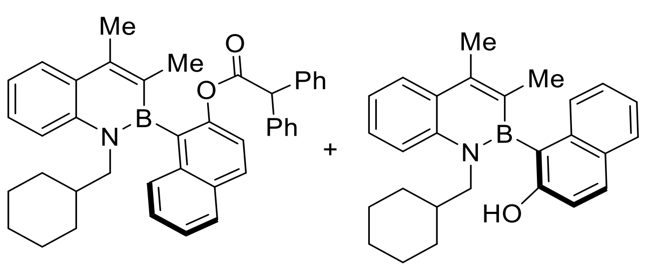

c = 43
s = >200

Following general procedure **E**, 1-(1-(cyclohexylmethyl)-3,4-dimethylbenzo[e][1,2]azaborinin-2(1H)-yl)naphthalen-2-ol **52** (119 mg, 0.30 mmol), 2,2-diphenylacetic pivalic anhydride **13** (62.2 mg, 0.21 mmol), (*R*)-BTM **14** (3.8 mg, 0.015 mmol), Et_3N (25.2 µL, 0.18 mmol) in $CHCl_3$ (3 mL) for 16 h gave, after purification by Biotage® Selekt™ (Sfär HC 25 g, 80 mL.min^{-1}, Petrol : EtOAc [100:0 to 90:10 10 CV, 90:10

to 75:25 10 CV]), the title ester **53** (73.1 mg, 41%) as a white amorphous solid. $[\alpha]_D^{20}$ +59.2 (*c* 1.3, $CHCl_3$); **Chiral HPLC analysis**, Chiralcel OD-H (99.5:0.5 hexane : IPA, flow rate 1.0 mL.min^{-1}, 270 nm, 30 °C) t_R (*S*): 5.2 min, t_R (*R*): 7.0 min, 99:1 er; **IR** ν_{max} (film) 2922, 1751, 1587, 1285, 1117, 908; **^{1}H NMR** (400 MHz, $CDCl_3$) δ_H: 0.27–0.40 (1H, m), 0.57–0.67 (1H, m), 0.77–0.99 (3H, m), 1.29–1.48 (5H, m), 1.70–1.77 (4H, m), 2.51 (3H, s), 3.55 (1H, dd, *J* 14.2, 6.0), 3.73 (1H, dd, *J* 14.3, 7.4), 4.99 (1H, s), 7.01–7.19 (10H, m), 7.31–7.37 (3H, m), 7.42–7.54 (4H, m), 7.89 (2H, dd, *J* 8.7, 2.7), 8.08 (1H, dd, *J* 8.1, 1.4); **^{13}C{^{1}H} NMR** (126 MHz, $CDCl_3$) δ_C: 15.8, 18.4, 25.8, 26.0, 26.2, 31.0, 31.3, 37.0, 55.4, 57.4, 116.7, 120.8, 121.2, 125.4, 125.9, 125.9, 126.6, 127.2, 127.3, 128.2, 128.4, 128.5, 128.6, 128.6, 129.0, 129.2, 131.5, 132.1 (br s), 136.1, 137.3 (br s), 137.9, 138.1, 140.5, 145.5, 149.6, 171.0; **^{11}B{^{1}H} NMR** (160 MHz, $CDCl_3$) δ_B: 37.86 (s); **HRMS** (ESI)$^+$ $C_{41}H_{40}BNO_2Na$ [M+Na]$^+$ found 612.3043, requires 612.3044 (–0.28 ppm).

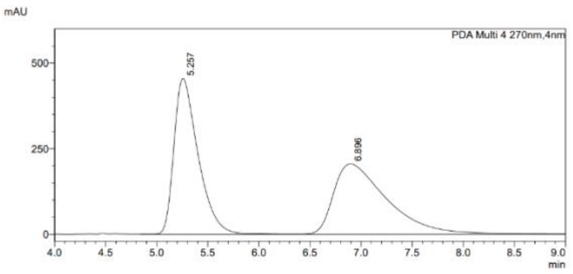

<Peak Table>

PDA Ch4 270nm

Peak#	Ret. Time	Area%
1	5.257	50.026
2	6.896	49.974
Total		100.000

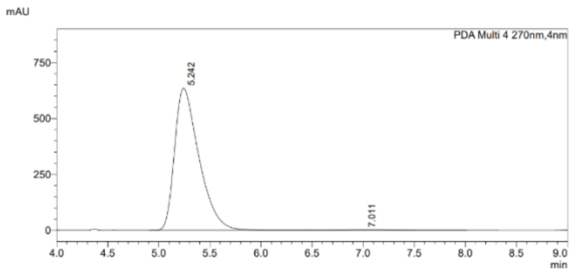

<Peak Table>

PDA Ch4 270nm

Peak#	Ret. Time	Area%
1	5.242	99.166
2	7.011	0.834
Total		100.000

The title alcohol (*R*)-1-(1-(cyclohexylmethyl)-3,4-dimethylbenzo[e][1,2]azaborinin-2(1H)-yl)naphthalen-2-ol **52** (64.8 mg, 55%): $[\alpha]_D^{20}$ +147.2 (*c* 1.2, $CHCl_3$); **Chiral HPLC analysis**, Chiralpak IB (99.5:0.5 hexane : IPA, flow rate 1.0 mL.min^{-1}, 270 nm, 30 °C) t_R (*S*): 15.8 min, t_R (*R*): 19.3 min, 13:87 er.

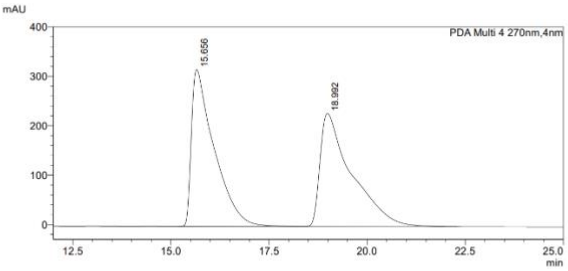

<Peak Table>

PDA Ch4 270nm

Peak#	Ret. Time	Area%
1	15.656	50.033
2	18.992	49.967
Total		100.000

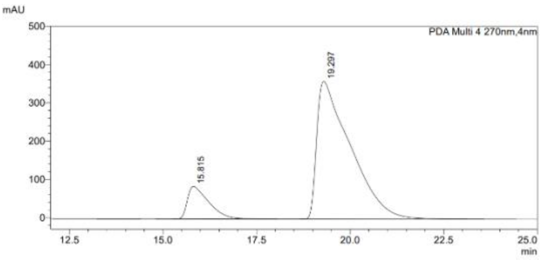

<Peak Table>

PDA Ch4 270nm

Peak#	Ret. Time	Area%
1	15.815	13.022
2	19.297	86.978
Total		100.000

(*S*)-1-(1-(cyclohexylmethyl)benzo[e][1,2]azaborinin-2(1H)-yl)naphthalen-2-yl 2,2 diphenylacetate **55**

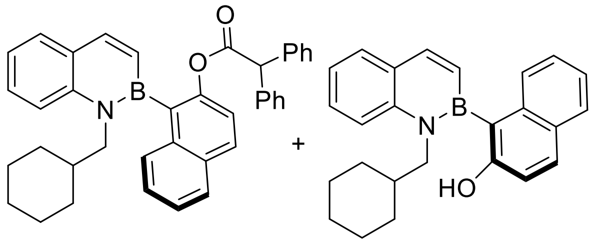

c = 46
s = 90

Following general procedure **E**, 1-(1-(cyclohexylmethyl)benzo[e][1,2]azaborinin-2(1H)-yl)naphthalen-2-ol **54** (117 mg, 0.30 mmol), 2,2-diphenylacetic pivalic anhydride **13** (62.2 mg, 0.21 mmol), (*R*)-BTM **14** (3.8 mg, 0.015 mmol), Et_3N (25.2 µL, 0.18 mmol) in $CHCl_3$ (3 mL) for 16 h gave, after purification on boron-capped silica by Biotage® Selekt™ (Sfär HC 25 g, 80 mL.min^{-1}, Petrol : EtOAc [100:0 to 90:10 10 CV, 90:10 to 75:25 10 CV]), the title ester **55** (65.4 mg, 39%) as a white amorphous solid. $[\alpha]_D^{20}$ +93.8 (*c* 0.2, $CHCl_3$); **Chiral HPLC analysis**, Chiralpak IA (99.5:0.5 hexane : IPA, flow rate 1.0 mL.min^{-1}, 270 nm, 30 °C) t_R (*S*): 14.5 min, t_R (*R*): 19.3 min, 97:3 er; **IR** ν_{max} (film) 2922, 1748, 1551, 1493, 1186, 1119, 908; **^{1}H NMR** (400 MHz, $CDCl_3$) δ_H: 0.39 (1H, q, *J* 11.1), 0.61 (1H, q, *J* 10.3, 8.7), 0.84–0.98 (3H, m), 1.30–1.49 (5H, m), 1.75 (1H, ddq, *J* 11.4, 7.4, 3.7), 3.59 (1H, dd, *J* 14.0, 6.4), 3.85 (1H, dd, *J* 14.1, 7.0), 5.04 (1H, s), 6.88 (1H, d, *J* 11.3), 7.06–7.39 (13H, m), 7.44–7.50 (2H, m), 7.56–7.64 (2H, m), 7.79 (1H, dd, *J* 7.8, 1.6), 7.90 (2H, dd, *J* 8.7, 2.9), 7.99 (1H, d, *J* 11.2); **^{13}C{^{1}H} NMR** (126 MHz, $CDCl_3$) δ_C: 25.8, 25.9, 26.2, 30.8, 31.1, 36.7, 55.2, 57.4, 116.6, 121.0, 121.2, 125.3, 126.0, 127.2, 127.3, 127.9, 128.2, 128.5, 128.5, 128.6, 128.6, 128.7, 129.0, 129.5, 130.7, 131.7, 136.1, 138.0, 138.1, 141.5, 144.6, 149.8, 171.2; **^{11}B{^{1}H} NMR** (128 MHz, $CDCl_3$) δ_B: 39.22 (br s); **HRMS** (ESI)$^+$ $C_{39}H_{36}BNO_2Na$ $[M+Na]^+$ found 584.2730, requires 584.2731 (–0.23 ppm).

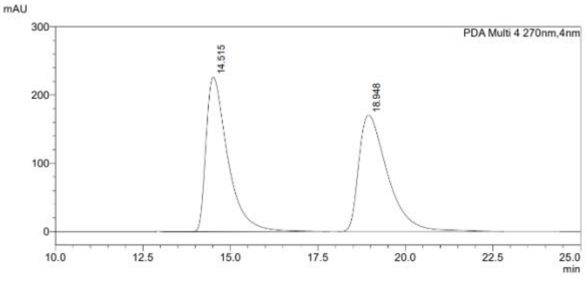

<Peak Table>

PDA Ch4 270nm

Peak#	Ret. Time	Area%
1	14.515	50.073
2	18.948	49.927
Total		100.000

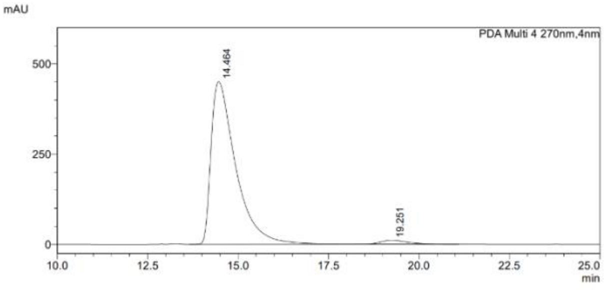

<Peak Table>

PDA Ch4 270nm

Peak#	Ret. Time	Area%
1	14.464	97.343
2	19.251	2.657
Total		100.000

The title alcohol (*R*)-1-(1-(cyclohexylmethyl)benzo[e][1,2]azaborinin-2(1H)-yl)naphthalen-2-ol **54** (51.1 mg, 46%): $[\alpha]_D^{20}$ +177.9 (*c* 0.9, $CHCl_3$); **Chiral HPLC analysis**, Chiralcel OD-H (99:1 hexane : IPA, flow rate 1.0 mL.min^{-1}, 254 nm, 30 °C) t_R (*R*): 25.0 min, t_R (*S*): 37.9 min, 91:9 er.

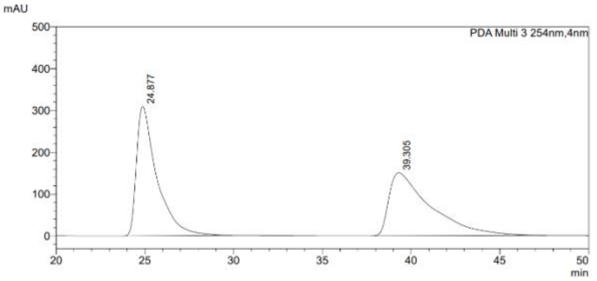

<Peak Table>

PDA Ch3 254nm

Peak#	Ret. Time	Area%
1	24.877	50.224
2	39.305	49.776
Total		100.000

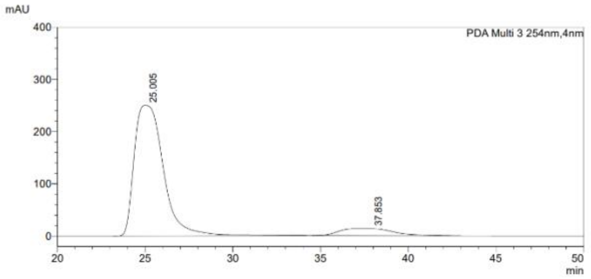

<Peak Table>

PDA Ch3 254nm

Peak#	Ret. Time	Area%
1	25.005	90.892
2	37.853	9.108
Total		100.000

(*S*)-1-(1-cyclopentyl-3,4-dimethylbenzo[e][1,2]azaborinin-2(1H)-yl)naphthalen-2-yl 2,2-diphenylacetate **57**

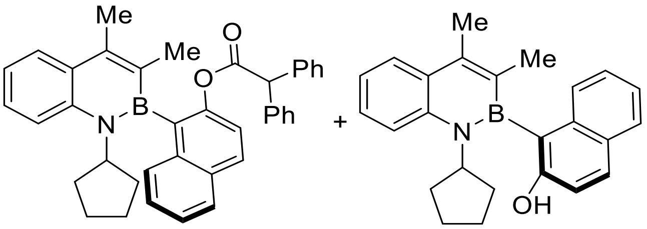

c = 11
s = 20

Following general procedure **E**, 1-(1-cyclopentyl-3,4-dimethylbenzo[e][1,2]azaborinin-2(1H)-yl)naphthalen-2-ol **56** (110 mg, 0.30 mmol), 2,2-diphenylacetic pivalic anhydride **13** (62.2 mg, 0.21 mmol), (*R*)-BTM **14** (3.8 mg, 0.015 mmol), Et_3N (25.2 µL, 0.18 mmol) in $CHCl_3$ (3 mL) for 16 h gave, after purification by Biotage® Selekt™ (Sfär HC 25 g, 80 mL.min^{-1}, Petrol : EtOAc [100:0 to 90:10 10 CV, 90:10 to 75:25 10 CV]), the title ester **57** (20.7 mg, 12%) as a white amorphous solid. $[\alpha]_D^{20}$ –61.7 (*c* 0.9, $CHCl_3$); **Chiral HPLC analysis**, Chiralpak IC (99:1 hexane : IPA, flow rate 1.0 mL.min^{-1}, 254 nm, 30 °C) t_R (*S*): 5.1 min, t_R (*R*): 6.0 min, 95:5 er; **IR** ν_{max} (film) 2953, 1746, 1587, 1310, 1192, 1117, 907; **^{1}H NMR** (400 MHz, $CDCl_3$) δ_H: 1.39–1.68 (4H, m), 1.77 (3H, s), 1.83–1.93 (2H, m), 1.98–2.08 (1H, m), 2.31–2.40 (1H, m), 2.52 (3H, s), 4.73 (1H, p, *J* 9.5), 5.05 (1H, s), 6.93–6.99 (4H, m), 7.03–7.10 (6H, m), 7.30 (1H, d, *J* 8.8), 7.36 (2H, dd, *J* 14.7, 6.9), 7.44–7.52 (3H, m), 7.58 (1H, d, *J* 9.8), 7.91 (2H, dd, *J* 10.1, 8.3), 8.12 (1H, dd, *J* 8.2, 1.7); **^{13}C{^{1}H} NMR** (126 MHz, $CDCl_3$) δ_C: 15.8, 18.3, 25.7, 28.8, 29.4, 57.4, 63.1, 118.7, 120.6, 121.4, 125.4, 125.5, 126.0, 126.5, 127.1, 128.4, 128.4, 128.5, 128.6, 128.6, 128.9, 129.3, 131.7, 132.9 (br s), 135.8, 137.1 (br s), 137.8, 138.0, 138.3, 145.4, 149.3, 171.4; **^{11}B{^{1}H}**

NMR (160 MHz, $CDCl_3$) δ_B: 37.72 (br s); **HRMS** (ESI)$^+$ $C_{39}H_{36}BNO_2Na$ [M+Na]$^+$ found 584.2725, requires 584.2731 (–1.14 ppm).

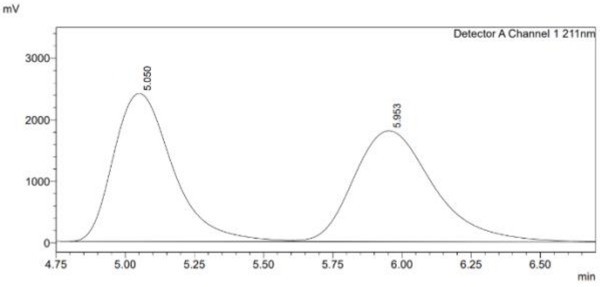

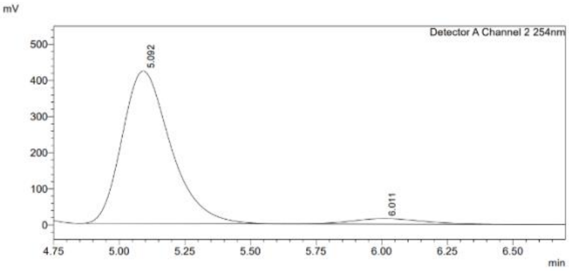

<Peak Table>

Detector A Channel 1 211nm

Peak#	Ret. Time	Area%
1	5.050	49.734
2	5.953	50.266
Total		100.000

<Peak Table>

Detector A Channel 2 254nm

Peak#	Ret. Time	Area%
1	5.092	94.779
2	6.011	5.221
Total		100.000

The title alcohol (*R*)-1-(1-cyclopentyl-3,4-dimethylbenzo[e][1,2]azaborinin-2(1H)-yl)naphthalen-2-ol **56** (69.7 mg, 63%): $[\alpha]_D^{20}$ +20.5 (*c* 1.3, $CHCl_3$); **Chiral HPLC analysis**, Chiralpak AD-H (99:1 hexane : IPA, flow rate 1.0 mL.min^{-1}, 270 nm, 30 °C) t_R (*S*): 35.7 min, t_R (*R*): 45.2 min, 44:56 er.

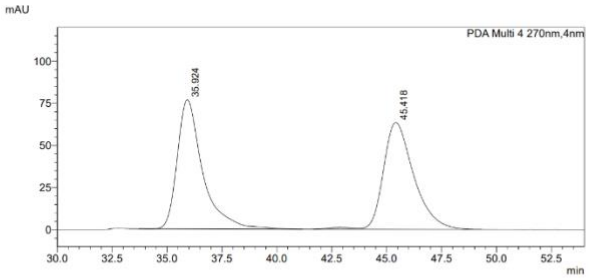

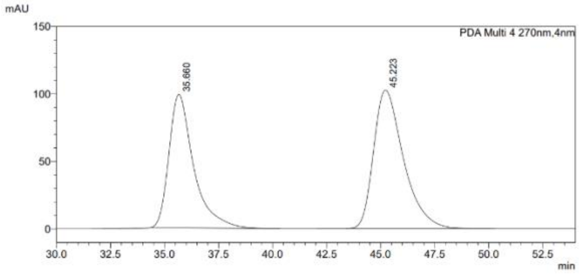

<Peak Table>

PDA Ch4 270nm

Peak#	Ret. Time	Area%
1	35.924	50.199
2	45.418	49.801
Total		100.000

<Peak Table>

PDA Ch4 270nm

Peak#	Ret. Time	Area%
1	35.660	44.375
2	45.223	55.625
Total		100.000

(*S*)-2-(1-benzyl-3-methylbenzo[e][1,2]azaborinin-2(1H)-yl)-3-methylphenyl 2,2 diphenylacetate **59**

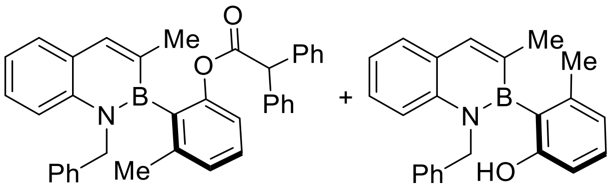

c = 27
s = 70

Following general procedure **E**, 2-(1-benzyl-3-methylbenzo[e][1,2]azaborinin-2(1H)-yl)-3-methylphenol **58** (67.9 mg, 0.20 mmol), 2,2-diphenylacetic anhydride **21** (56.9 mg, 0.14 mmol), (*R*)-BTM **14** (2.5 mg, 0.010 mmol), Et_3N (16.7 µL, 0.12 mmol) in $CHCl_3$ (2 mL) for 16 h gave, after purification by Biotage® Selekt™ (Sfär HC 25 g, 80 mL.min^{-1}, Petrol : EtOAc [100:0 to 90:10 12 CV, 90:10 to 75:25 10 CV]), the title ester **59** (26.3 mg, 16%) as

a white amorphous solid. $[a]_D^{20}$ +174.9 (*c* 1.1, $CHCl_3$); **Chiral HPLC analysis**, Chiralpak AD-H (99.5:0.5 hexane : IPA, flow rate 1.0 mL.min^{-1}, 270 nm, 30 °C) t_R (*R*): 14.1 min, t_R (*S*): 16.4 min, 2:98 er; **IR** ν_{max} (film) 3028, 1751, 1611, 1350, 1125, 1111, 906; **^{1}H NMR** (400 MHz, $CDCl_3$) δ_H: 2.04 (3H, s), 2.06 (3H, s), 4.92 (1H, d, *J* 16.9), 4.97 (1H, s), 5.16 (1H, d, *J* 16.9), 6.92–6.94 (2H, m), 6.96 (1H, d, *J* 8.1), 7.03–7.16 (14H, m), 7.21–7.35 (4H, m), 7.71 (1H, dd, *J* 7.6, 1.8), 7.81 (1H, s); **^{13}C{^{1}H} NMR** (126 MHz, $CDCl_3$) δ_C: 21.7, 22.0, 52.6, 57.3, 117.2, 118.7, 121.3, 126.1, 126.6, 127.1, 127.2, 127.2, 127.2, 127.5, 128.4, 128.5, 128.5, 128.6, 128.6, 129.5, 133.7 (br s), 137.9, 138.1, 138.7, 139.9 (br s), 140.2, 141.5, 142.2, 151.9, 171.2; **^{11}B{^{1}H} NMR** (128 MHz, $CDCl_3$) δ_B: 39.81 (br s); **HRMS** (ESI)$^+$ $C_{37}H_{32}BNO_2Na$ $[M+Na]^+$ found 556.2416, requires 556.2418 (–0.47 ppm).

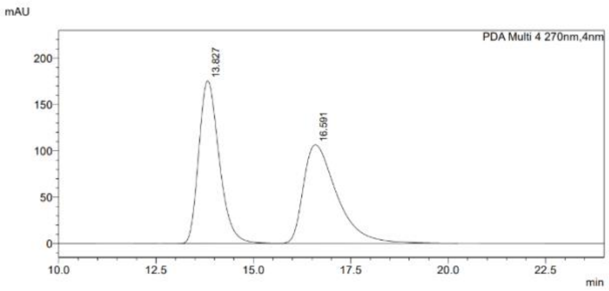

<Peak Table>

PDA Ch4 270nm

Peak#	Ret. Time	Area%
1	13.827	50.127
2	16.591	49.873
Total		100.000

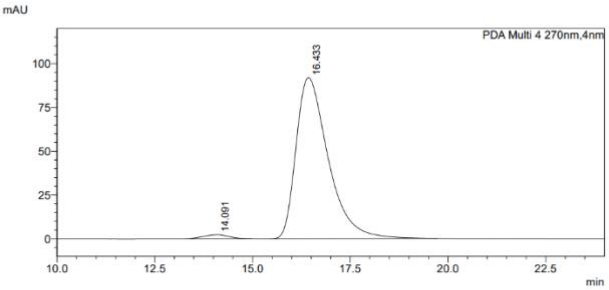

<Peak Table>

PDA Ch4 270nm

Peak#	Ret. Time	Area%
1	14.091	1.993
2	16.433	98.007
Total		100.000

The title alcohol (*R*)-2-(1-benzyl-3-methylbenzo[e][1,2]azaborinin-2(1H)-yl)-3-methylphenol **58** (45.7 mg, 67%): $[a]_D^{20}$ +11.1 (*c* 0.8, $CHCl_3$); **Chiral HPLC analysis**, Chiralpak IA (95:5 hexane : IPA, flow rate 1.0 mL.min^{-1}, 254 nm, 30 °C) t_R (*R*): 19.9 min, t_R (*S*): 25.6 min, 68:32 er.

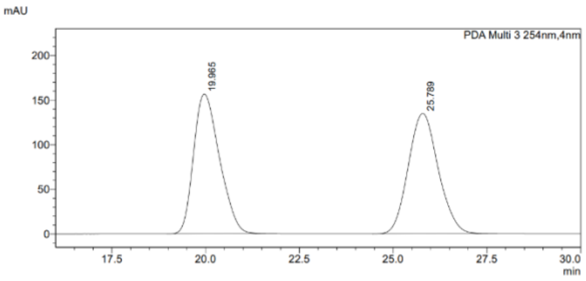

<Peak Table>

PDA Ch3 254nm

Peak#	Ret. Time	Area%
1	19.965	50.016
2	25.789	49.984
Total		100.000

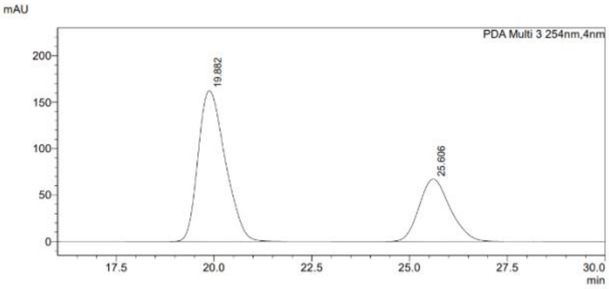

<Peak Table>

PDA Ch3 254nm

Peak#	Ret. Time	Area%
1	19.882	67.845
2	25.606	32.155
Total		100.000

(*S*)-1-(1-benzyl-3-methylbenzo[e][1,2]azaborinin-2(1H)-yl)-7-methoxynaphthalen-2-yl 2,2-diphenylacetate **61**

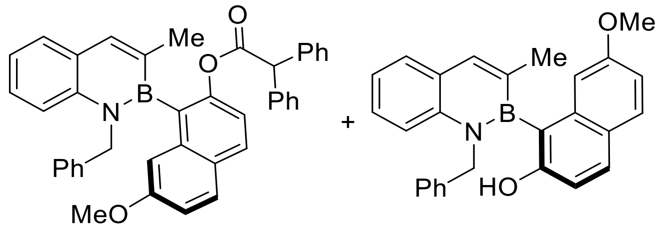

c = 44
s = 80

Following general procedure **E**, 1-(1-benzyl-3-methylbenzo[e][1,2]azaborinin-2(1H)-yl)-7-methoxynaphthalen-2-ol **60** (122 mg, 0.30 mmol), 2,2-diphenylacetic pivalic anhydride **13** (62.2 mg, 0.21 mmol), (*R*)-BTM **14** (3.8 mg, 0.015 mmol), Et_3N (25.2 µL, 0.18 mmol) in $CHCl_3$ (3 mL) for 16 h gave, after purification by Biotage® Selekt™ (Sfär HC 25 g, 80 mL.min^{-1}, Petrol : EtOAc [100:0 to 90:10 12 CV, 90:10 to 72:25 10 CV]), the title ester **61** (61.4 mg, 34%) as a white amorphous solid. $[\alpha]_D^{20}$ –8.3 (*c* 1.2, $CHCl_3$); **Chiral HPLC analysis**, Chiralpak IA (95:5 hexane : IPA, flow rate 1.0 mL.min^{-1}, 220 nm, 30 °C) t_R (*R*): 8.7 min, t_R (*S*): 11.9 min, 3:97 er; **IR** ν_{max} (film) 2934, 1748, 1611, 1452, 1229, 1123, 907; **^{1}H NMR** (400 MHz, $CDCl_3$) δ_H: 1.97 (3H, s), 3.63 (3H, s), 4.90 (1H, d, *J* 16.8), 5.02 (1H, s), 5.21 (1H, d, *J* 16.8), 6.77 (1H, d, *J* 2.5), 6.89 (2H, dd, *J* 6.6, 2.9), 7.05–7.15 (15H, m), 7.23–7.36 (3H, m), 7.72–7.77 (3H, m), 7.86 (1H, s); **^{13}C{^{1}H} NMR** (126 MHz, $CDCl_3$) δ_C: 21.7, 53.0, 55.3, 57.3, 106.7, 117.4, 118.0, 118.8, 126.1, 126.6, 127.0, 127.2, 127.3, 127.3, 127.7, 128.4, 128.5, 128.6, 128.6, 129.2, 129.6, 129.9, 137.2, 137.9, 138.1, 138.8, 140.3, 140.5 (br s), 142.3, 150.2, 157.8, 171.4; **^{11}B{^{1}H} NMR** (160 MHz, $CDCl_3$) δ_B: 39.20 (br s); **HRMS** (ESI)$^+$ $C_{41}H_{34}BNO_3Na$ [M+Na]$^+$ found 622.2527, requires 622.2524 (+0.49 ppm).

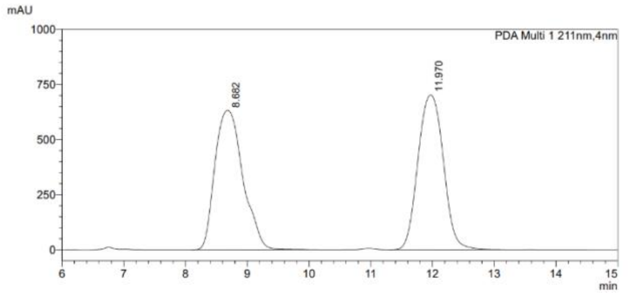

<Peak Table>

PDA Ch1 211nm

Peak#	Ret. Time	Area%
1	8.682	49.788
2	11.970	50.212
Total		100.000

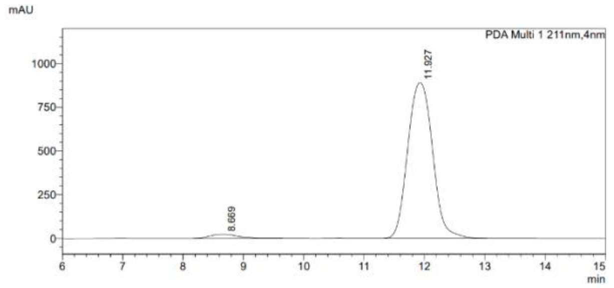

<Peak Table>

PDA Ch1 211nm

Peak#	Ret. Time	Area%
1	8.669	2.809
2	11.927	97.191
Total		100.000

The title alcohol (*R*)-1-(1-benzyl-3-methylbenzo[e][1,2]azaborinin-2(1H)-yl)-7-methoxynaphthalen-2-ol **60** (57.3 mg, 47%): $[\alpha]_D^{20}$ +230.7 (*c* 1.1, $CHCl_3$); **Chiral HPLC analysis**, Chiralpak IA (90:10 hexane : IPA, flow rate 1.0 mL.min^{-1}, 220 nm, 30 °C) t_R (*R*): 13.6 min, t_R (*S*): 18.4 min, 87:13 er.

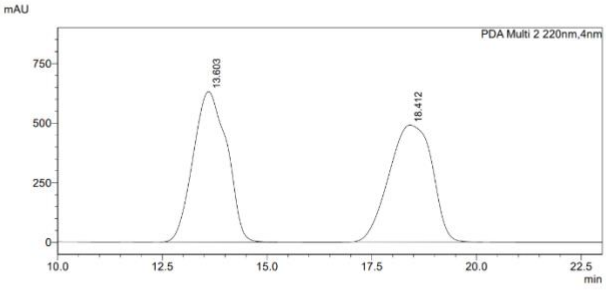

<Peak Table>

PDA Ch2 220nm

Peak#	Ret. Time	Area%
1	13.603	50.236
2	18.412	49.764
Total		100.000

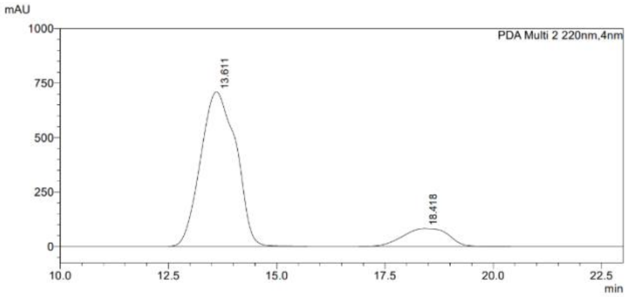

<Peak Table>

PDA Ch2 220nm

Peak#	Ret. Time	Area%
1	13.611	87.124
2	18.418	12.876
Total		100.000

(*S*)-1-(1-(4-methoxybenzyl)-3-methylbenzo[e][1,2]azaborinin-2(1H)-yl)naphthalen-2-yl 2,2-diphenylacetate **63**

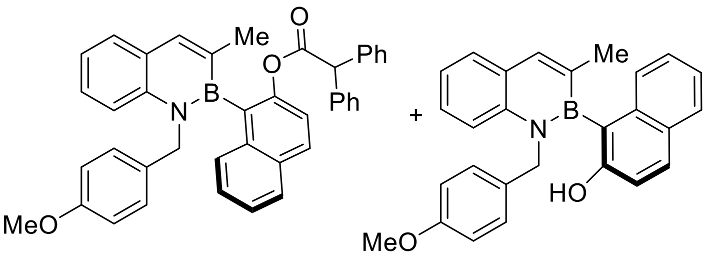

c = 45
s = 50

Following general procedure **E**, 1-(1-(4-methoxybenzyl)-3-methylbenzo[e][1,2]azaborinin-2(1H)-yl)naphthalen-2-ol **62** (81.1 mg, 0.20 mmol), 2,2-diphenylacetic pivalic anhydride **13** (41.5 mg, 0.14 mmol), (*R*)-BTM **14** (2.5 mg, 0.010 mmol), Et_3N (16.7 μL, 0.12 mmol) in $CHCl_3$ (2 mL) for 16 h gave, after purification by Biotage® Selekt™ (Sfär HC 25 g, 80 mL.min^{-1}, Petrol:EtOAc [100:0 to 90:10 10 CV, 90:10 to 75:25 10 CV, 75:25 to 50:50 10 CV]), the title ester **63** (37.4 mg, 31%) as a white amorphous solid. $[\alpha]_D^{20}$ +31.0 (*c* 1.8, $CHCl_3$); **Chiral HPLC analysis**, Chiralpak IA (97:3 hexane : IPA, flow rate 1.0 mL.min^{-1}, 211 nm, 30 °C) t_R (*R*): 16.0 min, t_R (*S*): 27.3 min, 4:96 er; **IR** ν_{max} (film) 3030, 1749, 1512, 1248, 1116, 724; **^{1}H NMR** (500 MHz, $CDCl_3$) δ_H: 1.95 (3H, s), 3.67 (3H, s), 4.85 (1H, d, *J* 16.4), 5.05 (1H, s), 5.09 (1H, d, *J* 16.4), 6.58 (2H, d, *J* 8.3), 6.80 (2H, d, *J* 8.2), 7.07–7.17 (10H, m), 7.25–7.36 (5H, m), 7.41 (1H, t, *J* 7.5), 7.50 (1H, d, *J* 8.3), 7.74 (1H, d, *J* 7.6), 7.83–7.86 (3H, m); **^{13}C{^{1}H} NMR** (126 MHz, $CDCl_3$) δ_C: 21.7, 52.6, 55.2, 57.4, 113.8, 117.3, 121.3, 121.4, 125.5, 126.1, 127.2, 127.3, 127.3, 127.3, 127.7, 128.2, 128.5, 128.6, 128.6, 128.6, 129.4, 129.6, 130.7, 131.6, 135.8, 137.9, 138.1, 140.3, 140.5 (br s), 142.2, 149.7, 158.2, 171.3; **^{11}B{^{1}H} NMR** (160 MHz, $CDCl_3$) δ_B: 40.16; **HRMS** (ESI)$^+$ $C_{41}H_{34}BNO_3Na$ [M+Na]$^+$ found 622.2527, requires 622.2524 (+0.47 ppm).

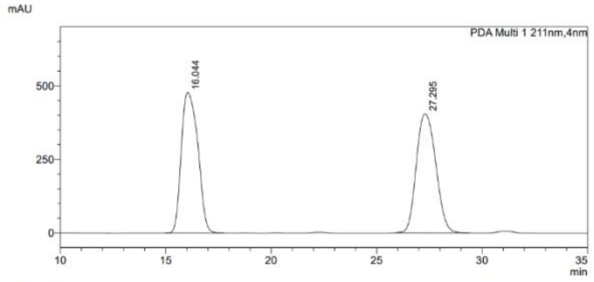

<Peak Table>

PDA Ch1 211nm

Peak#	Ret. Time	Area%
1	16.044	50.086
2	27.295	49.914
Total		100.000

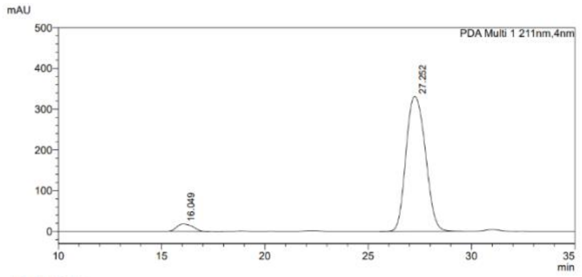

<Peak Table>

PDA Ch1 211nm

Peak#	Ret. Time	Area%
1	16.049	4.305
2	27.252	95.695
Total		100.000

The title alcohol (*R*)-1-(1-(4-methoxybenzyl)-3-methylbenzo[e][1,2]azaborinin-2(1H)-yl)naphthalen-2-ol **62** (29.4 mg, 36%): $[\alpha]_D^{20}$ +212.5 (*c* 1.3, $CHCl_3$); **Chiral HPLC analysis**, Chiralpak IA (95:5 hexane : IPA, flow rate 1.5 mL.min^{-1}, 220 nm, 30 °C) t_R (*R*): 25.7 min, t_R (*S*): 42.9 min, 88:12.

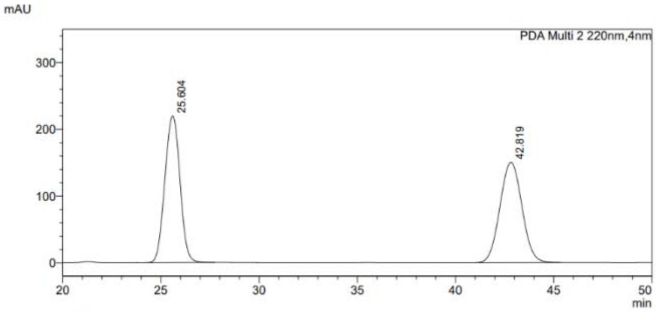

<Peak Table>

PDA Ch2 220nm

Peak#	Ret. Time	Area%
1	25.604	50.104
2	42.819	49.896
Total		100.000

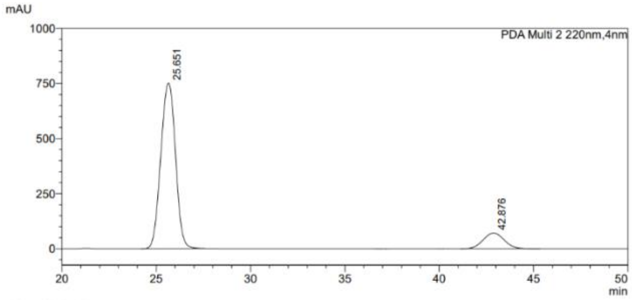

<Peak Table>

PDA Ch2 220nm

Peak#	Ret. Time	Area%
1	25.651	88.121
2	42.876	11.879
Total		100.000

(*S*)-1-(3-methyl-1-phenylbenzo[*e*][1,2]azaborinin-2(1*H*)-yl)naphthalen-2-yl 2,2 diphenylacetate **65**

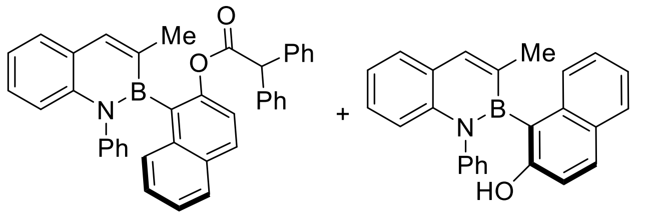

c = 10
s = 3

Following general procedure **E**, 1-(3-methyl-1-phenylbenzo[*e*][1,2]azaborinin-2(1*H*)-yl)naphthalen-2-ol **64**, (108 mg, 0.30 mmol), 2,2-diphenylacetic pivalic anhydride **13** (62.2 mg, 0.21 mmol), (*R*)-BTM **14** (3.8 mg, 0.015 mmol), Et_3N (25.2 µL, 0.18 mmol) in $CHCl_3$ (3 mL) for 16 h gave, after purification on boron capped silica by Biotage® Selekt™ (Sfär HC 25 g, 80 mL.min^{-1}, Petrol : EtOAc [100:0 to 95:5 8 CV, 95:5 to 90:10 8 CV, 90:10 to 75:25 8 CV]) the title ester **65** (21.7 mg, 13%) as a white amorphous solid. $[\alpha]_D^{20}$ –2.3 (*c* 1.3, $CHCl_3$); **Chiral HPLC analysis**, Chiralpak IC (99:1 hexane : IPA, flow rate 1.5 mL.min^{-1}, 254 nm, 30 °C) t_R (*S*): 5.5 min, t_R (*R*): 7.6 min, 74:26 er; **IR** ν_{max} (film) 3061, 3028, 1749, 1597, 1493, 1190, 1117, 908; **^{1}H NMR** (400 MHz, $CDCl_3$) δ_H: 2.00 (3H, d, *J* 1.3), 5.11 (1H, s), 6.36 (1H, dt, *J* 7.7, 1.9), 6.68 (1H, dd, *J* 7.8, 1.8), 6.79 (1H, dt, *J* 7.9, 1.7), 6.87 (1H, td, *J* 7.5, 1.8), 6.96–7.08 (9H, m), 7.11 (2H, app d, *J* 7.0), 7.16 (2H, app d, *J* 7.0), 7.27–7.39 (4H, m), 7.50 (1H, dd, *J* 7.8, 1.6), 7.63 (1H, d, *J* 8.9), 7.73 (1H, dd, *J* 7.7, 1.6), 7.77–7.79 (1H, m), 7.92 (1H, s); **^{13}C{^{1}H} NMR** (126 MHz, $CDCl_3$) δ_C: 21.6, 57.7, 117.8, 120.7, 121.6, 125.1, 125.9, 126.7, 126.8, 127.1, 127.2, 127.4, 127.4, 128.2, 128.2, 128.3, 128.5, 128.5, 128.6, 128.7, 128.7, 129.0, 129.1, 131.1, 135.7, 137.9, 138.2, 140.4 (br s), 141.96, 142.03, 143.7, 148.9, 171.2; **^{11}B{^{1}H} NMR** (160 MHz, $CDCl_3$) δ_B: 37.55 (br s); **HRMS** (ESI)$^+$ $C_{39}H_{30}BNO_2$ [M+Na]$^+$ found 578.2259, requires 578.2262 (–0.46 ppm).

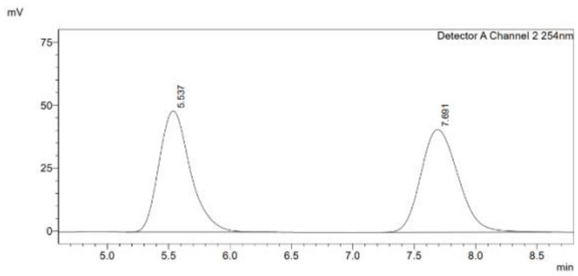

<Peak Table>

Detector A Channel 2 254nm

Peak#	Ret. Time	Area%
1	5.537	49.814
2	7.691	50.186
Total		100.000

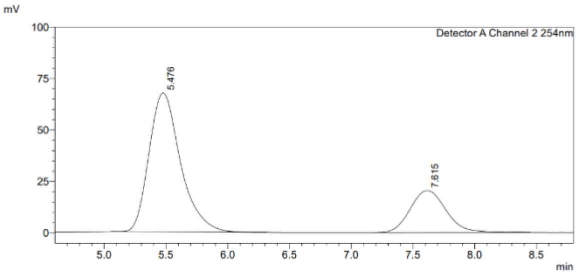

<Peak Table>

Detector A Channel 2 254nm

Peak#	Ret. Time	Area%
1	5.476	74.404
2	7.615	25.596
Total		100.000

The title alcohol (*R*)-1-(3-methyl-1-phenylbenzo[e][1,2]azaborinin-2(1H)-yl)naphthalen-2-ol **64** (83.5 mg, 77%): $[\alpha]_D^{20}$ –7.3 (*c* 1.6, $CHCl_3$); **Chiral HPLC analysis**, Chiralpak IA (95:5

hexane : IPA, flow rate 1.5 mL.min^{-1}, 220 nm, 30 °C) t_R (*R*): 10.9 min, t_R (*S*): 17.7 min, 53:47 er.

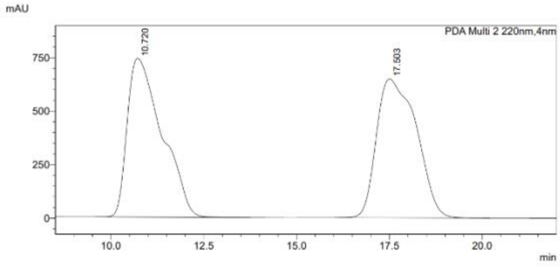

<Peak Table>

PDA Ch2 220nm

Peak#	Ret. Time	Area%
1	10.720	49.909
2	17.503	50.091
Total		100.000

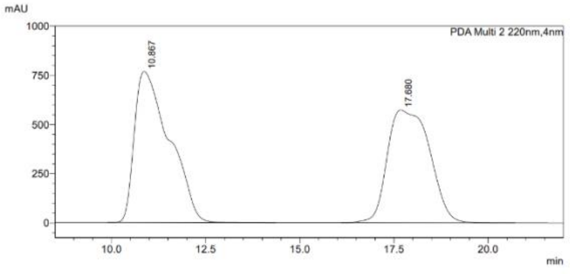

<Peak Table>

PDA Ch2 220nm

Peak#	Ret. Time	Area%
1	10.867	52.564
2	17.680	47.436
Total		100.000

(*S*)-1-(3-bromo-1-(cyclohexylmethyl)benzo[e][1,2]azaborinin-2(1H)-yl)naphthalen-2-yl 2,2-diphenylacetate **67**

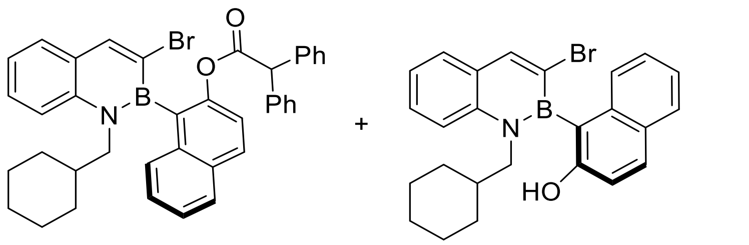

c = 2
s = 28

Following general procedure **E**, 1-(3-bromo-1-(cyclohexylmethyl)benzo[e][1,2]azaborinin-2(1H)-yl)naphthalen-2-ol **66** (134 mg, 0.30 mmol), 2,2-diphenylacetic pivalic anhydride **13** (62.2 mg, 0.21 mmol), (*R*)-BTM **14** (1.8 mg, 0.015 mmol), Et_3N (25.2 µL, 0.18 mmol) in $CHCl_3$ (3 mL) for 16 h gave, after purification by Biotage® Selekt™ (Sfär HC 25 g, 80 mL.min^{-1}, Petrol : EtOAc [100:0 to 90:10 CV, 90:10 to 75:25 10 CV]), the title ester **67** (2.3 mg, 1%) as a white amorphous solid. $[\alpha]_D^{20}$ +47.8 (*c* 0.1, $CHCl_3$); **Chiral HPLC analysis**, Chiralcel OD-H (98:2 hexane : IPA, flow rate 1.0 mL.min^{-1}, 254 nm, 30 °C) t_R (*S*): 6.5 min, t_R (*R*): 7.4 min, 96:4 er; **IR** ν_{max} (film) 2924, 1751, 1589, 1354, 1223, 908; **^{1}H NMR** (400 MHz, $CDCl_3$) δ_H: 0.33–0.36 (1H, m), 0.56 (1H, q, *J* 10.9), 0.85–0.91 (3H, m), 1.27–1.40 (5H, m), 1.60–1.69 (1H, m), 3.47 (1H, dd, *J* 14.1, 6.3), 3.73 (1H, dd, *J* 14.2, 7.1), 5.03 (1H, s), 7.05–7.10 (6H, m), 7.18–7.21 (4H, m), 7.32–7.46 (6H, m), 7.57 (1H, ddd, *J* 8.7, 7.0, 1.7), 7.69 (1H, dd, *J* 7.9, 1.7), 7.88–7.93 (2H, m), 8.31 (1H, s); **^{13}C{^{1}H} NMR** (126 MHz, $CDCl_3$) δ_C: 25.7, 25.9, 26.1, 30.8, 31.1, 37.0, 56.0, 57.5, 117.0, 121.0, 121.9, 125.5, 126.2, 127.2, 127.2, 127.3, 128.5, 128.6, 128.6, 128.7, 129.9, 130.0, 131.4, 135.7, 137.9, 138.1, 140.6, 145.7, 150.0, 170.5; **^{11}B{^{1}H} NMR** (160 MHz, $CDCl_3$) δ_B: 36.79 (br s); **HRMS** (ESI)$^+$ $C_{39}H_{35}B^{79}BrNO_2Na$ [M+Na]$^+$ found 662.1839, requires 662.1836 (+0.45 ppm).

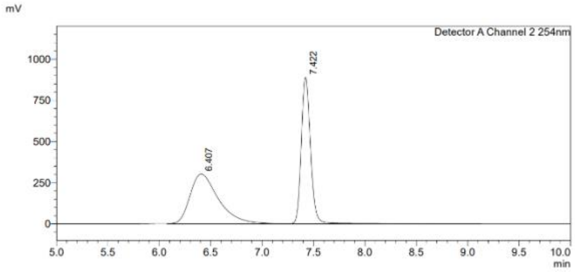

<Peak Table>

Detector A Channel 2 254nm

Peak#	Ret. Time	Area%
1	6.407	50.932
2	7.422	49.068
Total		100.000

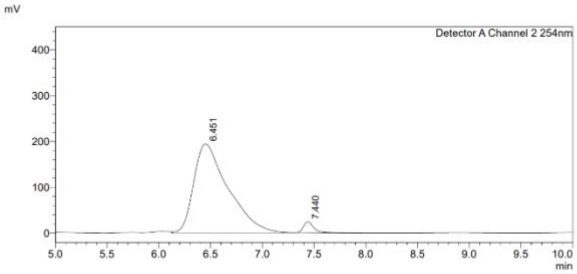

<Peak Table>

Detector A Channel 2 254nm

Peak#	Ret. Time	Area%
1	6.451	96.429
2	7.440	3.571
Total		100.000

The title alcohol (*R*)-1-(3-bromo-1-(cyclohexylmethyl)benzo[e][1,2]azaborinin-2(1H)-yl)naphthalen-2-ol **66** (110 mg, 82%): **Chiral HPLC analysis**, Chiralcel OD-H (99:1 hexane : IPA, flow rate 1.0 mL.min^{-1}, 211 nm, 30 °C) t_R (*R*): 9.5 min, t_R (*S*): 16.1 min, 51:49 er.

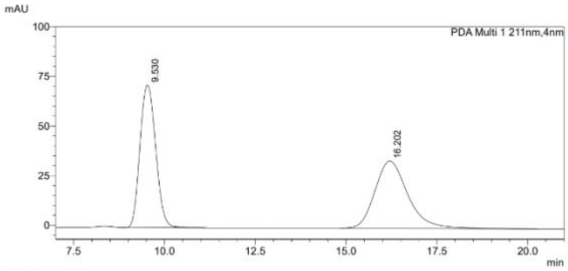

<Peak Table>

PDA Ch1 211nm

Peak#	Ret. Time	Area%
1	9.530	50.051
2	16.202	49.949
Total		100.000

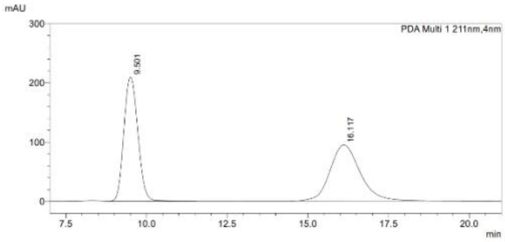

<Peak Table>

PDA Ch1 211nm

Peak#	Ret. Time	Area%
1	9.501	50.989
2	16.117	49.011
Total		100.000

(*S*)-4-(1-benzyl-3-methylbenzo[*e*][1,2]azaborinin-2(1*H*)-yl)naphthalen-2-yl 2,2 diphenylacetate **69**

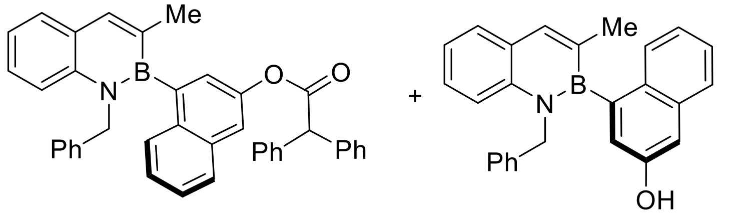

c = 56
s = 2

Following general procedure **E**, 4-(1-benzyl-3-methylbenzo[*e*][1,2]azaborinin-2(1*H*)-yl)naphthalen-2-ol **68** (75.1 mg, 0.20 mmol), 2,2-diphenylacetic pivalic anhydride **13** (41.5 mg, 0.14 mmol), (*R*)-BTM **14** (2.5 mg, 0.010 mmol), Et_3N (16.7 μL, 0.12 mmol) in $CHCl_3$ (2 mL) for 16 h gave, after purification on boron capped silica by Biotage® Selekt™ (Sfär HC 25 g, 80 mL.min^{-1}, Petrol : EtOAc [100:0 to 90:10 10 CV, 90:10 to 75:25 10 CV, 75:25 to 50:50 10 CV]), the title ester **69** (114 mg, 46%) as a white amorphous solid. $[\alpha]_D^{20}$ –8.2 (*c* 1.0, $CHCl_3$); **Chiral HPLC analysis**, Chiralpak IA (99.5:0.5 hexane : IPA, flow rate 1.0 mL.min^{-1}, 220 nm, 30 °C) t_R (*R*): 16.3 min, t_R (*S*): 24.8 min, 41:59 er; **IR** ν_{max} (film) 3030, 2932, 1757, 1495, 1206, 1126, 907; **^{1}H NMR** (400 MHz, $CDCl_3$) δ_H: 1.98 (3H, d, *J* 1.3), 5.14 (1H, d, *J* 16.7), 5.262 (1H, s), 5.264 (1H, d, *J* 16.7), 6.98 (2H, d, *J* 6.5), 7.11 (1H, d, *J* 2.3), 7.13–7.19 (3H, m), 7.23 (1H, ddd, *J* 8.0, 6.7, 1.4), 7.29–7.40 (13H, m), 7.44 (1H, ddd, *J* 8.2, 6.8, 1.2), 7.52 (1H, d, *J* 2.3), 7.59 (1H, dd, *J* 8.3, 1.1), 7.71 (1H, dd, *J* 7.7, 1.6), 7.80 (1H, d, *J* 7.6), 7.92 (1H, s); **^{13}C{^{1}H} NMR** (101 MHz, $CDCl_3$) δ_C: 22.0, 53.1, 57.3, 116.9, 117.9, 121.5, 123.4, 125.5, 125.9, 126.3, 126.8, 127.5, 127.6, 127.6, 128.5, 128.6, 128.8, 128.8, 128.8, 128.9, 128.9, 129.6, 133.1, 133.9, 138.4, 138.4, 138.8, 140.1, 140.6 (br s), 142.4, 143.2 (br s), 148.2, 171.1; **^{11}B{^{1}H} NMR** (128 MHz, $CDCl_3$) δ_B: 41.79 (br s); **HRMS** (ESI$^+$) $C_{40}H_{32}BNO_2Na$ [M+Na]$^+$ found 592.2422, requires 592.2418 (+0.61 ppm).

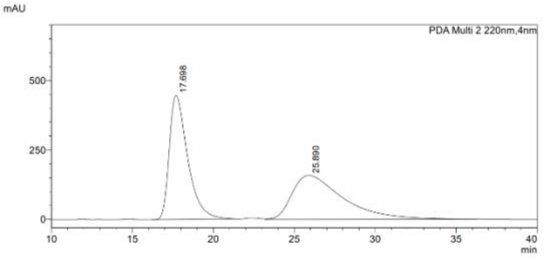

<Peak Table>

PDA Ch2 220nm

Peak#	Ret. Time	Area%
1	17.698	50.279
2	25.890	49.721
Total		100.000

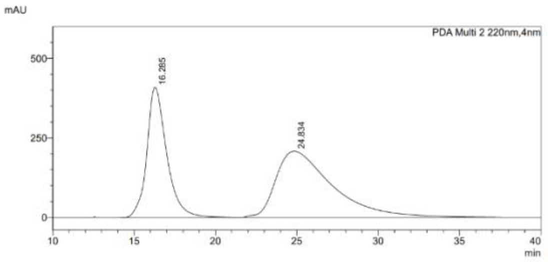

<Peak Table>

PDA Ch2 220nm

Peak#	Ret. Time	Area%
1	16.285	41.007
2	24.834	58.993
Total		100.000

The title alcohol (*R*)-4-(1-benzyl-3-bromobenzo[e][1,2]azaborinin-2(1H)-yl)naphthalen-2-ol **68** (38.3 mg, 51%): $[\alpha]_D^{20}$ +35.2 (*c* 1.8, $CHCl_3$); **Chiral HPLC analysis**, Chiralpak IA (90:10 hexane : IPA, flow rate 1.0 mL.min^{-1}, 220 nm, 30 °C) t_R (*R*): 34.3 min, t_R (*S*): 44.3 min, 62:38 er.

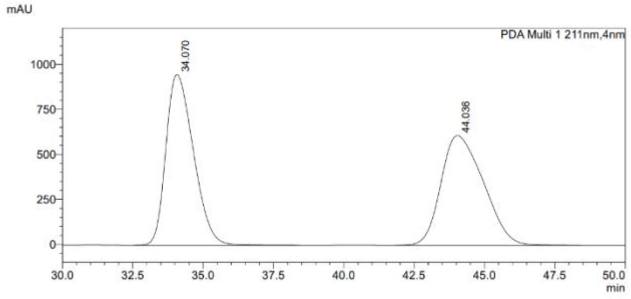

<Peak Table>

PDA Ch1 211nm

Peak#	Ret. Time	Area%
1	34.070	50.258
2	44.036	49.742
Total		100.000

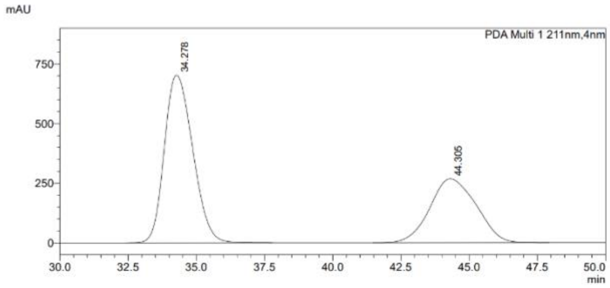

<Peak Table>

PDA Ch1 211nm

Peak#	Ret. Time	Area%
1	34.278	61.633
2	44.305	38.367
Total		100.000

7.2. Racemisations

(±)-1-(1-benzyl-3-methylbenzo[e][1,2]azaborinin-2(1H)-yl)naphthalen-2-ol **15**

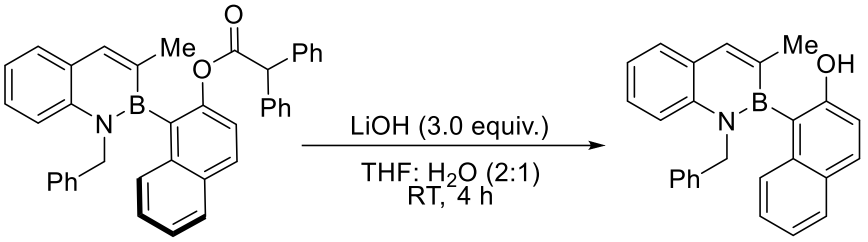

96:4 er

(*R*)-1-(1-benzyl-3-methylbenzo[e][1,2]azaborinin-2(1H)-yl)naphthalen-2-yl2,2-diphenylacetate **16** (95:5 er, 0.17 g, 0.30 mmol, 1.0 equiv.) and LiOH (38 mg, 0.90 mmol, 3.0 equiv.) in THF (0.50 mL) and H_2O (0.25 mL) were stirred at RT for 4 h. The mixture was then diluted with aq. HCl (1.0 M) and concentrated under reduced pressure to remove THF. The crude was extracted with EtOAc, dried (Na_2SO_4) and concentrated under reduced pressure to afford the crude product which was purified by Biotage® Selekt™ (Sfär HC 25 g, 80 mL.min^{-1}, Petrol : EtOAc [100:0 to 90:10 10 CV, 90:10 to 75:25 10 CV]) to give the title compound **15** (82 mg, 73%), as a white amorphous solid. $[\alpha]_D^{20}$ –8.9 (*c* 4.4, $CHCl_3$); **Chiral HPLC analysis**, Chiralpak IA (95:5 hexane : IPA, flow rate 1.0 mL.min^{-1}, 220 nm, 30 °C) t_R (*S*): 20.8 min, t_R (*R*): 24.3 min, 48:52 er.

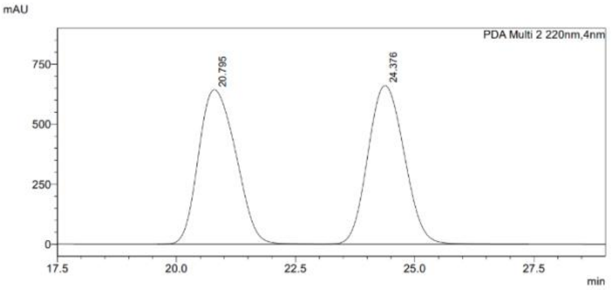

<Peak Table>

PDA Ch2 220nm

Peak#	Ret. Time	Area%
1	20.795	49.950
2	24.376	50.050
Total		100.000

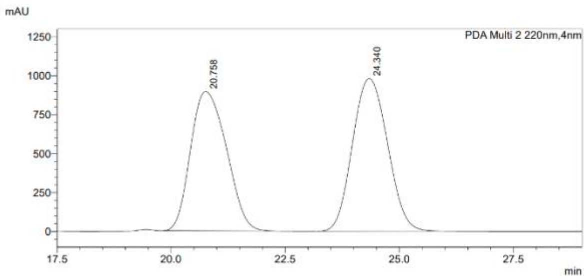

<Peak Table>

PDA Ch2 220nm

Peak#	Ret. Time	Area%
1	20.758	48.297
2	24.340	51.703
Total		100.000

(±)-1-(1-benzyl-3-methylbenzo[e][1,2]azaborinin-2(1H)-yl)naphthalen-2-ol **15**

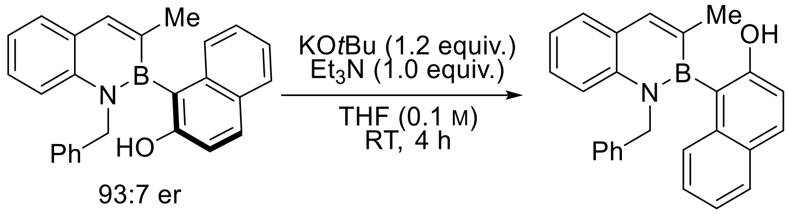

93:7 er

(*R*)-1-(1-benzyl-3-methylbenzo[e][1,2]azaborinin-2(1H)-yl)naphthalen-2-ol **15** (93:7 er, 19 mg, 0.050 mmol, 1.0 equiv.), KO*t*Bu (12% in THF, 60 µL, 0.060 mmol, 1.2 equiv.) and Et_3N (7.0 µL, 0.50 mmol, 1.0 equiv.) were stirred in THF (0.5 mL) at RT for 4 h. The mixture was diluted with aq. NH_4Cl and aq. HCl (1.0 M), then extracted with EtOAc. The organic layers were combined, dried (Na_2SO_4) and concentrated under reduced pressure to afford the crude product which was purified by Biotage® Selekt™ (Sfär HC 10 g, 60 mL.min^{-1}, Petrol : EtOAc [95:5 to 85:15 10 CV, 85:15 to 75:25 5 CV]), to give the title compound **15** (15 mg, 80%) as a white amorphous solid. **Chiral HPLC analysis**, Chiralpak IA (95:5 hexane : IPA, flow rate 1.0 mL.min^{-1}, 220 nm, 30 °C) t_R (*R*): 21.0 min, t_R (*S*): 24.5 min, 52:48 er.

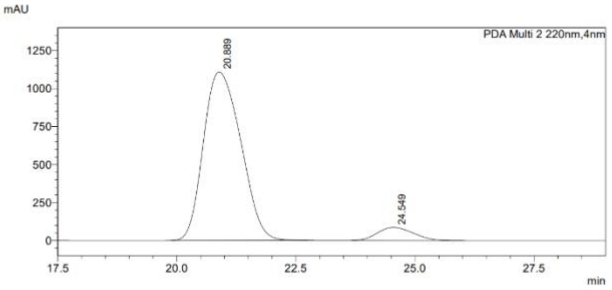

<Peak Table>

PDA Ch2 220nm

Peak#	Ret. Time	Area%
1	20.889	93.092
2	24.549	6.908
Total		100.000

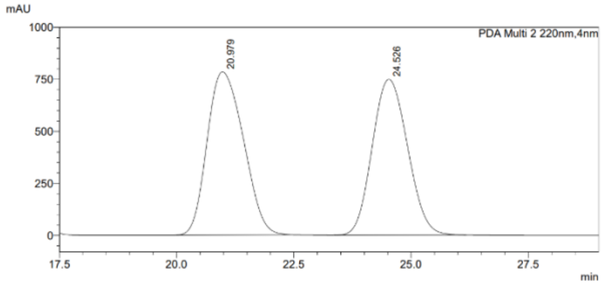

<Peak Table>

PDA Ch2 220nm

Peak#	Ret. Time	Area%
1	20.979	51.811
2	24.526	48.189
Total		100.000

7.3. Homologated alcohol substrate

(*S*)-(1-(1-benzyl-3-methylbenzo[e][1,2]azaborinin-2(1H)-yl)naphthalen-2-yl)methyl 2,2-diphenylacetate **72**

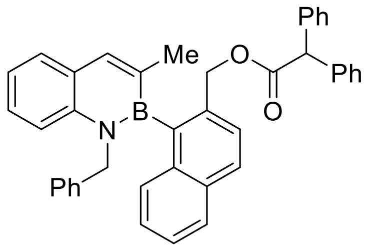

1-(1-benzyl-3-methylbenzo[e][1,2]azaborinin-2(1H)-yl)naphthalen-2-yl)methanol **70** (117 mg, 0.30 mmol, 1.0 equiv.), 2,2-diphenylacetic pivalic anhydride **13** (97.8 mg, 0.33 mmol, 1.1 equiv.), Et_3N (46.0 µL, 0.33 mmol, 1.1 equiv.) and (2*S*,3*R*)-HyperSe **71** (10.6 mg, 0.030 mmol, 0.01 equiv.) in $CHCl_3$ (3 mL) at RT for 3 h gave, after purification by Biotage® Selekt™ (Sfär HC, 25 g, 80 mL.min^{-1}, Petrol : EtOAc [100:0 to 90:10 10 CV, 90:10 to 75:25 10 CV]), the title compound **72** (101 mg, 58%) as an off-white amorphous solid. $[\alpha]_D^{20}$ +2.3 (*c* 0.9, $CHCl_3$); **Chiral HPLC analysis**, Chiralcel OD-H (95:5 hexane : IPA, flow rate 1.0 mL.min^{-1}, 270 nm, 30 °C) t_R (*R*): 11.7 min, t_R (*S*): 12.4 min, 42:58 er; **IR** ν_{max} (film) 3061, 2916, 1736, 1452, 1225, 1144, 908; **^{1}H NMR** (400 MHz, $CDCl_3$) δ_H: 1.92 (3H, d, *J* 1.4), 4.86 (1H, s), 4.97 (1H, d, *J* 16.8), 5.03 (2H, d, *J* 1.0), 5.19 (1H, d, *J* 16.8), 6.84 (2H, dd, *J* 7.5, 2.1), 7.06–7.08 (3H, m), 7.15–7.17 (2H, m), 7.24–7.39 (13H, m), 7.46 (1H, ddd, *J* 8.1, 6.8, 1.2), 7.55 (1H, dd, *J* 8.3, 1.1), 7.79 (1H, dd, *J* 7.6, 1.6), 7.83 (1H, d, *J* 8.5), 7.87 (1H, dd, *J* 8.3, 1.3), 7.94 (1H, s); **^{13}C{^{1}H} NMR** (126 MHz, $CDCl_3$) δ_C: 21.8, 52.9, 56.8, 67.9, 117.0, 121.5, 125.9, 125.9, 126.7, 127.3, 127.3, 127.5, 127.6, 128.1, 128.4, 128.4, 128.5, 128.6, 128.6, 128.7, 128.7, 129.7, 132.8, 134.7, 134.9, 138.3, 138.5, 138.7, 139.8 (br s), 140.5, 140.7 (br s), 142.0, 172.3; **^{11}B{^{1}H} NMR** (160 MHz, $CDCl_3$) δ_B: 39.07 (br s); **HRMS** (ESI)$^+$ $C_{41}H_{34}BNO_2Na$ [M+Na]$^+$ found 606.2573, requires 606.2575 (–0.35 ppm).

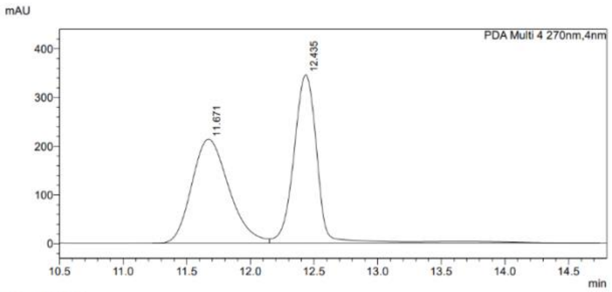

<Peak Table>

PDA Ch4 270nm

Peak#	Ret. Time	Area%
1	11.671	49.127
2	12.435	50.873
Total		100.000

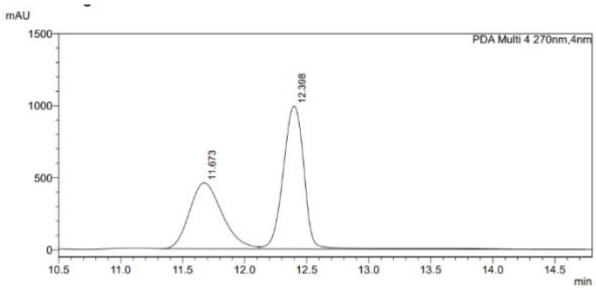

<Peak Table>

PDA Ch4 270nm

Peak#	Ret. Time	Area%
1	11.673	41.654
2	12.398	58.346
Total		100.000

(±)-1-(1-benzyl-3-methylbenzo[e][1,2]azaborinin-2(1H)-yl)naphthalen-2-yl)methanol **70** HPLC trace showing a "Batman" signal.

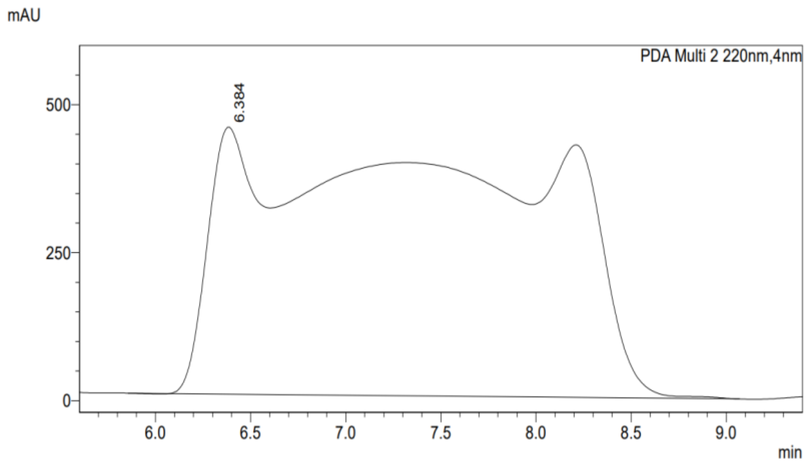

7.4. Dynamic Kinetic Resolution data

(*S*)-1-(1-benzylbenzo[e][1,2]azaborinin-2(1H)-yl)naphthalen-2-yl 2,2-diphenylacetate **25**

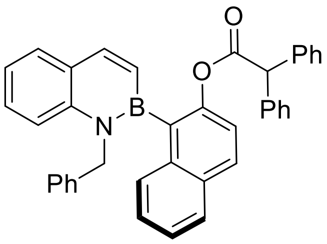

Following general procedure **F**, 1-(1-benzylbenzo[e][1,2]azaborinin-2(1H)-yl)naphthalen-2-ol **24** (57.3 mg, 0.16 mmol), 2,2-diphenylacetic pivalic anhydride **13** (47.0 mg, 0.16 mmol), (*R*)-BTM **14** (1.0 mg, 4.0 µmol), Et_3N (22.1 µL, 0.16 mmol) in $CHCl_3$ (1.6 mL) for 6.5 h gave, after purification by Biotage® Selekt™ (Sfär HC 25 g, 80 mL.min^{-1} ,Petrol : EtOAc [100:0 to 90:10 10 CV, 90:10 to 75:25 10 CV]), the title compound **25** (59.7 mg, 67%) as a white amorphous solid. $[\alpha]_D^{20}$ +3.0 (*c* 1.2, $CHCl_3$); **Chiral HPLC analysis**, Chiralpak IB (99.5:0.5 hexane : IPA, flow rate 1.0 mL.min^{-1}, 270 nm, 30 °C) t_R (*S*): 11.3 min, t_R (*R*): 15.1 min, 81:19 er.

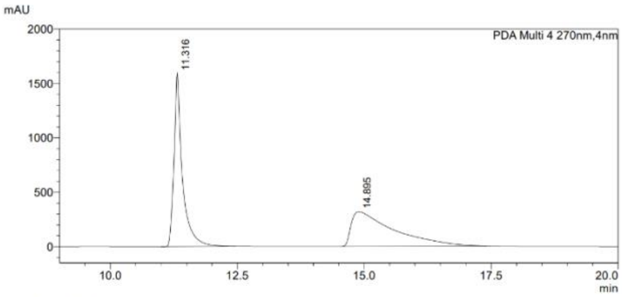

<Peak Table>

PDA Ch4 270nm

Peak#	Ret. Time	Area%
1	11.316	49.615
2	14.895	50.385
Total		100.000

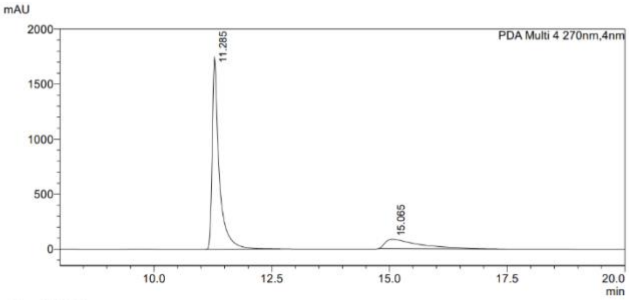

<Peak Table>

PDA Ch4 270nm

Peak#	Ret. Time	Area%
1	11.285	80.916
2	15.065	19.084
Total		100.000

(*S*)-1-(1-benzyl-4-methylbenzo[e][1,2]azaborinin-2(1H)-yl)naphthalen-2-yl 2,2 diphenylacetate **29**

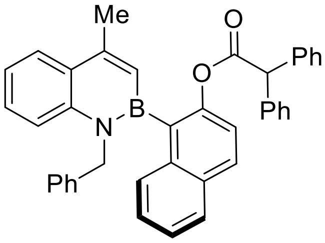

Following general procedure **F**, 1-(1-benzyl-4-methylbenzo[e][1,2]azaborinin-2(1H)-yl)naphthalen-2-ol **28** (75.1 mg, 0.20 mmol), 2,2-diphenylacetic anhydride **13** (81.3 mg, 0.2 mmol), (*R*)-BTM **14** (1.3 mg, 5.0 μmol), Et_3N (27.9 μL, 0.20 mmol) in $CHCl_3$ (2 mL) for 6.5 h, gave, after purification by Biotage® Selekt™ (Sfär HC 25 g, 80 mL.min^{-1}, Petrol : EtOAc, [100:0 to 90:10 12 CV, 90:10 to 75:25 10 CV]), the title ester **29** (69.4 mg, 61%) as a white amorphous solid. $[\alpha]_D^{20}$ +2.1 (*c* 1.3, $CHCl_3$); **Chiral HPLC analysis**, Chiralpak IA (99:1 hexane : IPA, flow rate 1.0 mL.min^{-1}, 211 nm, 30 °C) t_R (*R*): 22.6 min, t_R (*S*): 27.0 min, 28:72 er.

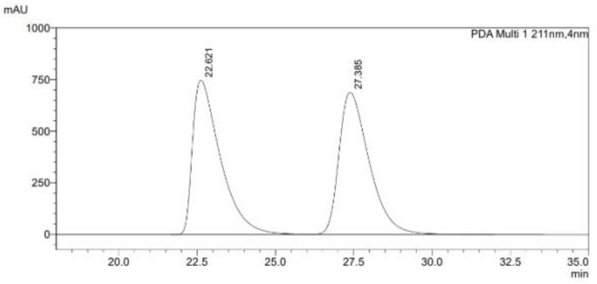

<Peak Table>

PDA Ch1 211nm

Peak#	Ret. Time	Area%
1	22.621	50.028
2	27.385	49.972
Total		100.000

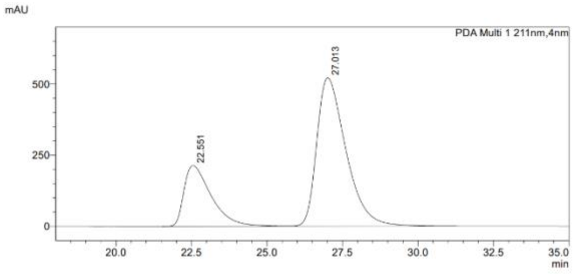

<Peak Table>

PDA Ch1 211nm

Peak#	Ret. Time	Area%
1	22.551	27.722
2	27.013	72.278
Total		100.000

The title alcohol (*R*)-1-(1-benzyl-4-methylbenzo[e][1,2]azaborinin-2(1H)-yl)naphthalen-2-ol **28** (17.4 mg, 23%): $[\alpha_D^{20}]$ +30.1 (*c* 0.8, $CHCl_3$); **Chiral HPLC analysis**, Chiralpak IA (95:5 hexane : IPA, flow rate 1.0 mL.min^{-1}, 211 nm, 30 °C) t_R (*S*):19.2 min, t_R (*R*): 31.6 min, 56:44 er.

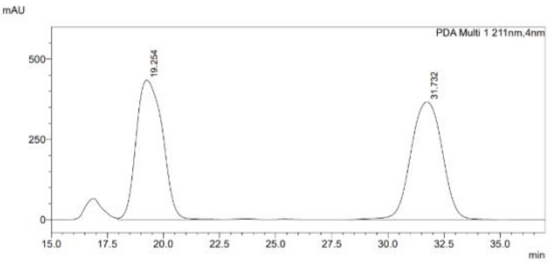

<Peak Table>

PDA Ch1 211nm

Peak#	Ret. Time	Area%
1	19.254	49.870
2	31.732	50.130
Total		100.000

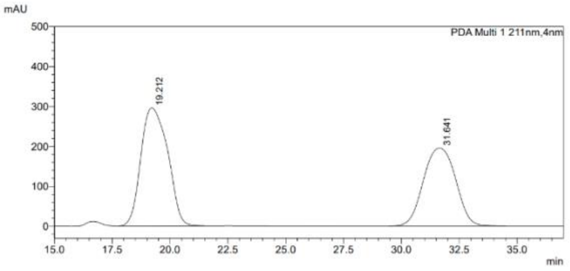

<Peak Table>

PDA Ch1 211nm

Peak#	Ret. Time	Area%
1	19.212	55.704
2	31.641	44.296
Total		100.000

(*S*)-1-(1-(cyclohexylmethyl)benzo[e][1,2]azaborinin-2(1H)-yl)naphthalen-2-yl 2,2 diphenylacetate **55**

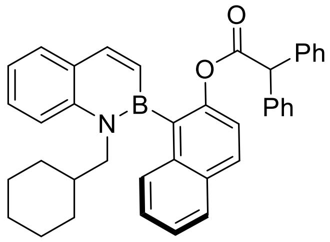

Following general procedure **F**, 1-(1-(cyclohexylmethyl)benzo[e][1,2]azaborinin-2(1H)-yl)naphthalen-2-ol **54** (57.2 mg, 0.15 mmol), 2,2-diphenylacetic pivalic anhydride **13** (44.5 mg, 0.15 mmol), (*R*)-BTM **14** (0.95, 3.8 μmol), Et_3N (20.9 μL, 0.15 mmol) in $CHCl_3$ (1.5 mL) for 6.5 h gave, after purification by Biotage® Selekt™ (Sfär HC 25 g, 80 mL.min^{-1}, Petrol : EtOAc [100:0 to 90:10 10 CV, 90:10 to 75:25 10 CV]), the title ester **55** (46.3 mg, 55%) as a white amorphous solid. $[a]_D^{20}$ +41.2 (*c* 0.9, $CHCl_3$); **Chiral HPLC analysis**, Chiralpak IA (99.5:0.5 hexane : IPA, flow rate 1.0 mL.min^{-1}, 270 nm, 30 °C) t_R (*S*): 14.5 min, t_R (*R*): 19.3 min, 88:12 er.

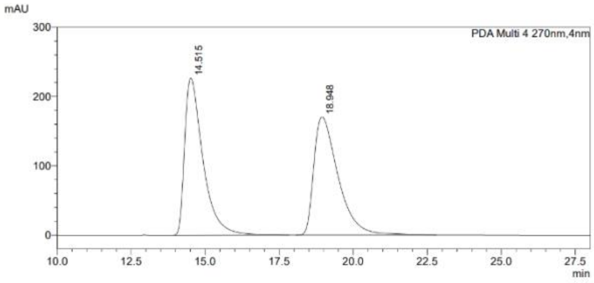

<Peak Table>

PDA Ch4 270nm

Peak#	Ret. Time	Area%
1	14.515	50.073
2	18.948	49.927
Total		100.000

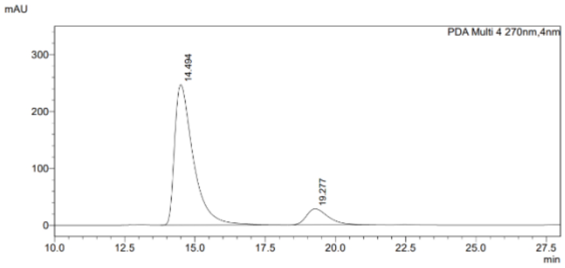

<Peak Table>

PDA Ch4 270nm

Peak#	Ret. Time	Area%
1	14.494	88.191
2	19.277	11.809
Total		100.000

8. Half-life of racemisation studies

The half-life of racemisation and ΔG‡ values of the 1,2-azaborine substrates were calculated using the following equations, with enantiomeric excess (ee) determined by HPLC analysis on a chiral stationary phase.[62] For more information regarding azaborine half life of racemisation, see references:[102, 103]

Equation 3: $$k_{rac} = gradient\ (s^{-1})$$

Equation 6: $$\Delta G^{\ddagger} = RT ln(\frac{k_B T}{k_{ent} h})$$

Equation 4: $$t_{rac}^{1/2} = \frac{ln2}{k_{rac}}$$

Equation 5: $$k_{ent} = \frac{k_{rac}}{2}$$

The general procedure for calculation of half-lives was as follows: following literature[62] the enantioenriched substrate (1 mg/mL) was dissolved in xylene and heated to a pre-determined temperature in an oil bath. After 5 mins, a t=0 time was recorded and the ee measured, subsequent time aliquots of 30-60 mins were then recorded.

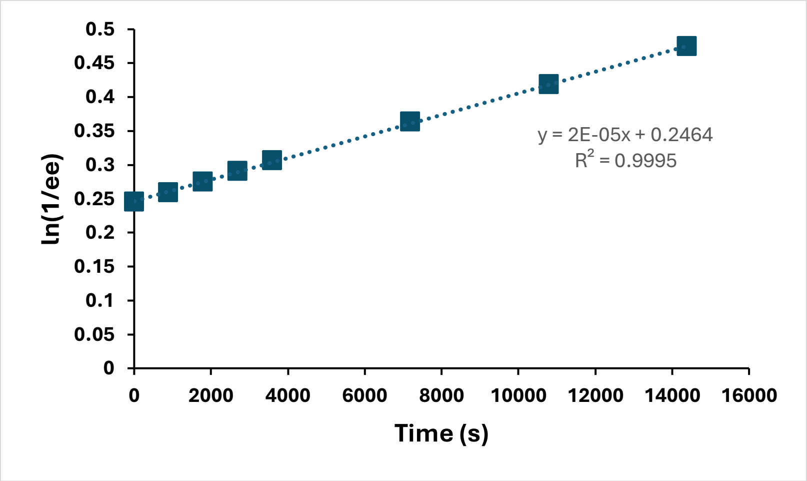

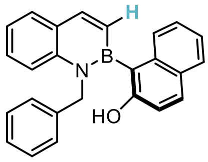

24

k_{rac} = 1.59 ×10^{-5} s^{-1}

k_{ent} = 7.95×10^{-6} s^{-1}

$t_{rac}^{1/2}$ = *12.1 hours (~5 days at RT)*

ΔG‡ = 25.8 kcal/mol at *41 °C*

*Figure 3: Graph of ln(1/ee) vs time (s) for the half life of racemisation for **24** measured at 41 ℃*

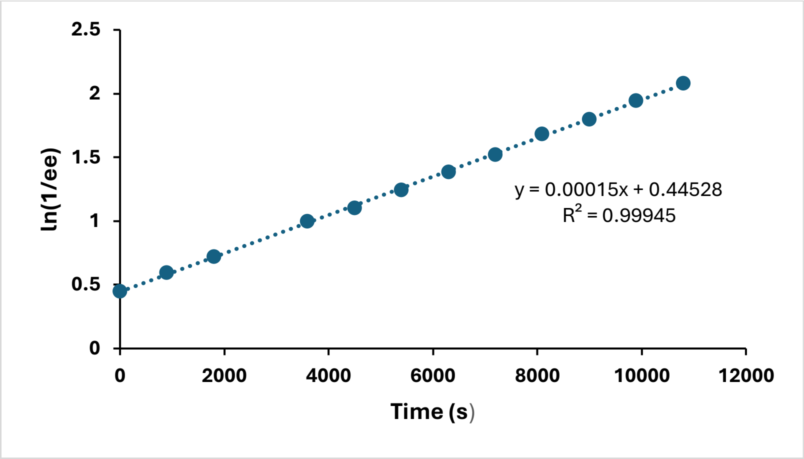

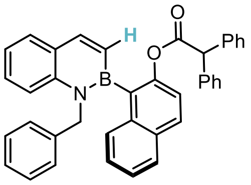

25

k_{rac} = 1.51 ×10^{-4} s^{-1}

k_{ent} = 7.53×10^{-5} s^{-1}

$t_{rac}^{1/2}$ = *1.3 hours (~ 11.5 years at RT)*

$\Delta G^{\ddagger} = 29.8$ kcal/mol *at 109 °C*

*Figure 4: Graph of ln(1/ee) vs time (s) for the half life of racemisation for **25** measured at 109 °C*

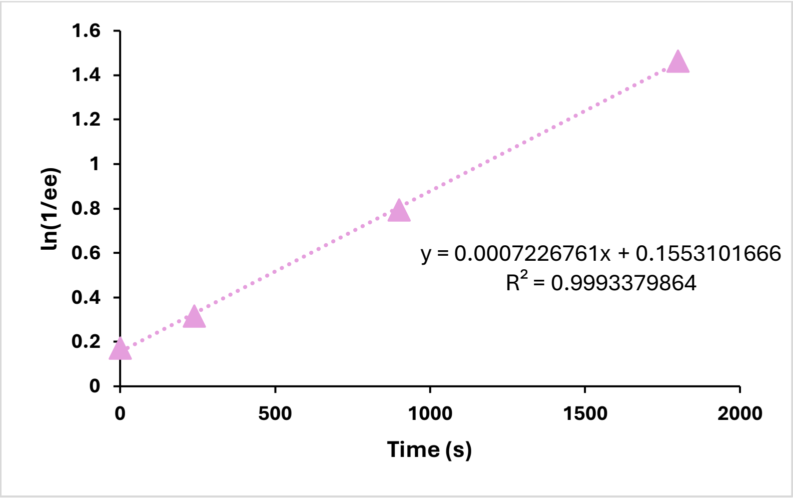

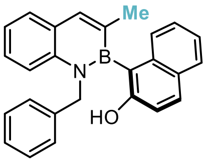

15

k_{rac} = 7.23×10^{-4} s^{-1}

k_{ent} = 3.62×10^{-4} s^{-1}

$t_{rac}^{1/2}$ = *0.27 hours (~27 years at RT)*

$\Delta G^{\ddagger} = 30.3$ kcal/mol *at 131 °C*

*Figure 5: Graph of ln(1/ee) vs time (s) for the half life of racemisation for **15** measured at 131 °C*

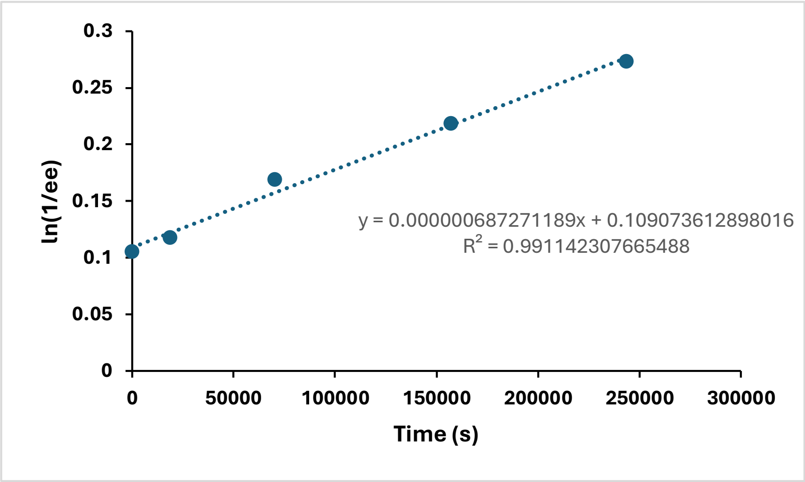

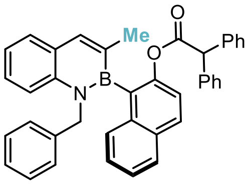

16

$k_{rac} = 6.87 \times 10^{-7}\ s^{-1}$

$k_{ent} = 3.44 \times 10^{-7}\ s^{-1}$

$t_{rac}^{1/2}$ = *280.1 hours (~103 years at RT)*

$\Delta G^{\ddagger}$ = 31.2 kcal/mol *at 80 °C*

*Figure 6: Graph of ln(1/ee) vs time (s) for the half life of racemisation for **16** measured at 80 ℃ over 4 days*

HPLC analysis software[63] used to calculate the half-life of racemisation of **70**:

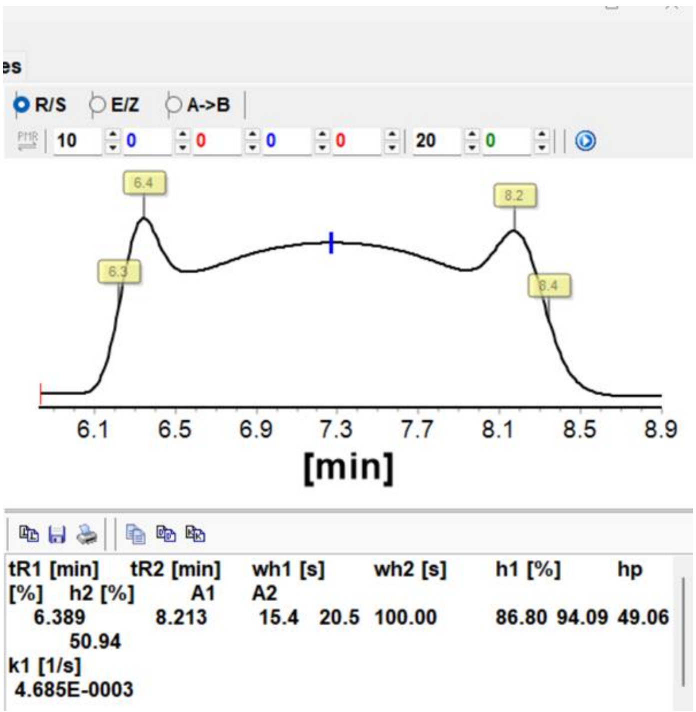

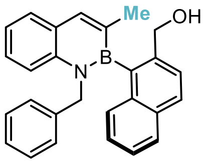

70

$k_{rac} = 9.37 \times 10^{-3}\ s^{-1}$

$k_{ent} = 4.69 \times 10^{-3}\ s^{-1}$

$t_{rac}^{1/2}$ = *1.2 mins (~2.3 mins at RT)*

$\Delta G^{\ddagger}$ = 21.0 kcal/mol *at 30 ℃*

*Figure 7: Depiction of the DCXplorerMCXVII sofware used to work out the half life of racemisation for **70** measured at 30 ℃ via chiral HPLC analysis.*

9. Crystallographic Analysis

X-ray diffraction data for compound (*R*)-**15** were collected at 100 K using a Rigaku XtaLAB Synergy-S diffractometer equipped with a HyPix-Arc 100° Hybrid Photon Counting (HPC) detector, PhotonJet-S microfocus sealed-tube X-ray source [Cu Kα radiation (λ = 1.54187 Å)], and MAX optics. Data were collected (using a calculated strategy) and processed (including correction for Lorentz, polarization and absorption) using CrysAlisPro.[104] The structure was solved by dual-space methods (SHELXT)[105] and refined by full-matrix least-squares against F^2 (SHELXL-2019/3)[106] Non-hydrogen atoms were refined anisotropically, and hydrogen atoms were refined using a riding model except for the OH hydrogen which was located from the difference Fourier map and refined isotropically subject to a distance restraint. All calculations were performed using the Olex2[107] interface. CCDC 2536174 contains the supplementary crystallographic data for this paper. These data can be obtained free of charge from The Cambridge Crystallographic Data Centre via www.ccdc.cam.ac.uk/structures.

Crystal data. $C_{26}H_{22}BNO$, M = 375.25, monoclinic, a = 8.6777(2), b = 11.3747(2), c = 10.3590(3) Å, β = 108.099(2) °, Vol. = 971.91(4) Å^3, T = 100 K, space group $P2_1$ (no. 4), Z = 2, 34250 reflections measured, 3812 unique (R_{int} = 0.0469), which were used in all calculations. The final R_1 [$I > 2\sigma(I)$] was 0.0292, wR_2 (all data) was 0.0771, and the Flack x was -0.05(9).

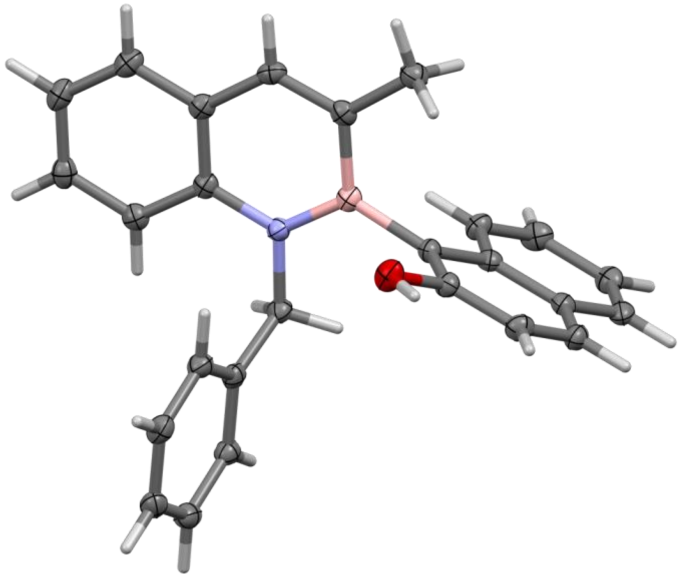

Figure 8: Thermal ellipsoid plot of the structure of 15 with ellipsoids drawn at the 50% probability level

10. NMR data

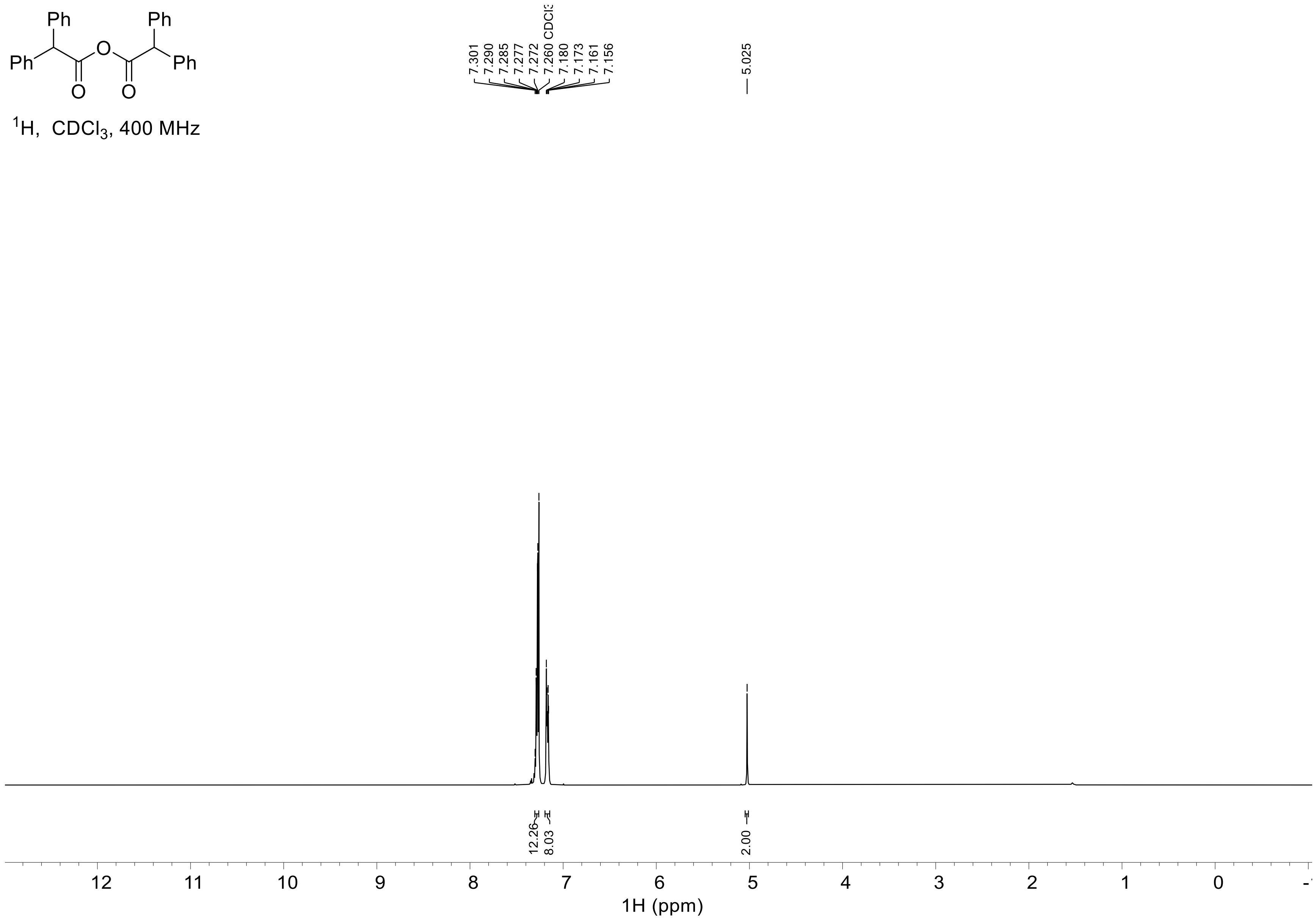
Ph
Ph
Ph
Ph
O
O
O
¹H, CDCl₃, 400 MHz
7.301
7.290
7.285
7.277
7.272
7.260 CDCl3
7.180
7.173
7.161
7.156
5.025
12.26
8.03
2.00
12
11
10
9
8
7
6
5
4
3
2
1
0
1H (ppm)

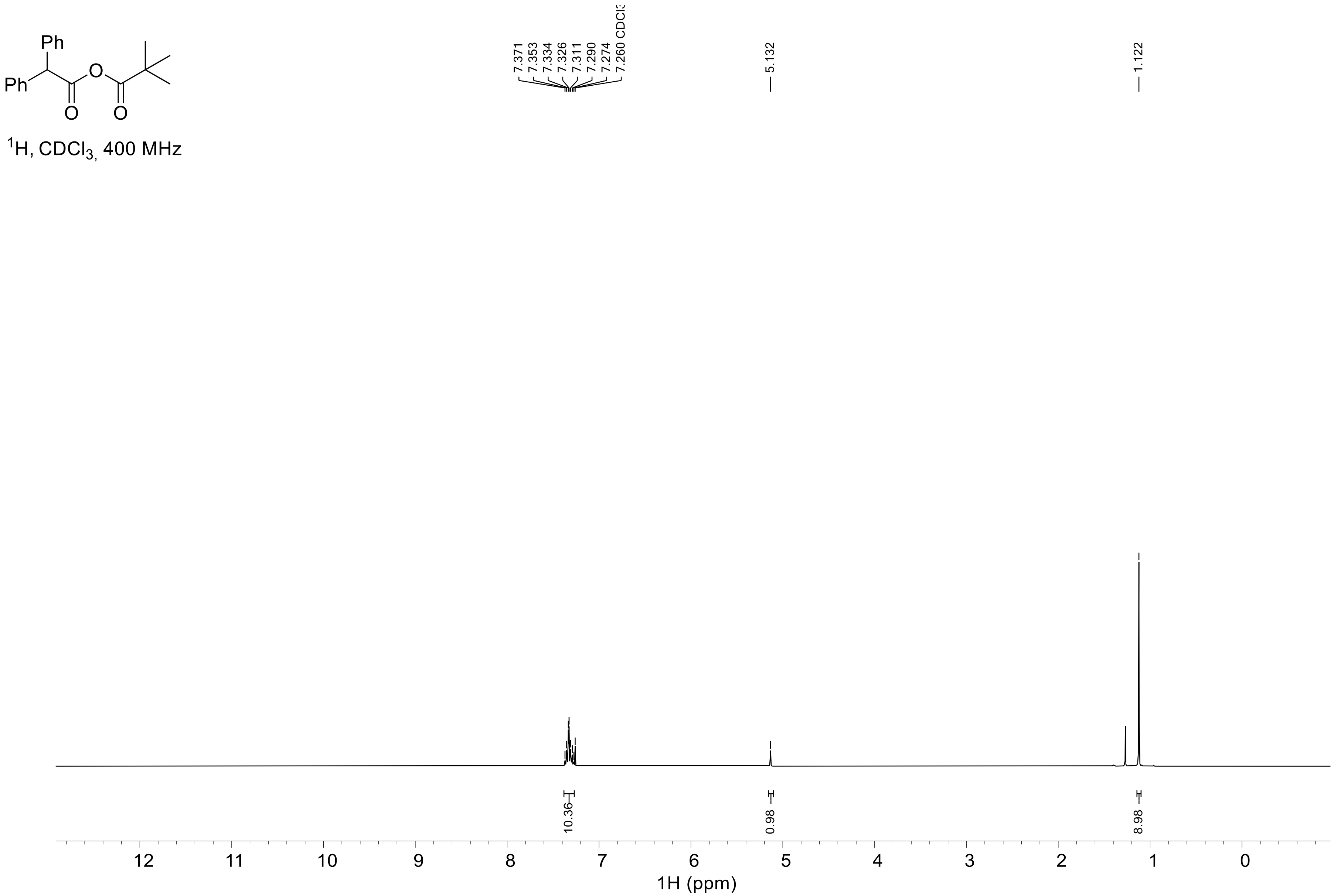
Ph
Ph
O
O
O
¹H, CDCl3, 400 MHz
7.371
7.353
7.334
7.326
7.311
7.290
7.274
7.260 CDCl3
5.132
1.122
10.36
0.98
8.98
12 11 10 9 8 7 6 5 4 3 2 1 0
1H (ppm)

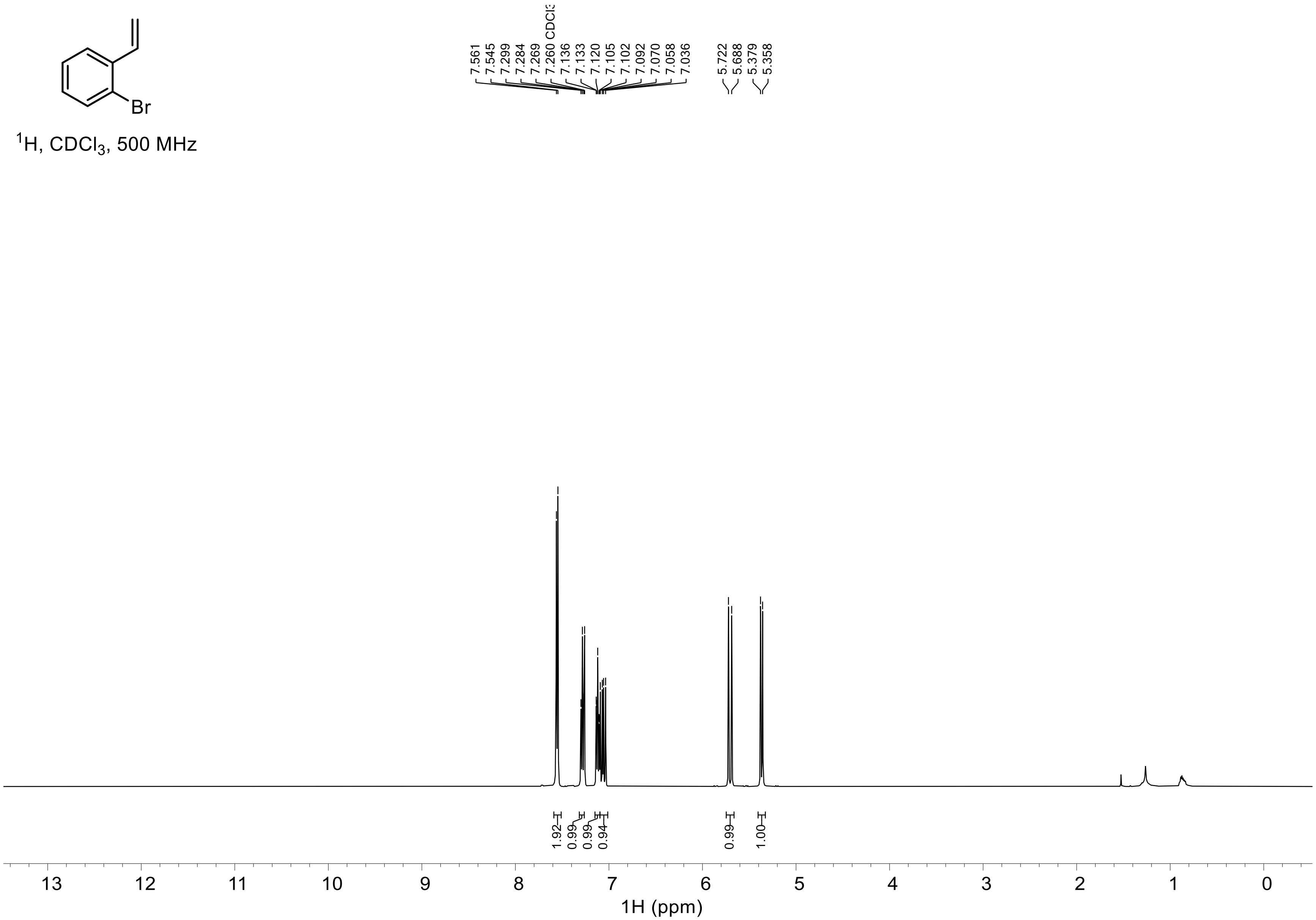

Br
1H, CDCl3, 500 MHz
7.561
7.545
7.299
7.284
7.269
7.260 CDCl3
7.136
7.133
7.120
7.105
7.102
7.092
7.070
7.058
7.036
5.722
5.688
5.379
5.358
1.92
0.99
0.99
0.94
0.99
1.00
13 12 11 10 9 8 7 6 5 4 3 2 1 0
1H (ppm)

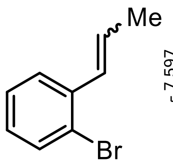

^1H, $CDCl_3$, 400 MHz

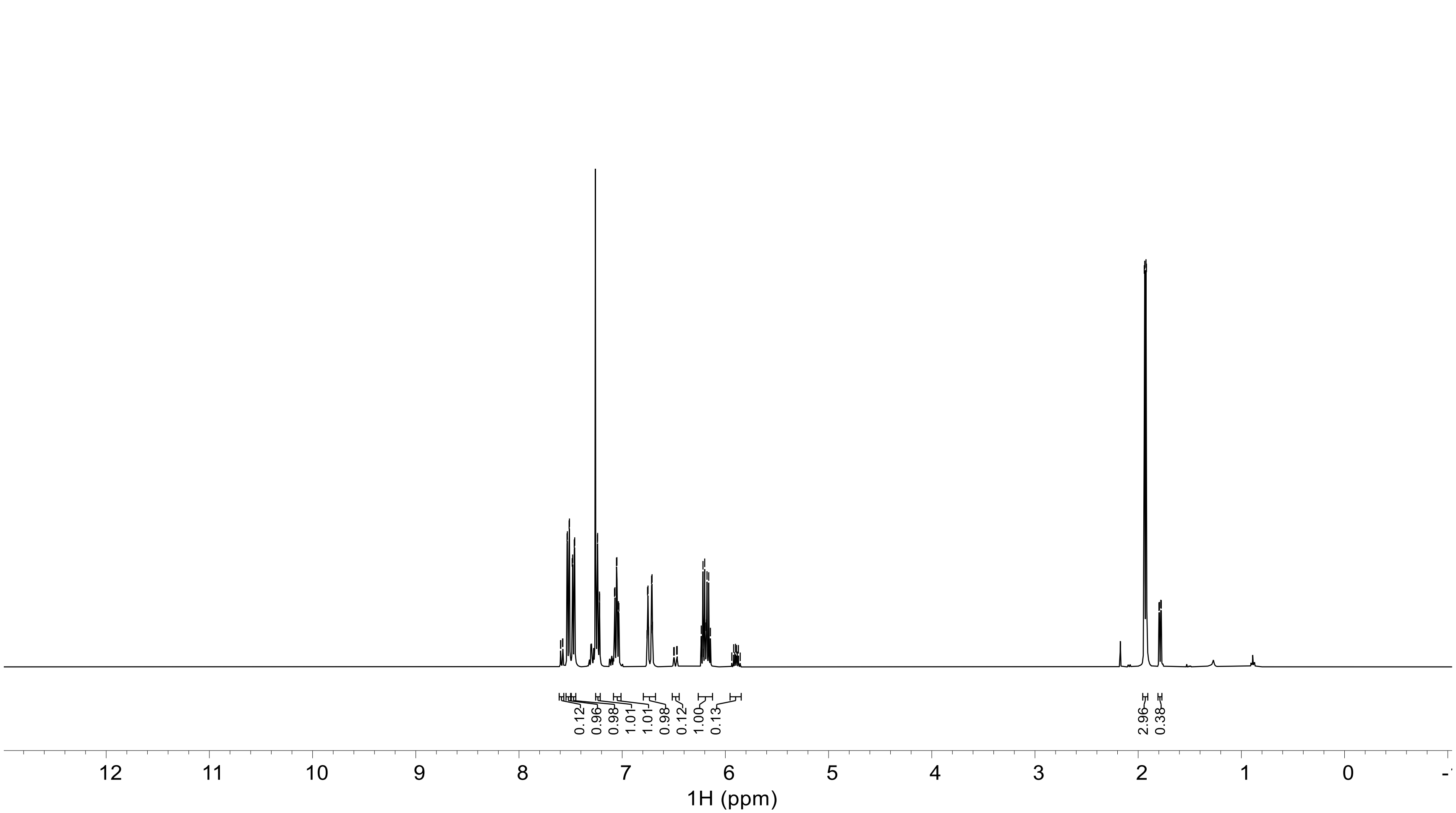

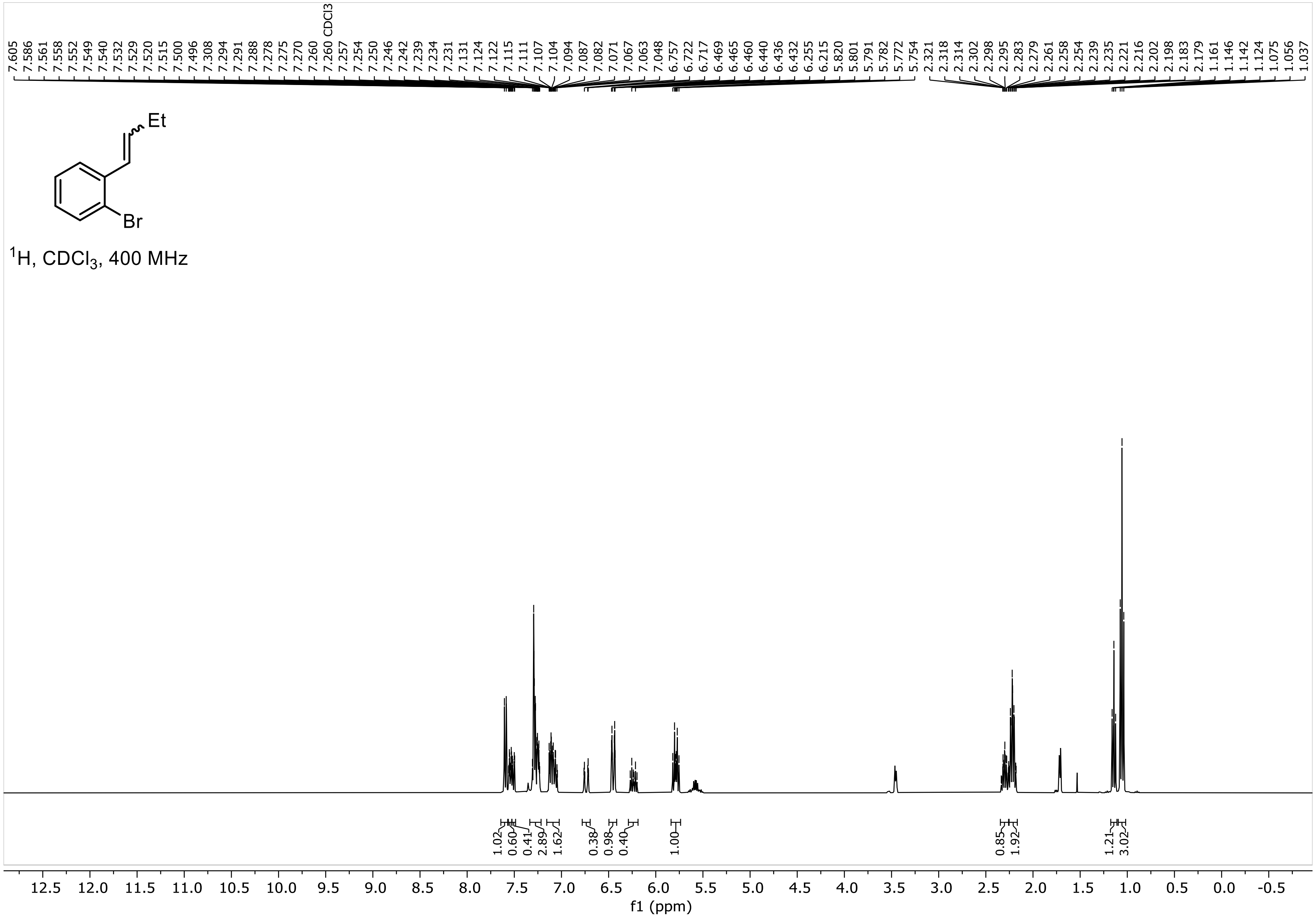
Et
Br
^{1}H, CDCl$_3$, 400 MHz
CDCl3
f1 (ppm)

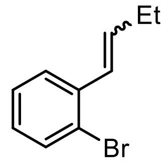

$^{13}C\ \{^1H\}$, $CDCl_3$, 101 MHz

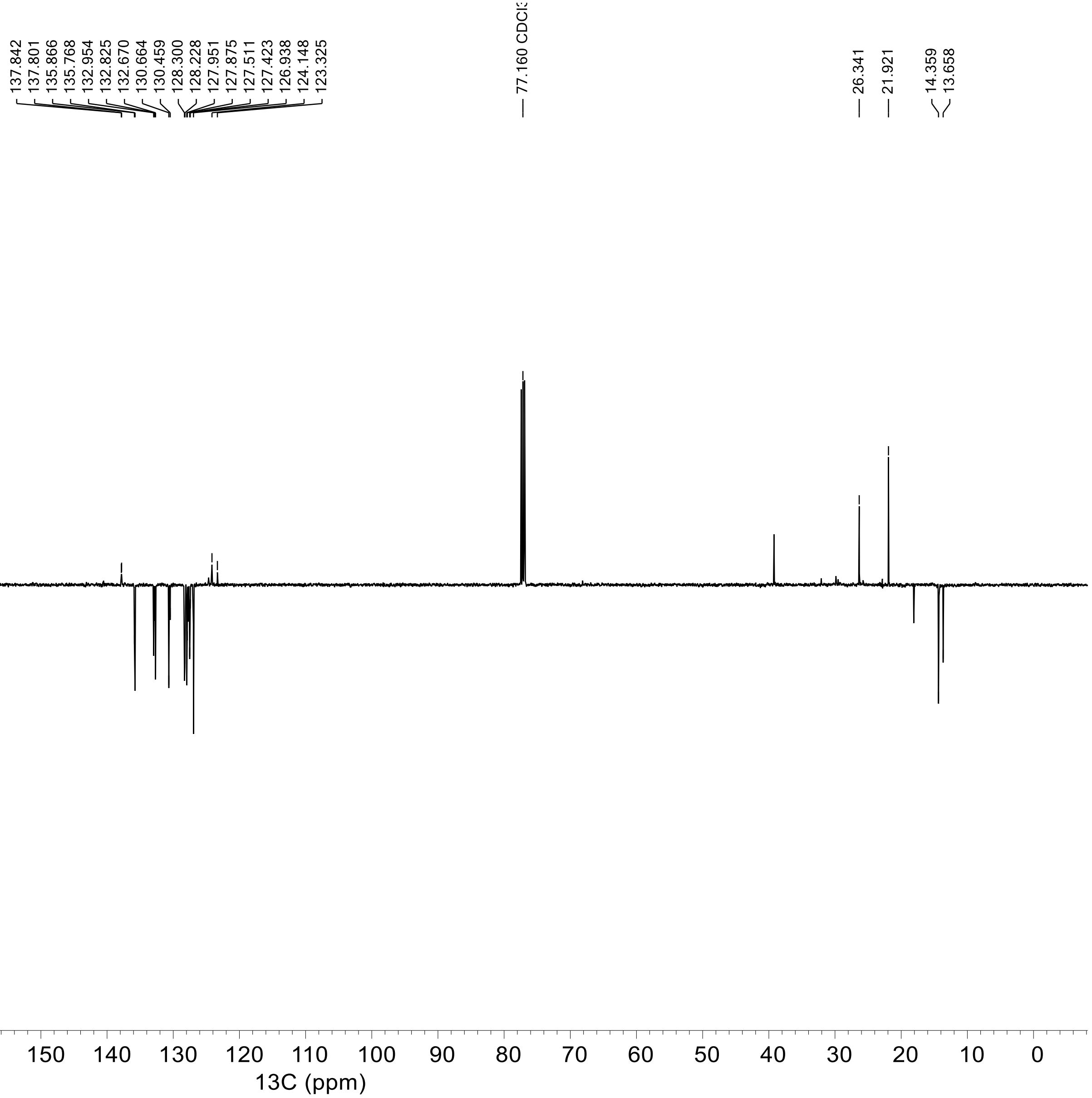

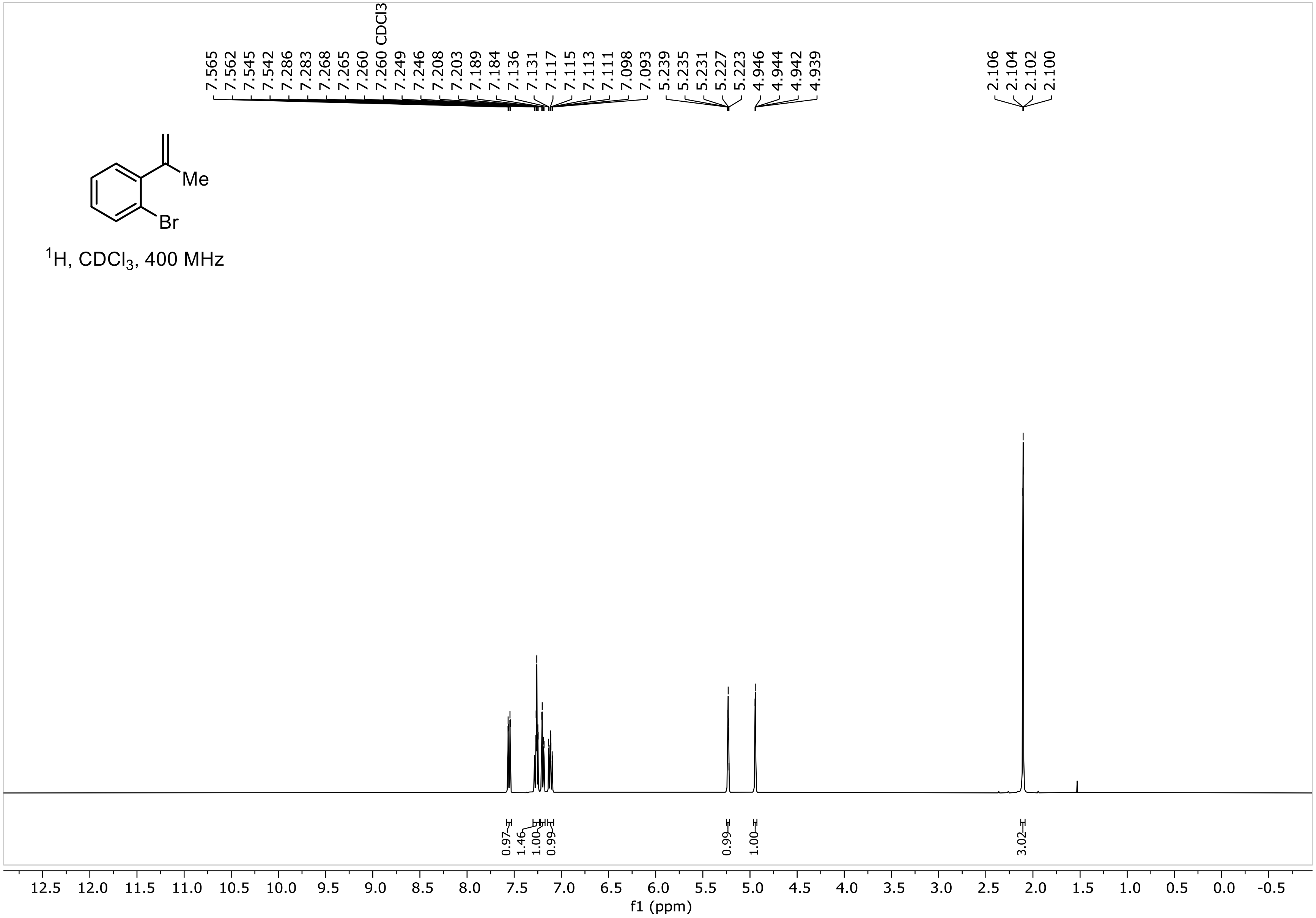

Me
Br
1H, CDCl3, 400 MHz
7.565
7.562
7.545
7.542
7.286
7.283
7.268
7.265
7.260
7.260 CDCl3
7.249
7.246
7.208
7.203
7.189
7.184
7.136
7.131
7.117
7.115
7.113
7.111
7.098
7.093
5.239
5.235
5.231
5.227
5.223
4.946
4.944
4.942
4.939
2.106
2.104
2.102
2.100
0.97
1.46
1.00
0.99
0.99
1.00
3.02
12.5 12.0 11.5 11.0 10.5 10.0 9.5 9.0 8.5 8.0 7.5 7.0 6.5 6.0 5.5 5.0 4.5 4.0 3.5 3.0 2.5 2.0 1.5 1.0 0.5 0.0 -0.5
f1 (ppm)

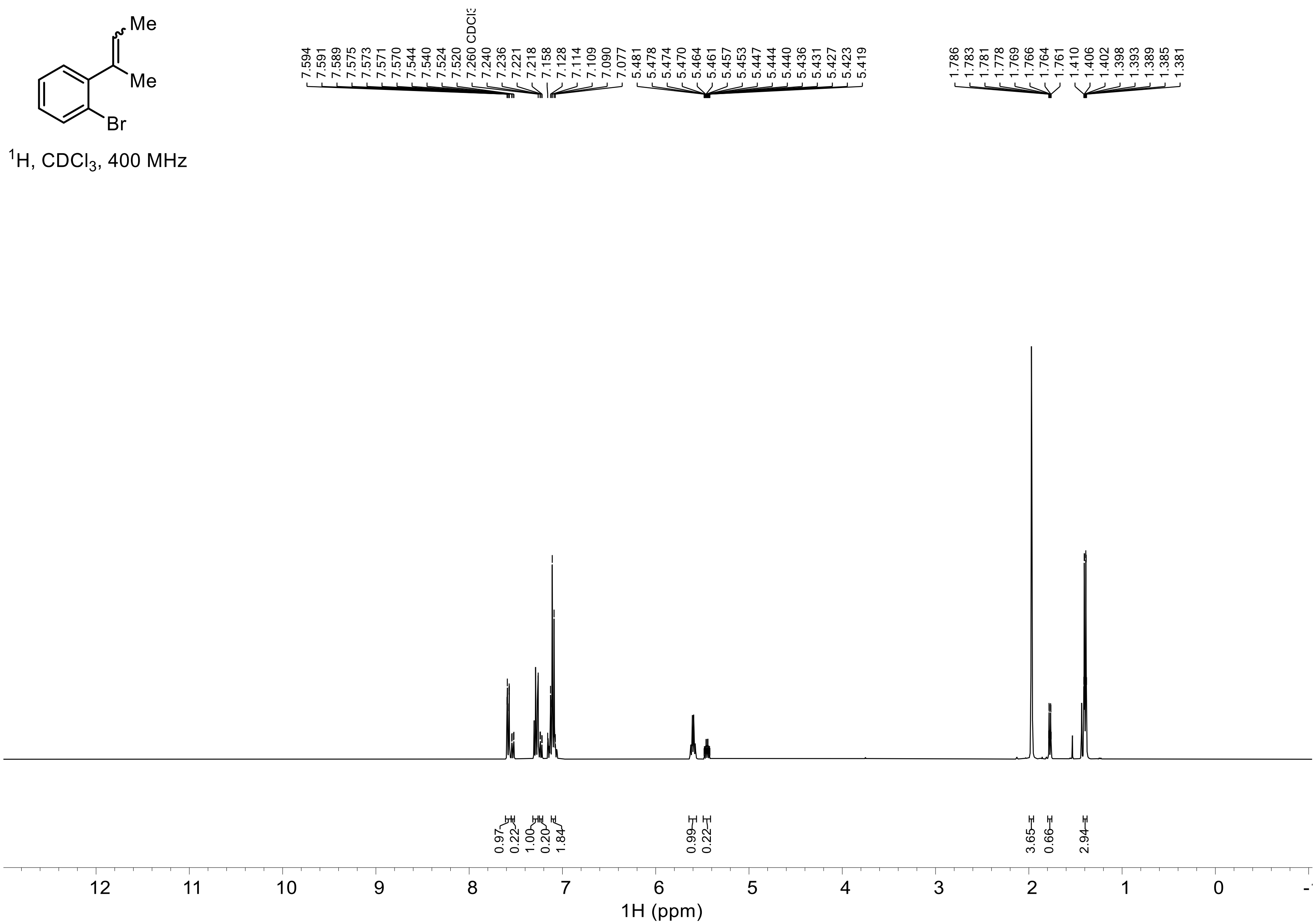

^1H, $CDCl_3$, 400 MHz

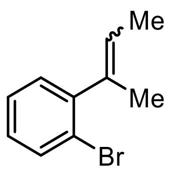

^{13}C {^1H}, $CDCl_3$, 101 MHz

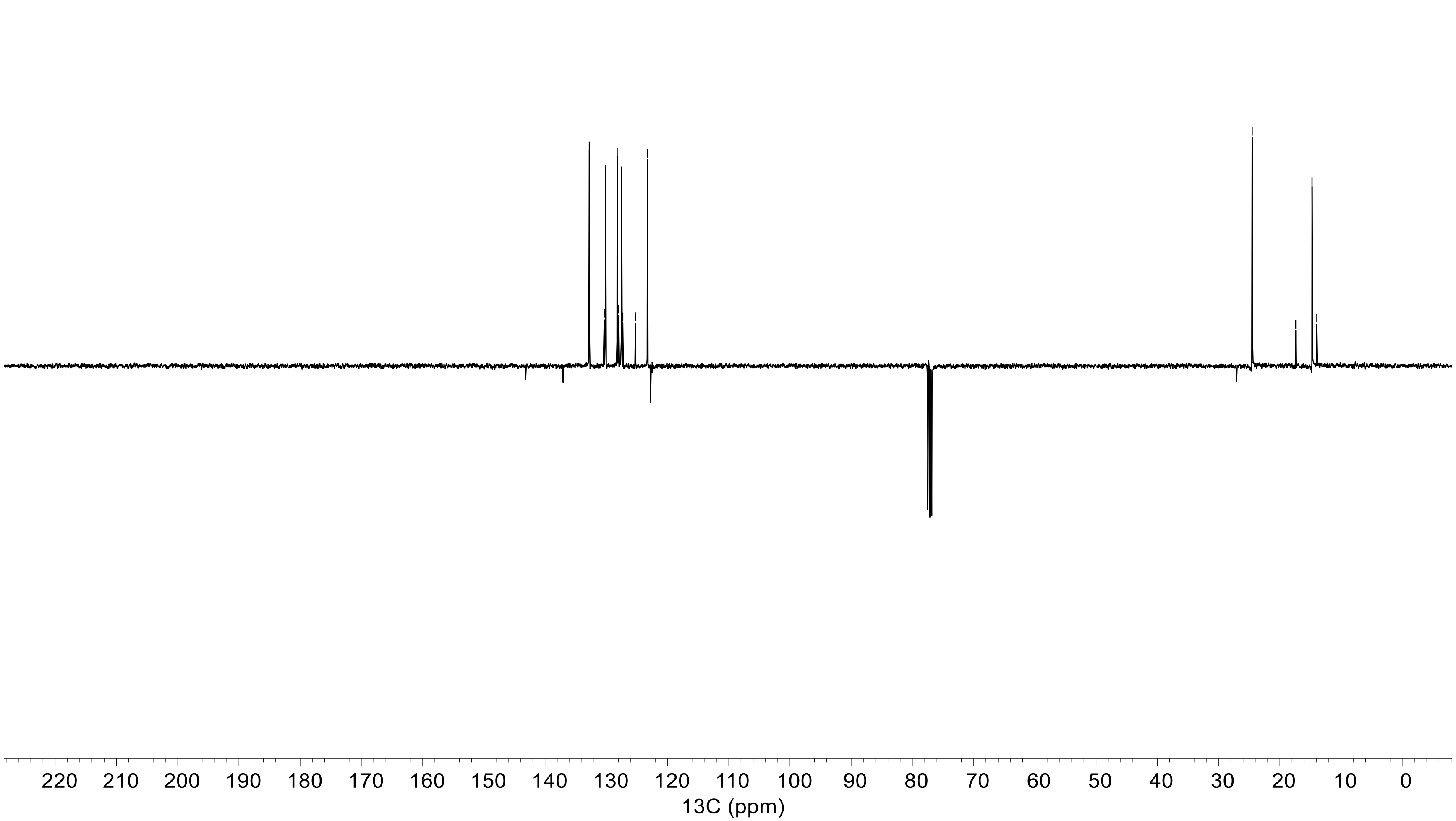

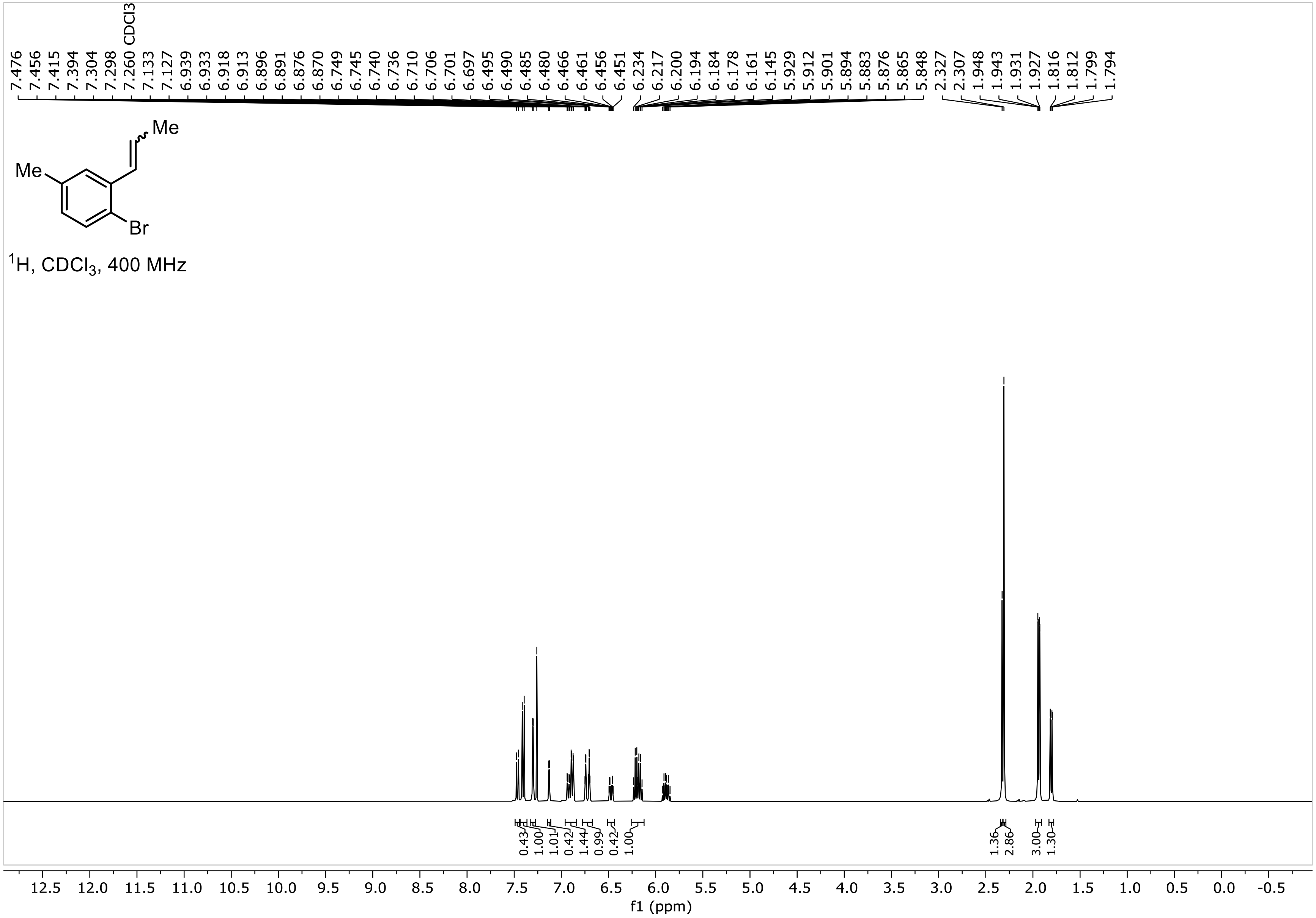

7.476 7.456 7.415 7.394 7.304 7.298 7.260 CDCl3 7.133 7.127 6.939 6.933 6.918 6.913 6.896 6.891 6.876 6.870 6.749 6.745 6.740 6.736 6.710 6.706 6.701 6.697 6.495 6.490 6.485 6.480 6.466 6.461 6.456 6.451 6.234 6.217 6.200 6.194 6.184 6.178 6.161 6.145 5.929 5.912 5.901 5.894 5.883 5.876 5.865 5.848 2.327 2.307 1.948 1.943 1.931 1.927 1.816 1.812 1.799 1.794
Me
Me
Br
^1H, $CDCl_3$, 400 MHz
0.43 1.00 1.01 0.42 1.44 0.99 0.42 1.00 1.36 2.86 3.00 1.30
f1 (ppm)

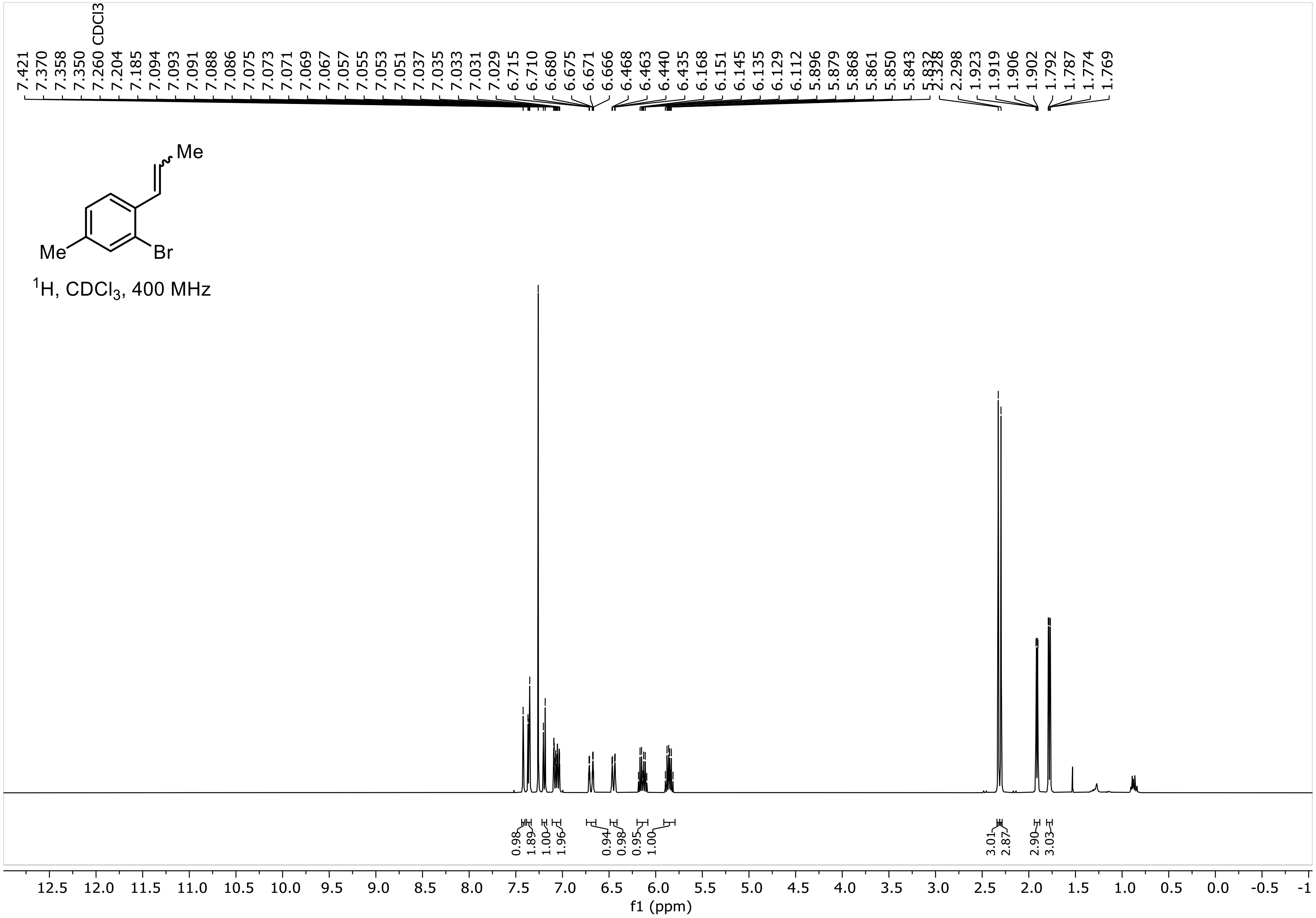
7.421
7.370
7.358
7.350
7.260 CDCl3
7.204
7.185
7.094
7.093
7.091
7.088
7.086
7.075
7.073
7.071
7.069
7.067
7.057
7.055
7.053
7.051
7.037
7.035
7.033
7.031
7.029
6.715
6.710
6.680
6.675
6.671
6.666
6.468
6.463
6.440
6.435
6.168
6.151
6.145
6.135
6.129
6.112
5.896
5.879
5.868
5.861
5.850
5.843
5.832
2.328
2.298
1.923
1.919
1.906
1.902
1.792
1.787
1.774
1.769
Me
Me
Br
^{1}H, CDCl$_3$, 400 MHz
0.98
1.89
1.00
1.96
0.94
0.98
0.95
1.00
3.01
2.87
2.90
3.03
f1 (ppm)

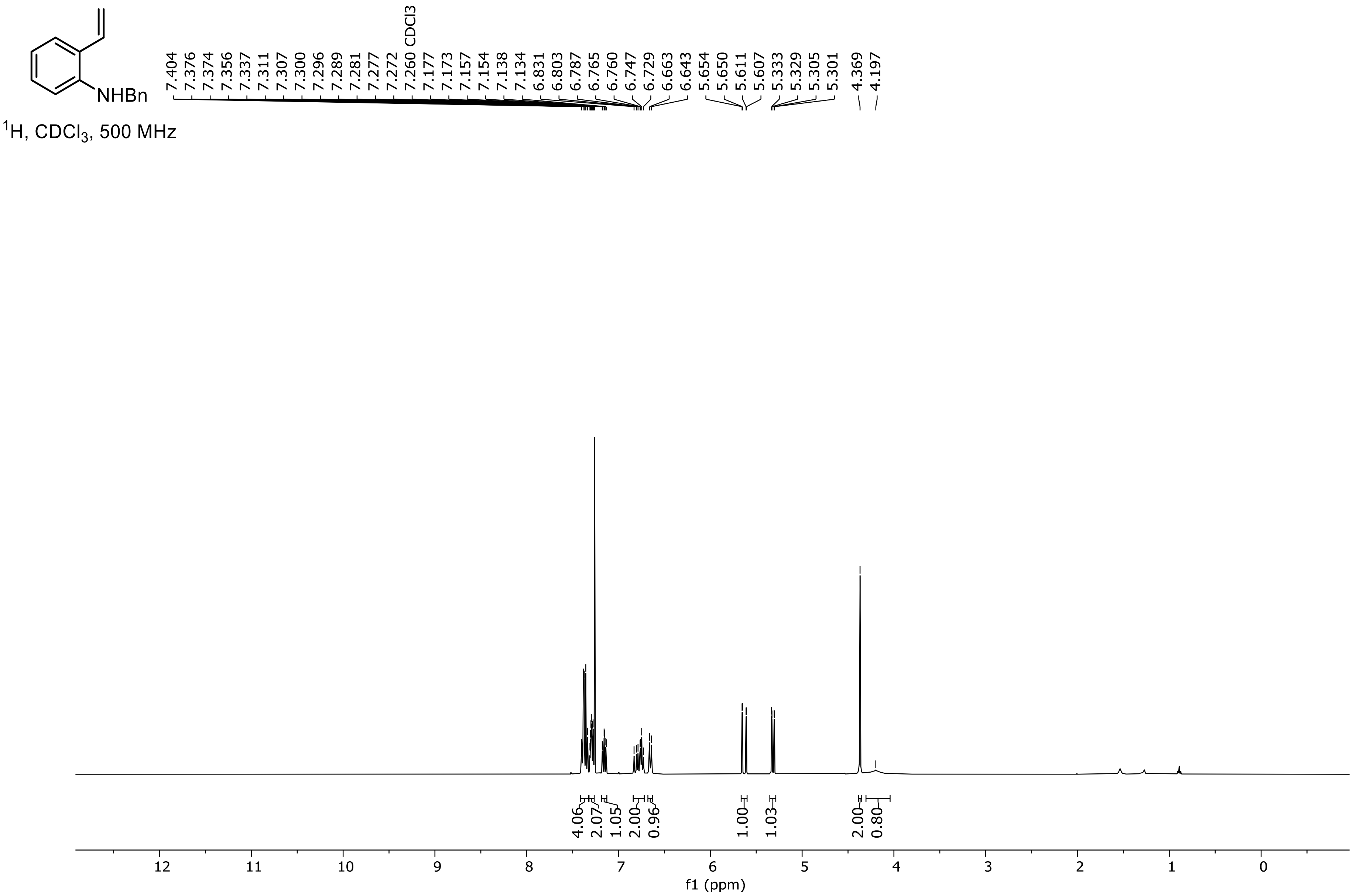

^1H, $CDCl_3$, 500 MHz

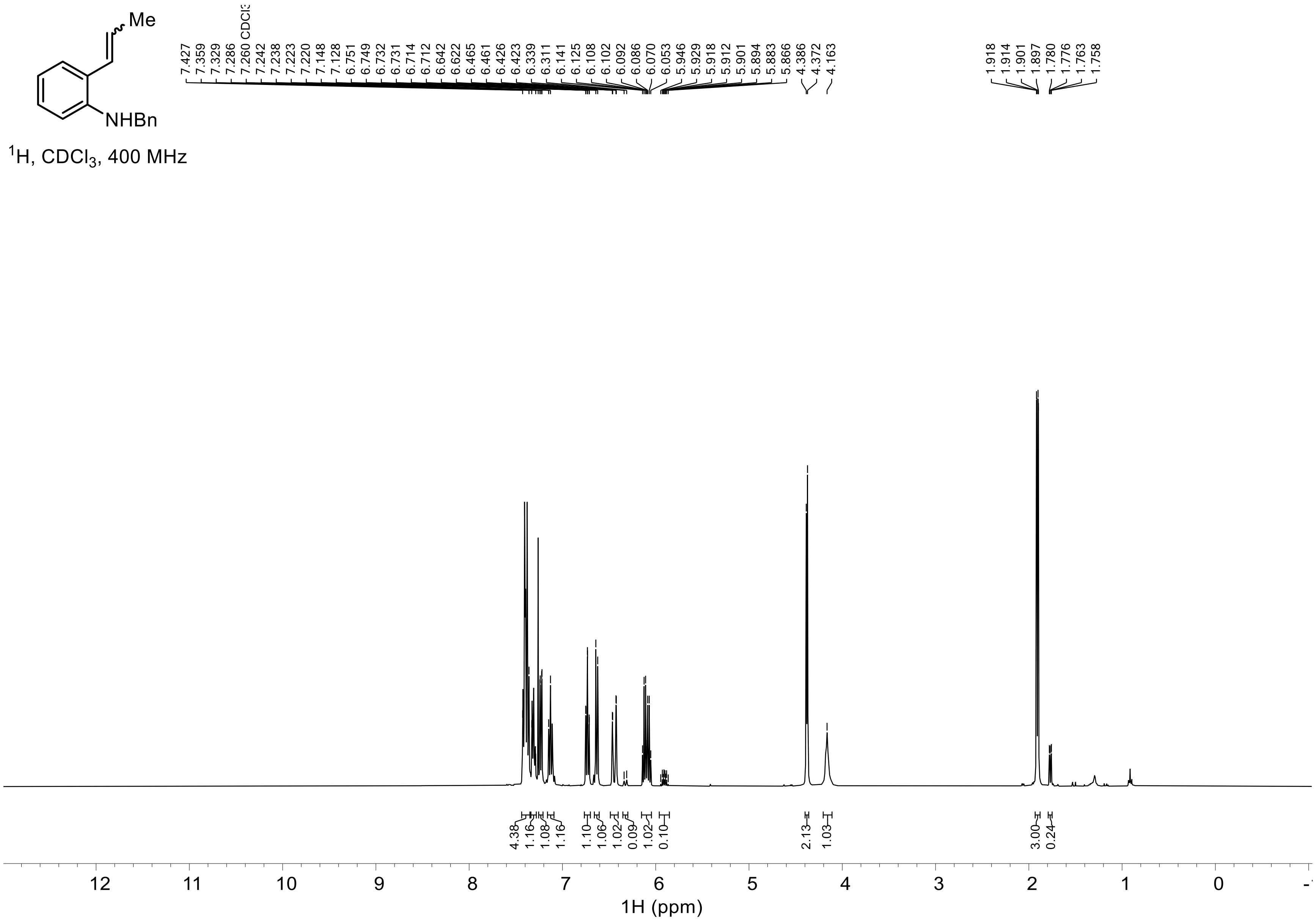

^{1}H, $CDCl_3$, 400 MHz

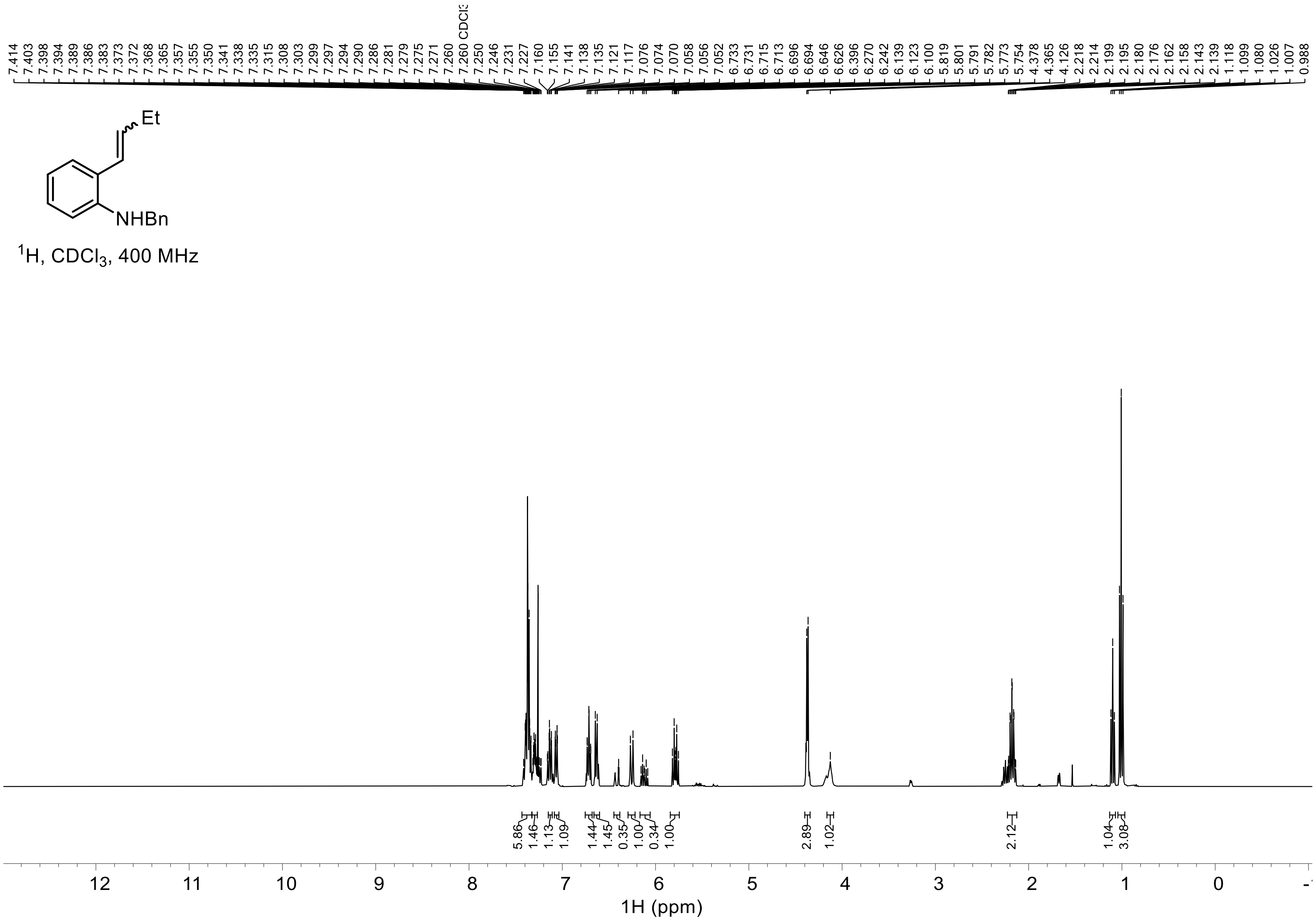

^1H, $CDCl_3$, 400 MHz

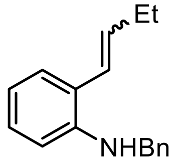

^{13}C {^1H}, $CDCl_3$, 126 MHz

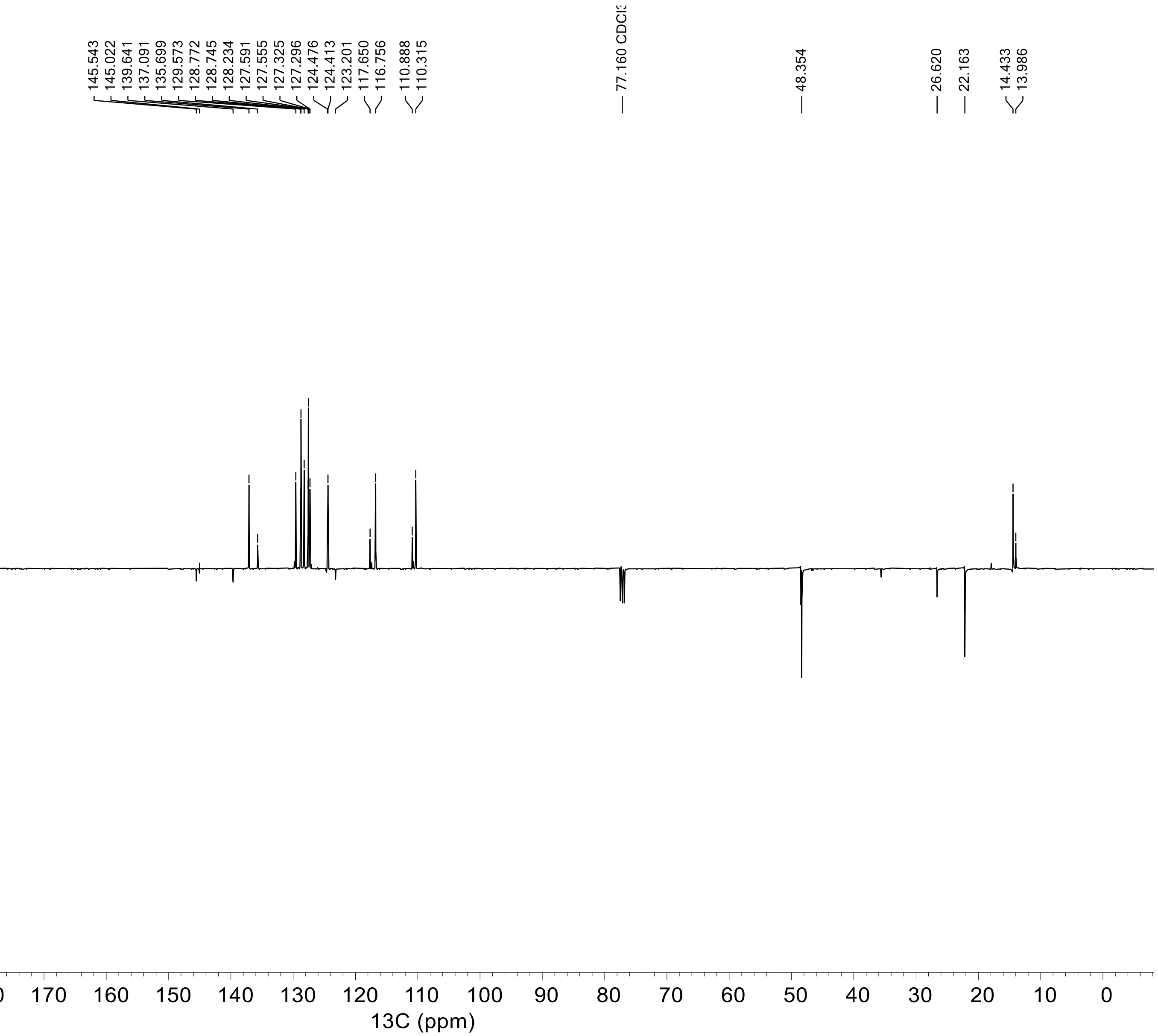

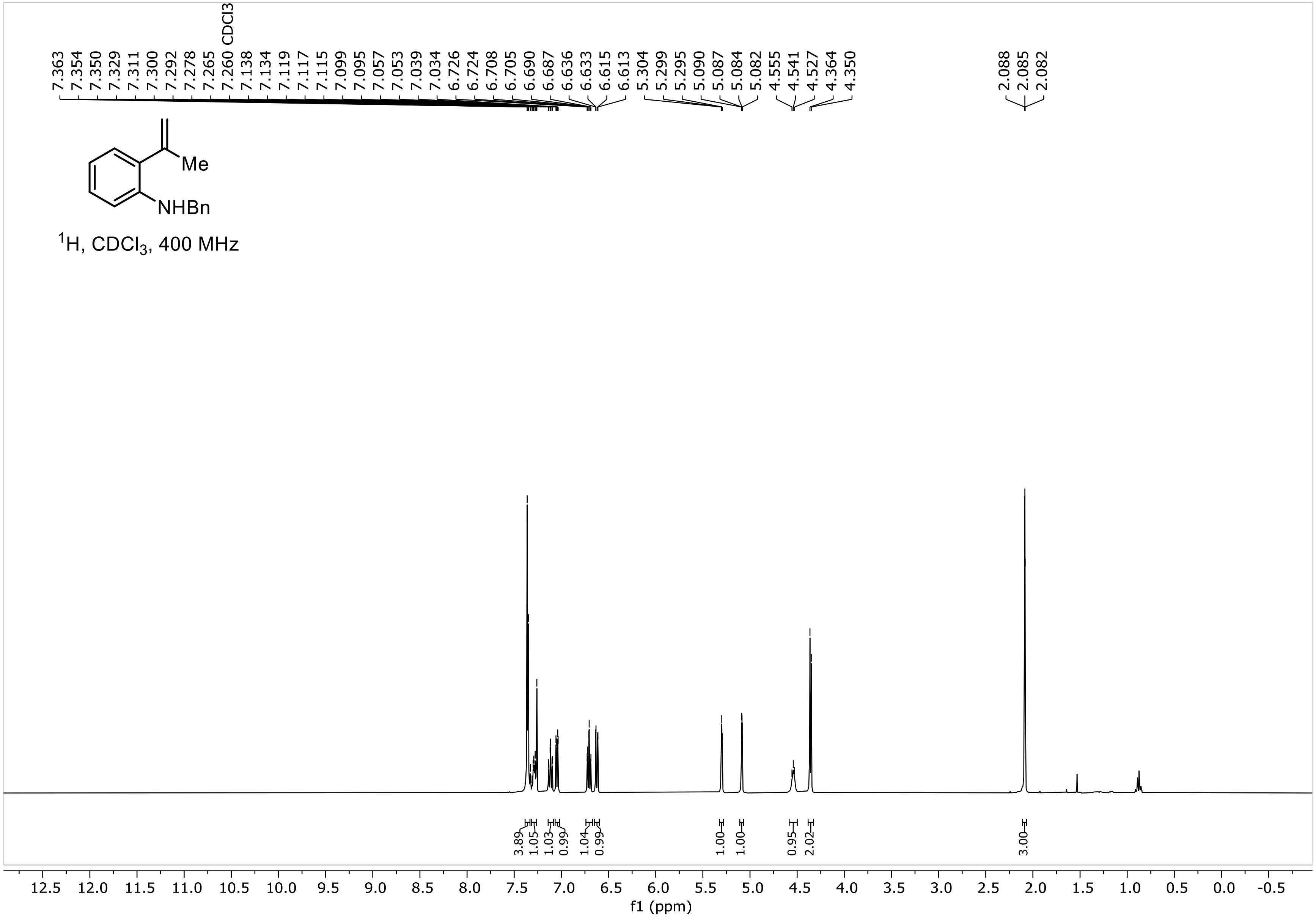

^1H, $CDCl_3$, 400 MHz

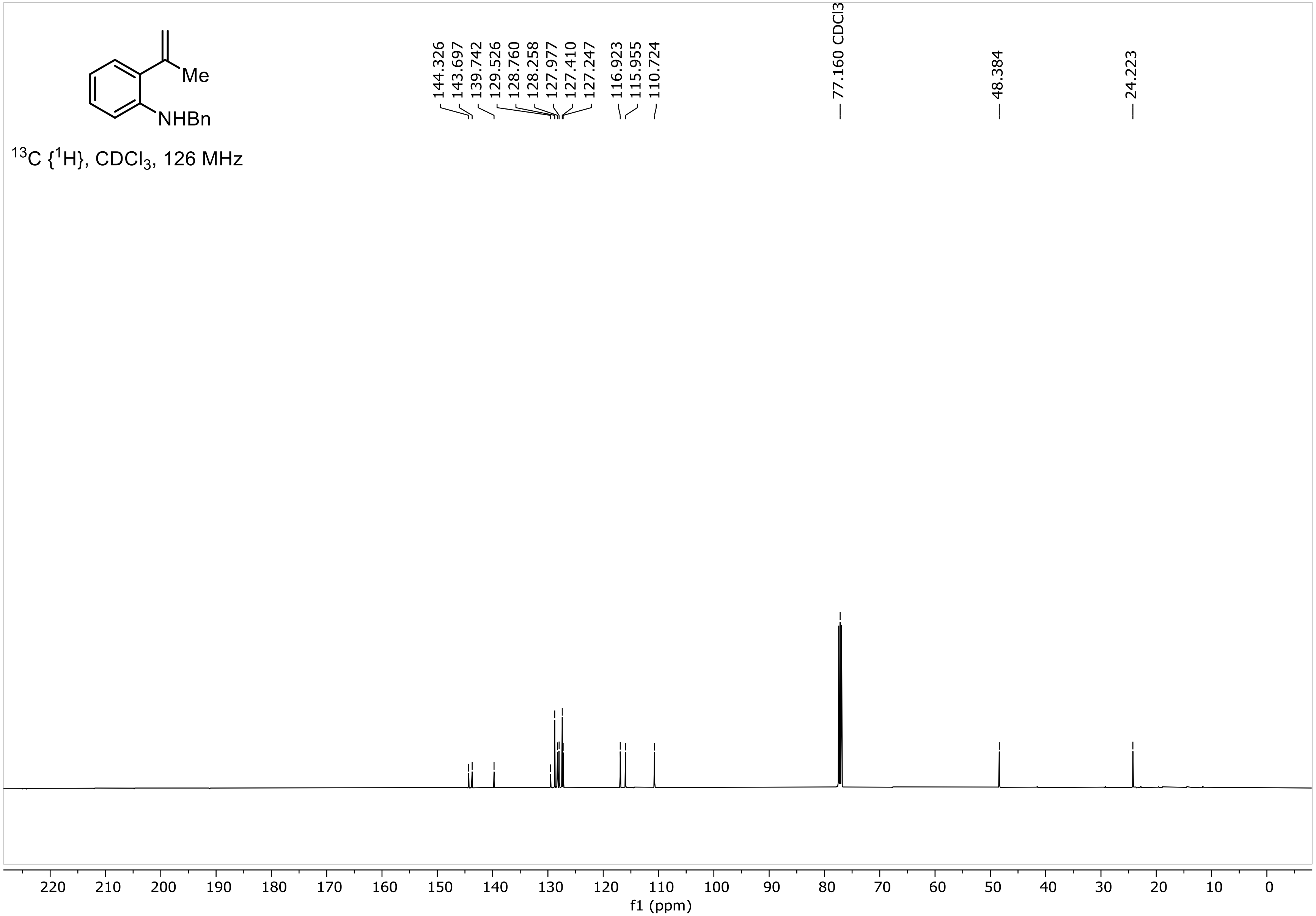
Me
NHBn
$^{13}C\ \{^1H\}$, $CDCl_3$, 126 MHz
144.326
143.697
139.742
129.526
128.760
128.258
127.977
127.410
127.247
116.923
115.955
110.724
77.160 CDCl3
48.384
24.223
220
210
200
190
180
170
160
150
140
130
120
110
100
90
80
70
60
50
40
30
20
10
0
f1 (ppm)

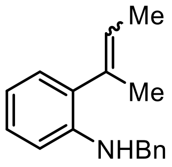

^{1}H, $CDCl_3$, 400 MHz

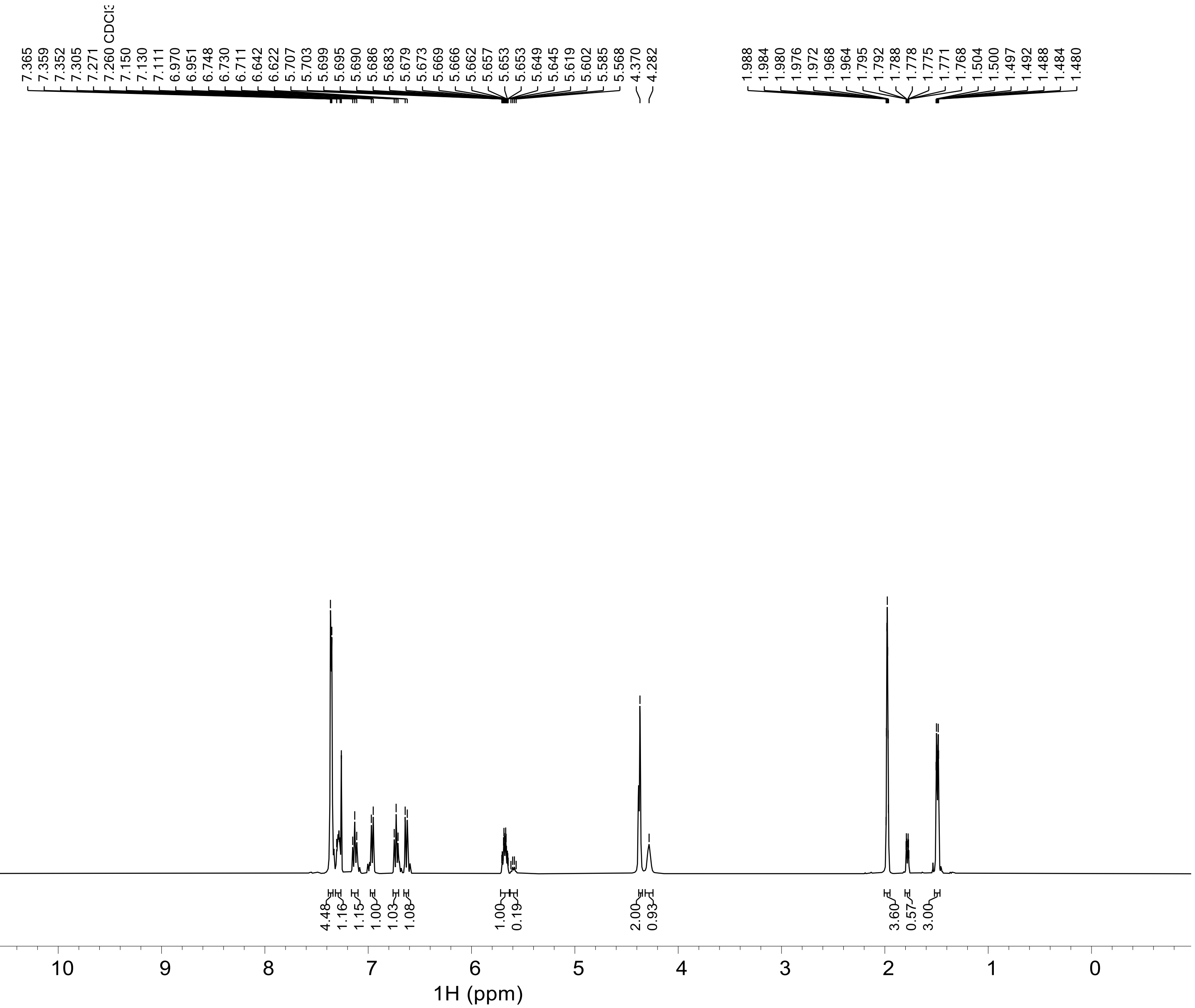

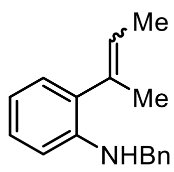

$^{13}C\ \{^{1}H\}$, $CDCl_3$, 101 MHz

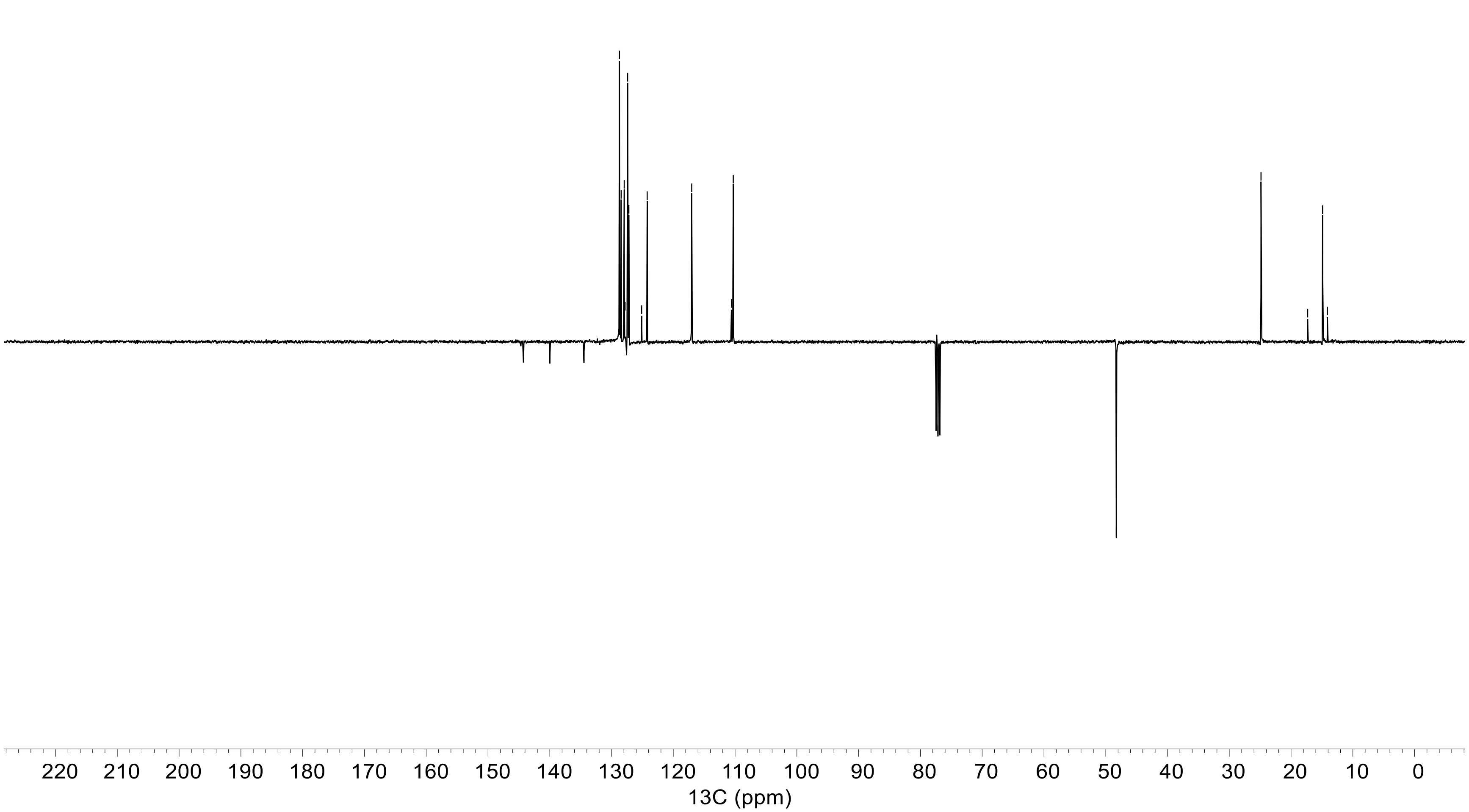

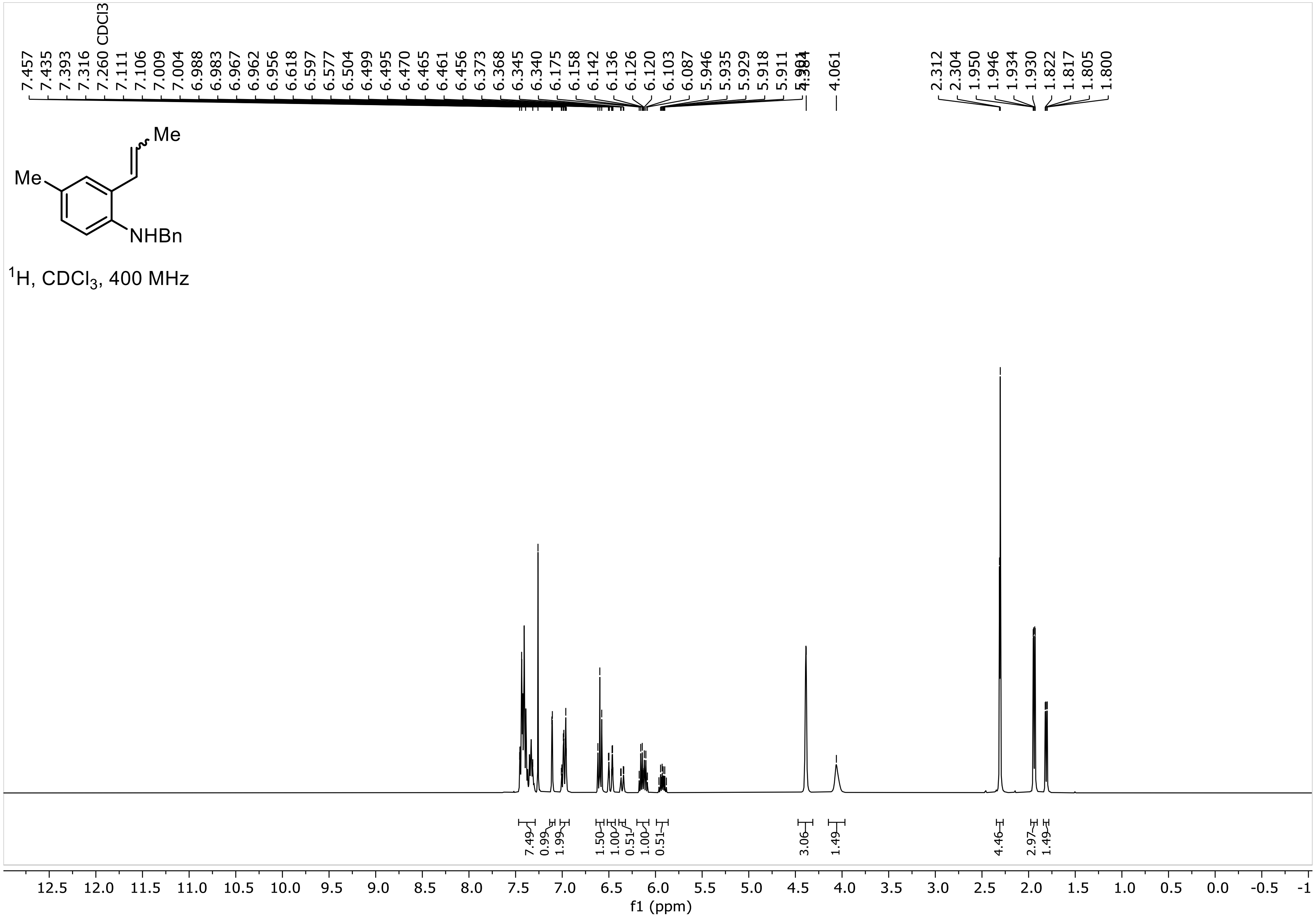
7.457
7.435
7.393
7.316
7.260 CDCl3
7.111
7.106
7.009
7.004
6.988
6.983
6.967
6.962
6.956
6.618
6.597
6.577
6.504
6.499
6.495
6.470
6.465
6.461
6.456
6.373
6.368
6.345
6.340
6.175
6.158
6.142
6.136
6.126
6.120
6.103
6.087
5.946
5.935
5.929
5.918
5.911
4.061
2.312
2.304
1.950
1.946
1.934
1.930
1.822
1.817
1.805
1.800
Me
Me
NHBn
1H, CDCl3, 400 MHz
7.49
0.99
1.99
1.50
1.00
0.51
1.00
0.51
3.06
1.49
4.46
2.97
1.49
f1 (ppm)

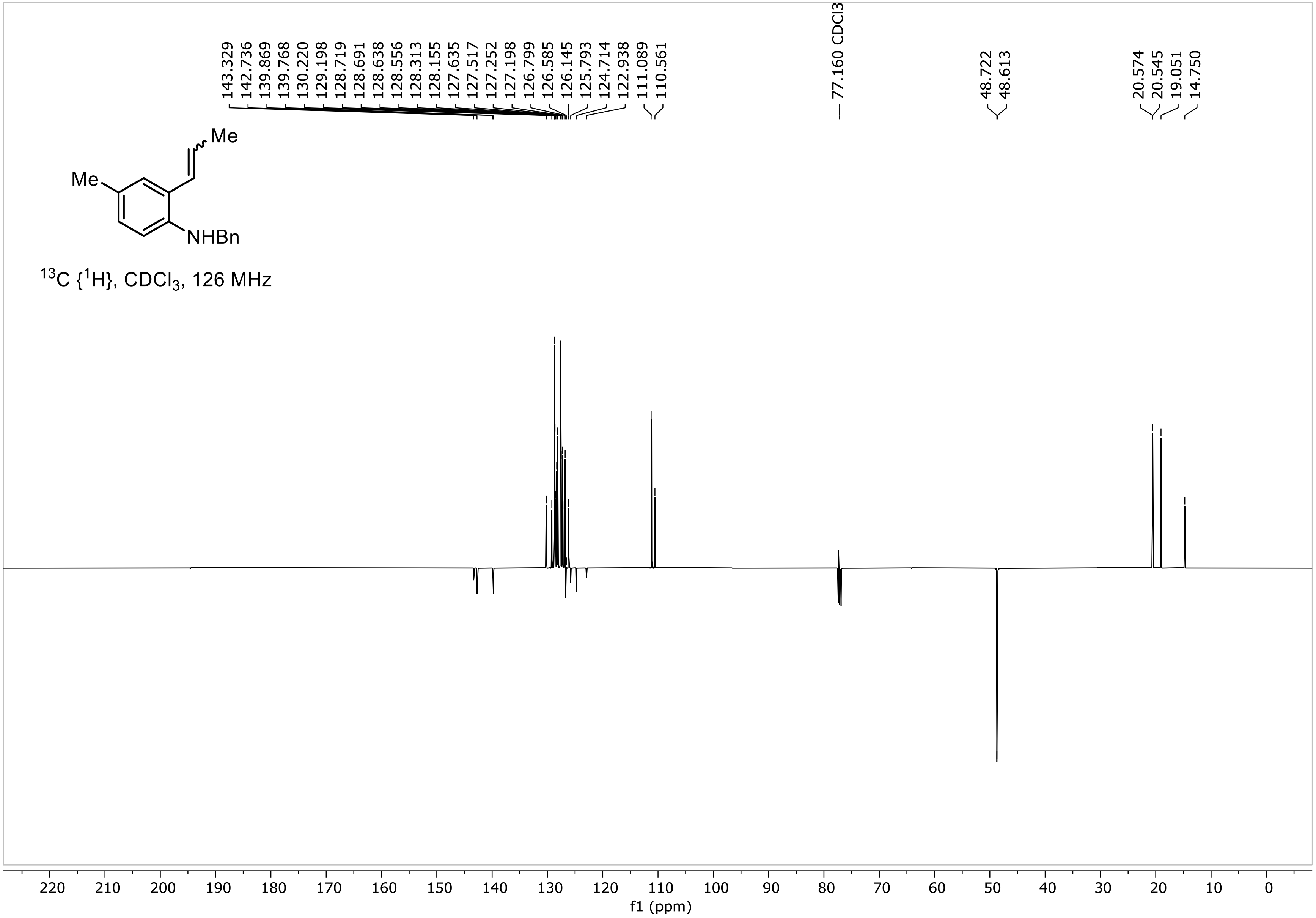
143.329
142.736
139.869
139.768
130.220
129.198
128.719
128.691
128.638
128.556
128.313
128.155
127.635
127.517
127.252
127.198
126.799
126.585
126.145
125.793
124.714
122.938
111.089
110.561
77.160 CDCl3
48.722
48.613
20.574
20.545
19.051
14.750
Me
Me
NHBn
^{13}C {^{1}H}, $CDCl_3$, 126 MHz
220 210 200 190 180 170 160 150 140 130 120 110 100 90 80 70 60 50 40 30 20 10 0
f1 (ppm)

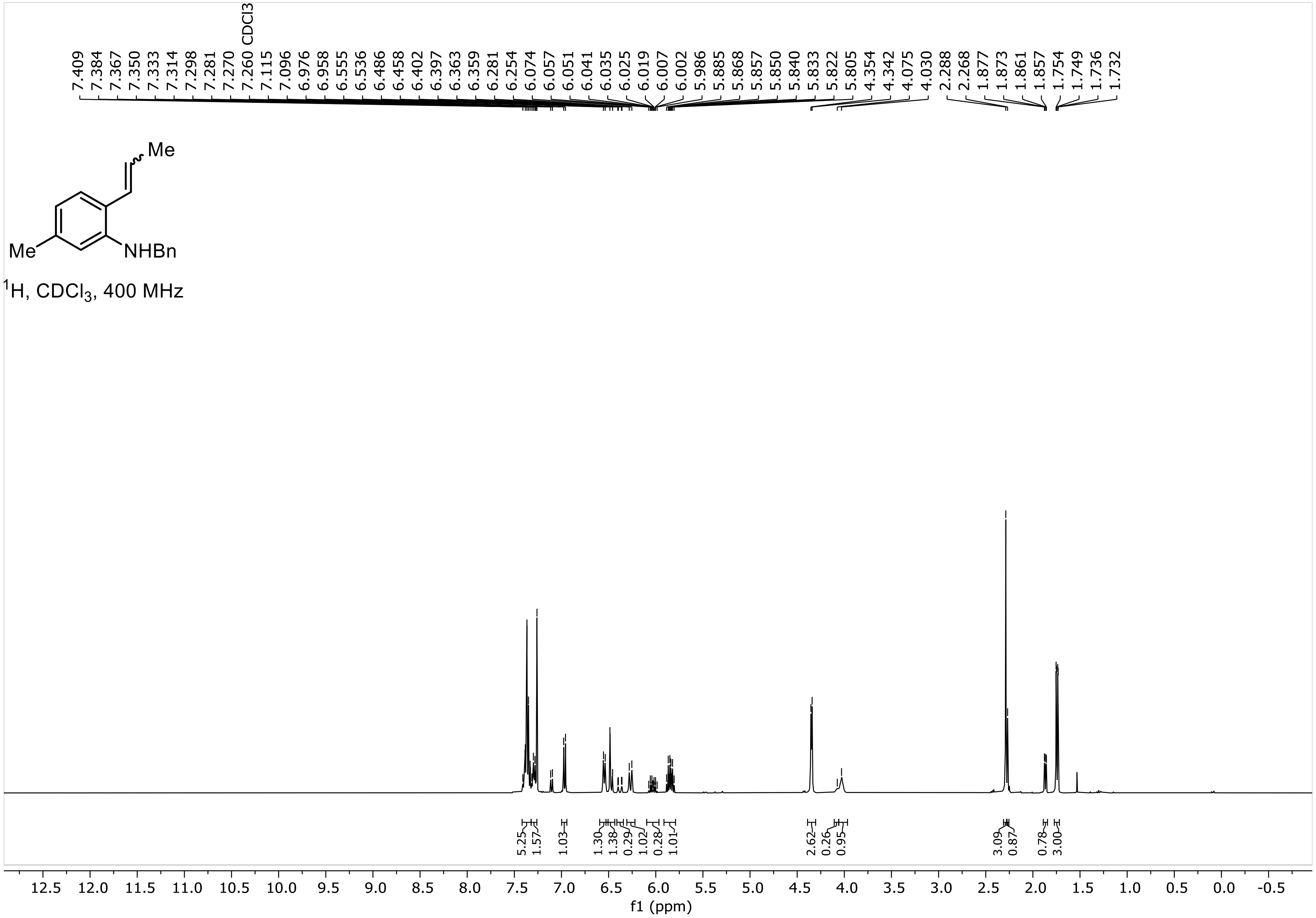
7.409
7.384
7.367
7.350
7.333
7.314
7.298
7.281
7.270
7.260 CDCl3
7.115
7.096
6.976
6.958
6.555
6.536
6.486
6.458
6.402
6.397
6.363
6.359
6.281
6.254
6.074
6.057
6.051
6.041
6.035
6.025
6.019
6.007
6.002
5.986
5.885
5.868
5.857
5.850
5.840
5.833
5.822
5.805
4.354
4.342
4.075
4.030
2.288
2.268
1.877
1.873
1.861
1.857
1.754
1.749
1.736
1.732
Me
Me
NHBn
1H, CDCl3, 400 MHz
5.25
1.57
1.03
1.30
1.38
0.29
1.02
0.28
1.01
2.62
0.26
0.95
3.09
0.87
0.78
3.00
12.5 12.0 11.5 11.0 10.5 10.0 9.5 9.0 8.5 8.0 7.5 7.0 6.5 6.0 5.5 5.0 4.5 4.0 3.5 3.0 2.5 2.0 1.5 1.0 0.5 0.0 -0.5
f1 (ppm)

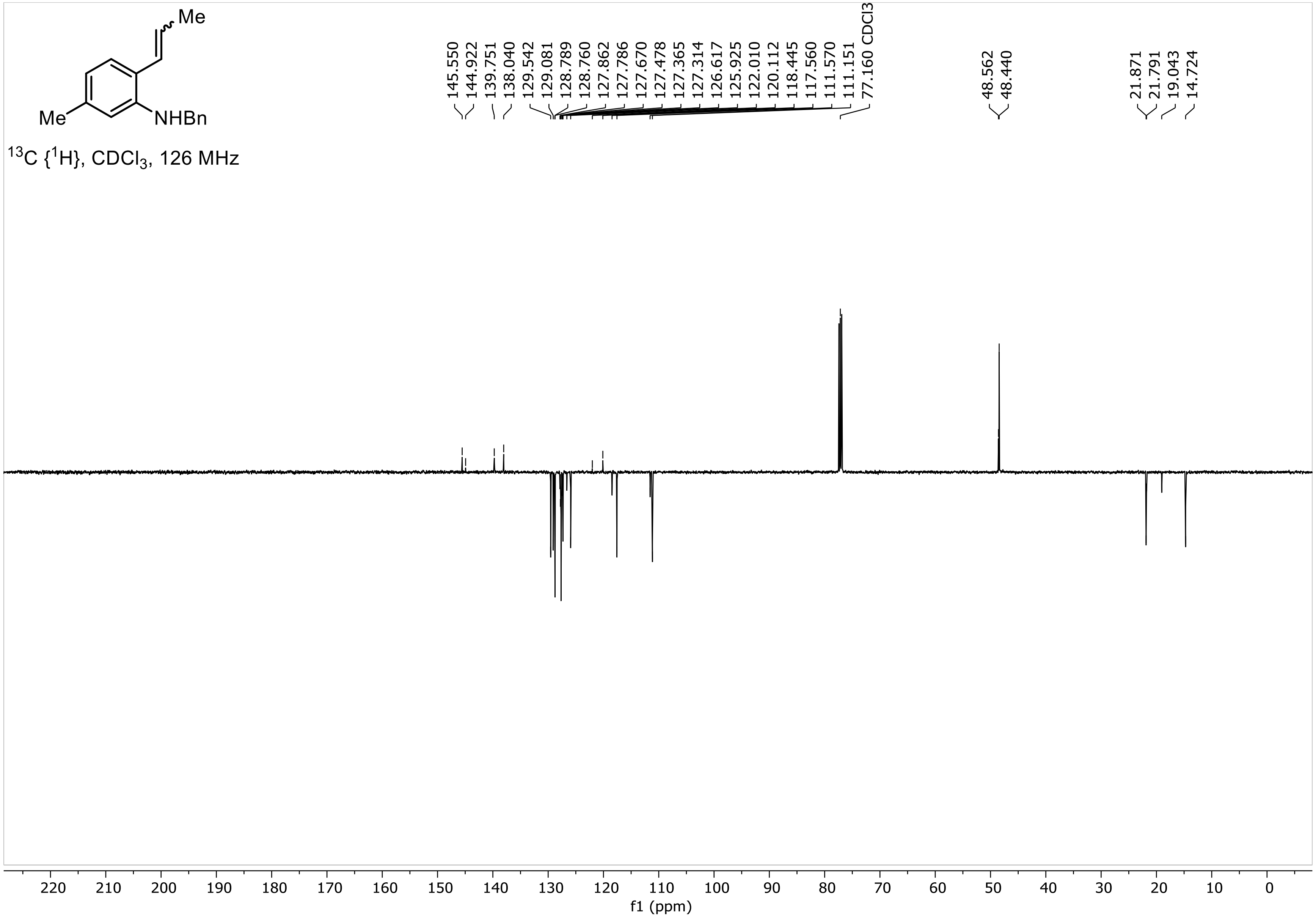
Me
Me
NHBn
^{13}C {^{1}H}, $CDCl_3$, 126 MHz
145.550
144.922
139.751
138.040
129.542
129.081
128.789
128.760
127.862
127.786
127.670
127.478
127.365
127.314
126.617
125.925
122.010
120.112
118.445
117.560
111.570
111.151
77.160 CDCl3
48.562
48.440
21.871
21.791
19.043
14.724
220 210 200 190 180 170 160 150 140 130 120 110 100 90 80 70 60 50 40 30 20 10 0
f1 (ppm)

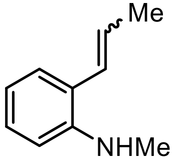

^{1}H, $CDCl_3$, 400 MHz

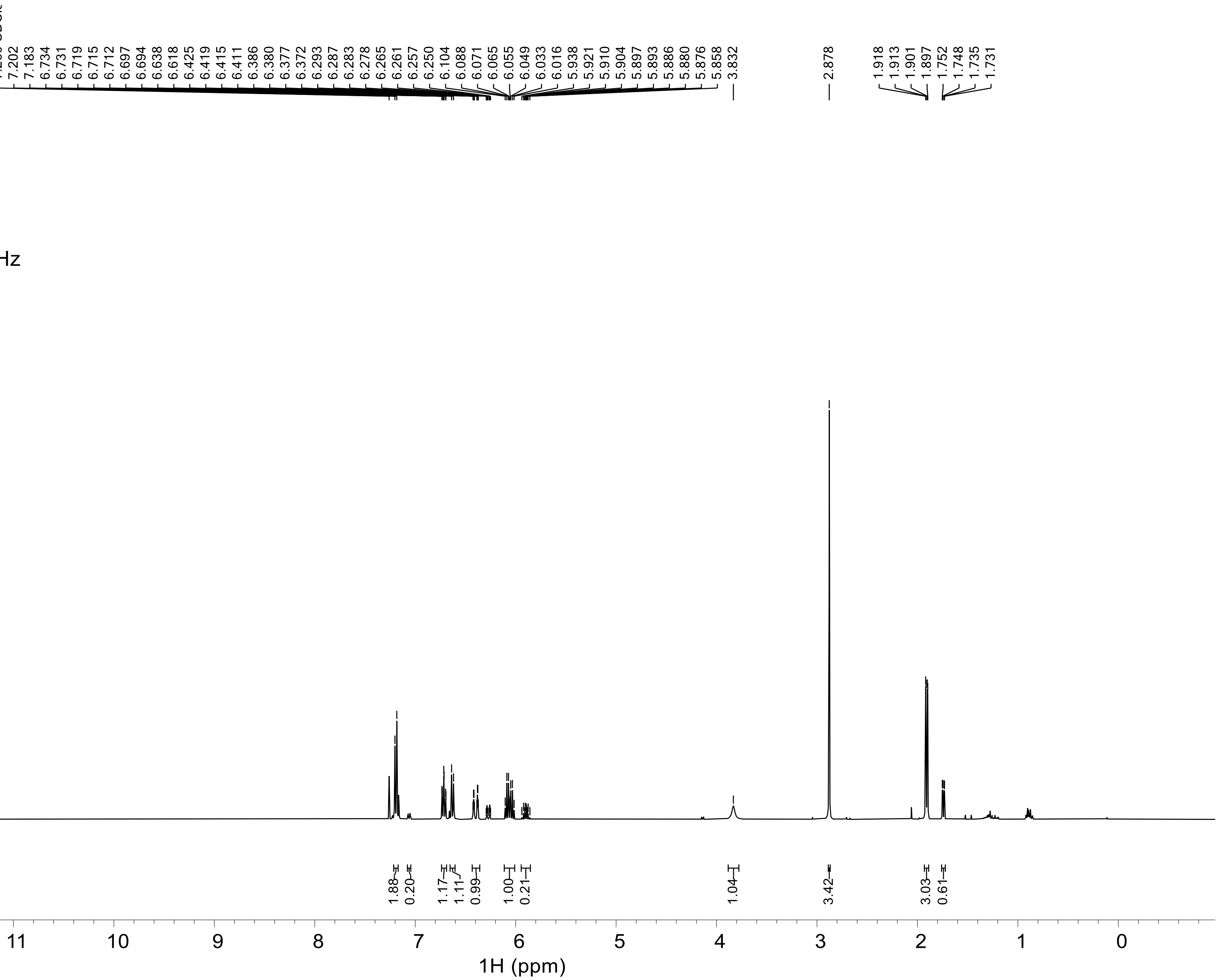

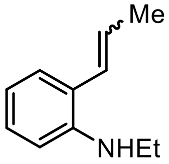

^{1}H, $CDCl_3$, 400 MHz

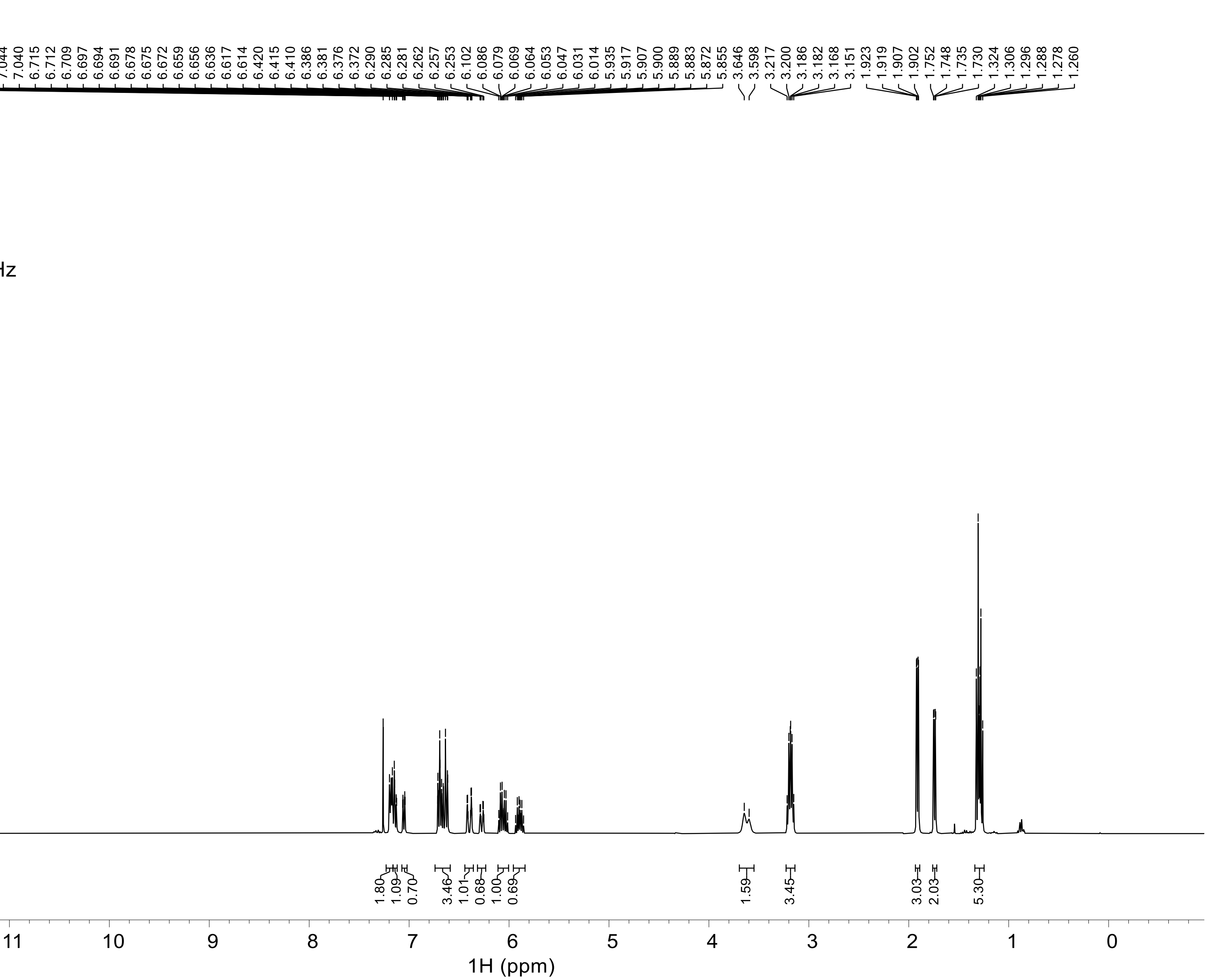

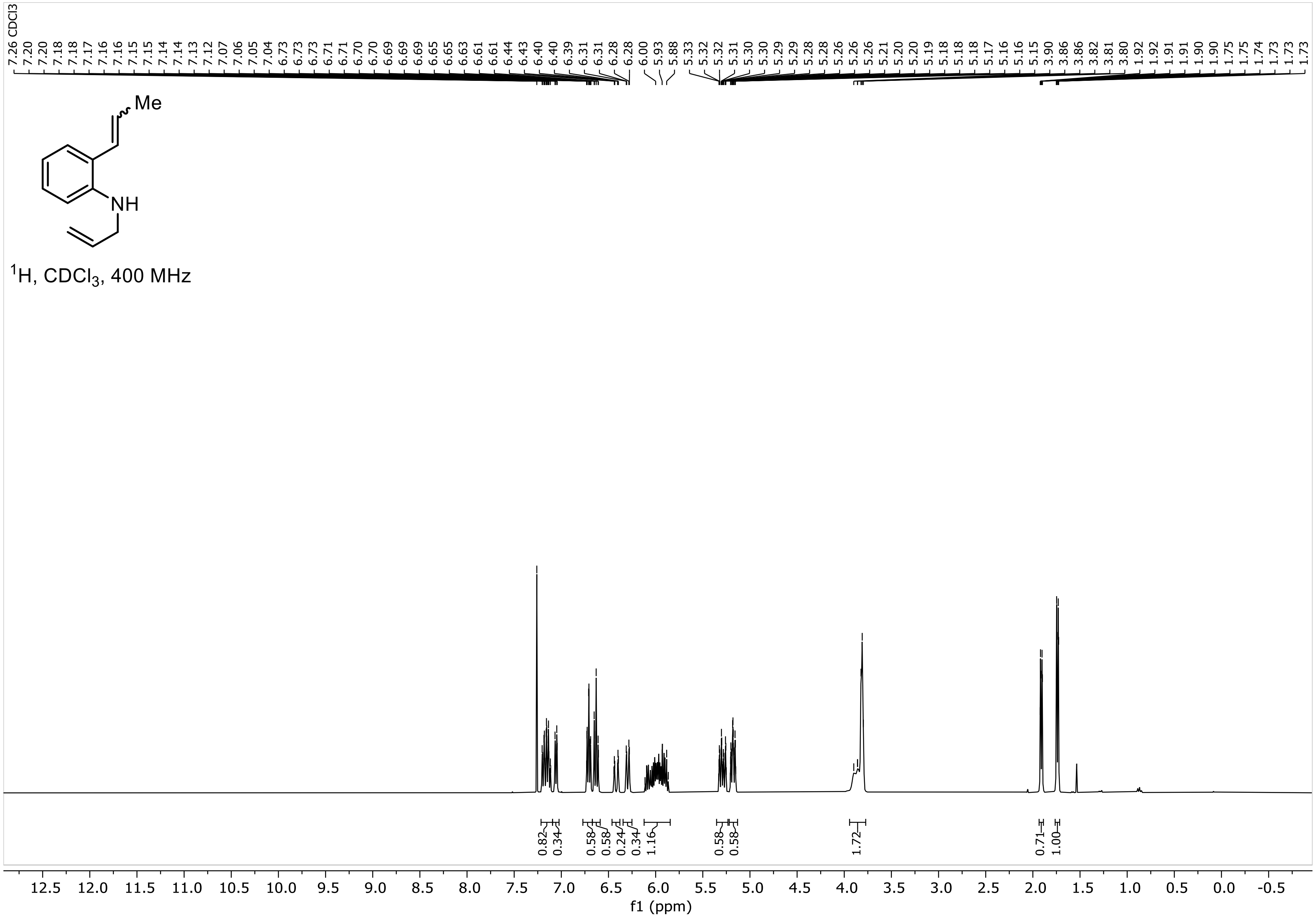
Me
NH
¹H, CDCl₃, 400 MHz
f1 (ppm)

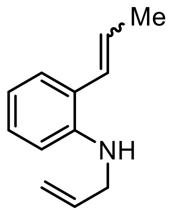

$^{13}C\ \{^1H\}$, $CDCl_3$, 101 MHz

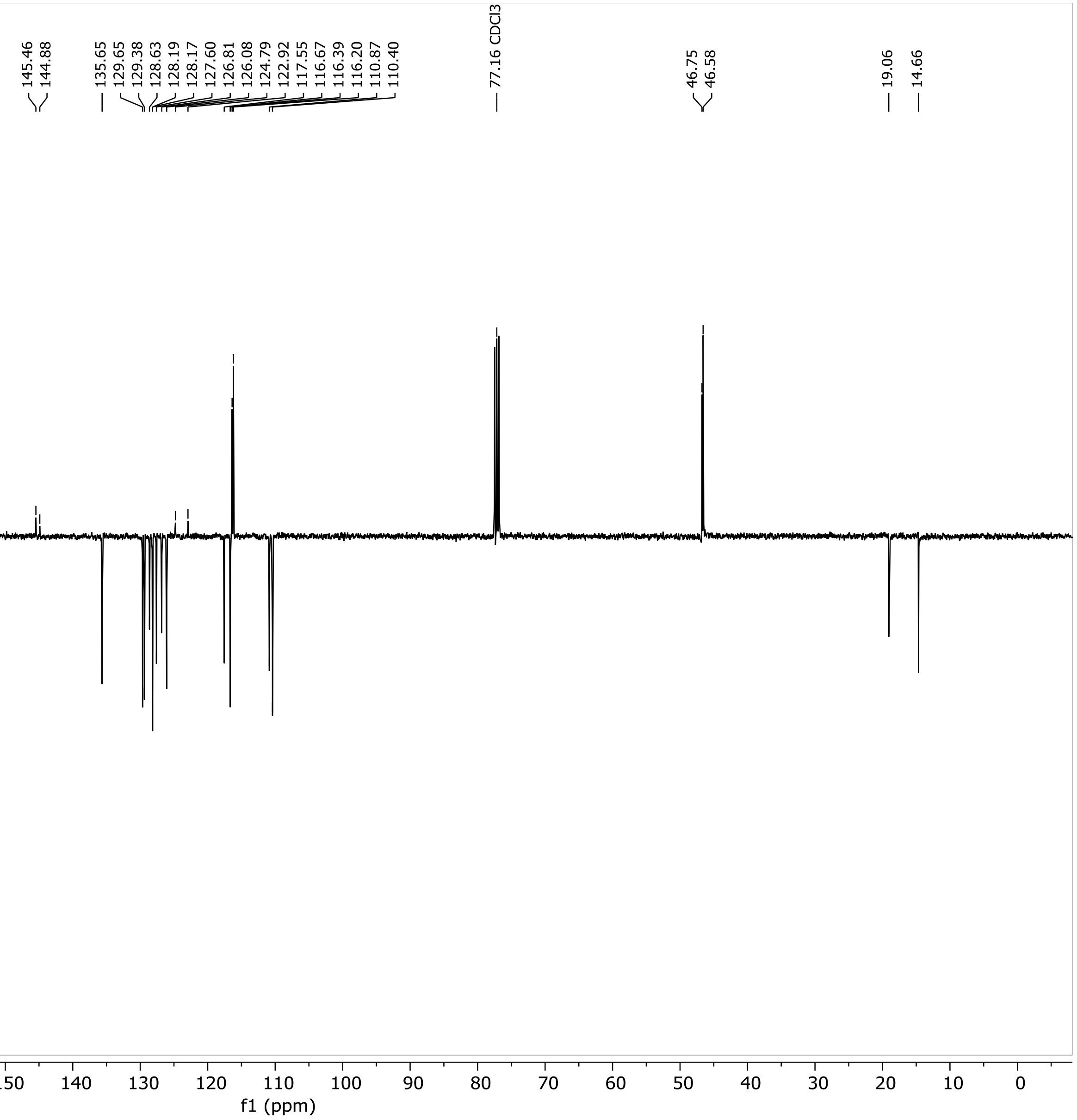

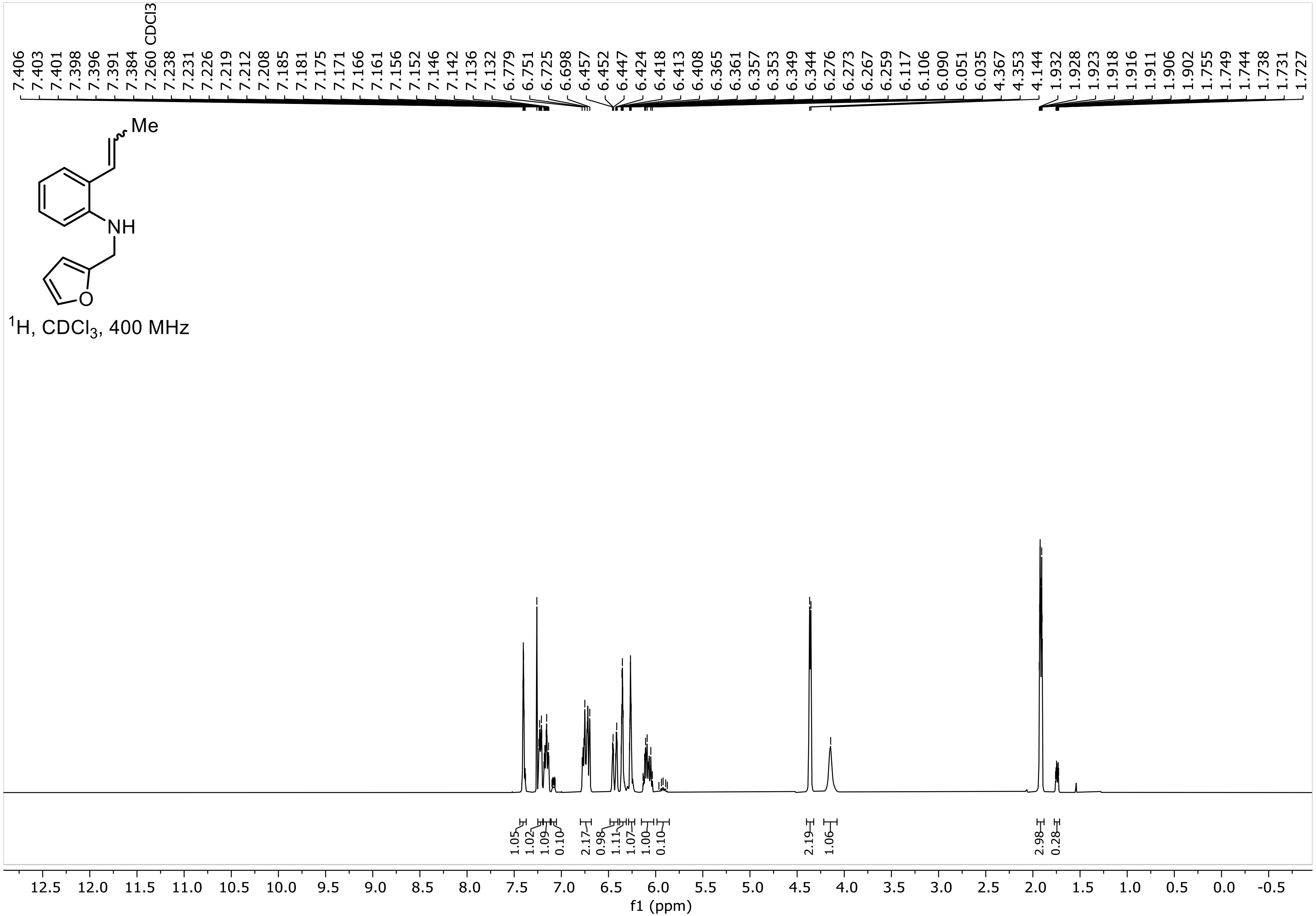
7.406 7.403 7.401 7.398 7.396 7.391 7.384 7.260 CDCl3 7.238 7.231 7.226 7.219 7.212 7.208 7.185 7.181 7.175 7.171 7.166 7.161 7.156 7.152 7.146 7.142 7.136 7.132 6.779 6.751 6.725 6.698 6.457 6.452 6.447 6.424 6.418 6.413 6.408 6.365 6.361 6.357 6.353 6.349 6.344 6.276 6.273 6.267 6.259 6.117 6.106 6.090 6.051 6.035 4.367 4.353 4.144 1.932 1.928 1.923 1.918 1.916 1.911 1.906 1.902 1.755 1.749 1.744 1.738 1.731 1.727
Me
NH
O
1H, CDCl3, 400 MHz
1.05 1.02 1.09 0.10 2.17 0.98 1.11 1.07 1.00 0.10 2.19 1.06 2.98 0.28
12.5 12.0 11.5 11.0 10.5 10.0 9.5 9.0 8.5 8.0 7.5 7.0 6.5 6.0 5.5 5.0 4.5 4.0 3.5 3.0 2.5 2.0 1.5 1.0 0.5 0.0 -0.5
f1 (ppm)

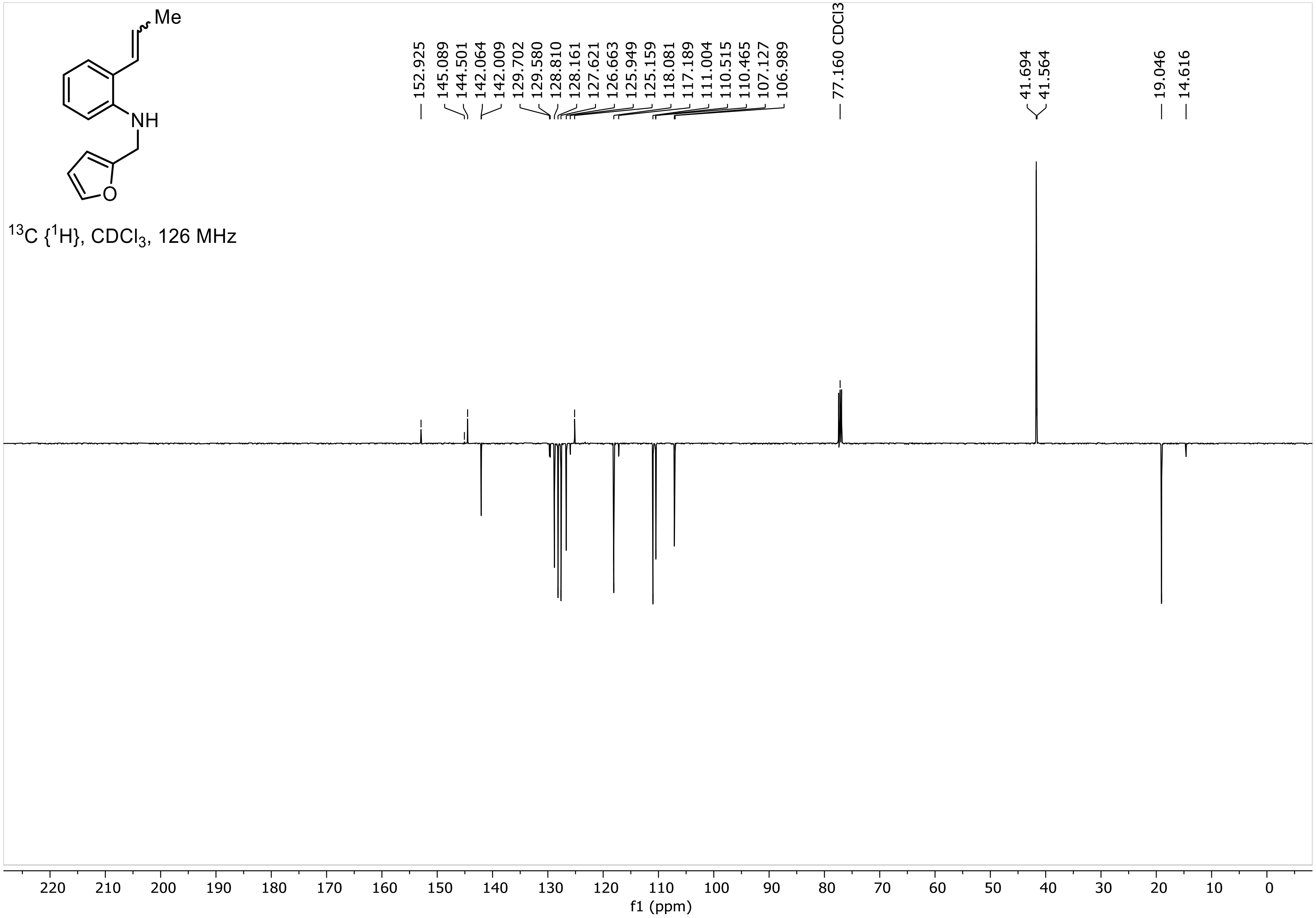
Me
NH
O
13C {1H}, CDCl3, 126 MHz
152.925
145.089
144.501
142.064
142.009
129.702
129.580
128.810
128.161
127.621
126.663
125.949
125.159
118.081
117.189
111.004
110.515
110.465
107.127
106.989
77.160 CDCl3
41.694
41.564
19.046
14.616
220 210 200 190 180 170 160 150 140 130 120 110 100 90 80 70 60 50 40 30 20 10 0
f1 (ppm)

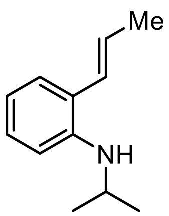

^{1}H, $CDCl_3$, 400 MHz

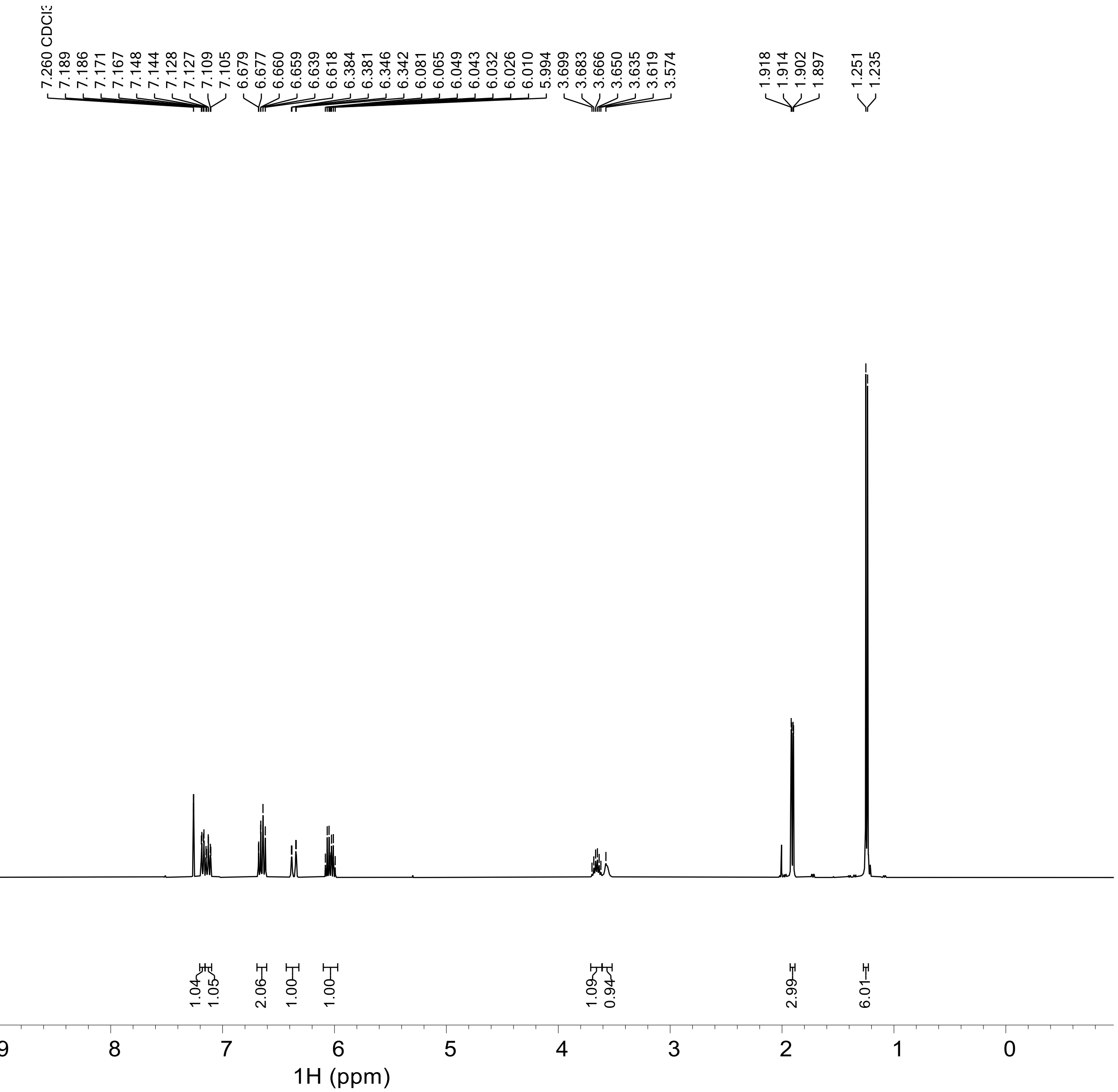

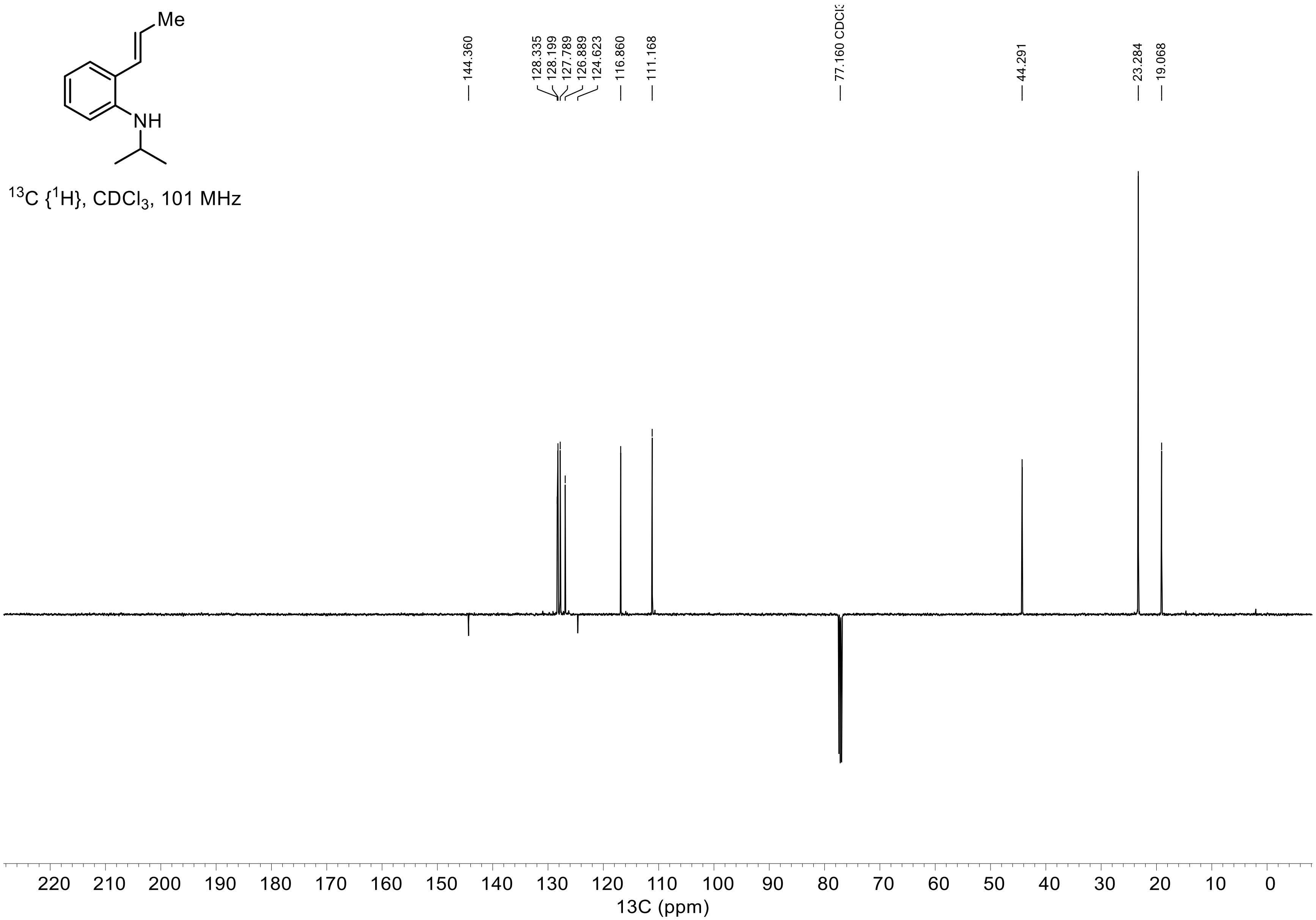
Me
NH
$^{13}C \{^1H\}$, $CDCl_3$, 101 MHz
144.360
128.335
128.199
127.789
126.889
124.623
116.860
111.168
77.160 CDCl3
44.291
23.284
19.068
220 210 200 190 180 170 160 150 140 130 120 110 100 90 80 70 60 50 40 30 20 10 0
13C (ppm)

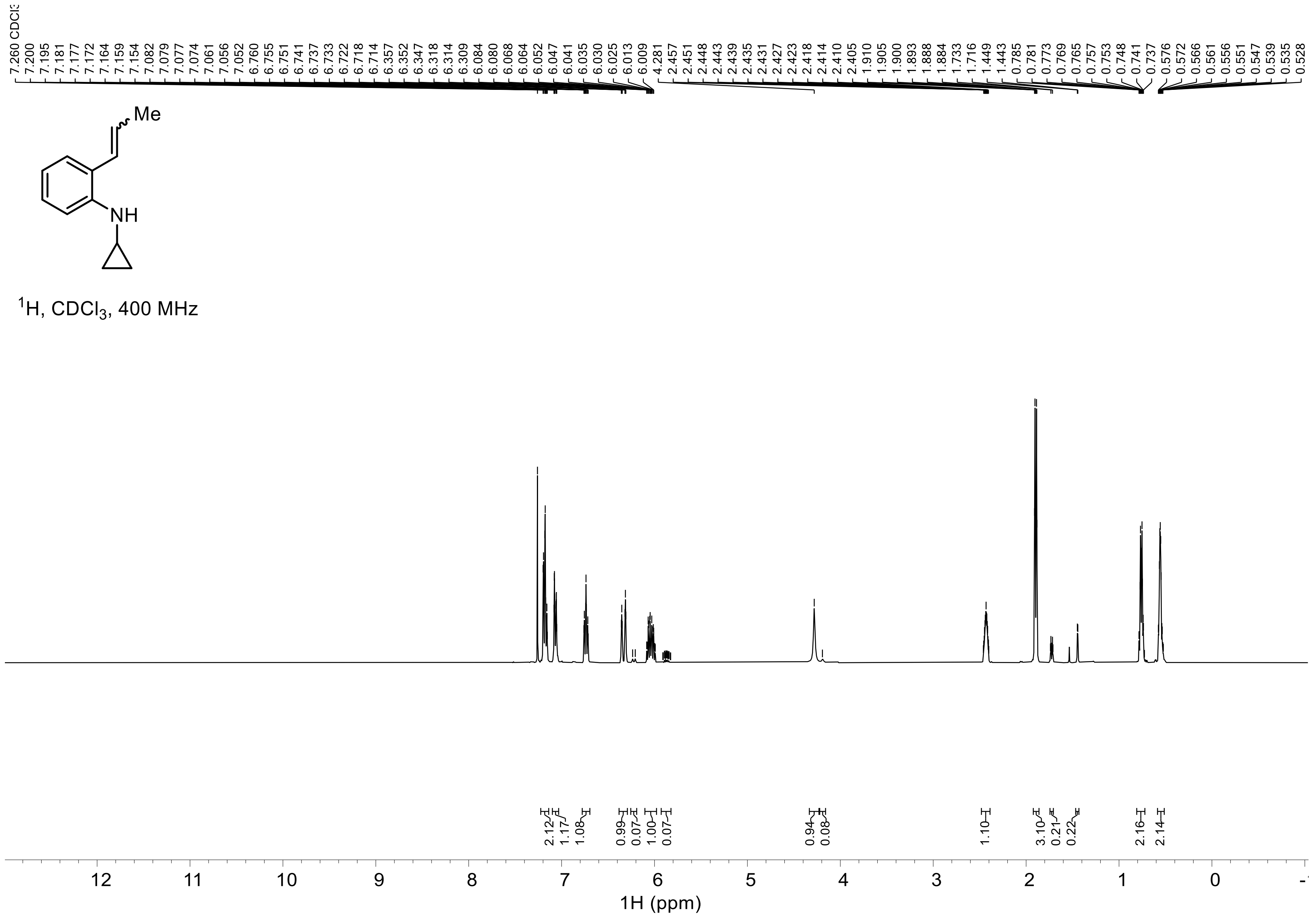

^{1}H, $CDCl_3$, 400 MHz

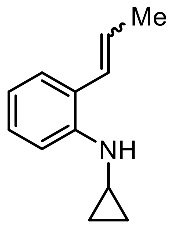

$^{13}C\ \{^{1}H\}$, $CDCl_3$, 126 MHz

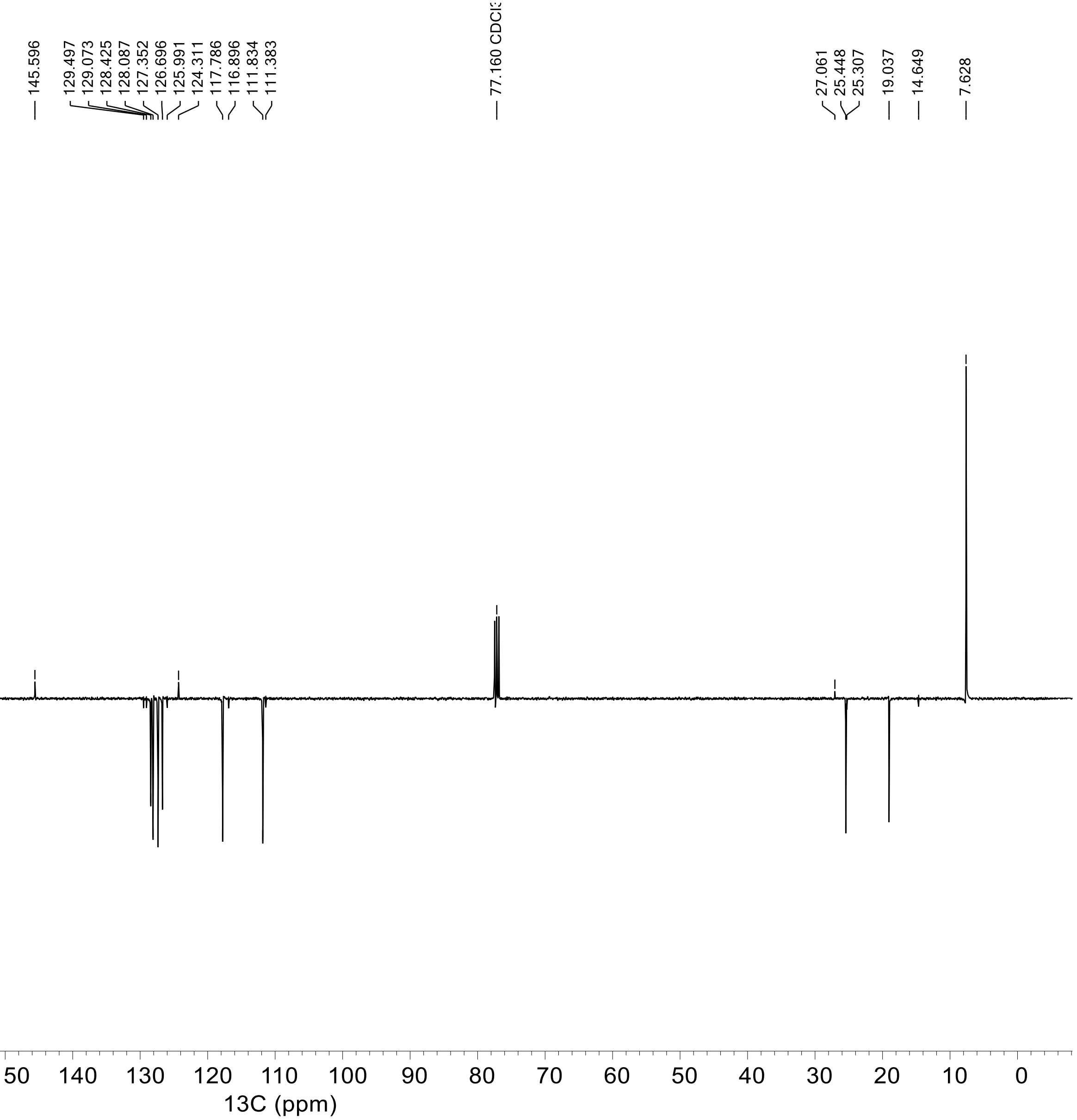

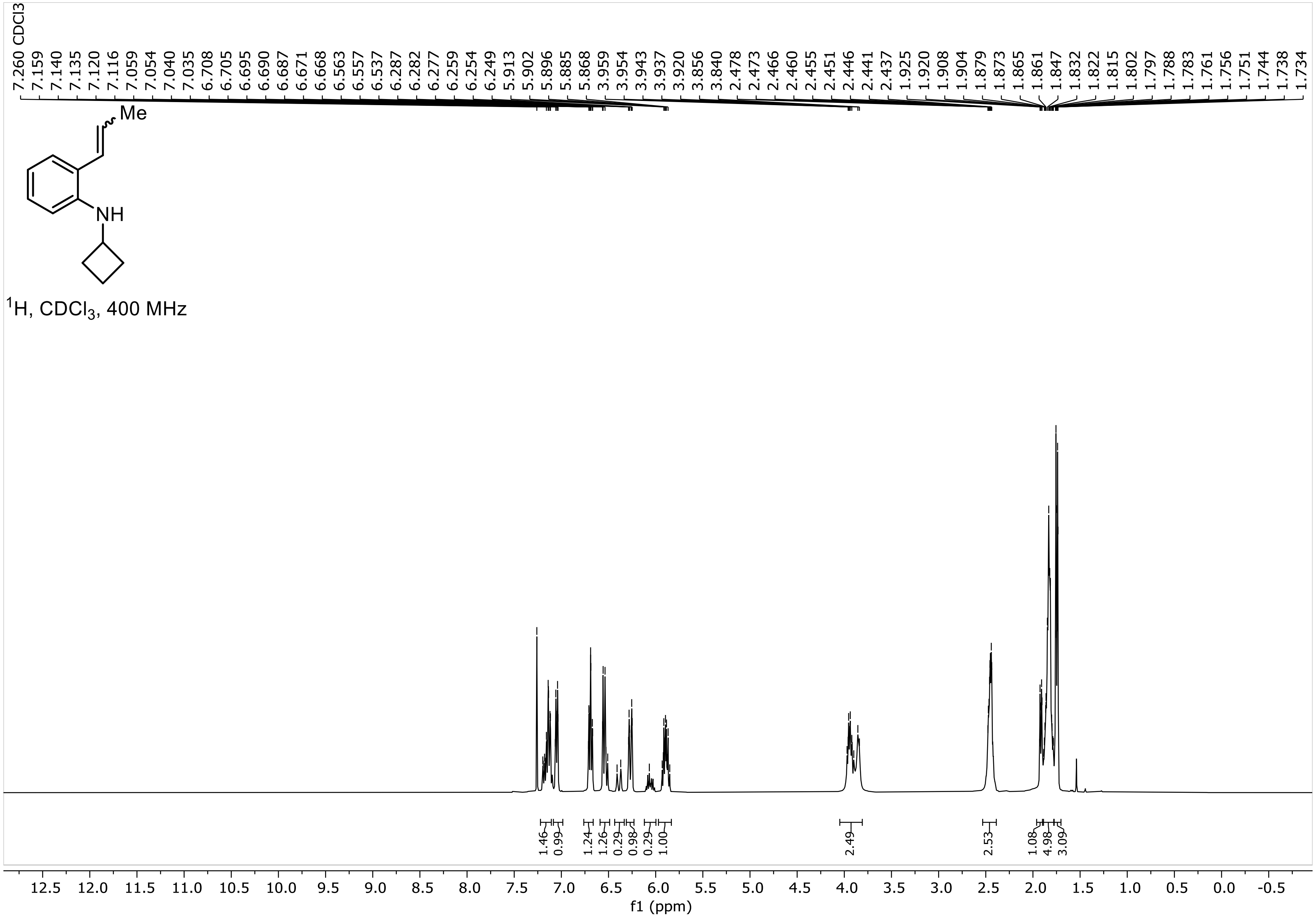
7.260 CDCl3
7.159 7.140 7.135 7.120 7.116 7.059 7.054 7.040 7.035 6.708 6.705 6.695 6.690 6.687 6.671 6.668 6.563 6.557 6.537 6.287 6.282 6.277 6.259 6.254 6.249 5.913 5.902 5.896 5.885 5.868 3.959 3.954 3.943 3.937 3.920 3.856 3.840 2.478 2.473 2.466 2.460 2.455 2.451 2.446 2.441 2.437 1.925 1.920 1.908 1.904 1.879 1.873 1.865 1.861 1.847 1.832 1.822 1.815 1.802 1.797 1.788 1.783 1.761 1.756 1.751 1.744 1.738 1.734
Me
NH
^{1}H, $CDCl_3$, 400 MHz
1.46 0.99 1.24 1.26 0.29 0.98 0.29 1.00 2.49 2.53 1.08 4.98 3.09
12.5 12.0 11.5 11.0 10.5 10.0 9.5 9.0 8.5 8.0 7.5 7.0 6.5 6.0 5.5 5.0 4.5 4.0 3.5 3.0 2.5 2.0 1.5 1.0 0.5 0.0 -0.5
f1 (ppm)

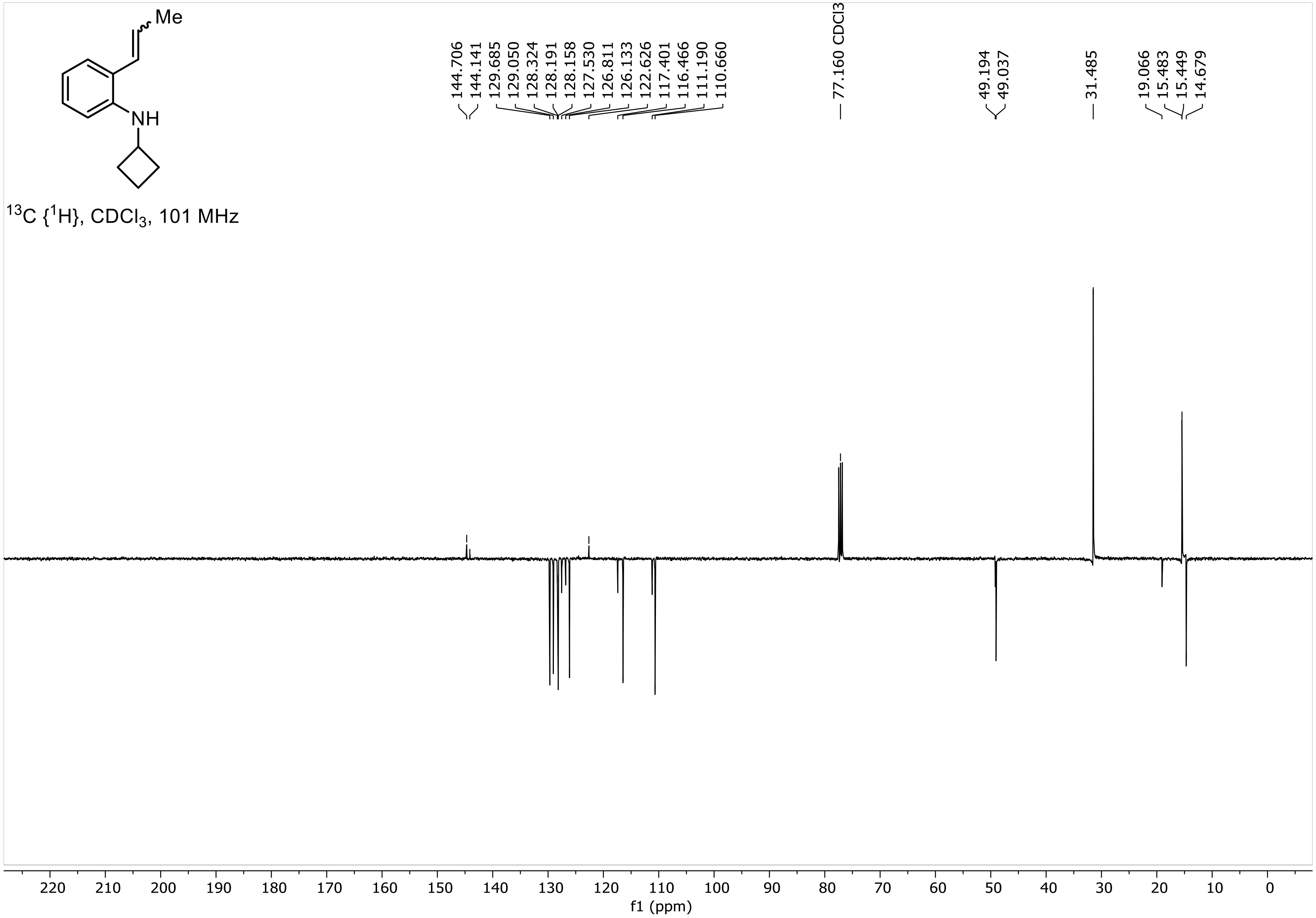
Me
NH
13C {1H}, CDCl3, 101 MHz
144.706
144.141
129.685
129.050
128.324
128.191
128.158
127.530
126.811
126.133
122.626
117.401
116.466
111.190
110.660
77.160 CDCl3
49.194
49.037
31.485
19.066
15.483
15.449
14.679
220 210 200 190 180 170 160 150 140 130 120 110 100 90 80 70 60 50 40 30 20 10 0
f1 (ppm)

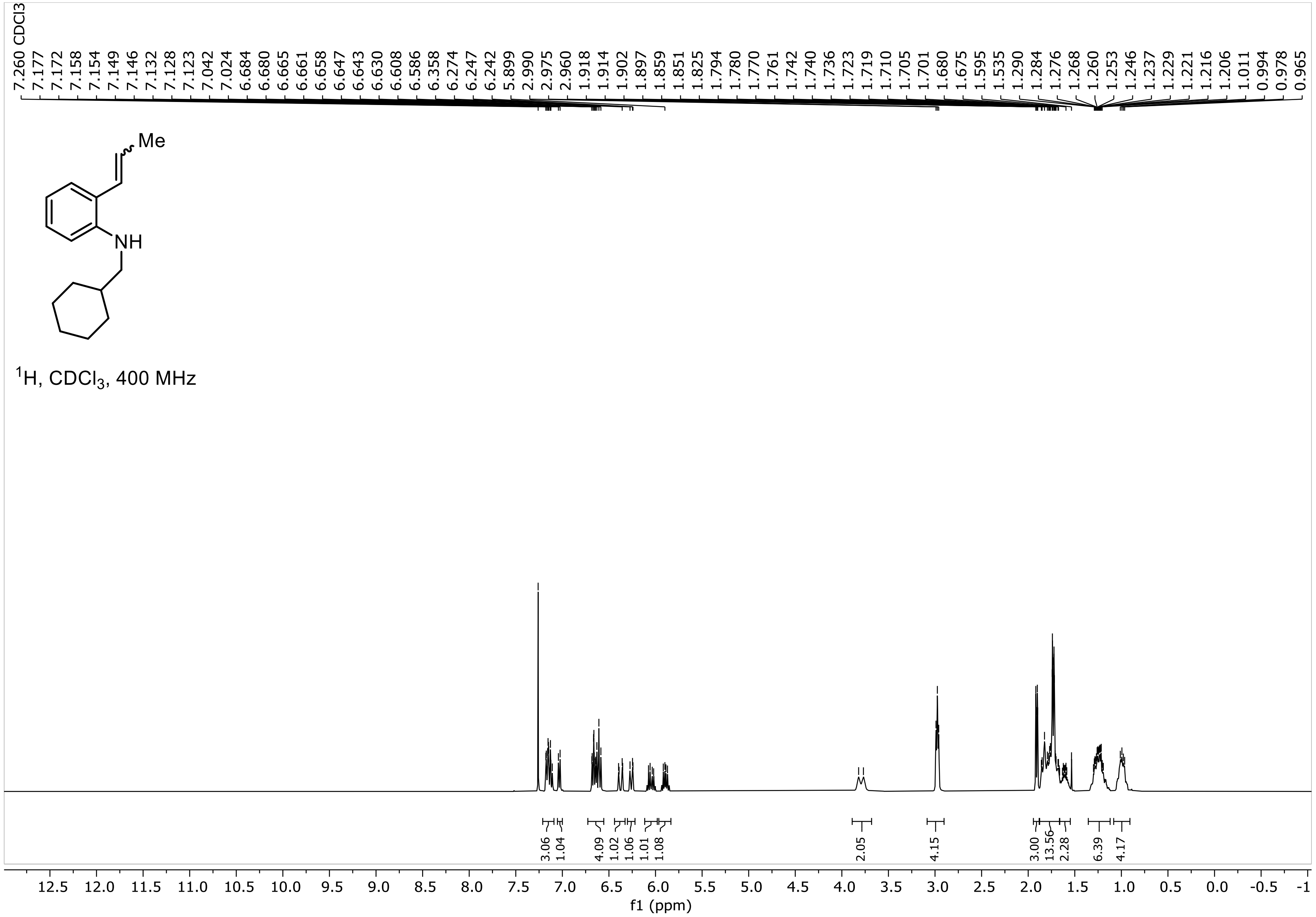
7.260 CDCl3
7.177 7.172 7.158 7.154 7.149 7.146 7.132 7.128 7.123 7.042 7.024 6.684 6.680 6.665 6.661 6.658 6.647 6.643 6.630 6.608 6.586 6.358 6.274 6.247 6.242 5.899 2.990 2.975 2.960 1.918 1.914 1.902 1.897 1.859 1.851 1.825 1.794 1.780 1.770 1.761 1.742 1.740 1.736 1.723 1.719 1.710 1.705 1.701 1.680 1.675 1.595 1.535 1.290 1.284 1.276 1.268 1.260 1.253 1.246 1.237 1.229 1.221 1.216 1.206 1.011 0.994 0.978 0.965
Me
NH
¹H, CDCl3, 400 MHz
3.06 1.04 4.09 1.02 1.06 1.01 1.08 2.05 4.15 3.00 13.56 2.28 6.39 4.17
12.5 12.0 11.5 11.0 10.5 10.0 9.5 9.0 8.5 8.0 7.5 7.0 6.5 6.0 5.5 5.0 4.5 4.0 3.5 3.0 2.5 2.0 1.5 1.0 0.5 0.0 -0.5 -1
f1 (ppm)

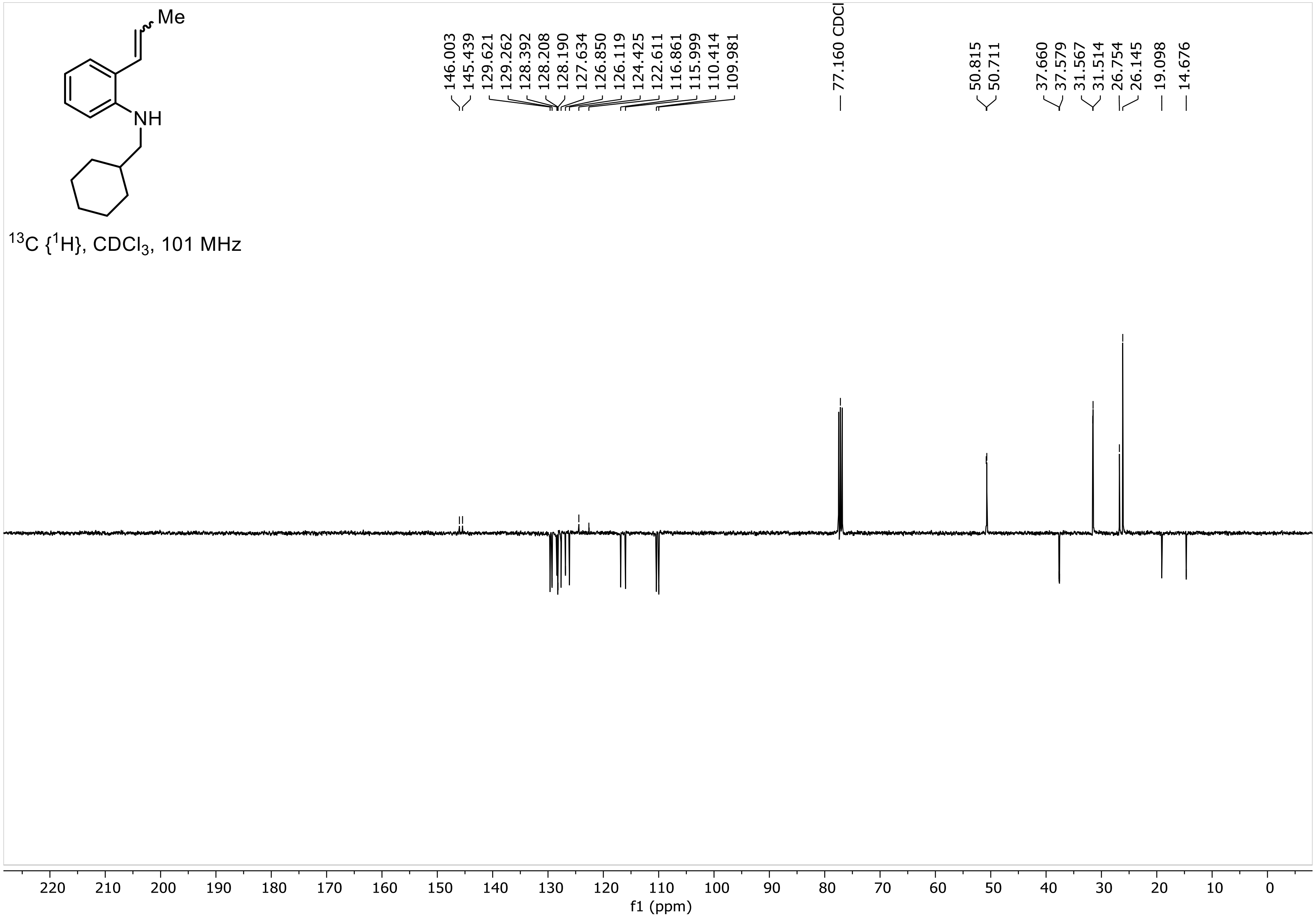

Me
NH
$^{13}C\ \{^{1}H\}$, $CDCl_3$, 101 MHz
146.003
145.439
129.621
129.262
128.392
128.208
128.190
127.634
126.850
126.119
124.425
122.611
116.861
115.999
110.414
109.981
77.160 CDCl
50.815
50.711
37.660
37.579
31.567
31.514
26.754
26.145
19.098
14.676
220 210 200 190 180 170 160 150 140 130 120 110 100 90 80 70 60 50 40 30 20 10 0
f1 (ppm)

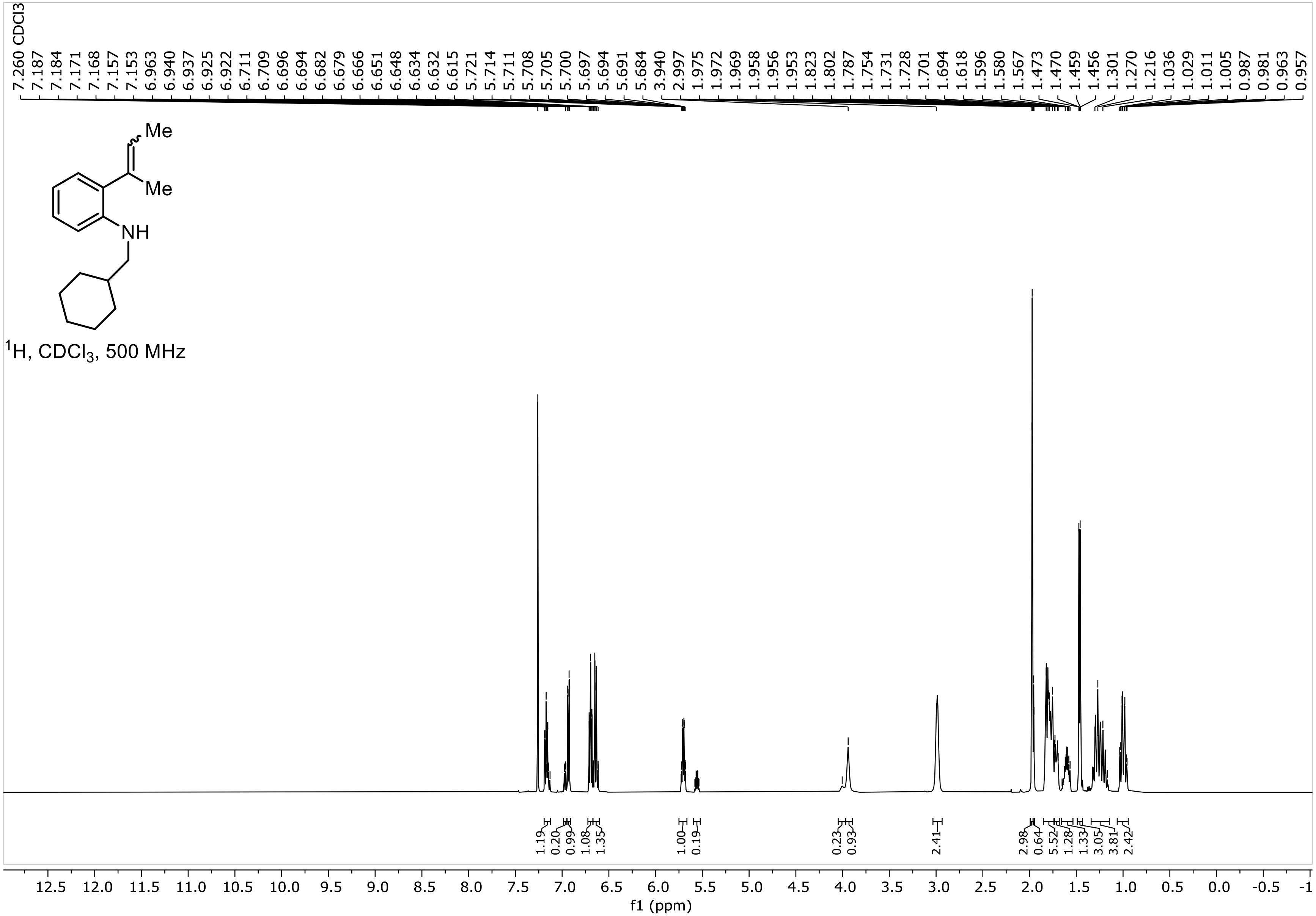
7.260 CDCl3
7.187 7.184 7.171 7.168 7.157 7.153 6.963 6.940 6.937 6.925 6.922 6.711 6.709 6.696 6.694 6.682 6.679 6.666 6.651 6.648 6.634 6.632 6.615 5.721 5.714 5.711 5.708 5.705 5.700 5.697 5.694 5.691 5.684 3.940 2.997 1.975 1.972 1.969 1.958 1.956 1.953 1.823 1.802 1.787 1.754 1.731 1.728 1.701 1.694 1.618 1.596 1.580 1.567 1.473 1.470 1.459 1.456 1.301 1.270 1.216 1.036 1.029 1.011 1.005 0.987 0.981 0.963 0.957
Me
Me
NH
1H, CDCl3, 500 MHz
1.19 0.20 0.99 1.08 1.35 1.00 0.19 0.23 0.93 2.41 2.98 0.64 5.52 1.28 1.33 3.05 3.81 2.42
12.5 12.0 11.5 11.0 10.5 10.0 9.5 9.0 8.5 8.0 7.5 7.0 6.5 6.0 5.5 5.0 4.5 4.0 3.5 3.0 2.5 2.0 1.5 1.0 0.5 0.0 -0.5 -1
f1 (ppm)

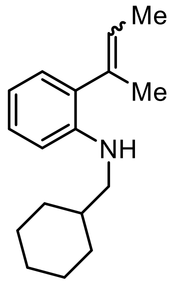

$^{13}C\{^1H\}$, $CDCl_3$, 126 MHz

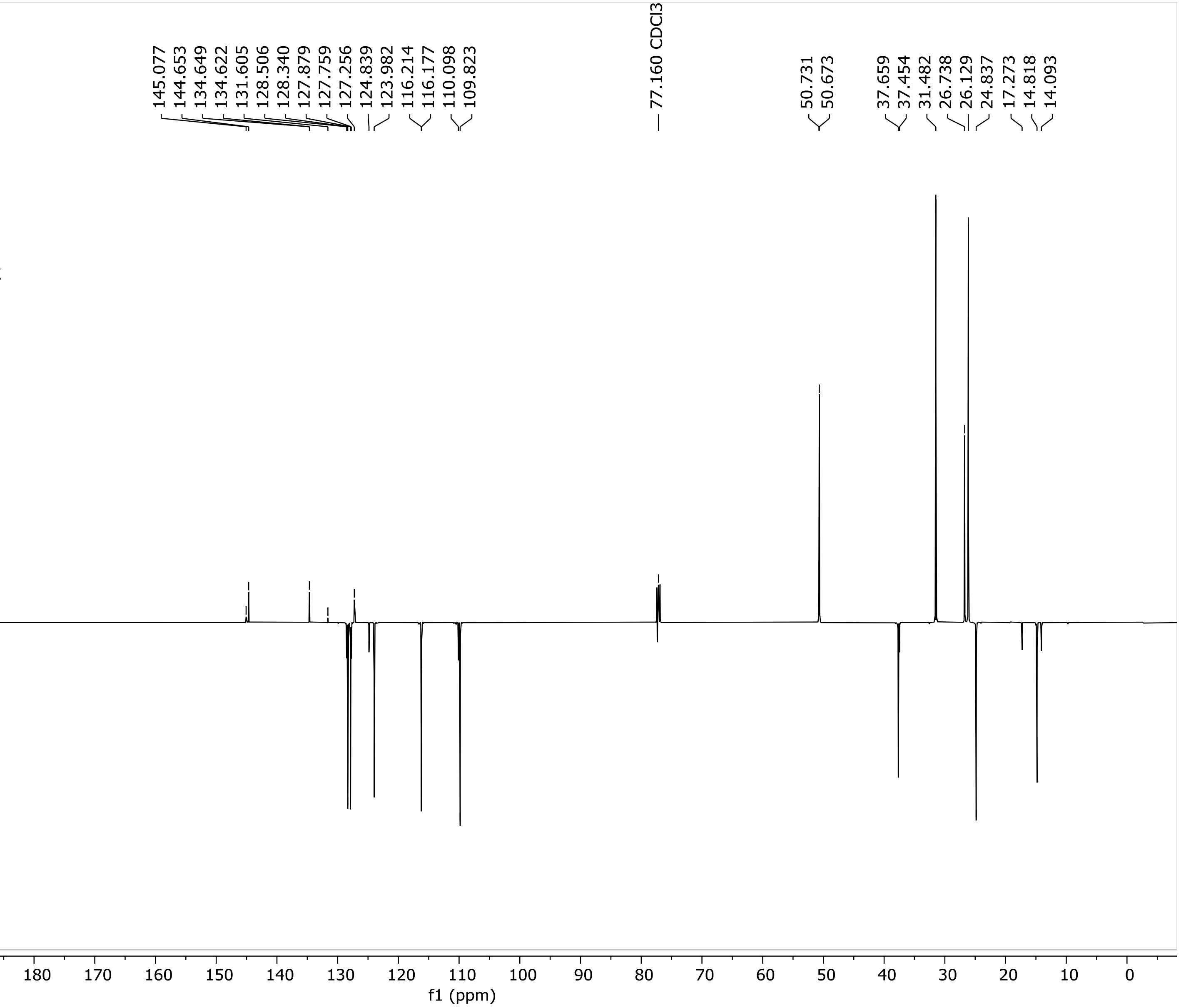

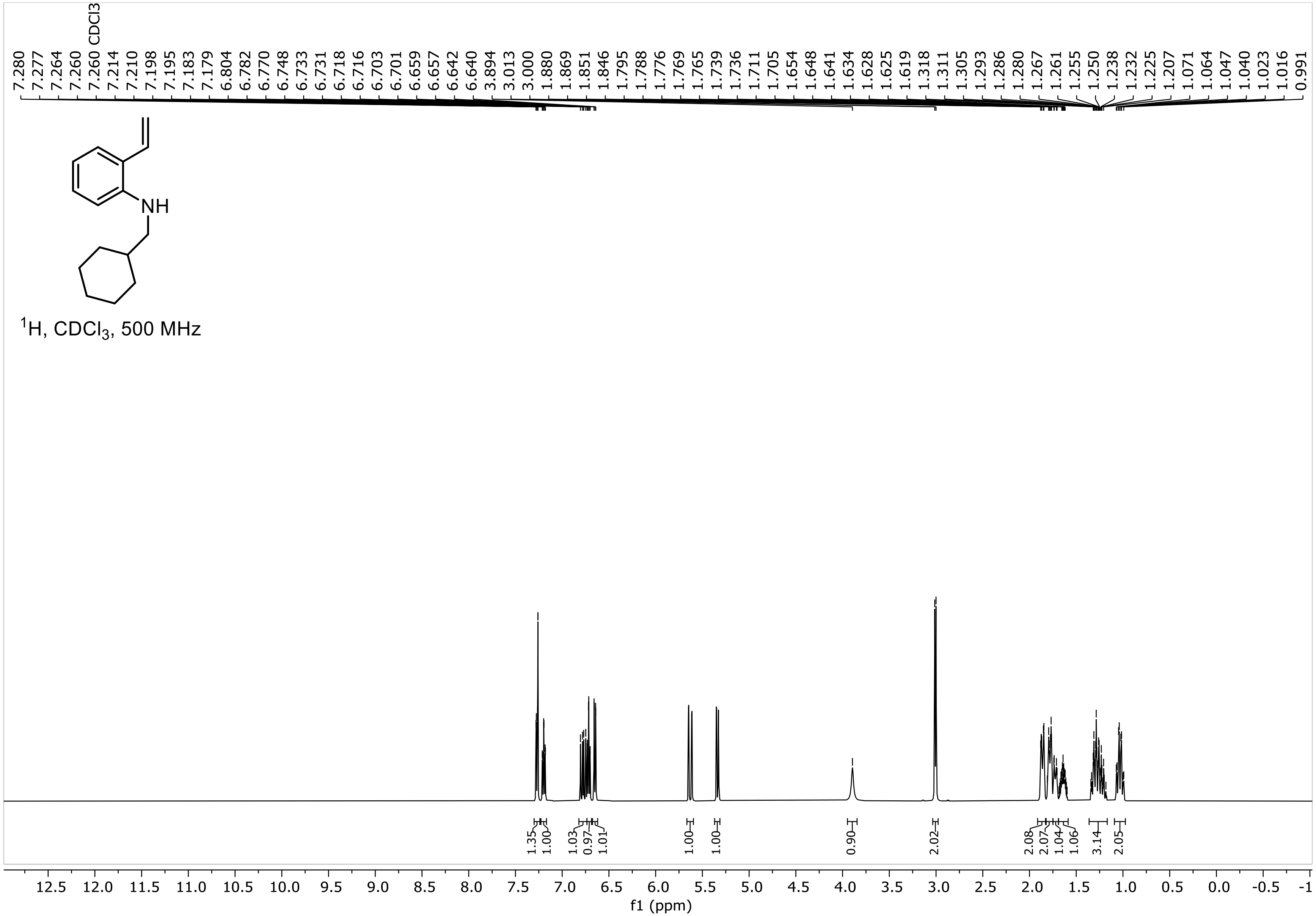

1H, CDCl3, 500 MHz
NH
f1 (ppm)

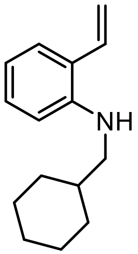

$^{13}C\ \{^1H\}$, $CDCl_3$, 126 MHz

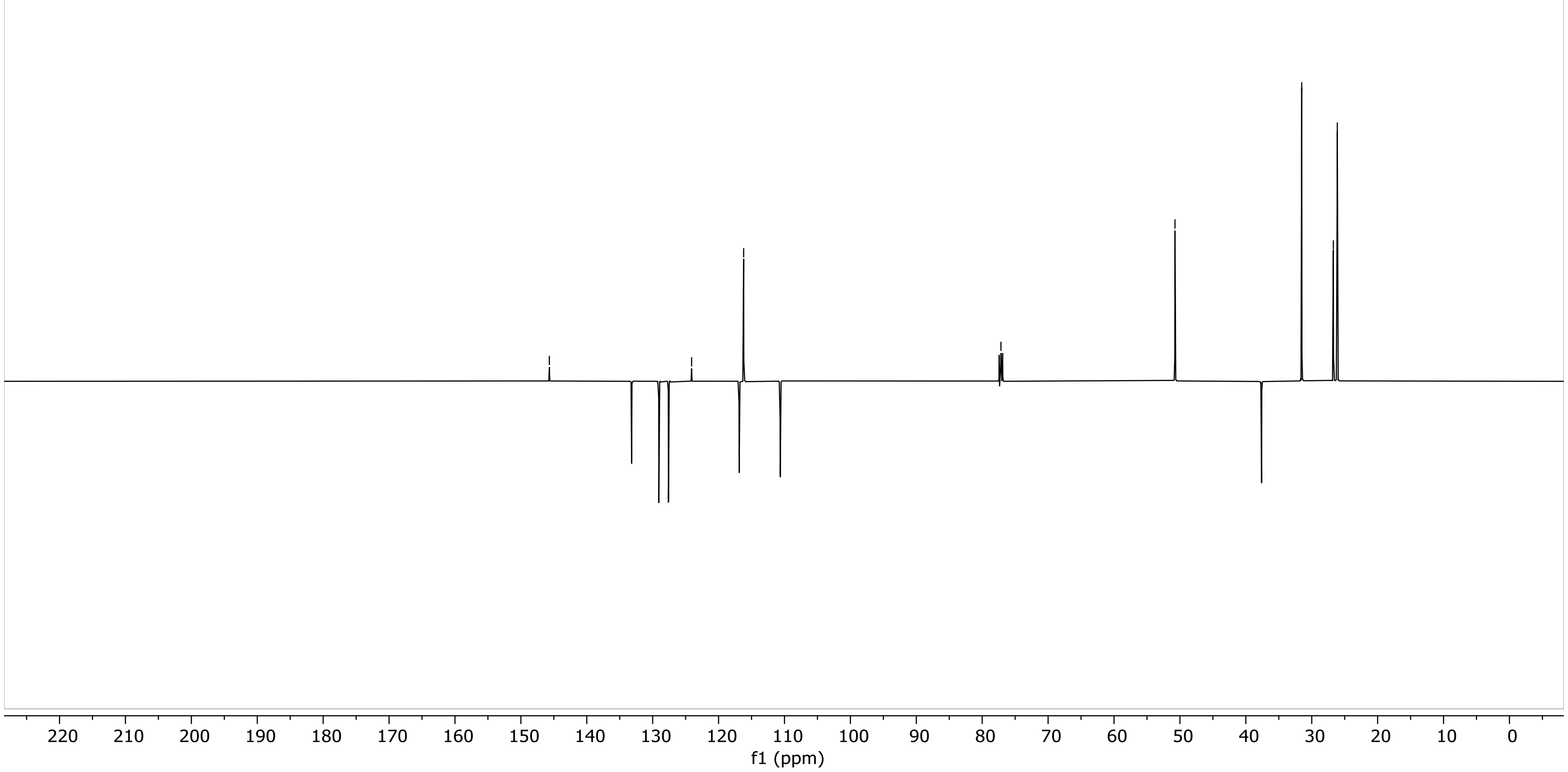

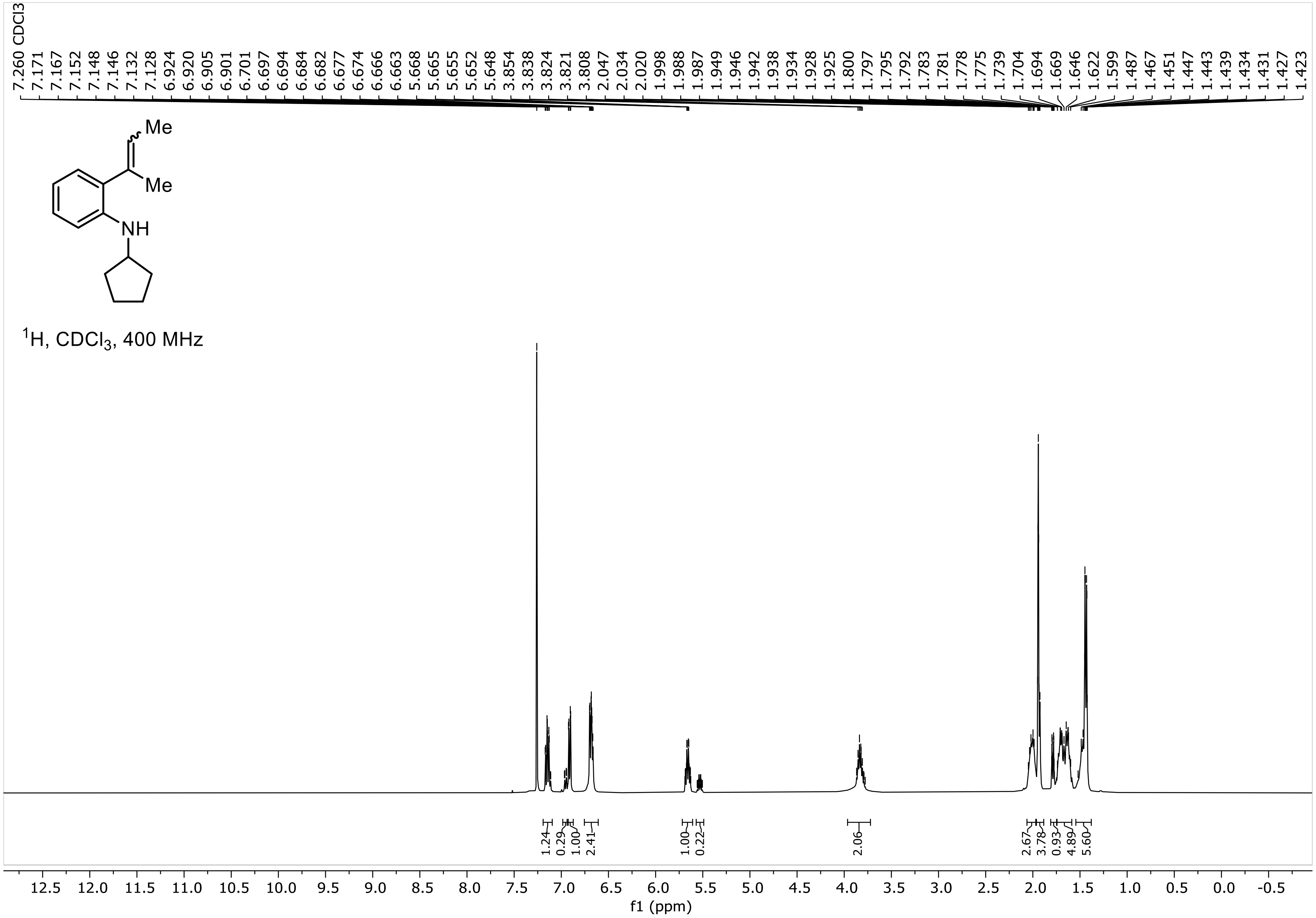

Me
Me
NH
¹H, CDCl₃, 400 MHz
7.260 CDCl3
f1 (ppm)

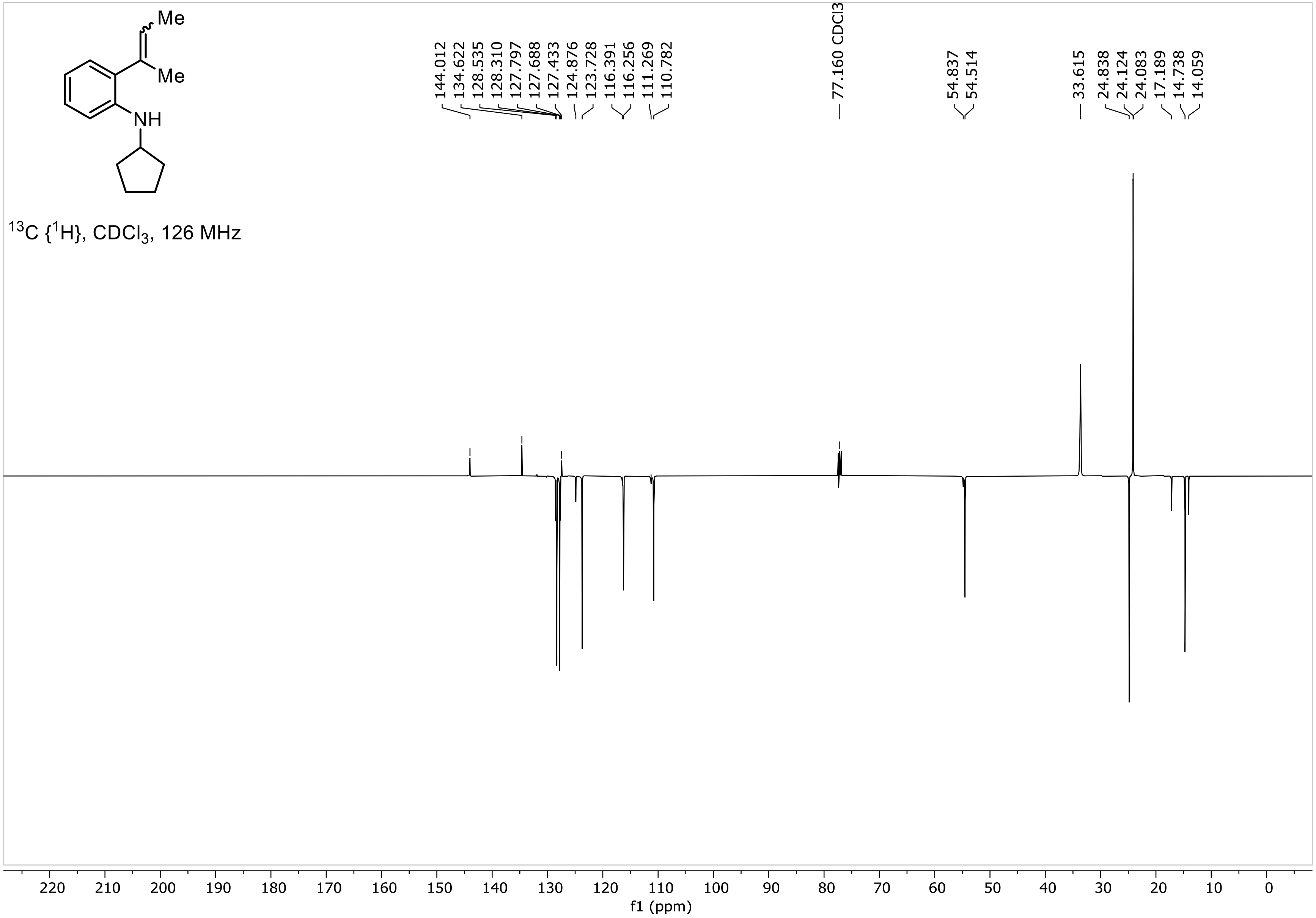
Me
Me
NH
$^{13}C\{^1H\}$, $CDCl_3$, 126 MHz
144.012
134.622
128.535
128.310
127.797
127.688
127.433
124.876
123.728
116.391
116.256
111.269
110.782
77.160 CDCl3
54.837
54.514
33.615
24.838
24.124
24.083
17.189
14.738
14.059
220 210 200 190 180 170 160 150 140 130 120 110 100 90 80 70 60 50 40 30 20 10 0
f1 (ppm)

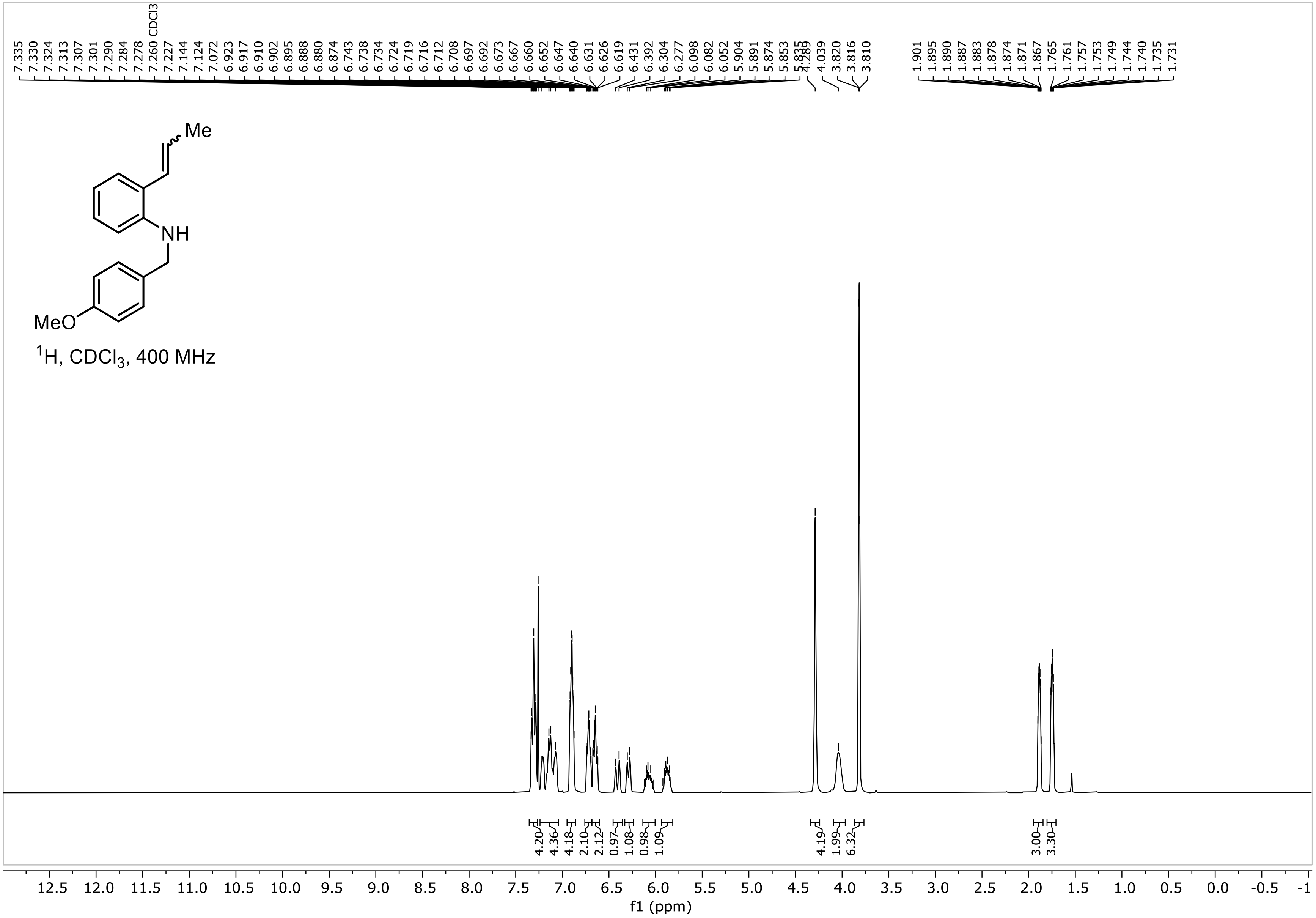

Me
NH
MeO
^{1}H, CDCl$_3$, 400 MHz
7.335 7.330 7.324 7.313 7.307 7.301 7.290 7.284 7.278 7.260 CDCl3 7.227 7.144 7.124 7.072 6.923 6.917 6.910 6.902 6.895 6.888 6.880 6.874 6.743 6.738 6.734 6.724 6.719 6.716 6.712 6.708 6.697 6.692 6.673 6.667 6.660 6.652 6.647 6.640 6.631 6.626 6.619 6.431 6.392 6.304 6.277 6.098 6.082 6.052 5.904 5.891 5.874 5.853 4.039 3.820 3.816 3.810
1.901 1.895 1.890 1.887 1.883 1.878 1.874 1.871 1.867 1.765 1.761 1.757 1.753 1.749 1.744 1.740 1.735 1.731
4.20 4.36 4.18 2.10 2.12 0.97 1.08 0.98 1.09 4.19 1.99 6.32 3.00 3.30
12.5 12.0 11.5 11.0 10.5 10.0 9.5 9.0 8.5 8.0 7.5 7.0 6.5 6.0 5.5 5.0 4.5 4.0 3.5 3.0 2.5 2.0 1.5 1.0 0.5 0.0 -0.5 -1
f1 (ppm)

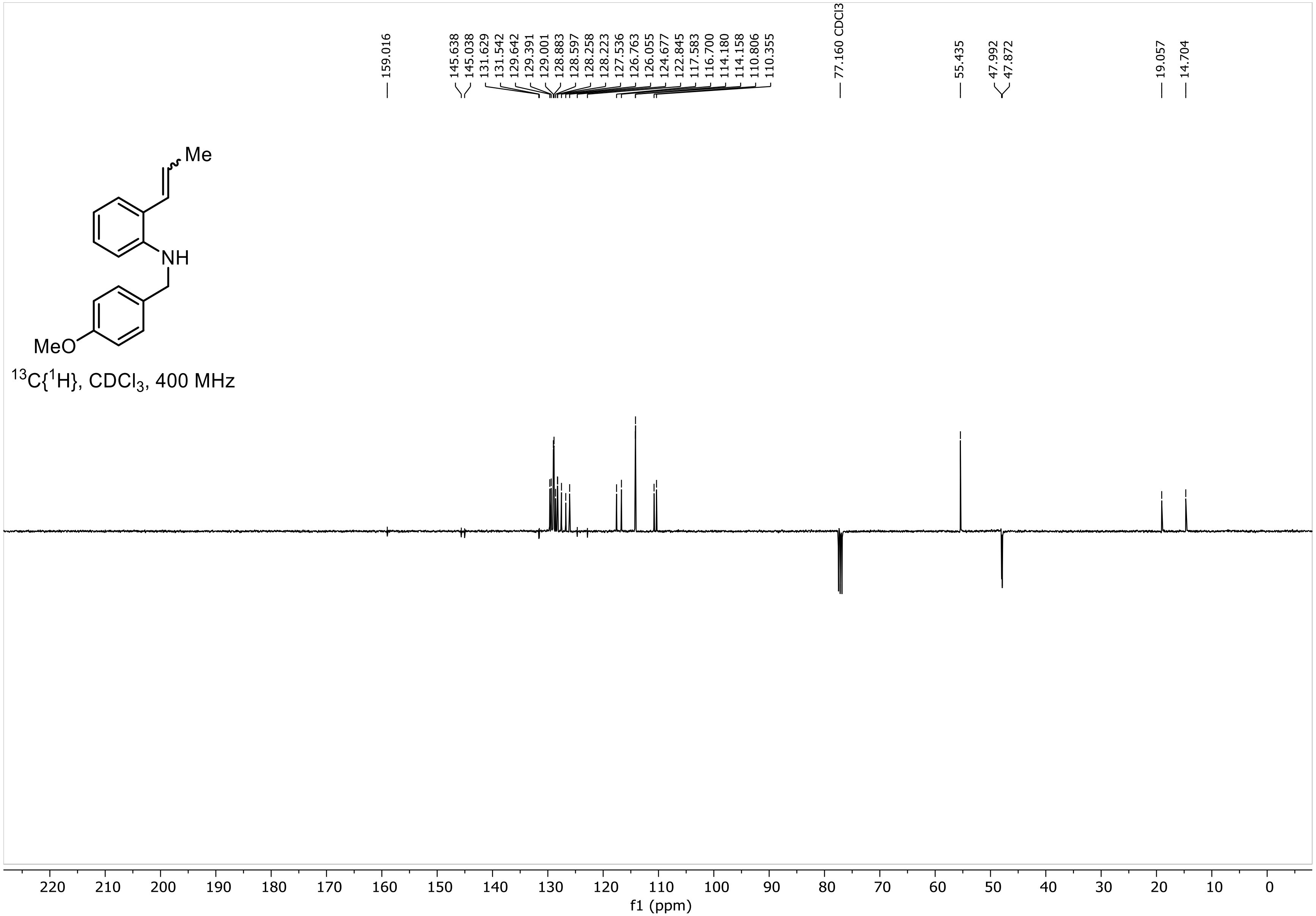

159.016
145.638
145.038
131.629
131.542
129.642
129.391
129.001
128.883
128.597
128.258
128.223
127.536
126.763
126.055
124.677
122.845
117.583
116.700
114.180
114.158
110.806
110.355
77.160 CDCl3
55.435
47.992
47.872
19.057
14.704
Me
NH
MeO
$^{13}C\{^1H\}$, $CDCl_3$, 400 MHz
220 210 200 190 180 170 160 150 140 130 120 110 100 90 80 70 60 50 40 30 20 10 0
f1 (ppm)

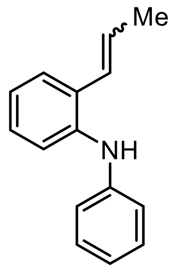

^{1}H, $CDCl_3$, 400 MHz

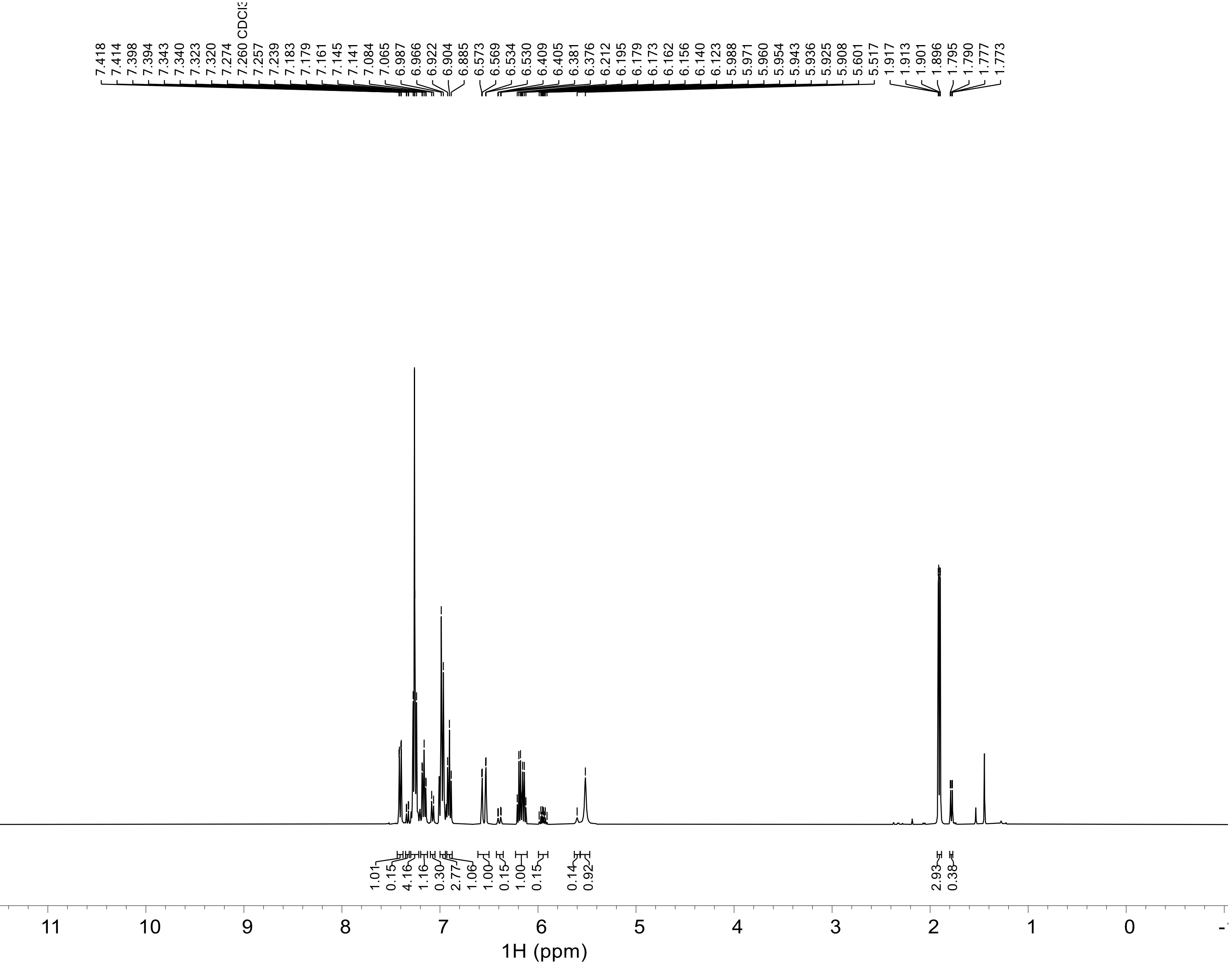

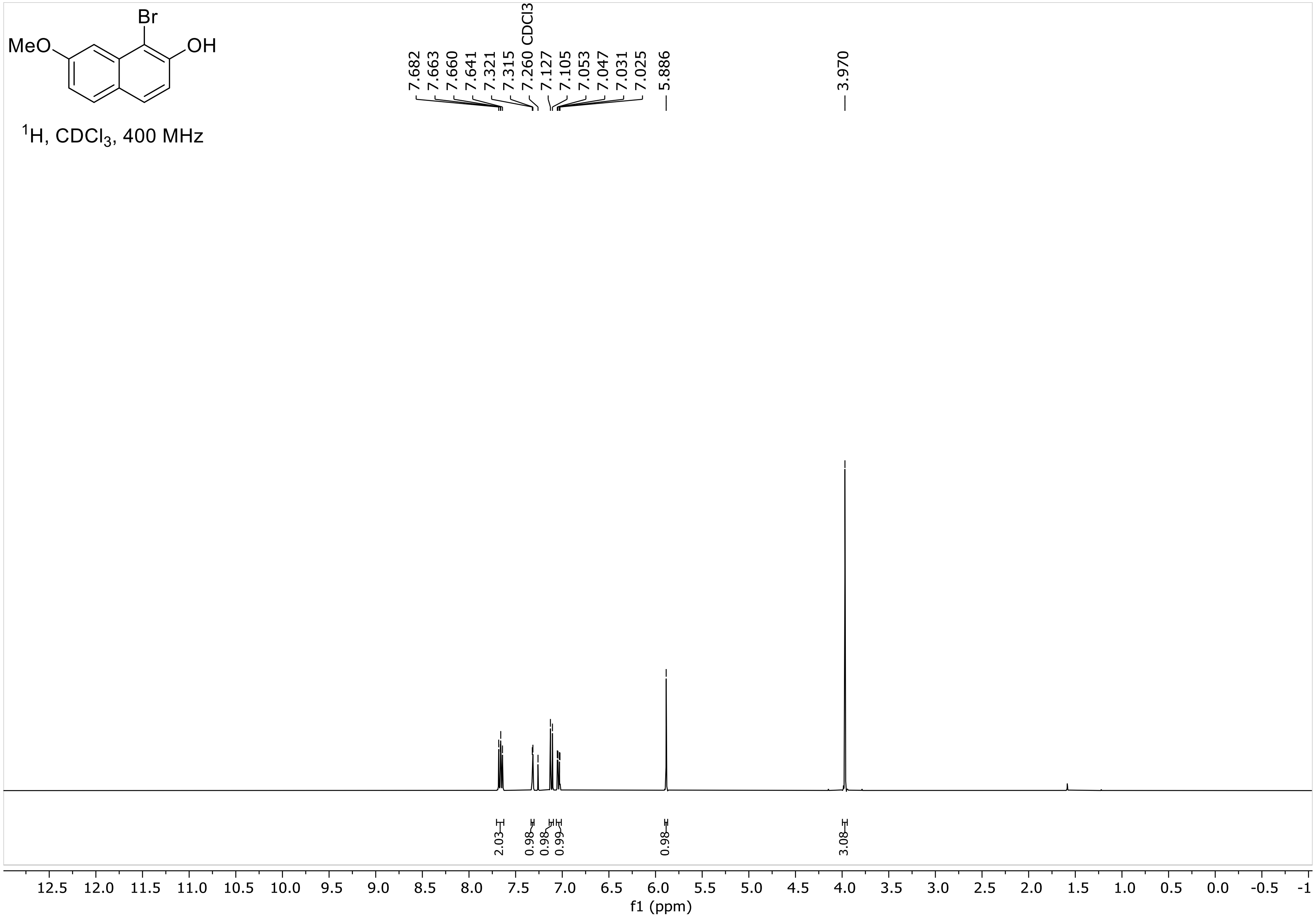

Br
MeO
OH
^{1}H, CDCl$_3$, 400 MHz
7.682
7.663
7.660
7.641
7.321
7.315
7.260 CDCl3
7.127
7.105
7.053
7.047
7.031
7.025
5.886
3.970
2.03
0.98
0.98
0.99
0.98
3.08
12.5 12.0 11.5 11.0 10.5 10.0 9.5 9.0 8.5 8.0 7.5 7.0 6.5 6.0 5.5 5.0 4.5 4.0 3.5 3.0 2.5 2.0 1.5 1.0 0.5 0.0 -0.5 -1
f1 (ppm)

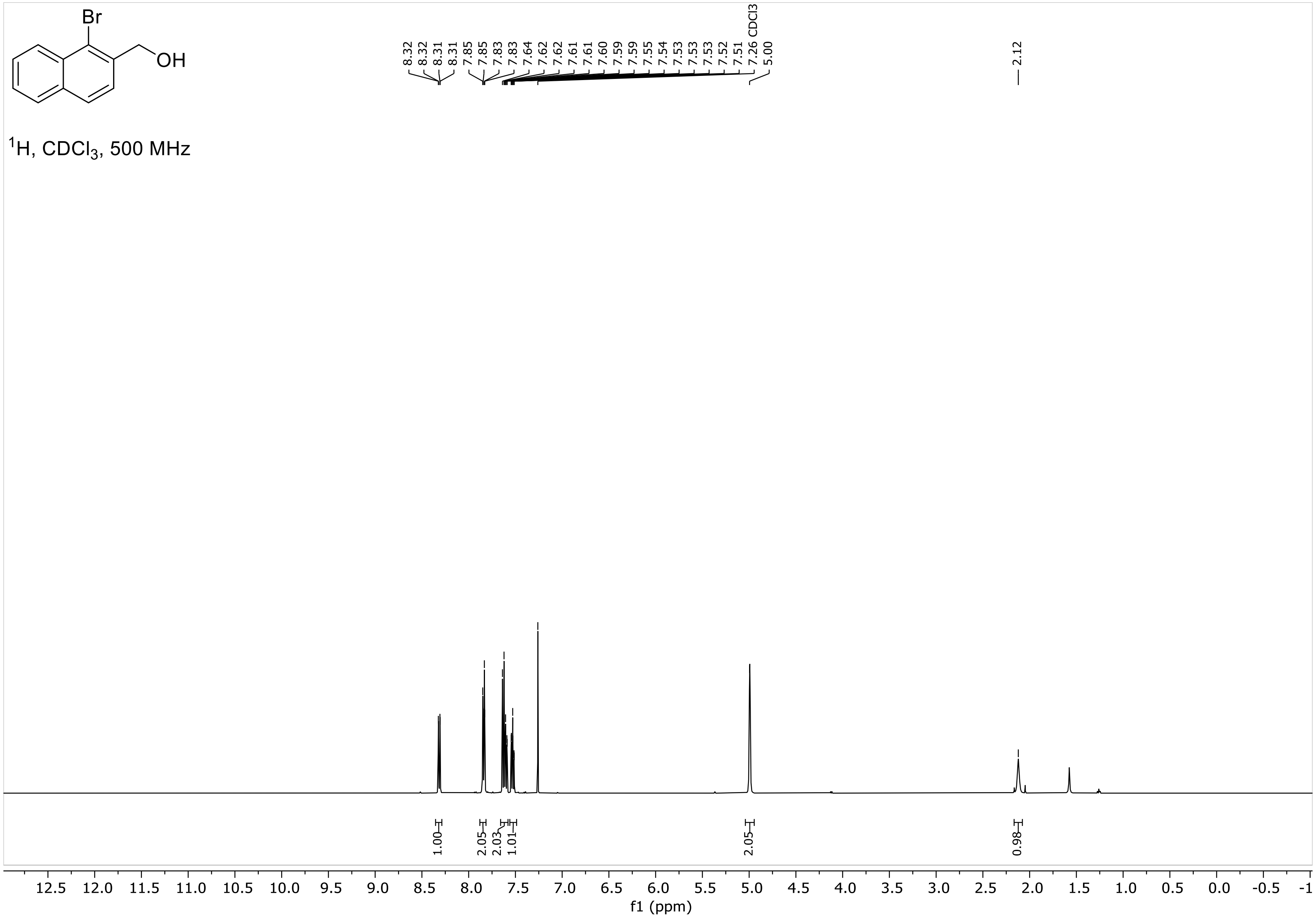
Br
OH
^{1}H, CDCl$_3$, 500 MHz
8.32
8.32
8.31
8.31
7.85
7.85
7.83
7.83
7.64
7.62
7.61
7.61
7.60
7.59
7.59
7.55
7.54
7.53
7.53
7.53
7.52
7.51
7.26 CDCl3
5.00
2.12
1.00
2.05
2.03
1.01
2.05
0.98
12.5 12.0 11.5 11.0 10.5 10.0 9.5 9.0 8.5 8.0 7.5 7.0 6.5 6.0 5.5 5.0 4.5 4.0 3.5 3.0 2.5 2.0 1.5 1.0 0.5 0.0 -0.5 -1
f1 (ppm)

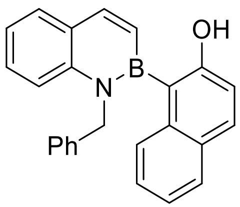

^{1}H, $CDCl_3$, 700 MHz

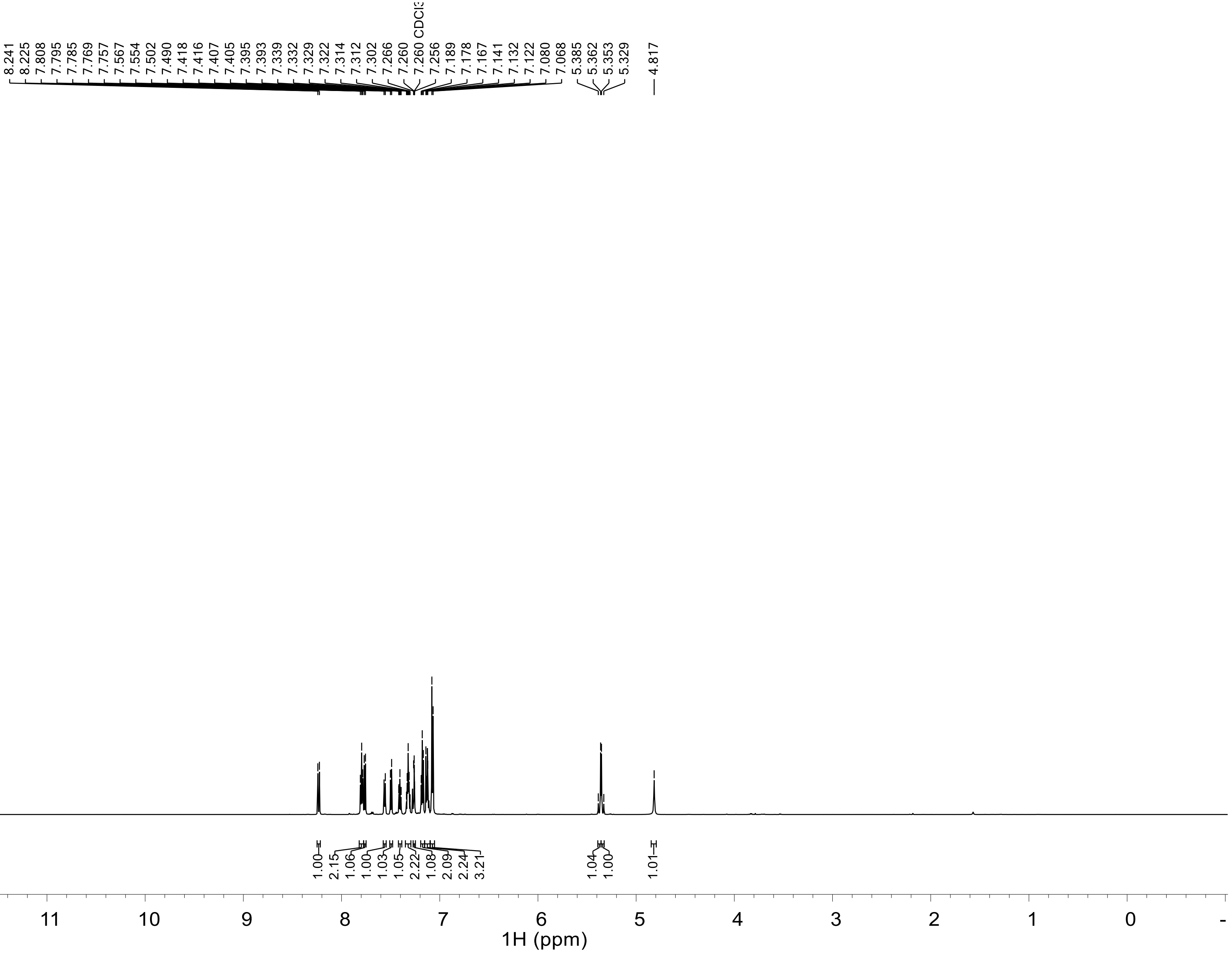

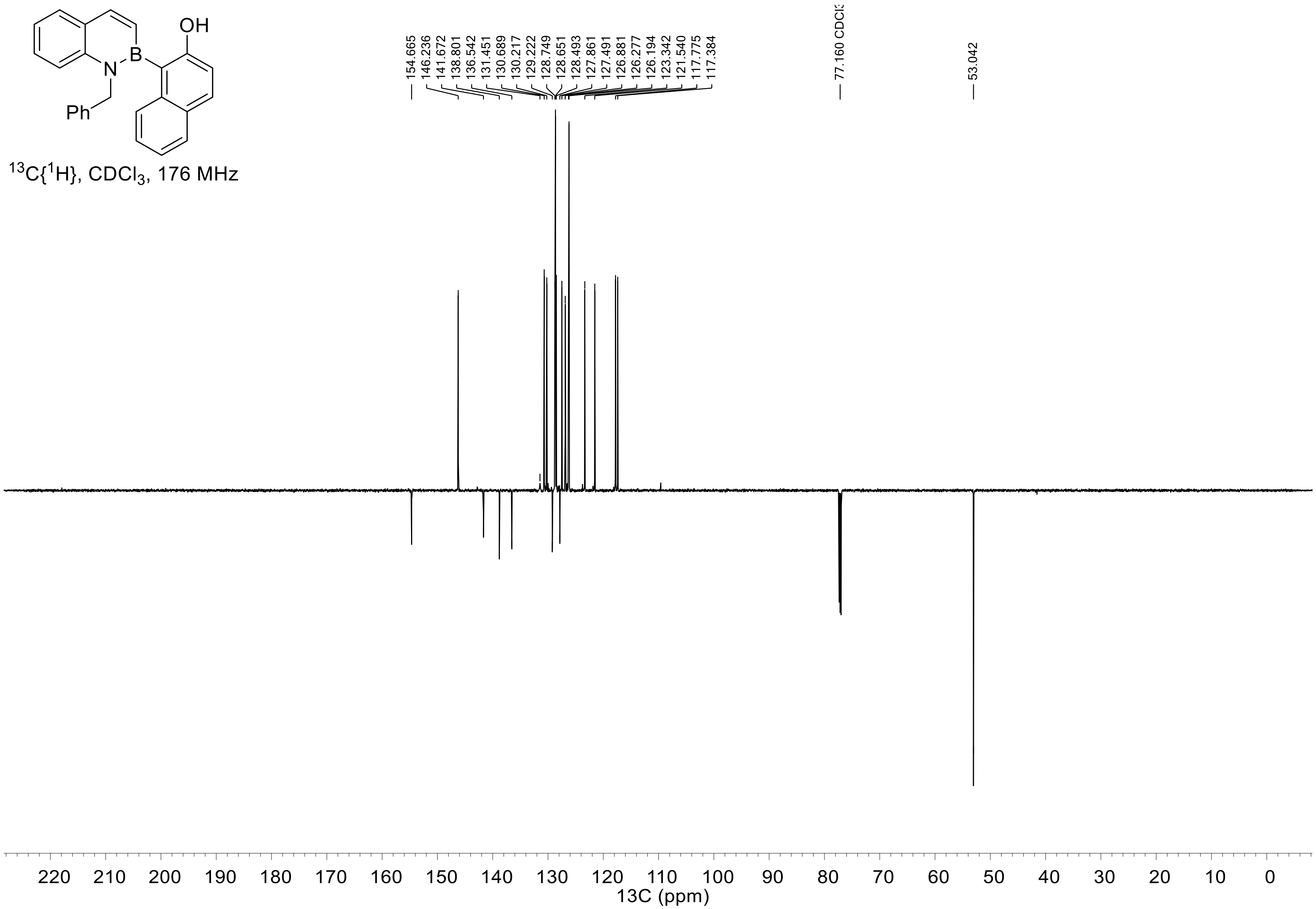
OH
B
N
Ph
$^{13}C\{^1H\}$, $CDCl_3$, 176 MHz
154.665
146.236
141.672
138.801
136.542
131.451
130.689
130.217
129.222
128.749
128.651
128.493
127.861
127.491
126.881
126.277
126.194
123.342
121.540
117.775
117.384
77.160 CDCl3
53.042
220 210 200 190 180 170 160 150 140 130 120 110 100 90 80 70 60 50 40 30 20 10 0
13C (ppm)

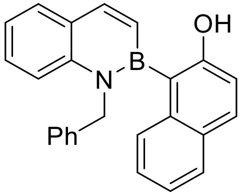

$^{11}B\ \{^{1}H\}$, $CDCl_3$, 128 MHz

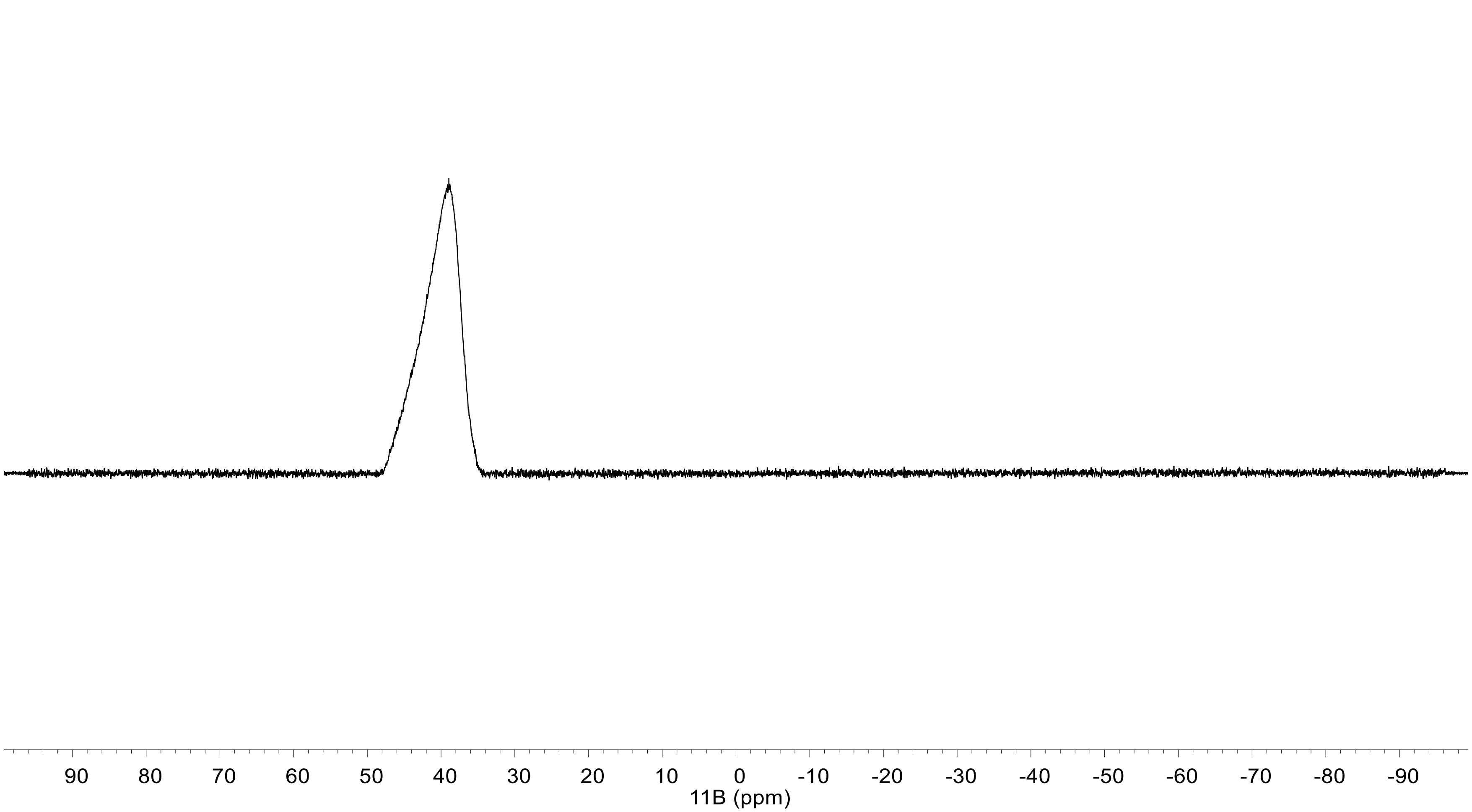

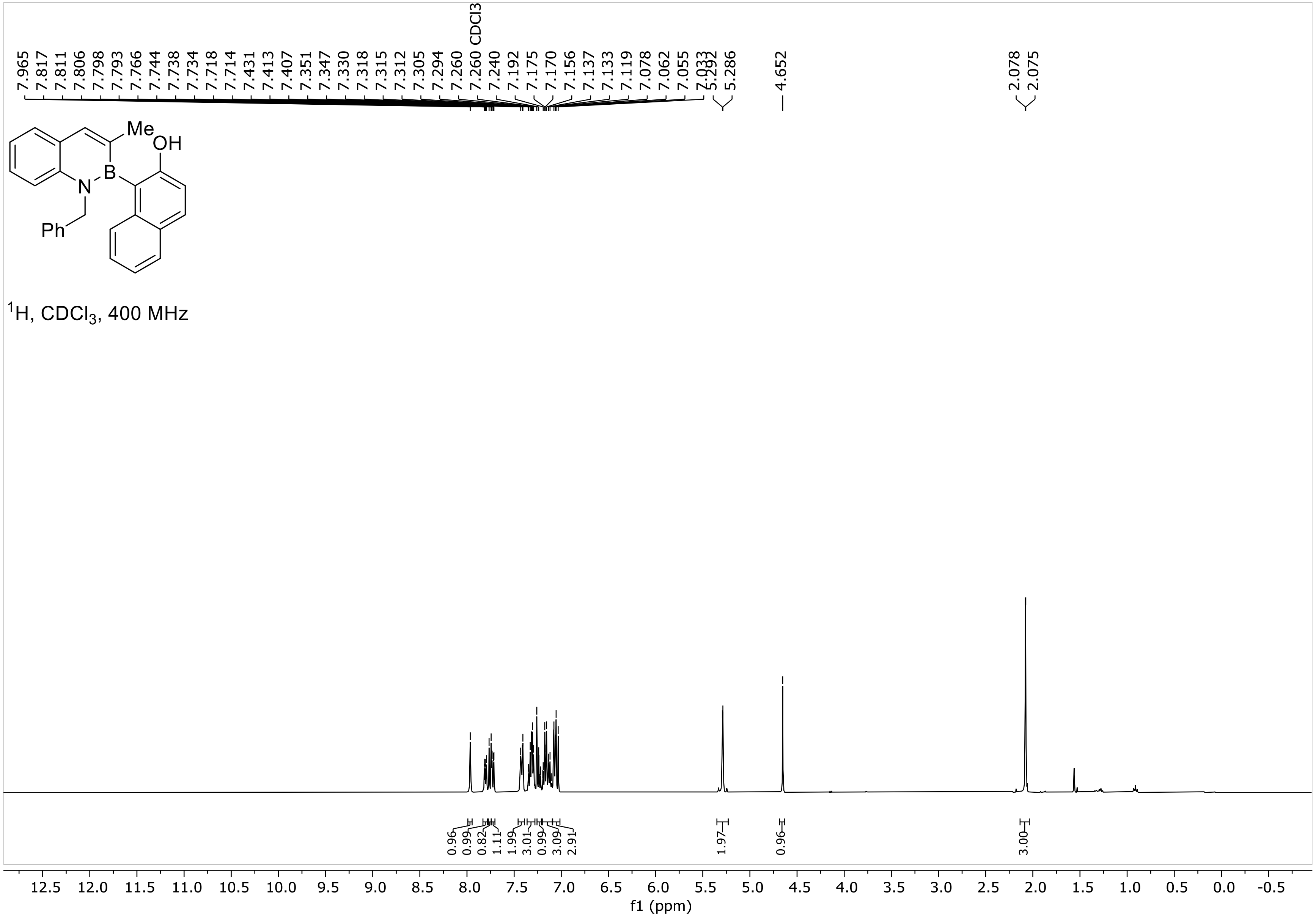

^{1}H, $CDCl_3$, 400 MHz

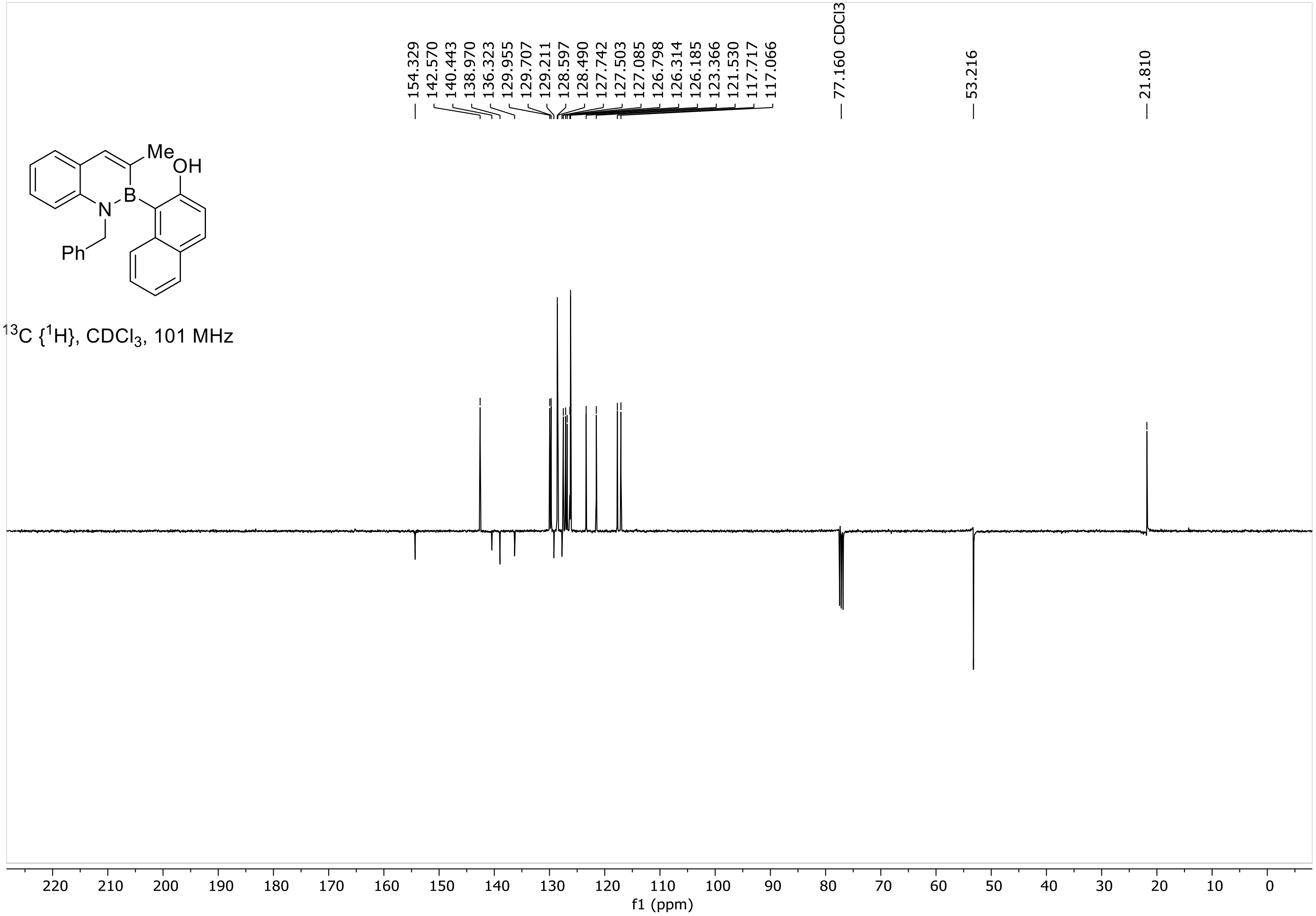

$^{13}C\ \{^1H\}$, $CDCl_3$, 101 MHz

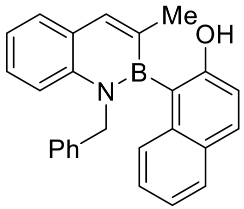

$^{11}B\ \{^1H\}$, $CDCl_3$, 128 MHz

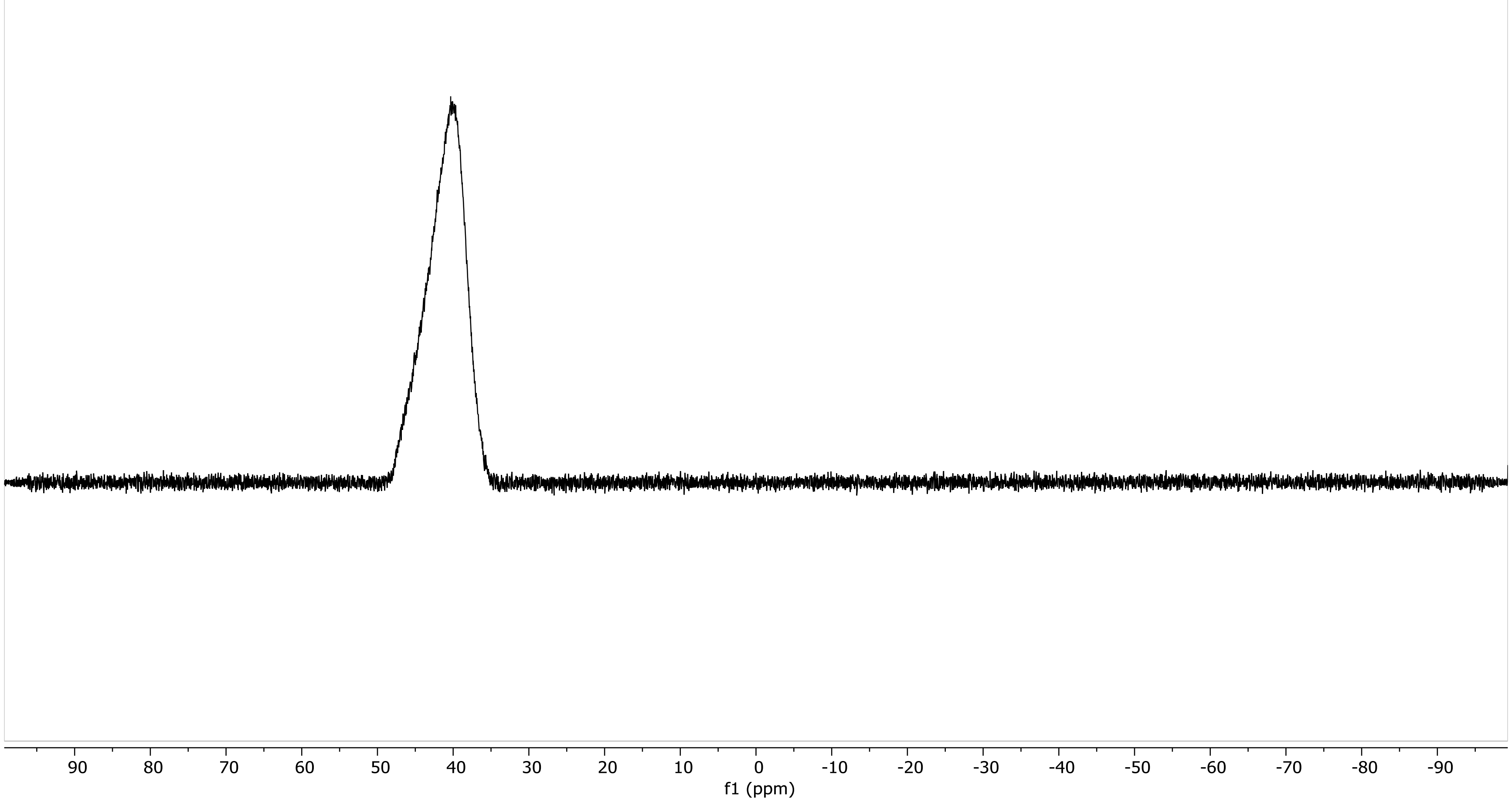

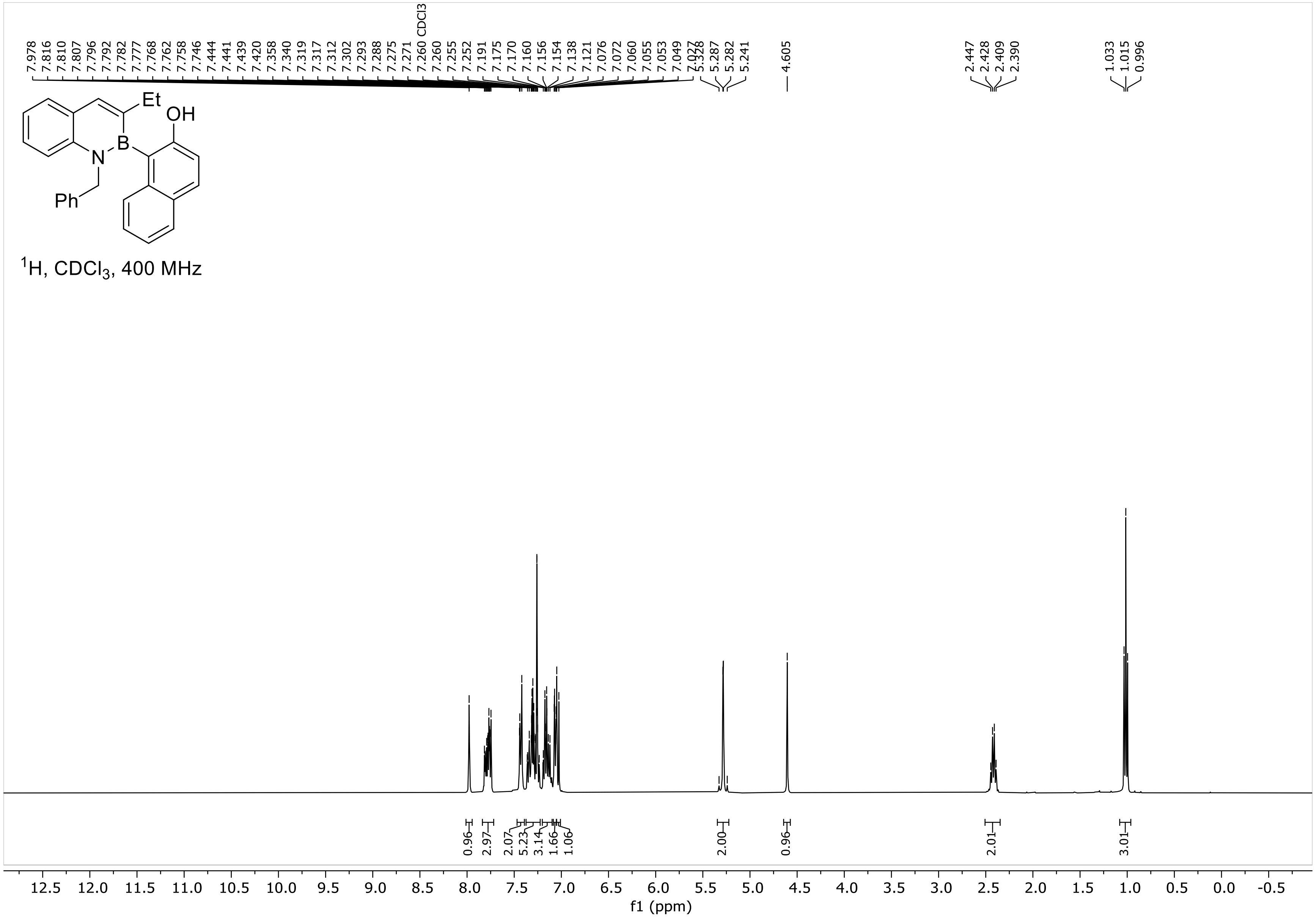

7.978 7.816 7.810 7.807 7.796 7.792 7.782 7.777 7.768 7.762 7.758 7.746 7.444 7.441 7.439 7.420 7.358 7.340 7.319 7.317 7.312 7.302 7.293 7.288 7.275 7.271 7.260 CDCl3 7.260 7.255 7.252 7.191 7.175 7.170 7.160 7.156 7.154 7.138 7.121 7.076 7.072 7.060 7.055 7.053 7.049
5.328 5.287 5.282 5.241
4.605
2.447 2.428 2.409 2.390
1.033 1.015 0.996
Et
OH
B
N
Ph
1H, CDCl3, 400 MHz
0.96 2.97 2.07 5.23 3.14 1.66 1.06 2.00 0.96 2.01 3.01
12.5 12.0 11.5 11.0 10.5 10.0 9.5 9.0 8.5 8.0 7.5 7.0 6.5 6.0 5.5 5.0 4.5 4.0 3.5 3.0 2.5 2.0 1.5 1.0 0.5 0.0 -0.5
f1 (ppm)

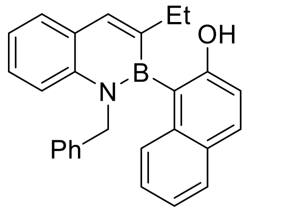

$^{13}C\{^1H\}$, $CDCl_3$, 126 MHz

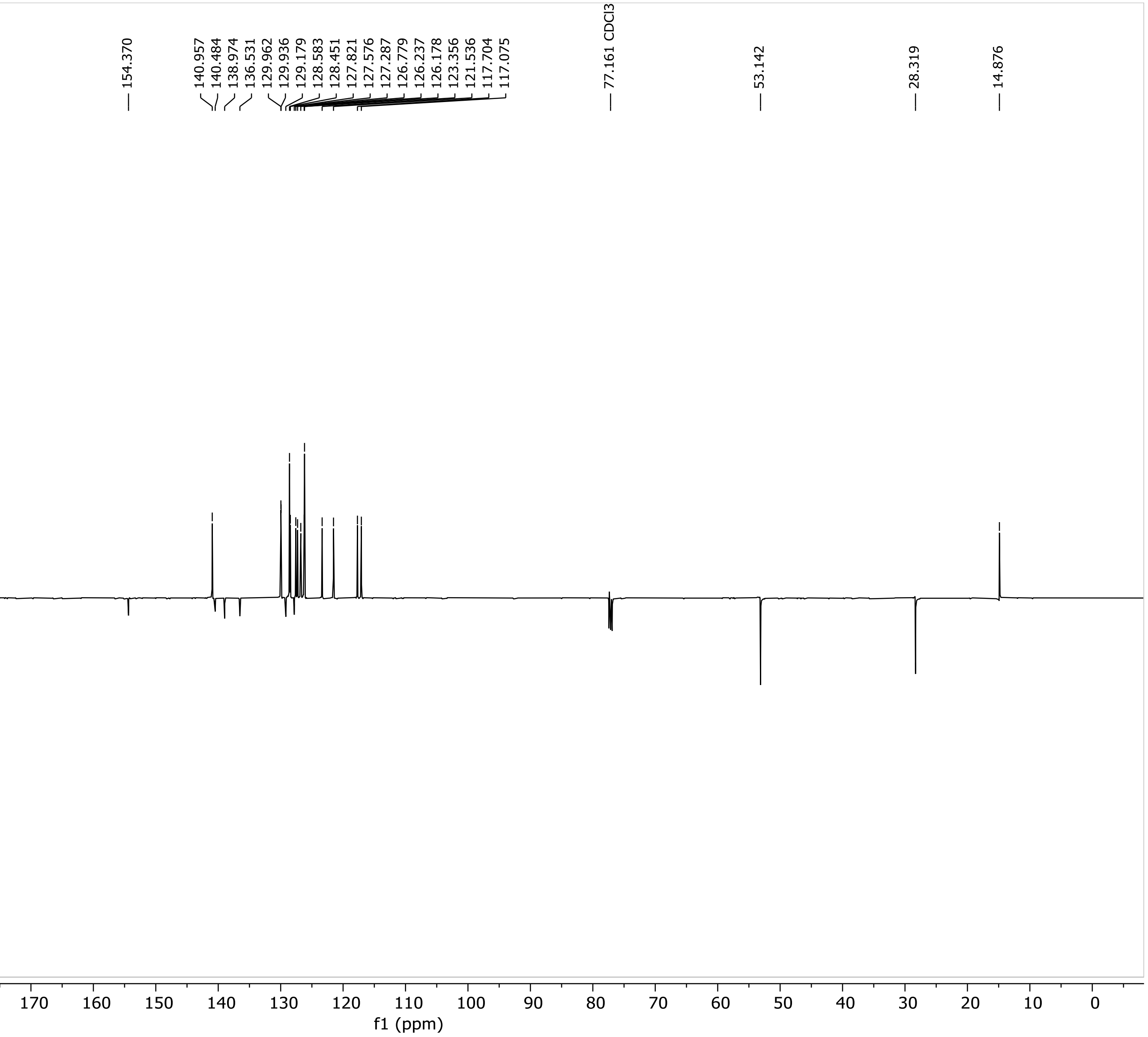

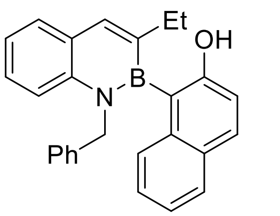

$^{11}B\ \{^{1}H\}$, $CDCl_3$, 128 MHz

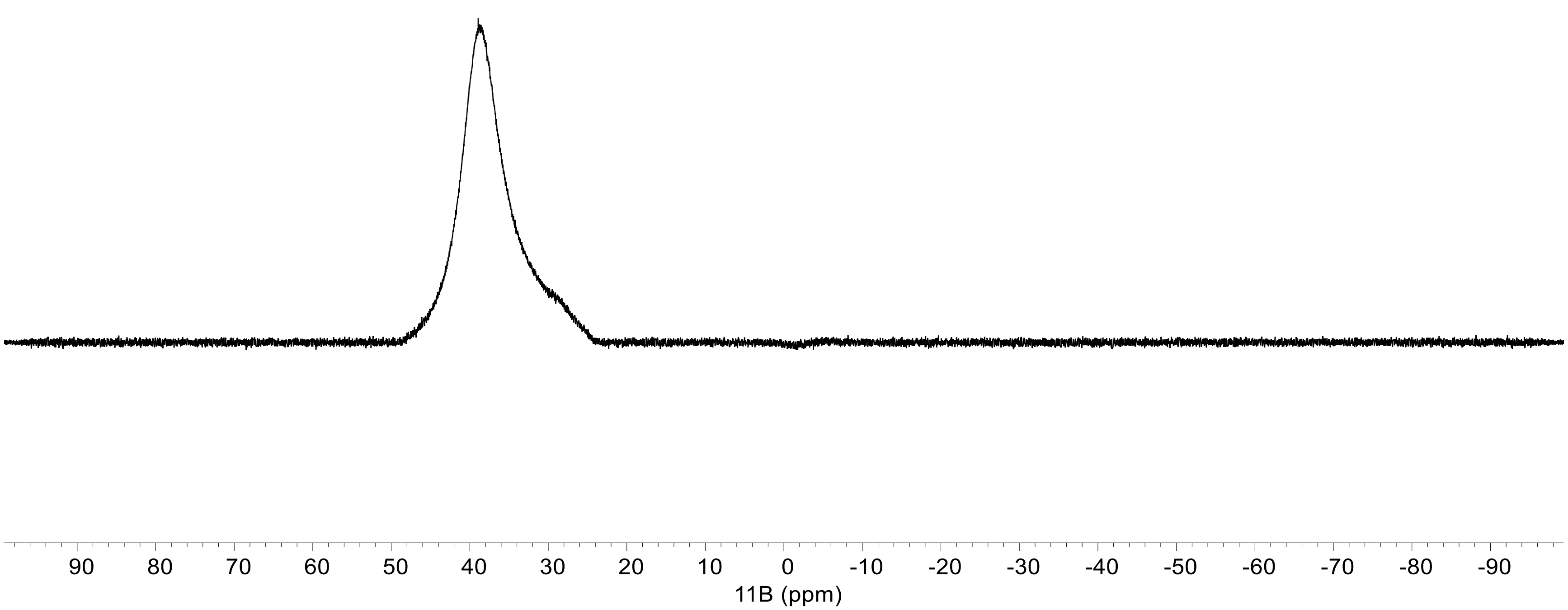

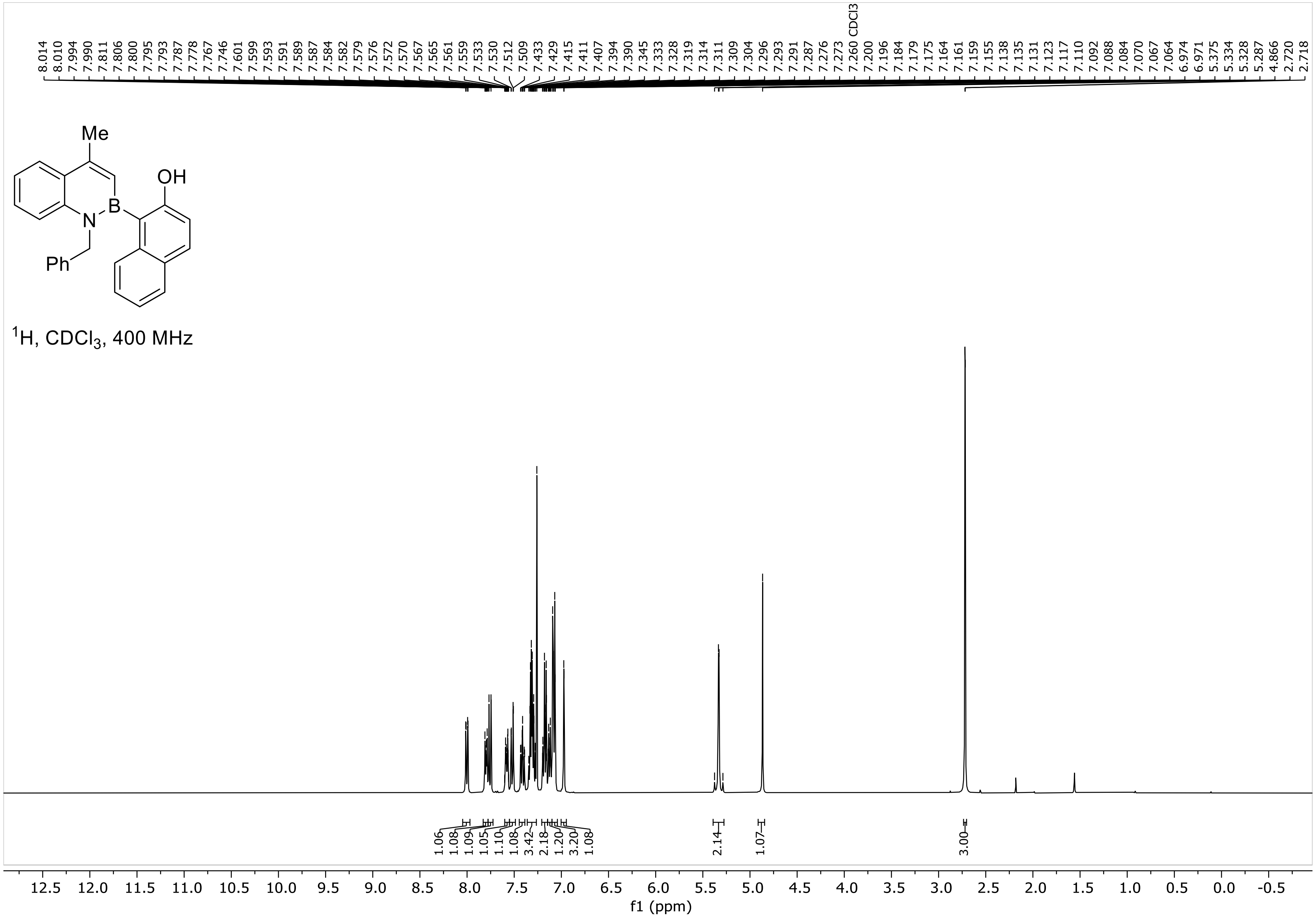
8.014
8.010
7.994
7.990
7.811
7.806
7.800
7.795
7.793
7.787
7.778
7.767
7.746
7.601
7.599
7.593
7.591
7.589
7.587
7.584
7.582
7.579
7.576
7.572
7.570
7.567
7.565
7.561
7.559
7.533
7.530
7.512
7.509
7.433
7.429
7.415
7.411
7.407
7.394
7.390
7.345
7.333
7.328
7.319
7.314
7.311
7.309
7.304
7.296
7.293
7.291
7.287
7.276
7.273
7.260 CDCl3
7.200
7.196
7.184
7.179
7.175
7.164
7.161
7.159
7.155
7.138
7.135
7.131
7.123
7.117
7.110
7.092
7.088
7.084
7.070
7.067
7.064
6.974
6.971
5.375
5.334
5.328
5.287
4.866
2.720
2.718
Me
OH
B
N
Ph
^{1}H, CDCl$_3$, 400 MHz
1.06
1.08
1.09
1.05
1.10
1.08
3.42
2.18
1.20
3.20
1.08
2.14
1.07
3.00
12.5 12.0 11.5 11.0 10.5 10.0 9.5 9.0 8.5 8.0 7.5 7.0 6.5 6.0 5.5 5.0 4.5 4.0 3.5 3.0 2.5 2.0 1.5 1.0 0.5 0.0 -0.5
f1 (ppm)

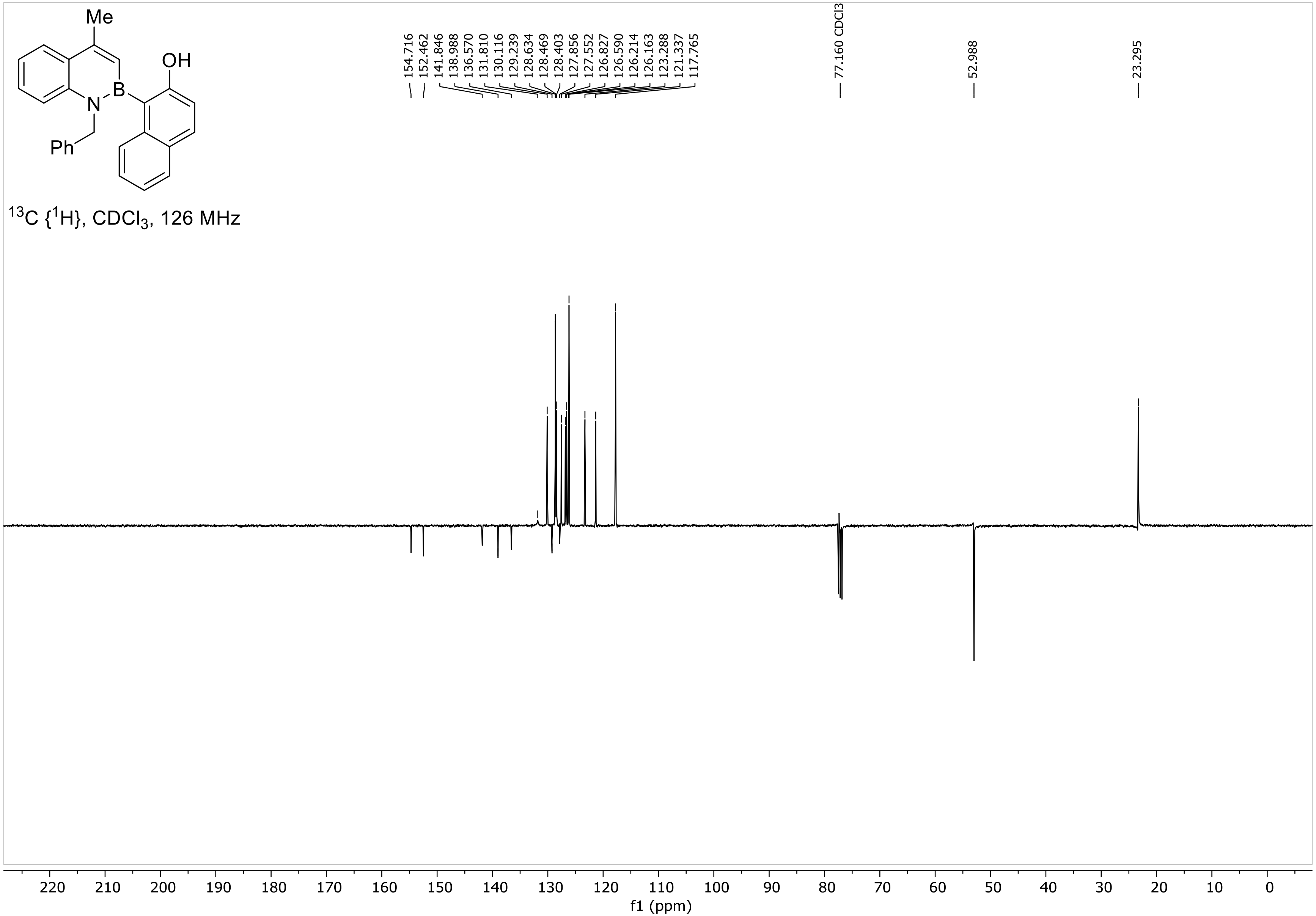
Me
OH
N
B
Ph
13C {1H}, CDCl3, 126 MHz
154.716
152.462
141.846
138.988
136.570
131.810
130.116
129.239
128.634
128.469
128.403
127.856
127.552
126.827
126.590
126.214
126.163
123.288
121.337
117.765
77.160 CDCl3
52.988
23.295
220 210 200 190 180 170 160 150 140 130 120 110 100 90 80 70 60 50 40 30 20 10 0
f1 (ppm)

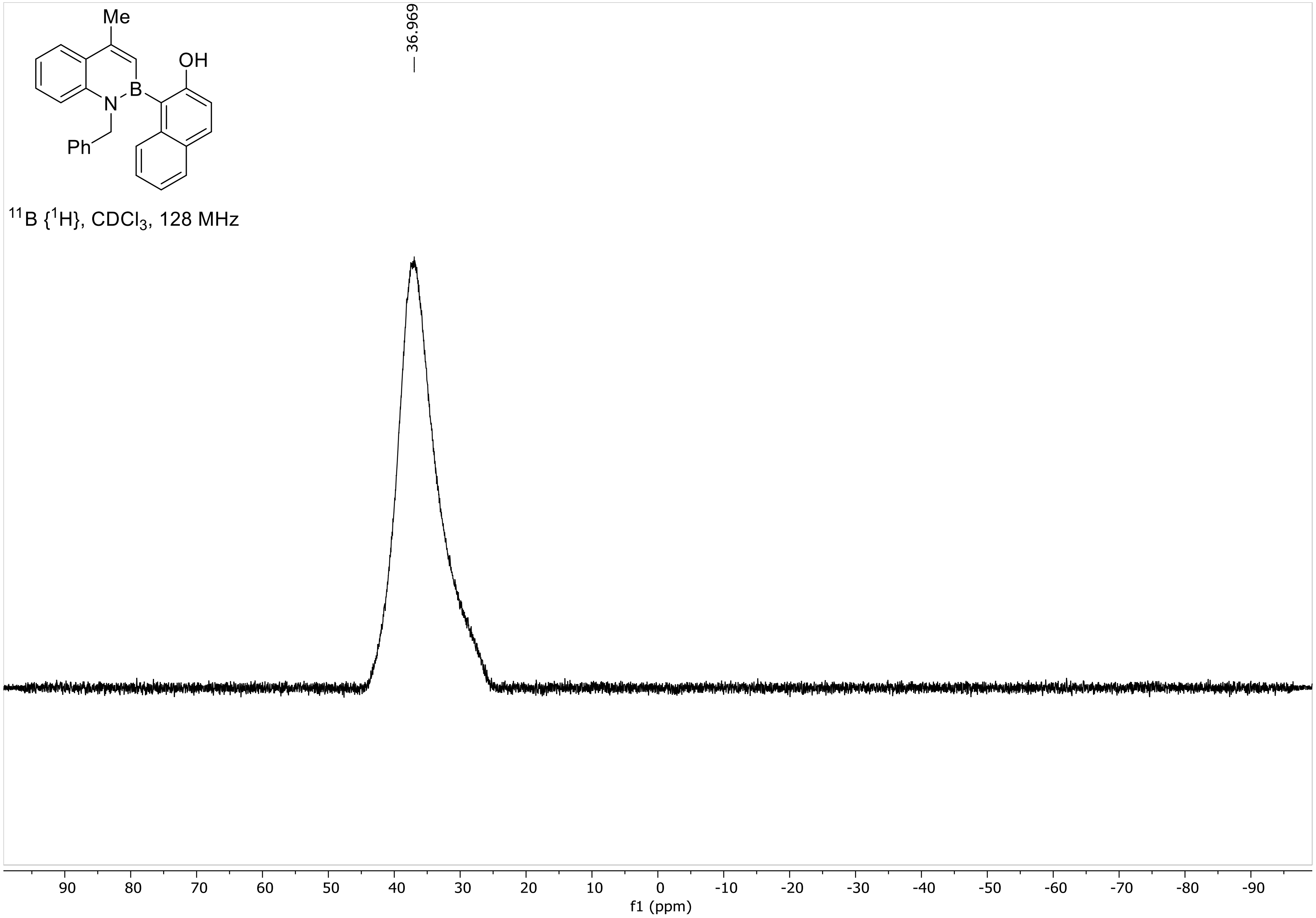
Me
OH
N
B
Ph
$^{11}B\ \{^1H\}$, $CDCl_3$, 128 MHz
36.969
90
80
70
60
50
40
30
20
10
0
-10
-20
-30
-40
-50
-60
-70
-80
-90
f1 (ppm)

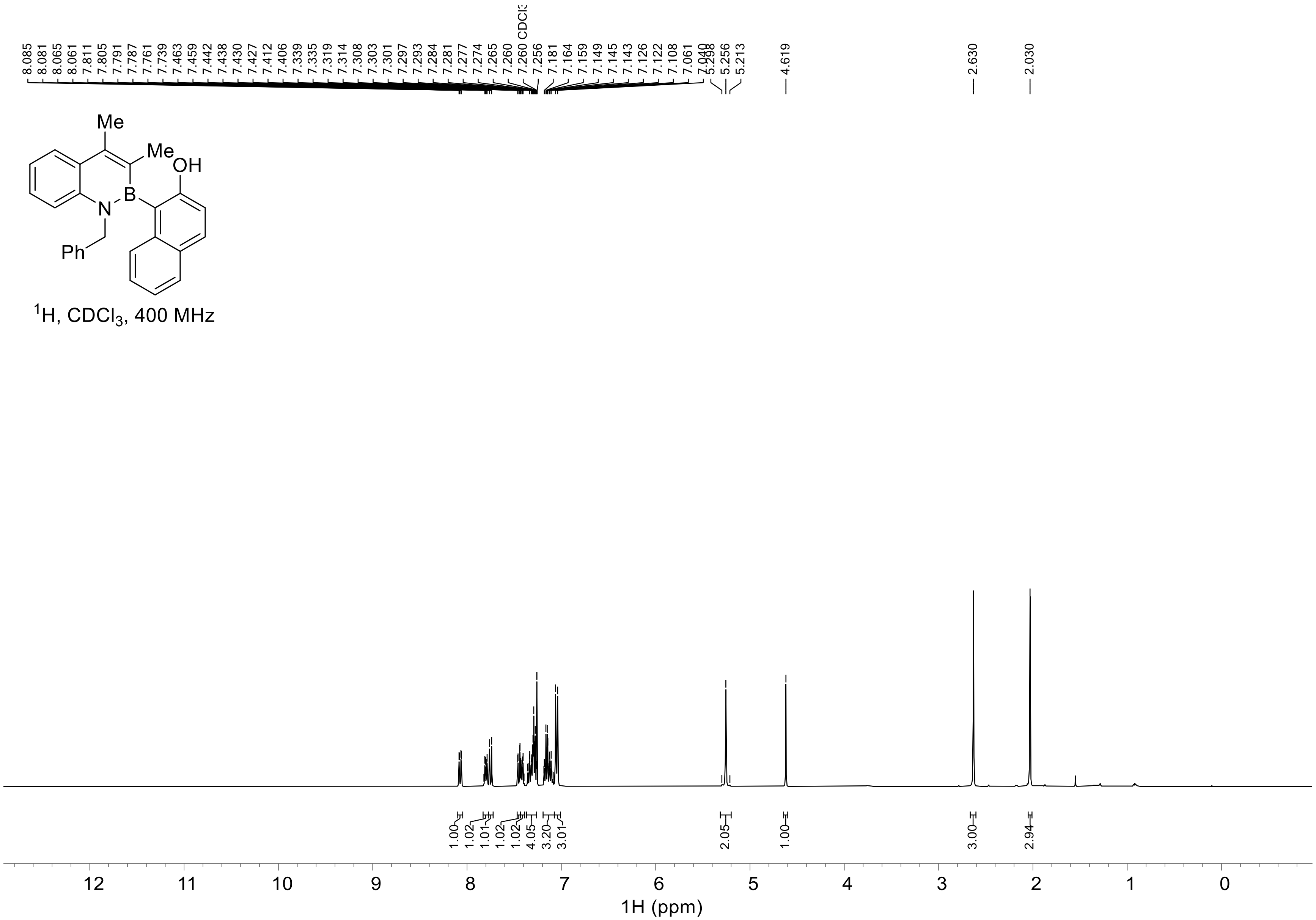

^1H, $CDCl_3$, 400 MHz

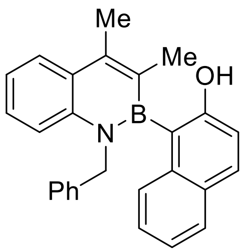

^{13}C {^{1}H}, $CDCl_3$, 101 MHz

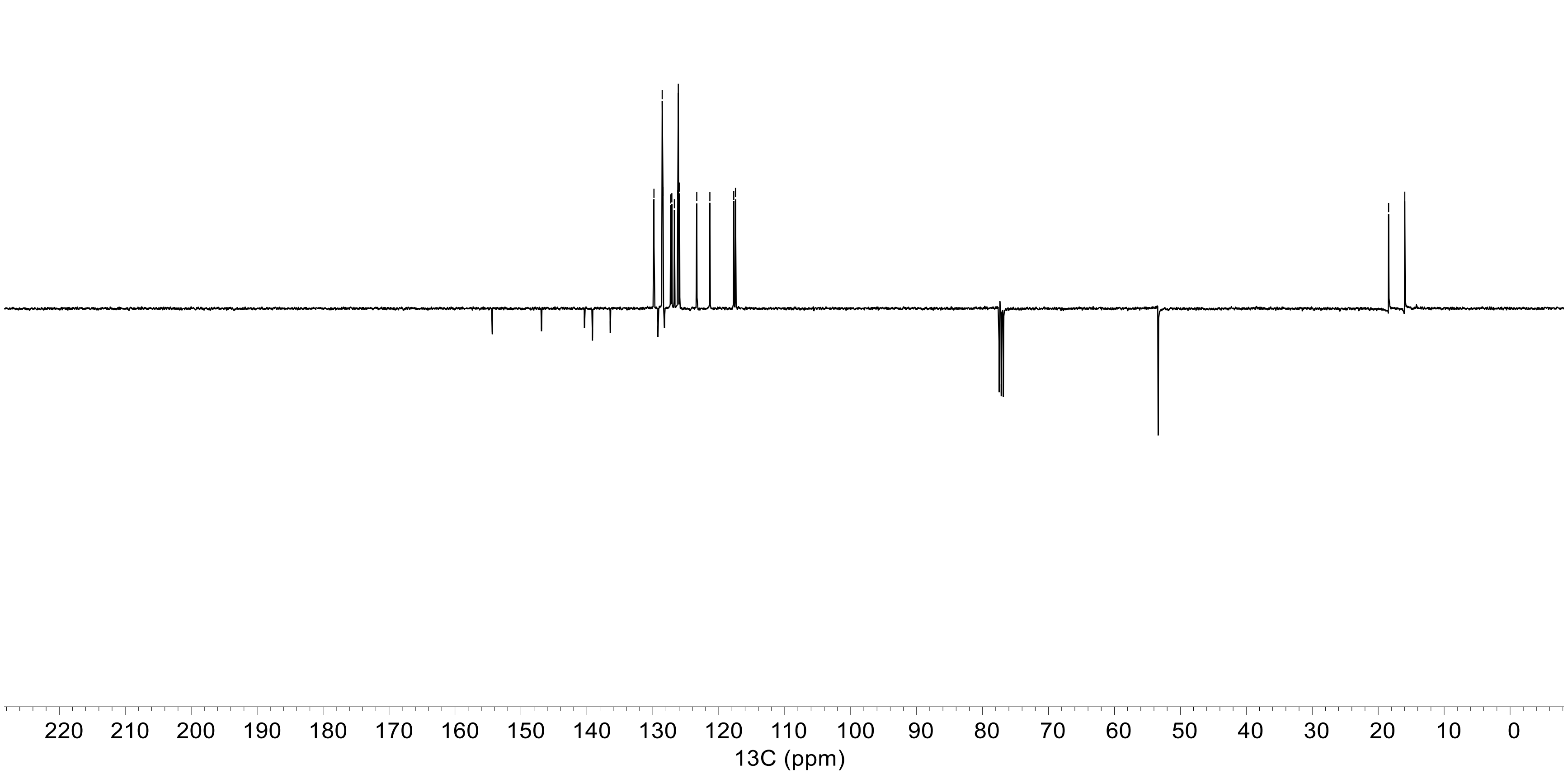

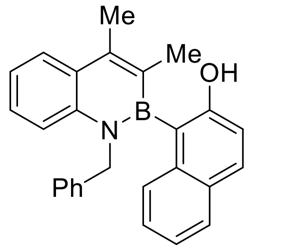

$^{11}B\ \{^1H\}$, $CDCl_3$, 128 MHz

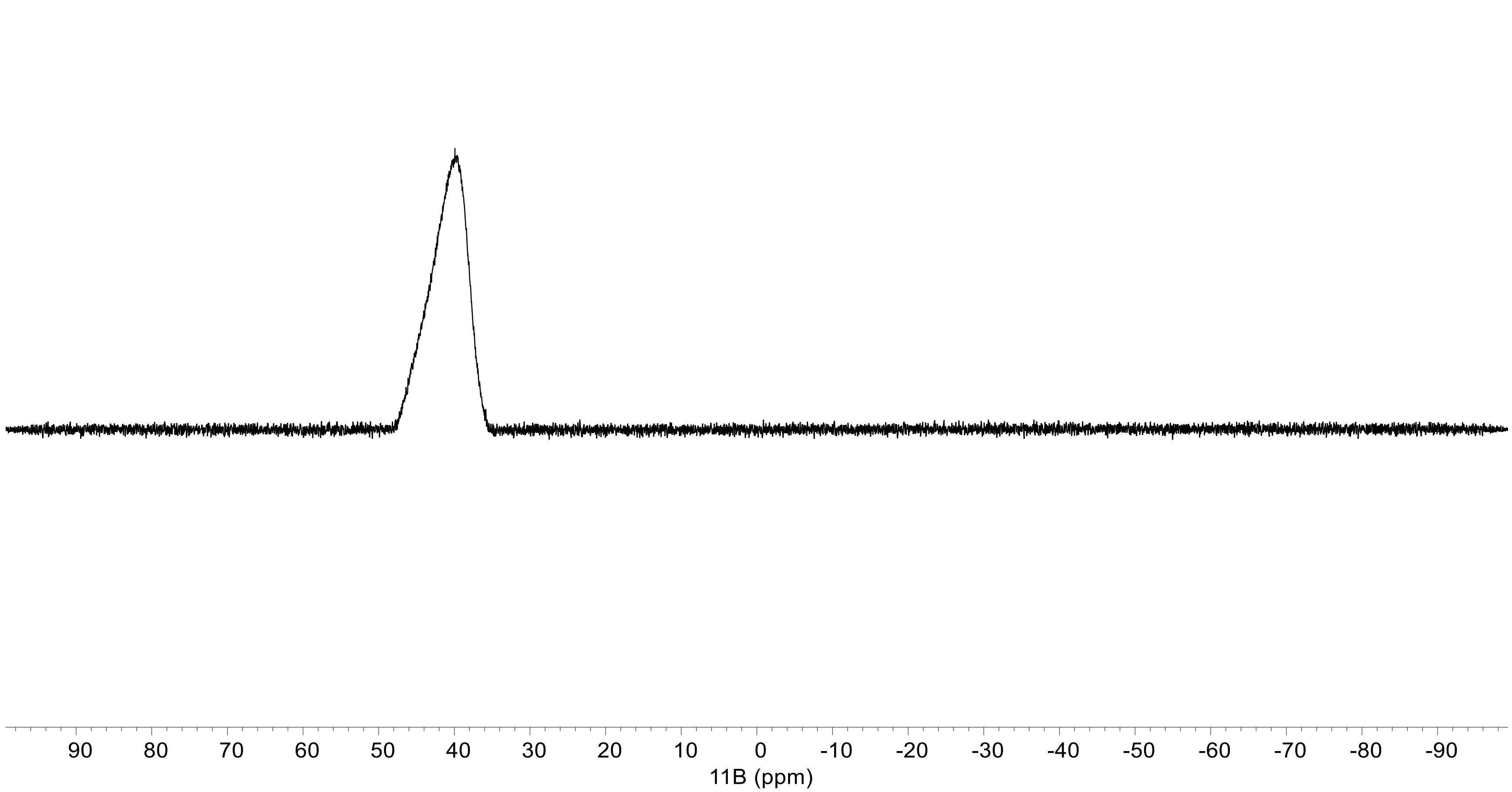

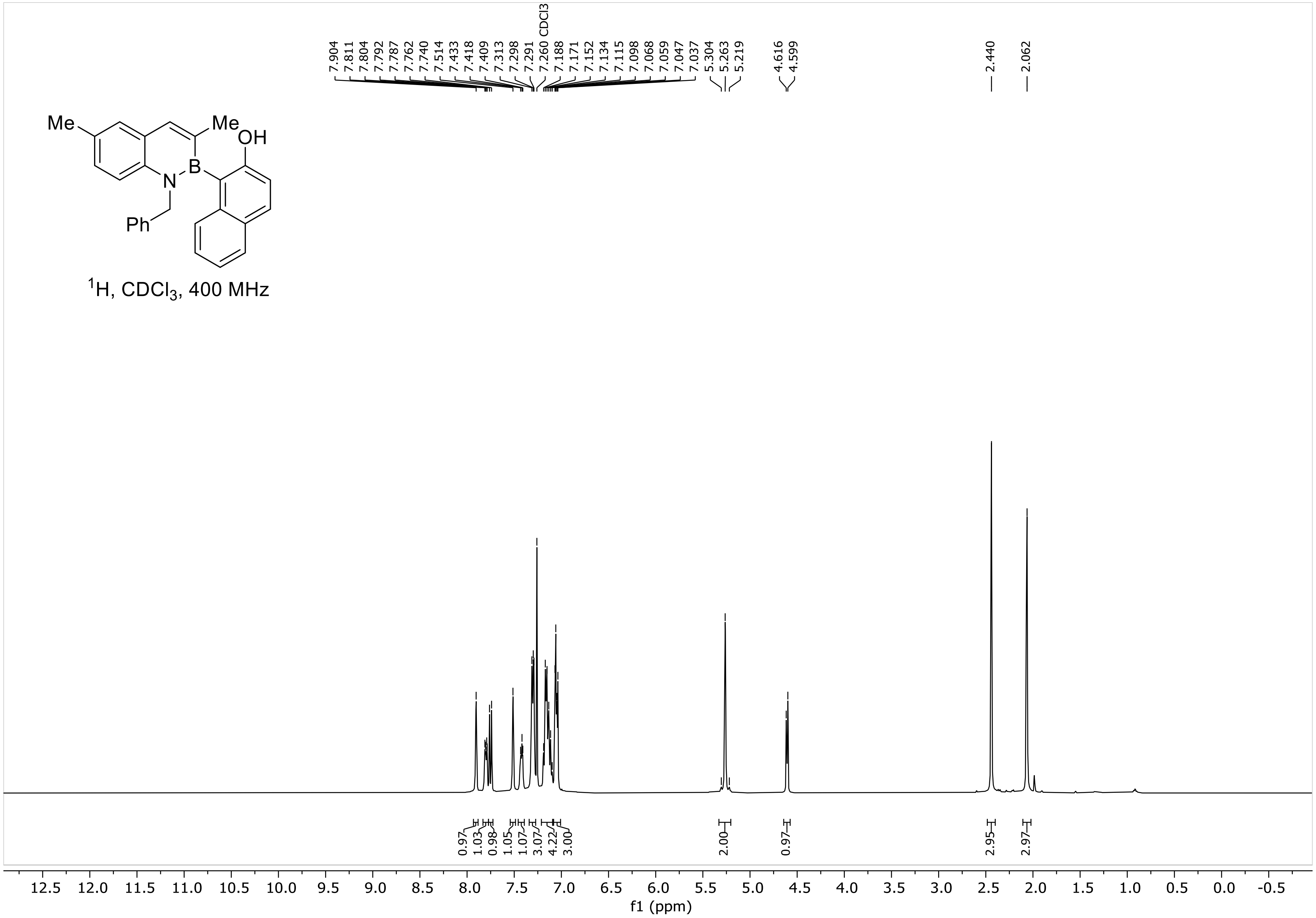

7.904
7.811
7.804
7.792
7.787
7.762
7.740
7.514
7.433
7.418
7.409
7.313
7.298
7.291
7.260 CDCl3
7.188
7.171
7.152
7.134
7.115
7.098
7.068
7.059
7.047
7.037
5.304
5.263
5.219
4.616
4.599
2.440
2.062
Me
Me
OH
N
B
Ph
^{1}H, $CDCl_3$, 400 MHz
0.97
1.03
0.98
1.05
1.07
3.07
4.22
3.00
2.00
0.97
2.95
2.97
12.5 12.0 11.5 11.0 10.5 10.0 9.5 9.0 8.5 8.0 7.5 7.0 6.5 6.0 5.5 5.0 4.5 4.0 3.5 3.0 2.5 2.0 1.5 1.0 0.5 0.0 -0.5
f1 (ppm)

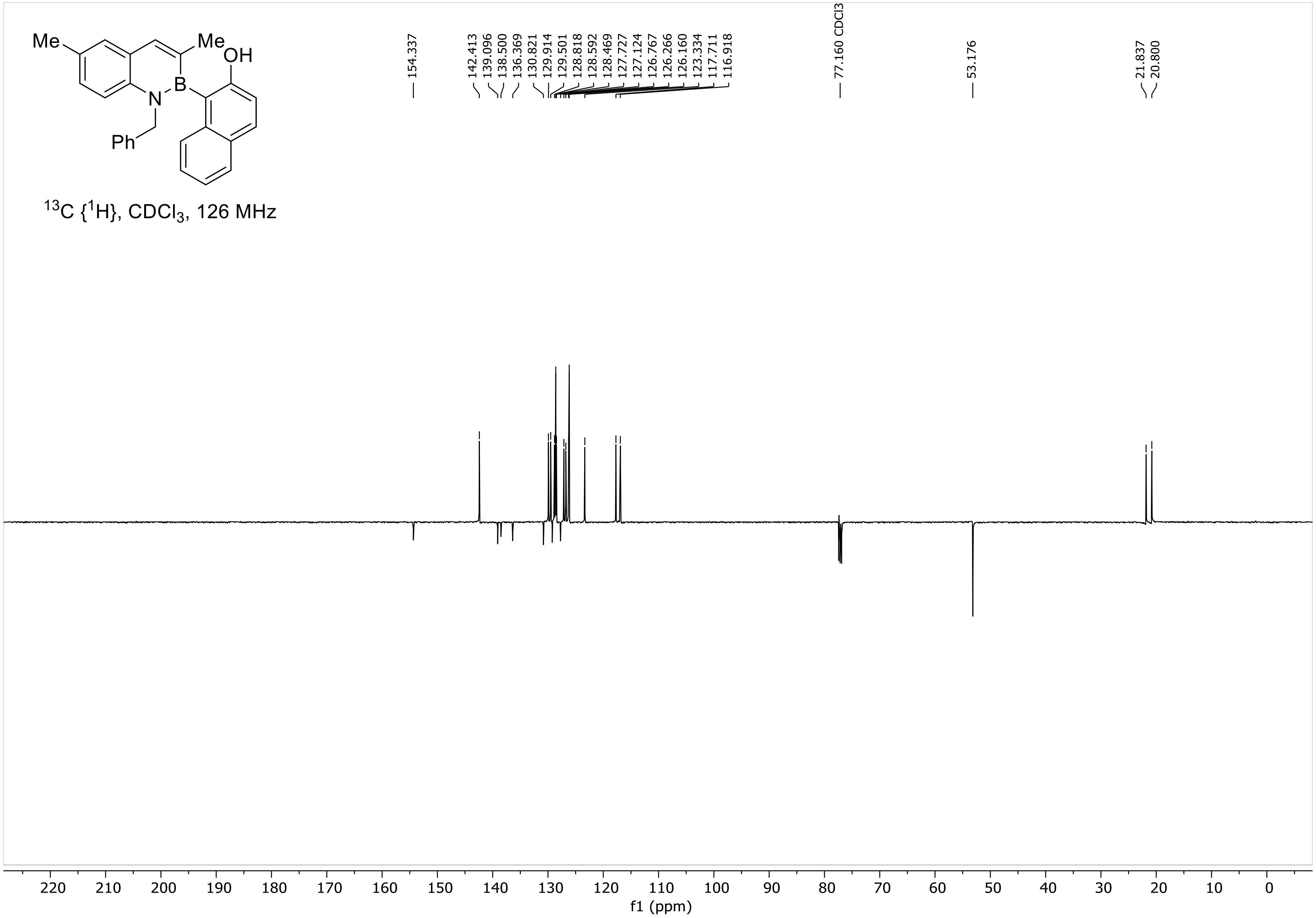

Me
Me
OH
N
B
Ph
^{13}C {^1H}, $CDCl_3$, 126 MHz
154.337
142.413
139.096
138.500
136.369
130.821
129.914
129.501
128.818
128.592
128.469
127.727
127.124
126.767
126.266
126.160
123.334
117.711
116.918
77.160 CDCl3
53.176
21.837
20.800
220 210 200 190 180 170 160 150 140 130 120 110 100 90 80 70 60 50 40 30 20 10 0
f1 (ppm)

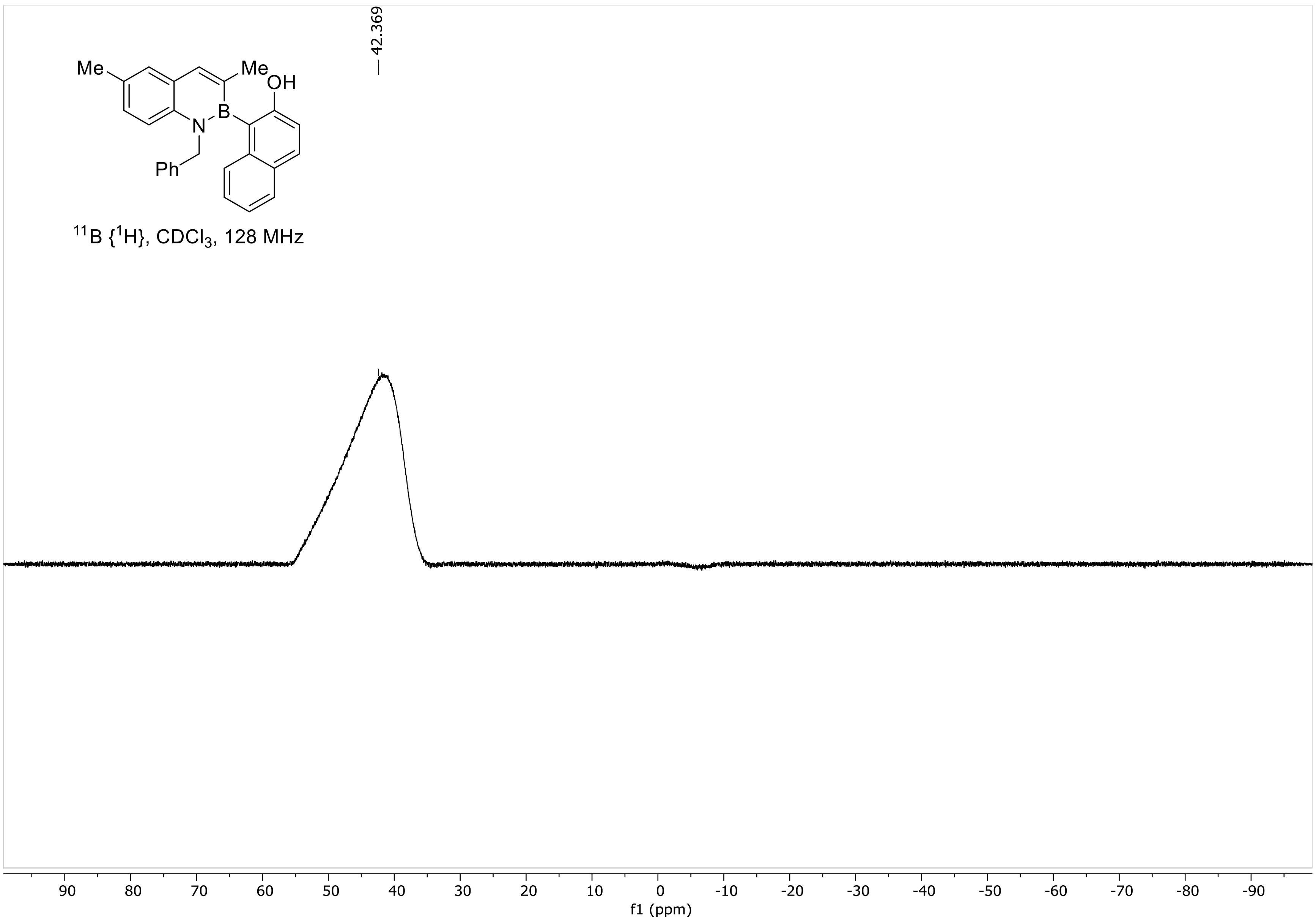

42.369
Me
Me
OH
B
N
Ph
^{11}B {^{1}H}, CDCl$_3$, 128 MHz
90
80
70
60
50
40
30
20
10
0
-10
-20
-30
-40
-50
-60
-70
-80
-90
f1 (ppm)

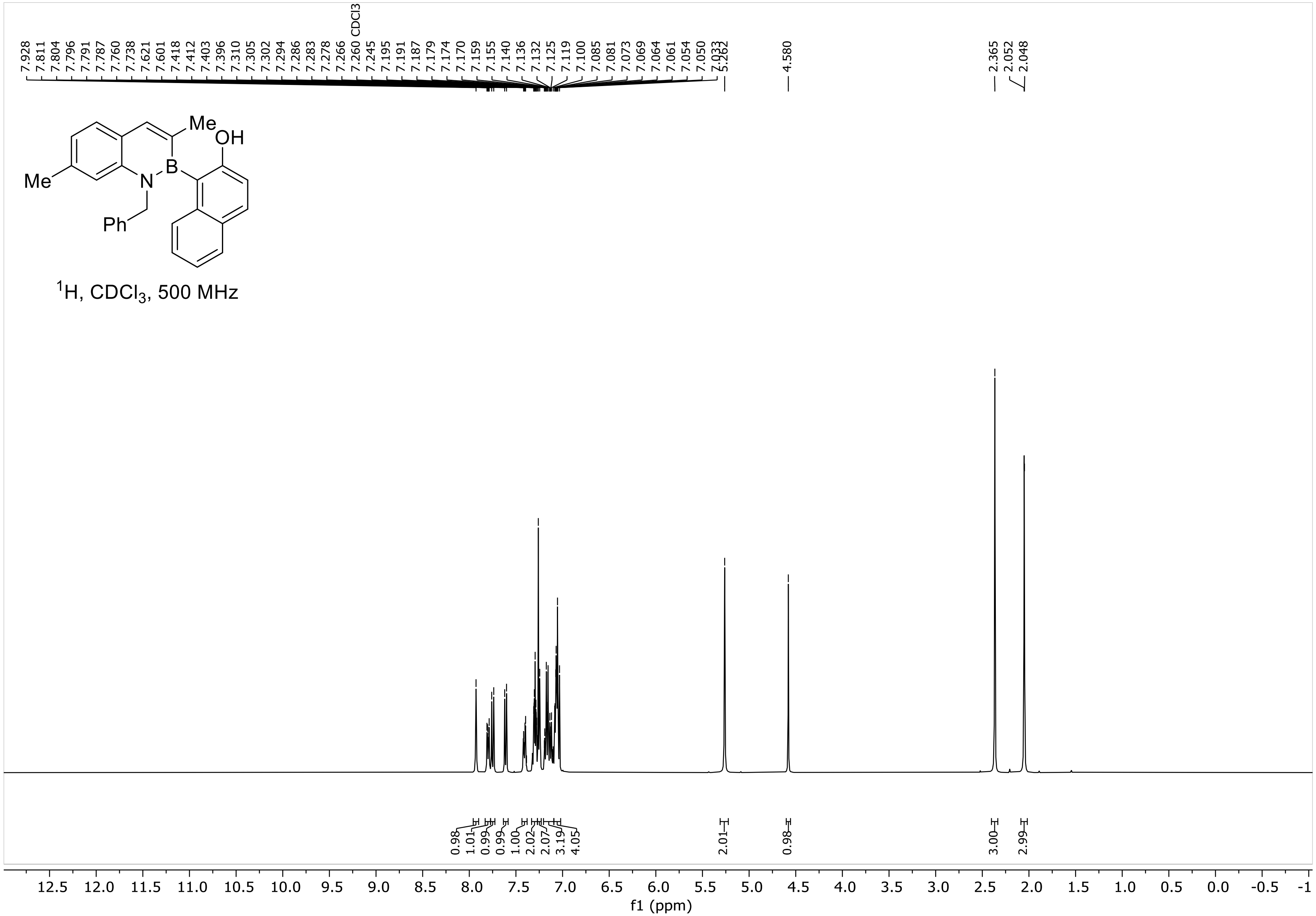

Me
OH
Me
N
B
Ph
^{1}H, CDCl$_3$, 500 MHz
7.928 7.811 7.804 7.796 7.791 7.787 7.760 7.738 7.621 7.601 7.418 7.412 7.403 7.396 7.310 7.305 7.302 7.294 7.286 7.283 7.278 7.266 7.260 CDCl3 7.245 7.195 7.191 7.187 7.179 7.174 7.170 7.159 7.155 7.140 7.136 7.132 7.125 7.119 7.100 7.085 7.081 7.073 7.069 7.064 7.061 7.054 7.050 7.033 5.262
4.580
2.365 2.052 2.048
0.98 1.01 0.99 0.99 1.00 2.02 2.07 3.19 4.05
2.01
0.98
3.00
2.99
12.5 12.0 11.5 11.0 10.5 10.0 9.5 9.0 8.5 8.0 7.5 7.0 6.5 6.0 5.5 5.0 4.5 4.0 3.5 3.0 2.5 2.0 1.5 1.0 0.5 0.0 -0.5 -1
f1 (ppm)

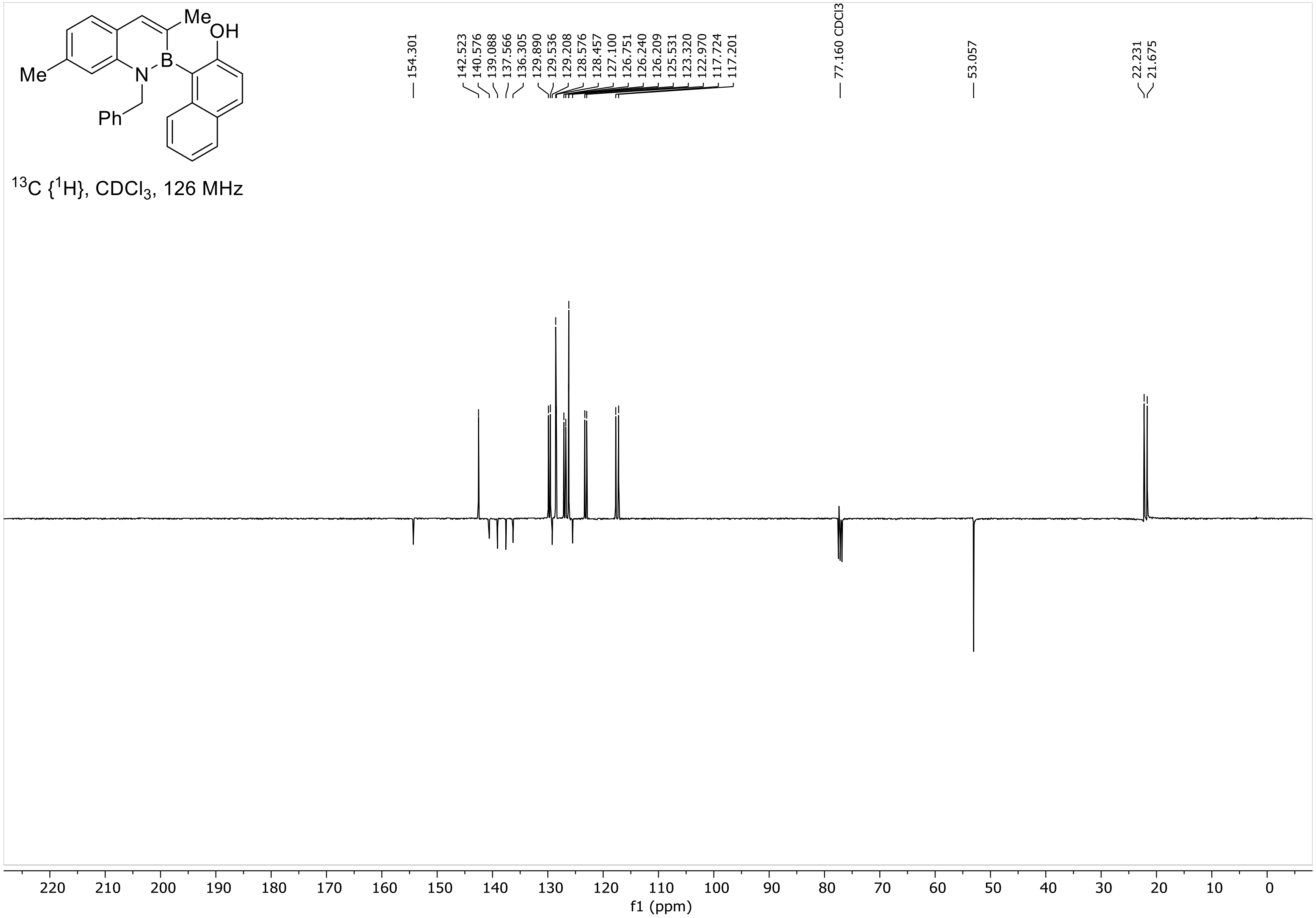
Me
OH
Me
N
B
Ph
$^{13}C\ \{^1H\}$, $CDCl_3$, 126 MHz
154.301
142.523
140.576
139.088
137.566
136.305
129.890
129.536
129.208
128.576
128.457
127.100
126.751
126.240
126.209
125.531
123.320
122.970
117.724
117.201
77.160 CDCl3
53.057
22.231
21.675
f1 (ppm)

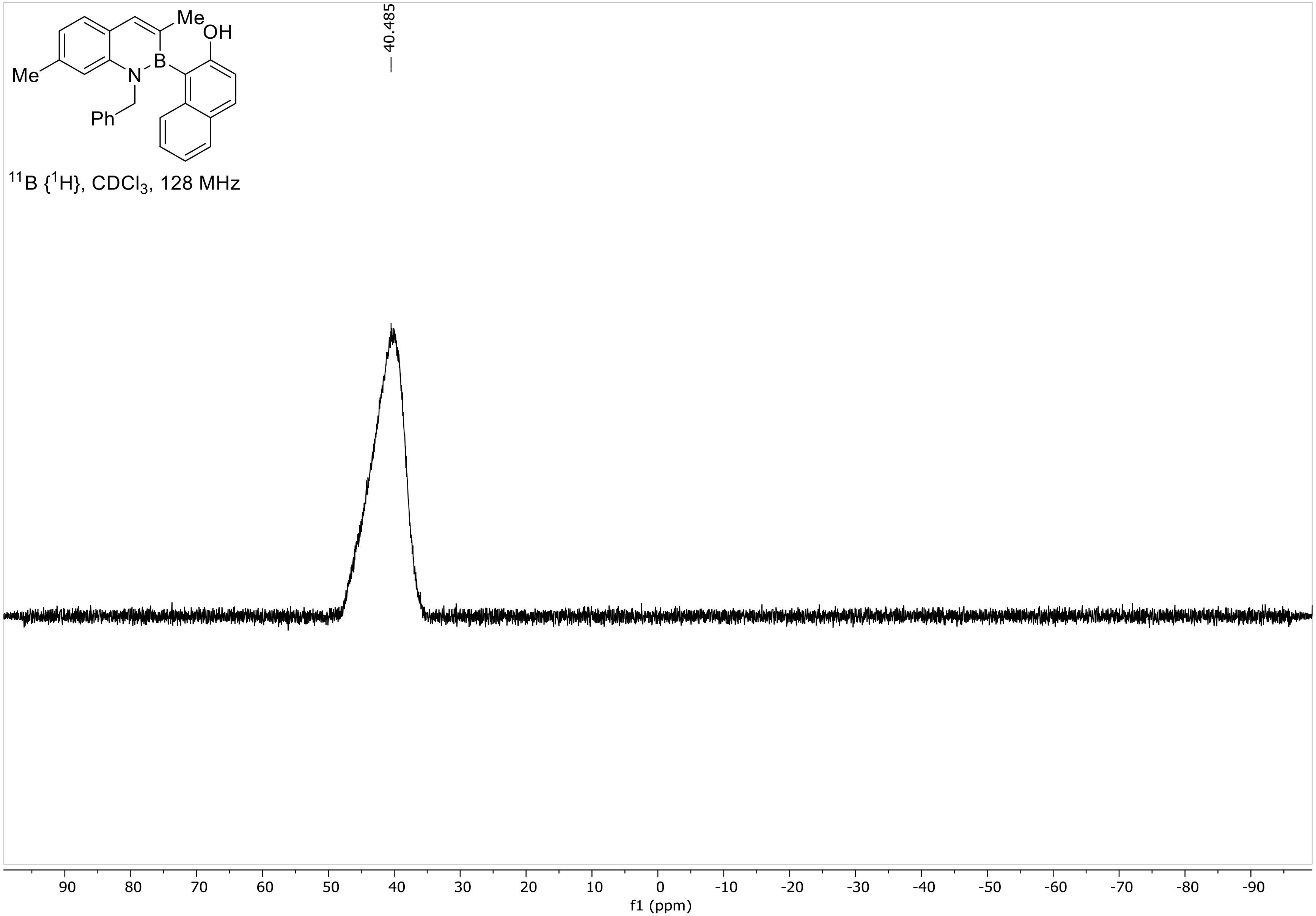
Me
OH
Me
N
B
Ph
^{11}B {^{1}H}, CDCl$_3$, 128 MHz
40.485
90 80 70 60 50 40 30 20 10 0 -10 -20 -30 -40 -50 -60 -70 -80 -90
f1 (ppm)

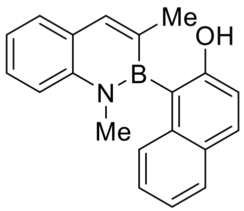

^{1}H, $CDCl_3$, 400 MHz

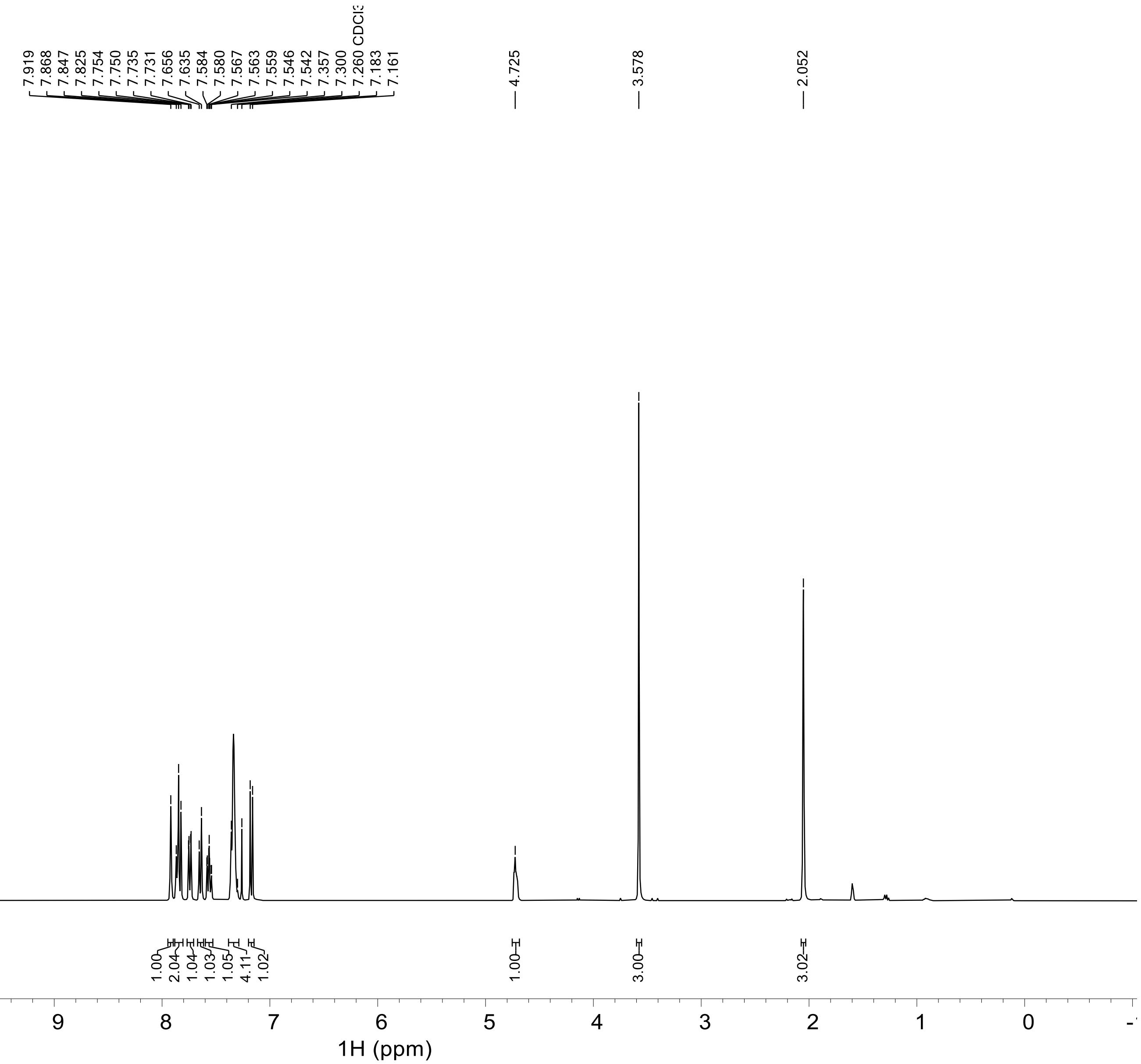

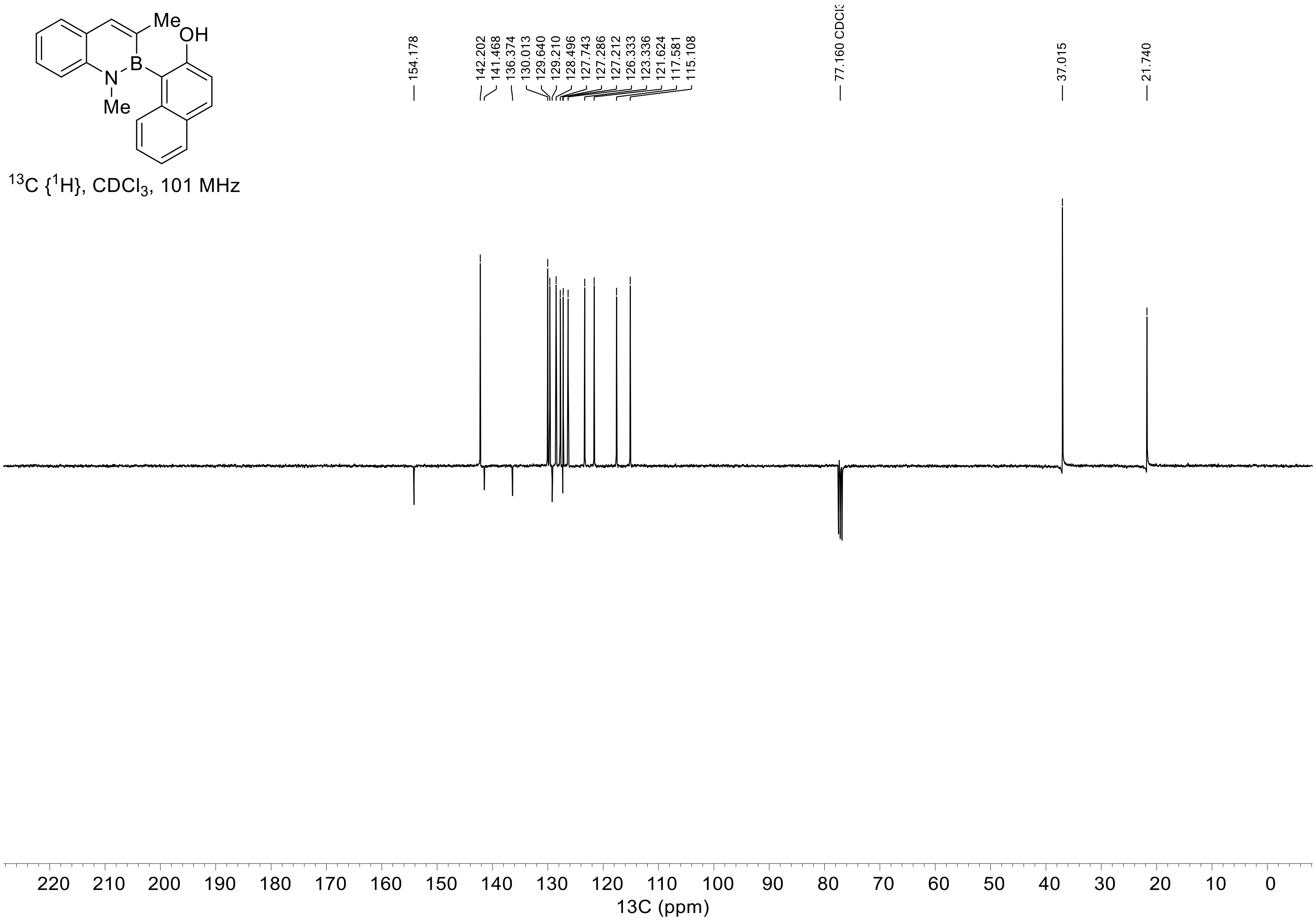

Me
OH
B
N
Me
^{13}C {^{1}H}, CDCl$_3$, 101 MHz
154.178
142.202
141.468
136.374
130.013
129.640
129.210
128.496
127.743
127.286
127.212
126.333
123.336
121.624
117.581
115.108
77.160 CDCl3
37.015
21.740
220 210 200 190 180 170 160 150 140 130 120 110 100 90 80 70 60 50 40 30 20 10 0
13C (ppm)

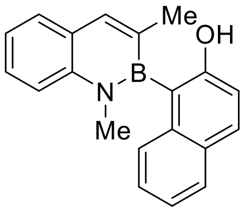

^{11}B {^{1}H}, $CDCl_3$, 128 MHz

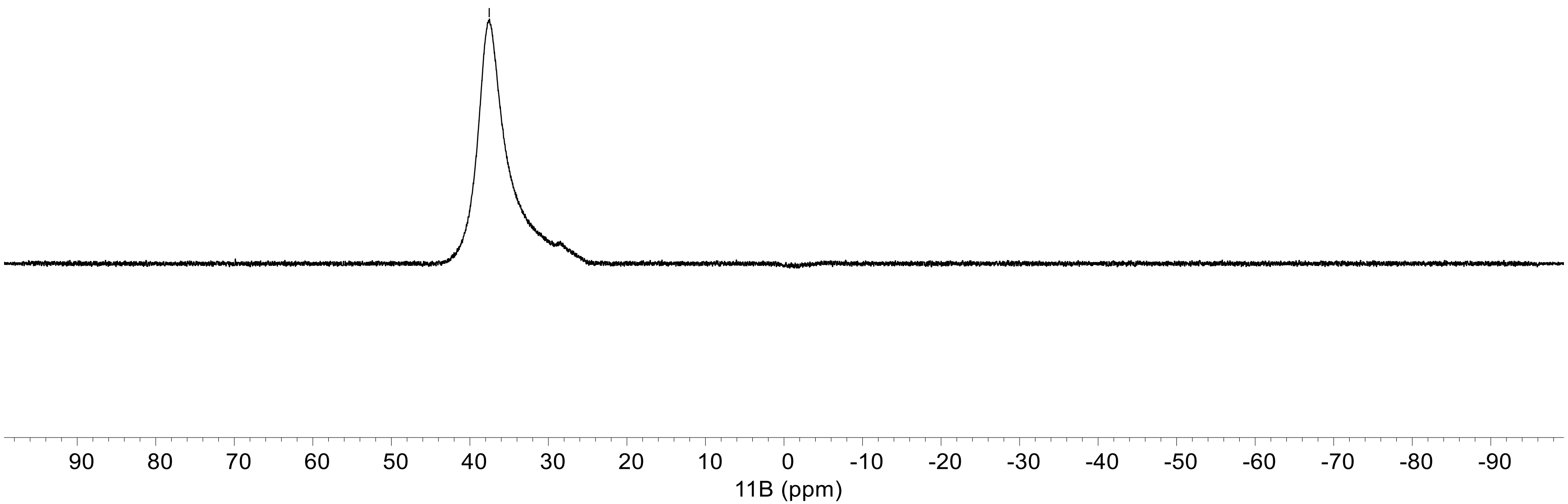

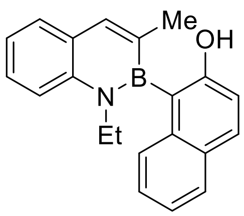

^1H, $CDCl_3$, 400 MHz

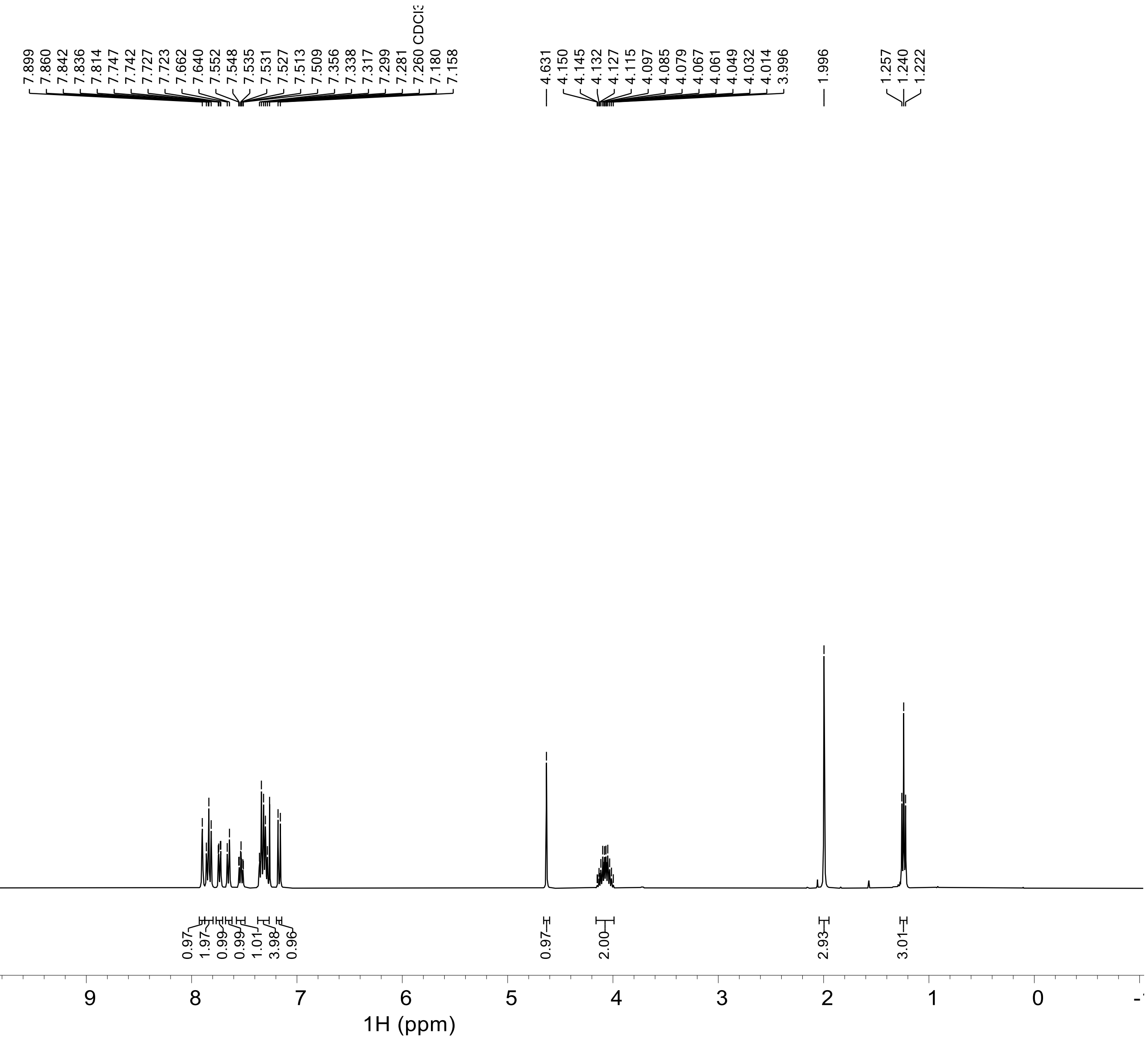

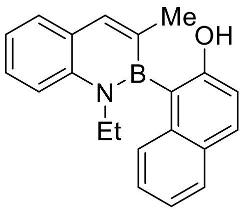

^{13}C {^{1}H}, $CDCl_3$, 101 MHz

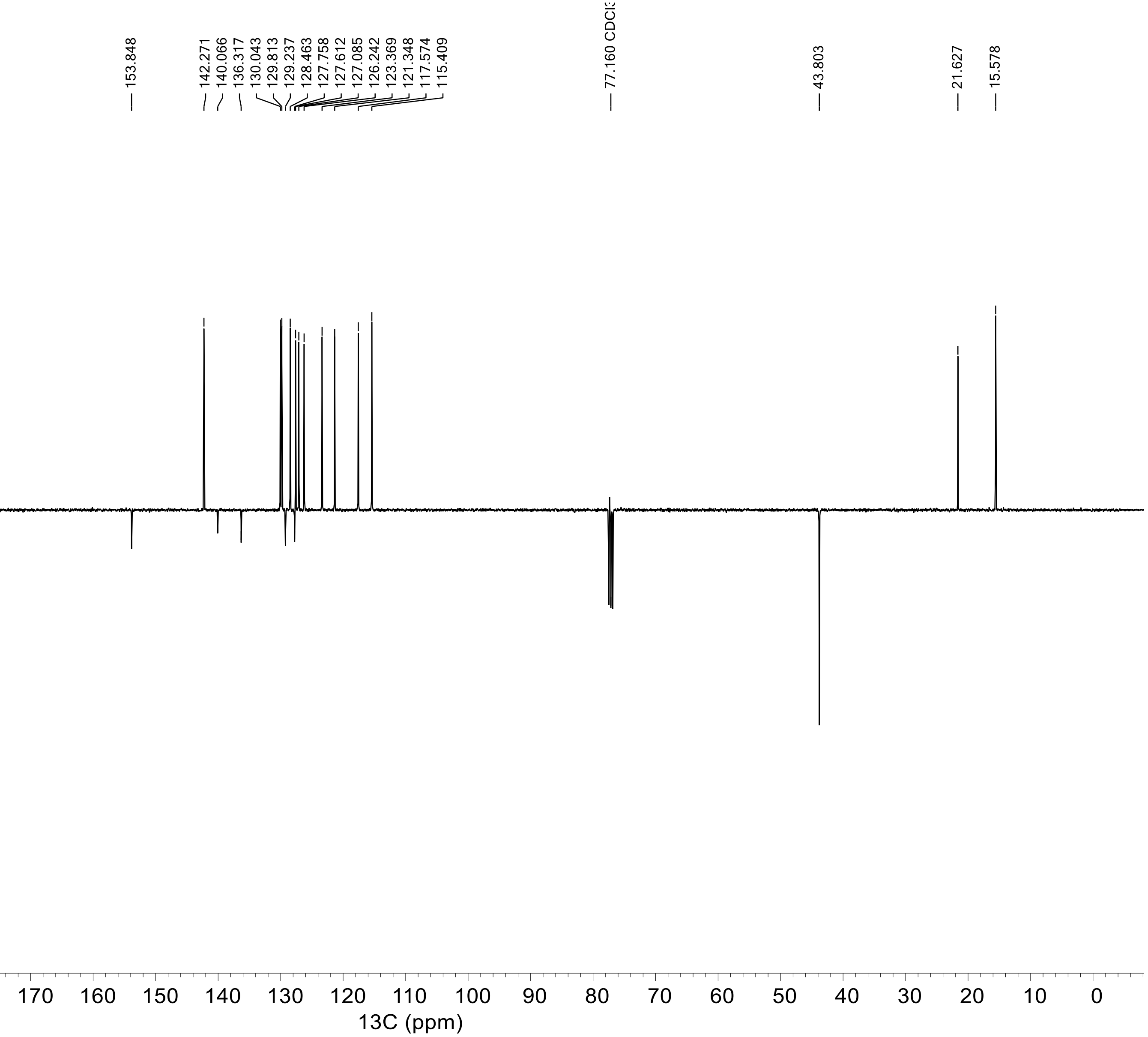

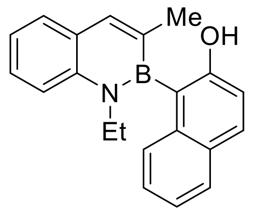

^{11}B {^{1}H}, $CDCl_3$, 128 MHz

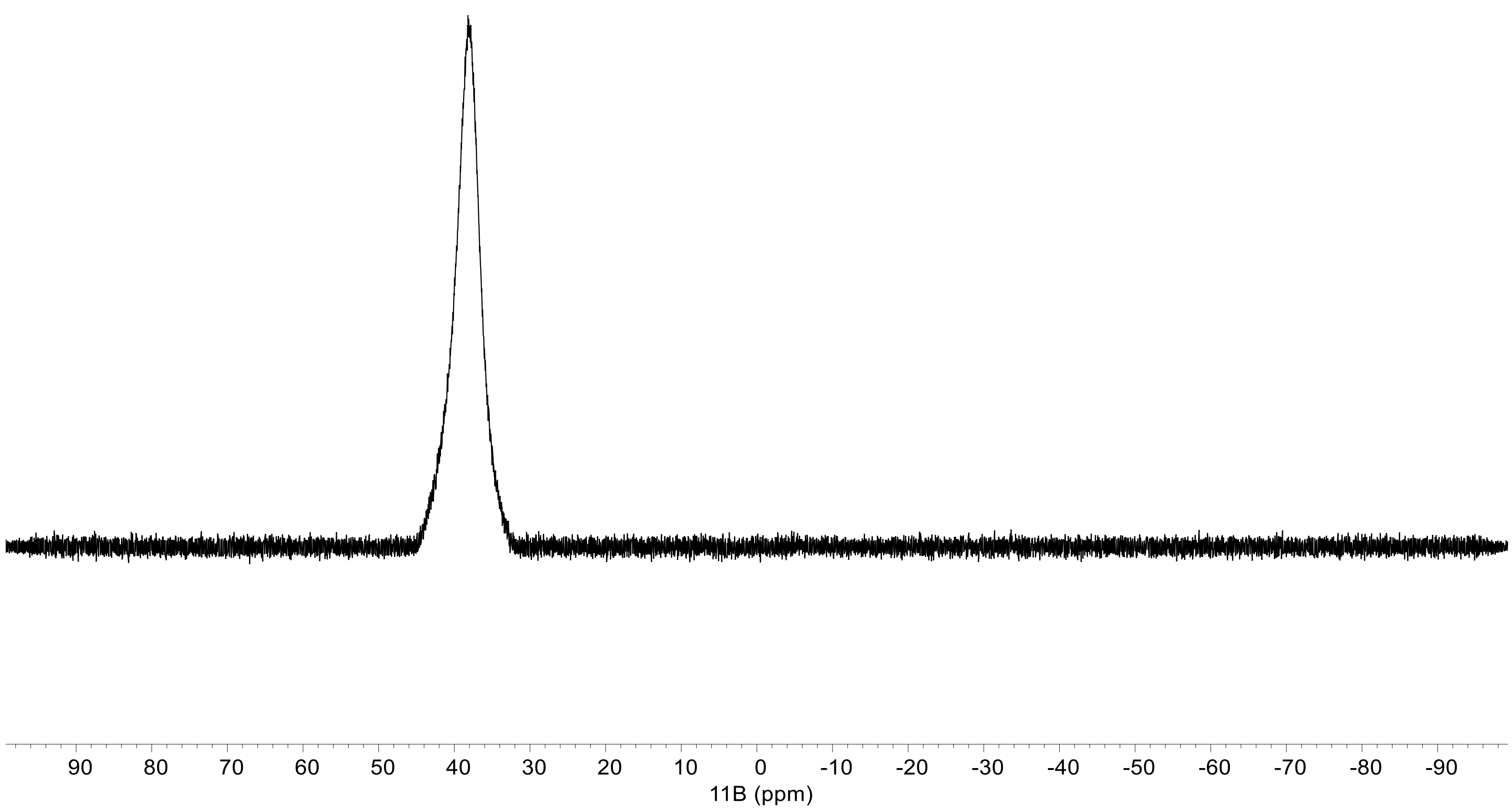

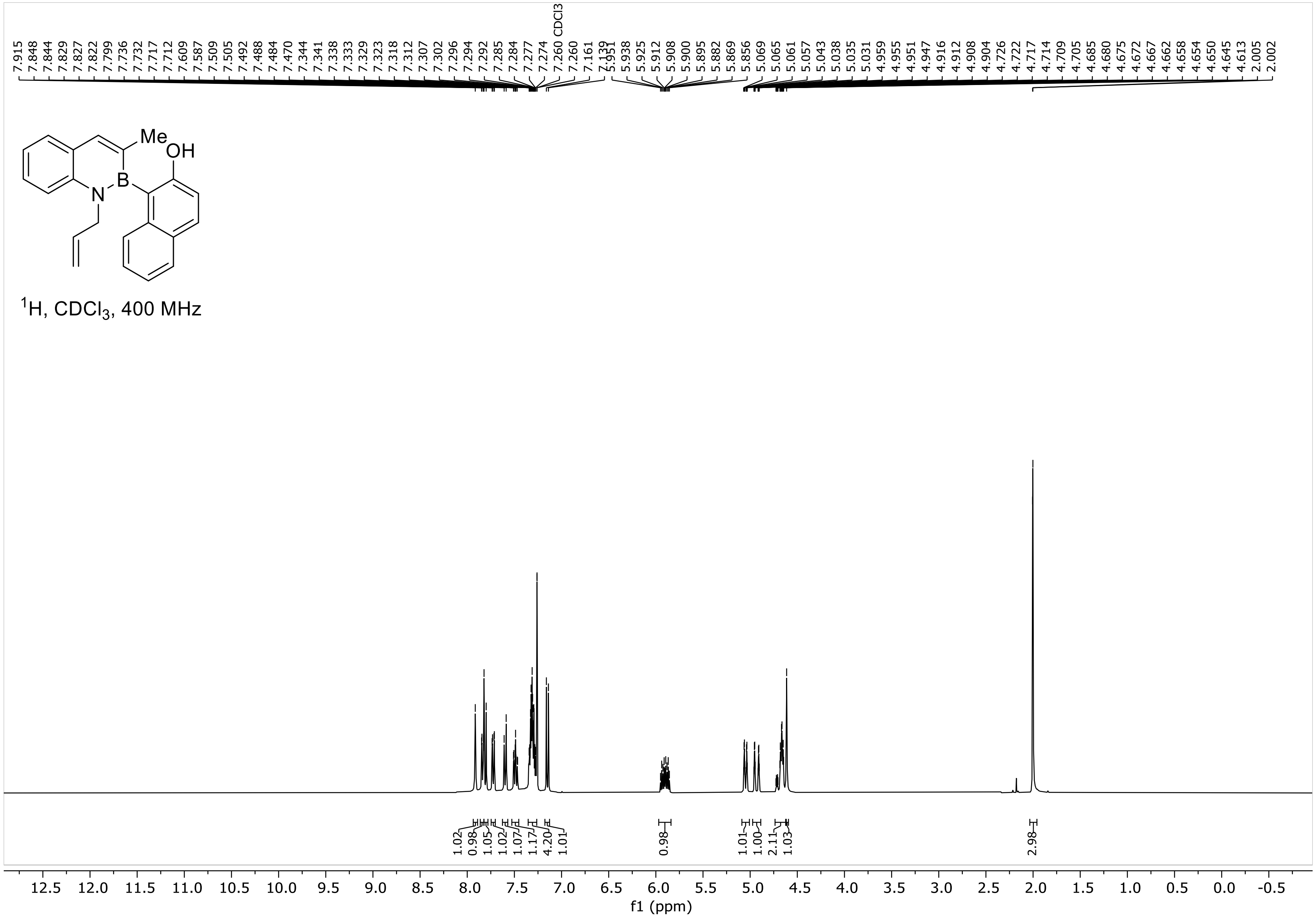
7.915 7.848 7.844 7.829 7.827 7.822 7.799 7.736 7.732 7.717 7.712 7.609 7.587 7.509 7.505 7.492 7.488 7.484 7.470 7.344 7.341 7.338 7.333 7.329 7.323 7.318 7.312 7.307 7.302 7.296 7.294 7.292 7.285 7.284 7.277 7.274 7.260 CDCl3 7.260 7.161 7.139 5.951 5.938 5.925 5.912 5.908 5.900 5.895 5.882 5.869 5.856 5.069 5.065 5.061 5.057 5.043 5.038 5.035 5.031 4.959 4.955 4.951 4.947 4.916 4.912 4.908 4.904 4.726 4.722 4.717 4.714 4.709 4.705 4.685 4.680 4.675 4.672 4.667 4.662 4.658 4.654 4.650 4.645 4.613 2.005 2.002
Me
OH
B
N
^{1}H, CDCl$_3$, 400 MHz
1.02 0.98 1.05 1.02 1.07 1.17 4.20 1.01 0.98 1.01 1.00 2.11 1.03 2.98
12.5 12.0 11.5 11.0 10.5 10.0 9.5 9.0 8.5 8.0 7.5 7.0 6.5 6.0 5.5 5.0 4.5 4.0 3.5 3.0 2.5 2.0 1.5 1.0 0.5 0.0 -0.5
f1 (ppm)

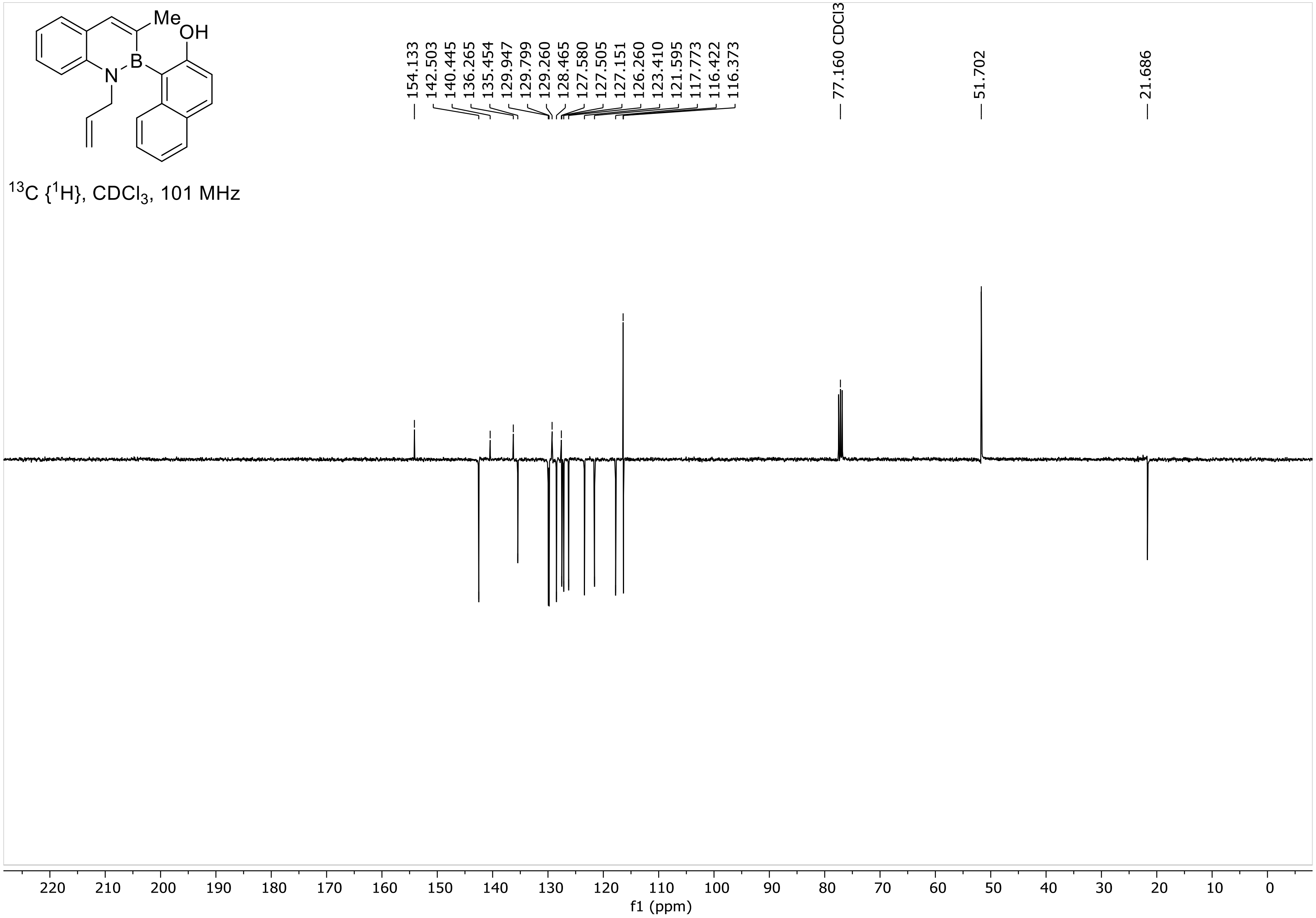
Me
OH
B
N
$^{13}C\ \{^1H\}$, $CDCl_3$, 101 MHz
154.133
142.503
140.445
136.265
135.454
129.947
129.799
129.260
128.465
127.580
127.505
127.151
126.260
123.410
121.595
117.773
116.422
116.373
77.160 CDCl3
51.702
21.686
220 210 200 190 180 170 160 150 140 130 120 110 100 90 80 70 60 50 40 30 20 10 0
f1 (ppm)

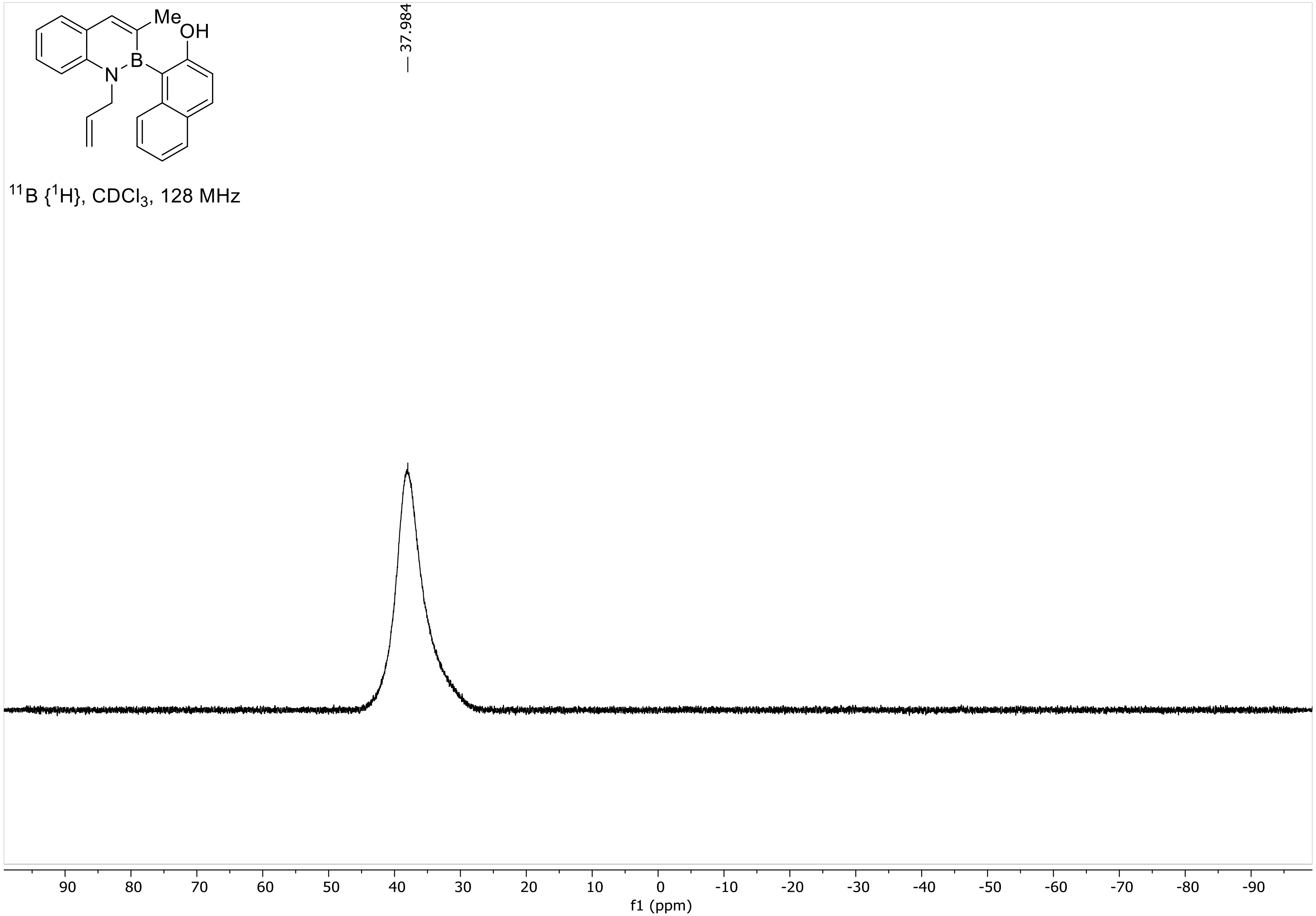
Me
OH
N
B
37.984
^{11}B {^{1}H}, $CDCl_3$, 128 MHz
90 80 70 60 50 40 30 20 10 0 -10 -20 -30 -40 -50 -60 -70 -80 -90
f1 (ppm)

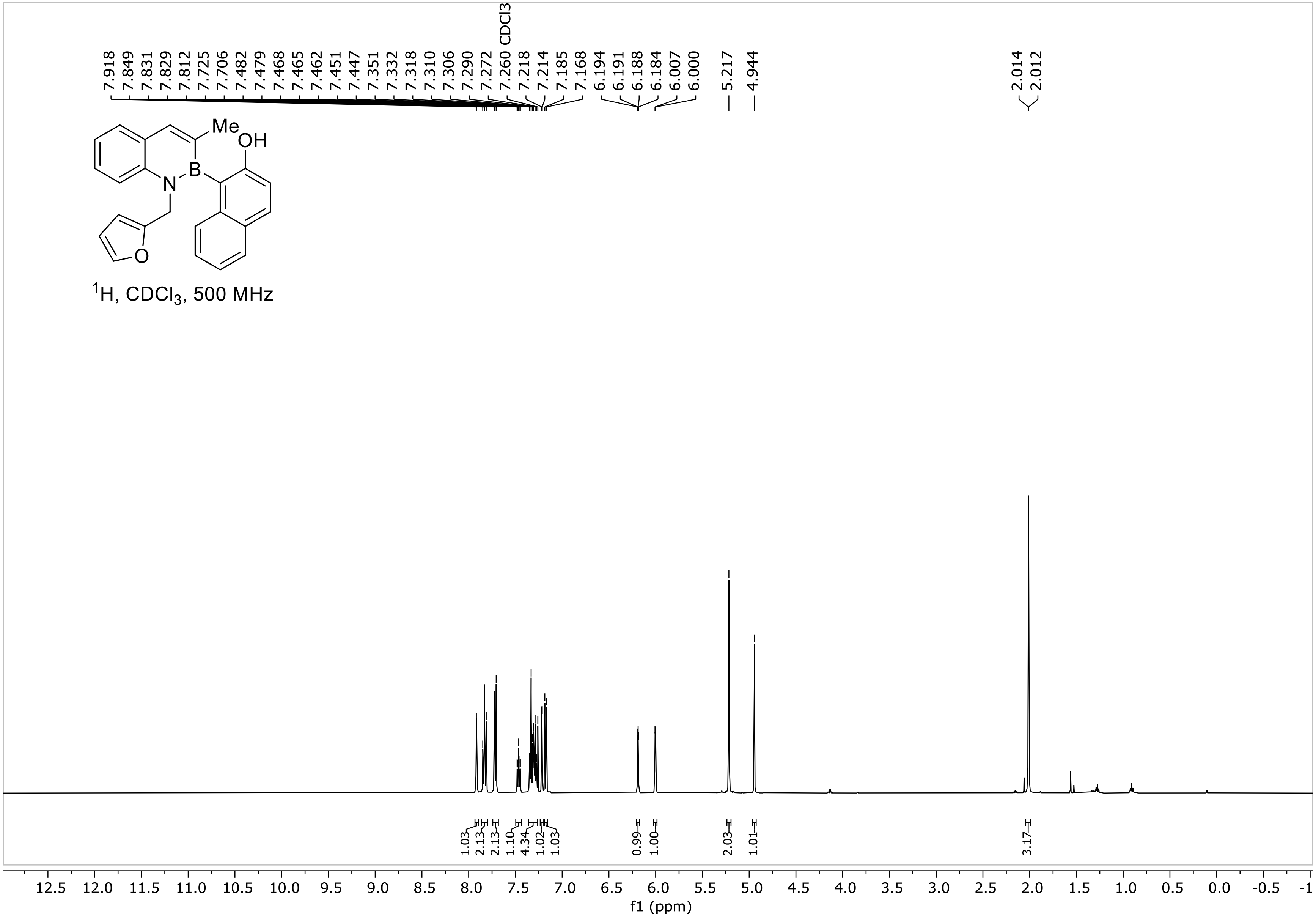

7.918
7.849
7.831
7.829
7.812
7.725
7.706
7.482
7.479
7.468
7.465
7.462
7.451
7.447
7.351
7.332
7.318
7.310
7.306
7.290
7.272
7.260 CDCl3
7.218
7.214
7.185
7.168
6.194
6.191
6.188
6.184
6.007
6.000
5.217
4.944
2.014
2.012
Me
OH
N
B
O
^{1}H, CDCl$_3$, 500 MHz
1.03
2.13
2.13
1.10
4.34
1.02
1.03
0.99
1.00
2.03
1.01
3.17
12.5 12.0 11.5 11.0 10.5 10.0 9.5 9.0 8.5 8.0 7.5 7.0 6.5 6.0 5.5 5.0 4.5 4.0 3.5 3.0 2.5 2.0 1.5 1.0 0.5 0.0 -0.5 -1
f1 (ppm)

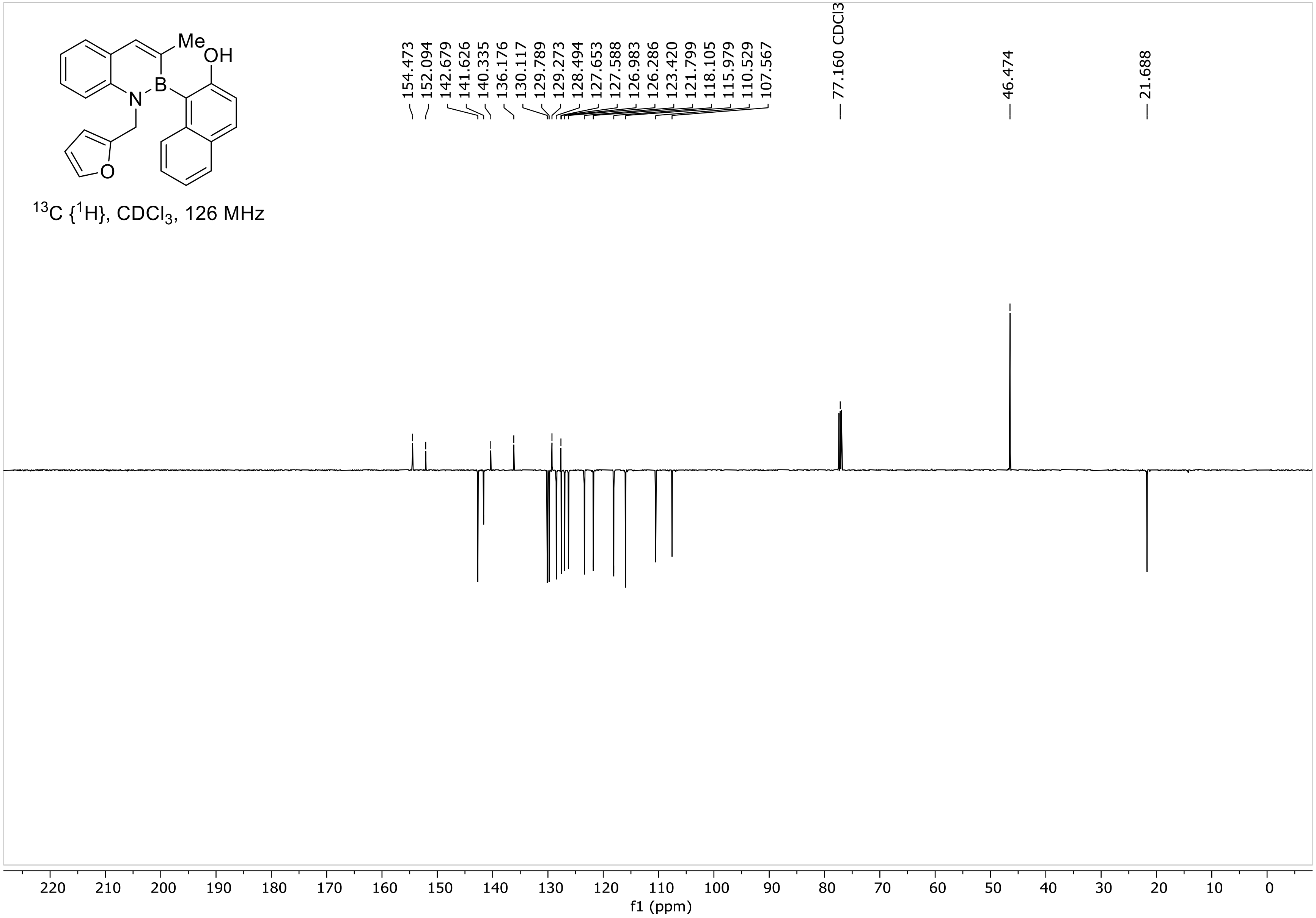

Me
OH
N
B
O
^{13}C {^{1}H}, CDCl$_3$, 126 MHz
154.473
152.094
142.679
141.626
140.335
136.176
130.117
129.789
129.273
128.494
127.653
127.588
126.983
126.286
123.420
121.799
118.105
115.979
110.529
107.567
77.160 CDCl3
46.474
21.688
220 210 200 190 180 170 160 150 140 130 120 110 100 90 80 70 60 50 40 30 20 10 0
f1 (ppm)

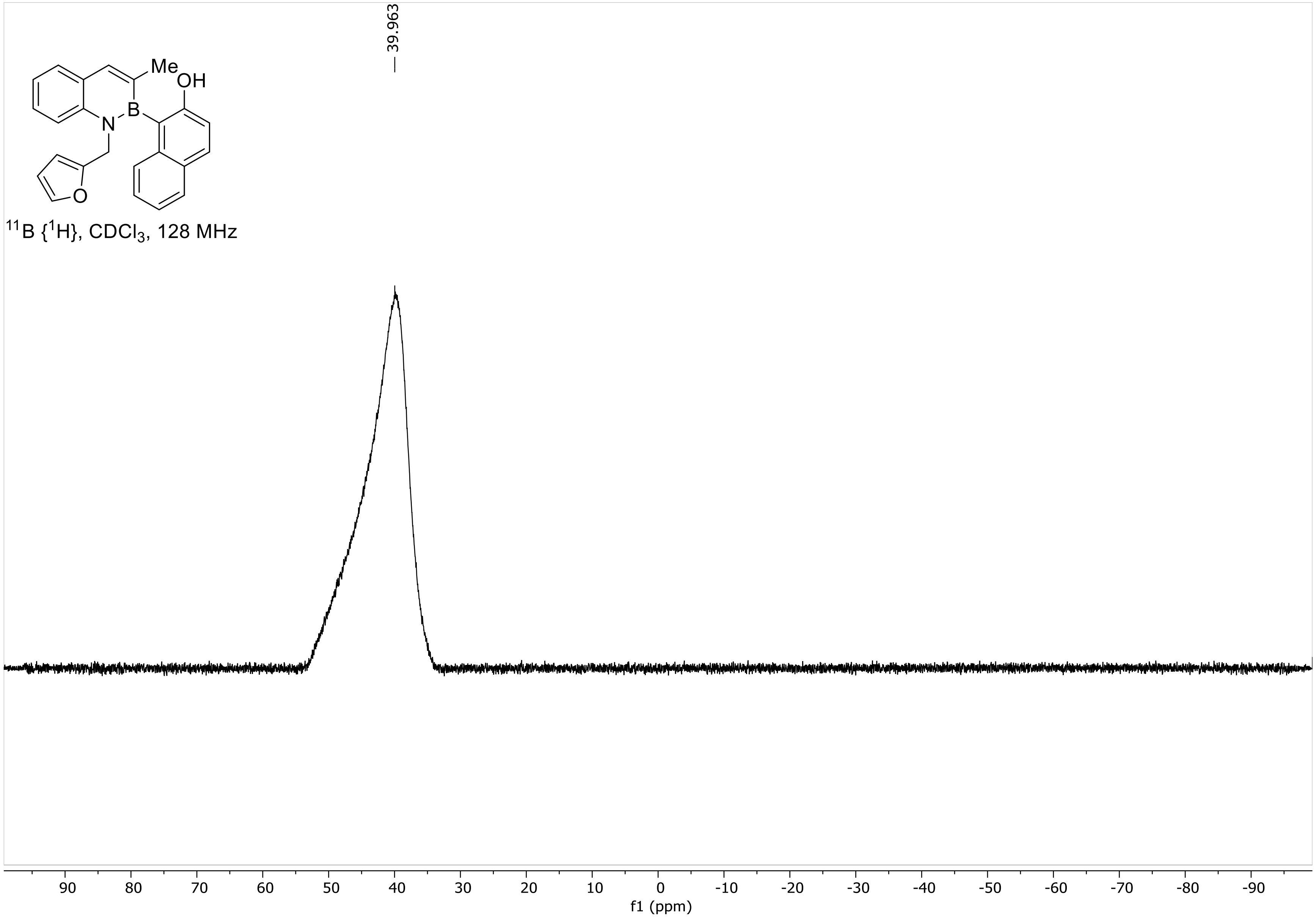
39.963
Me
OH
N
B
O
^{11}B {^{1}H}, $CDCl_3$, 128 MHz
90 80 70 60 50 40 30 20 10 0 -10 -20 -30 -40 -50 -60 -70 -80 -90
f1 (ppm)

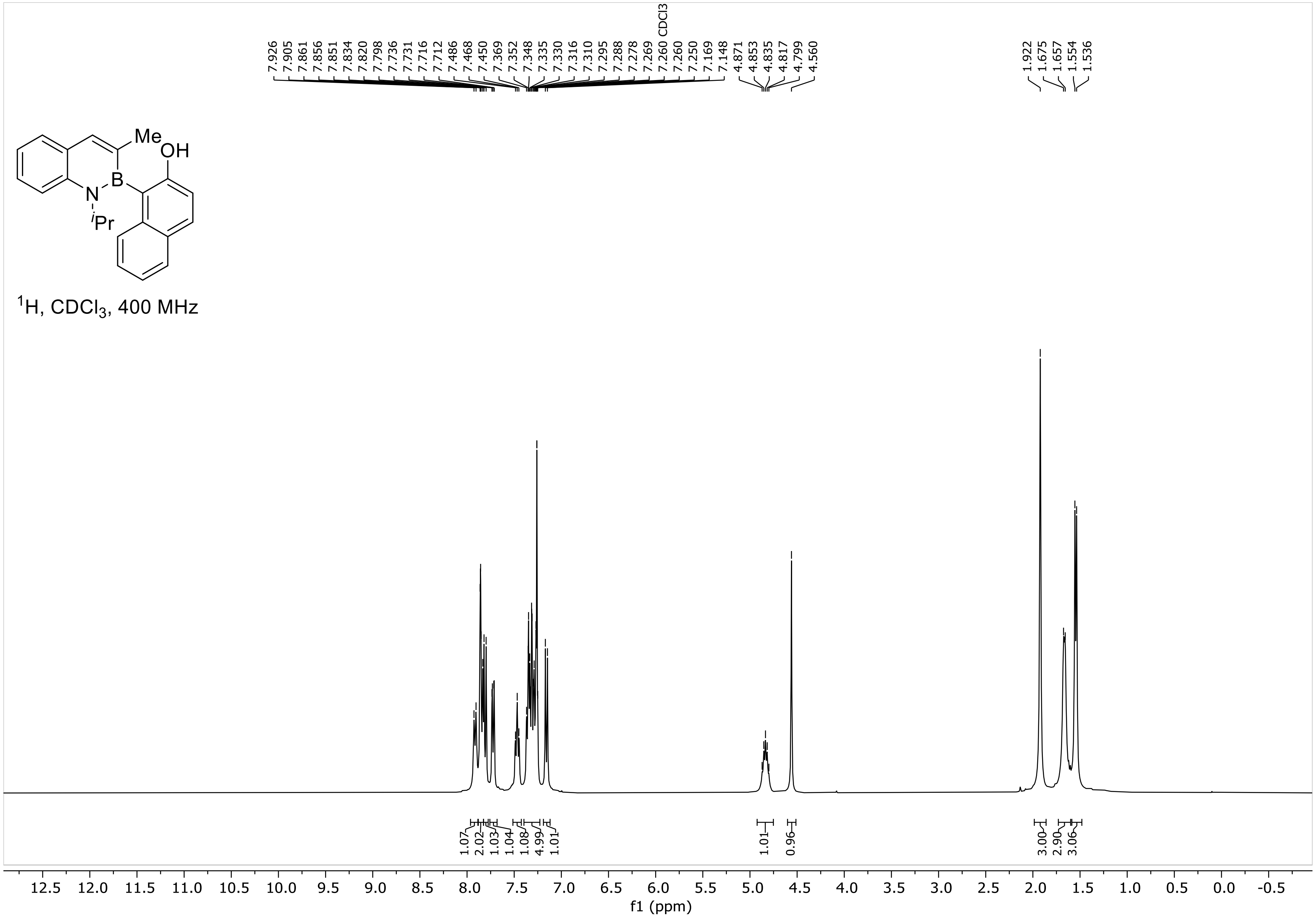

Me
OH
B
N
iPr
1H, CDCl3, 400 MHz
7.926
7.905
7.861
7.856
7.851
7.834
7.820
7.798
7.736
7.731
7.716
7.712
7.486
7.468
7.450
7.369
7.352
7.348
7.335
7.330
7.316
7.310
7.295
7.288
7.278
7.269
7.260 CDCl3
7.260
7.250
7.169
7.148
4.871
4.853
4.835
4.817
4.799
4.560
1.922
1.675
1.657
1.554
1.536
1.07
2.02
1.03
1.04
1.08
4.99
1.01
1.01
0.96
3.00
2.90
3.06
12.5 12.0 11.5 11.0 10.5 10.0 9.5 9.0 8.5 8.0 7.5 7.0 6.5 6.0 5.5 5.0 4.5 4.0 3.5 3.0 2.5 2.0 1.5 1.0 0.5 0.0 -0.5
f1 (ppm)

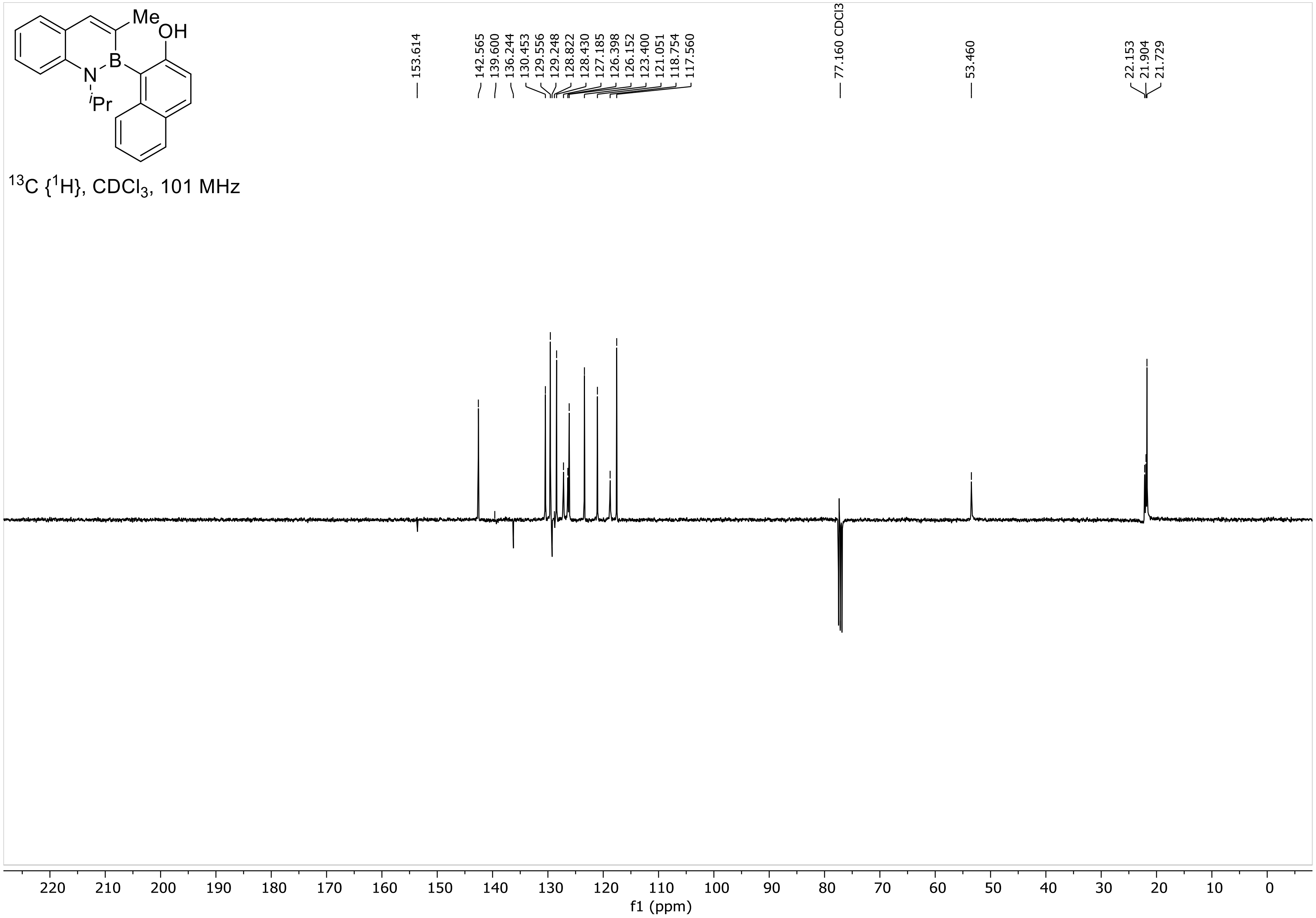
Me
OH
B
N
iPr
^{13}C {^{1}H}, $CDCl_3$, 101 MHz
153.614
142.565
139.600
136.244
130.453
129.556
129.248
128.822
128.430
127.185
126.398
126.152
123.400
121.051
118.754
117.560
77.160 CDCl3
53.460
22.153
21.904
21.729
220 210 200 190 180 170 160 150 140 130 120 110 100 90 80 70 60 50 40 30 20 10 0
f1 (ppm)

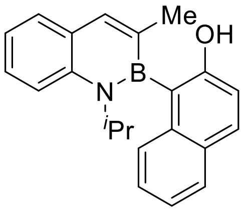

$^{11}B\{^1H\}$, $CDCl_3$, 128 MHz

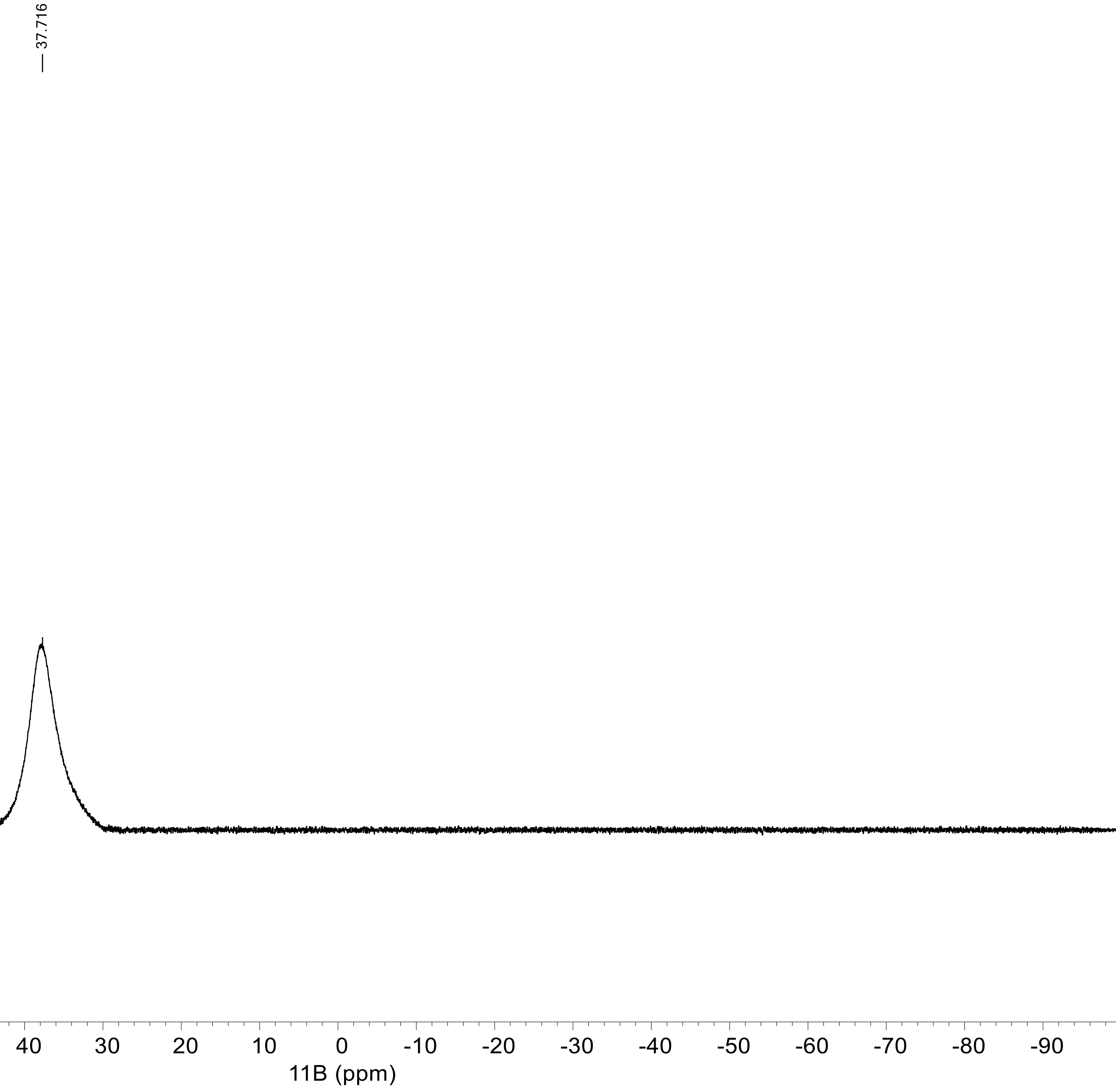

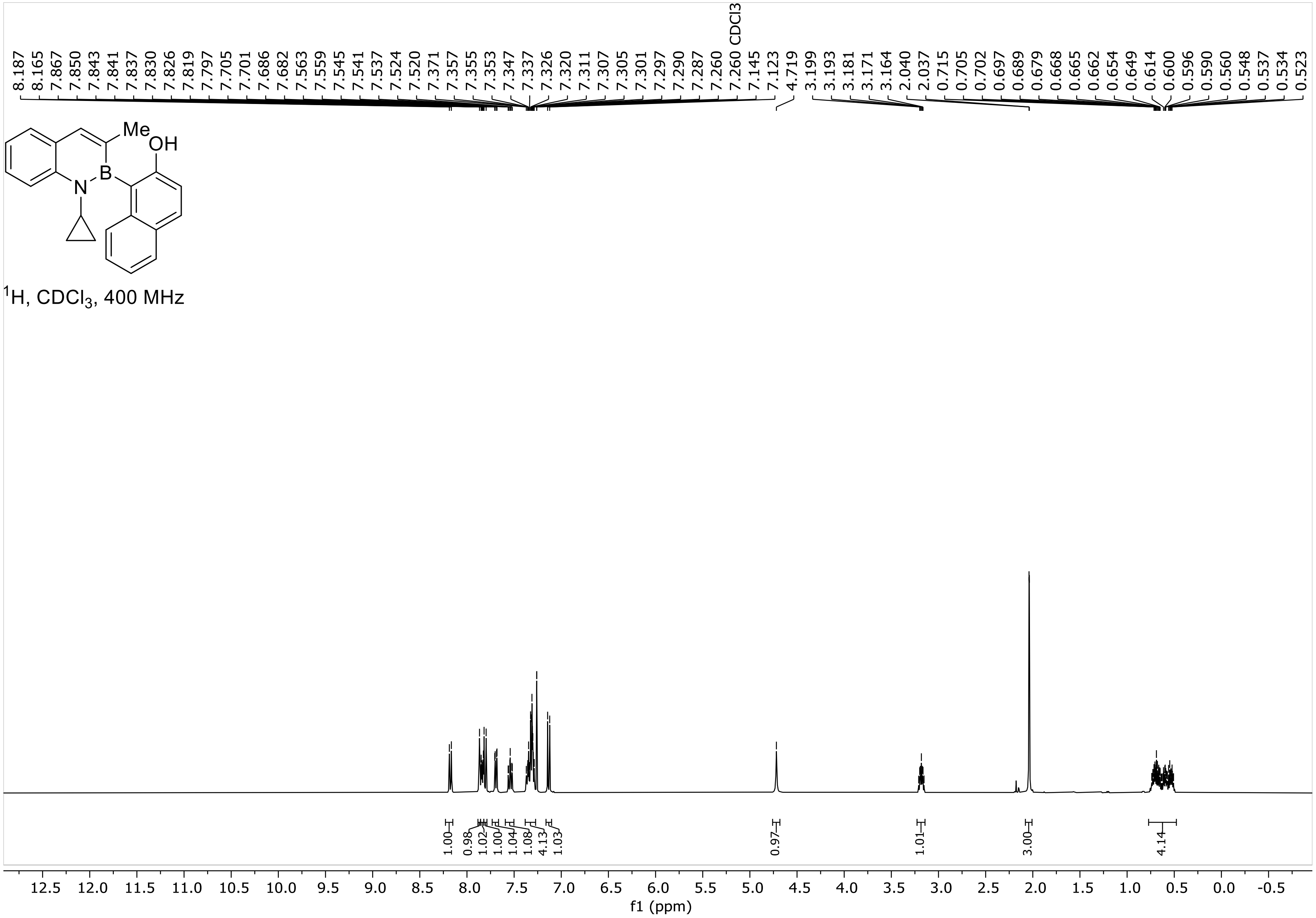
8.187 8.165 7.867 7.850 7.843 7.841 7.837 7.830 7.826 7.819 7.797 7.705 7.701 7.686 7.682 7.563 7.559 7.545 7.541 7.537 7.524 7.520 7.371 7.357 7.355 7.353 7.347 7.337 7.326 7.320 7.311 7.307 7.305 7.301 7.297 7.290 7.287 7.260 7.260 CDCl3 7.145 7.123 4.719 3.199 3.193 3.181 3.171 3.164 2.040 2.037 0.715 0.705 0.702 0.697 0.689 0.679 0.668 0.665 0.662 0.654 0.649 0.614 0.600 0.596 0.590 0.560 0.548 0.537 0.534 0.523
Me
OH
N
B
1H, CDCl3, 400 MHz
1.00 0.98 1.02 1.00 1.04 1.08 4.13 1.03 0.97 1.01 3.00 4.14
12.5 12.0 11.5 11.0 10.5 10.0 9.5 9.0 8.5 8.0 7.5 7.0 6.5 6.0 5.5 5.0 4.5 4.0 3.5 3.0 2.5 2.0 1.5 1.0 0.5 0.0 -0.5
f1 (ppm)

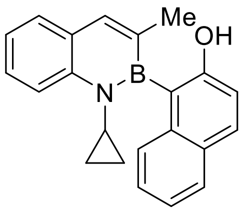

$^{13}C\ \{^{1}H\}$, $CDCl_3$, 126 MHz

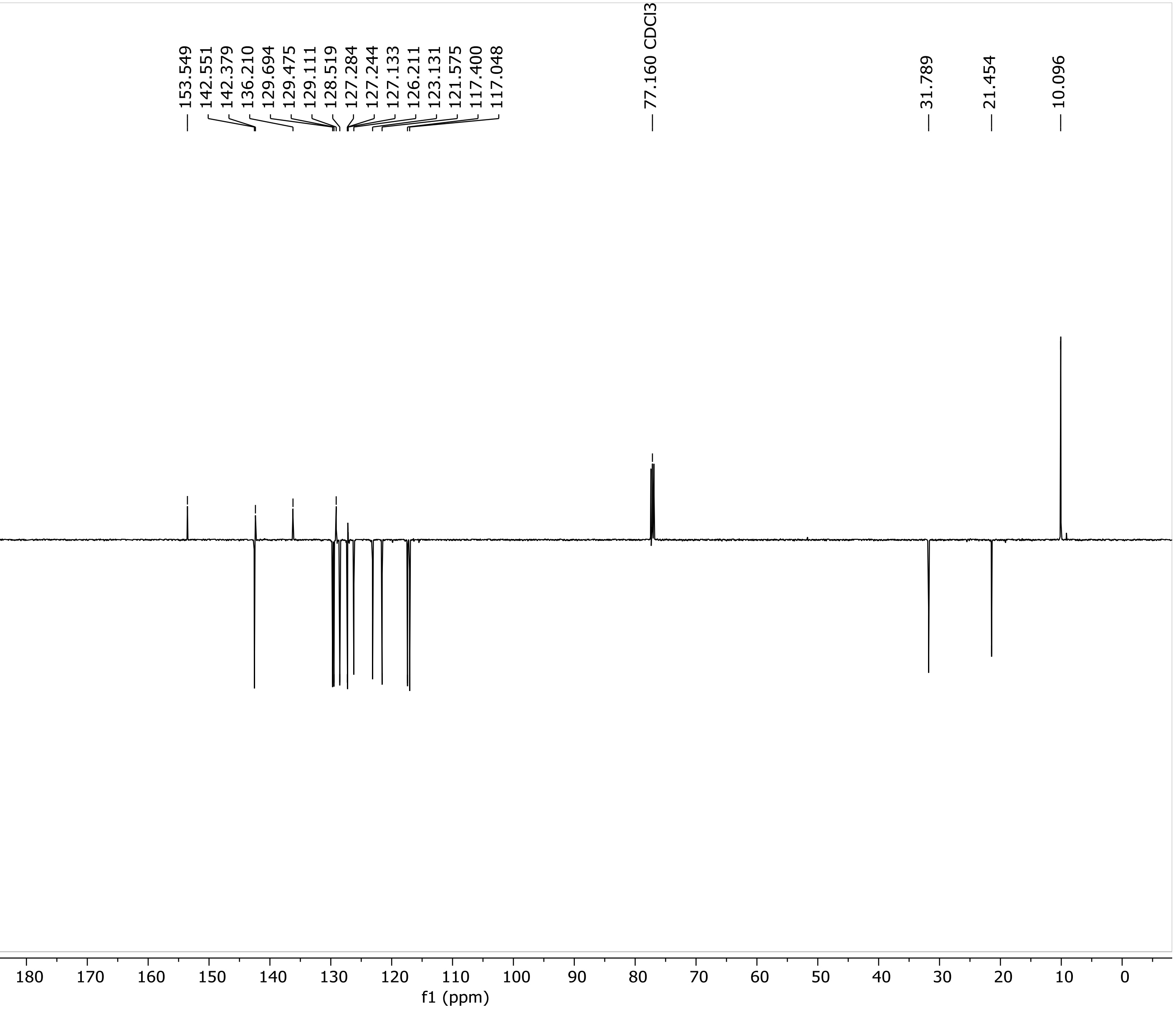

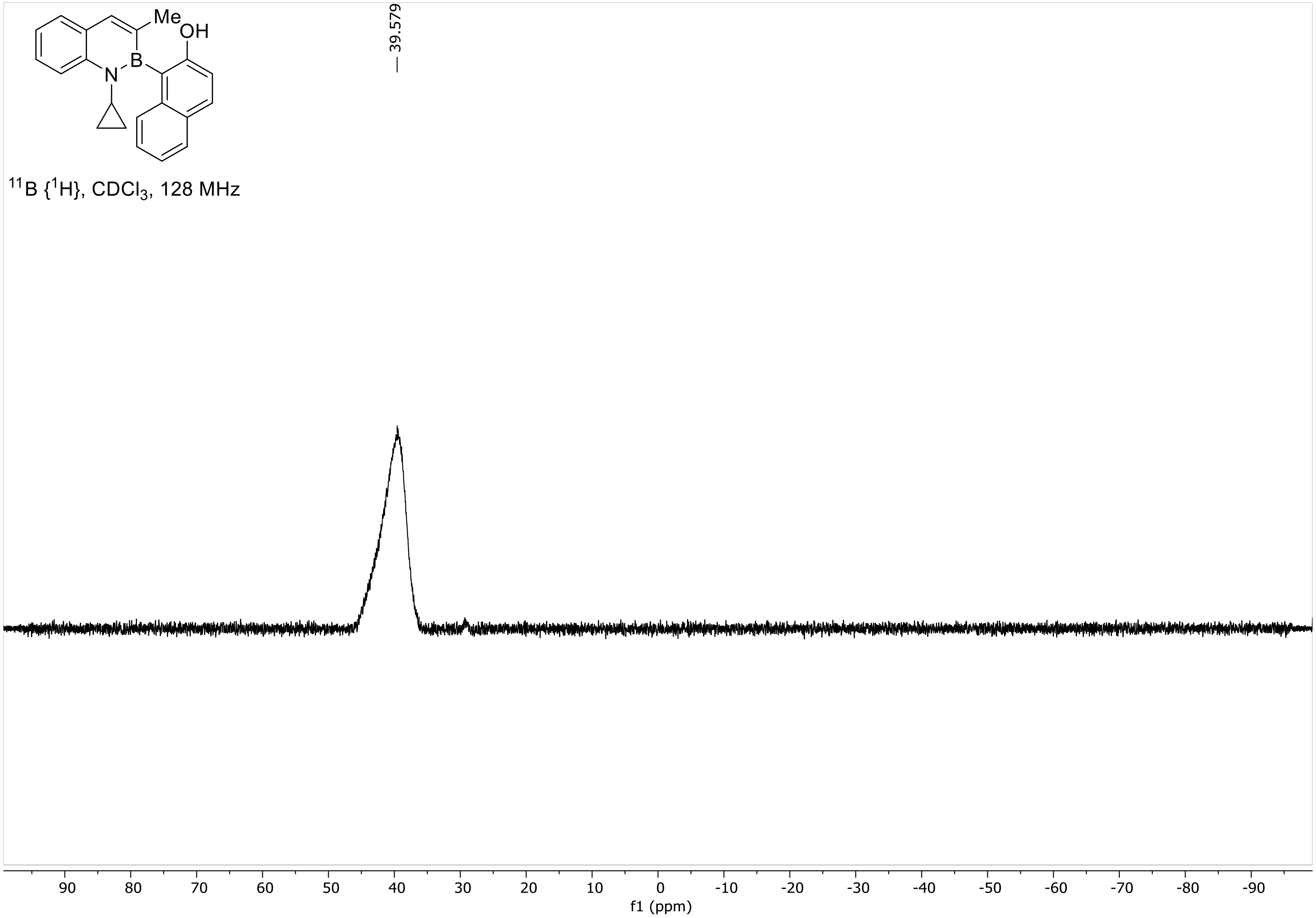
Me
OH
B
N
— 39.579
^{11}B {^{1}H}, CDCl$_3$, 128 MHz
90
80
70
60
50
40
30
20
10
0
-10
-20
-30
-40
-50
-60
-70
-80
-90
f1 (ppm)

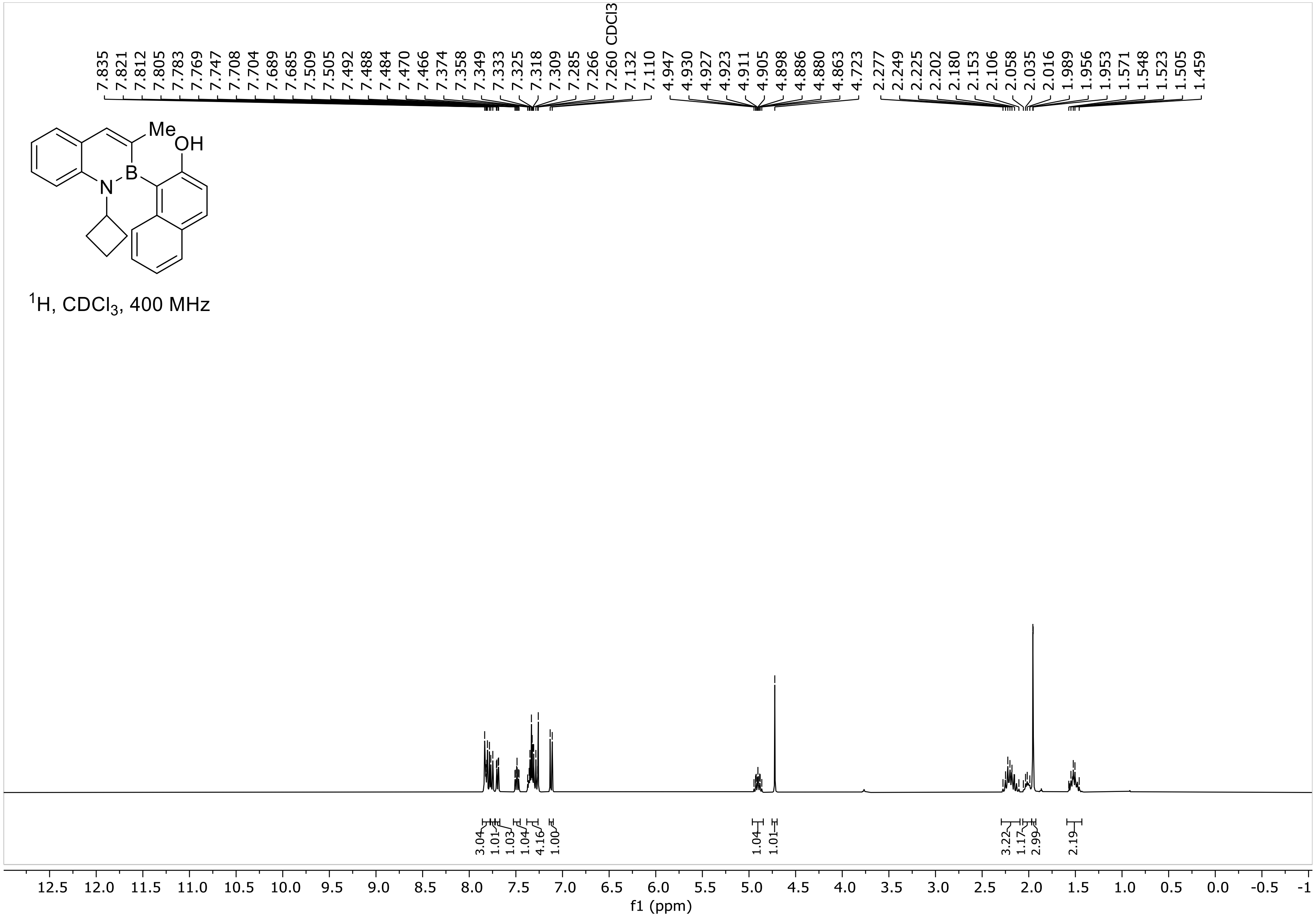

7.835
7.821
7.812
7.805
7.783
7.769
7.747
7.708
7.704
7.689
7.685
7.509
7.505
7.492
7.488
7.484
7.470
7.466
7.374
7.358
7.349
7.333
7.325
7.318
7.309
7.285
7.266
7.260 CDCl3
7.132
7.110
4.947
4.930
4.927
4.923
4.911
4.905
4.898
4.886
4.880
4.863
4.723
2.277
2.249
2.225
2.202
2.180
2.153
2.106
2.058
2.035
2.016
1.989
1.956
1.953
1.571
1.548
1.523
1.505
1.459
Me
OH
B
N
^{1}H, CDCl$_3$, 400 MHz
3.04
1.01
1.03
1.04
4.16
1.00
1.04
1.01
3.22
1.17
2.99
2.19
12.5 12.0 11.5 11.0 10.5 10.0 9.5 9.0 8.5 8.0 7.5 7.0 6.5 6.0 5.5 5.0 4.5 4.0 3.5 3.0 2.5 2.0 1.5 1.0 0.5 0.0 -0.5 -1
f1 (ppm)

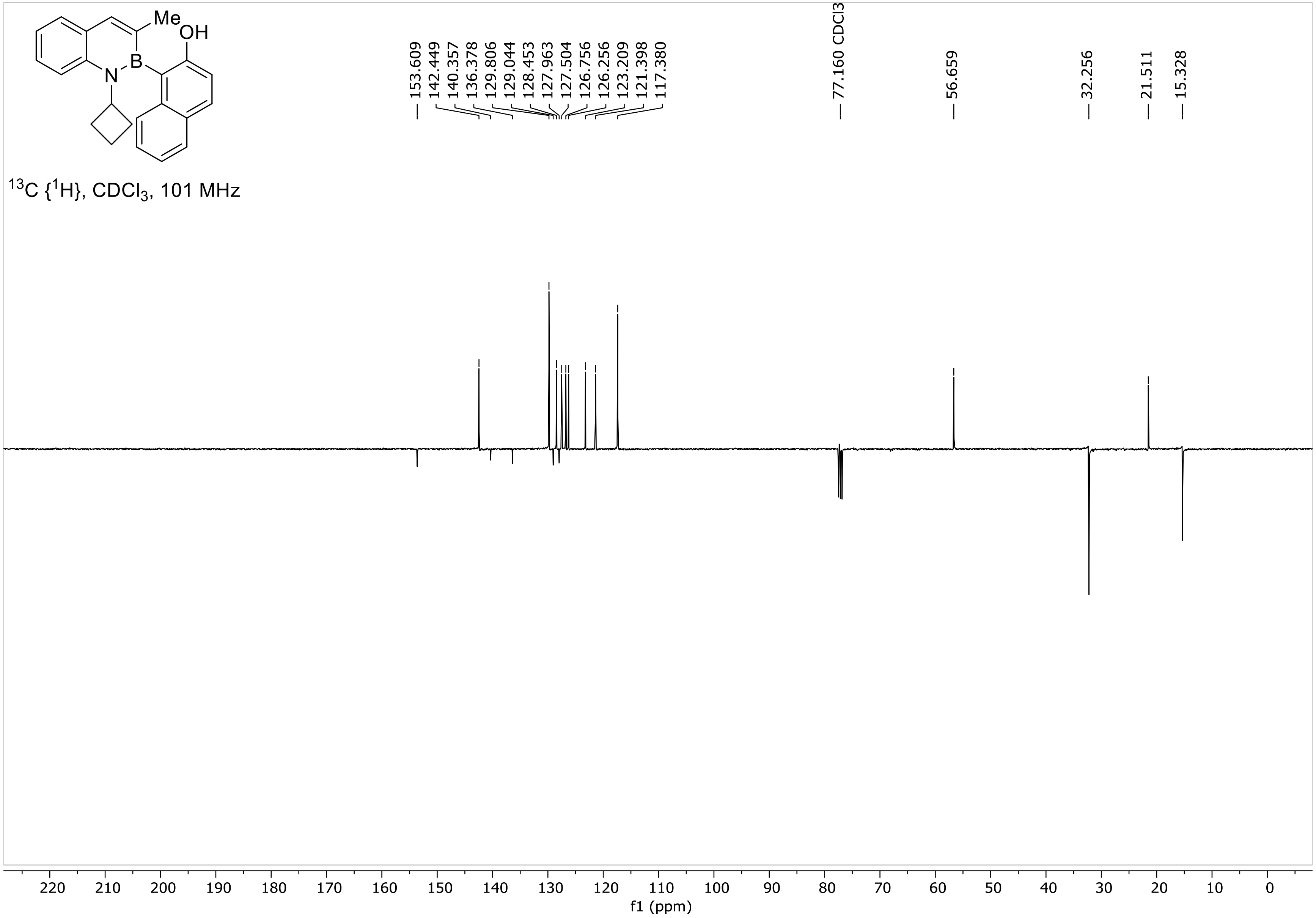
Me
OH
N
B
13C {1H}, CDCl3, 101 MHz
153.609
142.449
140.357
136.378
129.806
129.044
128.453
127.963
127.504
126.756
126.256
123.209
121.398
117.380
77.160 CDCl3
56.659
32.256
21.511
15.328
220
210
200
190
180
170
160
150
140
130
120
110
100
90
80
70
60
50
40
30
20
10
0
f1 (ppm)

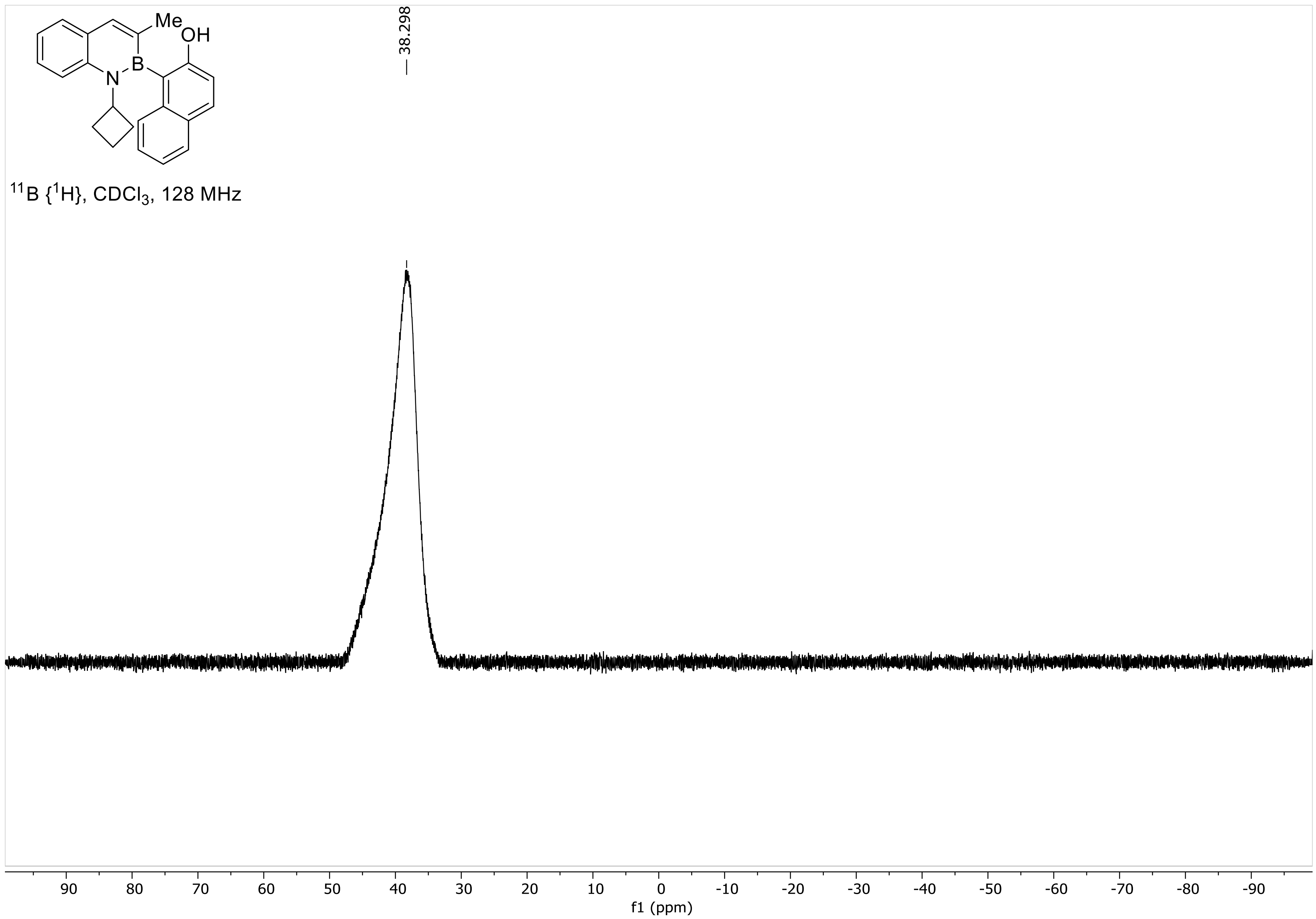
Me
OH
B
N
38.298
11B {1H}, CDCl3, 128 MHz
90
80
70
60
50
40
30
20
10
0
-10
-20
-30
-40
-50
-60
-70
-80
-90
f1 (ppm)

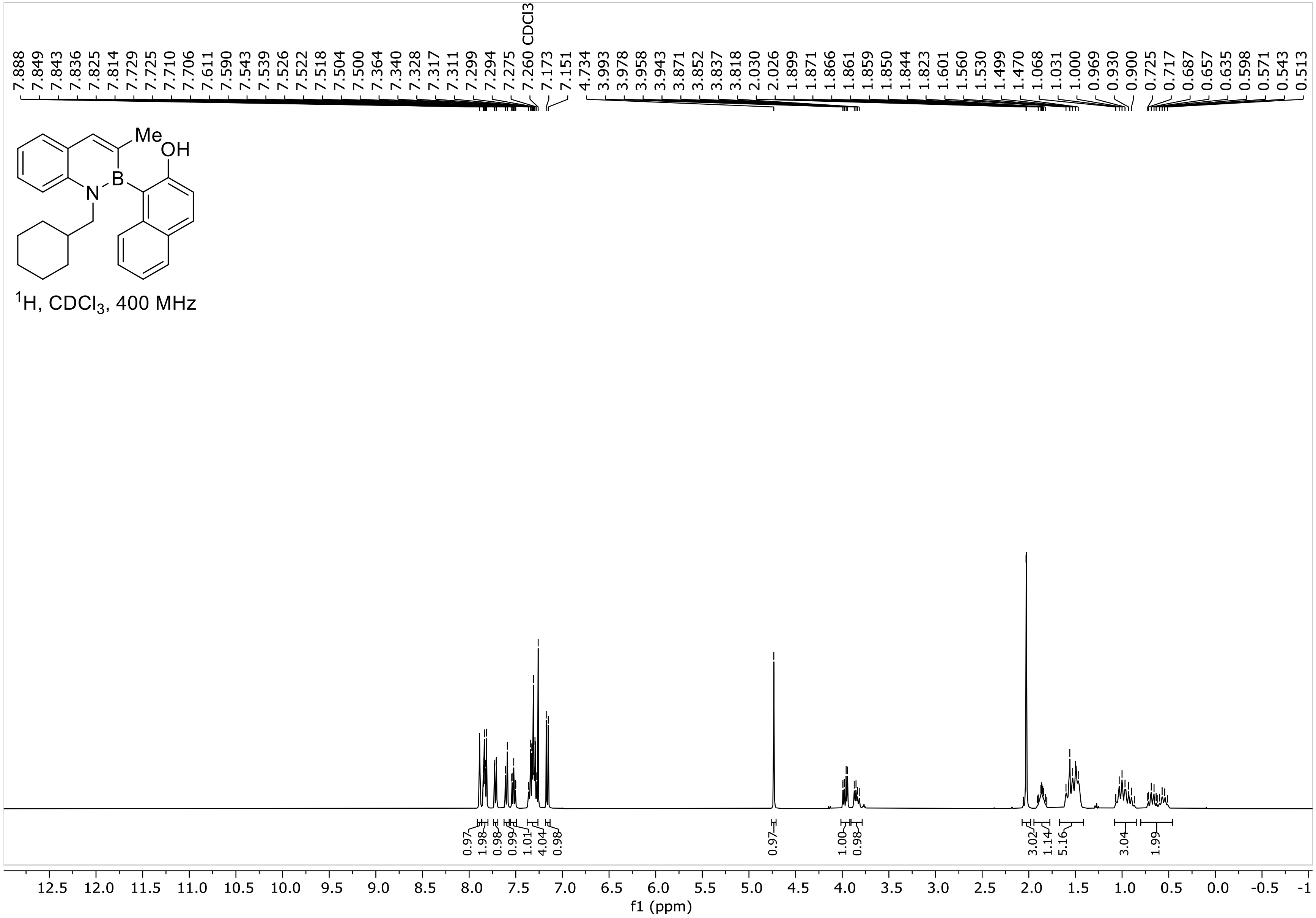

Me
OH
B
N
^{1}H, CDCl$_3$, 400 MHz
CDCl3
7.888 7.849 7.843 7.836 7.825 7.814 7.729 7.725 7.710 7.706 7.611 7.590 7.543 7.539 7.526 7.522 7.518 7.504 7.500 7.364 7.340 7.328 7.317 7.311 7.299 7.294 7.275 7.260 7.173 7.151 4.734 3.993 3.978 3.958 3.943 3.871 3.852 3.837 3.818 2.030 2.026 1.899 1.871 1.866 1.861 1.859 1.850 1.844 1.823 1.601 1.560 1.530 1.499 1.470 1.068 1.031 1.000 0.969 0.930 0.900 0.725 0.717 0.687 0.657 0.635 0.598 0.571 0.543 0.513
0.97 1.98 0.98 0.99 1.01 4.04 0.98 0.97 1.00 0.98 3.02 1.14 5.16 3.04 1.99
12.5 12.0 11.5 11.0 10.5 10.0 9.5 9.0 8.5 8.0 7.5 7.0 6.5 6.0 5.5 5.0 4.5 4.0 3.5 3.0 2.5 2.0 1.5 1.0 0.5 0.0 -0.5 -1
f1 (ppm)

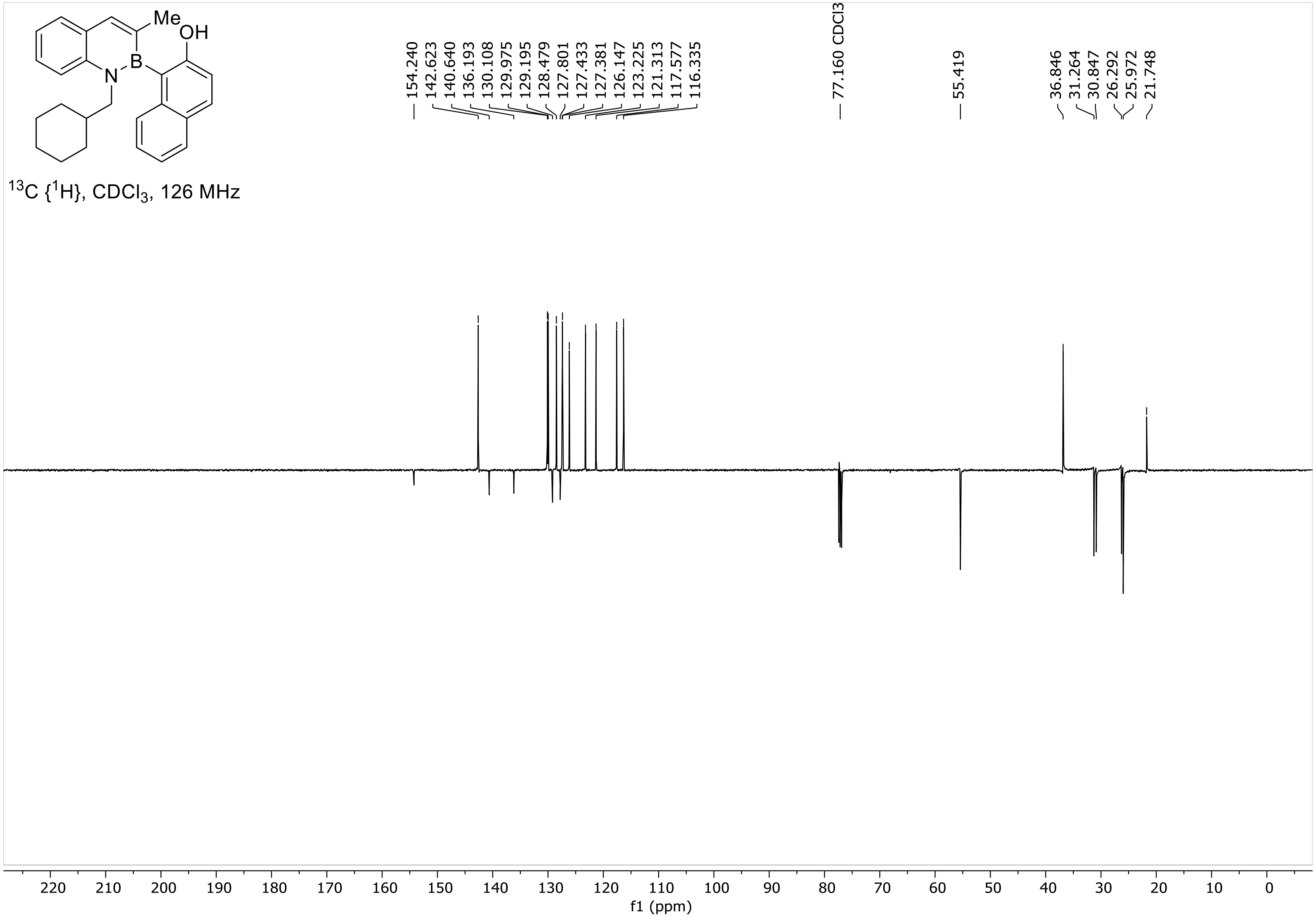
Me
OH
N
B
^{13}C {^{1}H}, CDCl$_3$, 126 MHz
154.240
142.623
140.640
136.193
130.108
129.975
129.195
128.479
127.801
127.433
127.381
126.147
123.225
121.313
117.577
116.335
77.160 CDCl3
55.419
36.846
31.264
30.847
26.292
25.972
21.748
220 210 200 190 180 170 160 150 140 130 120 110 100 90 80 70 60 50 40 30 20 10 0
f1 (ppm)

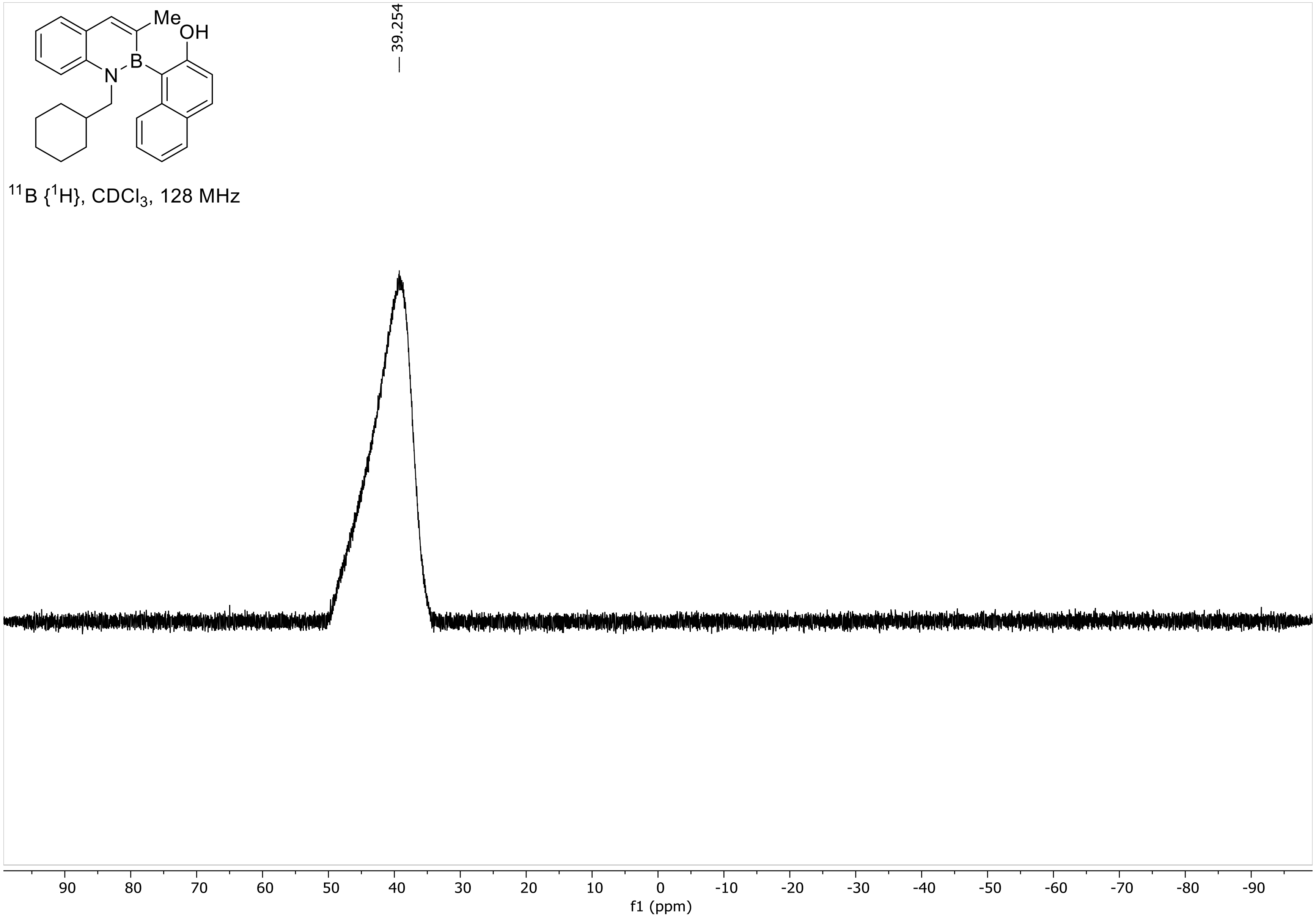
Me
OH
B
N
39.254
^{11}B {^{1}H}, CDCl$_3$, 128 MHz
90
80
70
60
50
40
30
20
10
0
-10
-20
-30
-40
-50
-60
-70
-80
-90
f1 (ppm)

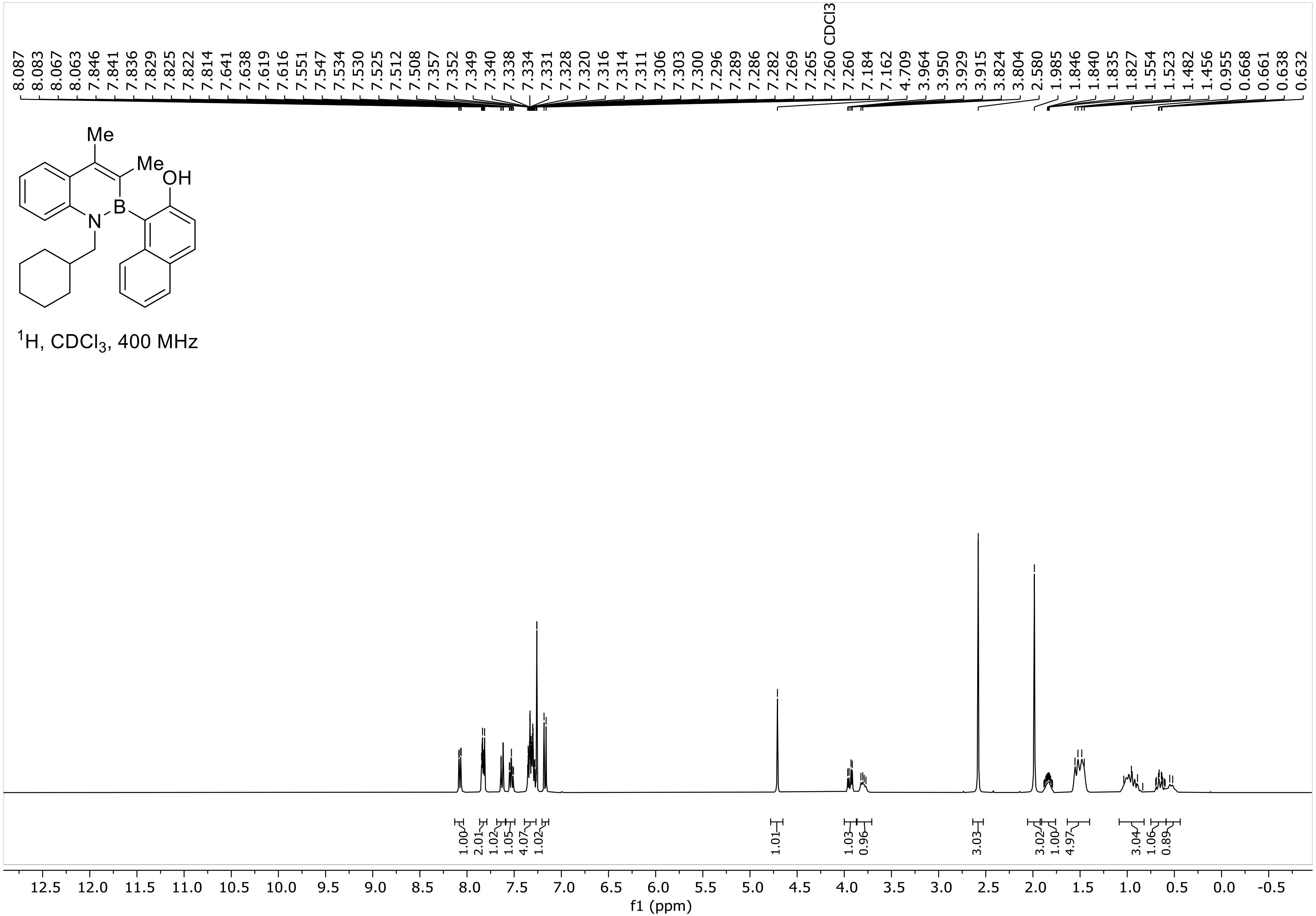
8.087
8.083
8.067
8.063
7.846
7.841
7.836
7.829
7.825
7.822
7.814
7.641
7.638
7.619
7.616
7.551
7.547
7.534
7.530
7.525
7.512
7.508
7.357
7.352
7.349
7.340
7.338
7.334
7.331
7.328
7.320
7.316
7.314
7.311
7.306
7.303
7.300
7.296
7.289
7.286
7.282
7.269
7.265
7.260 CDCl3
7.260
7.184
7.162
4.709
3.964
3.950
3.929
3.915
3.824
3.804
2.580
1.985
1.846
1.840
1.835
1.827
1.554
1.523
1.482
1.456
0.955
0.668
0.661
0.638
0.632
Me
Me
OH
B
N
¹H, CDCl₃, 400 MHz
1.00
2.01
1.02
1.05
4.07
1.02
1.01
1.03
0.96
3.03
3.02
1.00
4.97
3.04
1.06
0.89
12.5 12.0 11.5 11.0 10.5 10.0 9.5 9.0 8.5 8.0 7.5 7.0 6.5 6.0 5.5 5.0 4.5 4.0 3.5 3.0 2.5 2.0 1.5 1.0 0.5 0.0 -0.5
f1 (ppm)

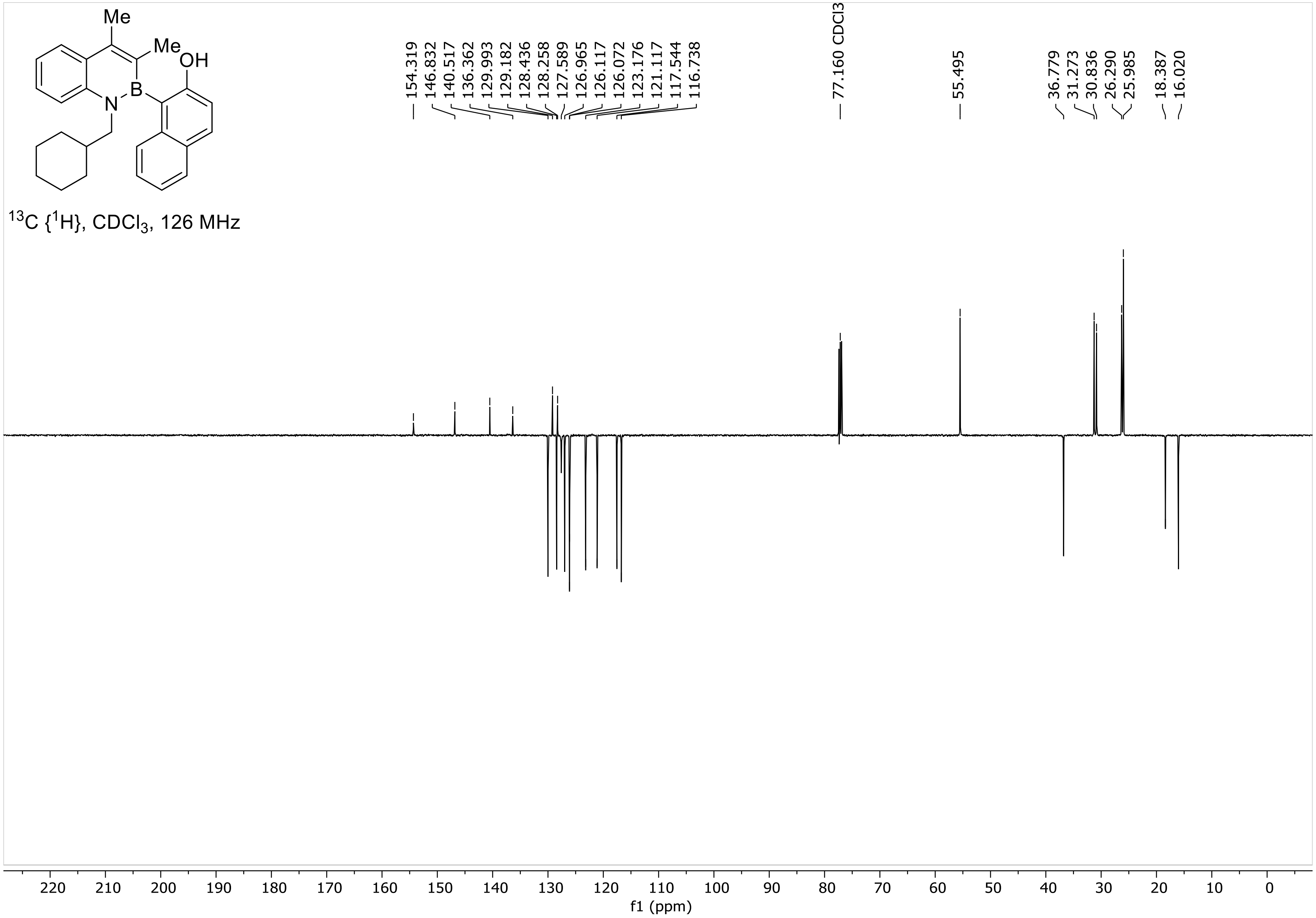

Me
Me
OH
N
B
¹³C {¹H}, CDCl₃, 126 MHz
154.319
146.832
140.517
136.362
129.993
129.182
128.436
128.258
127.589
126.965
126.117
126.072
123.176
121.117
117.544
116.738
77.160 CDCl3
55.495
36.779
31.273
30.836
26.290
25.985
18.387
16.020
f1 (ppm)

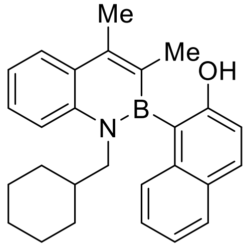

$^{11}B\ \{^1H\}$, $CDCl_3$, 128 MHz

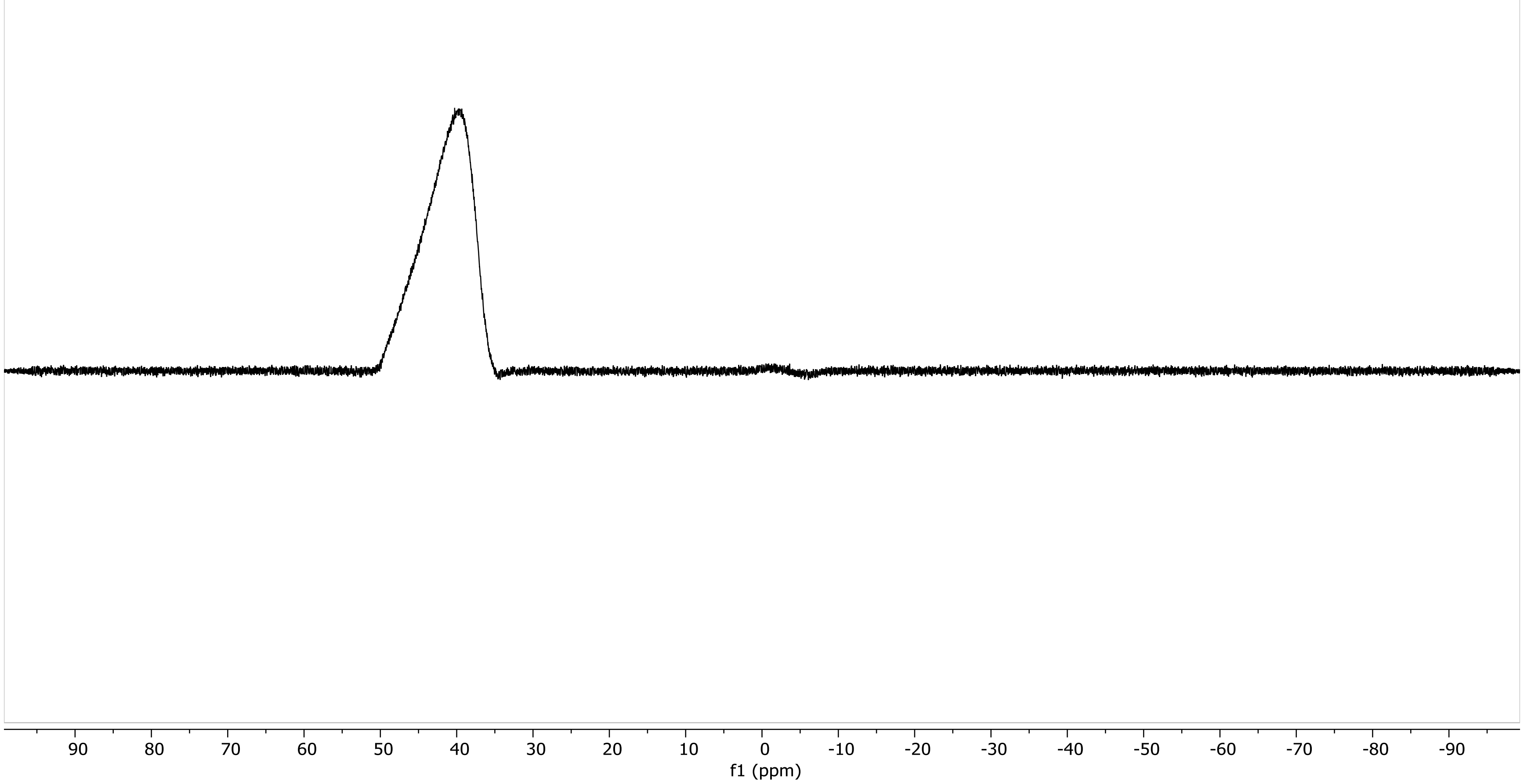

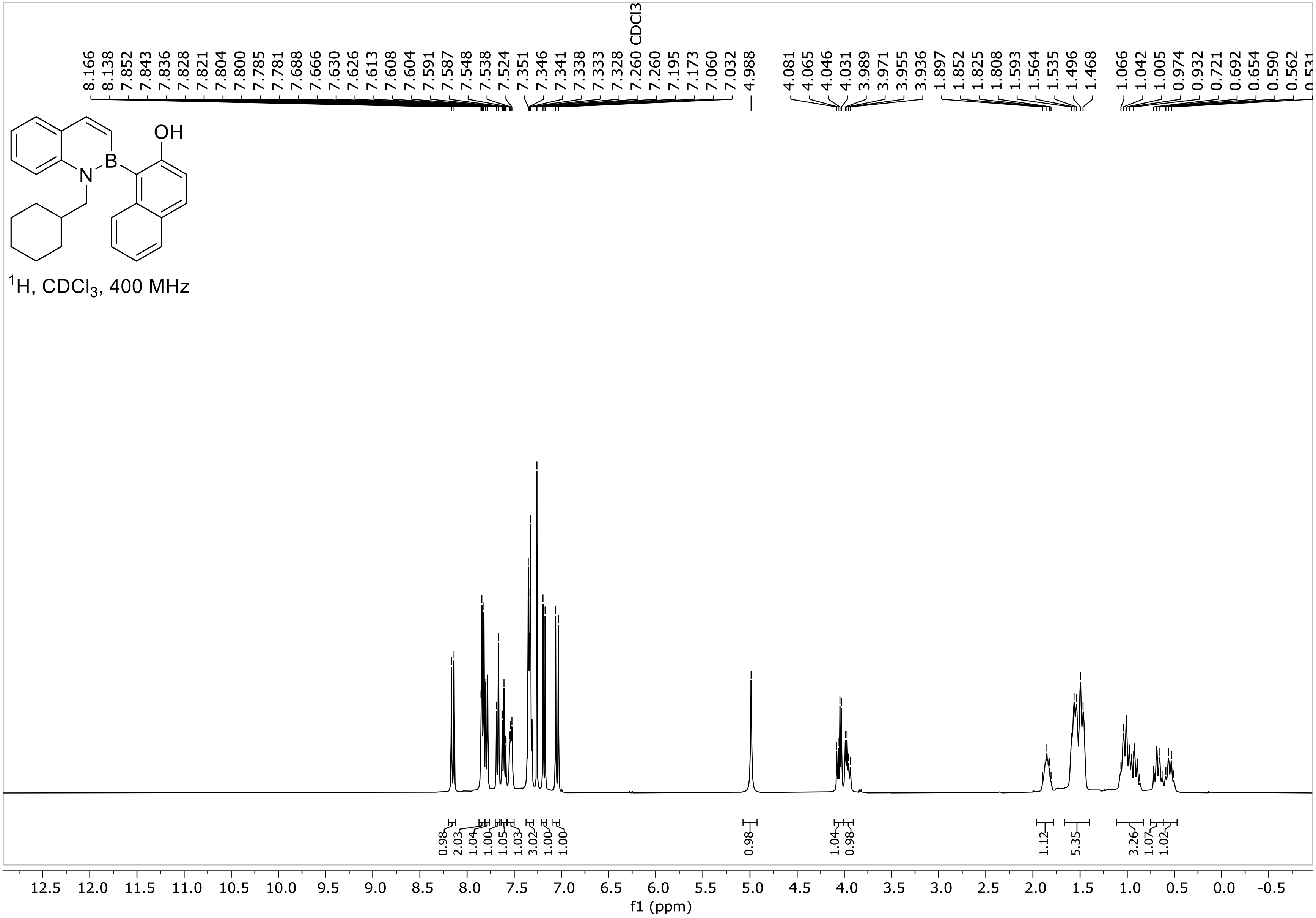
OH
N
B
^{1}H, $CDCl_3$, 400 MHz
CDCl3
8.166 8.138 7.852 7.843 7.836 7.828 7.821 7.804 7.800 7.785 7.781 7.688 7.666 7.630 7.626 7.613 7.608 7.604 7.591 7.587 7.548 7.538 7.524 7.351 7.346 7.341 7.338 7.333 7.328 7.260 7.260 7.195 7.173 7.060 7.032 4.988 4.081 4.065 4.046 4.031 3.989 3.971 3.955 3.936 1.897 1.852 1.825 1.808 1.593 1.564 1.535 1.496 1.468 1.066 1.042 1.005 0.974 0.932 0.721 0.692 0.654 0.590 0.562
0.98 2.03 1.04 1.00 1.05 1.03 3.02 1.00 1.00 0.98 1.04 0.98 1.12 5.35 3.26 1.07 1.02
12.5 12.0 11.5 11.0 10.5 10.0 9.5 9.0 8.5 8.0 7.5 7.0 6.5 6.0 5.5 5.0 4.5 4.0 3.5 3.0 2.5 2.0 1.5 1.0 0.5 0.0 -0.5
f1 (ppm)

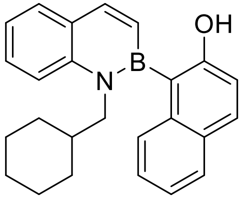

^{13}C {^{1}H}, $CDCl_3$, 126 MHz

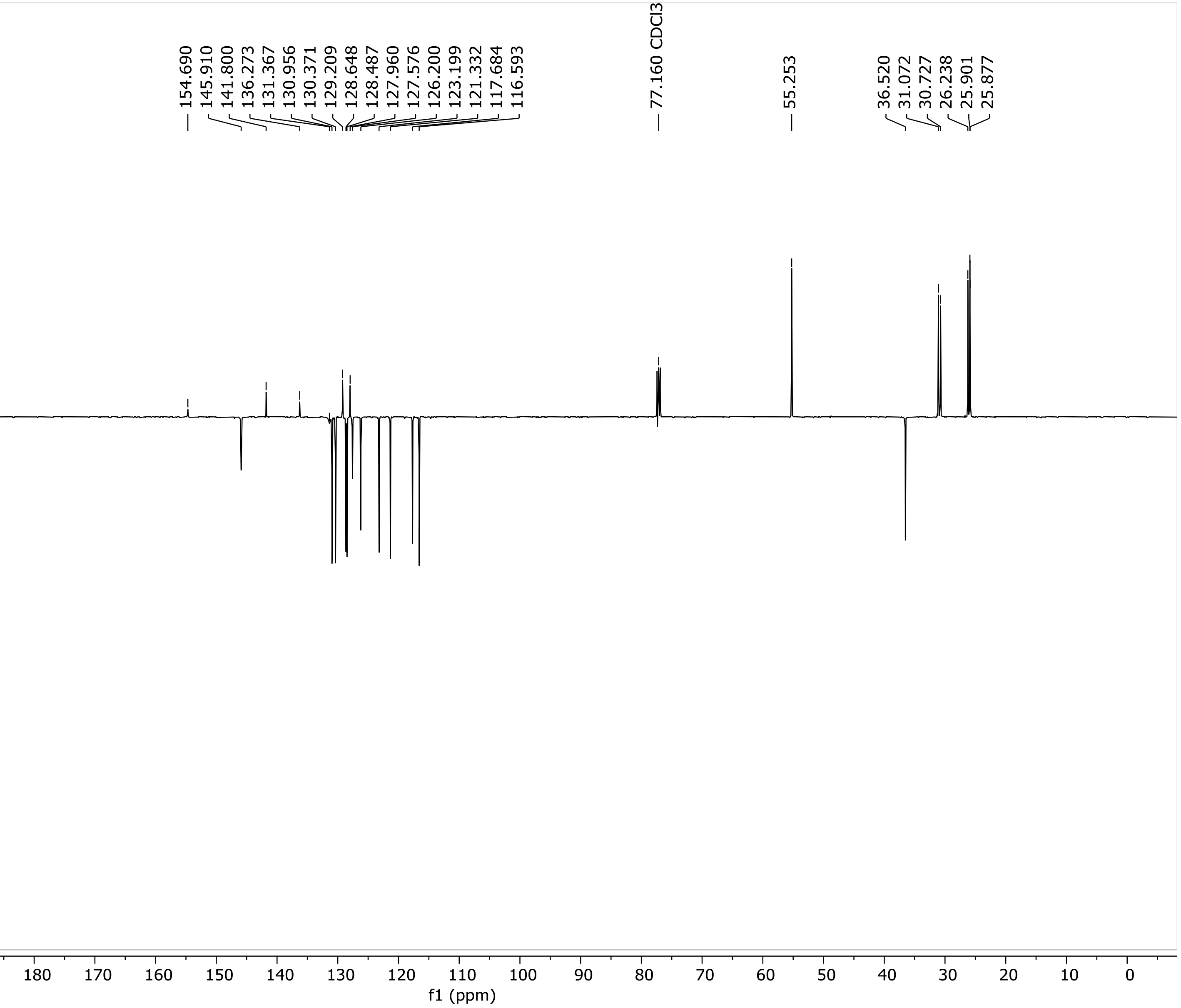

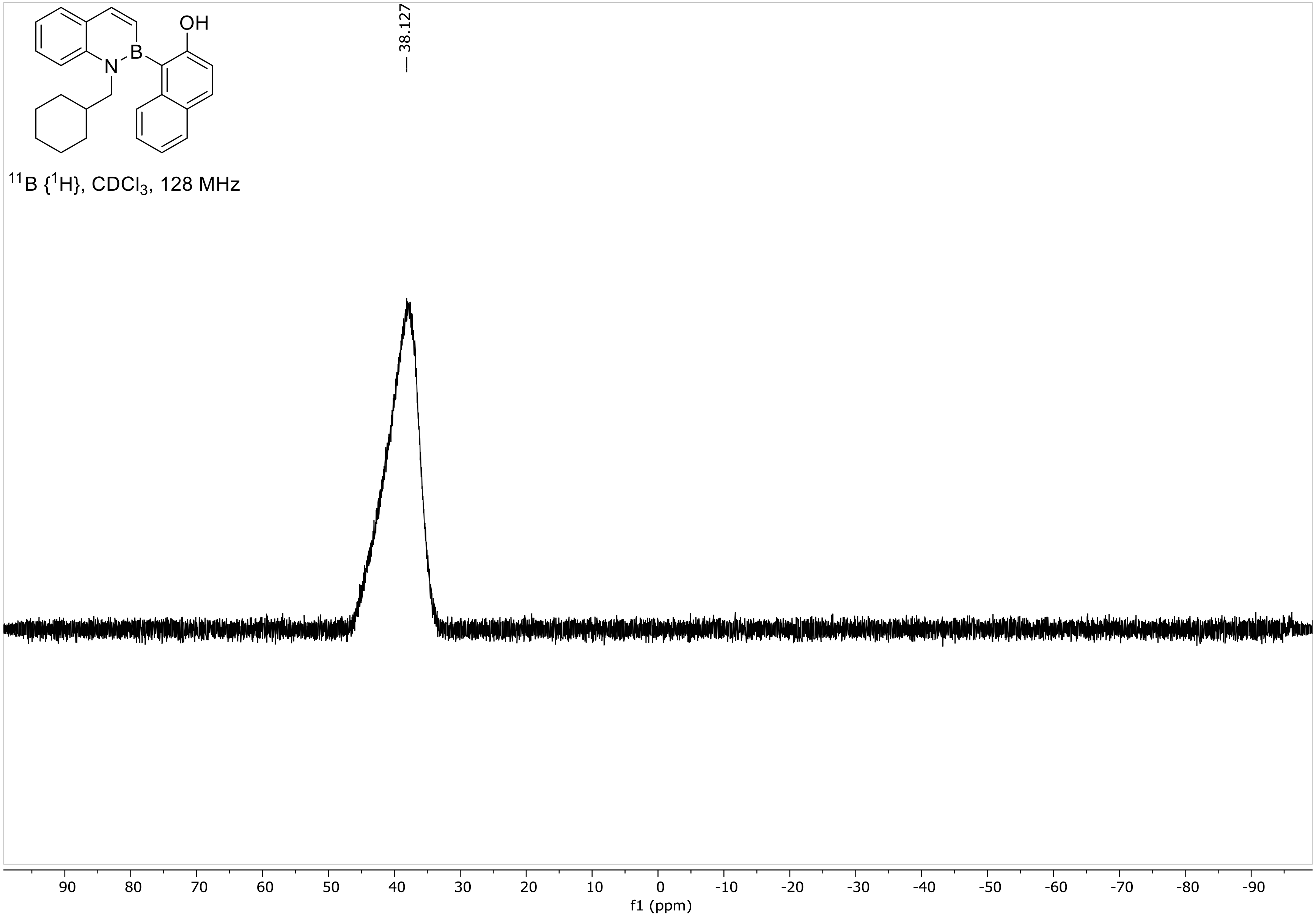
38.127
$^{11}B\ \{^{1}H\}$, $CDCl_3$, 128 MHz
OH
N
B
90 80 70 60 50 40 30 20 10 0 -10 -20 -30 -40 -50 -60 -70 -80 -90
f1 (ppm)

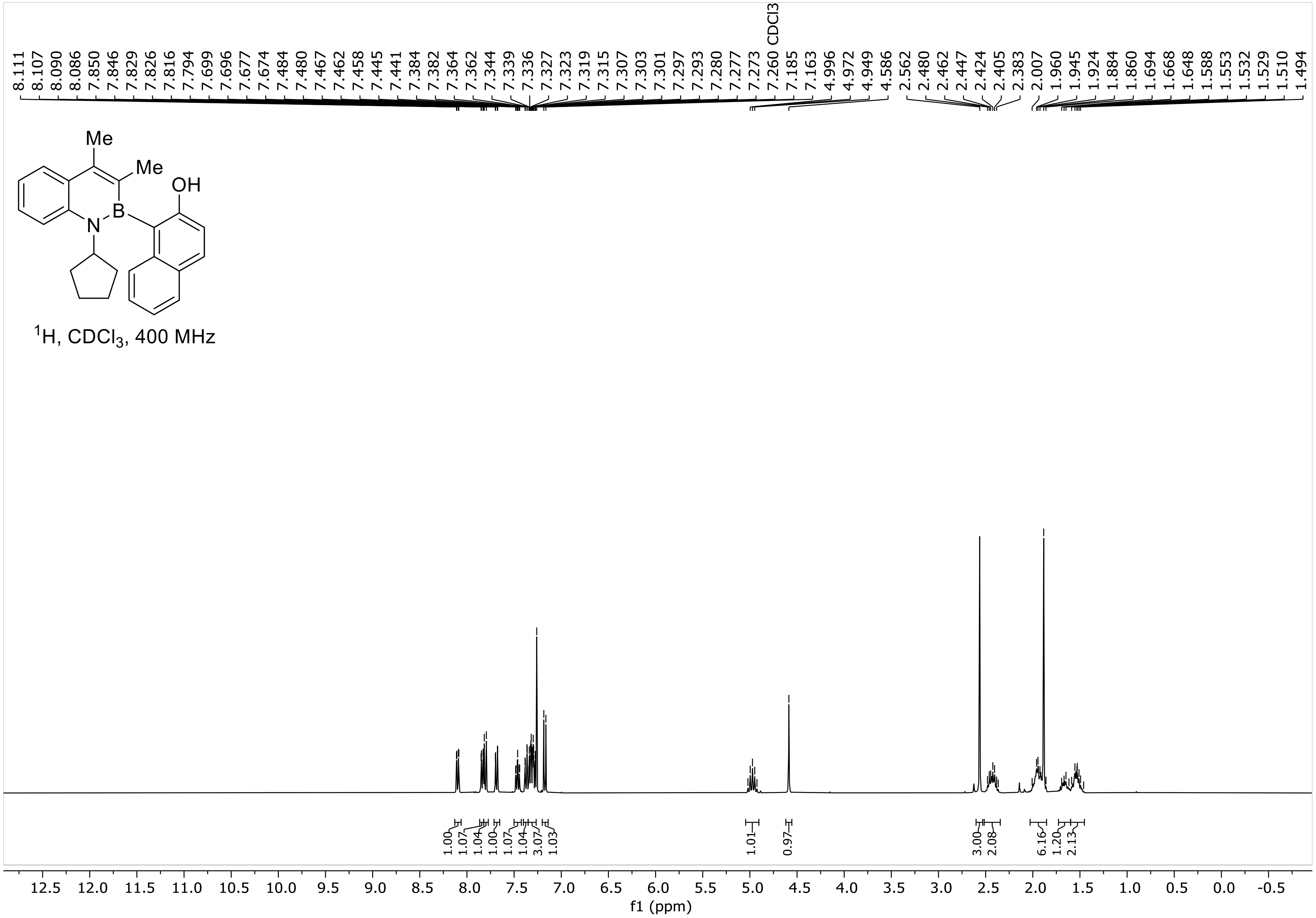
Me
Me
OH
N
B
^{1}H, CDCl$_3$, 400 MHz
CDCl3
8.111 8.107 8.090 8.086 7.850 7.846 7.829 7.826 7.816 7.794 7.699 7.696 7.677 7.674 7.484 7.480 7.467 7.462 7.458 7.445 7.441 7.384 7.382 7.364 7.362 7.344 7.339 7.336 7.327 7.323 7.319 7.315 7.307 7.303 7.301 7.297 7.293 7.280 7.277 7.273 7.260 7.185 7.163 4.996 4.972 4.949 4.586 2.562 2.480 2.462 2.447 2.424 2.405 2.383 2.007 1.960 1.945 1.924 1.884 1.860 1.694 1.668 1.648 1.588 1.553 1.532 1.529 1.510 1.494
1.00 1.07 1.04 1.00 1.07 1.04 3.07 1.03 1.01 0.97 3.00 2.08 6.16 1.20 2.13
12.5 12.0 11.5 11.0 10.5 10.0 9.5 9.0 8.5 8.0 7.5 7.0 6.5 6.0 5.5 5.0 4.5 4.0 3.5 3.0 2.5 2.0 1.5 1.0 0.5 0.0 -0.5
f1 (ppm)

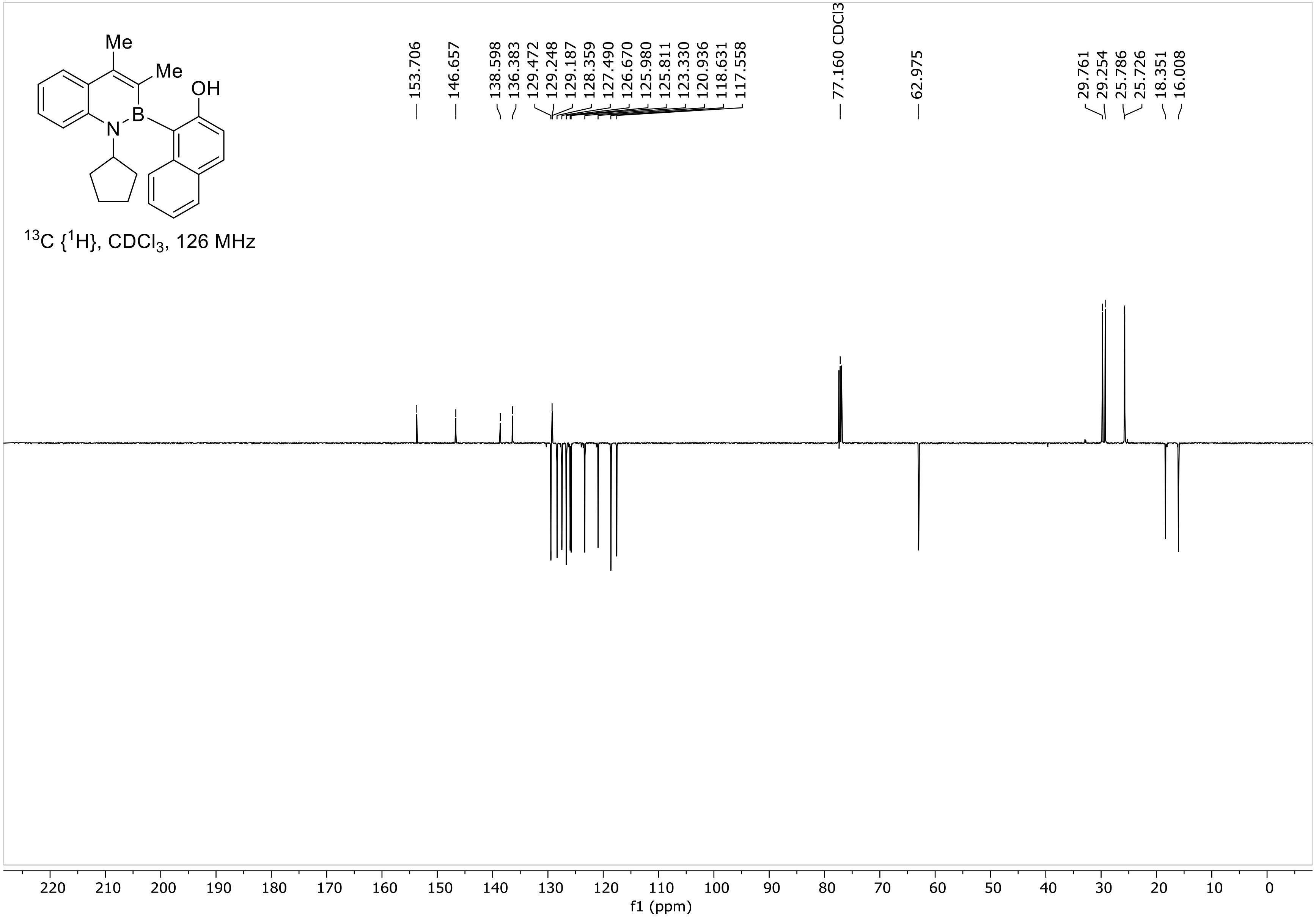
Me
Me
OH
B
N
^{13}C {^1H}, $CDCl_3$, 126 MHz
153.706
146.657
138.598
136.383
129.472
129.248
129.187
128.359
127.490
126.670
125.980
125.811
123.330
120.936
118.631
117.558
77.160 CDCl3
62.975
29.761
29.254
25.786
25.726
18.351
16.008
220
210
200
190
180
170
160
150
140
130
120
110
100
90
80
70
60
50
40
30
20
10
0
f1 (ppm)

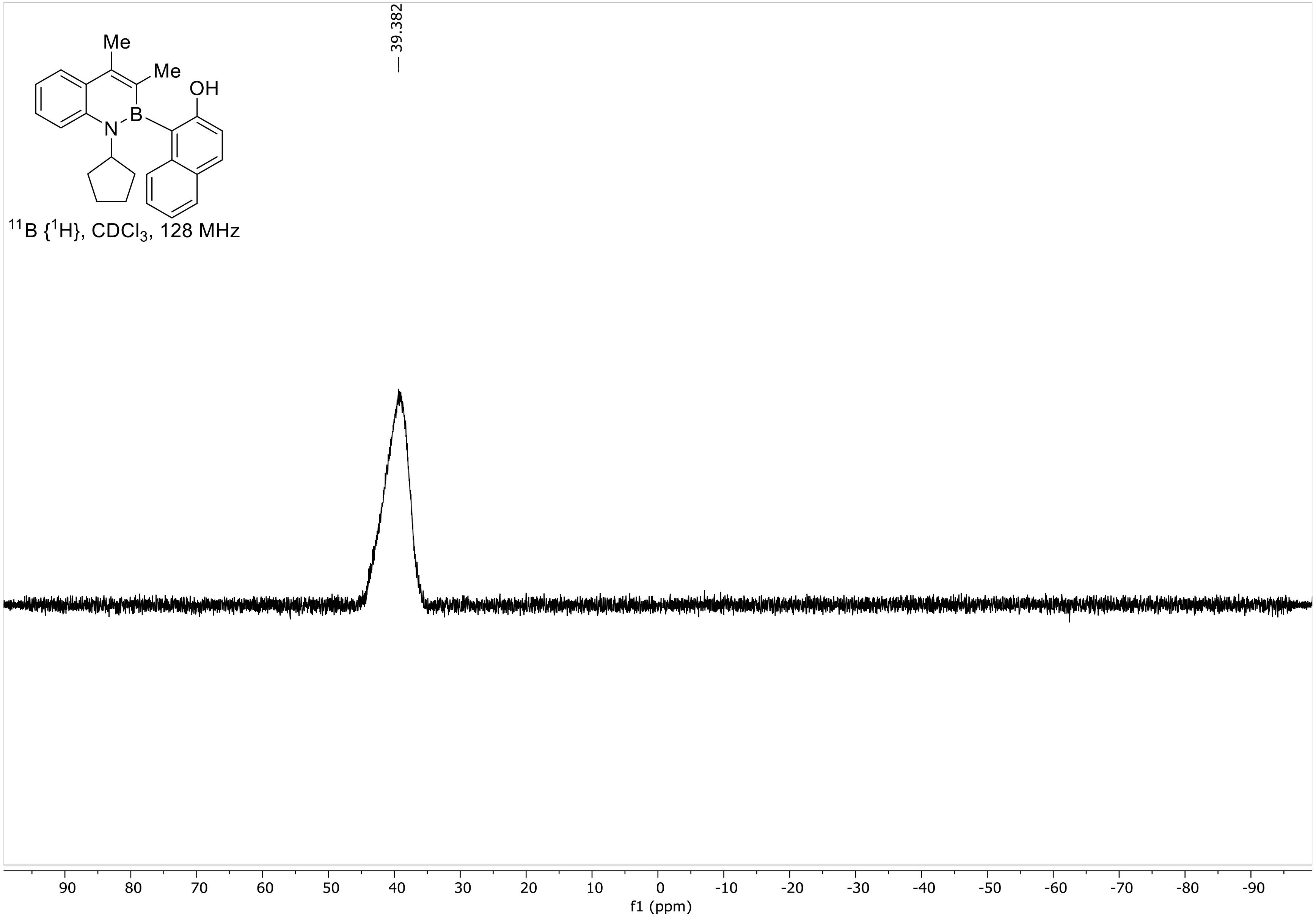

Me
Me
OH
B
N
^{11}B {^{1}H}, $CDCl_3$, 128 MHz
39.382
90
80
70
60
50
40
30
20
10
0
-10
-20
-30
-40
-50
-60
-70
-80
-90
f1 (ppm)

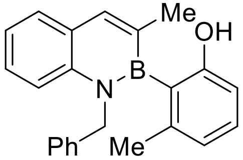

^{1}H, $CDCl_3$, 400 MHz

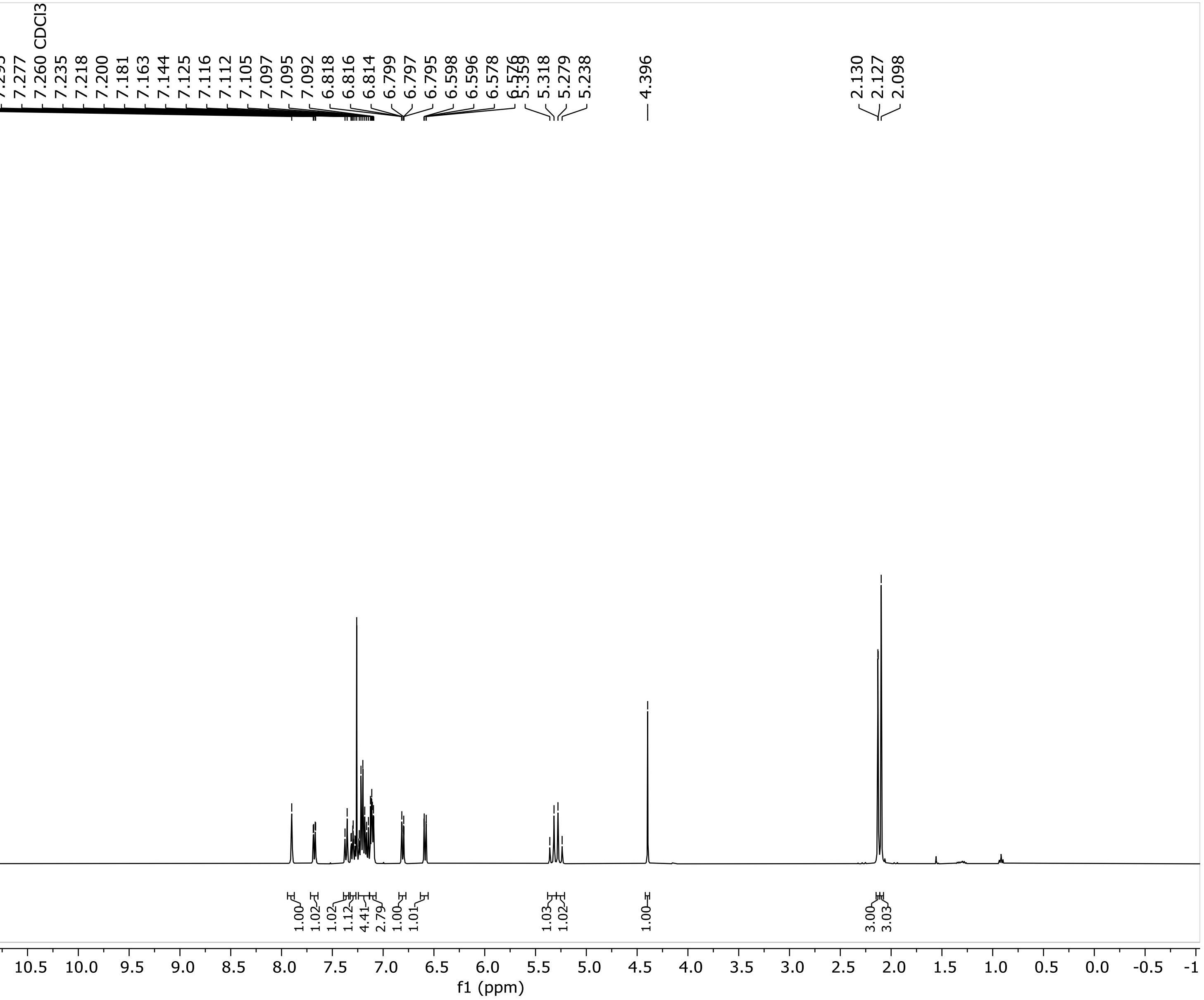

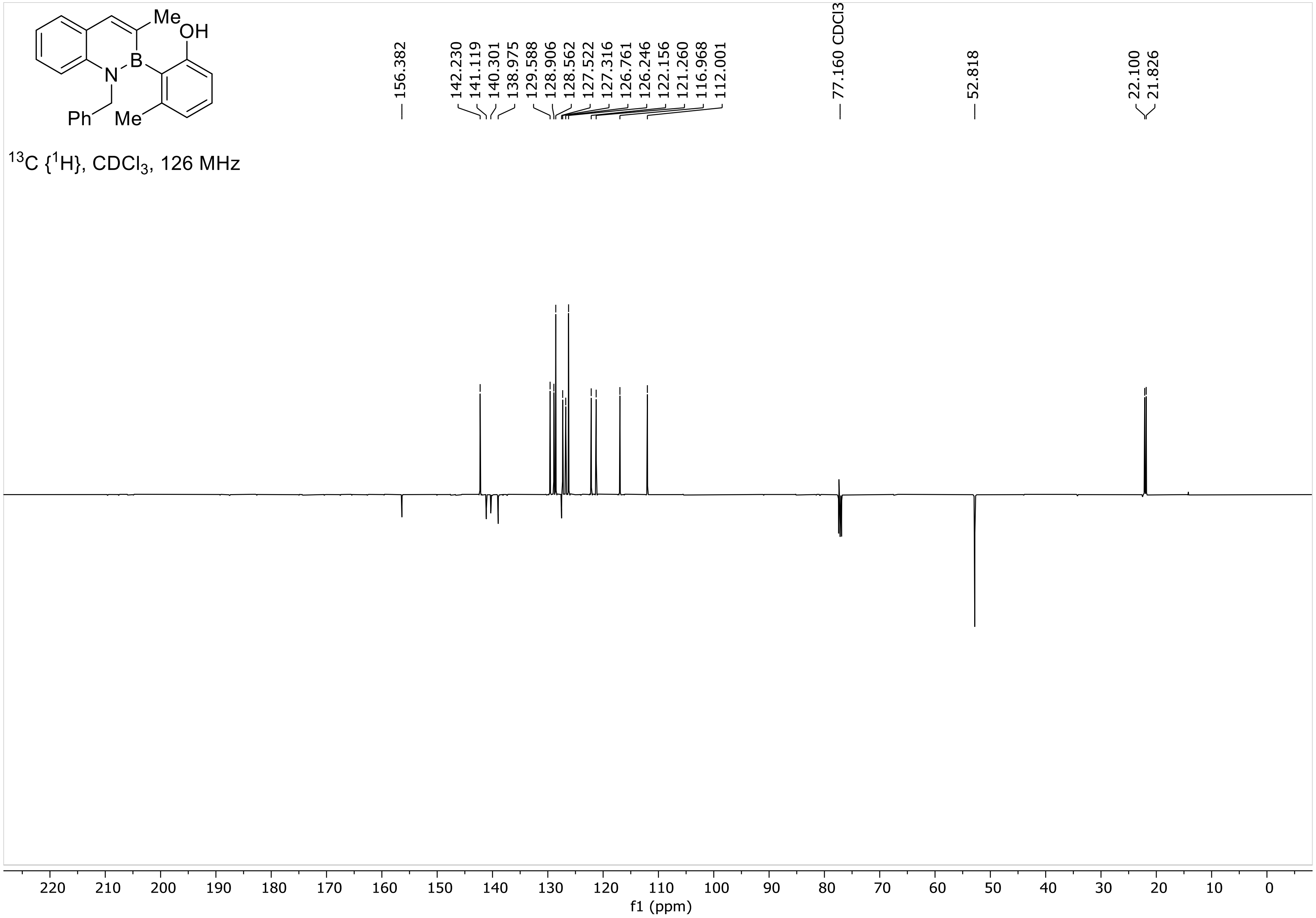
Me
OH
B
N
Ph
Me
^{13}C {^{1}H}, CDCl$_3$, 126 MHz
156.382
142.230
141.119
140.301
138.975
129.588
128.906
128.562
127.522
127.316
126.761
126.246
122.156
121.260
116.968
112.001
77.160 CDCl3
52.818
22.100
21.826
220 210 200 190 180 170 160 150 140 130 120 110 100 90 80 70 60 50 40 30 20 10 0
f1 (ppm)

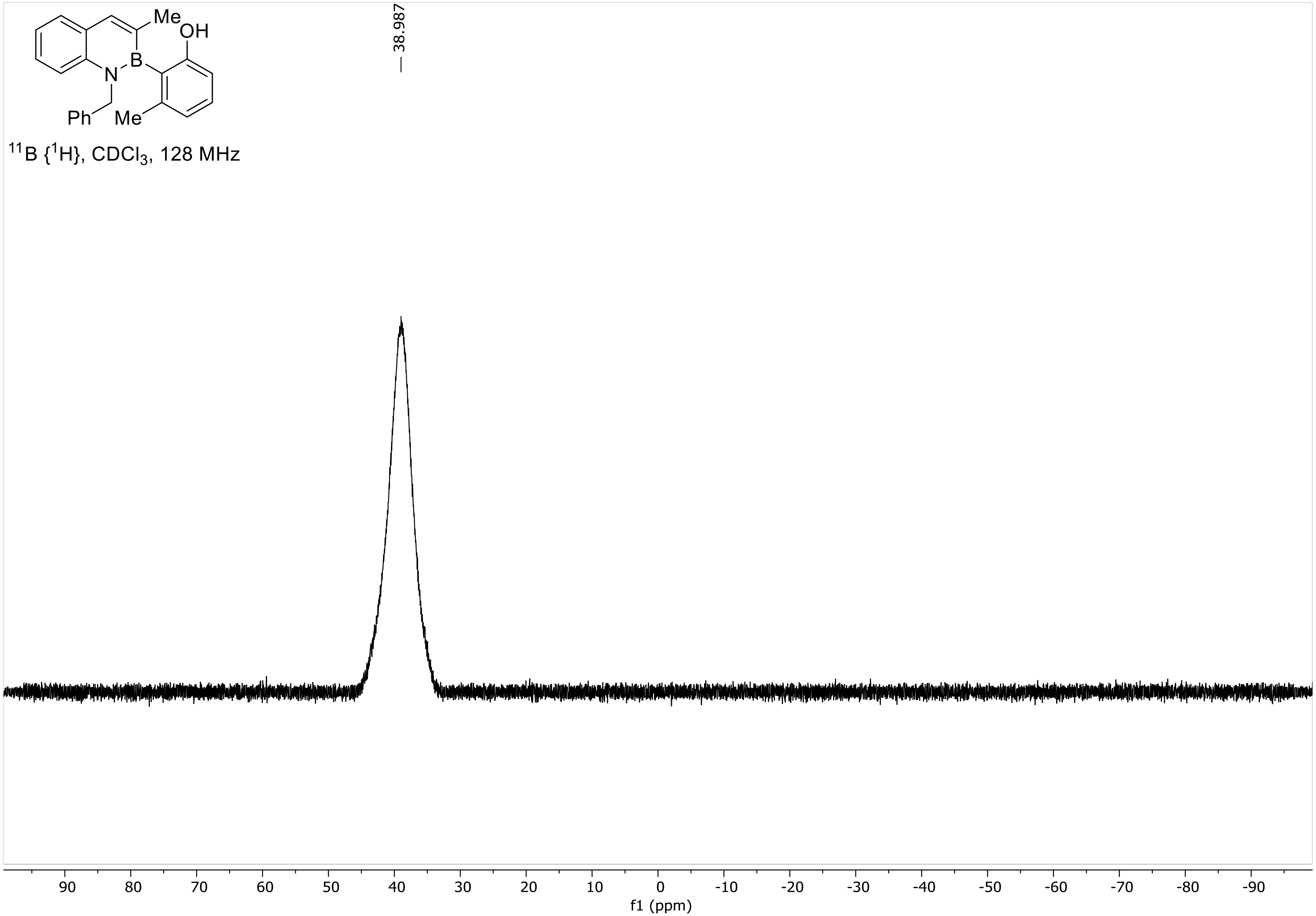

Me
OH
B
N
Ph
Me
$^{11}B\{^1H\}$, $CDCl_3$, 128 MHz
38.987
90
80
70
60
50
40
30
20
10
0
-10
-20
-30
-40
-50
-60
-70
-80
-90
f1 (ppm)

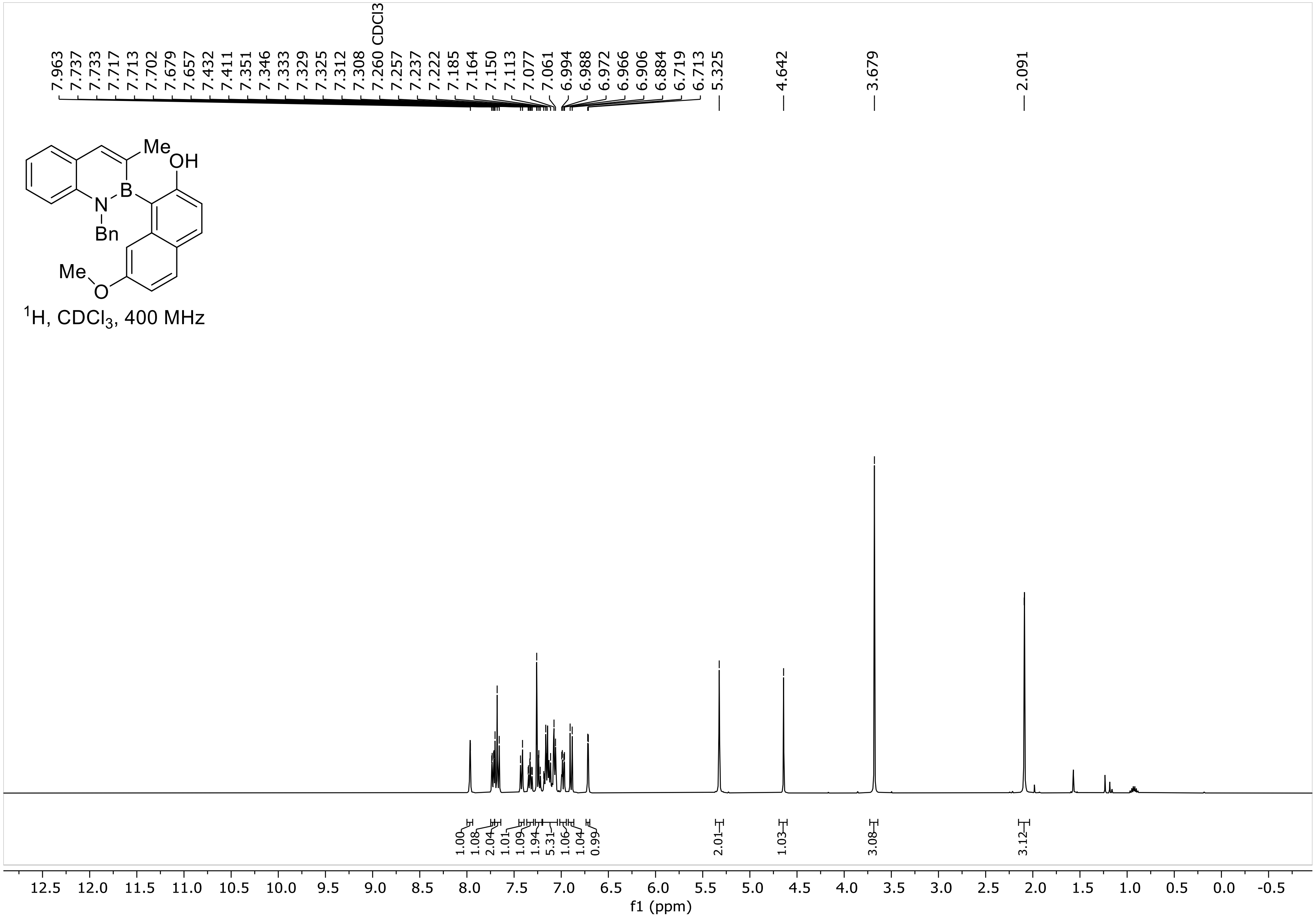
7.963 7.737 7.733 7.717 7.713 7.702 7.679 7.657 7.432 7.411 7.351 7.346 7.333 7.329 7.325 7.312 7.308 7.260 CDCl3 7.257 7.237 7.222 7.185 7.164 7.150 7.113 7.077 7.061 6.994 6.988 6.972 6.966 6.906 6.884 6.719 6.713 5.325 4.642 3.679 2.091
Me
OH
B
N
Bn
Me
O
^{1}H, $CDCl_3$, 400 MHz
1.00 1.08 2.04 1.01 1.09 1.94 5.31 1.06 1.04 0.99 2.01 1.03 3.08 3.12
12.5 12.0 11.5 11.0 10.5 10.0 9.5 9.0 8.5 8.0 7.5 7.0 6.5 6.0 5.5 5.0 4.5 4.0 3.5 3.0 2.5 2.0 1.5 1.0 0.5 0.0 -0.5
f1 (ppm)

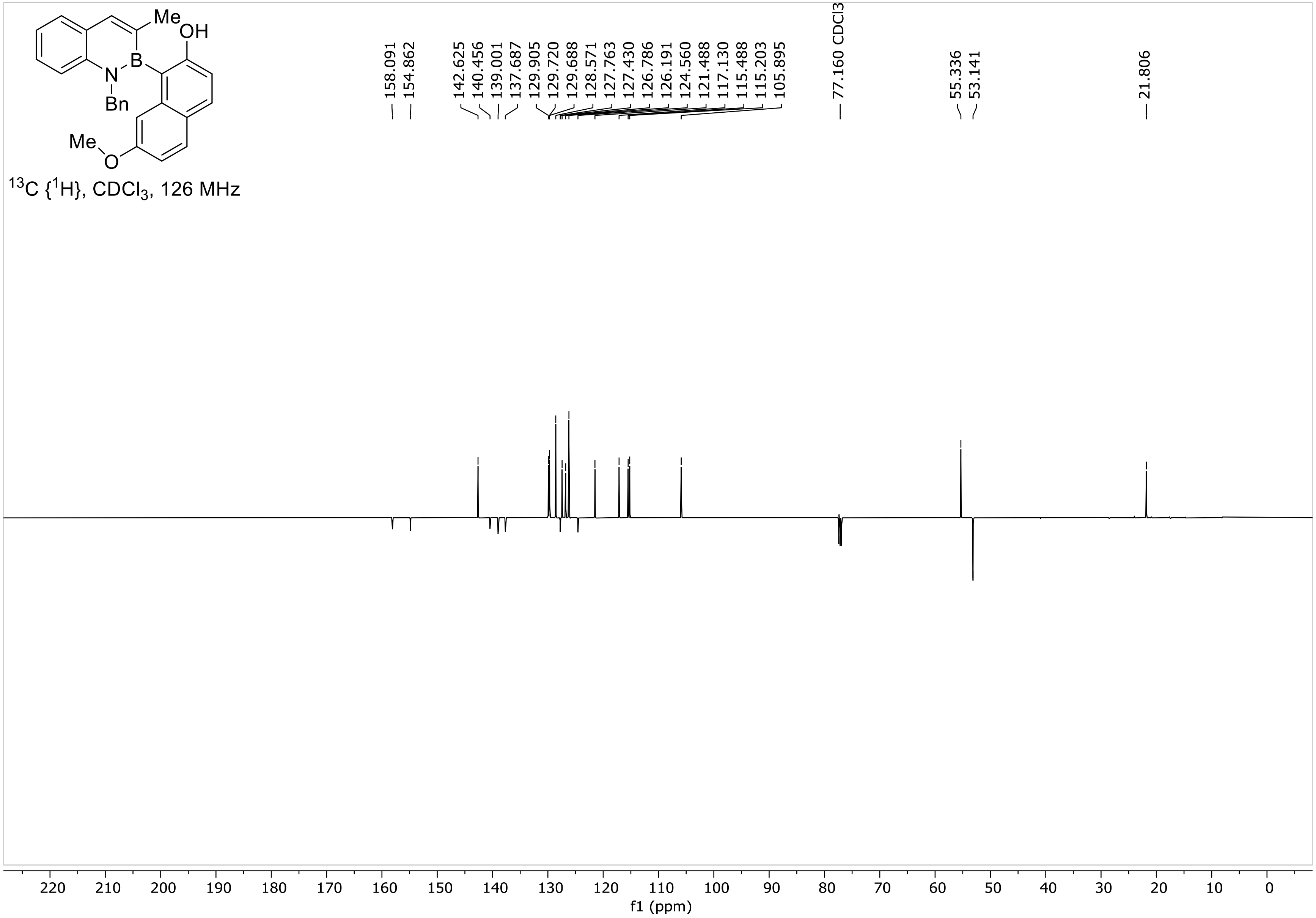
Me
OH
B
N
Bn
Me
O
^{13}C {^1H}, $CDCl_3$, 126 MHz
158.091
154.862
142.625
140.456
139.001
137.687
129.905
129.720
129.688
128.571
127.763
127.430
126.786
126.191
124.560
121.488
117.130
115.488
115.203
105.895
77.160 CDCl3
55.336
53.141
21.806
220 210 200 190 180 170 160 150 140 130 120 110 100 90 80 70 60 50 40 30 20 10 0
f1 (ppm)

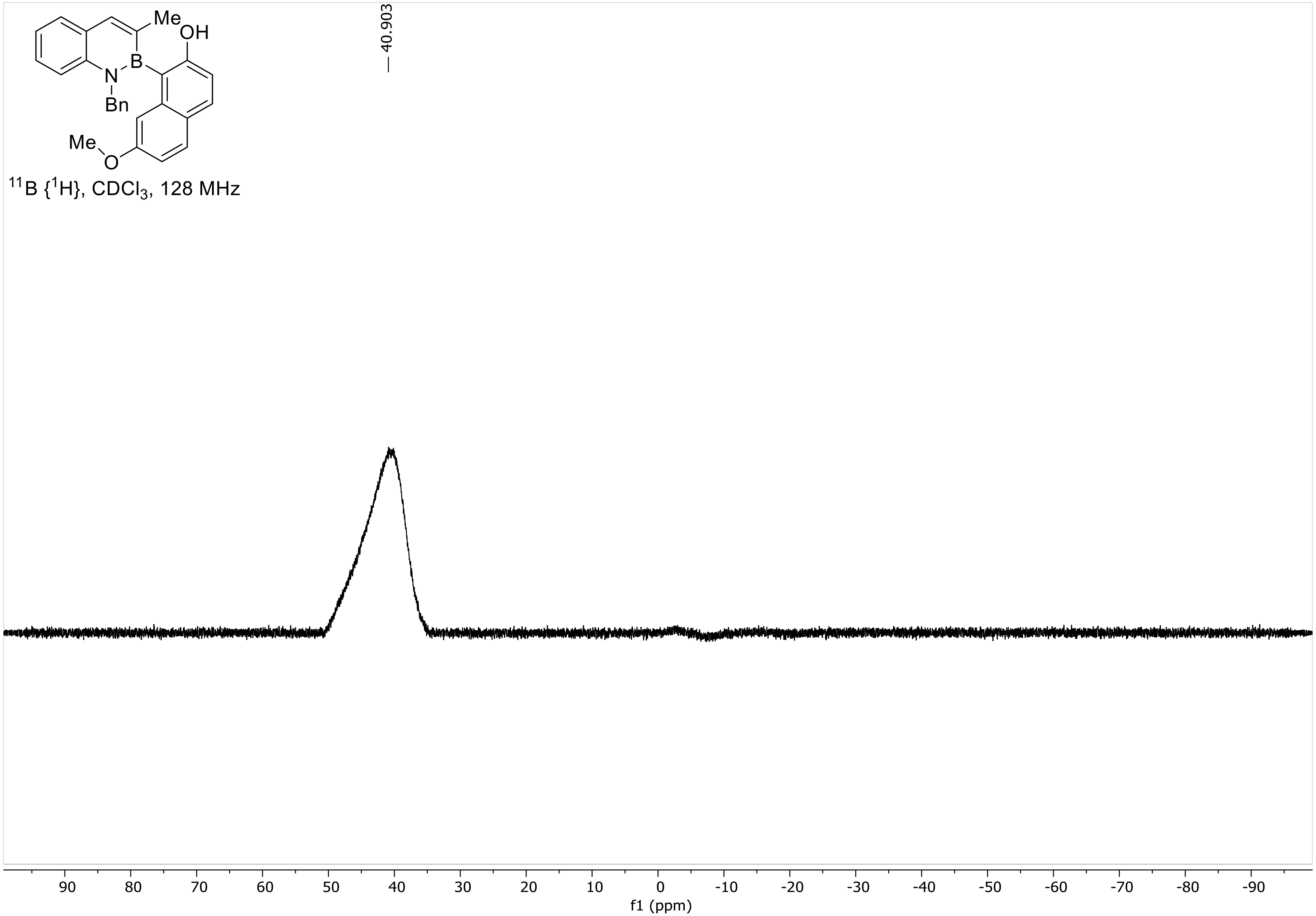
Me
OH
B
N
Bn
Me
O
11B {1H}, CDCl3, 128 MHz
40.903
90 80 70 60 50 40 30 20 10 0 -10 -20 -30 -40 -50 -60 -70 -80 -90
f1 (ppm)

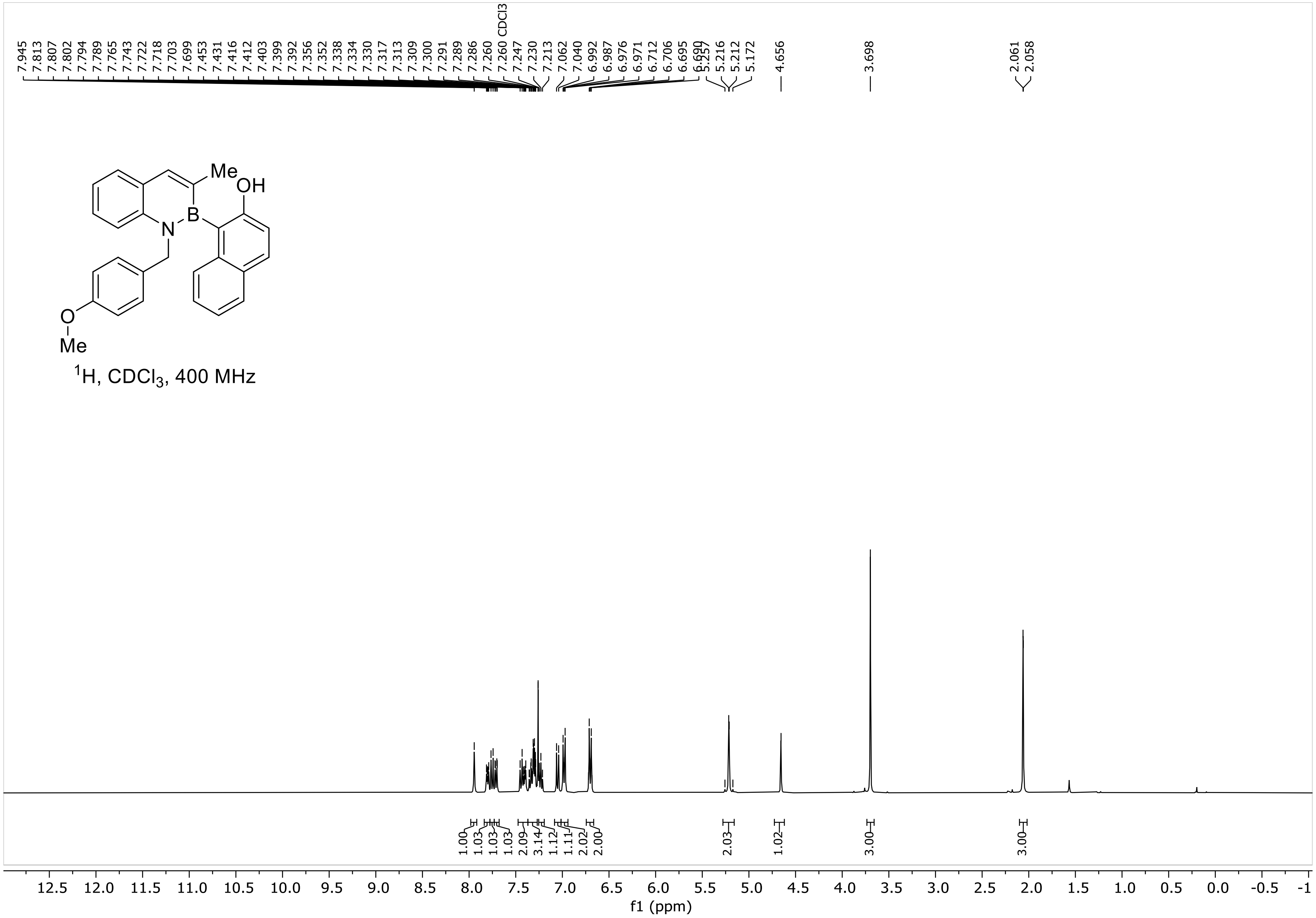
Me
OH
B
N
O
Me
1H, CDCl3, 400 MHz
CDCl3
f1 (ppm)

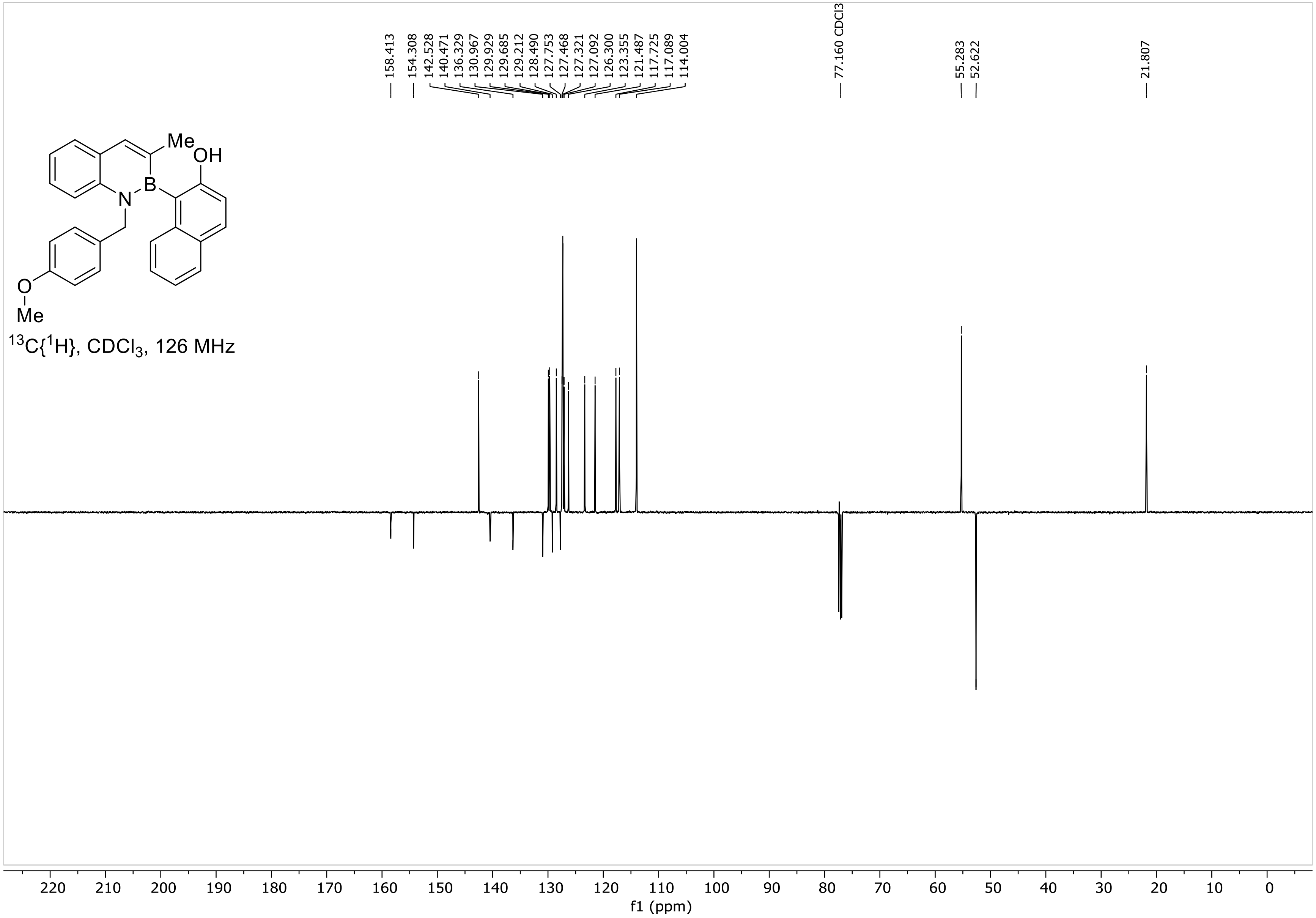
158.413
154.308
142.528
140.471
136.329
130.967
129.929
129.685
129.212
128.490
127.753
127.468
127.321
127.092
126.300
123.355
121.487
117.725
117.089
114.004
77.160 CDCl3
55.283
52.622
21.807
Me
OH
B
N
O
Me
$^{13}C\{^{1}H\}$, $CDCl_3$, 126 MHz
220 210 200 190 180 170 160 150 140 130 120 110 100 90 80 70 60 50 40 30 20 10 0
f1 (ppm)

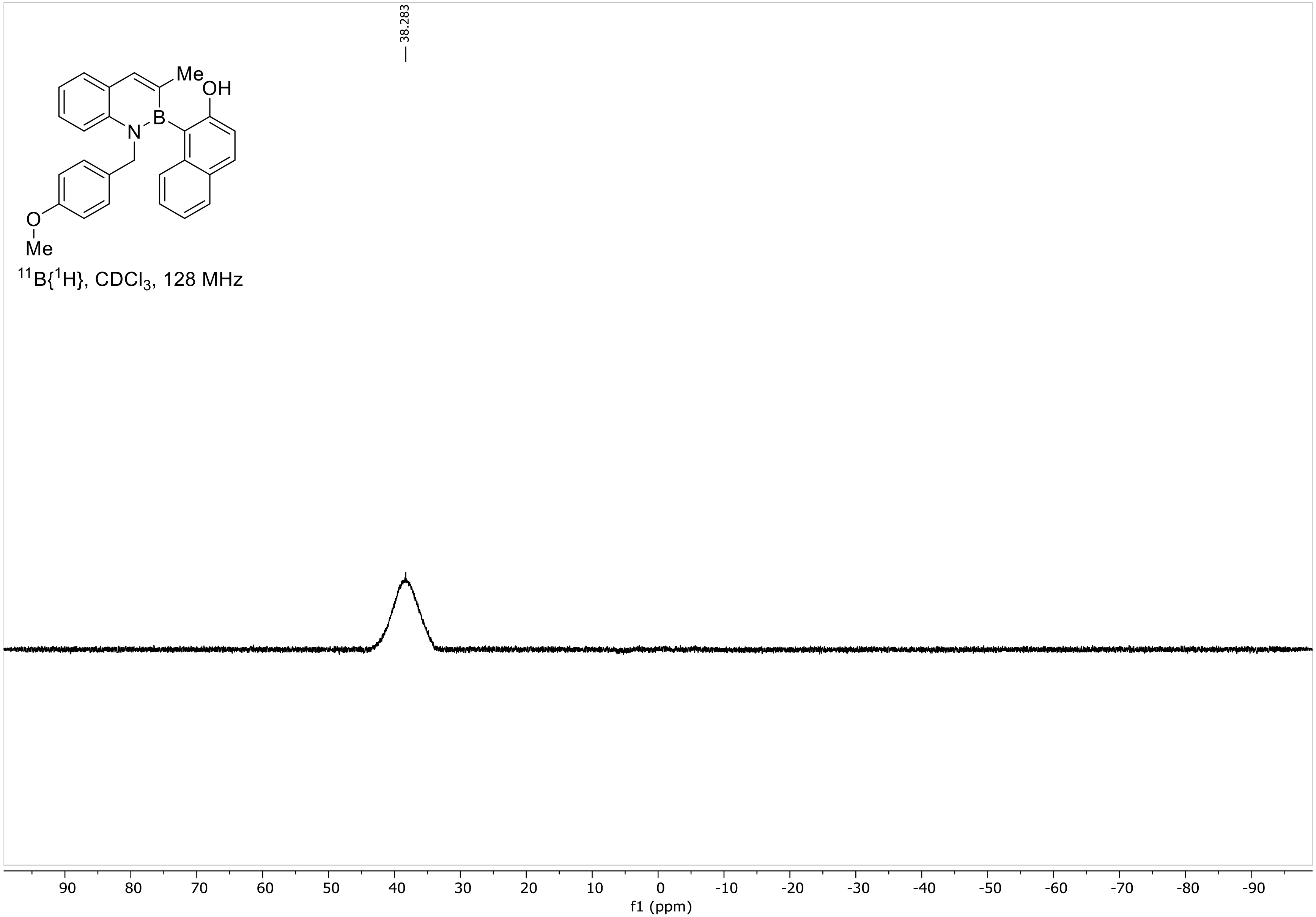

38.283
Me
OH
N
B
O
Me
11B{1H}, CDCl3, 128 MHz
90 80 70 60 50 40 30 20 10 0 -10 -20 -30 -40 -50 -60 -70 -80 -90
f1 (ppm)

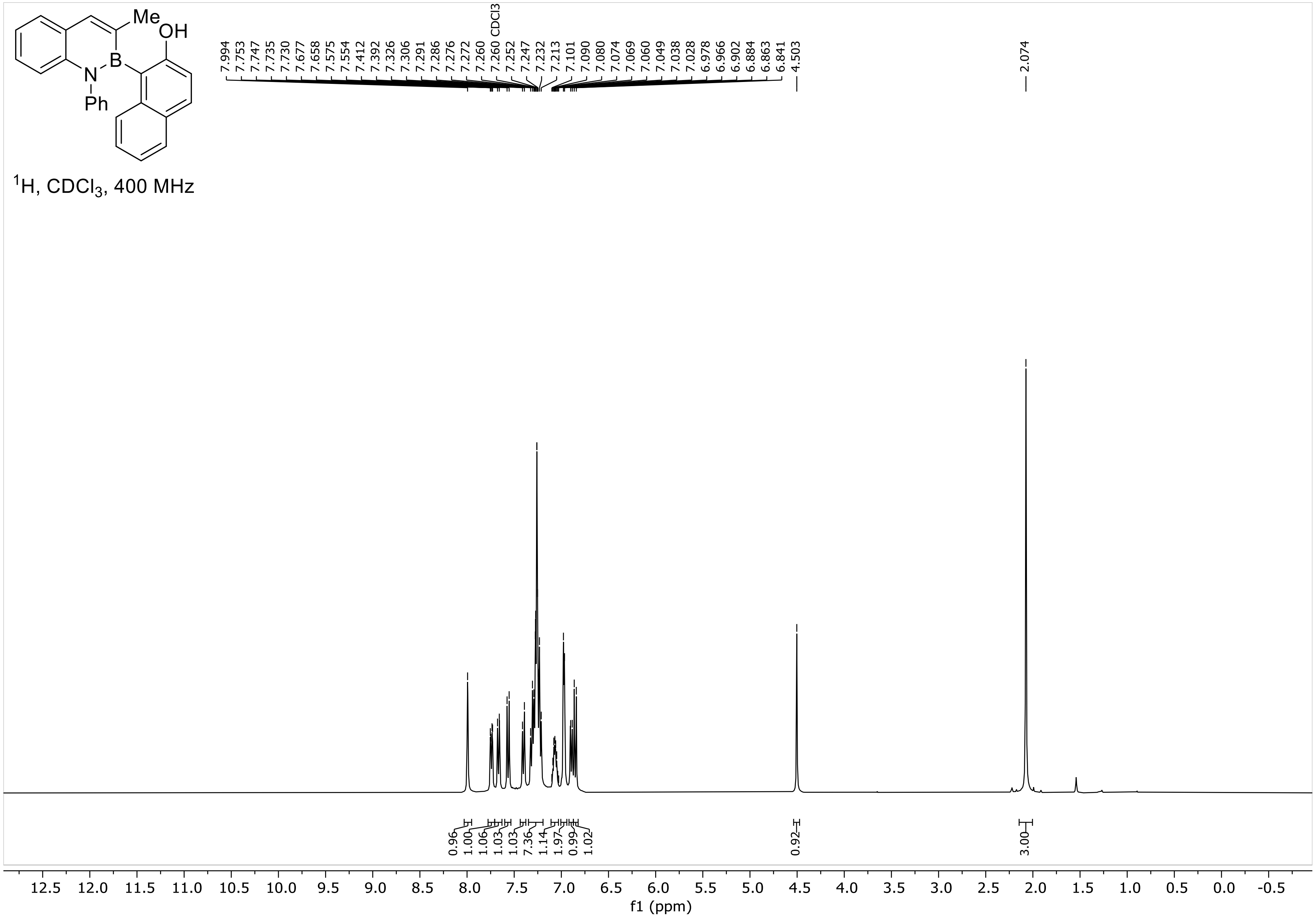
Me
OH
B
N
Ph
^1H, $CDCl_3$, 400 MHz
7.994 7.753 7.747 7.735 7.730 7.677 7.658 7.575 7.554 7.412 7.392 7.326 7.306 7.291 7.286 7.276 7.272 7.260 7.260 CDCl3 7.252 7.247 7.232 7.213 7.101 7.090 7.080 7.074 7.069 7.060 7.049 7.038 7.028 6.978 6.966 6.902 6.884 6.863 6.841
4.503
2.074
0.96 1.00 1.06 1.03 1.03 7.36 1.14 1.97 0.99 1.02
0.92
3.00
f1 (ppm)

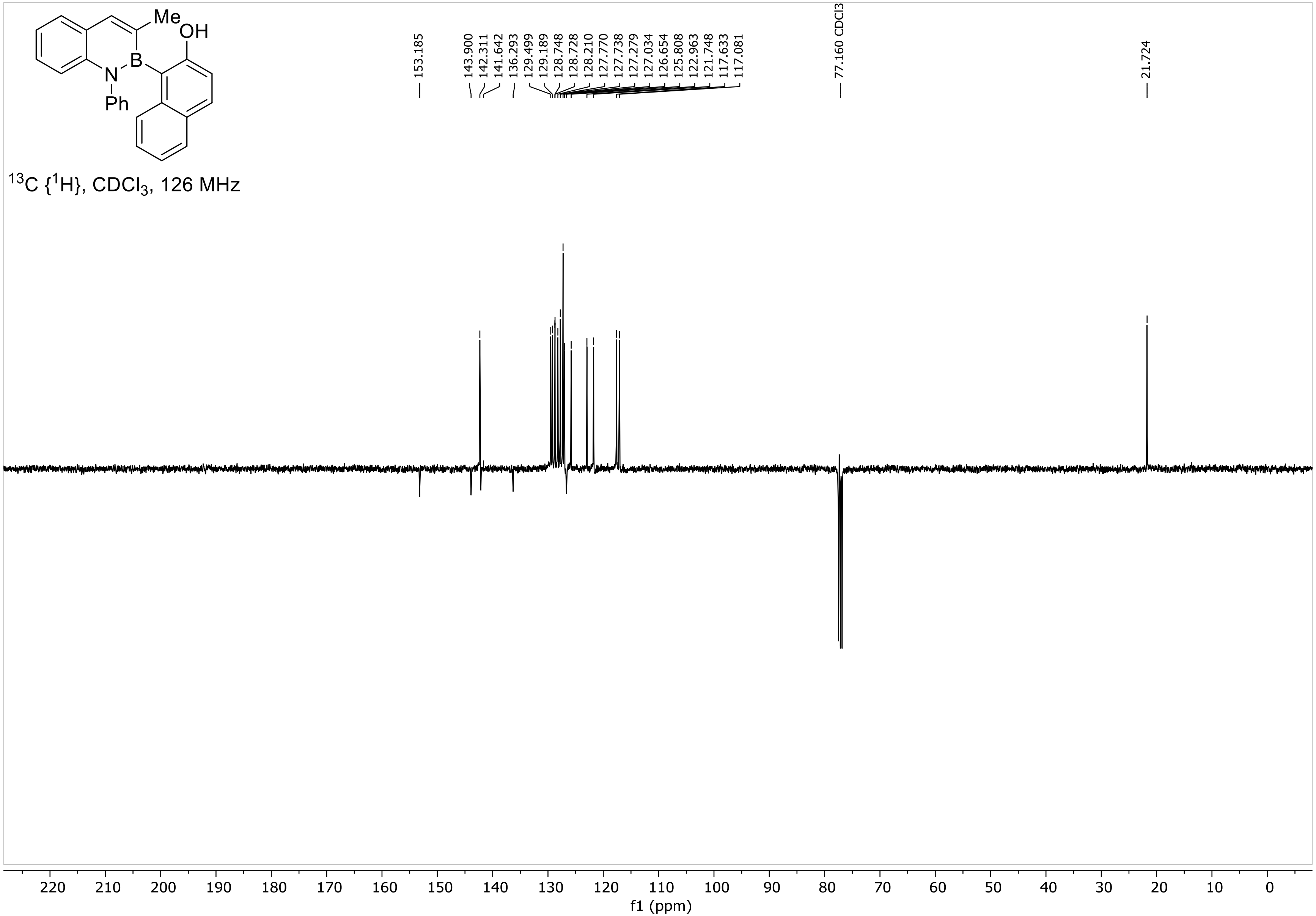
Me
OH
B
N
Ph
13C {1H}, CDCl3, 126 MHz
153.185
143.900
142.311
141.642
136.293
129.499
129.189
128.748
128.728
128.210
127.770
127.738
127.279
127.034
126.654
125.808
122.963
121.748
117.633
117.081
77.160 CDCl3
21.724
220 210 200 190 180 170 160 150 140 130 120 110 100 90 80 70 60 50 40 30 20 10 0
f1 (ppm)

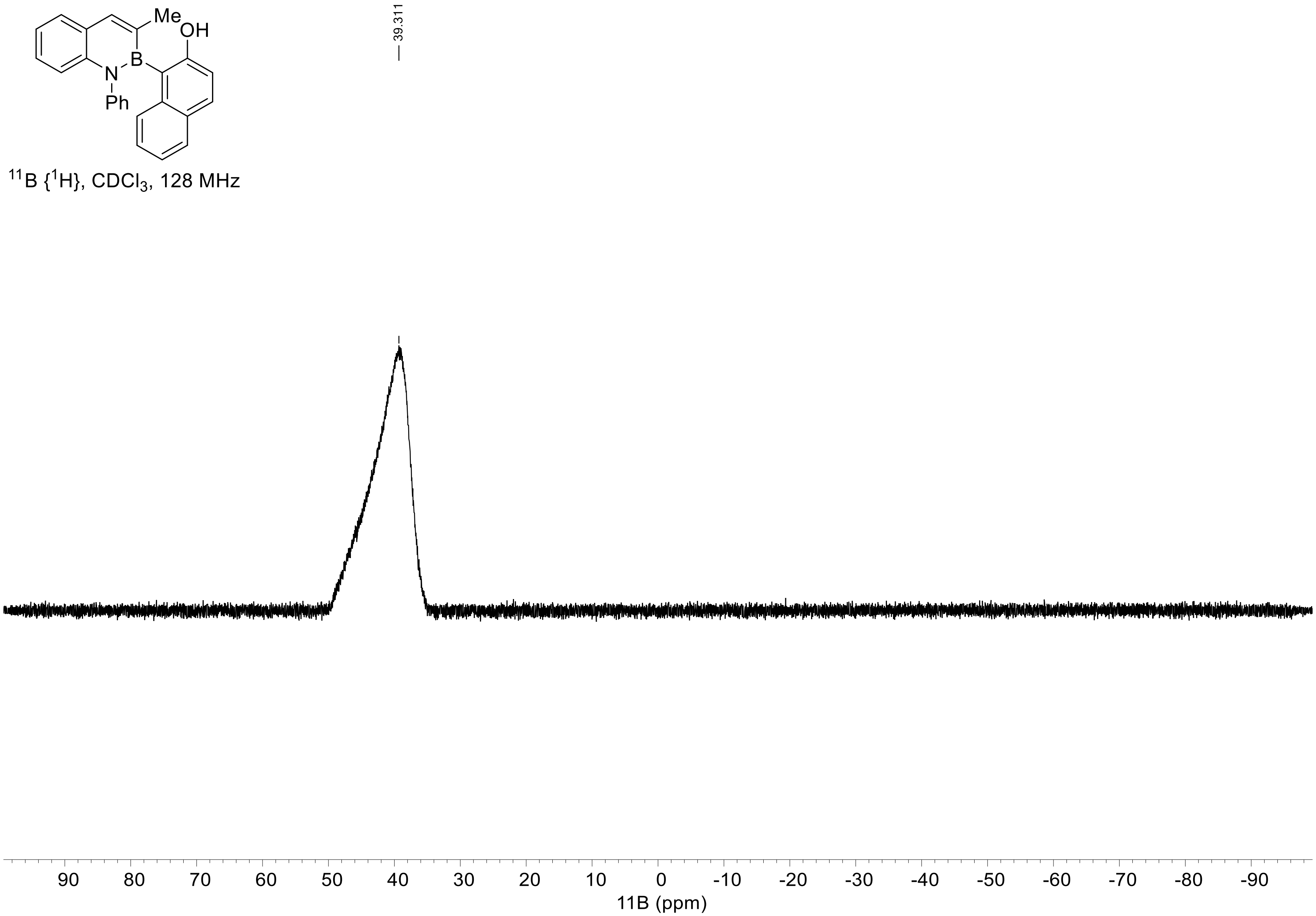

$^{11}B\ \{^{1}H\}$, $CDCl_3$, 128 MHz

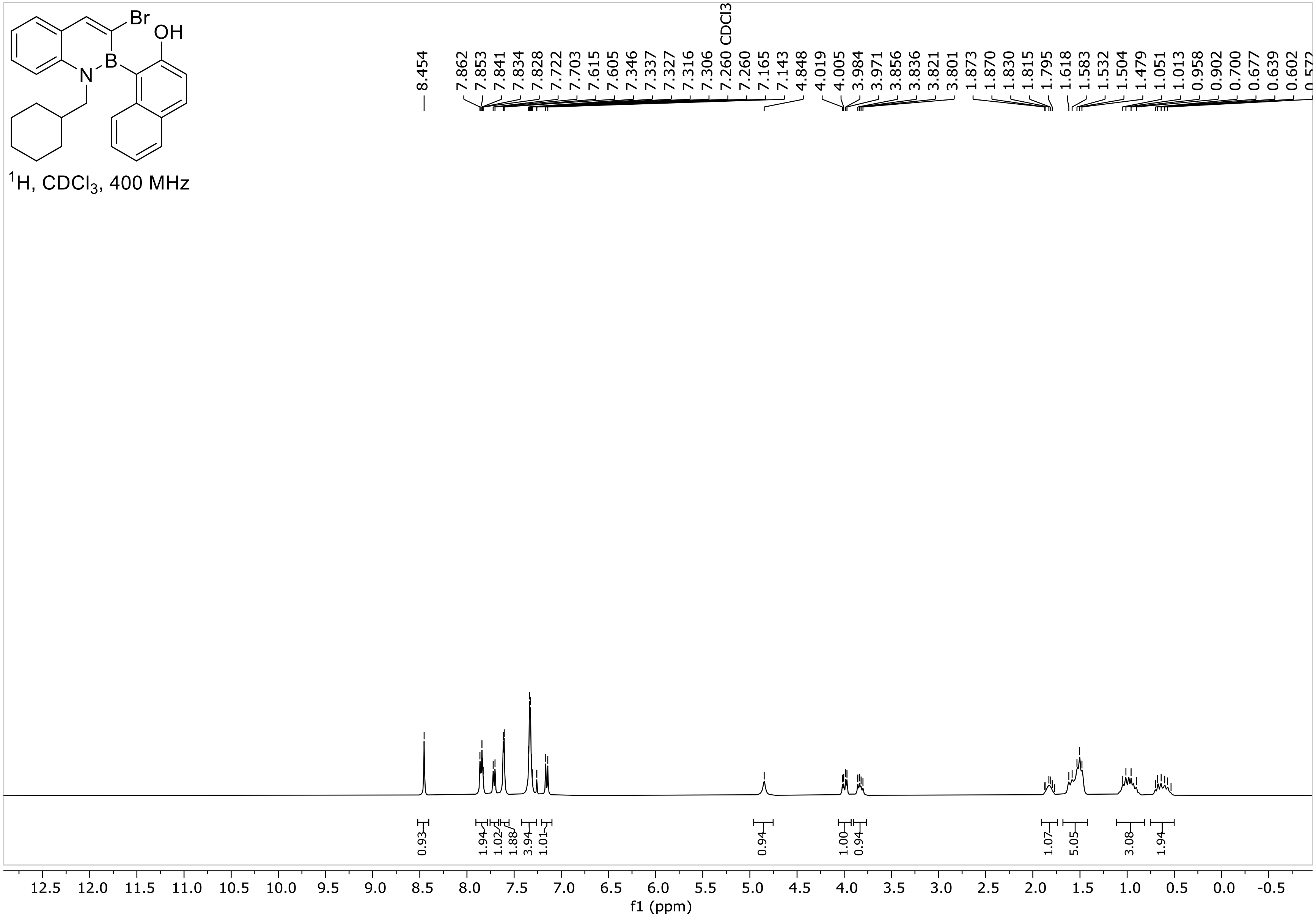

Br
OH
N
B
^{1}H, CDCl$_3$, 400 MHz
8.454
7.862
7.853
7.841
7.834
7.828
7.722
7.703
7.615
7.605
7.346
7.337
7.327
7.316
7.306
7.260 CDCl3
7.260
7.165
7.143
4.848
4.019
4.005
3.984
3.971
3.856
3.836
3.821
3.801
1.873
1.870
1.830
1.815
1.795
1.618
1.583
1.532
1.504
1.479
1.051
1.013
0.958
0.902
0.700
0.677
0.639
0.602
0.93
1.94
1.02
1.88
3.94
1.01
0.94
1.00
0.94
1.07
5.05
3.08
1.94
12.5 12.0 11.5 11.0 10.5 10.0 9.5 9.0 8.5 8.0 7.5 7.0 6.5 6.0 5.5 5.0 4.5 4.0 3.5 3.0 2.5 2.0 1.5 1.0 0.5 0.0 -0.5
f1 (ppm)

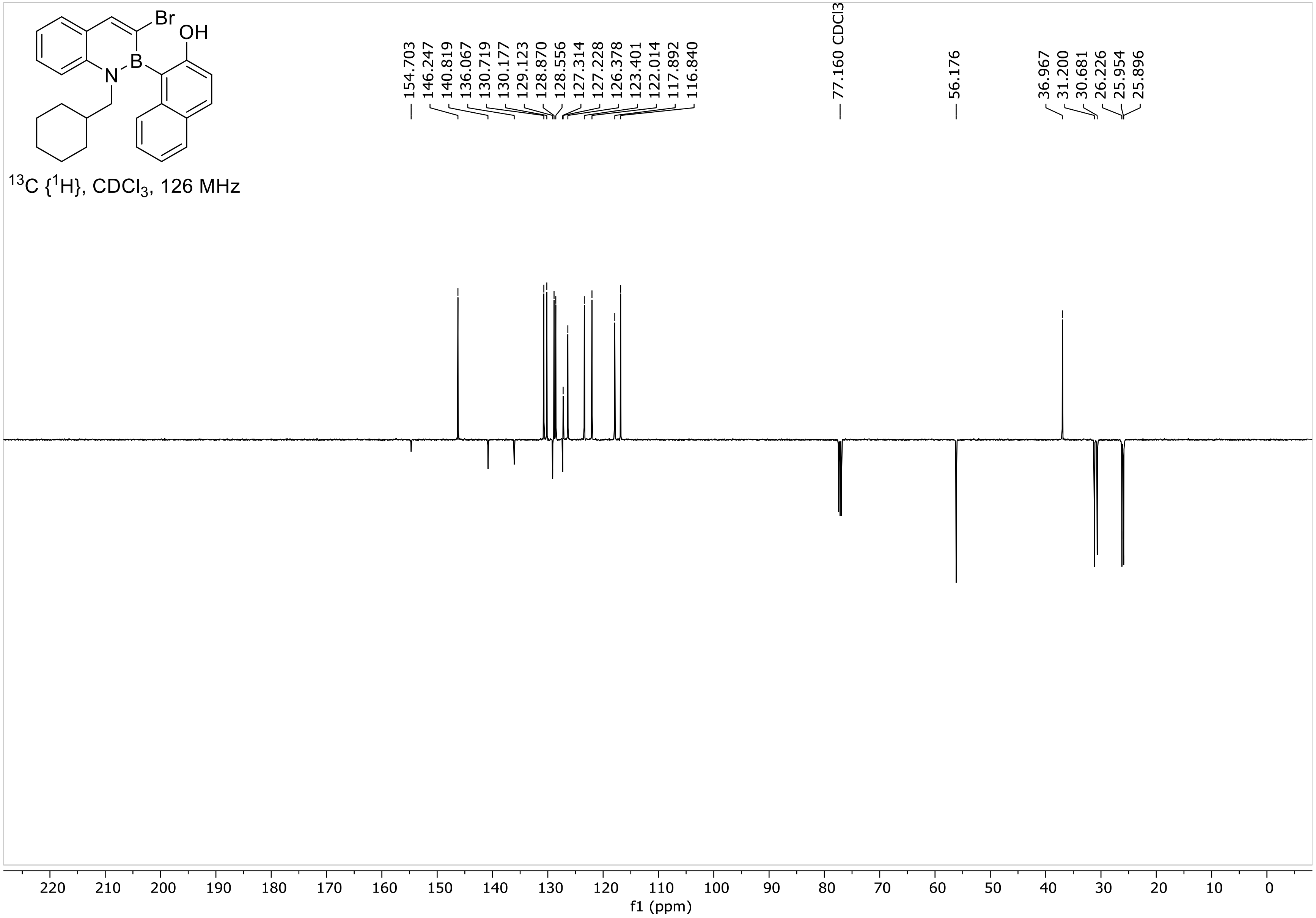
Br
OH
N
B
^{13}C {^{1}H}, CDCl$_3$, 126 MHz
154.703
146.247
140.819
136.067
130.719
130.177
129.123
128.870
128.556
127.314
127.228
126.378
123.401
122.014
117.892
116.840
77.160 CDCl3
56.176
36.967
31.200
30.681
26.226
25.954
25.896
220 210 200 190 180 170 160 150 140 130 120 110 100 90 80 70 60 50 40 30 20 10 0
f1 (ppm)

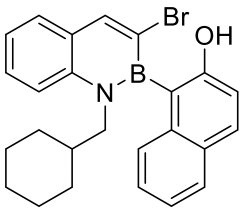

$^{11}B\ \{^{1}H\}$, $CDCl_3$, 128 MHz

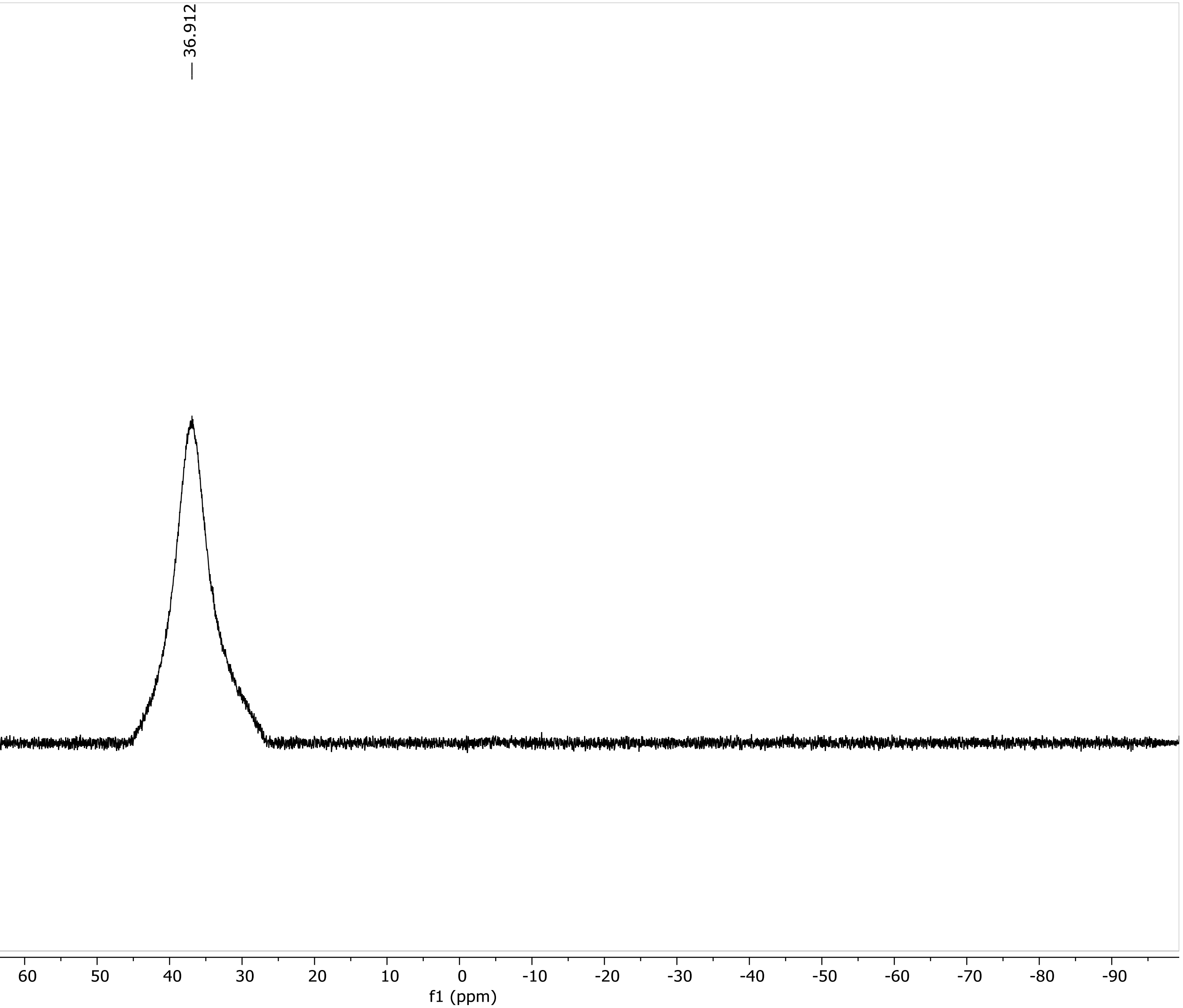

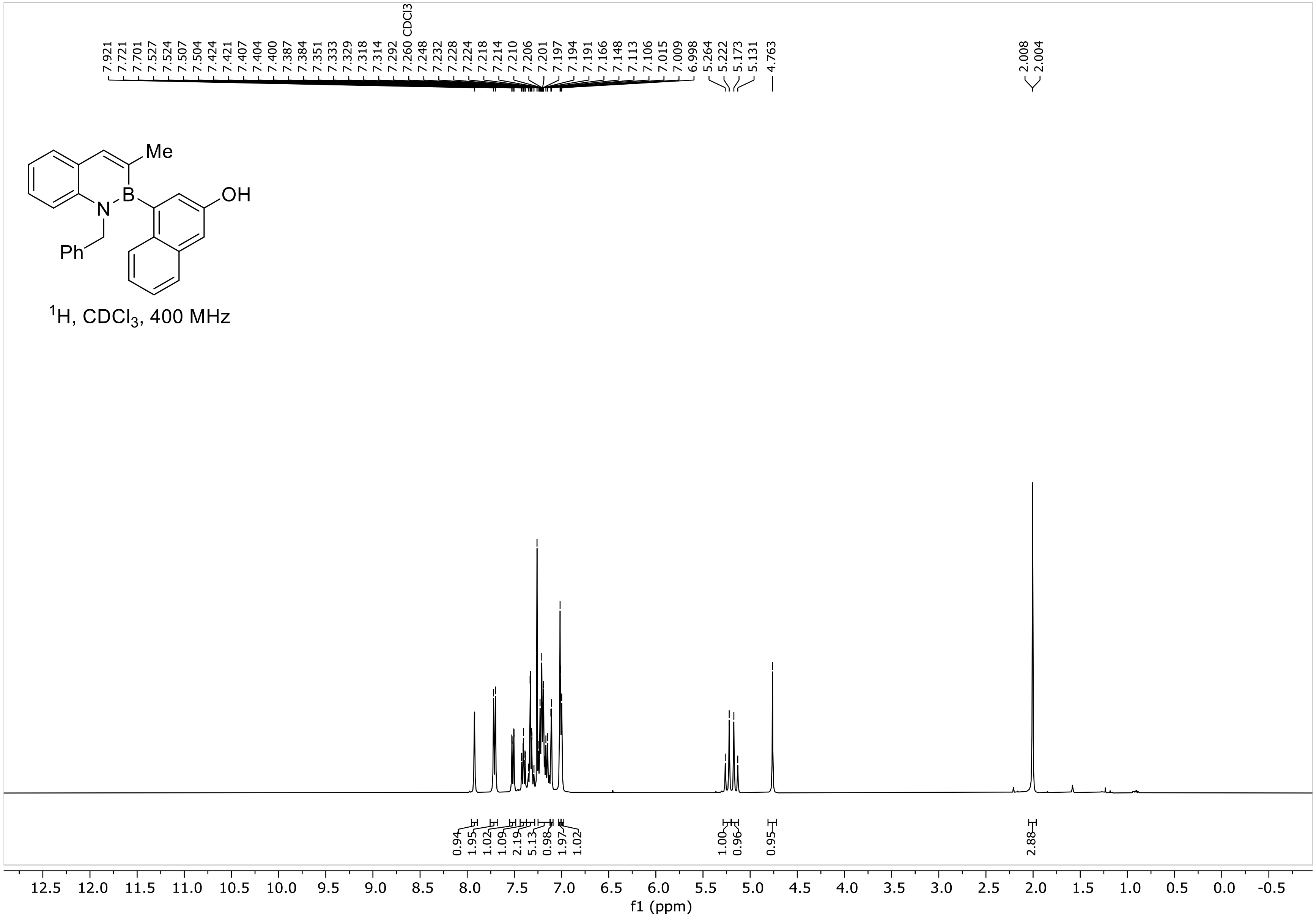
7.921 7.721 7.701 7.527 7.524 7.507 7.504 7.424 7.421 7.407 7.404 7.400 7.387 7.384 7.351 7.333 7.329 7.318 7.314 7.292 7.260 CDCl3 7.248 7.232 7.228 7.224 7.218 7.214 7.210 7.206 7.201 7.197 7.194 7.191 7.166 7.148 7.113 7.106 7.015 7.009 6.998 5.264 5.222 5.173 5.131 4.763
2.008 2.004
Me
B
N
OH
Ph
^{1}H, $CDCl_3$, 400 MHz
0.94 1.95 1.02 1.09 2.19 5.13 0.98 1.97 1.02
1.00 0.96
0.95
2.88
12.5 12.0 11.5 11.0 10.5 10.0 9.5 9.0 8.5 8.0 7.5 7.0 6.5 6.0 5.5 5.0 4.5 4.0 3.5 3.0 2.5 2.0 1.5 1.0 0.5 0.0 -0.5
f1 (ppm)

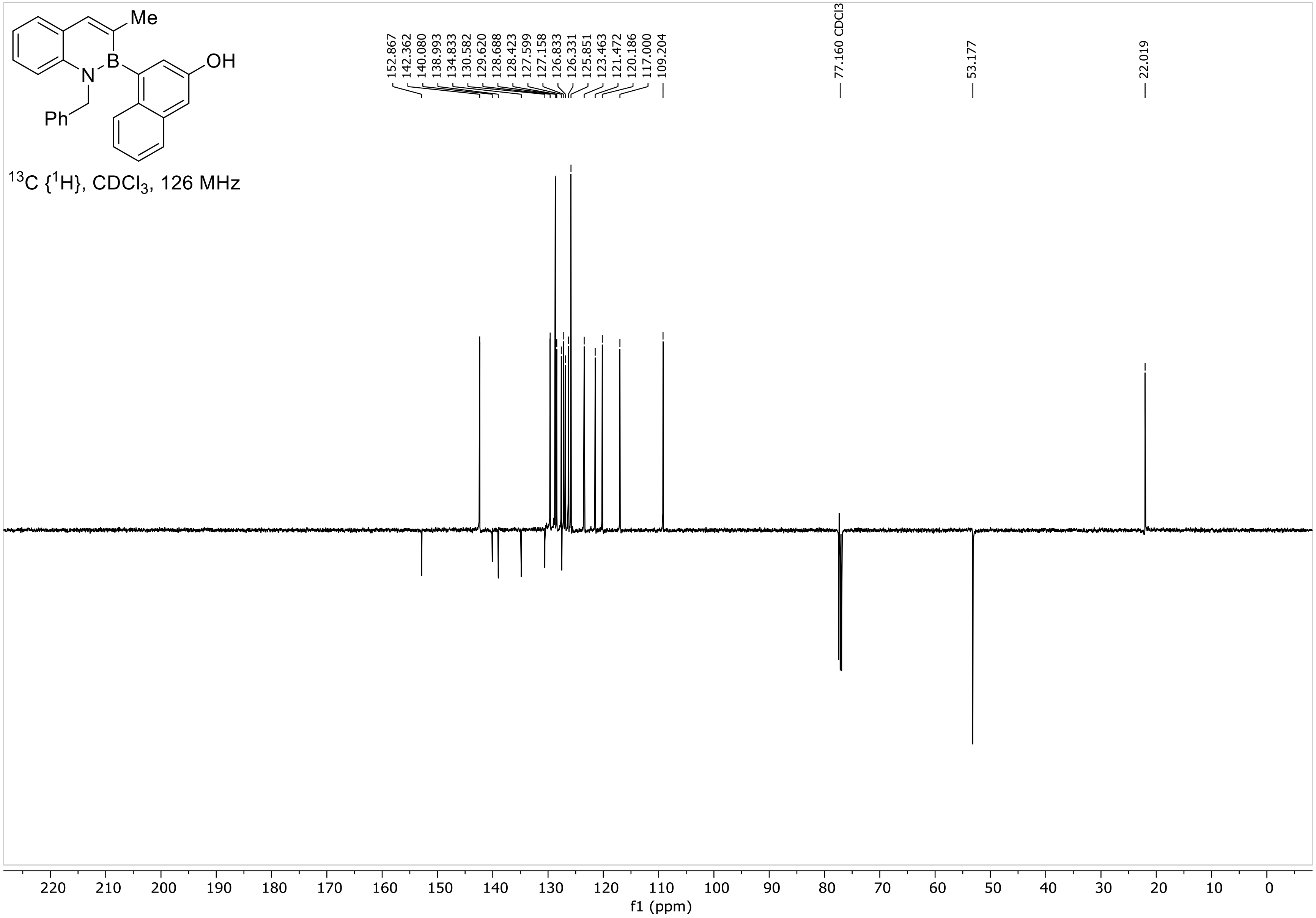
Me
B
OH
N
Ph
13C {1H}, CDCl3, 126 MHz
152.867
142.362
140.080
138.993
134.833
130.582
129.620
128.688
128.423
127.599
127.158
126.833
126.331
125.851
123.463
121.472
120.186
117.000
109.204
77.160 CDCl3
53.177
22.019
220 210 200 190 180 170 160 150 140 130 120 110 100 90 80 70 60 50 40 30 20 10 0
f1 (ppm)

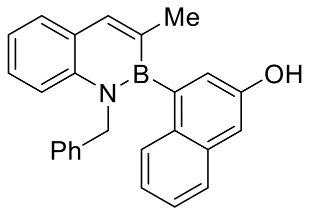

$^{11}B\ \{^1H\}$, $CDCl_3$, 128 MHz

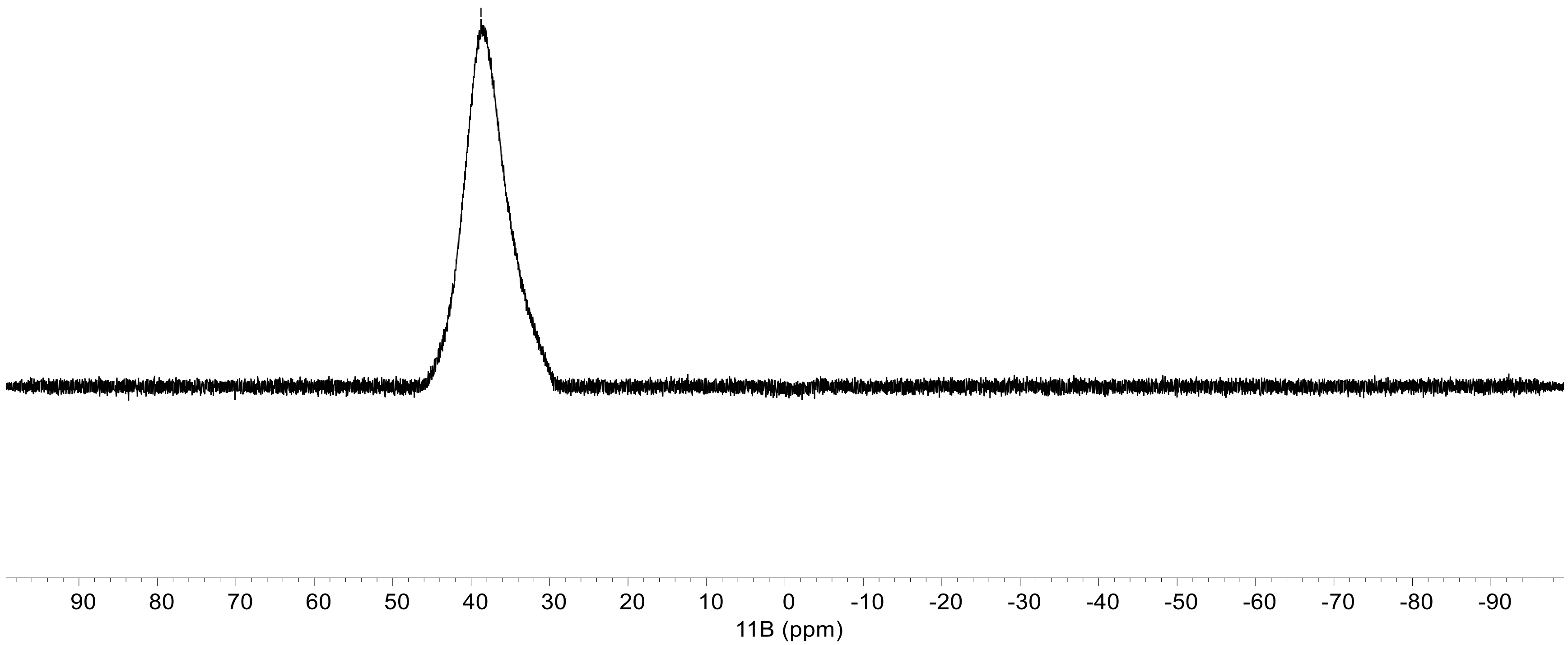

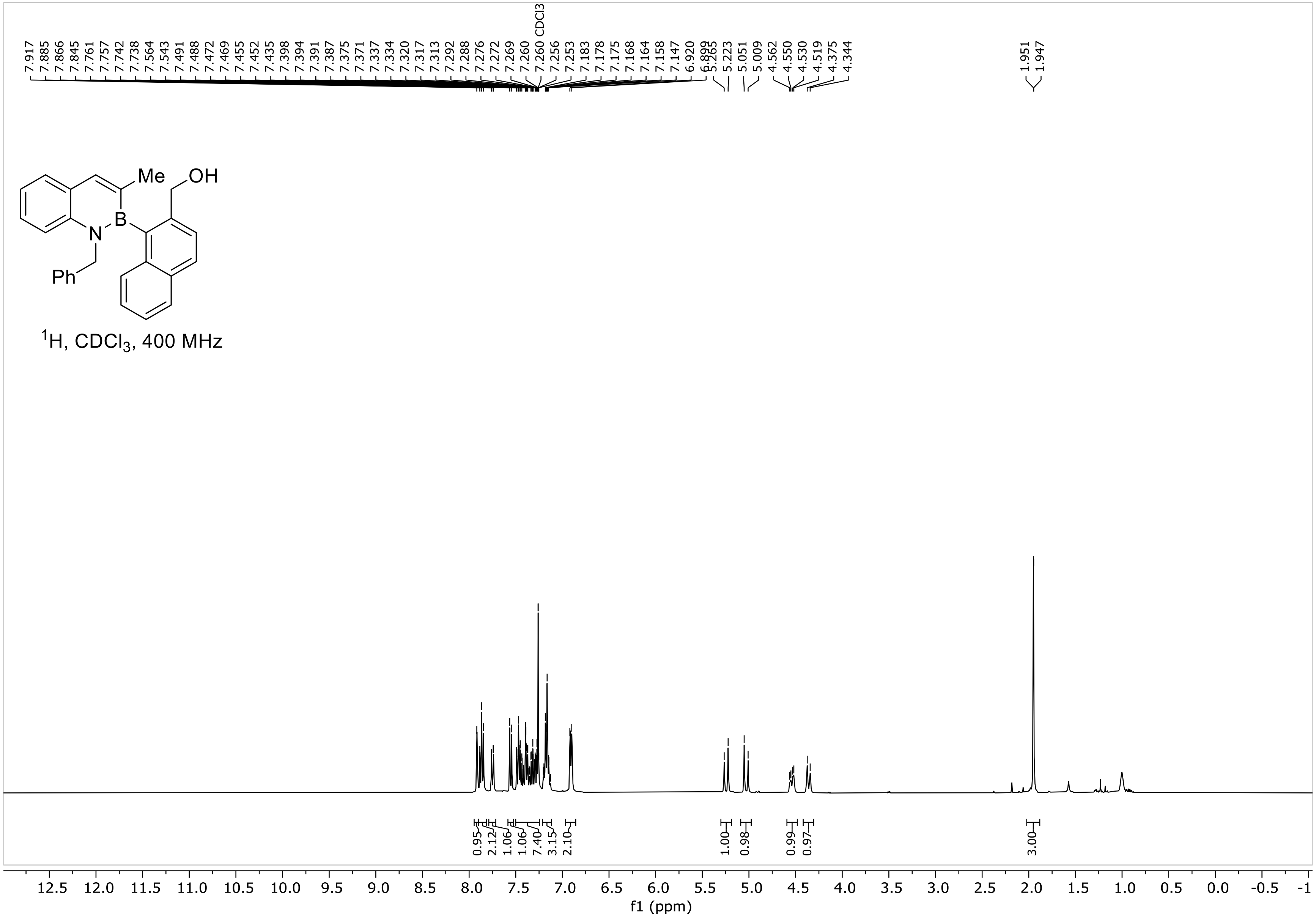

^{1}H, $CDCl_3$, 400 MHz

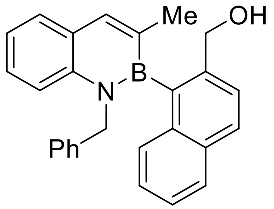

$^{13}C \{^{1}H\}$, $CDCl_3$, 126 MHz

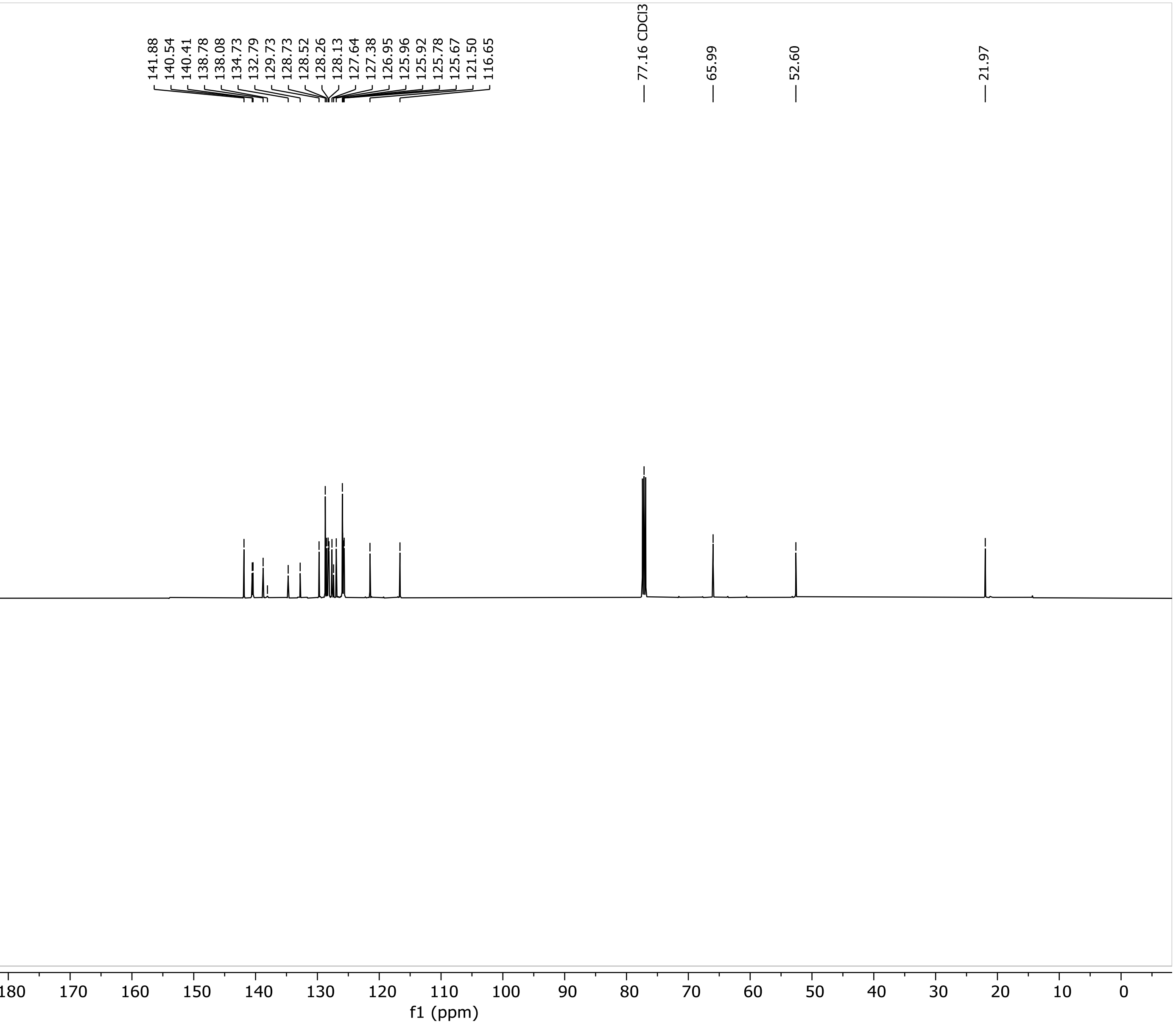

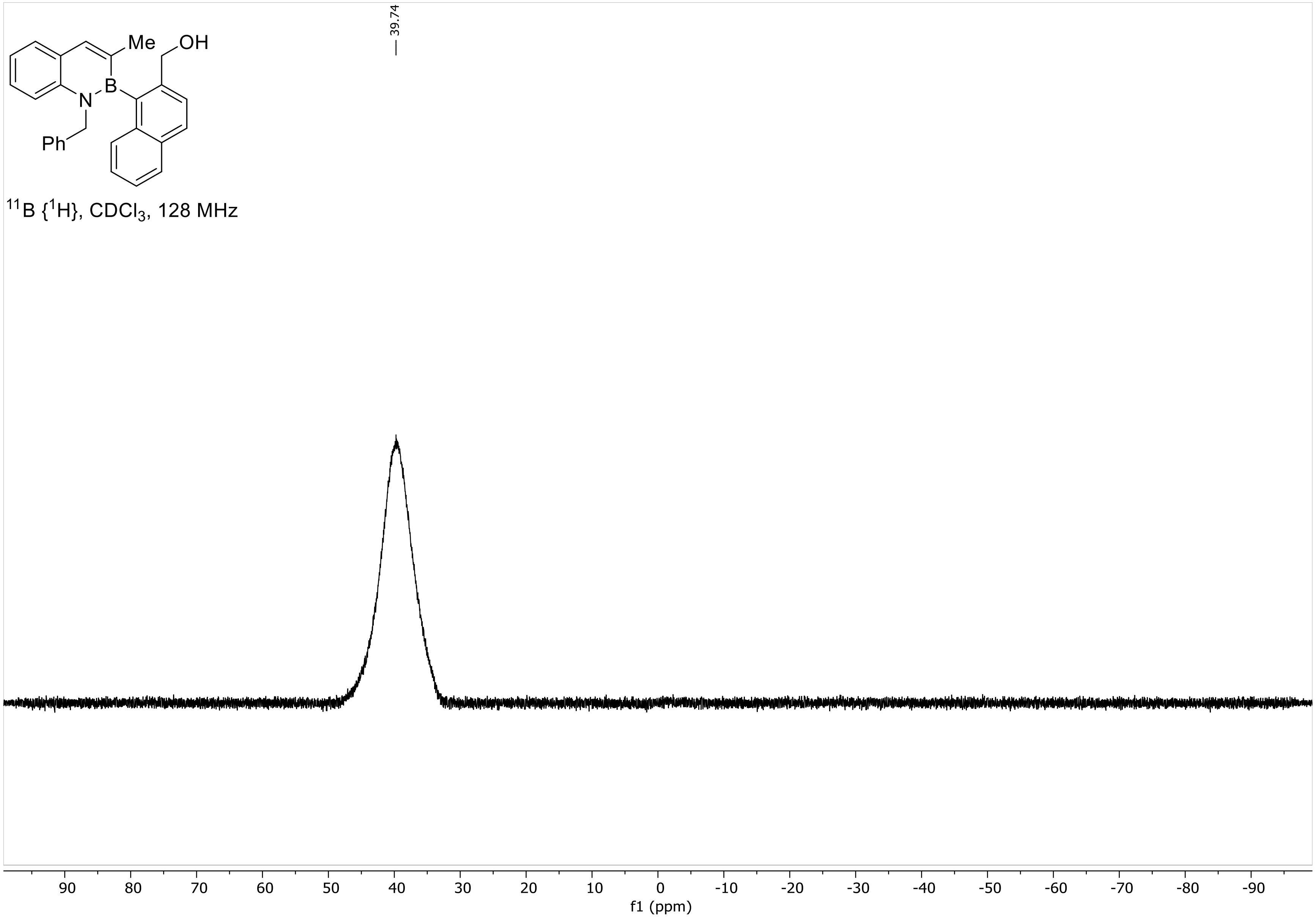

Me
OH
B
N
Ph
^{11}B {^{1}H}, CDCl$_3$, 128 MHz
39.74
90
80
70
60
50
40
30
20
10
0
-10
-20
-30
-40
-50
-60
-70
-80
-90
f1 (ppm)

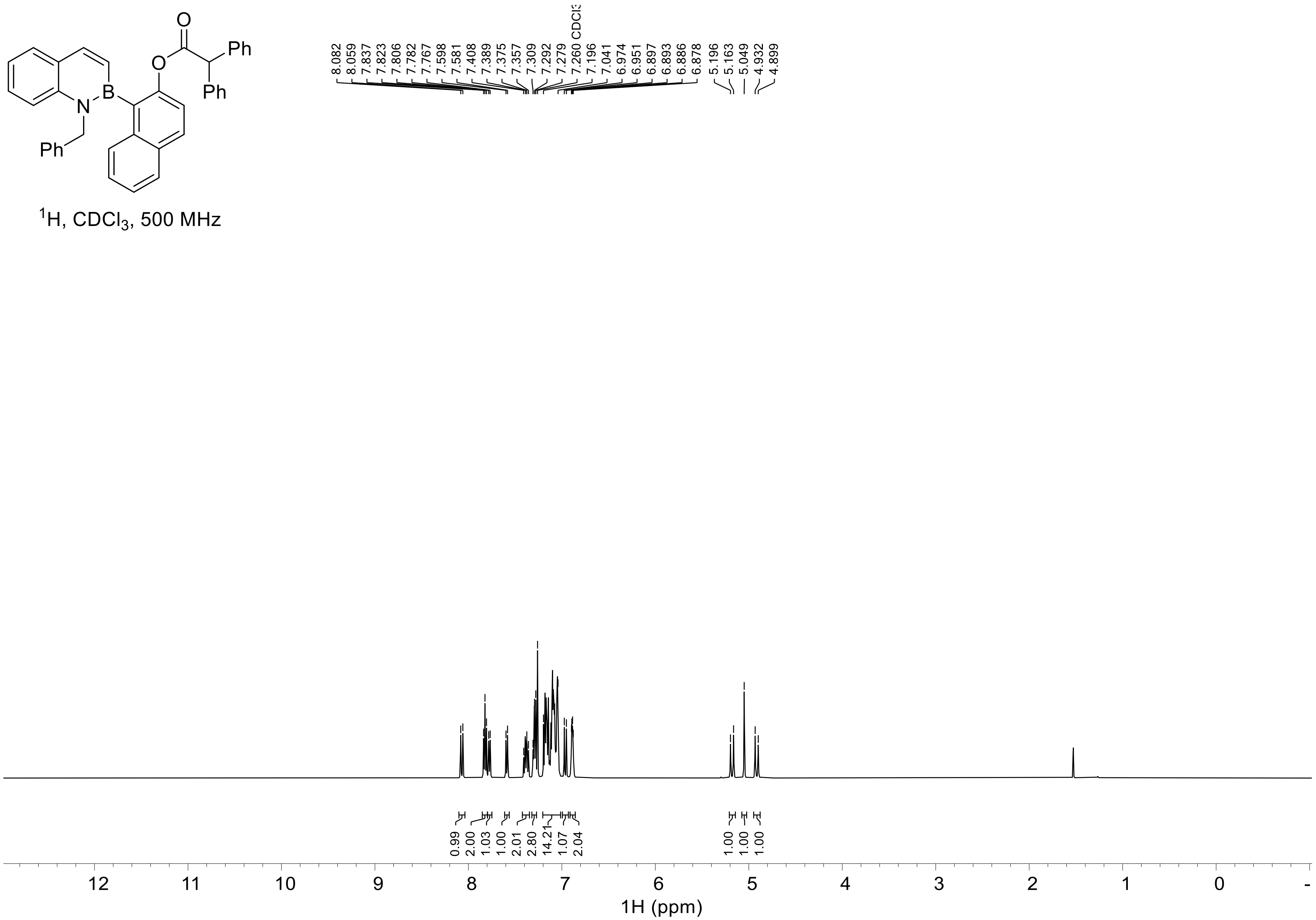
O
Ph
Ph
B
N
Ph
1H, CDCl3, 500 MHz
8.082
8.059
7.837
7.823
7.806
7.782
7.767
7.598
7.581
7.408
7.389
7.375
7.357
7.309
7.292
7.279
7.260 CDCl3
7.196
7.041
6.974
6.951
6.897
6.893
6.886
6.878
5.196
5.163
5.049
4.932
4.899
0.99
2.00
1.03
1.00
2.01
2.80
14.21
1.07
2.04
1.00
1.00
1.00
12
11
10
9
8
7
6
5
4
3
2
1
0
1H (ppm)

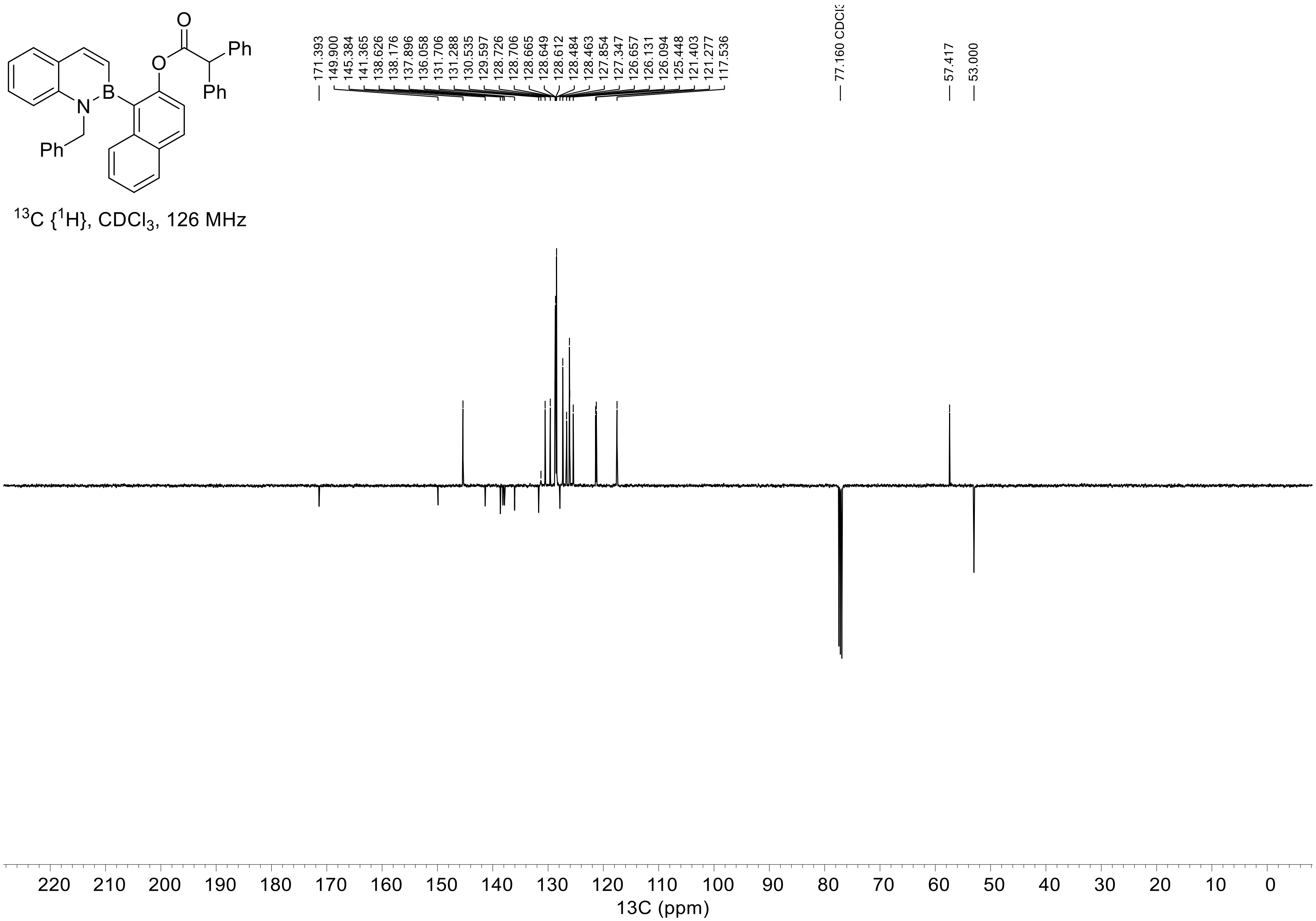
O
O
Ph
Ph
B
N
Ph
13C {1H}, CDCl3, 126 MHz
171.393
149.900
145.384
141.365
138.626
138.176
137.896
136.058
131.706
131.288
130.535
129.597
128.726
128.706
128.665
128.649
128.612
128.484
128.463
127.854
127.347
126.657
126.131
126.094
125.448
121.403
121.277
117.536
77.160 CDCl3
57.417
53.000
220 210 200 190 180 170 160 150 140 130 120 110 100 90 80 70 60 50 40 30 20 10 0
13C (ppm)

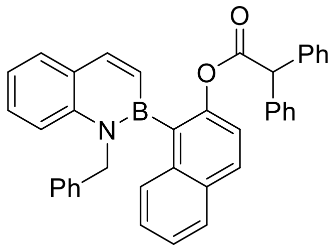

$^{11}B\ \{^{1}H\}$, $CDCl_3$, 128 MHz

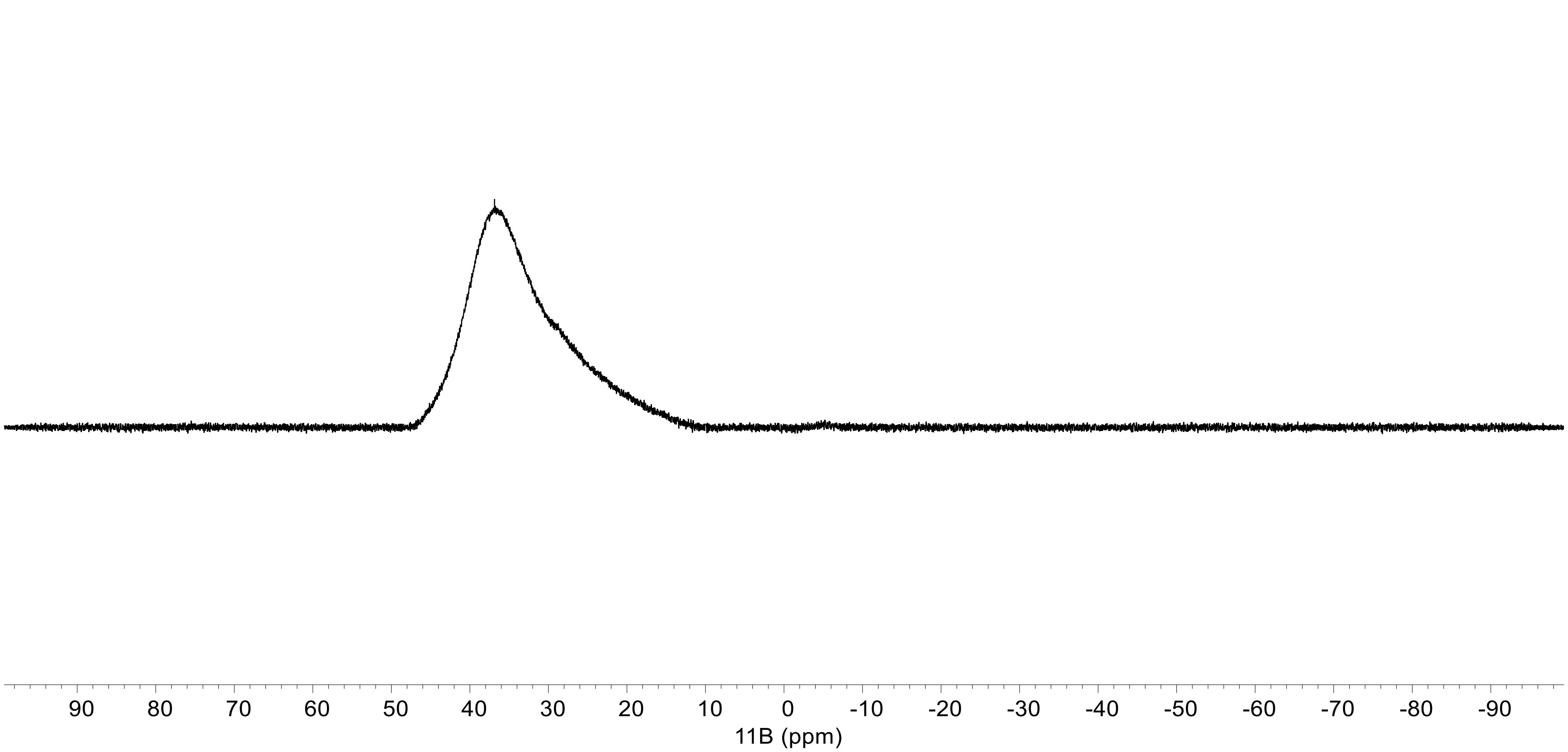

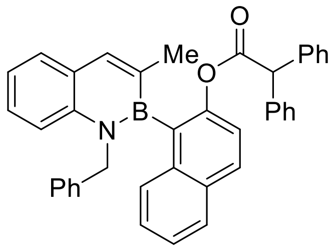

^1H, $CDCl_3$, 400 MHz

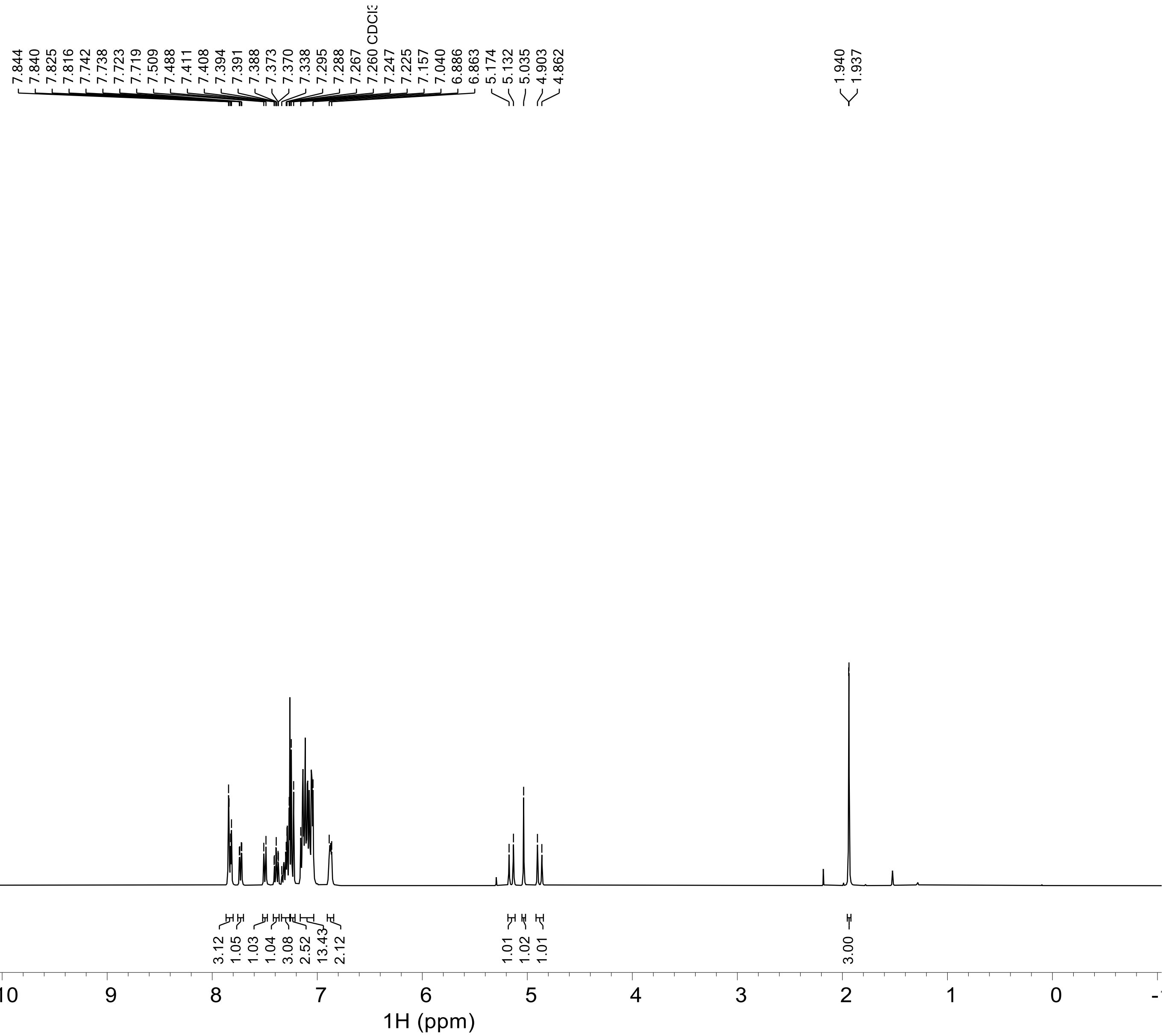

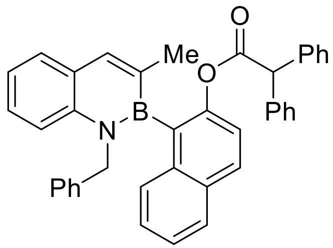

$^{13}C\ \{^{1}H\}$, $CDCl_3$, 101 MHz

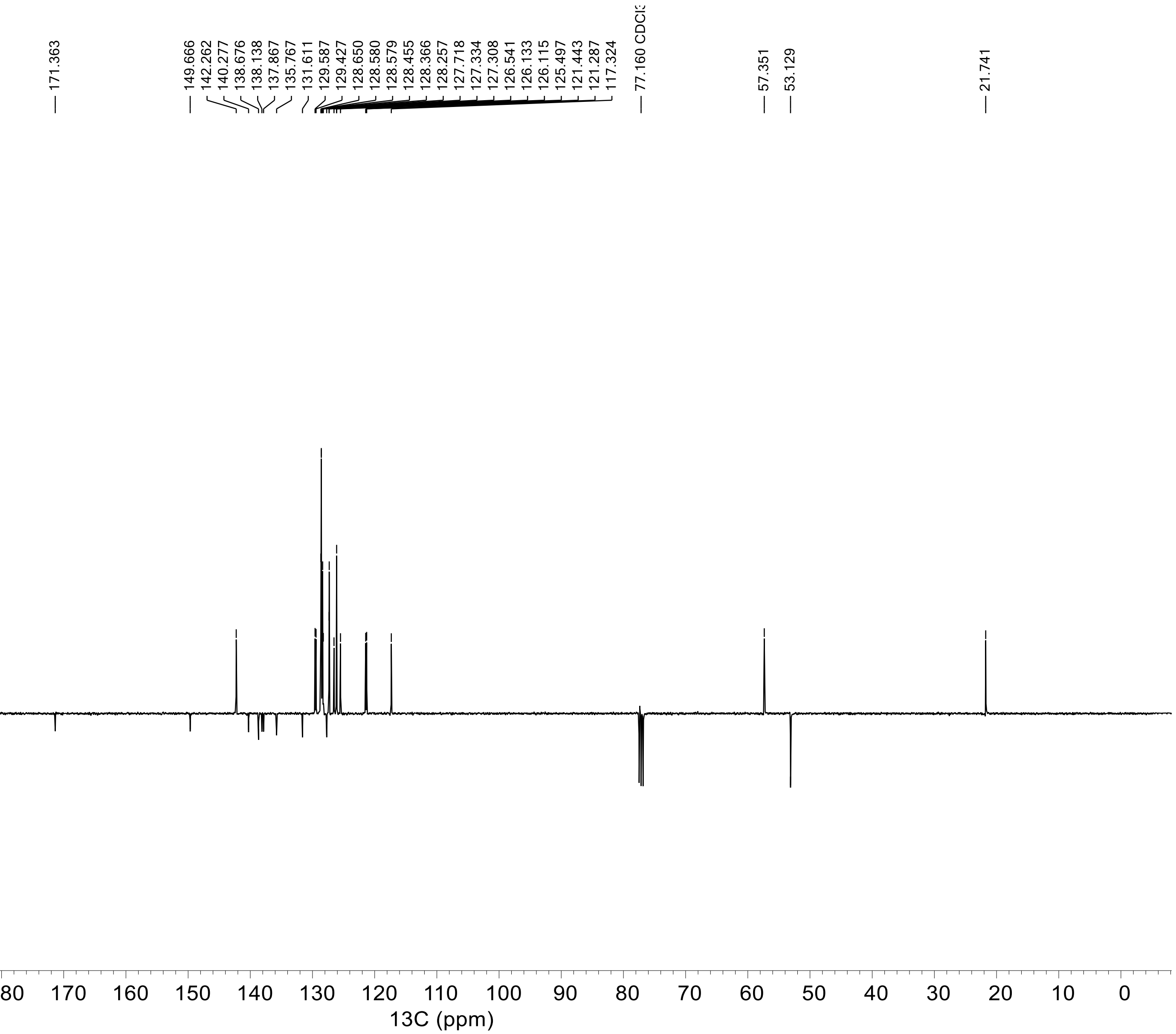

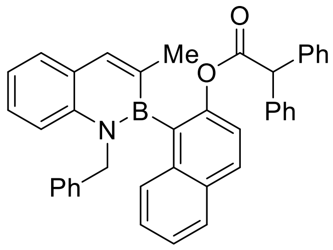

$^{11}B\ \{^{1}H\}$, $CDCl_3$, 128 MHz

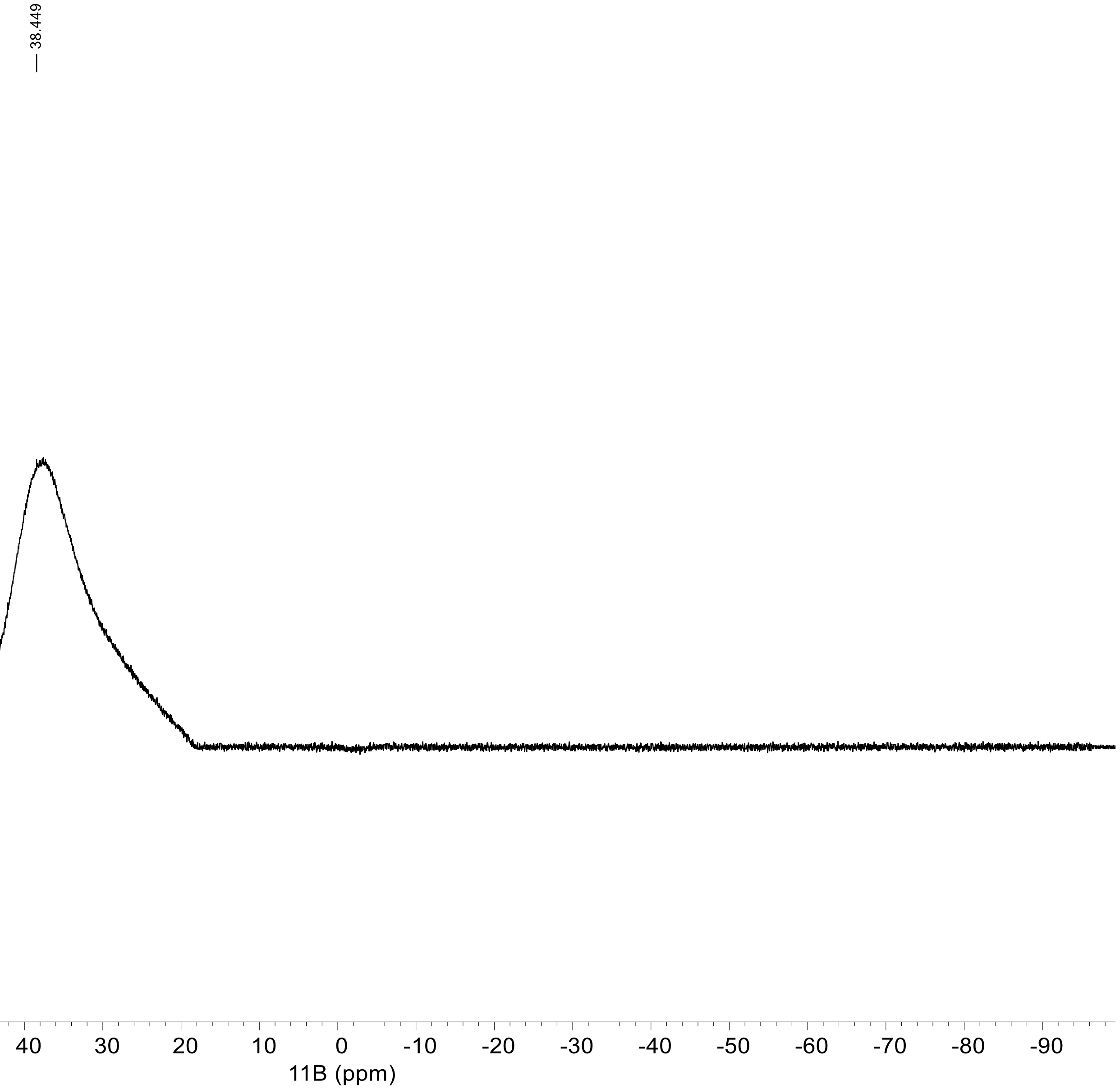

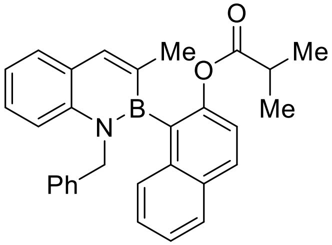

^{1}H, $CDCl_3$, 400 MHz

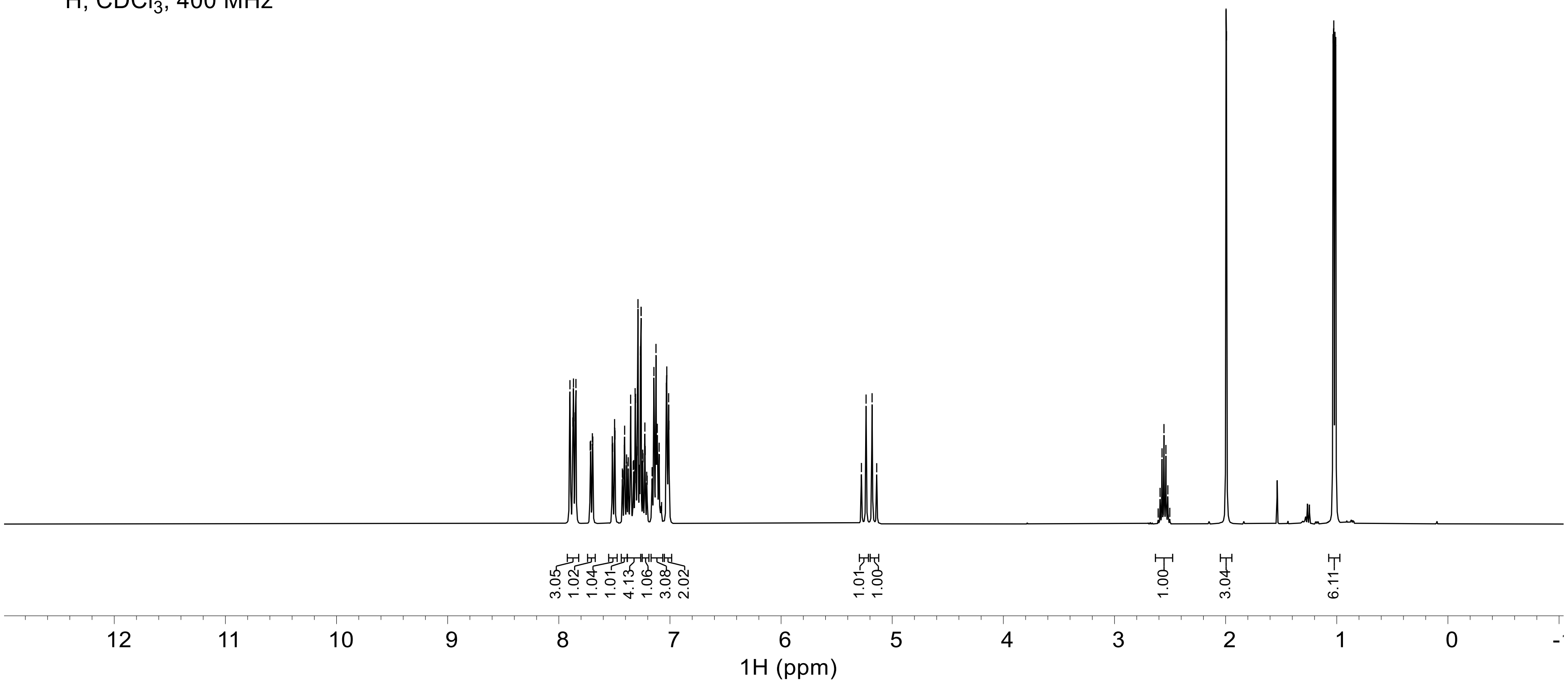

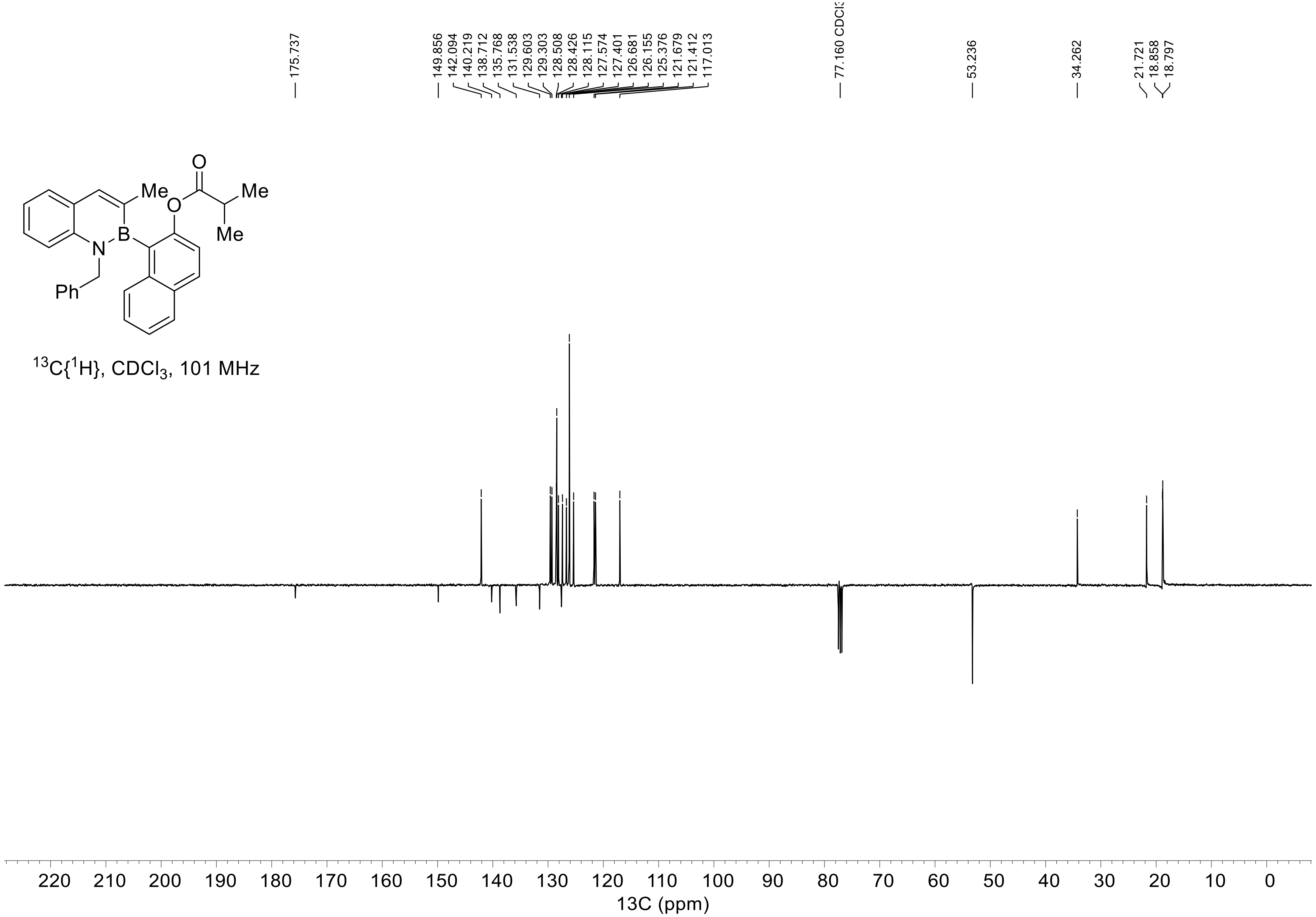
175.737
149.856
142.094
140.219
138.712
135.768
131.538
129.603
129.303
128.508
128.426
128.115
127.574
127.401
126.681
126.155
125.376
121.679
121.412
117.013
77.160 CDCl3
53.236
34.262
21.721
18.858
18.797
O
Me
O
Me
Me
B
N
Ph
13C{1H}, CDCl3, 101 MHz
220 210 200 190 180 170 160 150 140 130 120 110 100 90 80 70 60 50 40 30 20 10 0
13C (ppm)

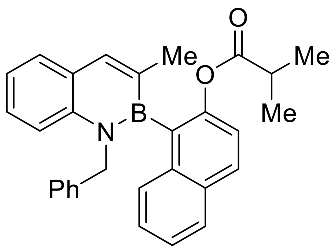

$^{11}B\{^{1}H\}$, $CDCl_3$, 128 MHz

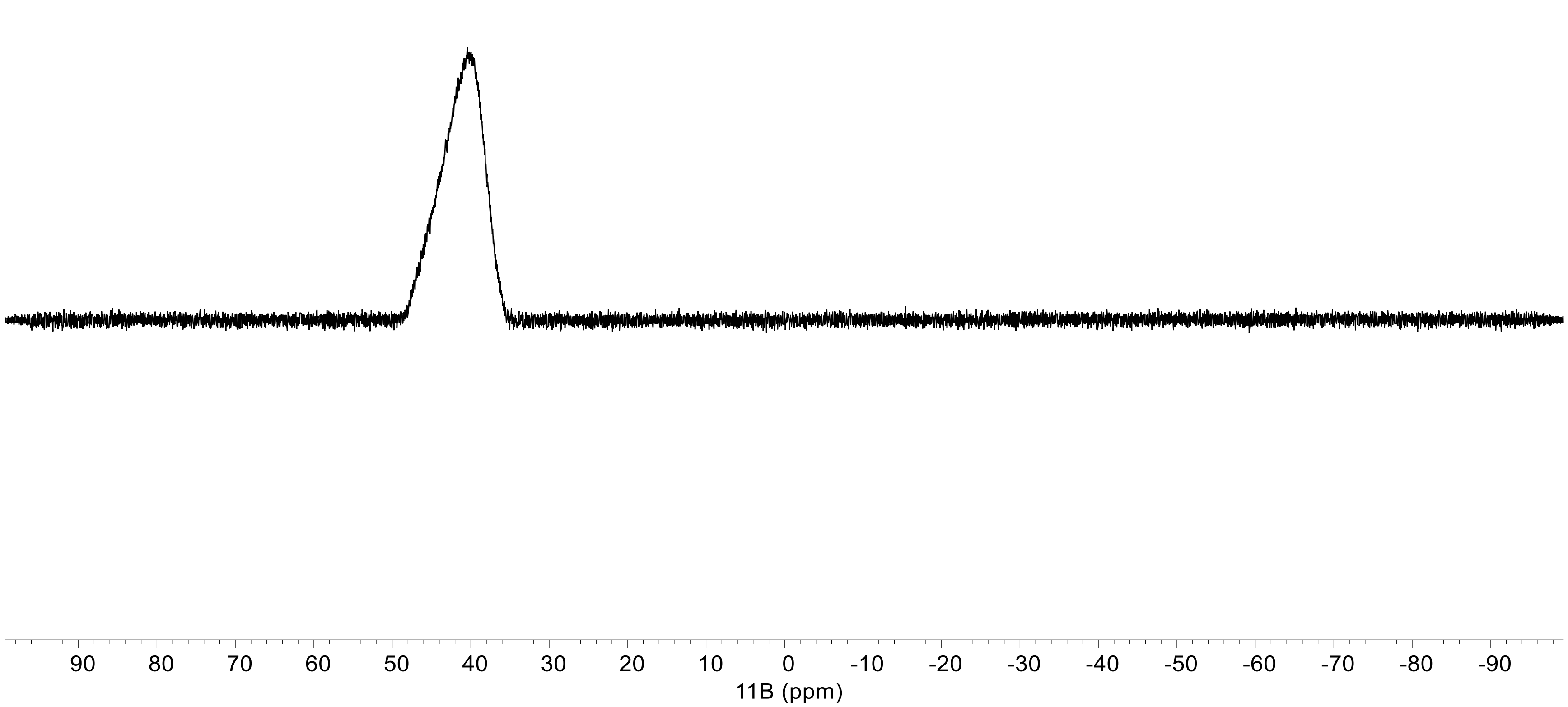

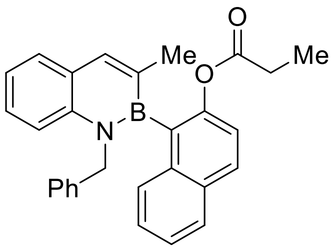

^{1}H, $CDCl_3$, 400 MHz

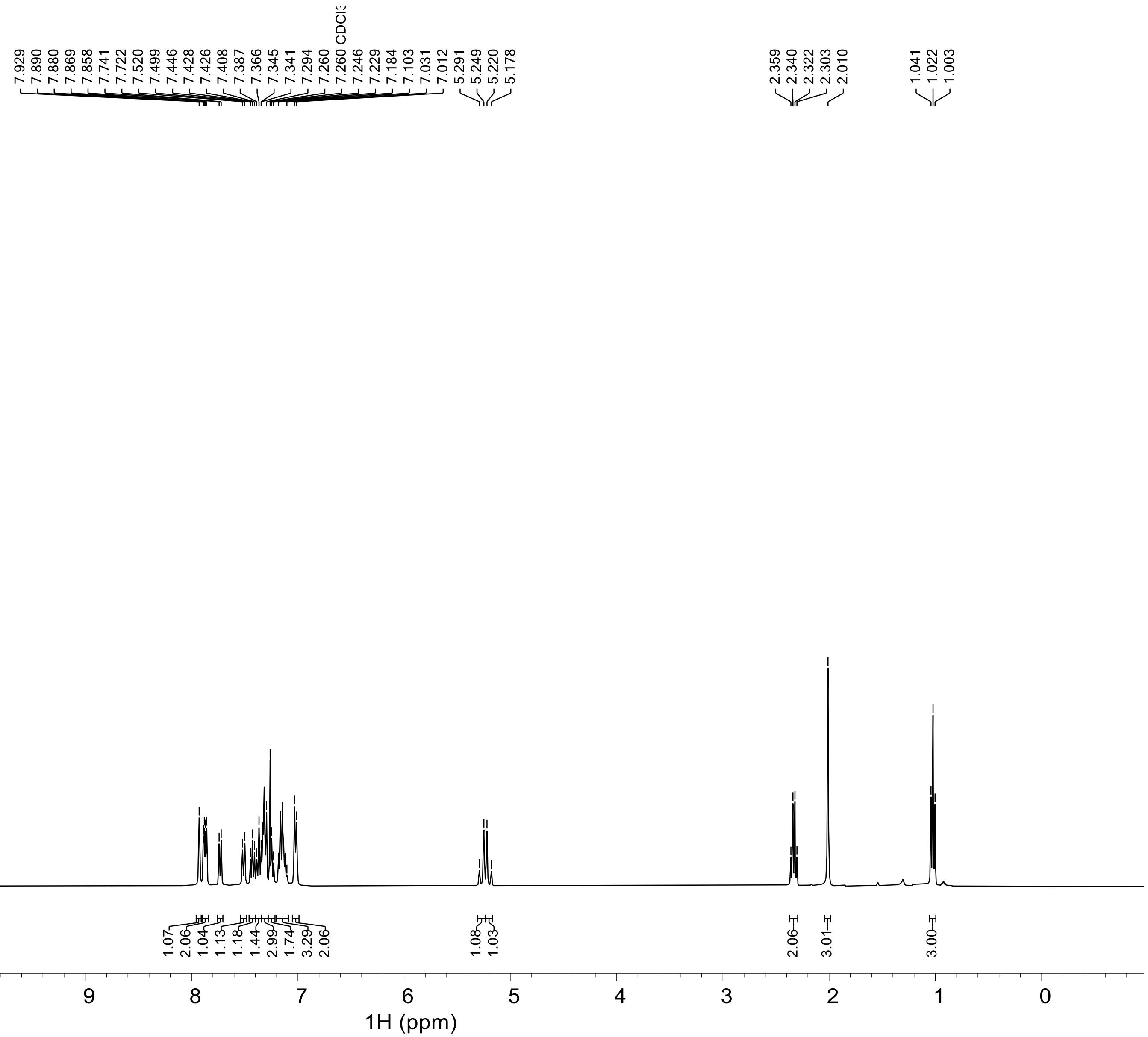

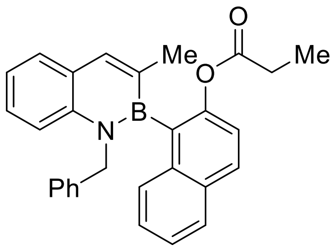

$^{13}C\ \{^1H\}$, $CDCl_3$, 101 MHz

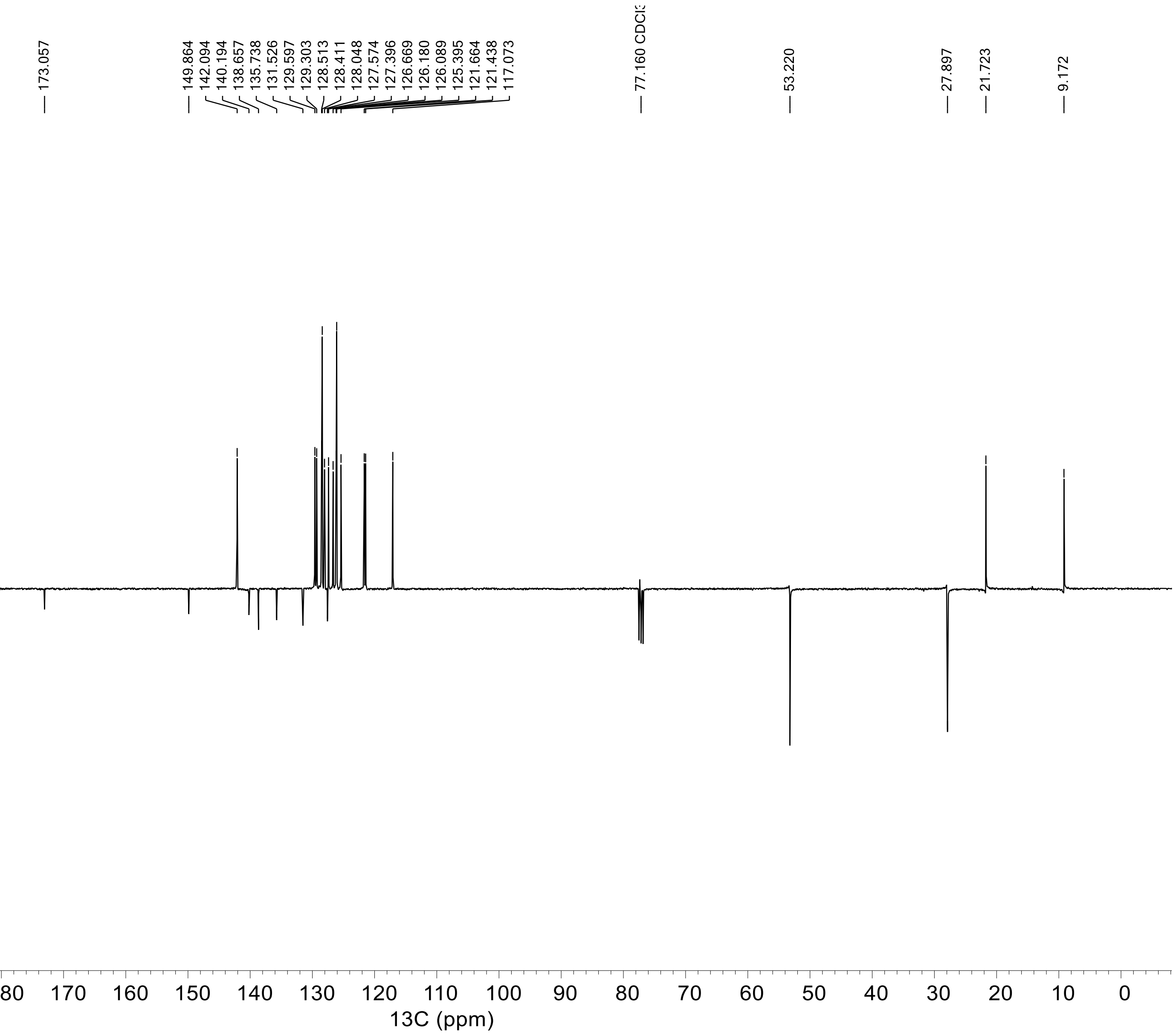

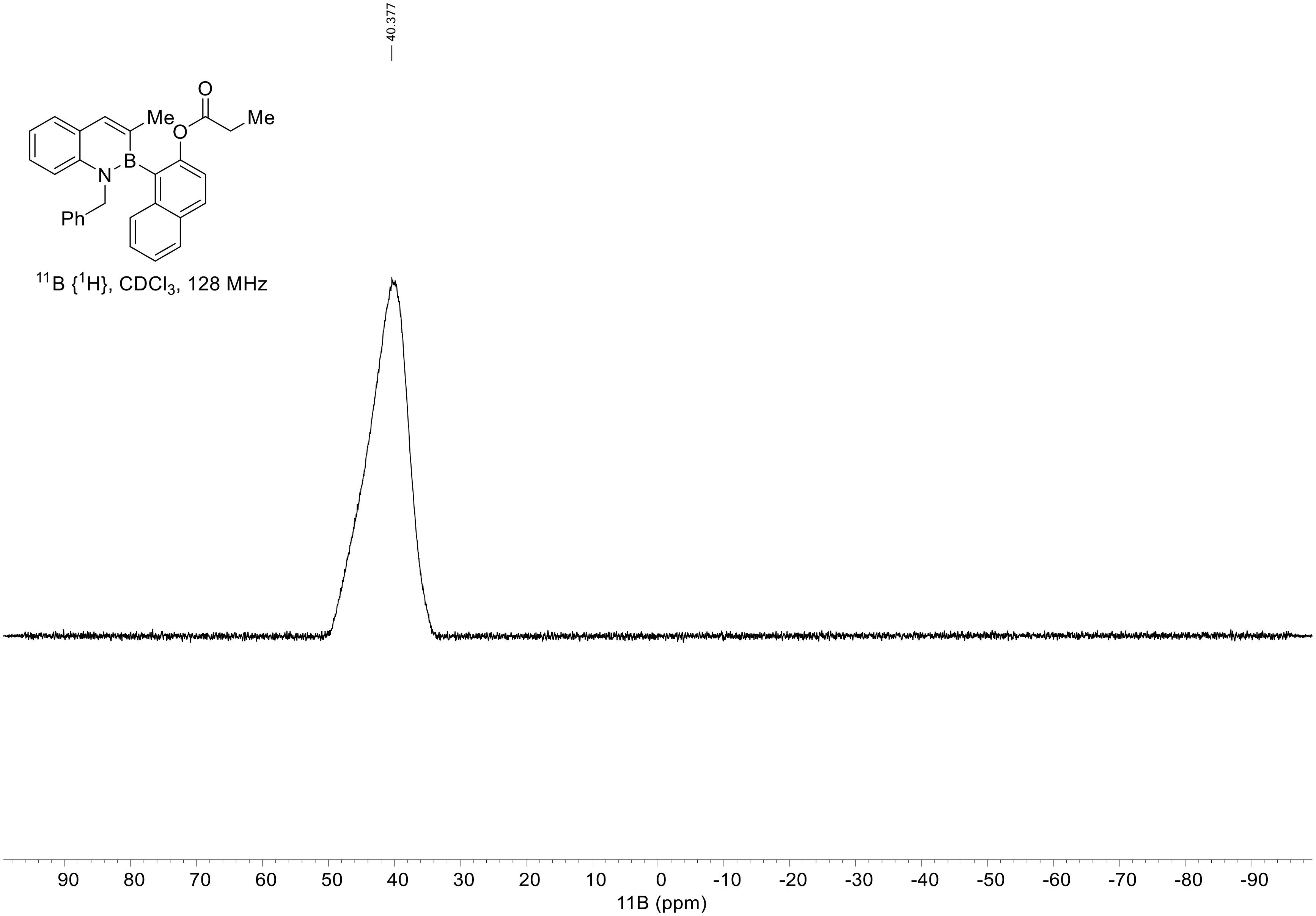
40.377
Me
O
O
Me
B
N
Ph
$^{11}B\ \{^{1}H\}$, $CDCl_3$, 128 MHz
90 80 70 60 50 40 30 20 10 0 -10 -20 -30 -40 -50 -60 -70 -80 -90
11B (ppm)

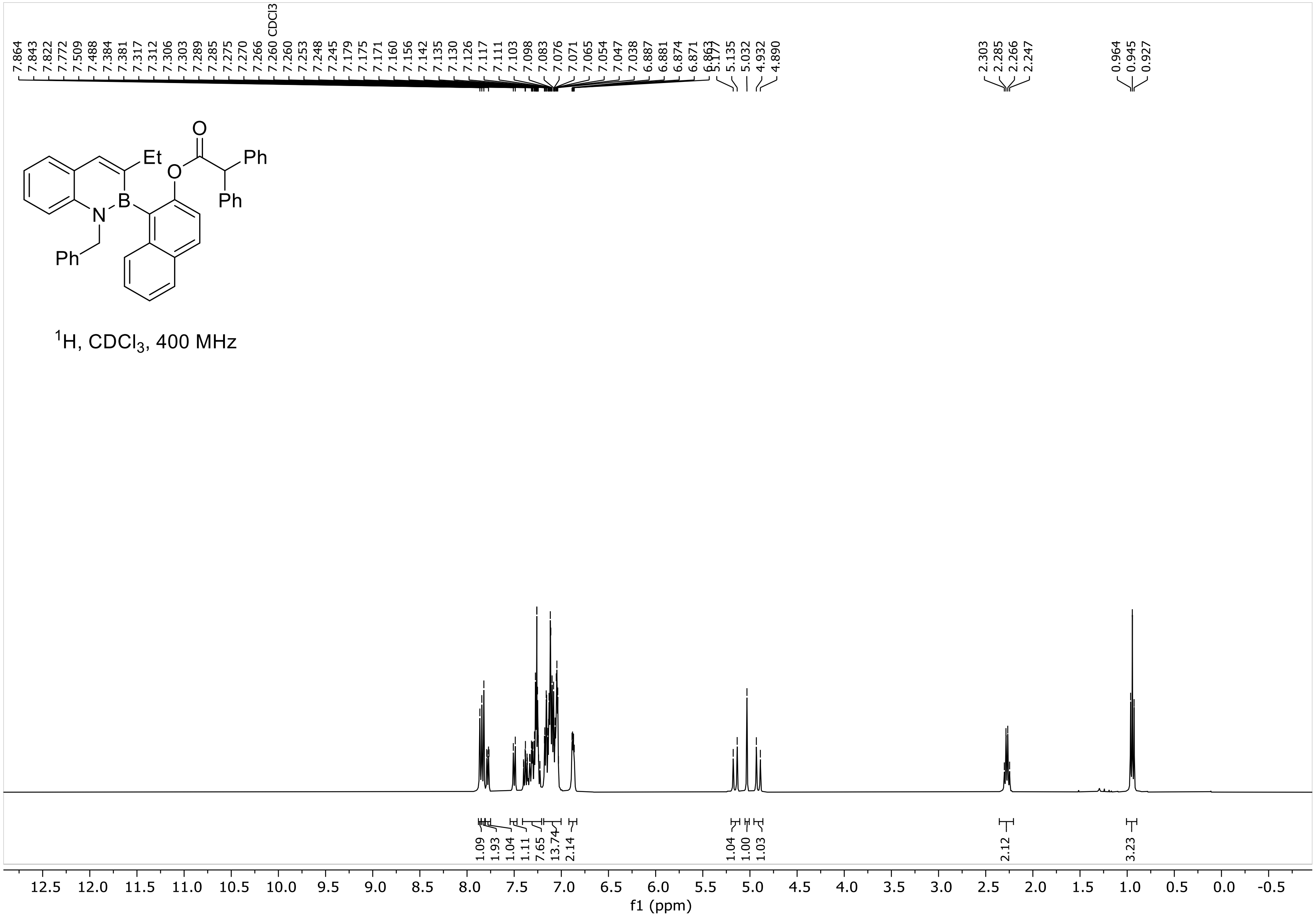

7.864 7.843 7.822 7.772 7.509 7.488 7.384 7.381 7.317 7.312 7.306 7.303 7.289 7.285 7.275 7.270 7.266 7.260 CDCl3 7.260 7.253 7.248 7.245 7.179 7.175 7.171 7.160 7.156 7.142 7.135 7.130 7.126 7.117 7.111 7.103 7.098 7.083 7.076 7.071 7.065 7.054 7.047 7.038 6.887 6.881 6.874 6.871 6.863 5.177 5.135 5.032 4.932 4.890
2.303 2.285 2.266 2.247
0.964 0.945 0.927
Et
O
O
Ph
Ph
B
N
Ph
^{1}H, CDCl$_3$, 400 MHz
1.09 1.93 1.04 1.11 7.65 13.74 2.14 1.04 1.00 1.03 2.12 3.23
12.5 12.0 11.5 11.0 10.5 10.0 9.5 9.0 8.5 8.0 7.5 7.0 6.5 6.0 5.5 5.0 4.5 4.0 3.5 3.0 2.5 2.0 1.5 1.0 0.5 0.0 -0.5
f1 (ppm)

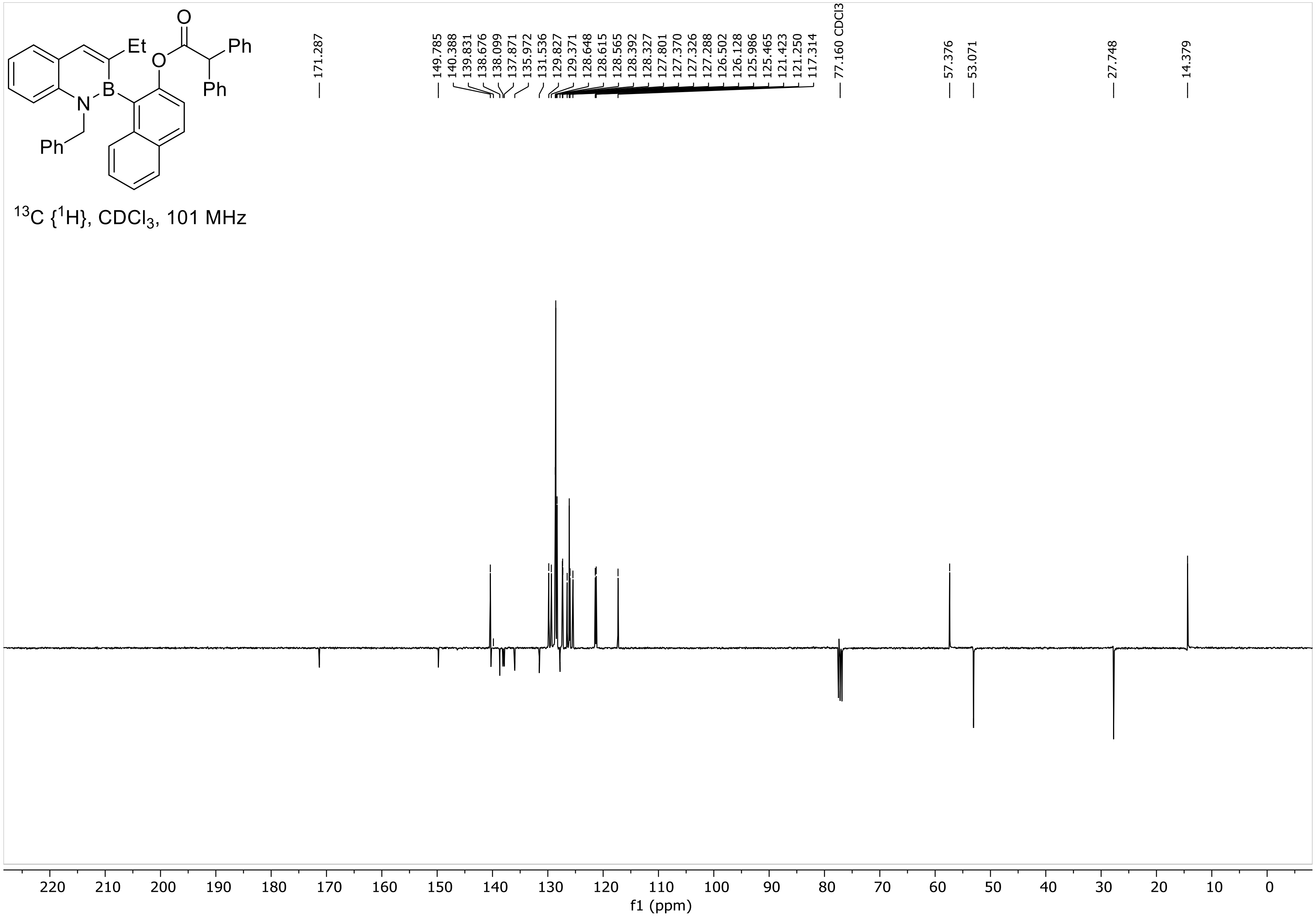
Et
O
O
Ph
Ph
N
B
Ph
$^{13}C\ \{^{1}H\}$, $CDCl_3$, 101 MHz
171.287
149.785
140.388
139.831
138.676
138.099
137.871
135.972
131.536
129.827
129.371
128.648
128.615
128.565
128.392
128.327
127.801
127.370
127.326
127.288
126.502
126.128
125.986
125.465
121.423
121.250
117.314
77.160 CDCl3
57.376
53.071
27.748
14.379
220 210 200 190 180 170 160 150 140 130 120 110 100 90 80 70 60 50 40 30 20 10 0
f1 (ppm)

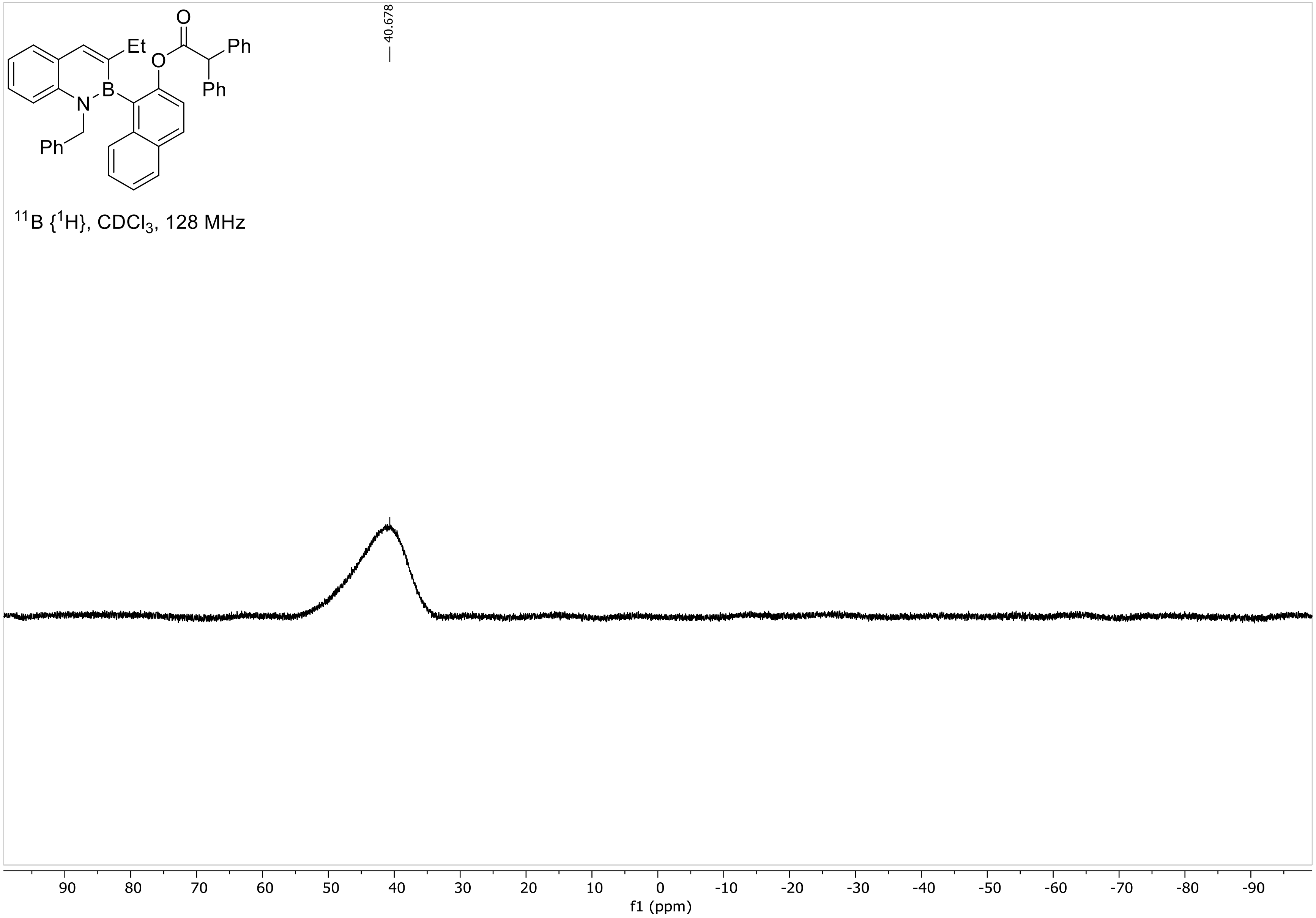

11B {1H}, CDCl3, 128 MHz
40.678
90 80 70 60 50 40 30 20 10 0 -10 -20 -30 -40 -50 -60 -70 -80 -90
f1 (ppm)

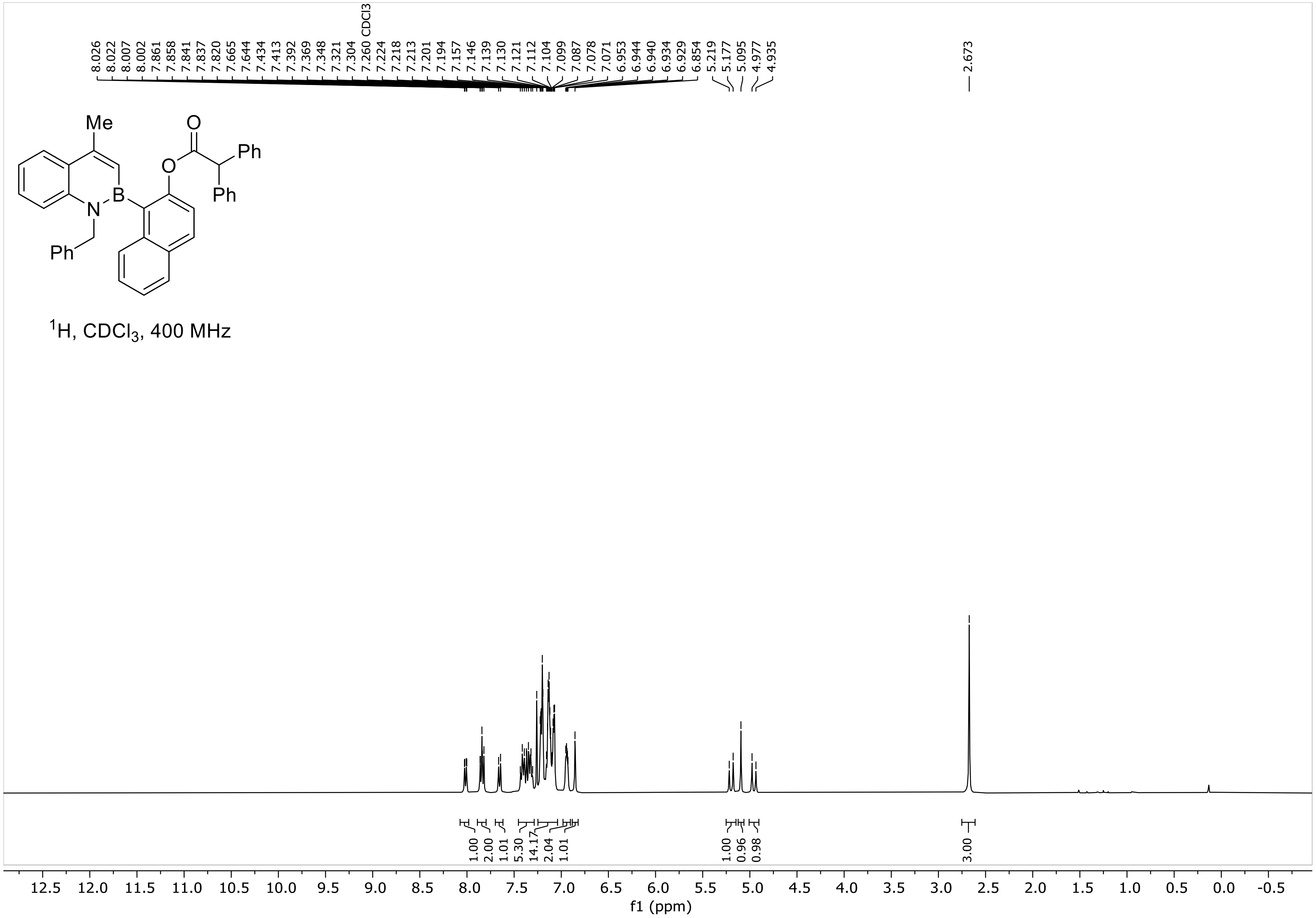

8.026 8.022 8.007 8.002 7.861 7.858 7.841 7.837 7.820 7.665 7.644 7.434 7.413 7.392 7.369 7.348 7.321 7.304 7.260 CDCl3 7.224 7.218 7.213 7.201 7.194 7.157 7.146 7.139 7.130 7.121 7.112 7.104 7.099 7.087 7.078 7.071 6.953 6.944 6.940 6.934 6.929 6.854 5.219 5.177 5.095 4.977 4.935
2.673
Me
O
Ph
O
N
B
Ph
Ph
1H, CDCl3, 400 MHz
1.00 2.00 1.01 5.30 14.17 2.04 1.01
1.00 0.96 0.98
3.00
12.5 12.0 11.5 11.0 10.5 10.0 9.5 9.0 8.5 8.0 7.5 7.0 6.5 6.0 5.5 5.0 4.5 4.0 3.5 3.0 2.5 2.0 1.5 1.0 0.5 0.0 -0.5
f1 (ppm)

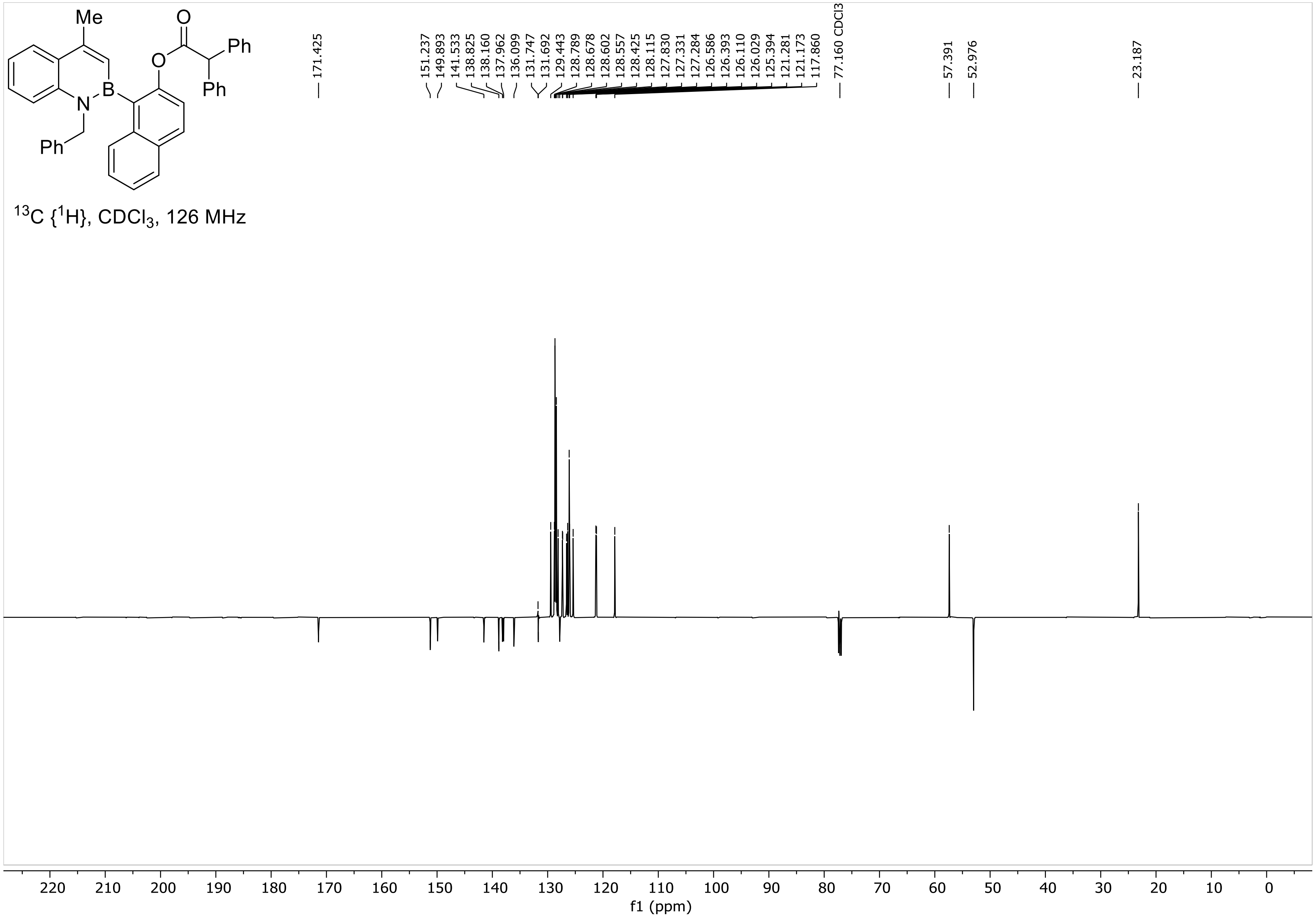

Me
O
Ph
Ph
N
B
Ph
13C {1H}, CDCl3, 126 MHz
171.425
151.237
149.893
141.533
138.825
138.160
137.962
136.099
131.747
131.692
129.443
128.789
128.678
128.602
128.557
128.425
128.115
127.830
127.331
127.284
126.586
126.393
126.110
126.029
125.394
121.281
121.173
117.860
77.160 CDCl3
57.391
52.976
23.187
220 210 200 190 180 170 160 150 140 130 120 110 100 90 80 70 60 50 40 30 20 10 0
f1 (ppm)

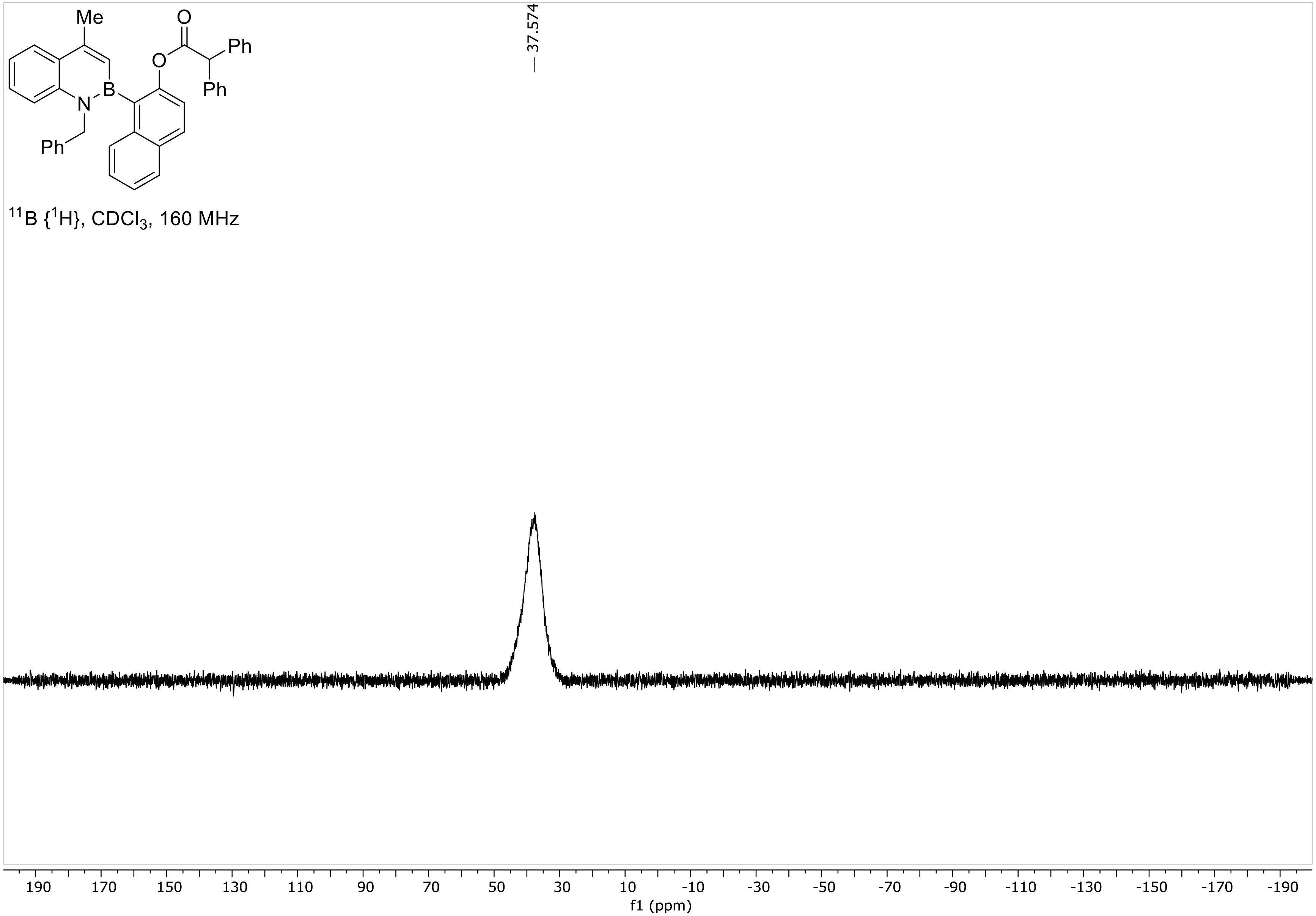

Me
O
Ph
Ph
O
N
B
Ph
11B {1H}, CDCl3, 160 MHz
37.574
190 170 150 130 110 90 70 50 30 10 -10 -30 -50 -70 -90 -110 -130 -150 -170 -190
f1 (ppm)

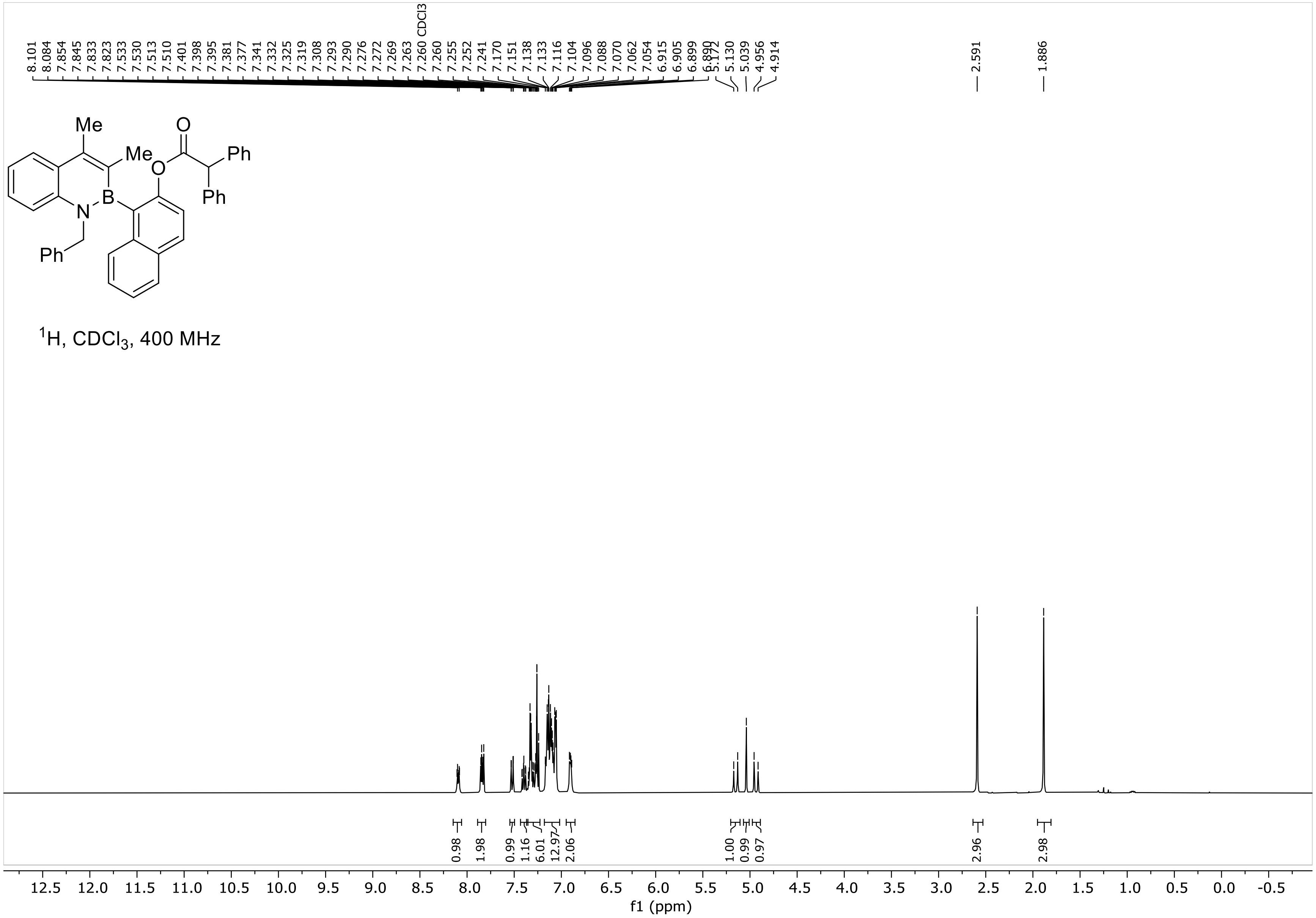

CDCl3
8.101 8.084 7.854 7.845 7.833 7.823 7.533 7.530 7.513 7.510 7.401 7.398 7.395 7.381 7.377 7.341 7.332 7.325 7.319 7.308 7.293 7.290 7.276 7.272 7.269 7.263 7.260 7.260 7.255 7.252 7.241 7.170 7.151 7.138 7.133 7.116 7.104 7.096 7.088 7.070 7.062 7.054 6.915 6.905 6.899 6.890 5.172 5.130 5.039 4.956 4.914
2.591
1.886
Me
O
Me
O
Ph
B
N
Ph
Ph
1H, CDCl3, 400 MHz
0.98 1.98 0.99 1.16 6.01 12.97 2.06 1.00 0.99 0.97 2.96 2.98
12.5 12.0 11.5 11.0 10.5 10.0 9.5 9.0 8.5 8.0 7.5 7.0 6.5 6.0 5.5 5.0 4.5 4.0 3.5 3.0 2.5 2.0 1.5 1.0 0.5 0.0 -0.5
f1 (ppm)

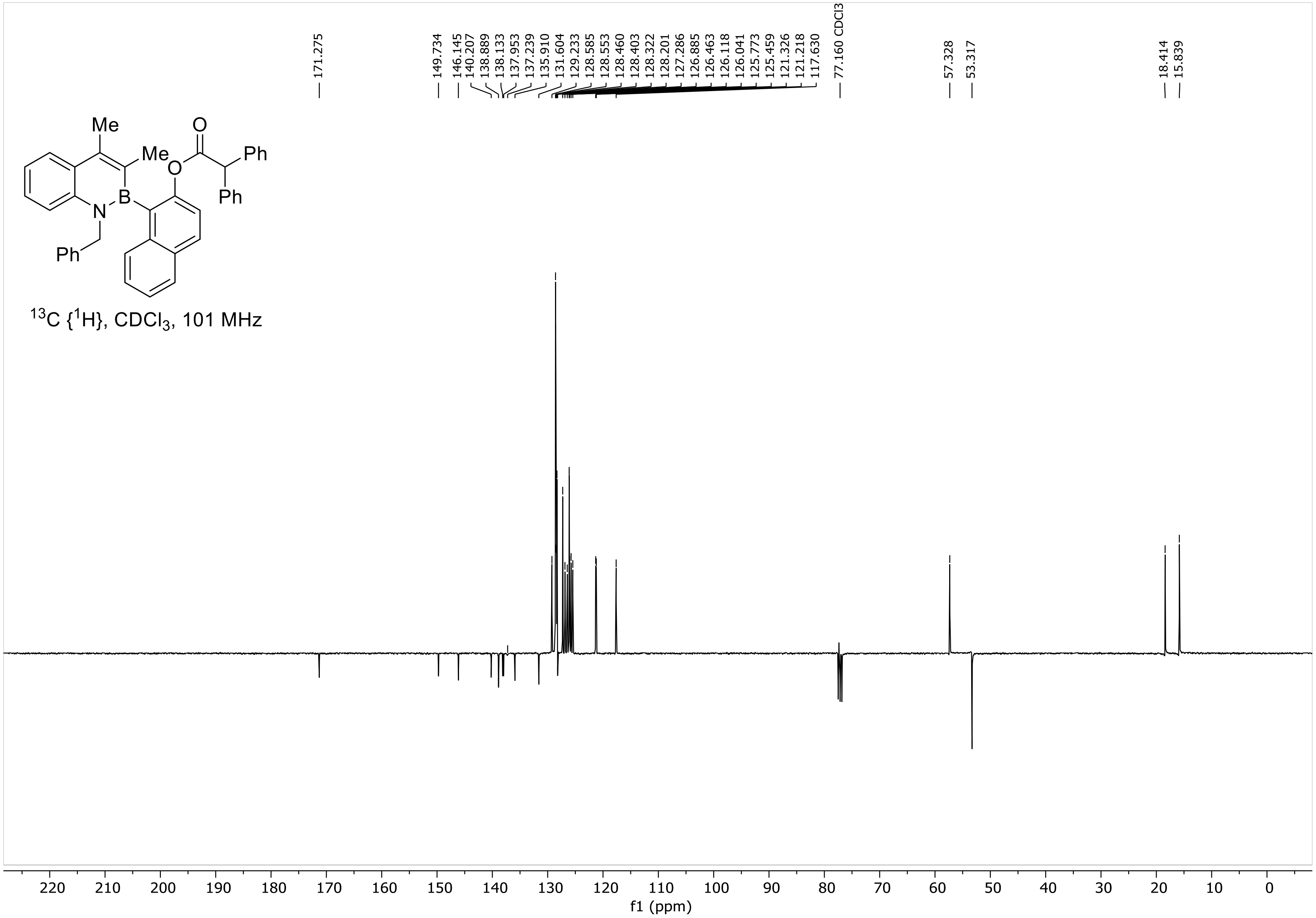

171.275
149.734
146.145
140.207
138.889
138.133
137.953
137.239
135.910
131.604
129.233
128.585
128.553
128.460
128.403
128.322
128.201
127.286
126.885
126.463
126.118
126.041
125.773
125.459
121.326
121.218
117.630
77.160 CDCl3
57.328
53.317
18.414
15.839
Me
Me
O
O
Ph
Ph
B
N
Ph
13C {1H}, CDCl3, 101 MHz
220 210 200 190 180 170 160 150 140 130 120 110 100 90 80 70 60 50 40 30 20 10 0
f1 (ppm)

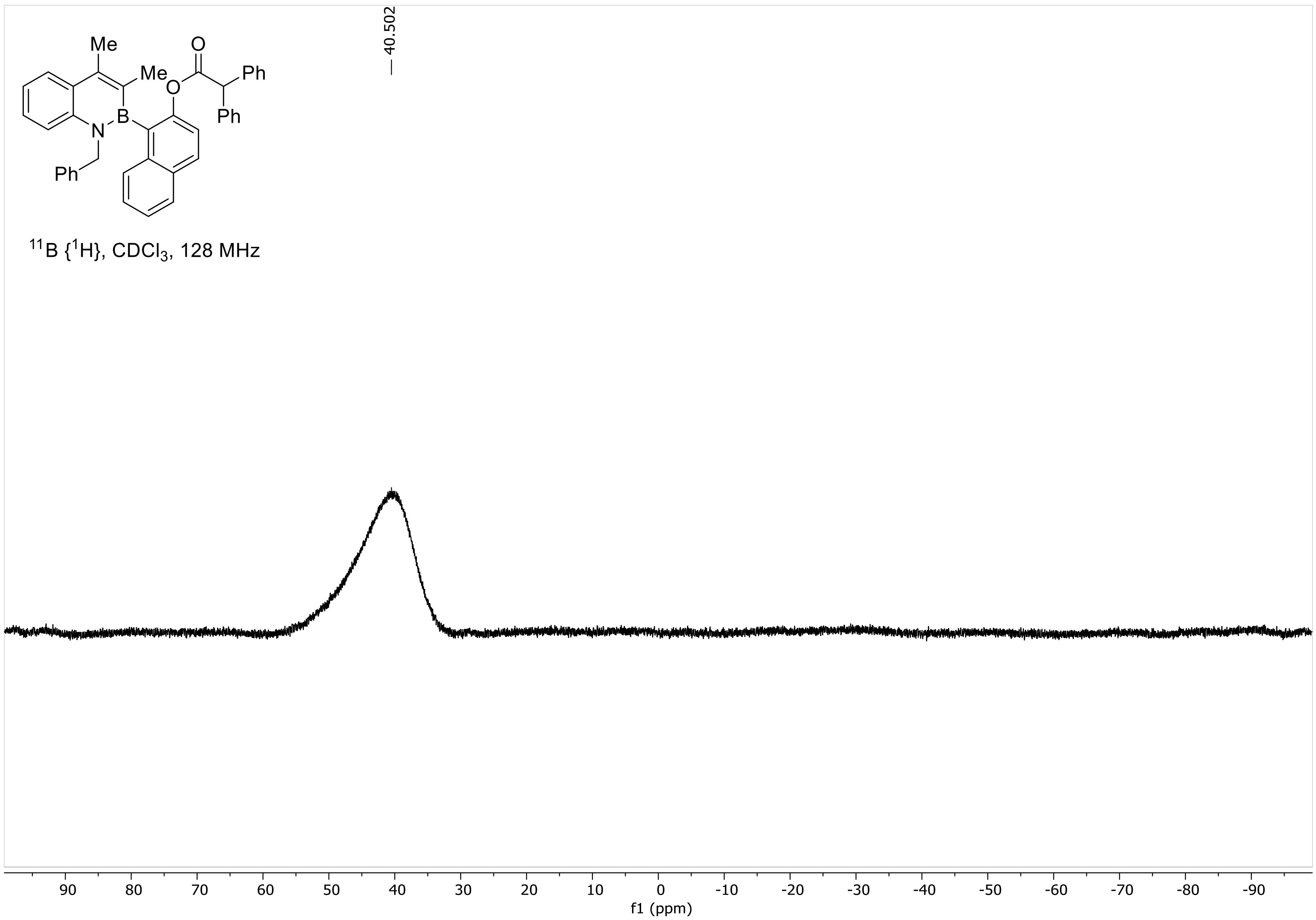
$^{11}B\{^1H\}$, $CDCl_3$, 128 MHz
40.502
90 80 70 60 50 40 30 20 10 0 -10 -20 -30 -40 -50 -60 -70 -80 -90
f1 (ppm)

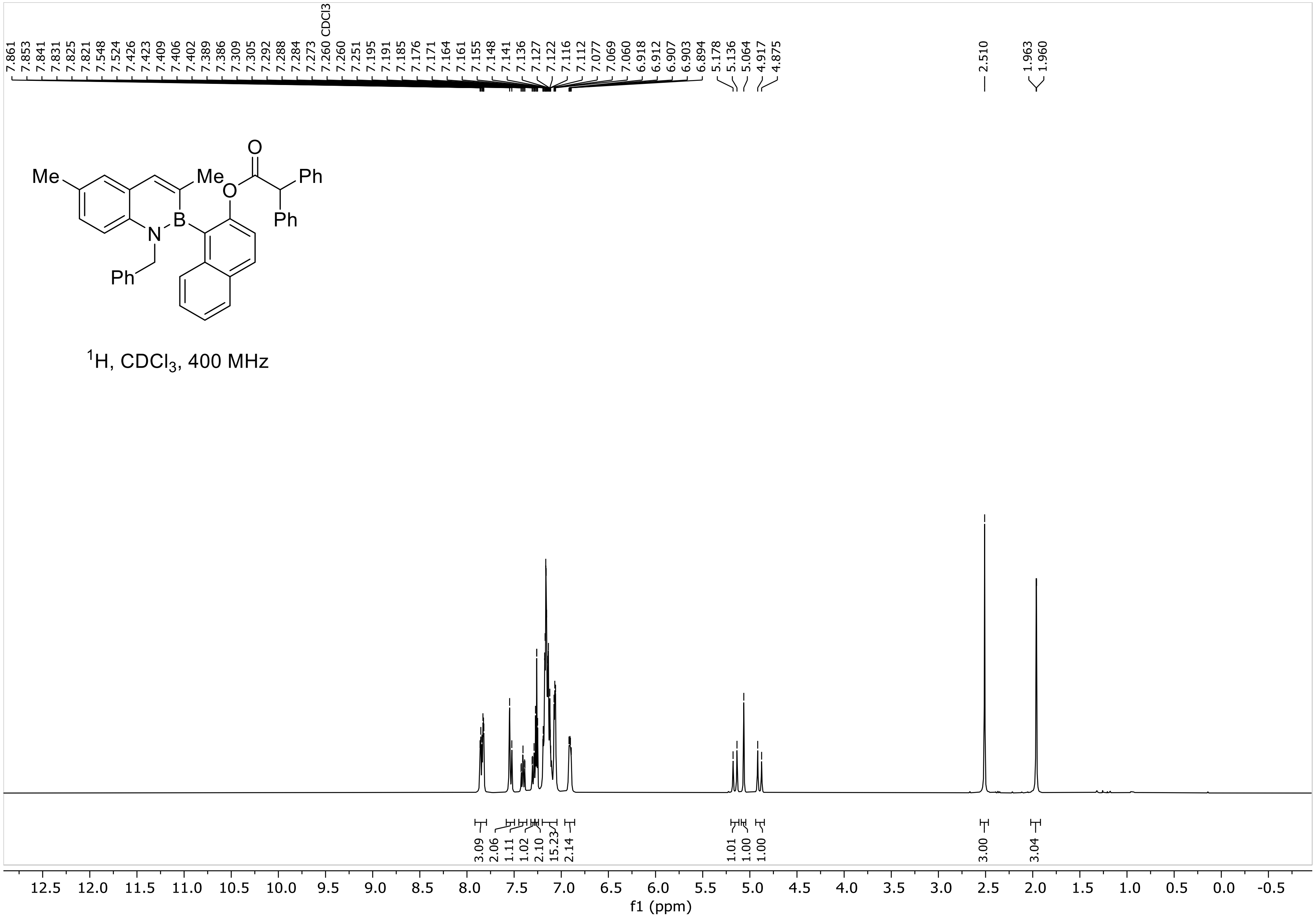

7.861 7.853 7.841 7.831 7.825 7.821 7.548 7.524 7.426 7.423 7.409 7.406 7.402 7.389 7.386 7.309 7.305 7.292 7.288 7.284 7.273 7.260 CDCl3 7.260 7.251 7.195 7.191 7.185 7.176 7.171 7.164 7.161 7.155 7.148 7.141 7.136 7.127 7.122 7.116 7.112 7.077 7.069 7.060 6.918 6.912 6.907 6.903 6.894 5.178 5.136 5.064 4.917 4.875 2.510 1.963 1.960
Me
Me
O
O
Ph
Ph
N
B
Ph
^{1}H, CDCl$_3$, 400 MHz
3.09 2.06 1.11 1.02 2.10 15.23 2.14 1.01 1.00 1.00 3.00 3.04
12.5 12.0 11.5 11.0 10.5 10.0 9.5 9.0 8.5 8.0 7.5 7.0 6.5 6.0 5.5 5.0 4.5 4.0 3.5 3.0 2.5 2.0 1.5 1.0 0.5 0.0 -0.5
f1 (ppm)

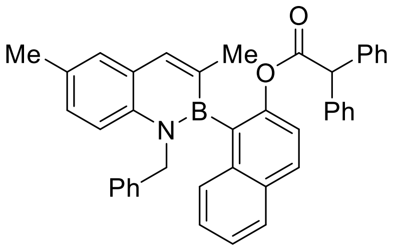

$^{13}C\ \{^{1}H\}$, $CDCl_3$, 126 MHz

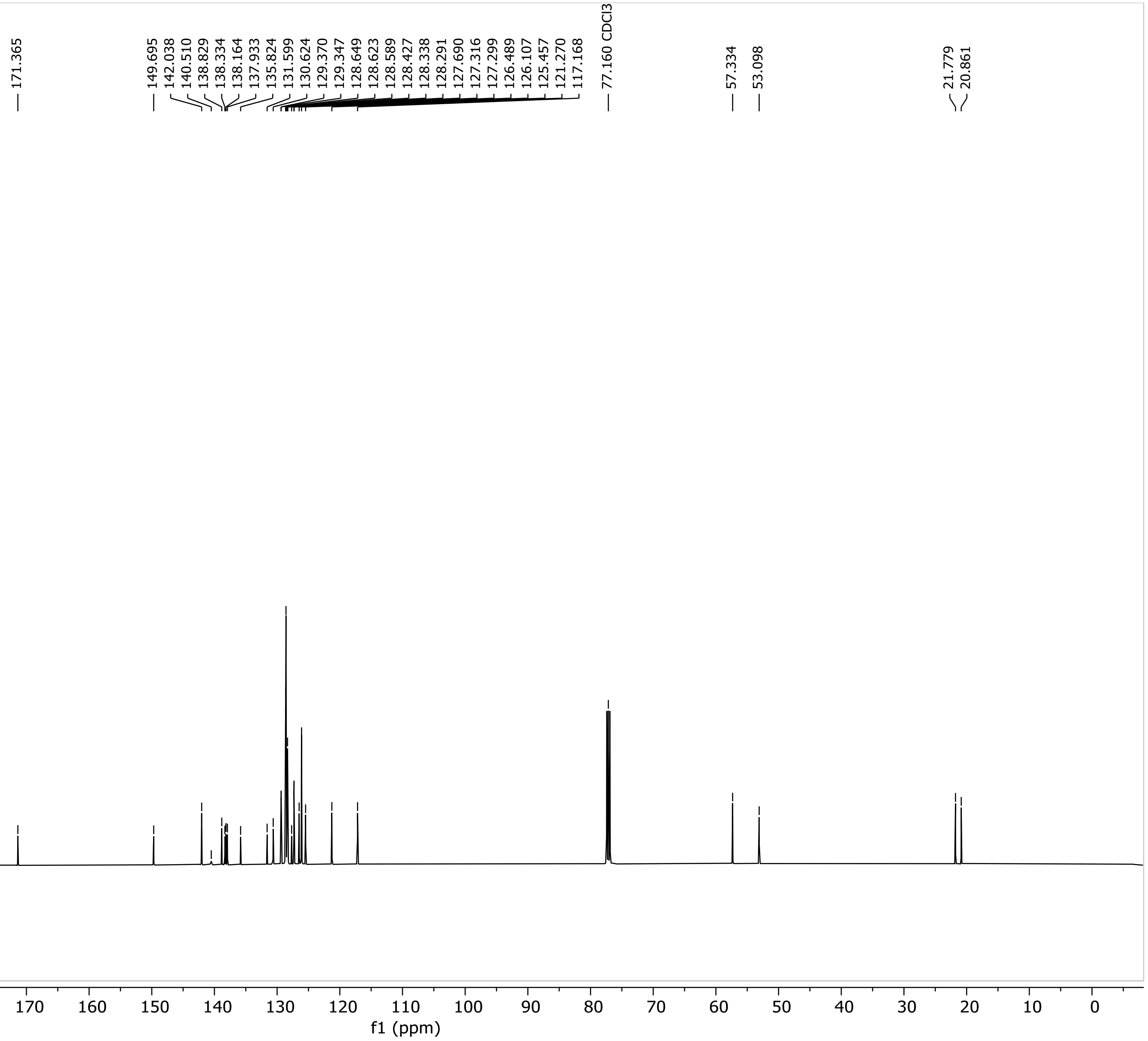

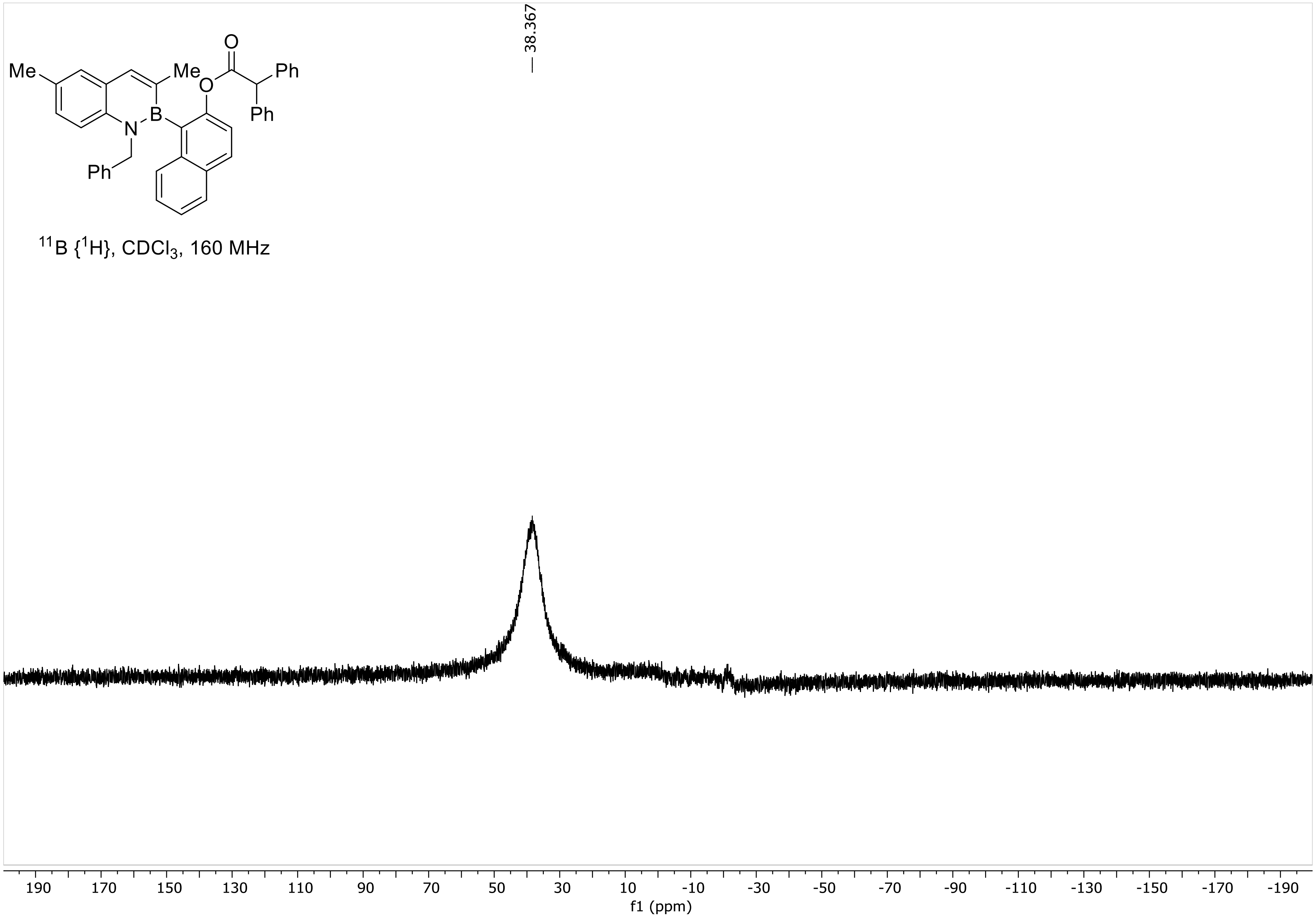
38.367
Me
Me
O
O
Ph
Ph
B
N
Ph
11B {1H}, CDCl3, 160 MHz
190 170 150 130 110 90 70 50 30 10 -10 -30 -50 -70 -90 -110 -130 -150 -170 -190
f1 (ppm)

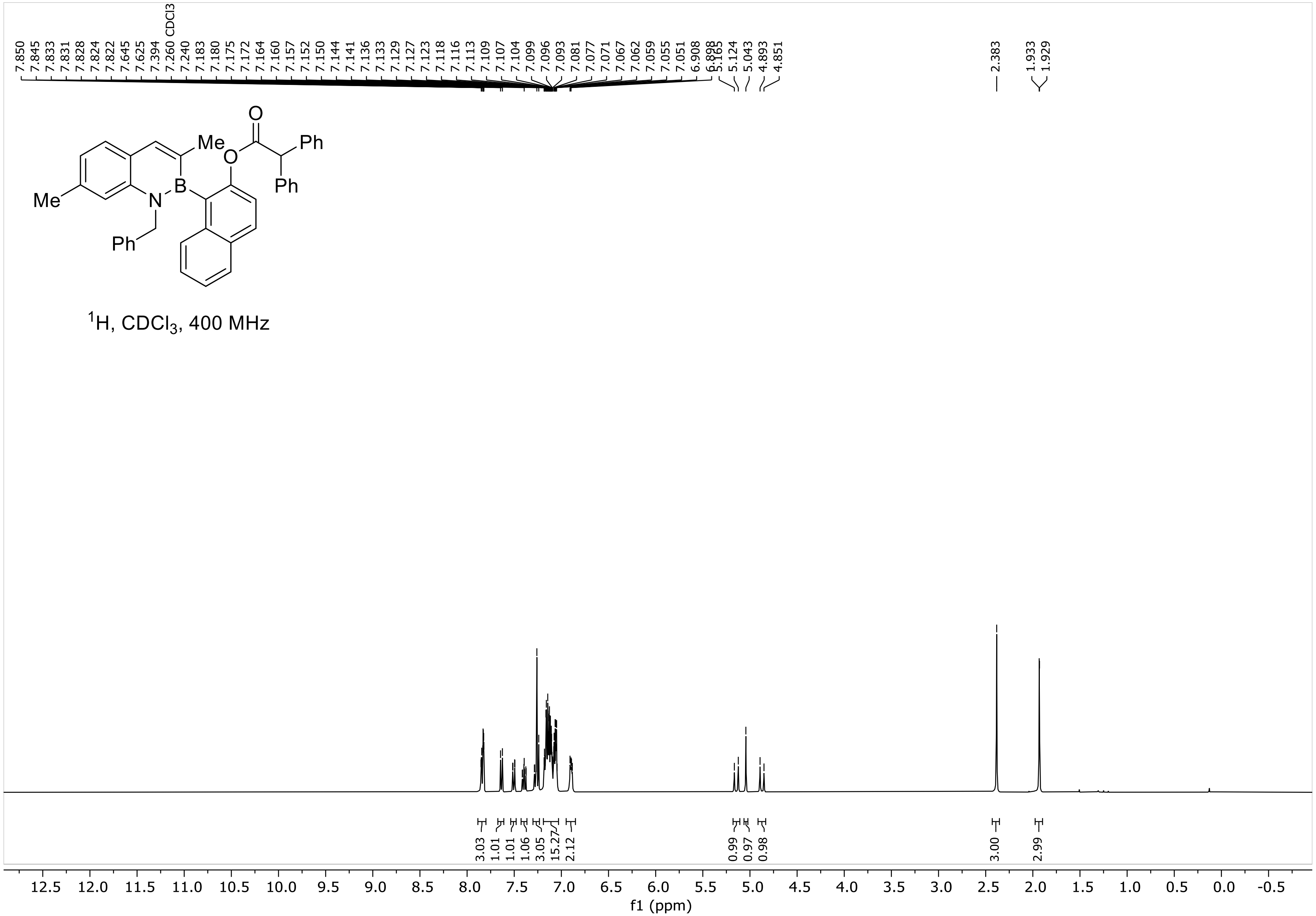

Me
Me
O
O
Ph
Ph
B
N
Ph
Me
¹H, CDCl₃, 400 MHz
f1 (ppm)

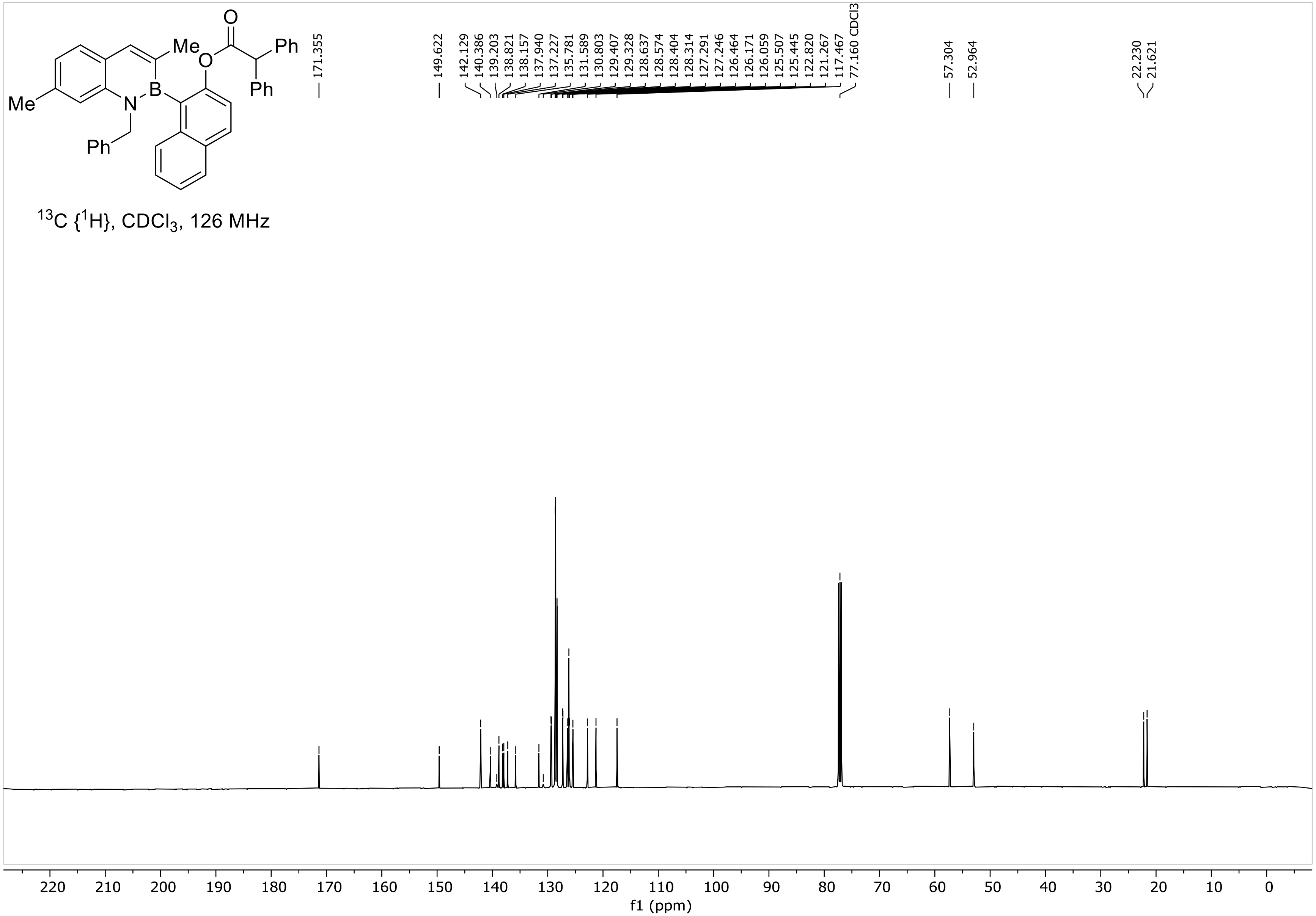
Me
Me
Me
N
B
O
O
Ph
Ph
Ph
^{13}C {^1H}, $CDCl_3$, 126 MHz
171.355
149.622
142.129
140.386
139.203
138.821
138.157
137.940
137.227
135.781
131.589
130.803
129.407
129.328
128.637
128.574
128.404
128.314
127.291
127.246
126.464
126.171
126.059
125.507
125.445
122.820
121.267
117.467
77.160 CDCl3
57.304
52.964
22.230
21.621
220 210 200 190 180 170 160 150 140 130 120 110 100 90 80 70 60 50 40 30 20 10 0
f1 (ppm)

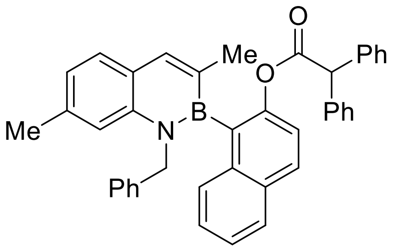

^{11}B {^{1}H}, $CDCl_3$, 160 MHz

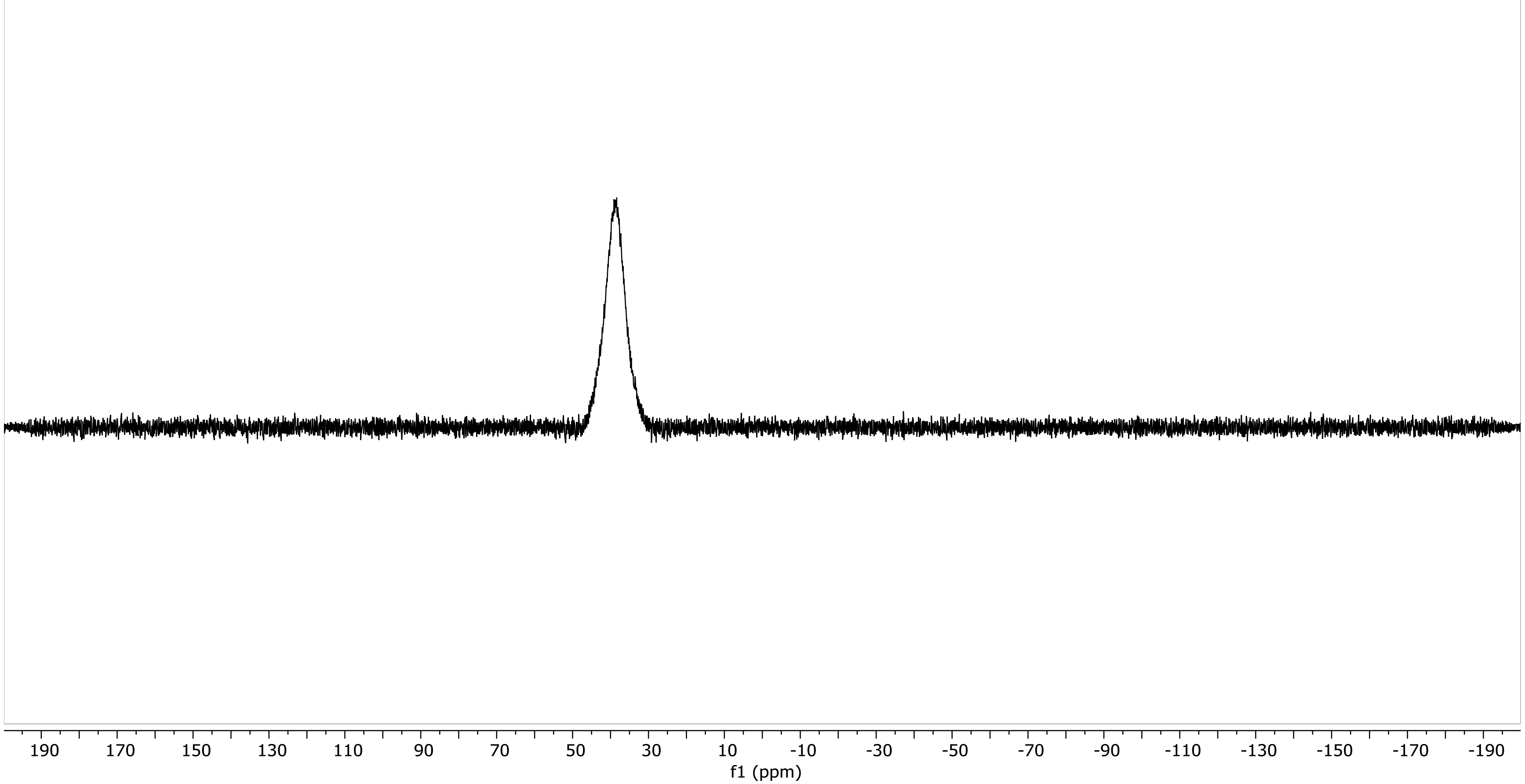

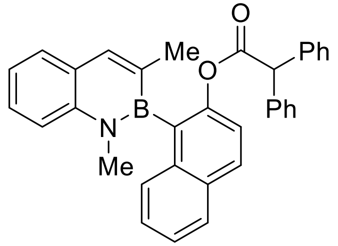

^{1}H, $CDCl_3$, 400 MHz

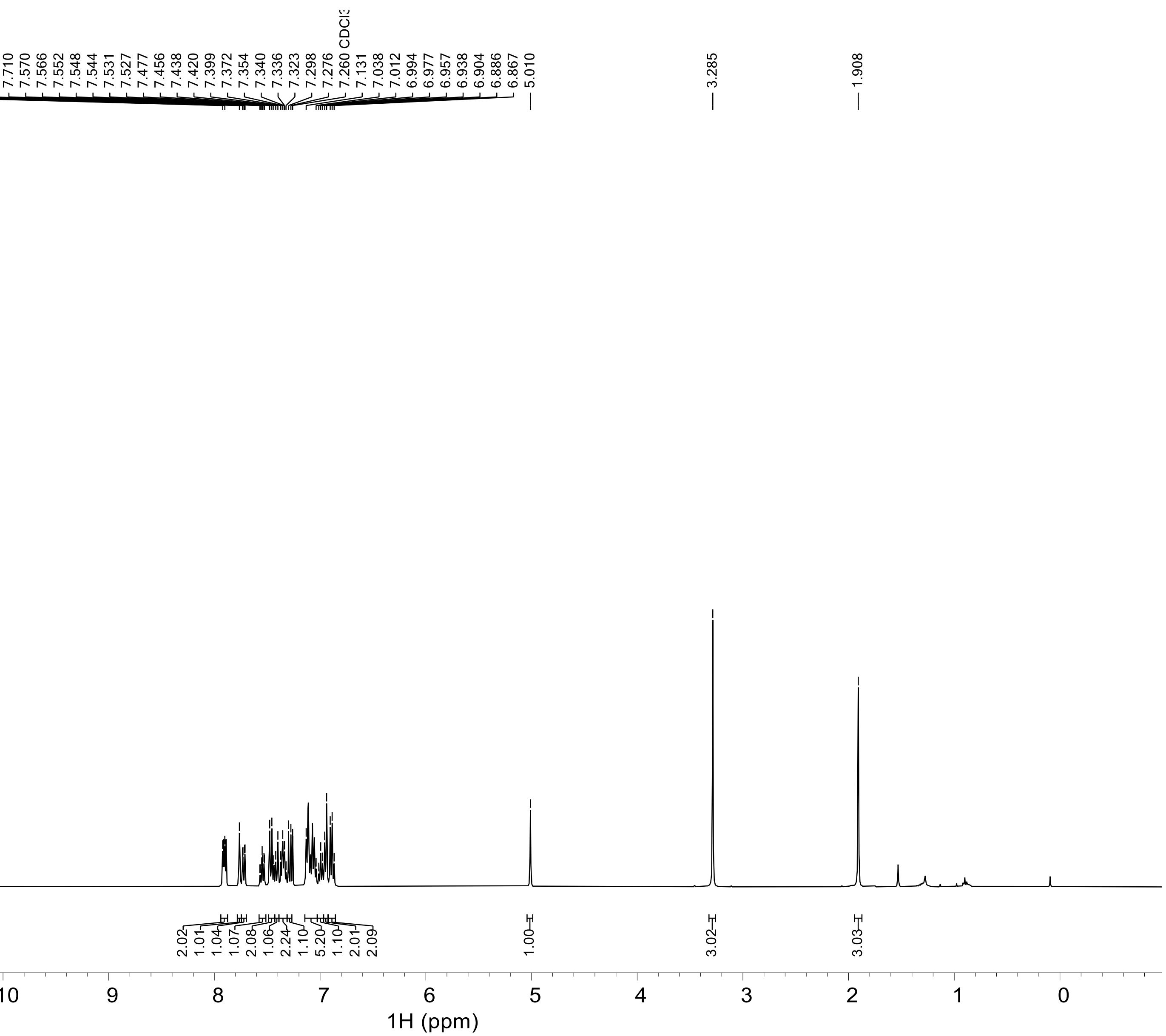

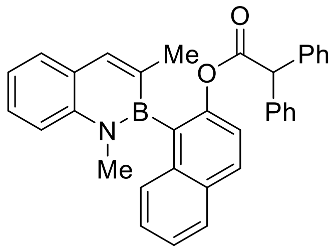

$^{13}C\ \{^1H\}$, $CDCl_3$, 126 MHz

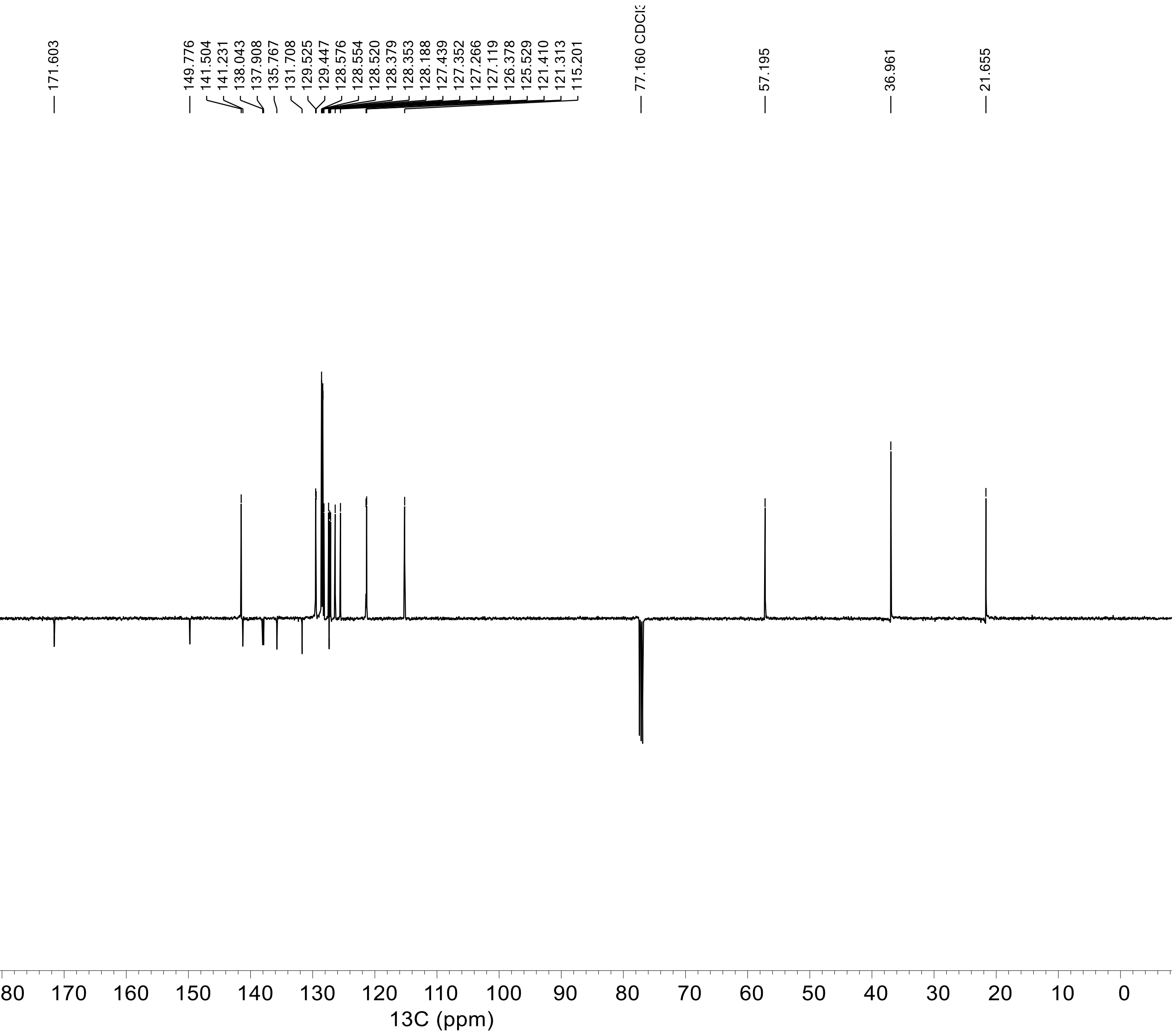

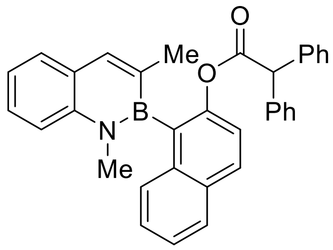

$^{11}B\ \{^{1}H\}$, $CDCl_3$, 128 MHz

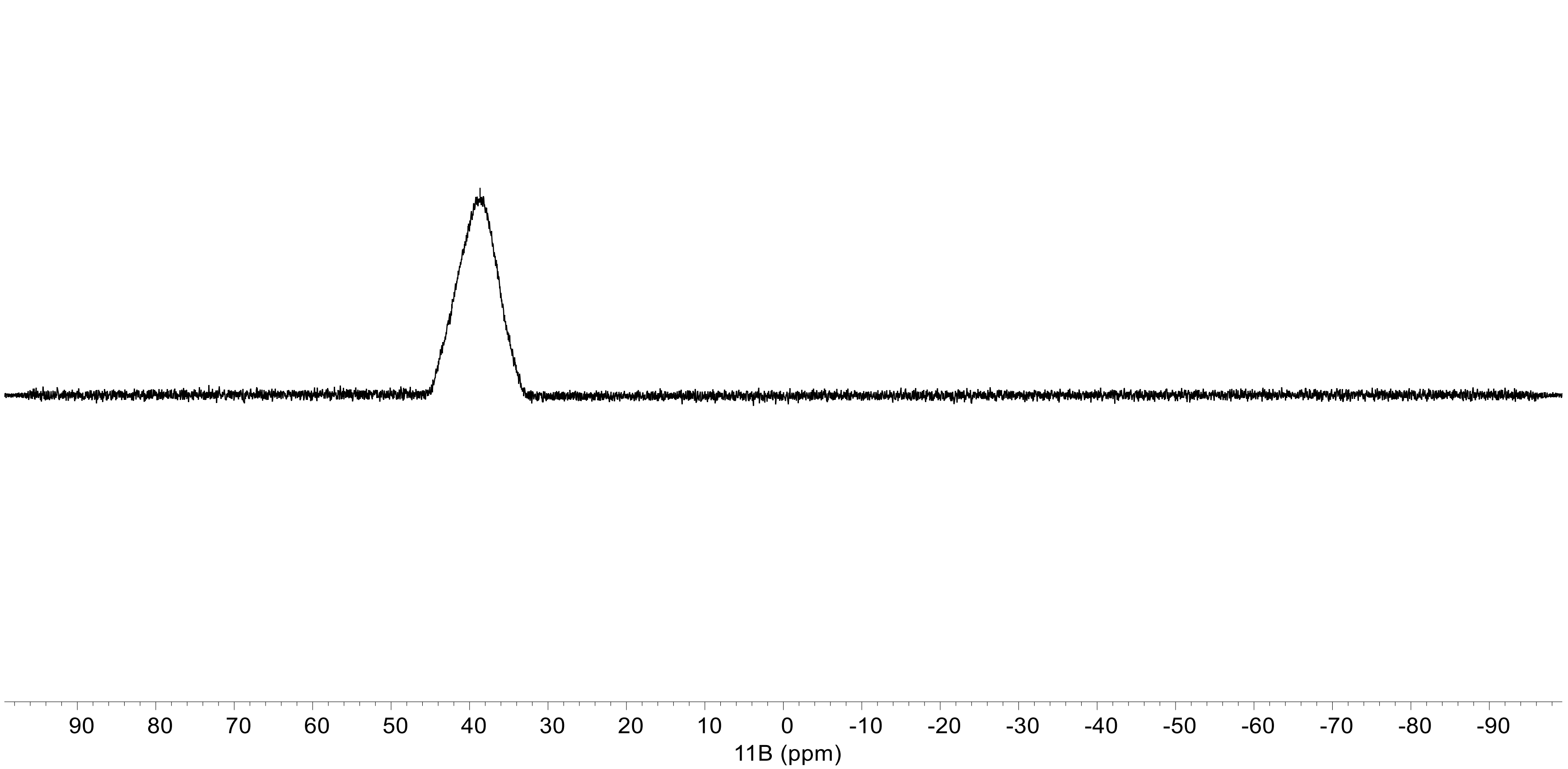

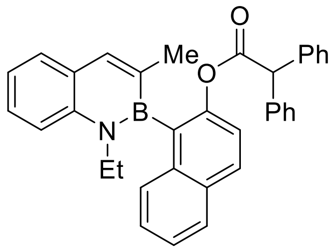

^1H, $CDCl_3$, 400 MHz

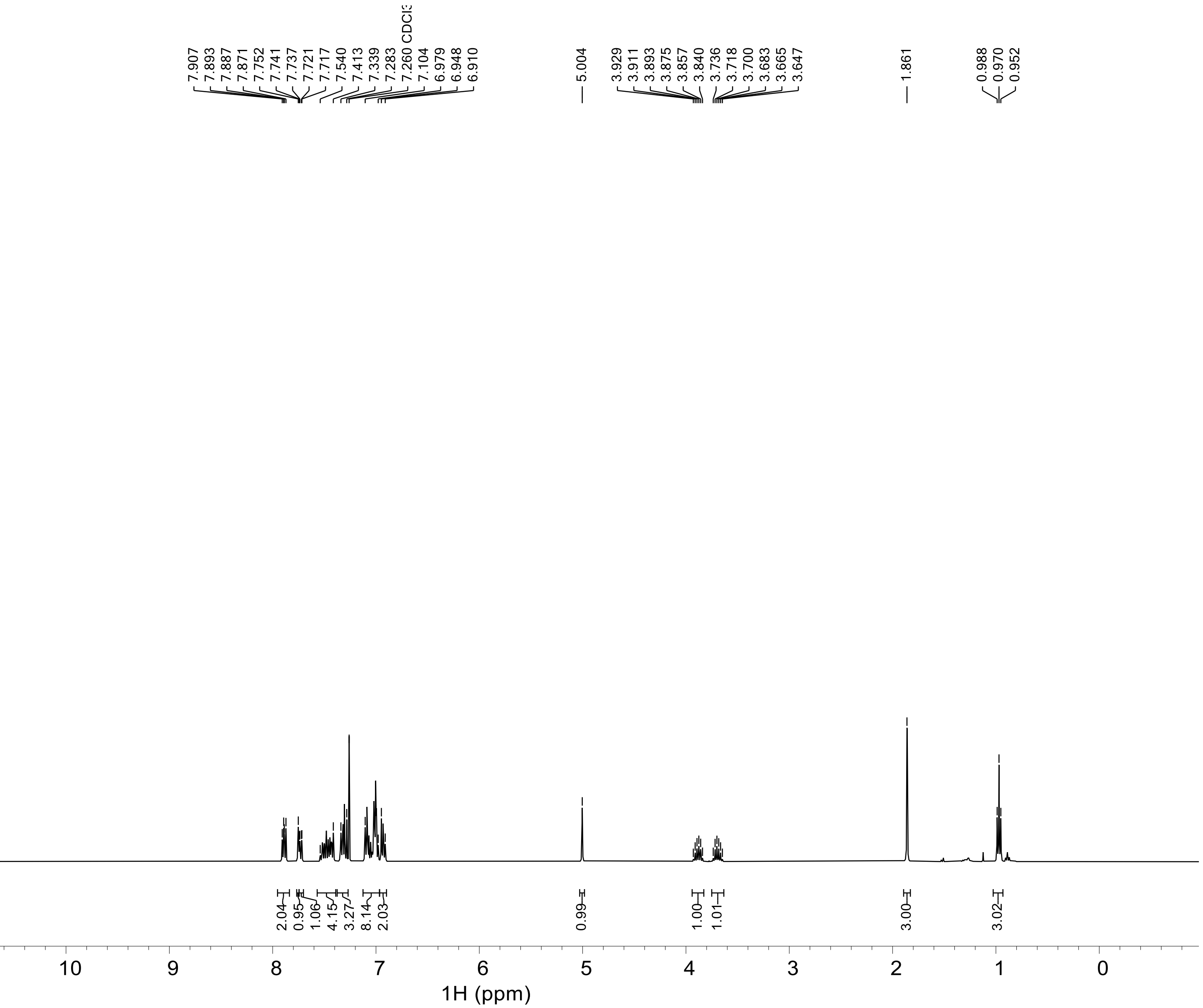

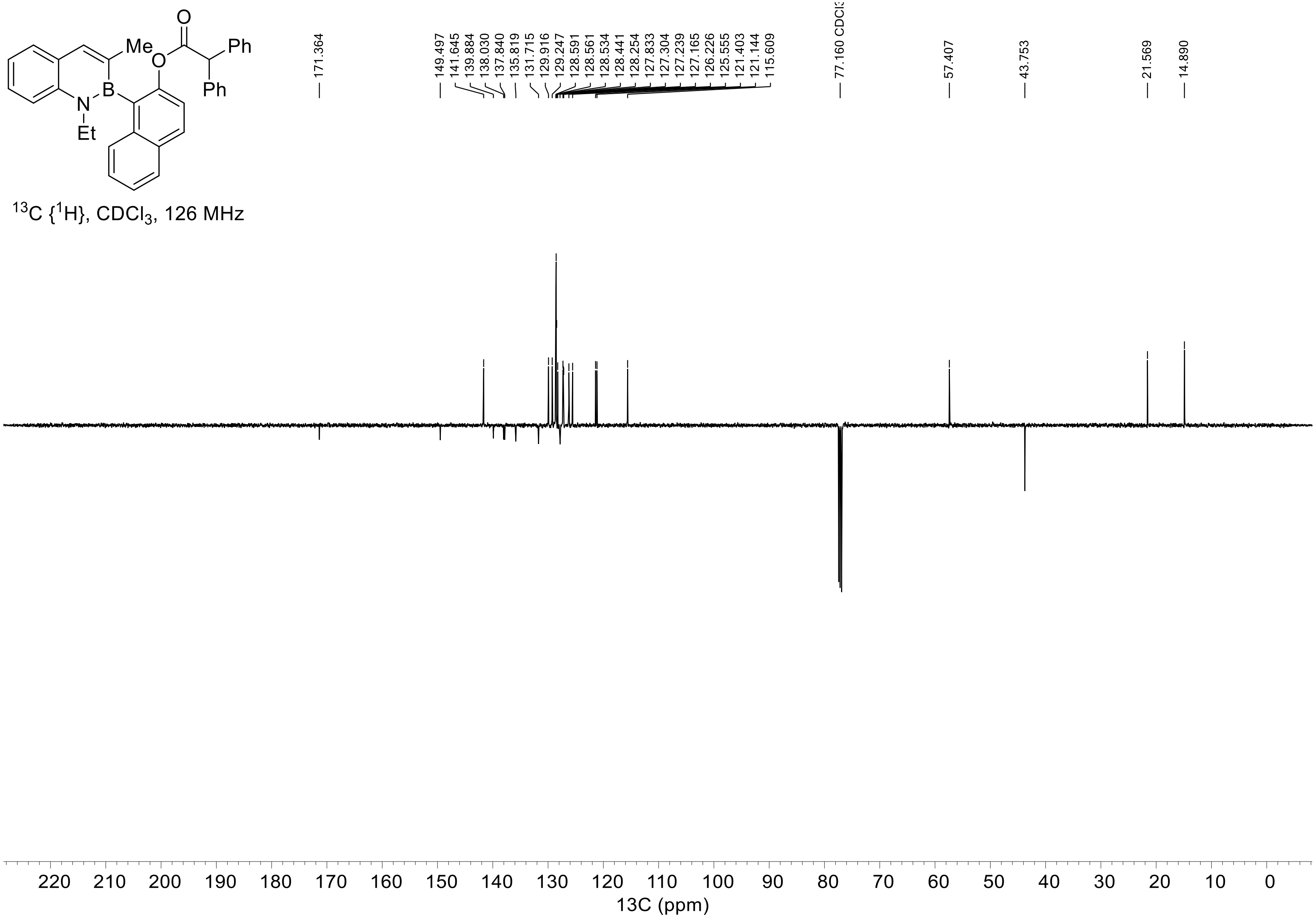

$^{13}C\{^1H\}$, $CDCl_3$, 126 MHz

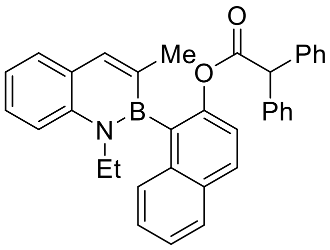

^{11}B {^{1}H}, $CDCl_3$, 128 MHz

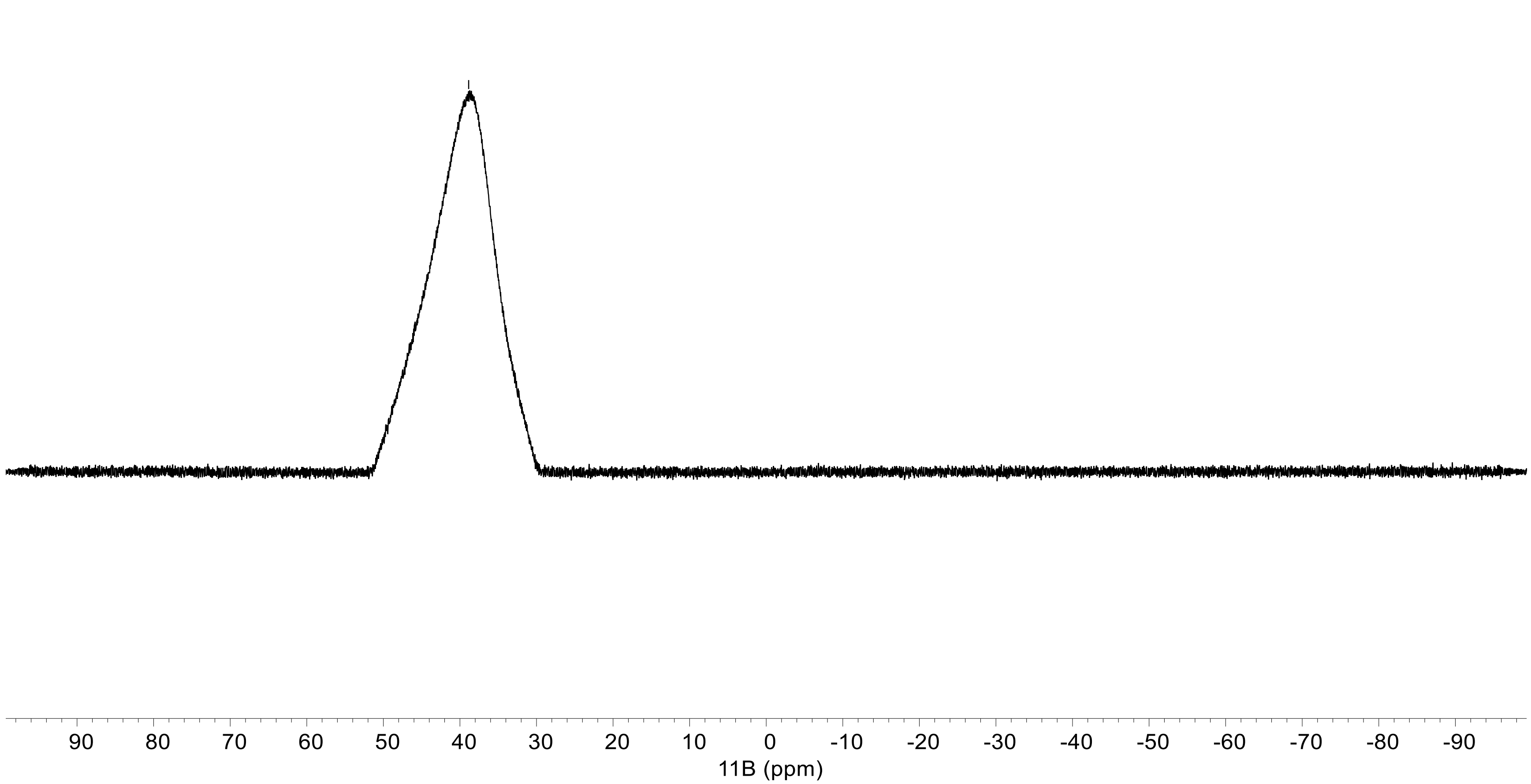

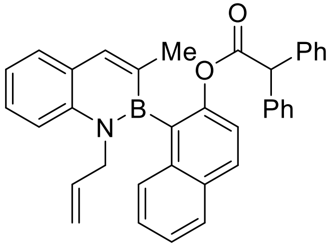

^{1}H, $CDCl_3$, 400 MHz

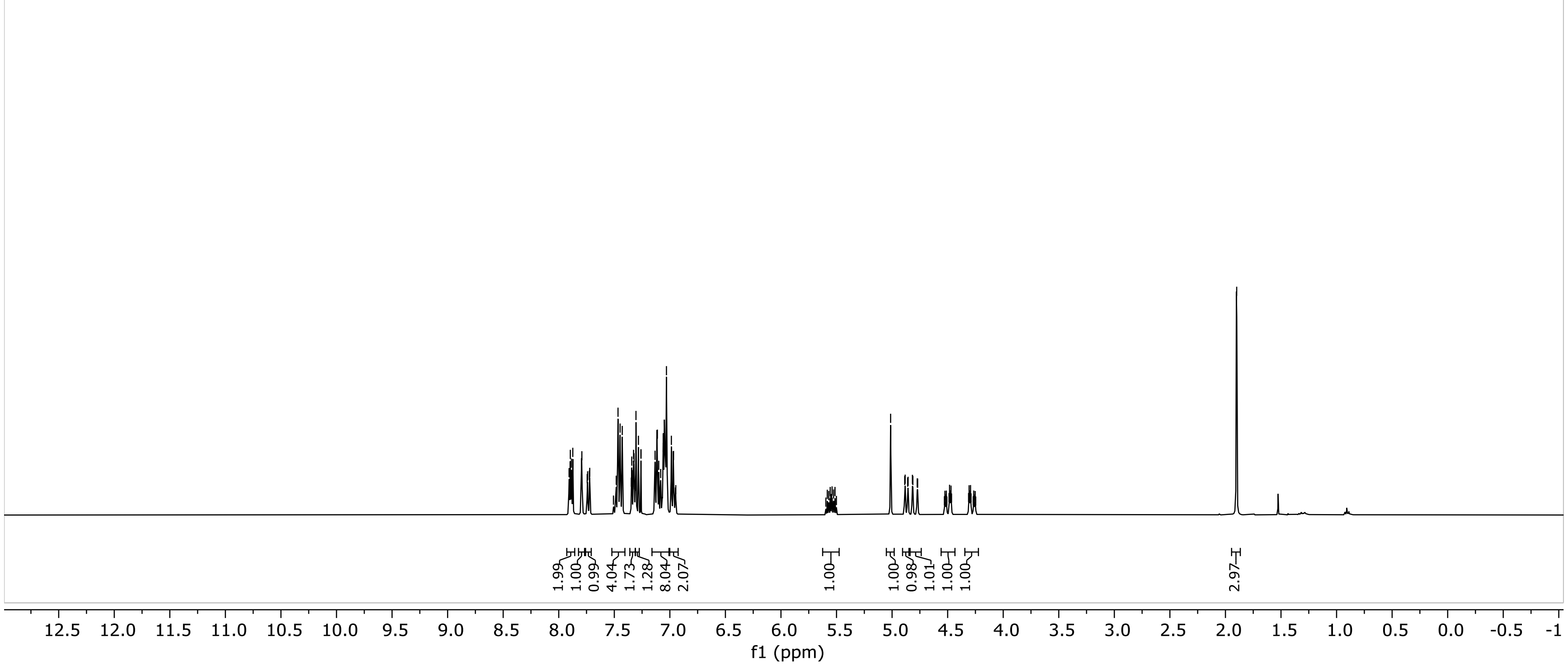

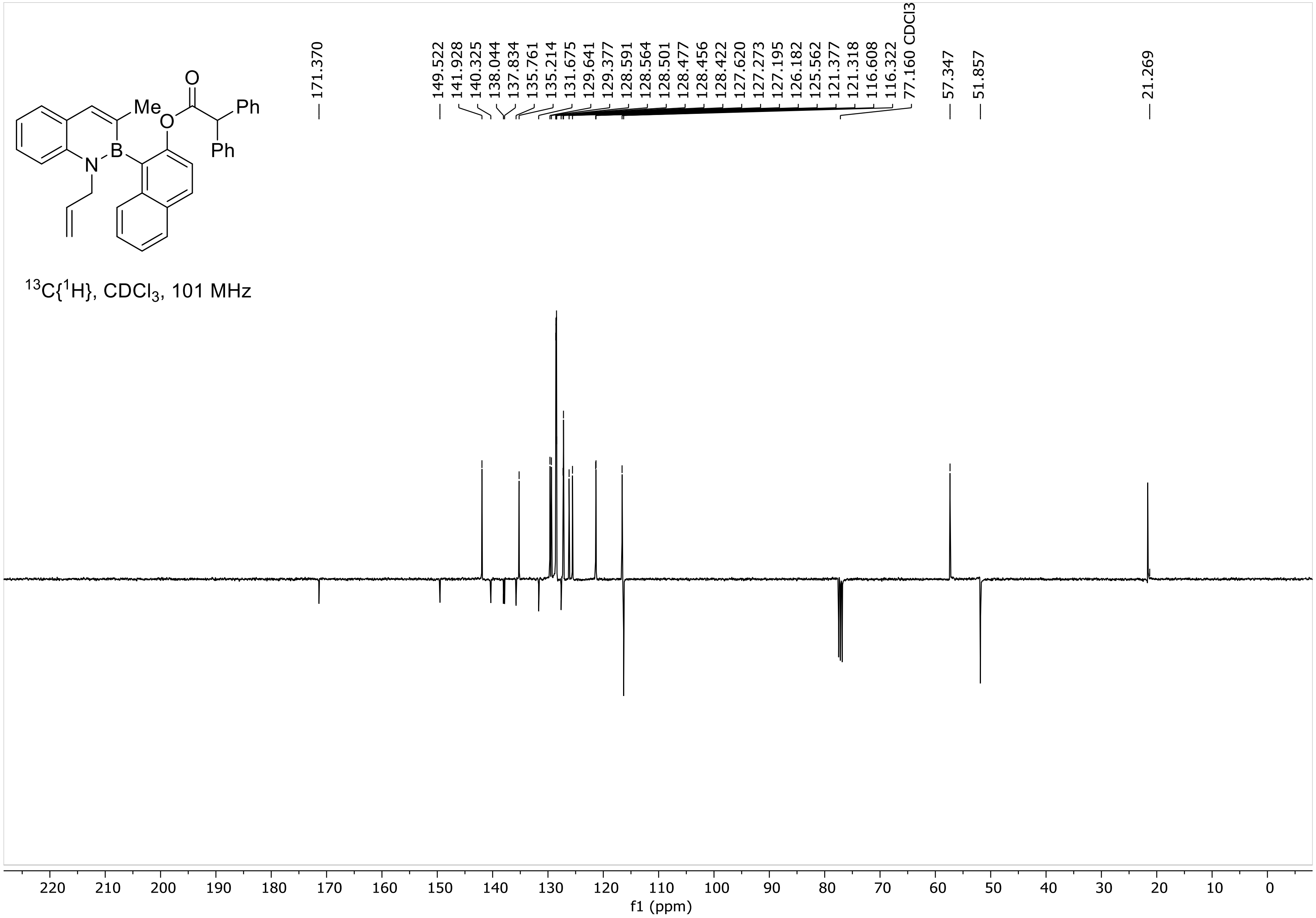

Me
O
Ph
Ph
N
B
O
^{13}C{^{1}H}, CDCl$_3$, 101 MHz
171.370
149.522
141.928
140.325
138.044
137.834
135.761
135.214
131.675
129.641
129.377
128.591
128.564
128.501
128.477
128.456
128.422
127.620
127.273
127.195
126.182
125.562
121.377
121.318
116.608
116.322
77.160 CDCl3
57.347
51.857
21.269
220 210 200 190 180 170 160 150 140 130 120 110 100 90 80 70 60 50 40 30 20 10 0
f1 (ppm)

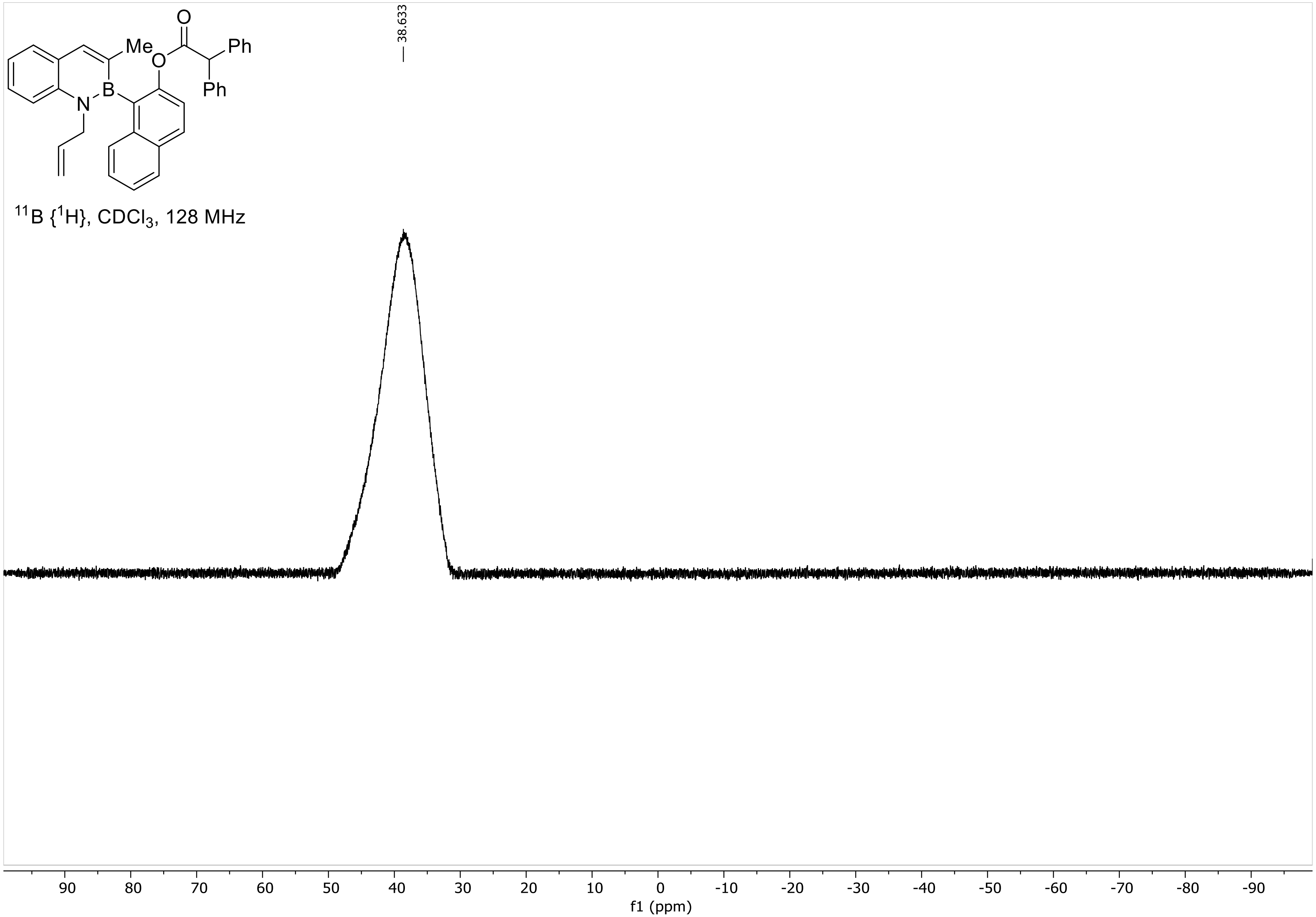
38.633
O
Me
O
Ph
Ph
N
B
$^{11}B\ \{^{1}H\}$, $CDCl_3$, 128 MHz
90 80 70 60 50 40 30 20 10 0 -10 -20 -30 -40 -50 -60 -70 -80 -90
f1 (ppm)

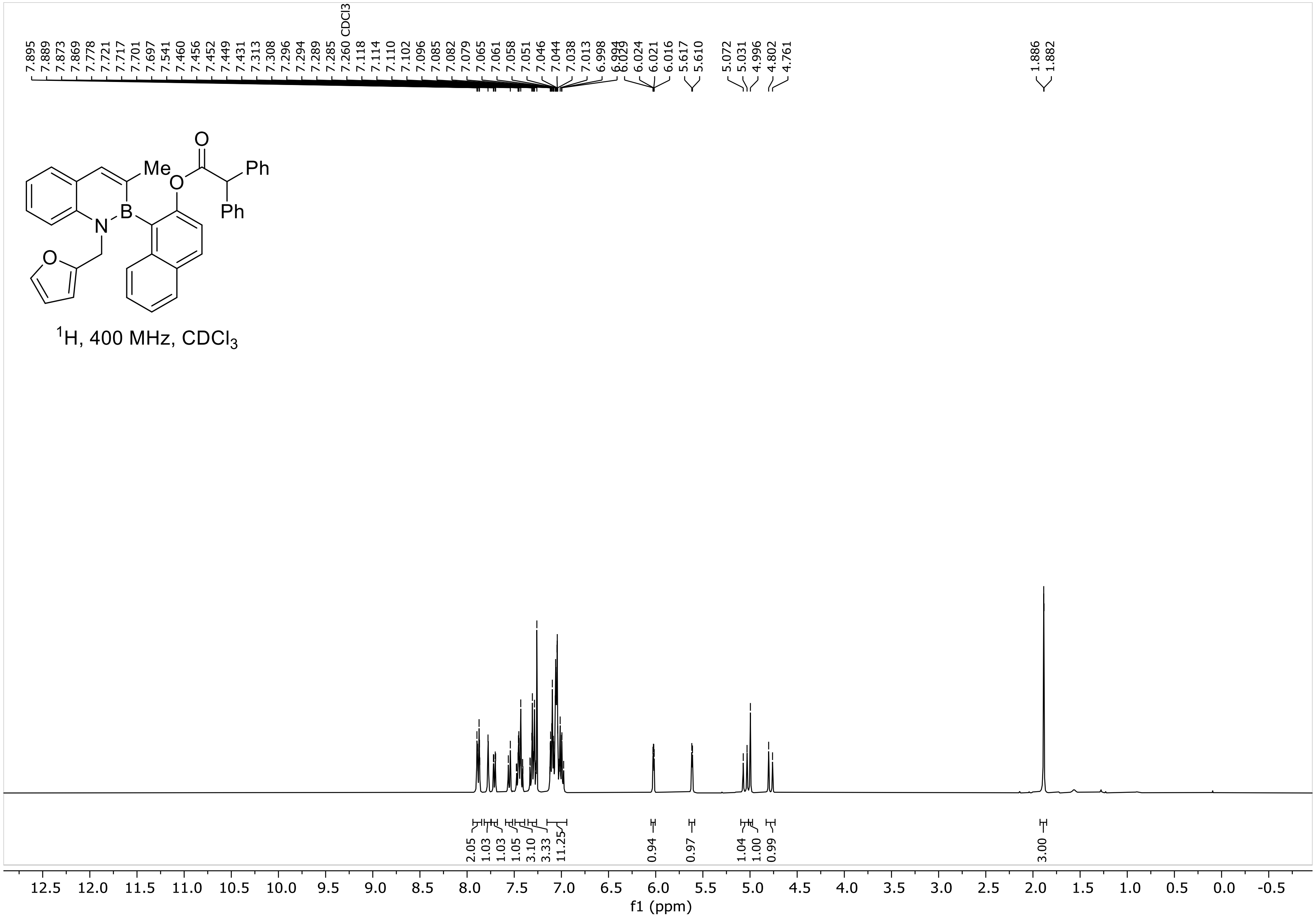
Me
O
O
Ph
Ph
B
N
O
^{1}H, 400 MHz, CDCl$_3$
7.895 7.889 7.873 7.869 7.778 7.721 7.717 7.701 7.697 7.541 7.460 7.456 7.452 7.449 7.431 7.313 7.308 7.296 7.294 7.289 7.285 7.260 CDCl3 7.118 7.114 7.110 7.102 7.096 7.085 7.082 7.079 7.065 7.061 7.058 7.051 7.046 7.044 7.038 7.013 6.998 6.994 6.029 6.024 6.021 6.016 5.617 5.610 5.072 5.031 4.996 4.802 4.761 1.886 1.882
2.05 1.03 1.03 1.05 3.10 3.33 11.25 0.94 0.97 1.04 1.00 0.99 3.00
12.5 12.0 11.5 11.0 10.5 10.0 9.5 9.0 8.5 8.0 7.5 7.0 6.5 6.0 5.5 5.0 4.5 4.0 3.5 3.0 2.5 2.0 1.5 1.0 0.5 0.0 -0.5
f1 (ppm)

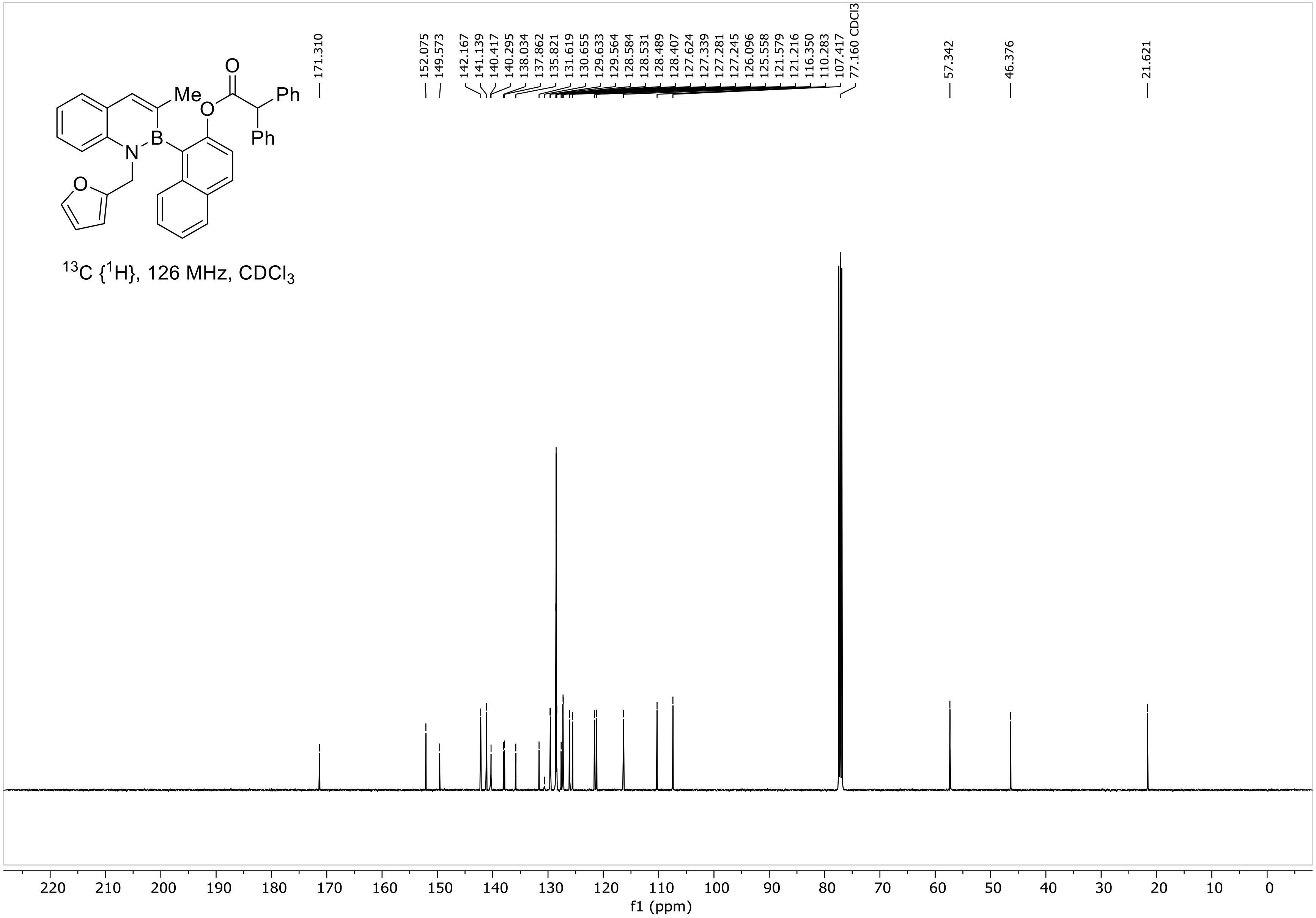

Me
O
O
Ph
Ph
B
N
O
$^{13}C\ \{^{1}H\}$, 126 MHz, $CDCl_3$
171.310
152.075
149.573
142.167
141.139
140.417
140.295
138.034
137.862
135.821
131.619
130.655
129.633
129.564
128.584
128.531
128.489
128.407
127.624
127.339
127.281
127.245
126.096
125.558
121.579
121.216
116.350
110.283
107.417
77.160 CDCl3
57.342
46.376
21.621
220 210 200 190 180 170 160 150 140 130 120 110 100 90 80 70 60 50 40 30 20 10 0
f1 (ppm)

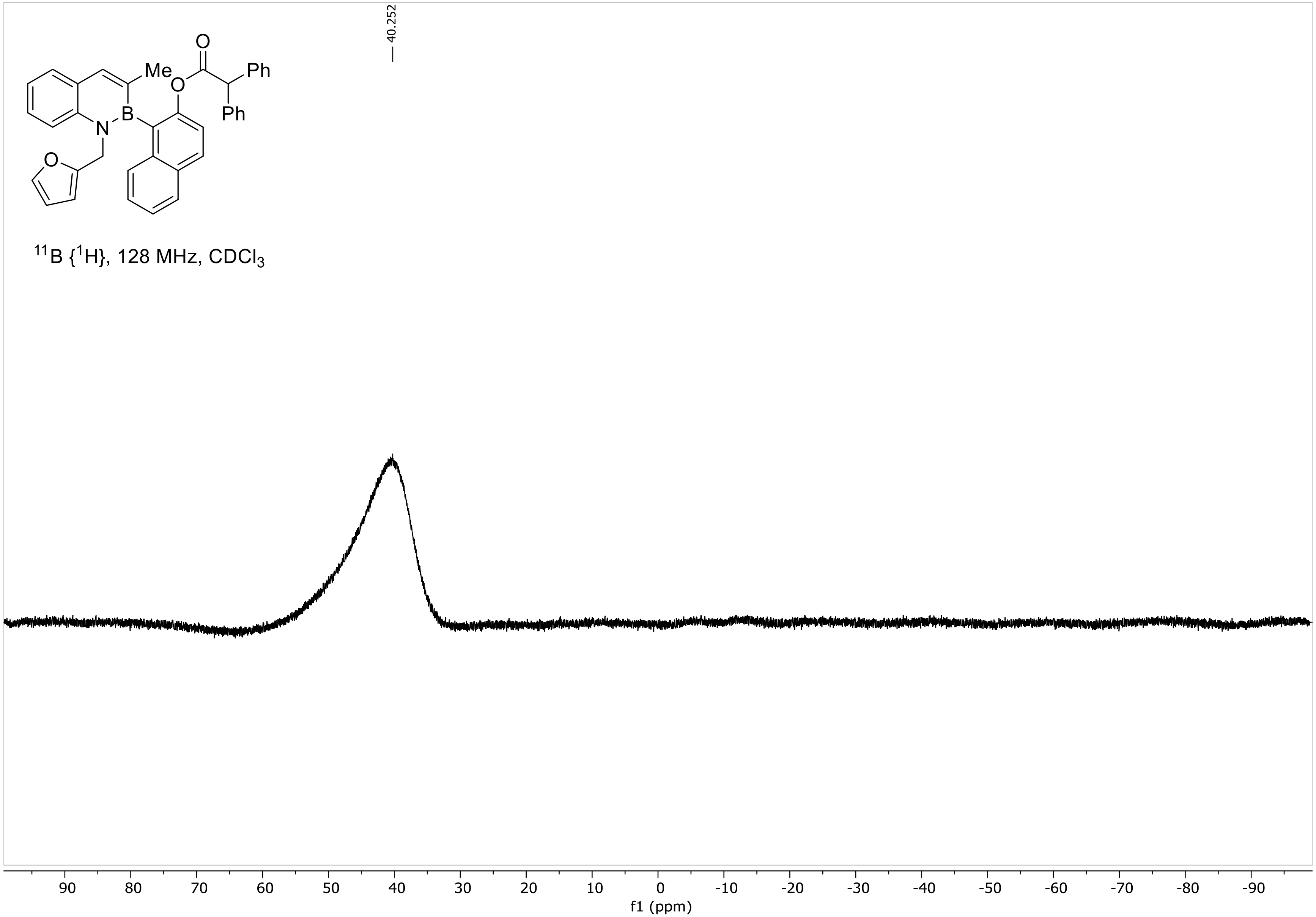
40.252
Me
O
O
Ph
Ph
B
N
O
11B {1H}, 128 MHz, CDCl3
90 80 70 60 50 40 30 20 10 0 -10 -20 -30 -40 -50 -60 -70 -80 -90
f1 (ppm)

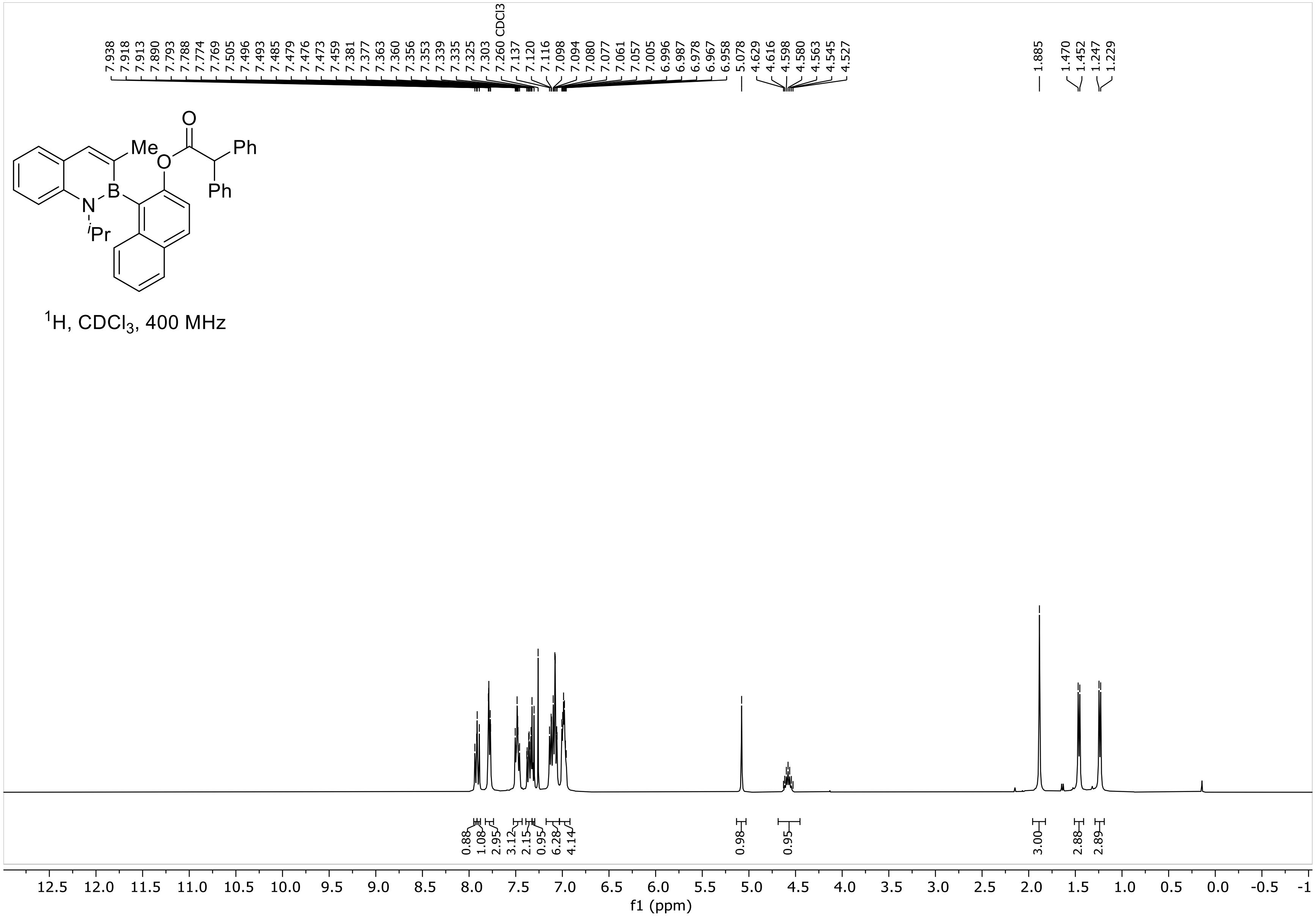

7.938 7.918 7.913 7.890 7.793 7.788 7.774 7.769 7.505 7.496 7.493 7.485 7.479 7.476 7.473 7.459 7.381 7.377 7.363 7.360 7.356 7.353 7.339 7.335 7.325 7.303 7.260 CDCl3 7.137 7.120 7.116 7.098 7.094 7.080 7.077 7.061 7.057 7.005 6.996 6.987 6.978 6.967 6.958 5.078 4.629 4.616 4.598 4.580 4.563 4.545 4.527
1.885 1.470 1.452 1.247 1.229
Me
O
O
Ph
Ph
N
B
iPr
^{1}H, $CDCl_3$, 400 MHz
0.88 1.08 2.95 3.12 2.15 0.95 6.28 4.14 0.98 0.95 3.00 2.88 2.89
12.5 12.0 11.5 11.0 10.5 10.0 9.5 9.0 8.5 8.0 7.5 7.0 6.5 6.0 5.5 5.0 4.5 4.0 3.5 3.0 2.5 2.0 1.5 1.0 0.5 0.0 -0.5 -1
f1 (ppm)

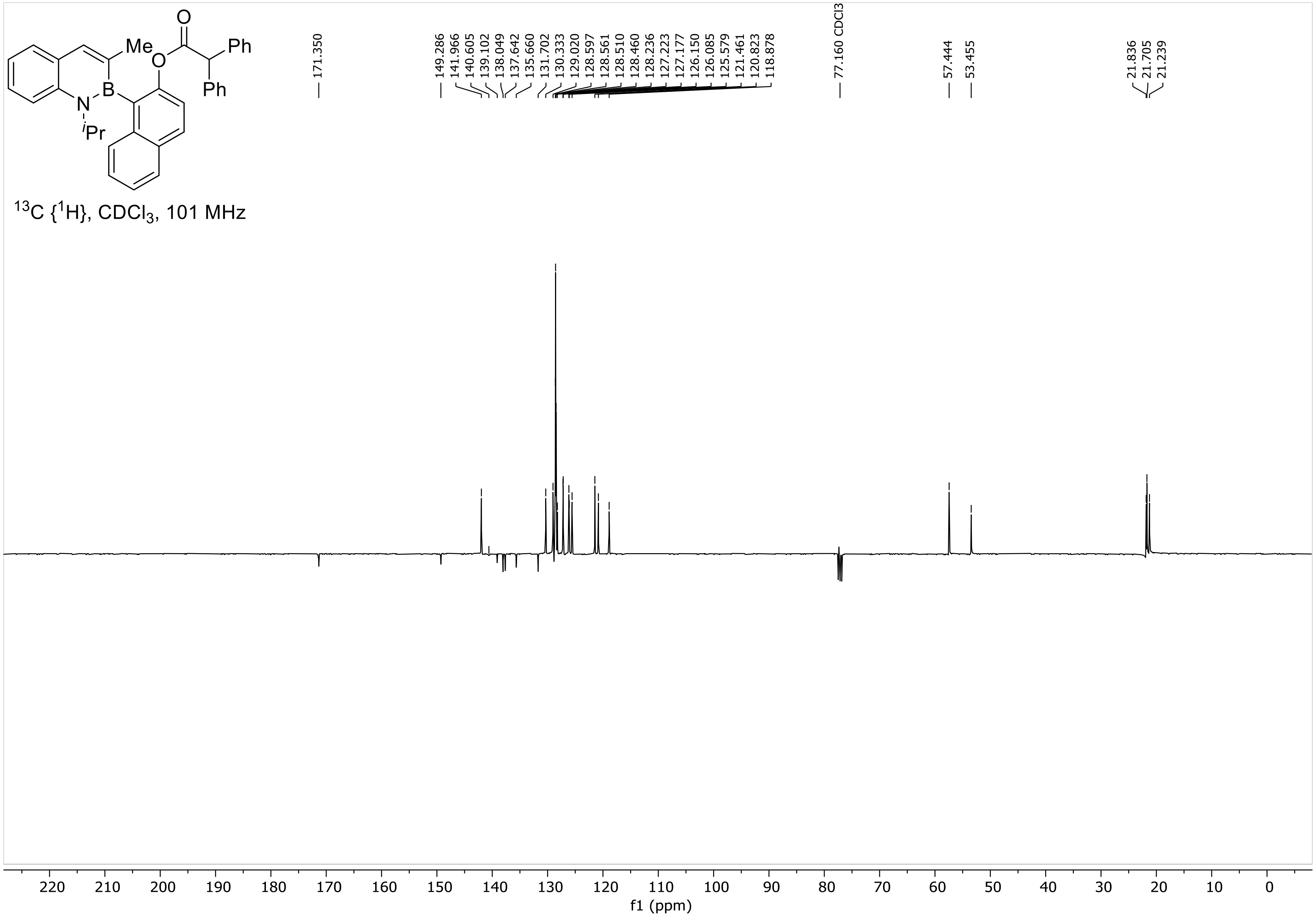
Me
O
O
Ph
Ph
N
B
iPr
13C {1H}, CDCl3, 101 MHz
171.350
149.286
141.966
140.605
139.102
138.049
137.642
135.660
131.702
130.333
129.020
128.597
128.561
128.510
128.460
128.236
127.223
127.177
126.150
126.085
125.579
121.461
120.823
118.878
77.160 CDCl3
57.444
53.455
21.836
21.705
21.239
220 210 200 190 180 170 160 150 140 130 120 110 100 90 80 70 60 50 40 30 20 10 0
f1 (ppm)

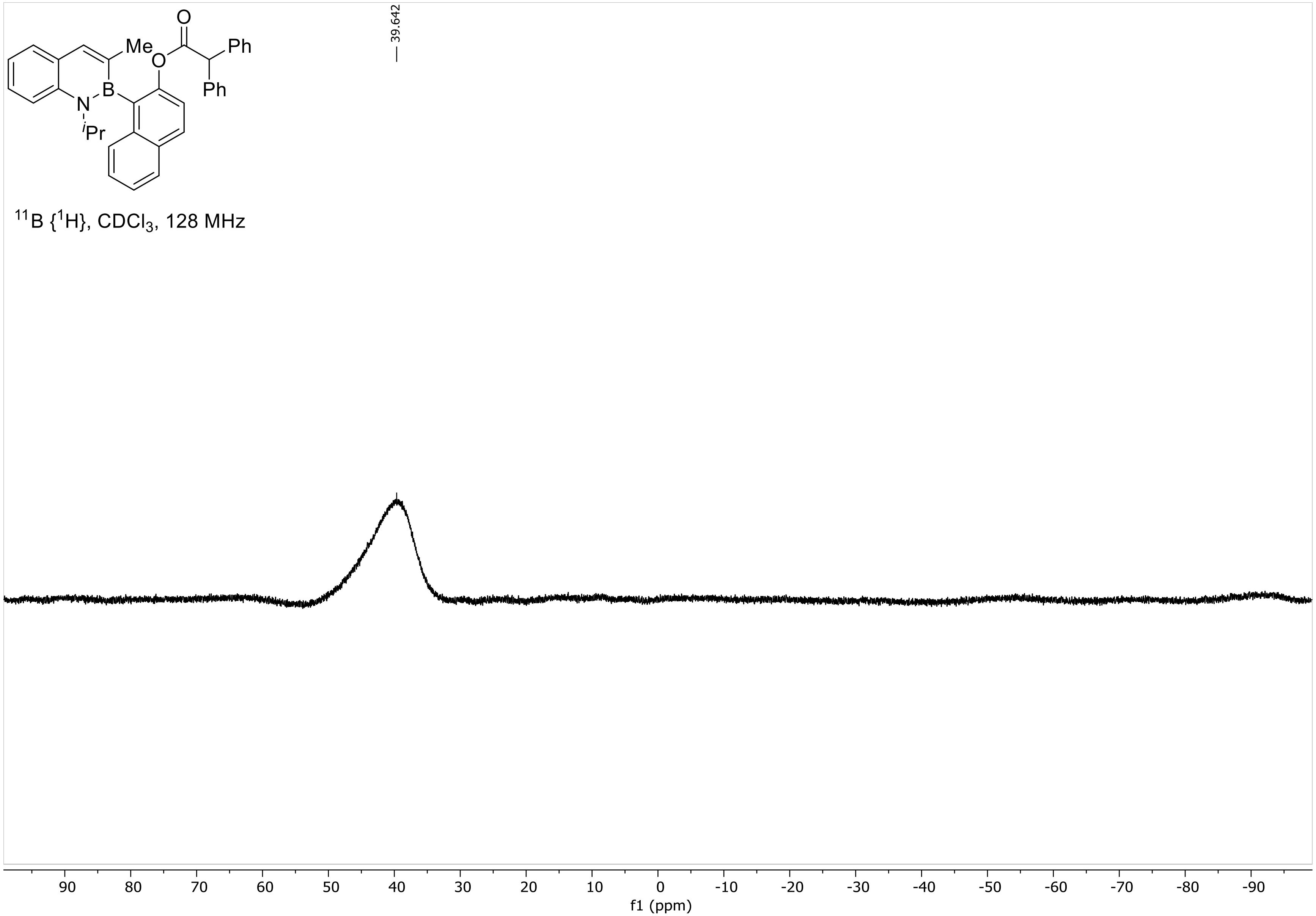
39.642
Me
O
O
Ph
Ph
B
N
iPr
11B {1H}, CDCl3, 128 MHz
90 80 70 60 50 40 30 20 10 0 -10 -20 -30 -40 -50 -60 -70 -80 -90
f1 (ppm)

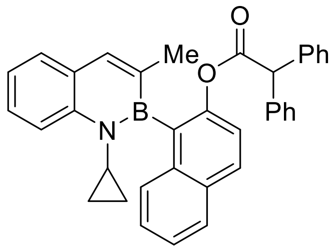

^1H, $CDCl_3$, 400 MHz

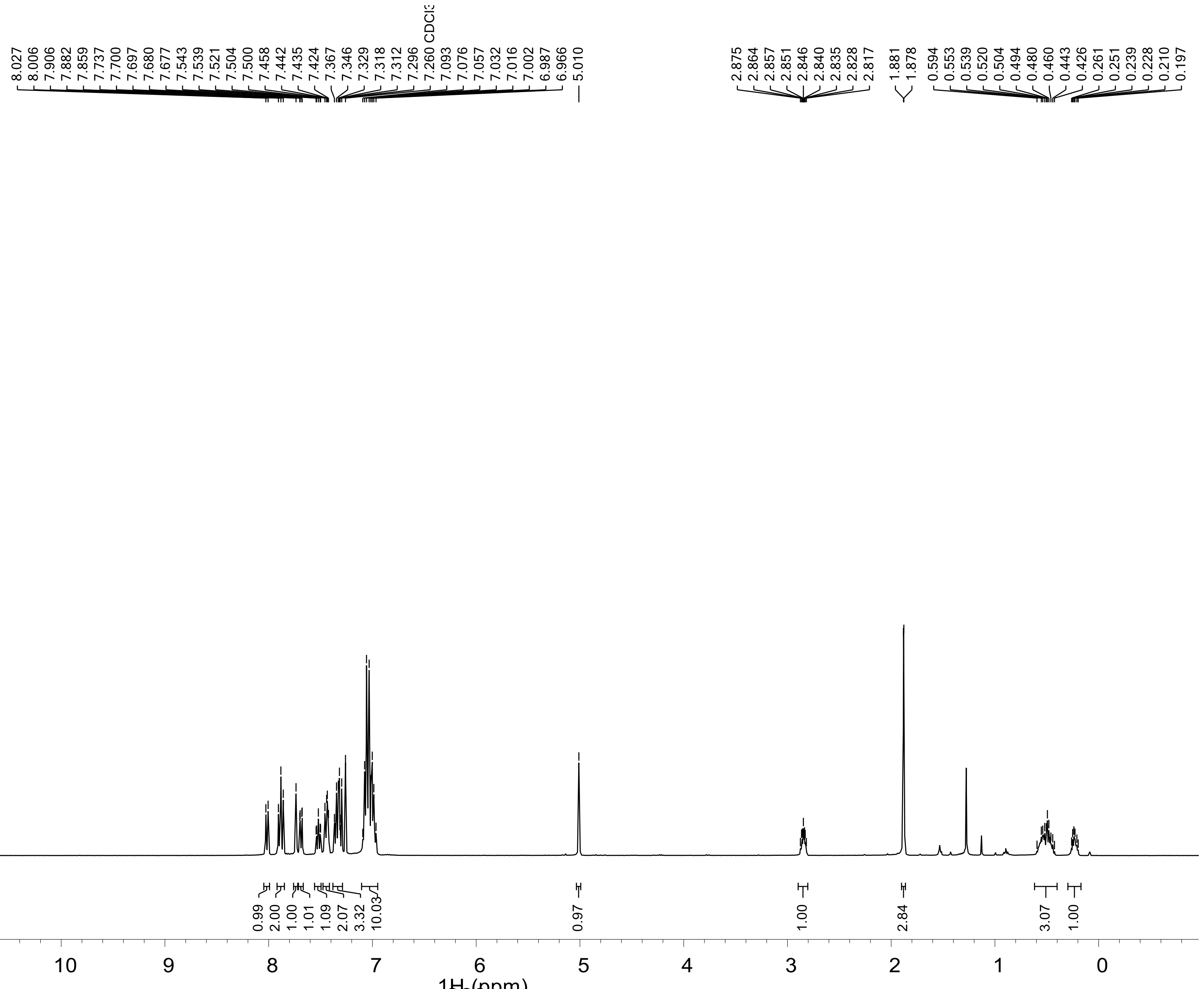

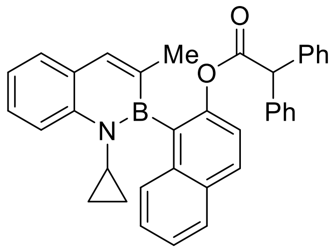

^{13}C {^{1}H}, $CDCl_3$, 101 MHz

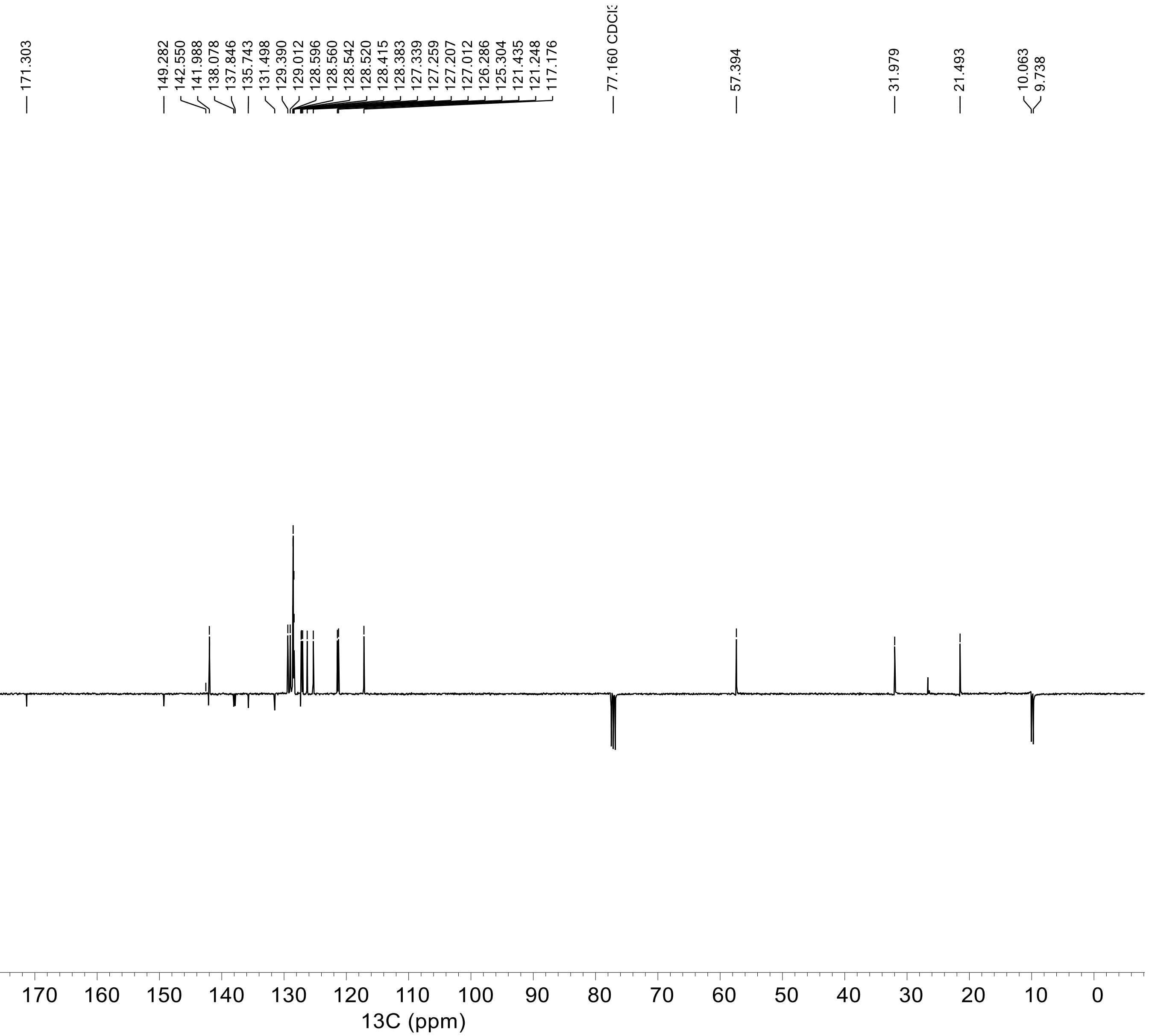

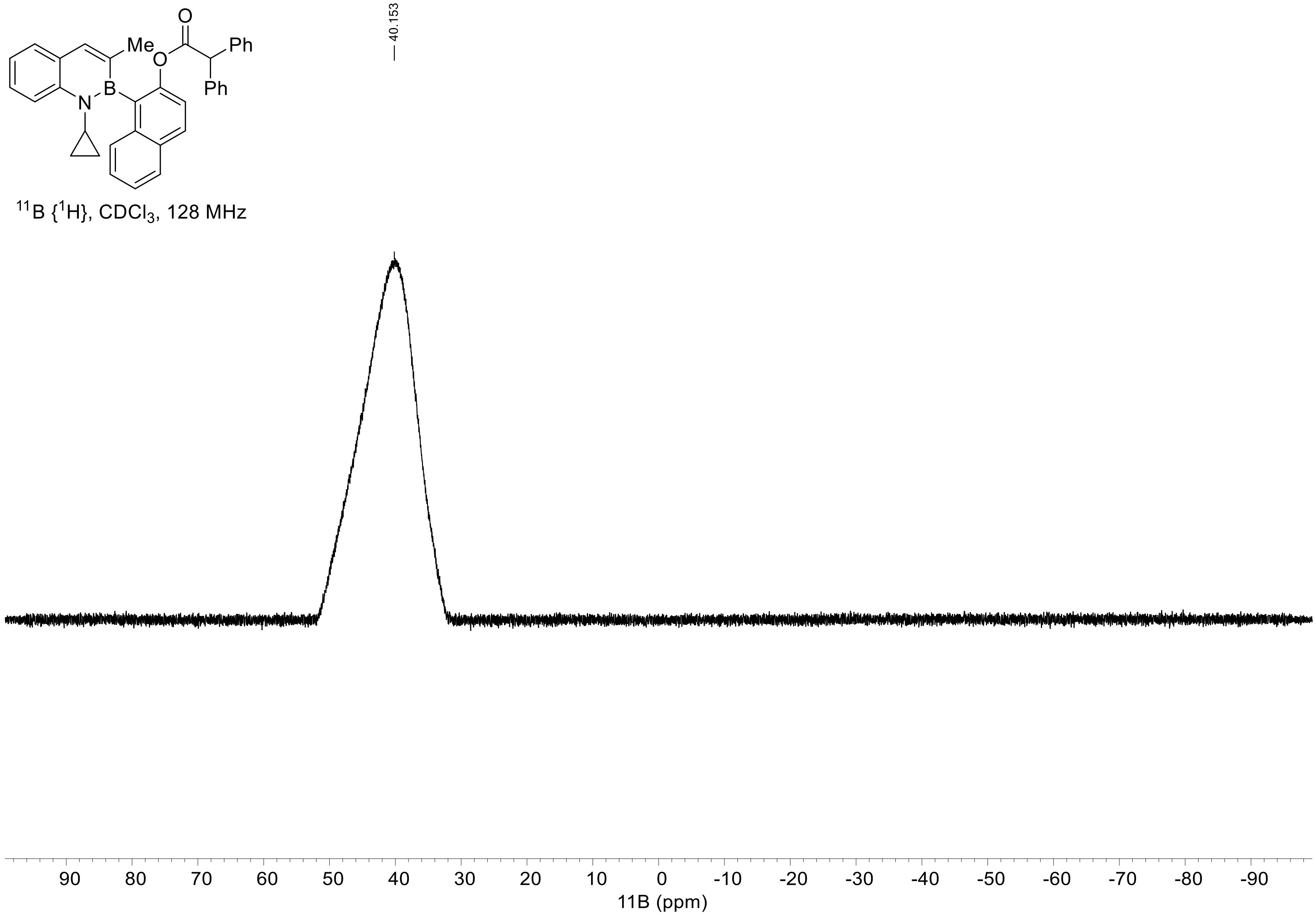
O
Me
O
Ph
B
N
Ph
^{11}B {^{1}H}, $CDCl_3$, 128 MHz
40.153
90 80 70 60 50 40 30 20 10 0 -10 -20 -30 -40 -50 -60 -70 -80 -90
11B (ppm)

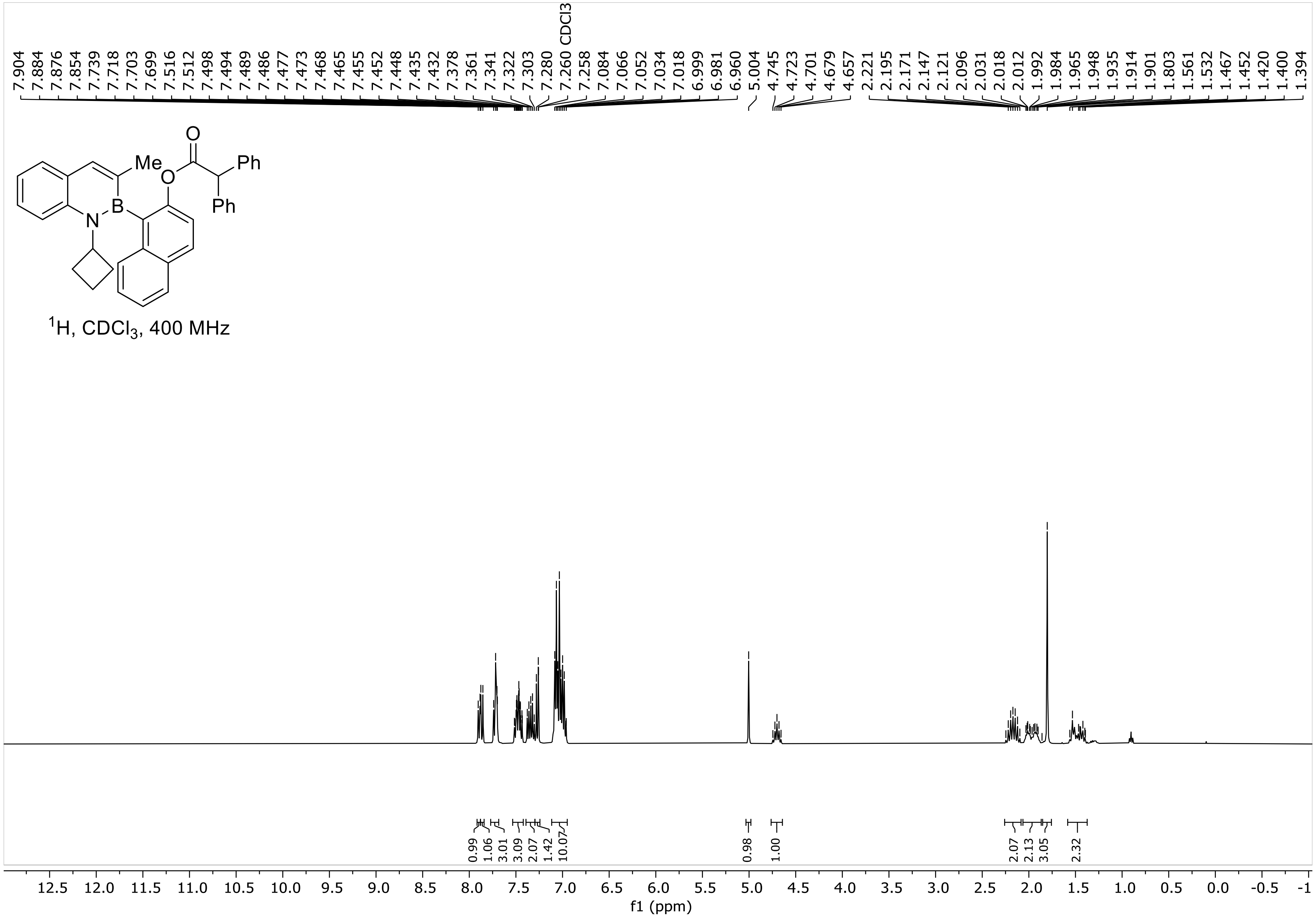
7.904 7.884 7.876 7.854 7.739 7.718 7.703 7.699 7.516 7.512 7.498 7.494 7.489 7.486 7.477 7.473 7.468 7.465 7.455 7.452 7.448 7.435 7.432 7.378 7.361 7.341 7.322 7.303 7.280 7.260 CDCl3 7.258 7.084 7.066 7.052 7.034 7.018 6.999 6.981 6.960 5.004 4.745 4.723 4.701 4.679 4.657 2.221 2.195 2.171 2.147 2.121 2.096 2.031 2.018 2.012 1.992 1.984 1.965 1.948 1.935 1.914 1.901 1.803 1.561 1.532 1.467 1.452 1.420 1.400 1.394
O
Me
O
Ph
Ph
B
N
^{1}H, $CDCl_3$, 400 MHz
0.99 1.06 3.01 3.09 2.07 1.42 10.07 0.98 1.00 2.07 2.13 3.05 2.32
12.5 12.0 11.5 11.0 10.5 10.0 9.5 9.0 8.5 8.0 7.5 7.0 6.5 6.0 5.5 5.0 4.5 4.0 3.5 3.0 2.5 2.0 1.5 1.0 0.5 0.0 -0.5 -1
f1 (ppm)

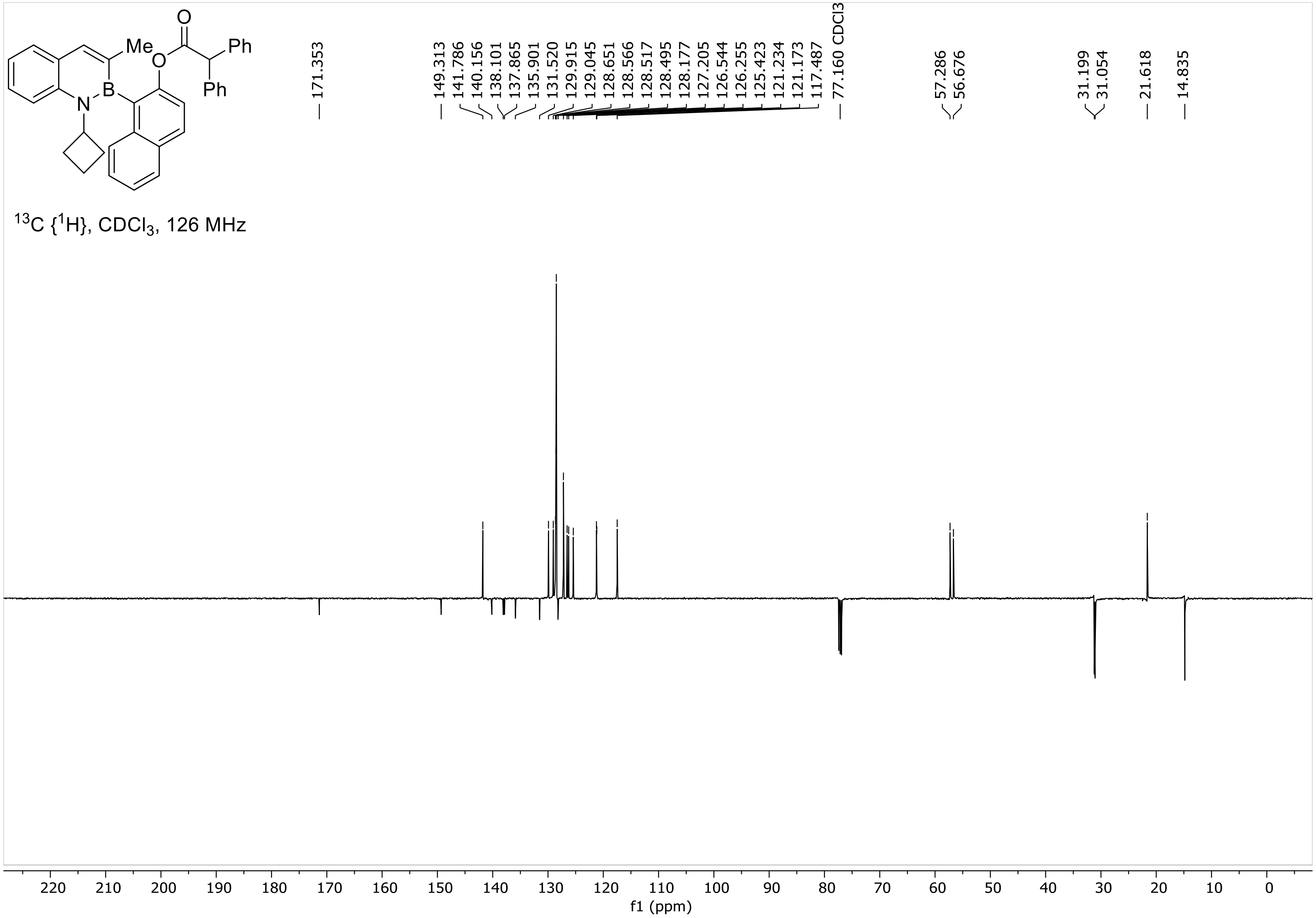

Me
O
O
Ph
Ph
B
N
13C {1H}, CDCl3, 126 MHz
171.353
149.313
141.786
140.156
138.101
137.865
135.901
131.520
129.915
129.045
128.651
128.566
128.517
128.495
128.177
127.205
126.544
126.255
125.423
121.234
121.173
117.487
77.160 CDCl3
57.286
56.676
31.199
31.054
21.618
14.835
220 210 200 190 180 170 160 150 140 130 120 110 100 90 80 70 60 50 40 30 20 10 0
f1 (ppm)

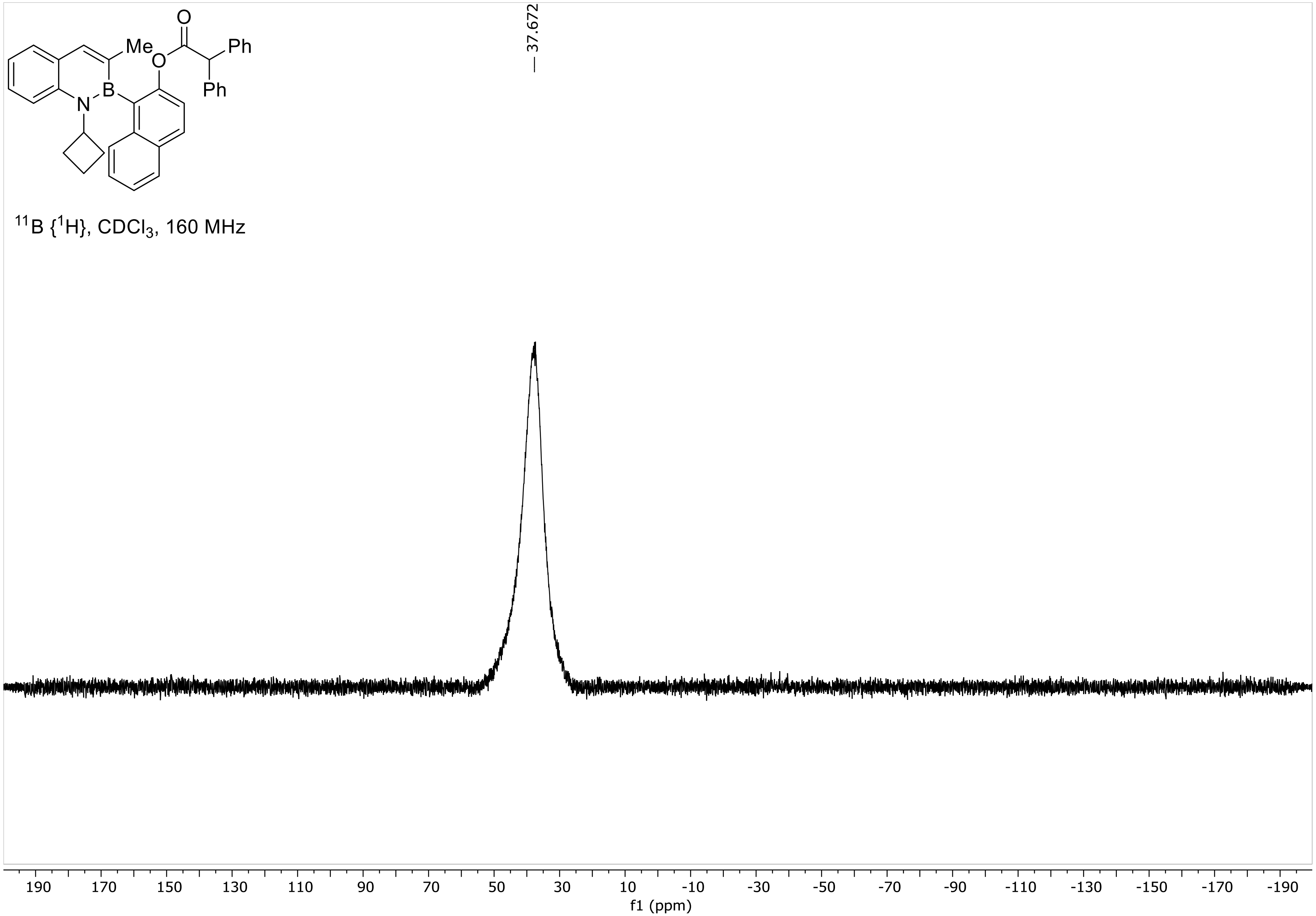
Me
O
O
Ph
Ph
N
B
11B {1H}, CDCl3, 160 MHz
— 37.672
190
170
150
130
110
90
70
50
30
10
-10
-30
-50
-70
-90
-110
-130
-150
-170
-190
f1 (ppm)

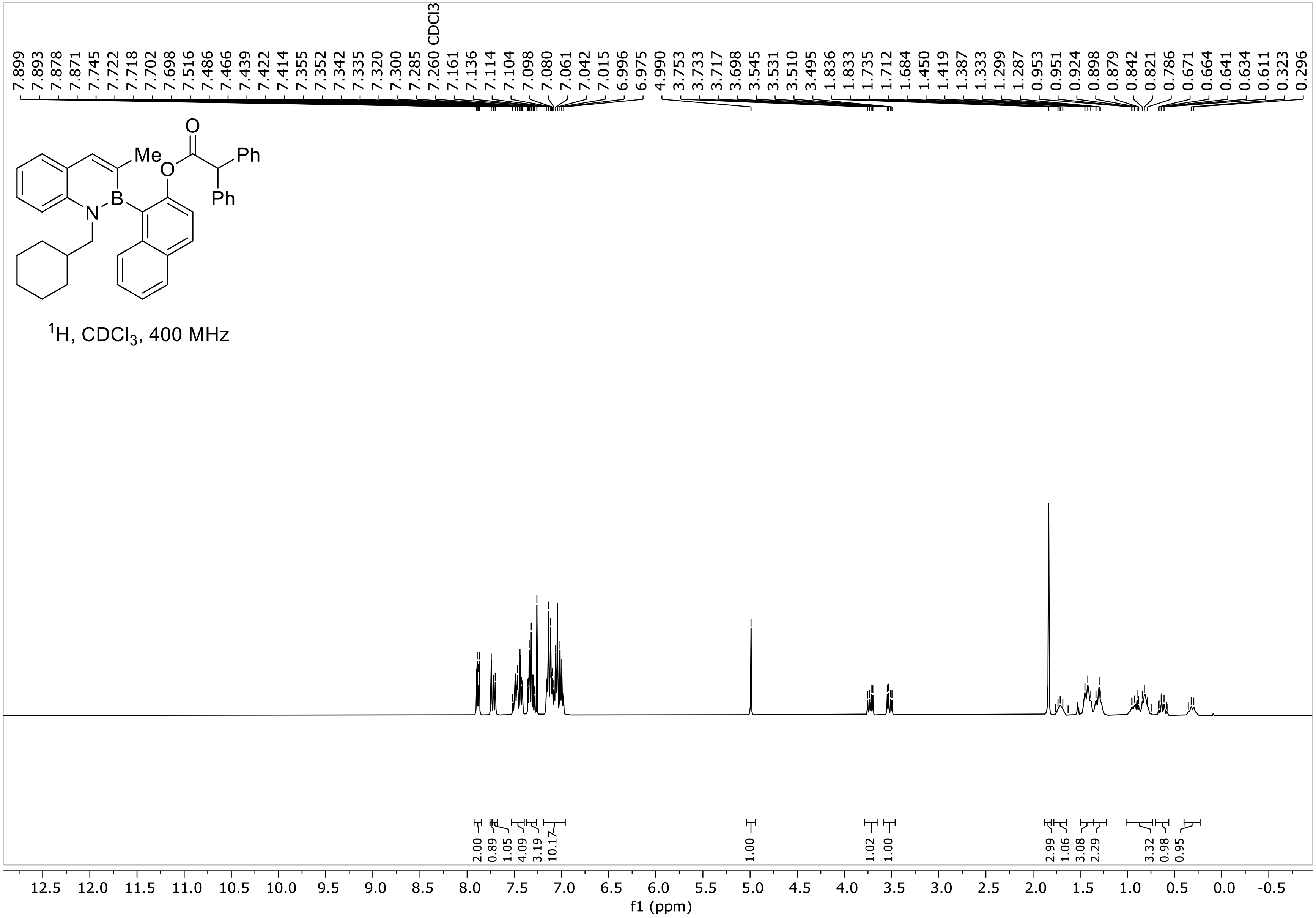
7.899 7.893 7.878 7.871 7.745 7.722 7.718 7.702 7.698 7.516 7.486 7.466 7.439 7.422 7.414 7.355 7.352 7.342 7.335 7.320 7.300 7.285 7.260 CDCl3 7.161 7.136 7.114 7.104 7.098 7.080 7.061 7.042 7.015 6.996 6.975 4.990 3.753 3.733 3.717 3.698 3.545 3.531 3.510 3.495 1.836 1.833 1.735 1.712 1.684 1.450 1.419 1.387 1.333 1.299 1.287 0.953 0.951 0.924 0.898 0.879 0.842 0.821 0.786 0.671 0.664 0.641 0.634 0.611 0.323 0.296
Me
O
O
Ph
Ph
N
B
1H, CDCl3, 400 MHz
2.00 0.89 1.05 4.09 3.19 10.17 1.00 1.02 1.00 2.99 1.06 3.08 2.29 3.32 0.98 0.95
12.5 12.0 11.5 11.0 10.5 10.0 9.5 9.0 8.5 8.0 7.5 7.0 6.5 6.0 5.5 5.0 4.5 4.0 3.5 3.0 2.5 2.0 1.5 1.0 0.5 0.0 -0.5
f1 (ppm)

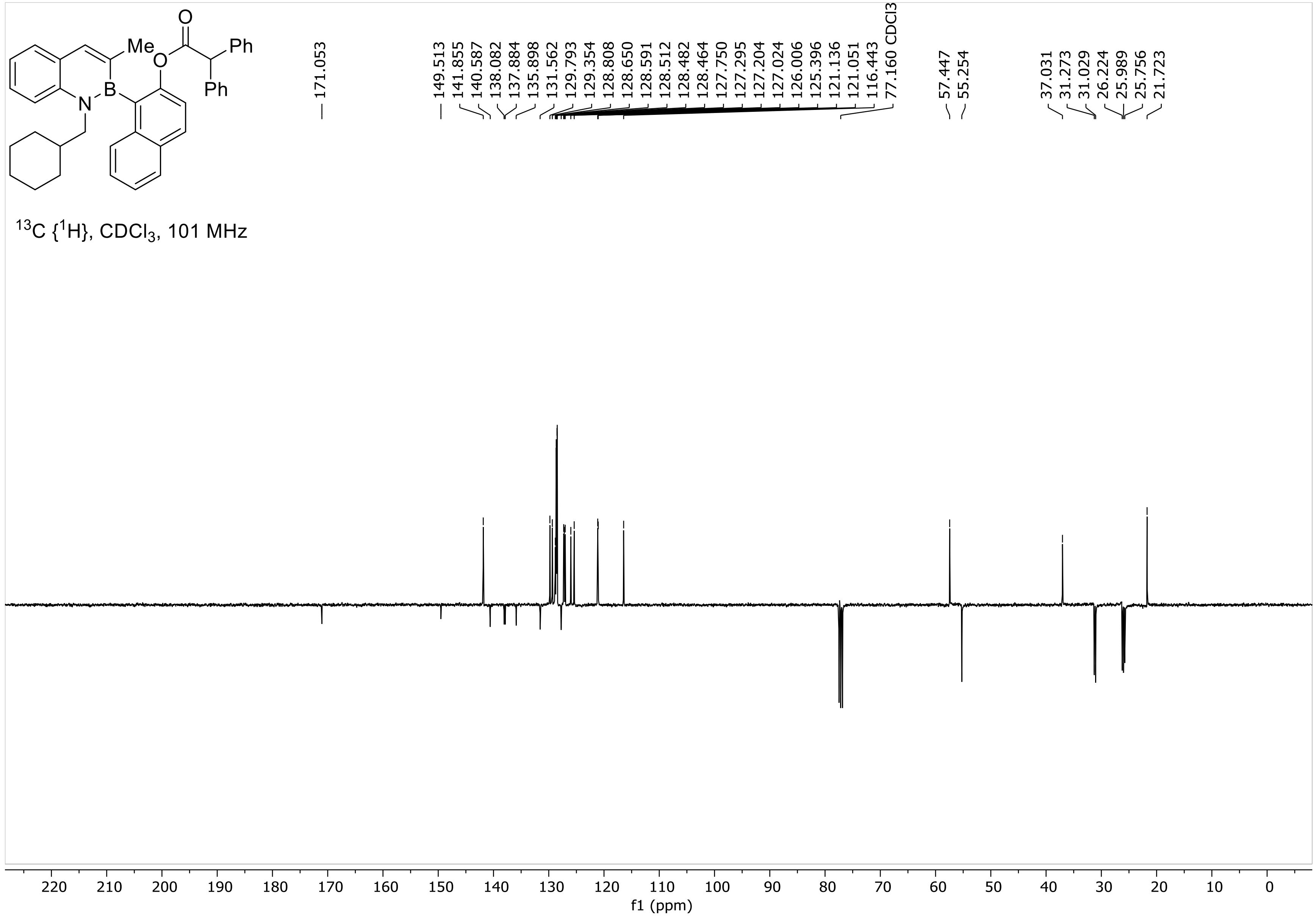
Me
O
O
Ph
Ph
N
B
13C {1H}, CDCl3, 101 MHz
171.053
149.513
141.855
140.587
138.082
137.884
135.898
131.562
129.793
129.354
128.808
128.650
128.591
128.512
128.482
128.464
127.750
127.295
127.204
127.024
126.006
125.396
121.136
121.051
116.443
77.160 CDCl3
57.447
55.254
37.031
31.273
31.029
26.224
25.989
25.756
21.723
220 210 200 190 180 170 160 150 140 130 120 110 100 90 80 70 60 50 40 30 20 10 0
f1 (ppm)

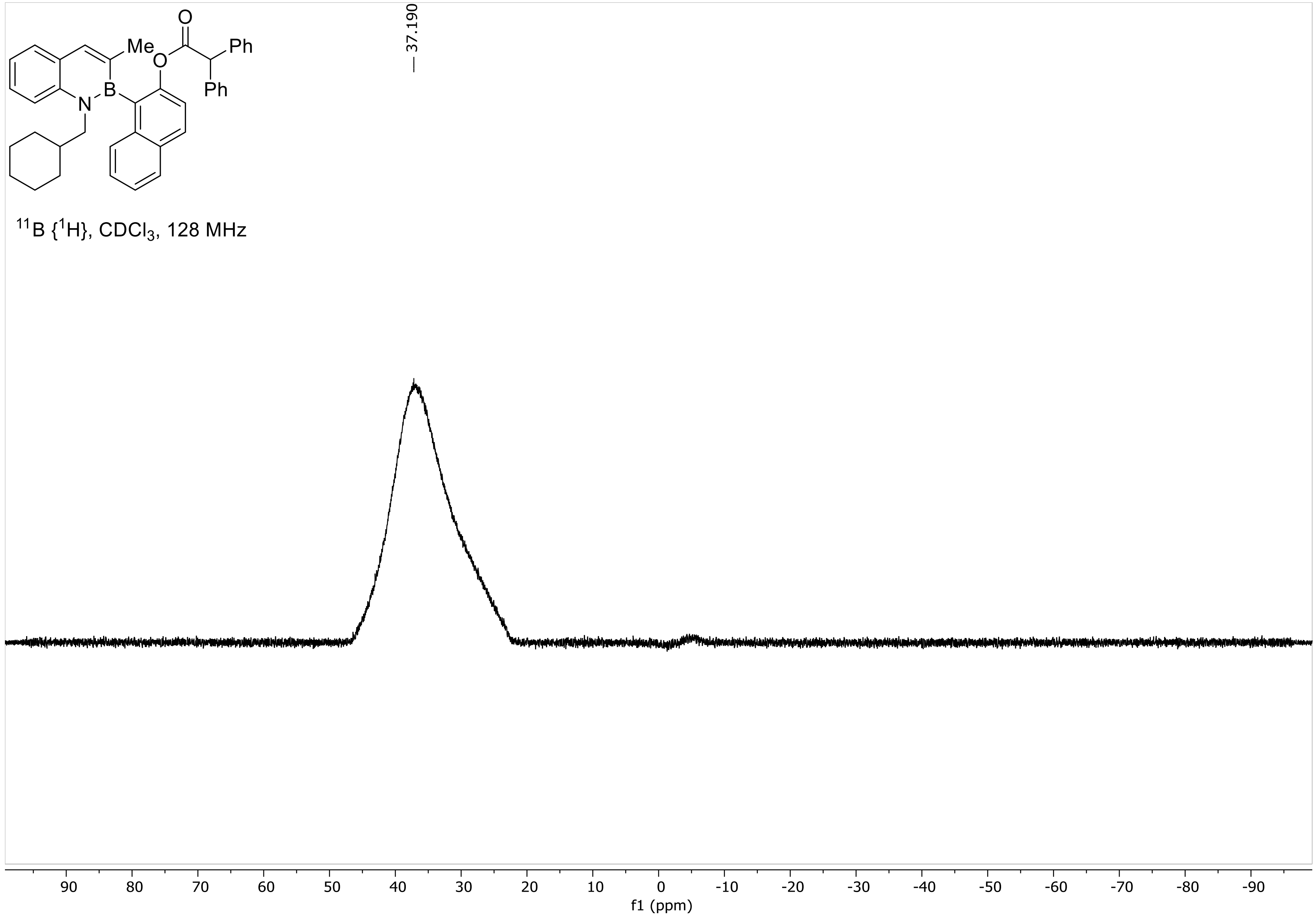

Me
O
O
Ph
Ph
N
B
37.190
11B {1H}, CDCl3, 128 MHz
90
80
70
60
50
40
30
20
10
0
-10
-20
-30
-40
-50
-60
-70
-80
-90
f1 (ppm)

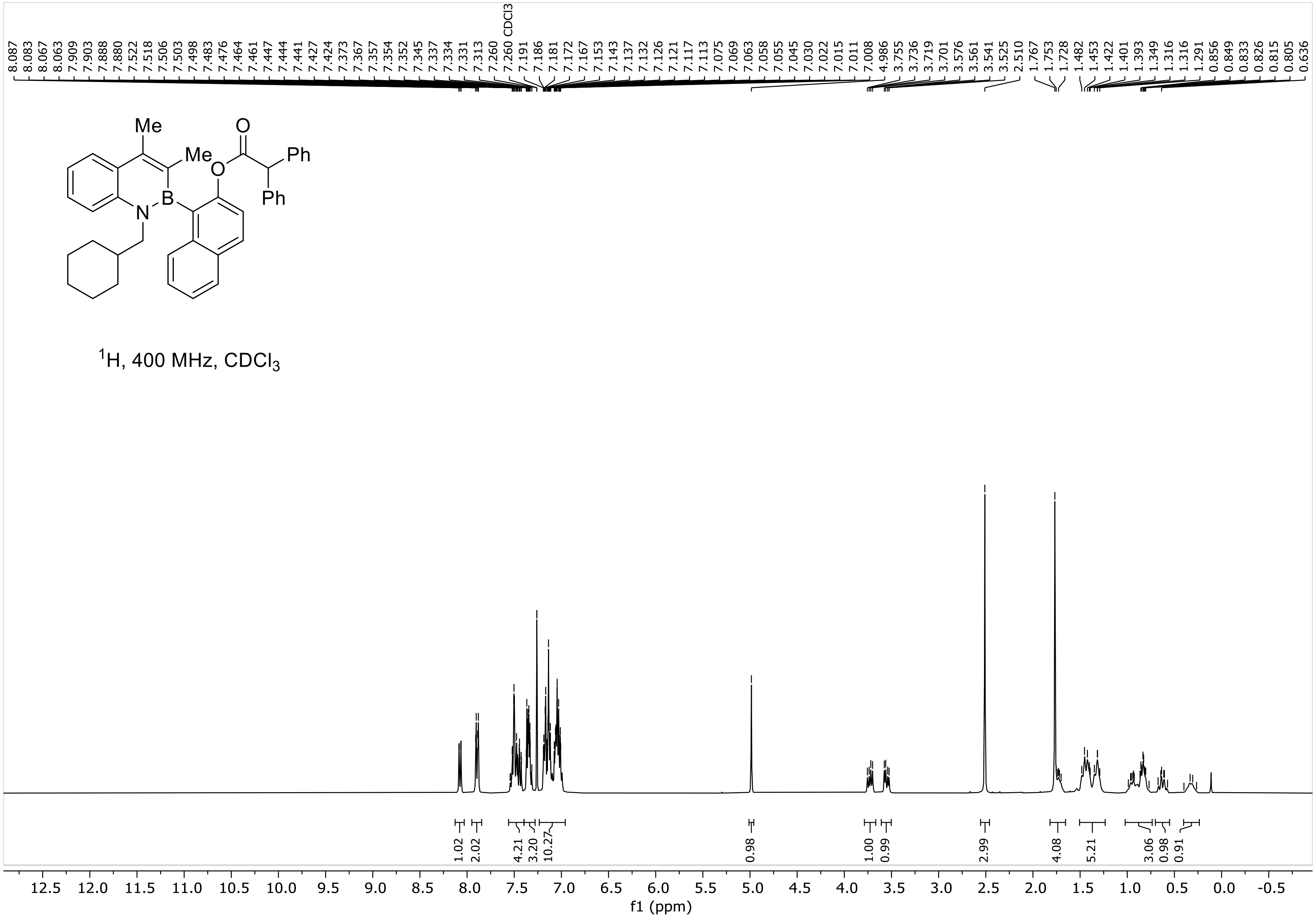
8.087
8.083
8.067
8.063
7.909
7.903
7.888
7.880
7.522
7.518
7.506
7.503
7.498
7.483
7.476
7.464
7.461
7.447
7.444
7.441
7.427
7.424
7.373
7.367
7.357
7.354
7.352
7.345
7.337
7.334
7.331
7.313
7.260
7.260 CDCl3
7.191
7.186
7.181
7.172
7.167
7.153
7.143
7.137
7.132
7.126
7.121
7.117
7.113
7.075
7.069
7.063
7.058
7.055
7.045
7.030
7.022
7.015
7.011
7.008
4.986
3.755
3.736
3.719
3.701
3.576
3.561
3.541
3.525
2.510
1.767
1.753
1.728
1.482
1.453
1.422
1.401
1.393
1.349
1.316
1.291
0.856
0.849
0.833
0.826
0.815
0.805
0.636
Me
O
Me
O
Ph
B
N
Ph
1H, 400 MHz, CDCl3
1.02
2.02
4.21
3.20
10.27
0.98
1.00
0.99
2.99
4.08
5.21
3.06
0.98
0.91
12.5 12.0 11.5 11.0 10.5 10.0 9.5 9.0 8.5 8.0 7.5 7.0 6.5 6.0 5.5 5.0 4.5 4.0 3.5 3.0 2.5 2.0 1.5 1.0 0.5 0.0 -0.5
f1 (ppm)

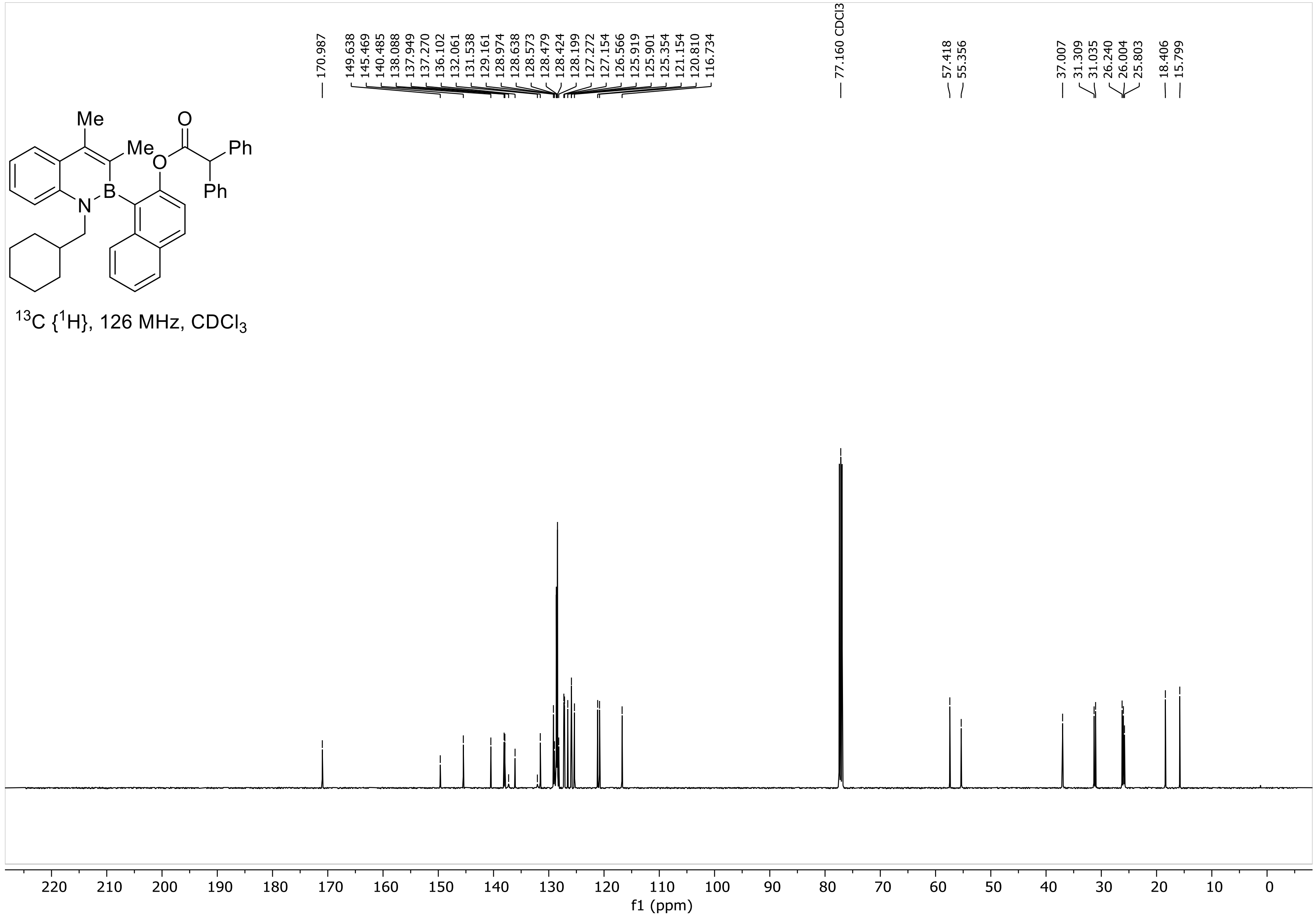

170.987
149.638
145.469
140.485
138.088
137.949
137.270
136.102
132.061
131.538
129.161
128.974
128.638
128.573
128.479
128.424
128.199
127.272
127.154
126.566
125.919
125.901
125.354
121.154
120.810
116.734
77.160 CDCl3
57.418
55.356
37.007
31.309
31.035
26.240
26.004
25.803
18.406
15.799
Me
Me
O
O
Ph
Ph
N
B
^{13}C {^{1}H}, 126 MHz, $CDCl_3$
220 210 200 190 180 170 160 150 140 130 120 110 100 90 80 70 60 50 40 30 20 10 0
f1 (ppm)

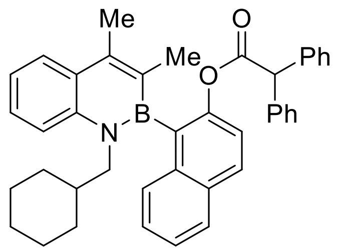

^{11}B {^{1}H}, 160 MHz, $CDCl_3$

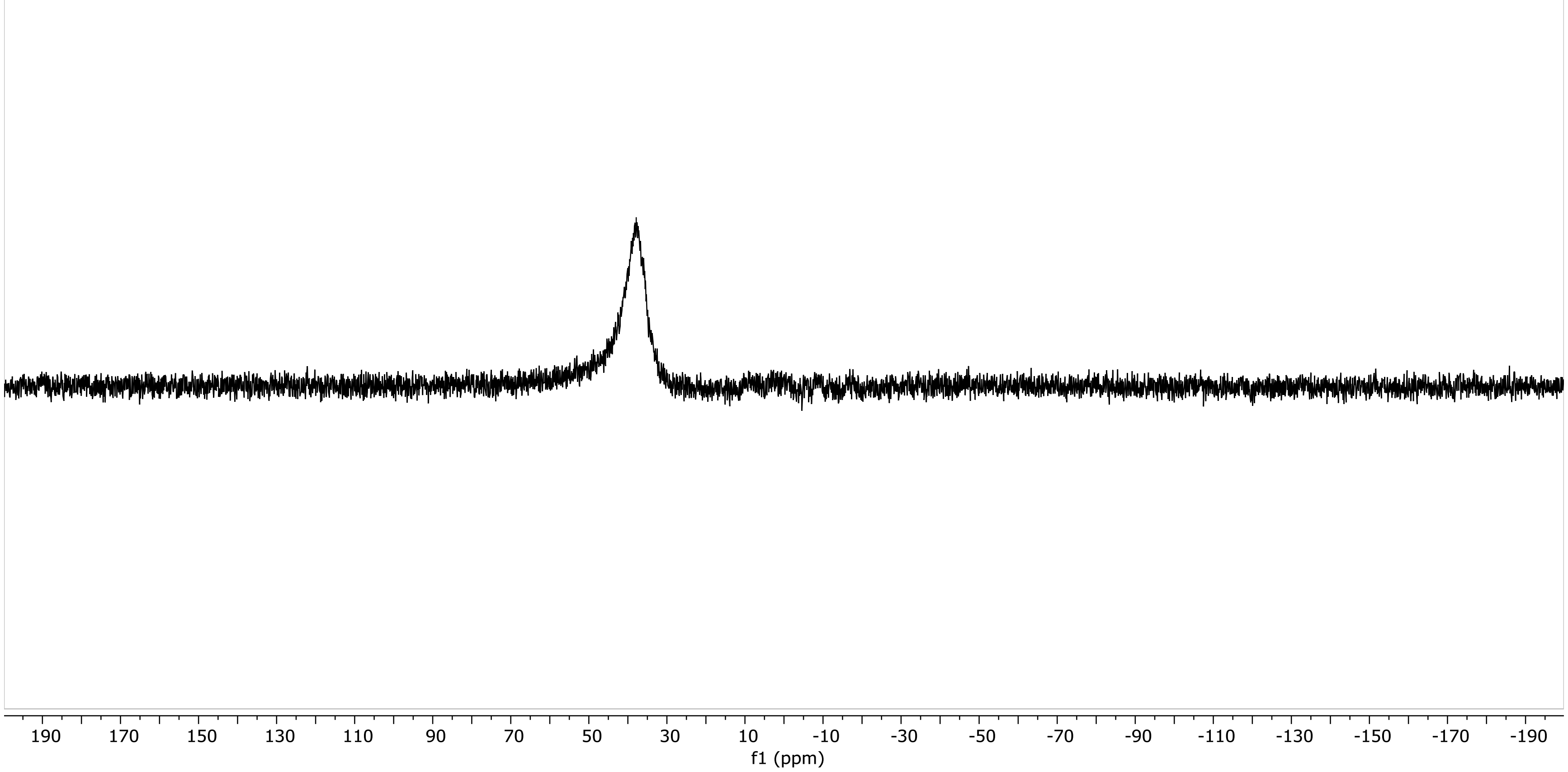

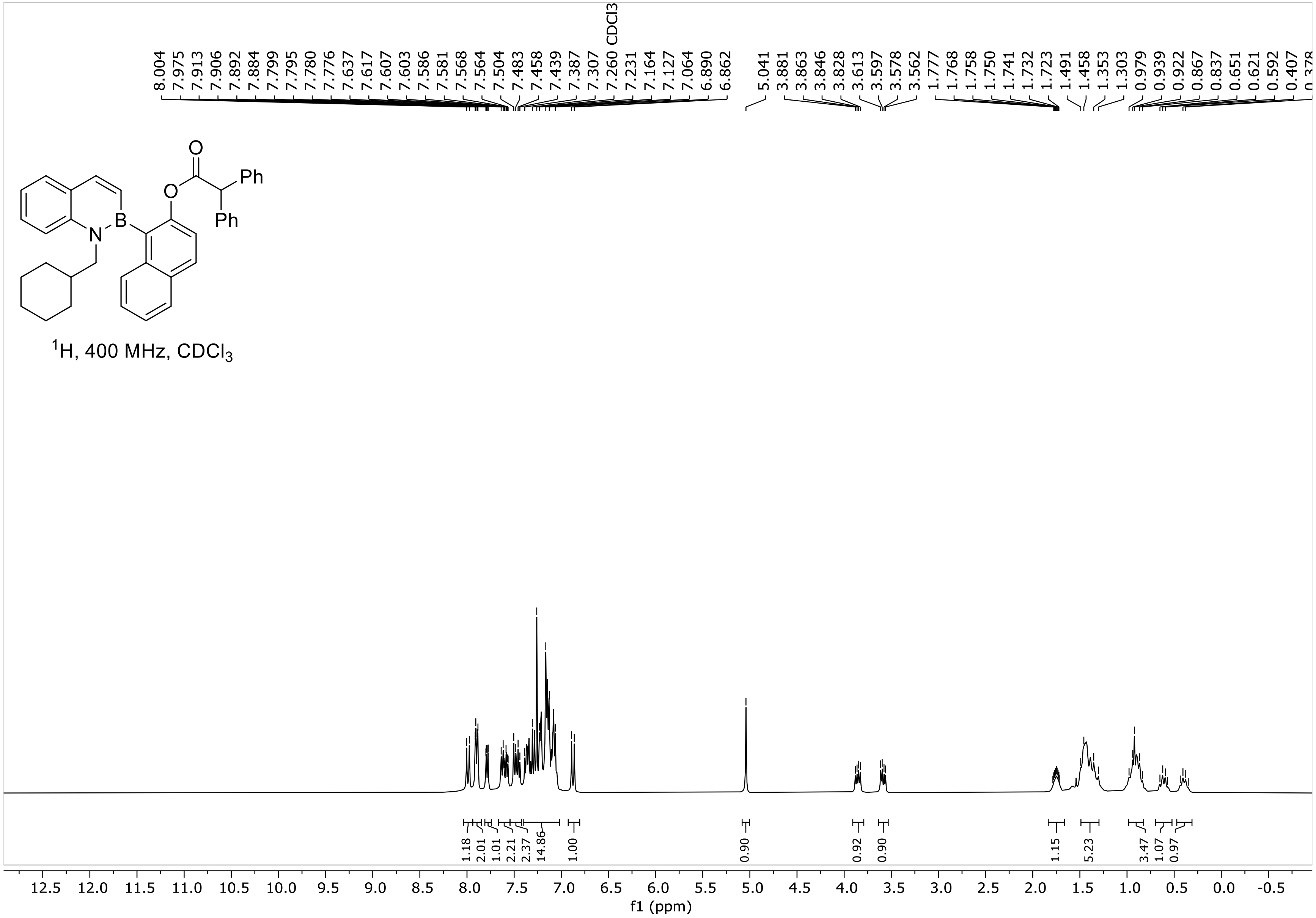
CDCl3
8.004 7.975 7.913 7.906 7.892 7.884 7.799 7.795 7.780 7.776 7.637 7.617 7.607 7.603 7.586 7.581 7.568 7.564 7.504 7.483 7.458 7.439 7.387 7.307 7.260 7.231 7.164 7.127 7.064 6.890 6.862
5.041 3.881 3.863 3.846 3.828 3.613 3.597 3.578 3.562 1.777 1.768 1.758 1.750 1.741 1.732 1.723 1.491 1.458 1.353 1.303 0.979 0.939 0.922 0.867 0.837 0.651 0.621 0.592 0.407
O
Ph
O
B
N
Ph
1H, 400 MHz, CDCl3
1.18 2.01 1.01 2.21 2.37 14.86 1.00 0.90 0.92 0.90 1.15 5.23 3.47 1.07 0.97
12.5 12.0 11.5 11.0 10.5 10.0 9.5 9.0 8.5 8.0 7.5 7.0 6.5 6.0 5.5 5.0 4.5 4.0 3.5 3.0 2.5 2.0 1.5 1.0 0.5 0.0 -0.5
f1 (ppm)

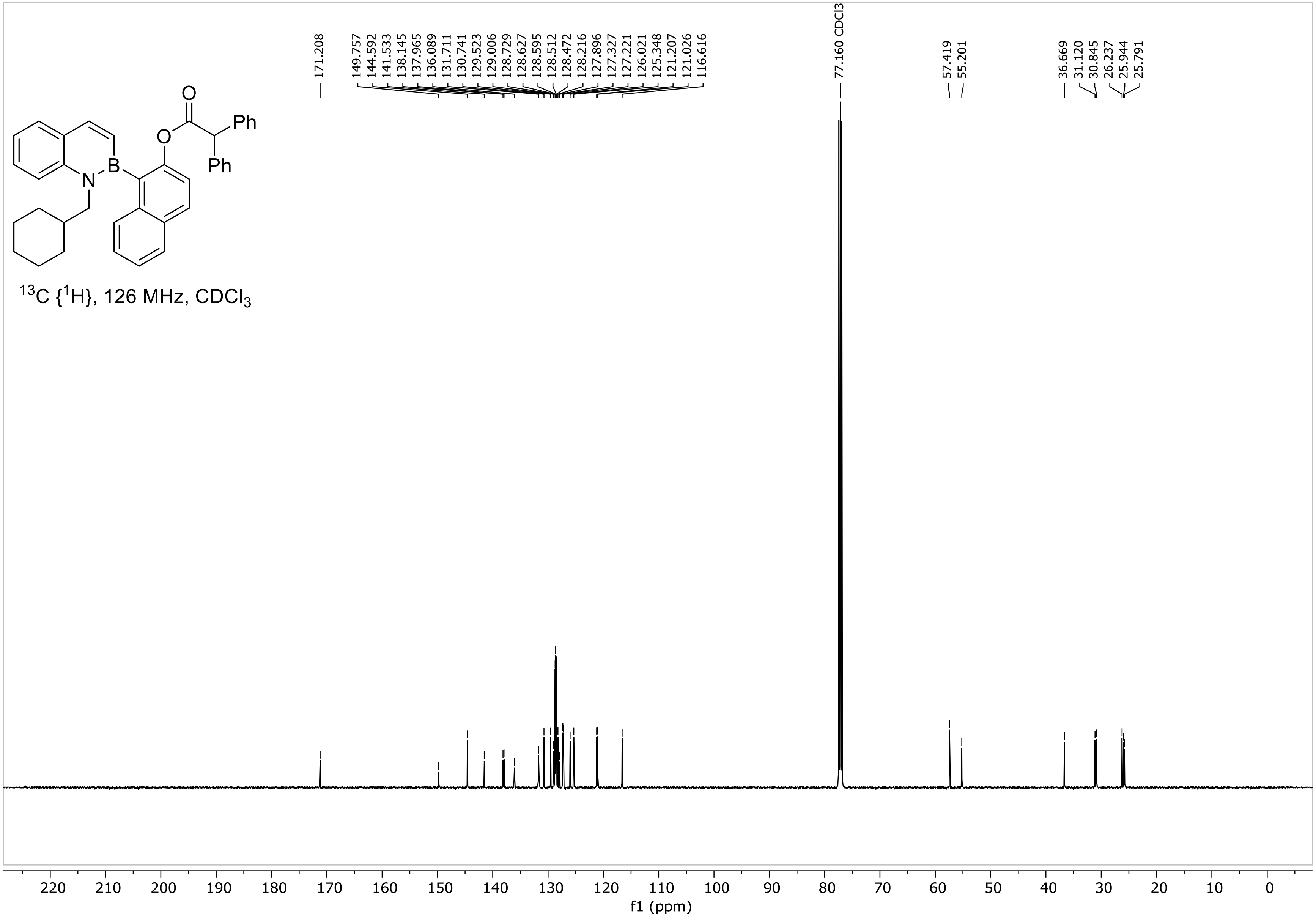

171.208
149.757
144.592
141.533
138.145
137.965
136.089
131.711
130.741
129.523
129.006
128.729
128.627
128.595
128.512
128.472
128.216
127.896
127.327
127.221
126.021
125.348
121.207
121.026
116.616
77.160 CDCl3
57.419
55.201
36.669
31.120
30.845
26.237
25.944
25.791
O
Ph
O
B
N
Ph
13C {1H}, 126 MHz, CDCl3
f1 (ppm)
220 210 200 190 180 170 160 150 140 130 120 110 100 90 80 70 60 50 40 30 20 10 0

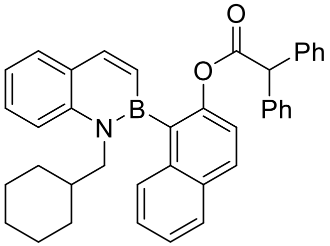

$^{11}B\ \{^1H\}$, 128 MHz, $CDCl_3$

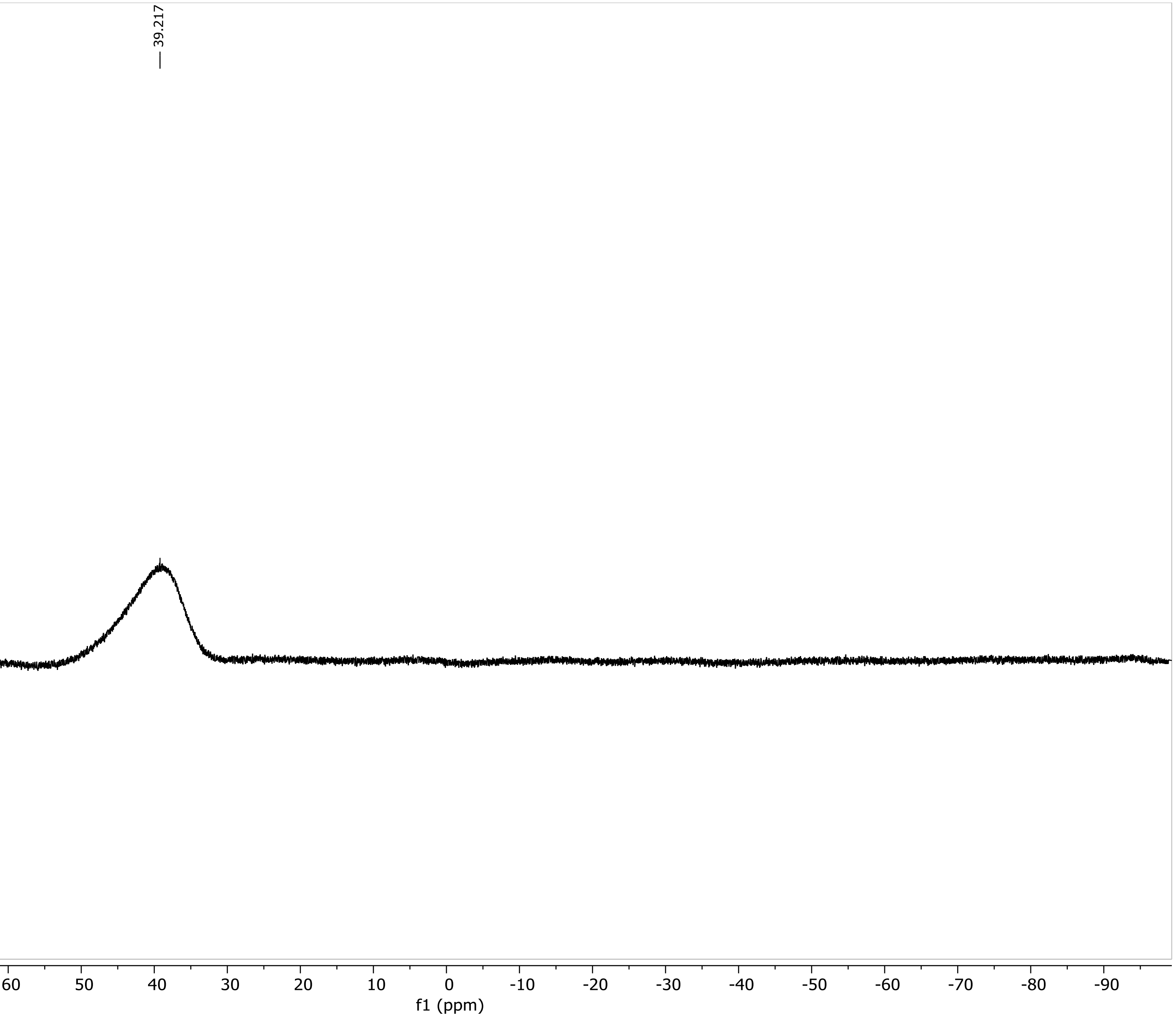

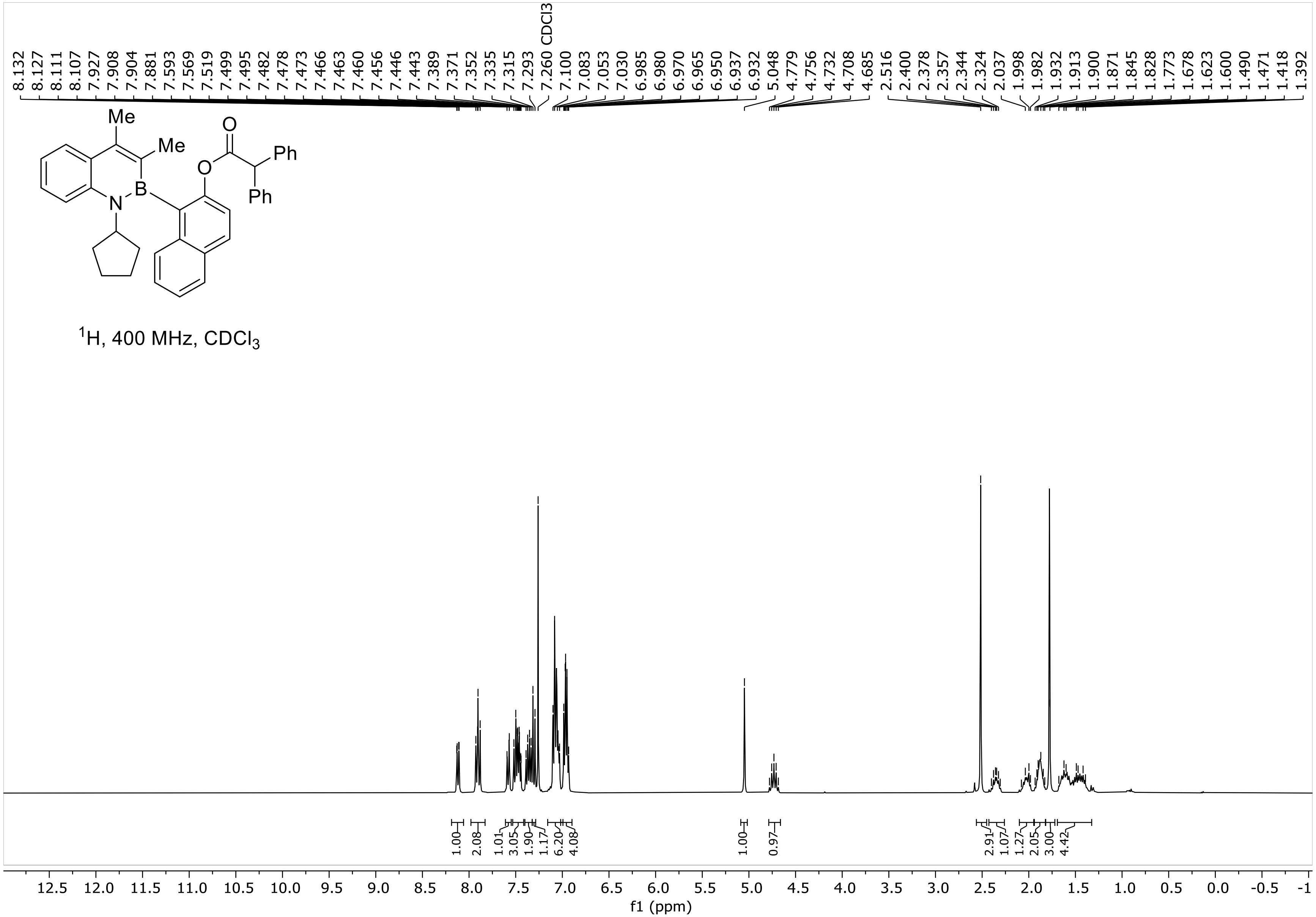
8.132 8.127 8.111 8.107 7.927 7.908 7.904 7.881 7.593 7.569 7.519 7.499 7.495 7.482 7.478 7.473 7.466 7.463 7.460 7.456 7.446 7.443 7.389 7.371 7.352 7.335 7.315 7.293 7.260 CDCl3 7.100 7.083 7.053 7.030 6.985 6.980 6.970 6.965 6.950 6.937 6.932 5.048 4.779 4.756 4.732 4.708 4.685 2.516 2.400 2.378 2.357 2.344 2.324 2.037 1.998 1.982 1.932 1.913 1.900 1.871 1.845 1.828 1.773 1.678 1.623 1.600 1.490 1.471 1.418 1.392
Me
Me
O
O
Ph
Ph
B
N
^{1}H, 400 MHz, $CDCl_3$
1.00 2.08 1.01 3.05 1.90 1.17 6.20 4.08 1.00 0.97 2.91 1.07 1.27 2.05 3.00 4.42
12.5 12.0 11.5 11.0 10.5 10.0 9.5 9.0 8.5 8.0 7.5 7.0 6.5 6.0 5.5 5.0 4.5 4.0 3.5 3.0 2.5 2.0 1.5 1.0 0.5 0.0 -0.5 -1
f1 (ppm)

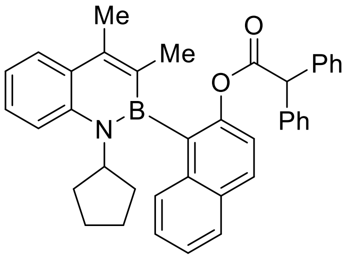

^{13}C {^{1}H}, 126 MHz, $CDCl_3$

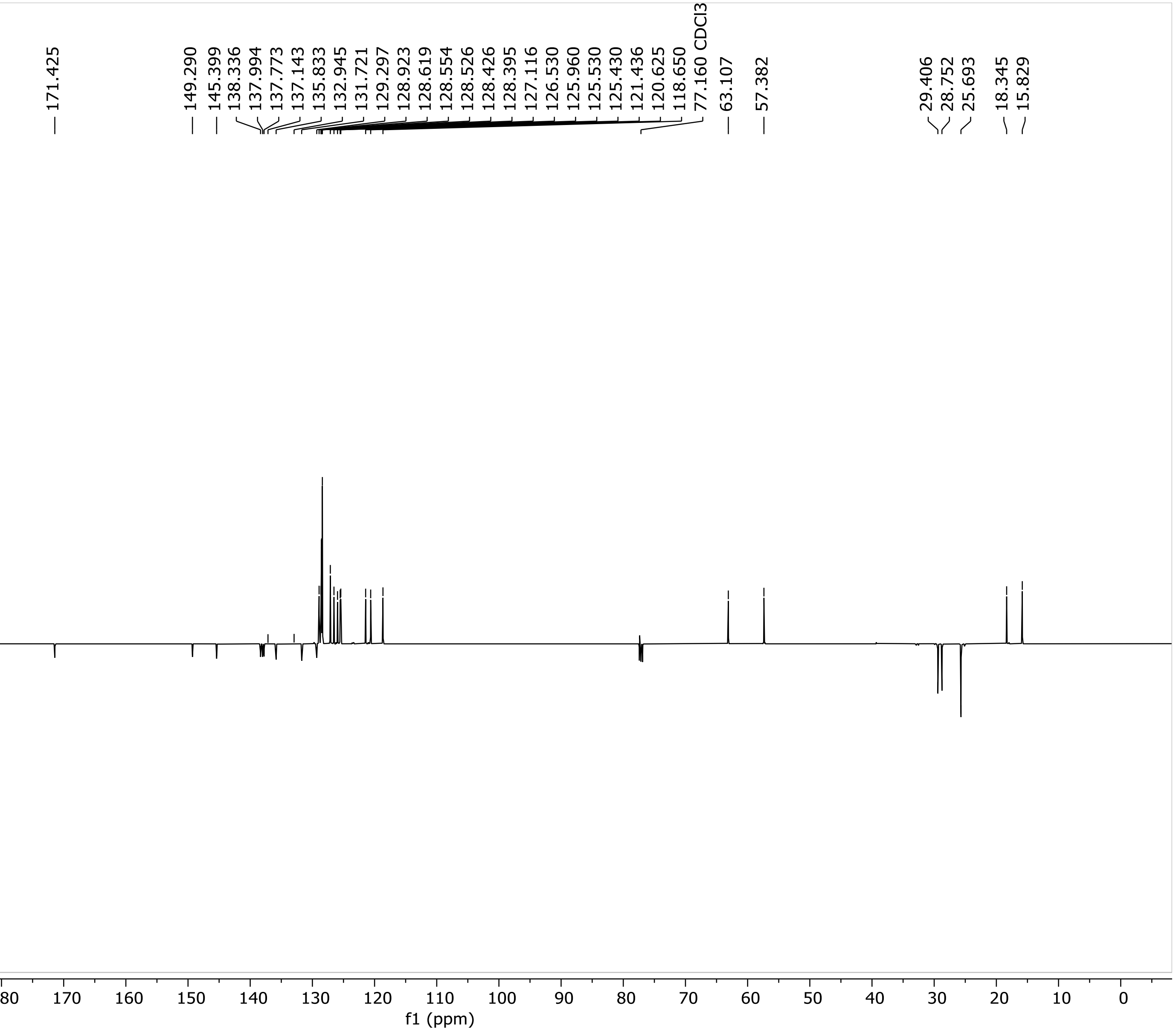

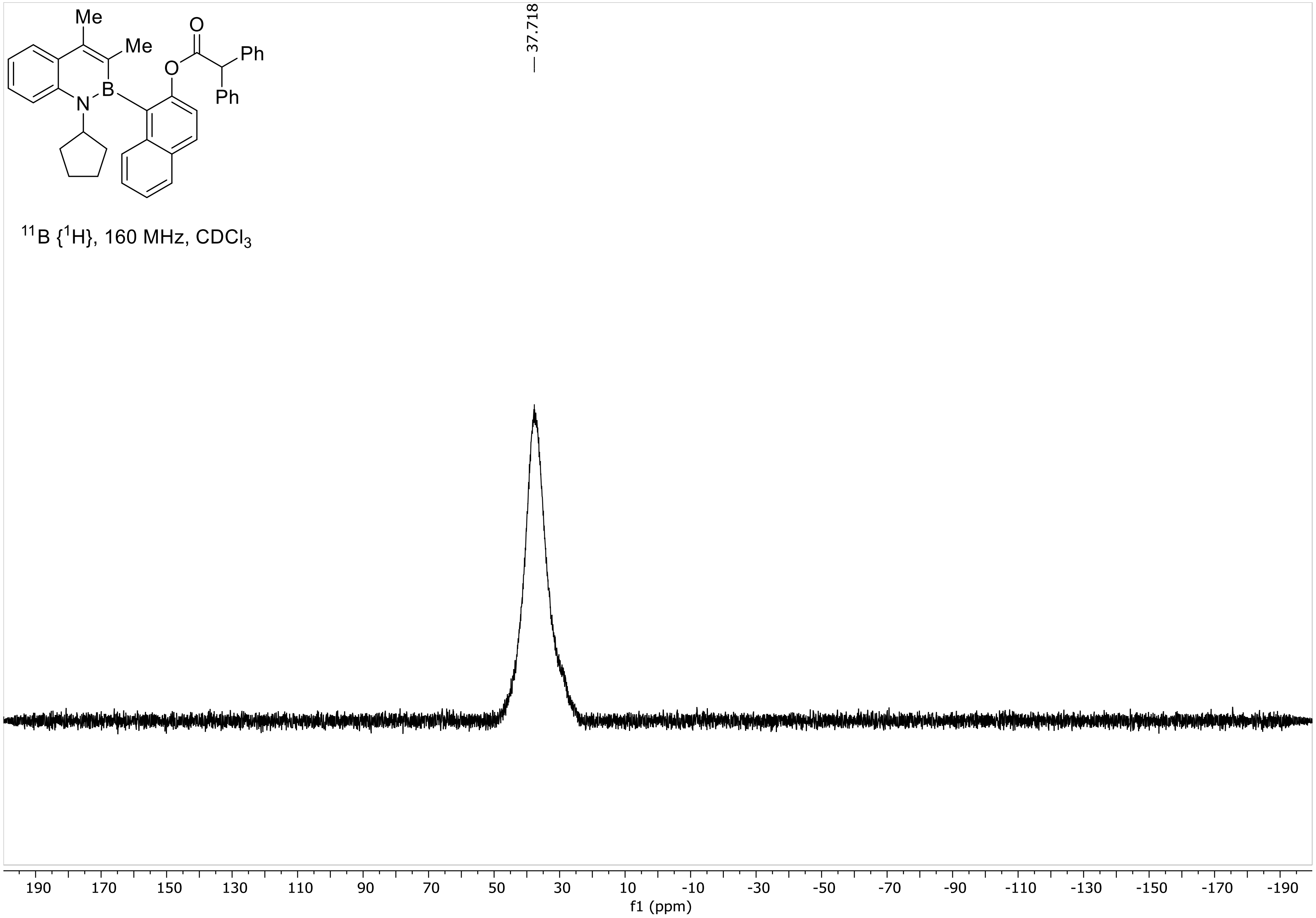
37.718
Me
Me
O
O
Ph
Ph
B
N
11B {1H}, 160 MHz, CDCl3
190 170 150 130 110 90 70 50 30 10 -10 -30 -50 -70 -90 -110 -130 -150 -170 -190
f1 (ppm)

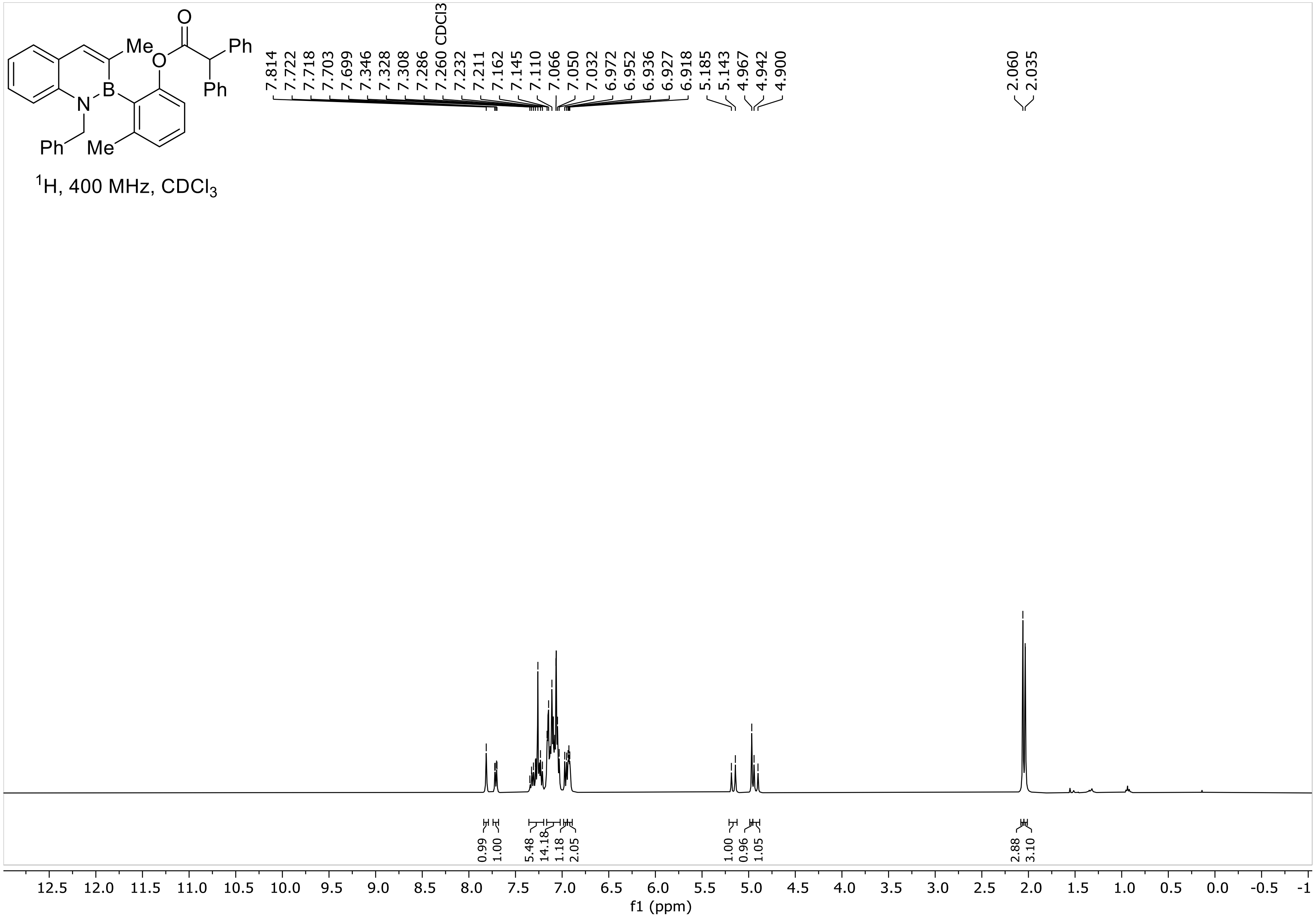

Me
O
O
Ph
Ph
N
B
Ph
Me
1H, 400 MHz, CDCl3
7.814
7.722
7.718
7.703
7.699
7.346
7.328
7.308
7.286
7.260 CDCl3
7.232
7.211
7.162
7.145
7.110
7.066
7.050
7.032
6.972
6.952
6.936
6.927
6.918
5.185
5.143
4.967
4.942
4.900
2.060
2.035
0.99
1.00
5.48
14.18
1.18
2.05
1.00
0.96
1.05
2.88
3.10
12.5 12.0 11.5 11.0 10.5 10.0 9.5 9.0 8.5 8.0 7.5 7.0 6.5 6.0 5.5 5.0 4.5 4.0 3.5 3.0 2.5 2.0 1.5 1.0 0.5 0.0 -0.5 -1
f1 (ppm)

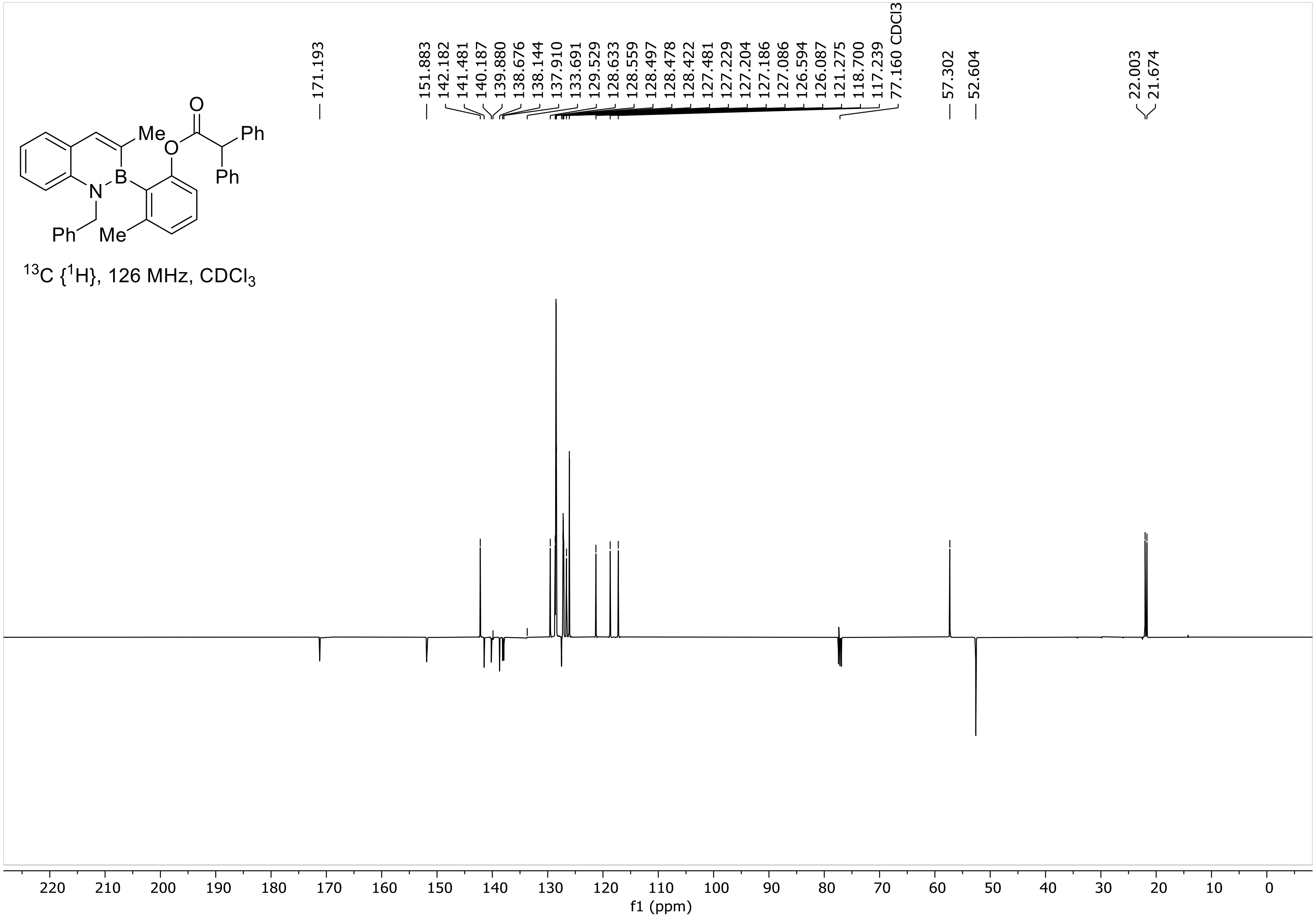

$^{13}C\ \{^1H\}$, 126 MHz, $CDCl_3$
171.193
151.883
142.182
141.481
140.187
139.880
138.676
138.144
137.910
133.691
129.529
128.633
128.559
128.497
128.478
128.422
127.481
127.229
127.204
127.186
127.086
126.594
126.087
121.275
118.700
117.239
77.160 CDCl3
57.302
52.604
22.003
21.674
f1 (ppm)

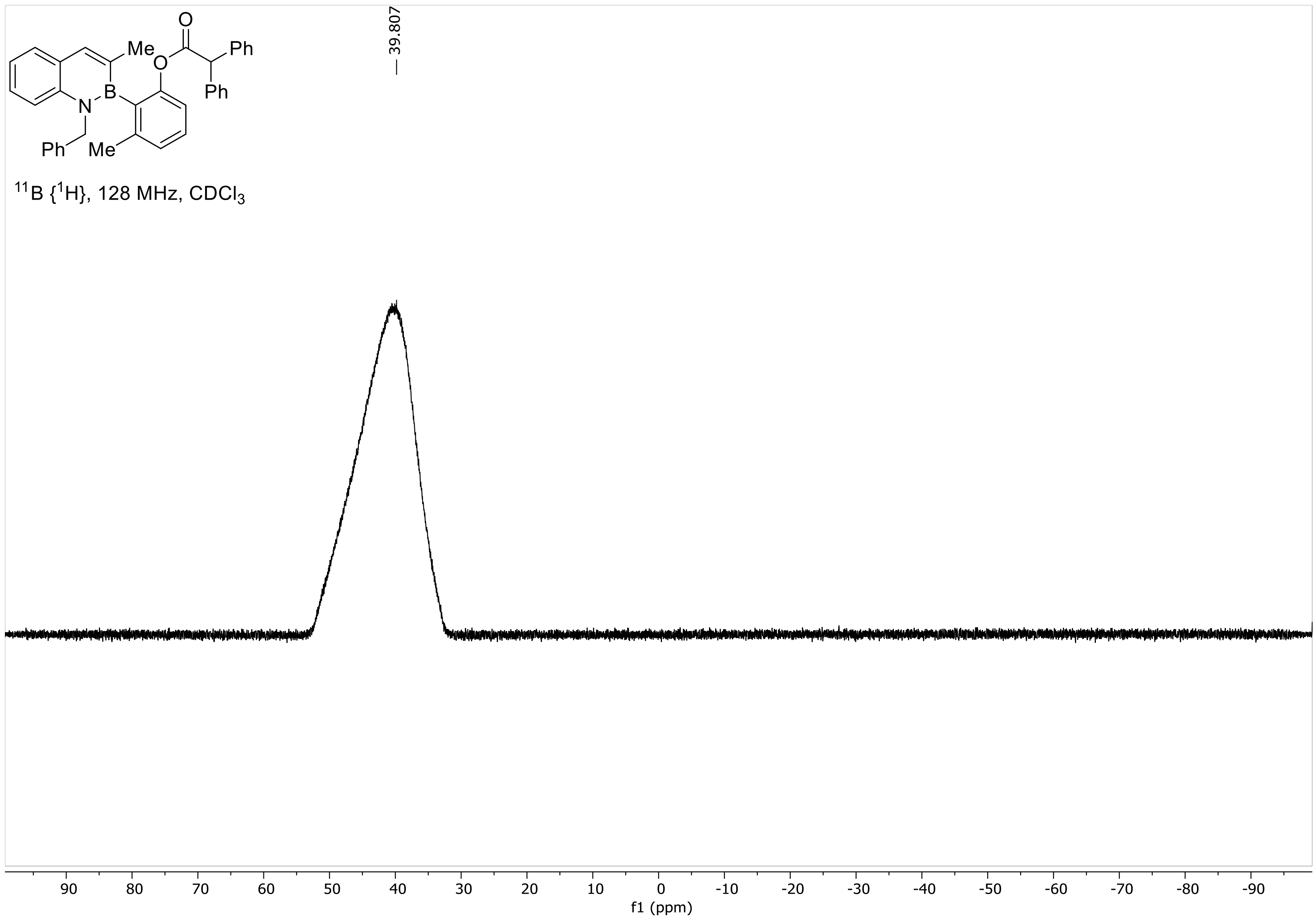
Me
O
O
Ph
Ph
N
B
Ph
Me
11B {1H}, 128 MHz, CDCl3
39.807
90
80
70
60
50
40
30
20
10
0
-10
-20
-30
-40
-50
-60
-70
-80
-90
f1 (ppm)

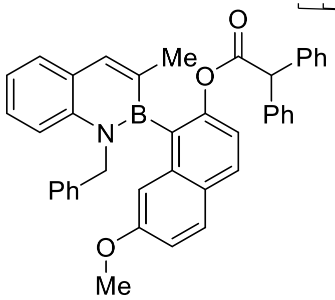

^1H, 400 MHz, $CDCl_3$

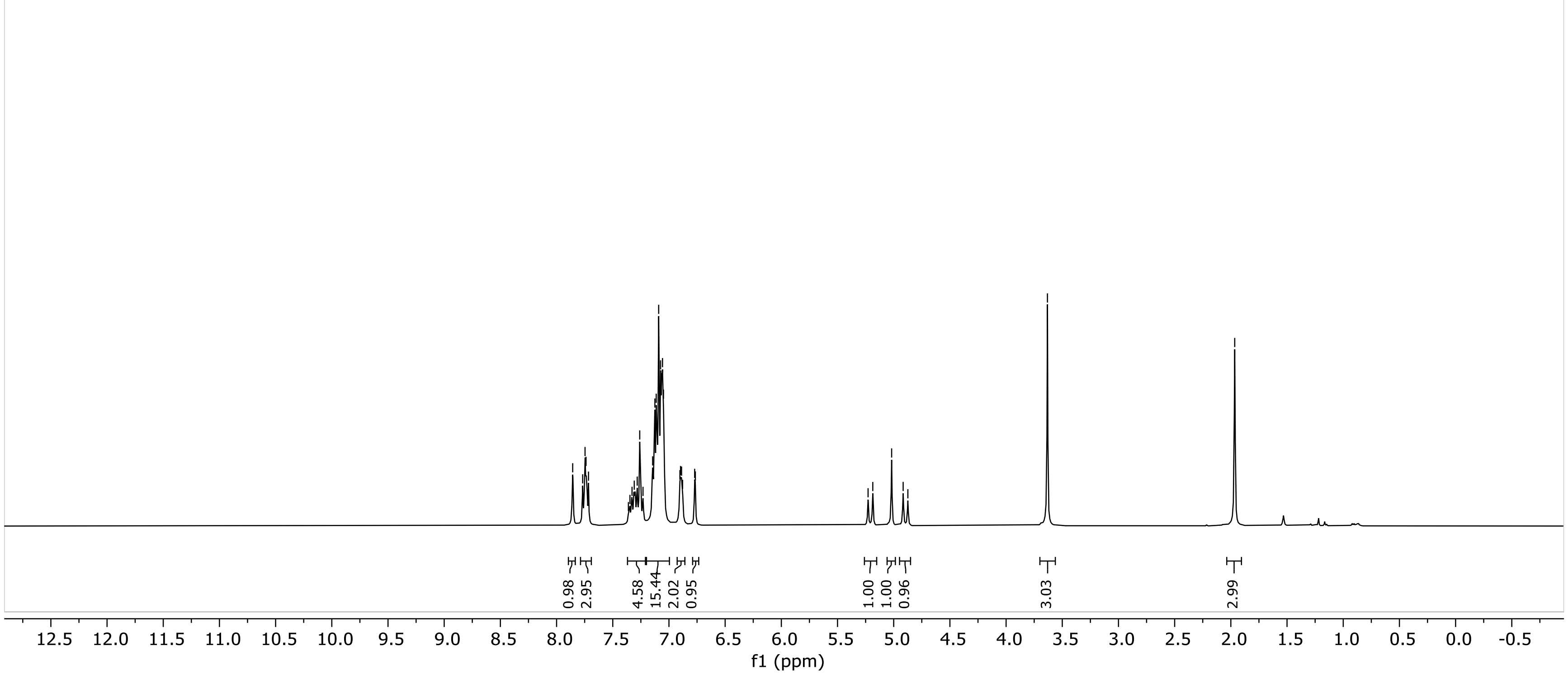

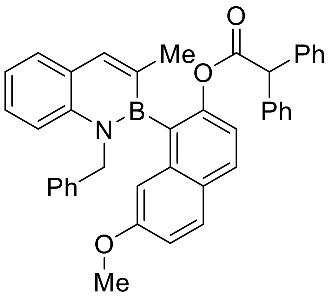

^{13}C {^{1}H}, 126 MHz, $CDCl_3$

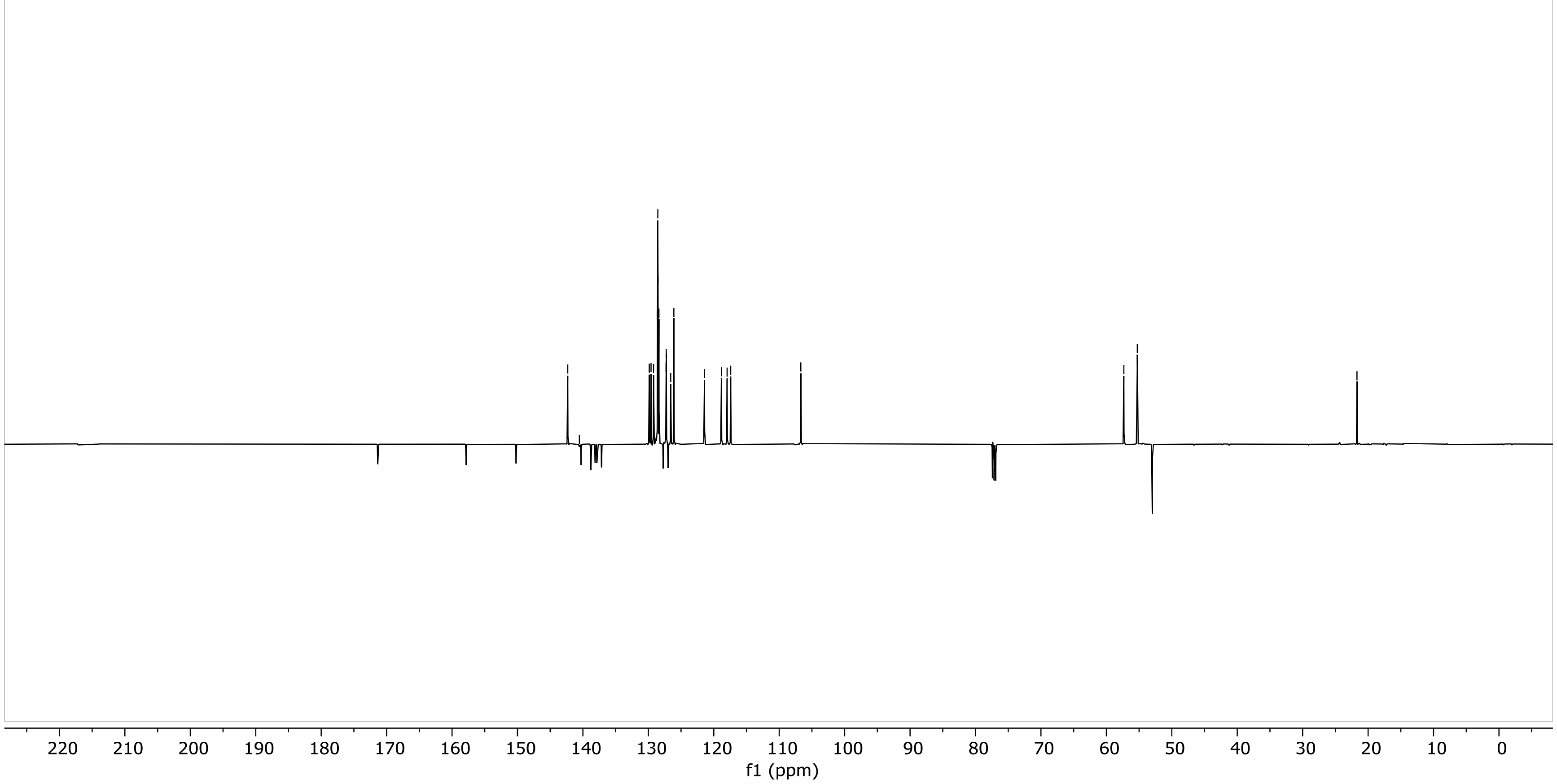

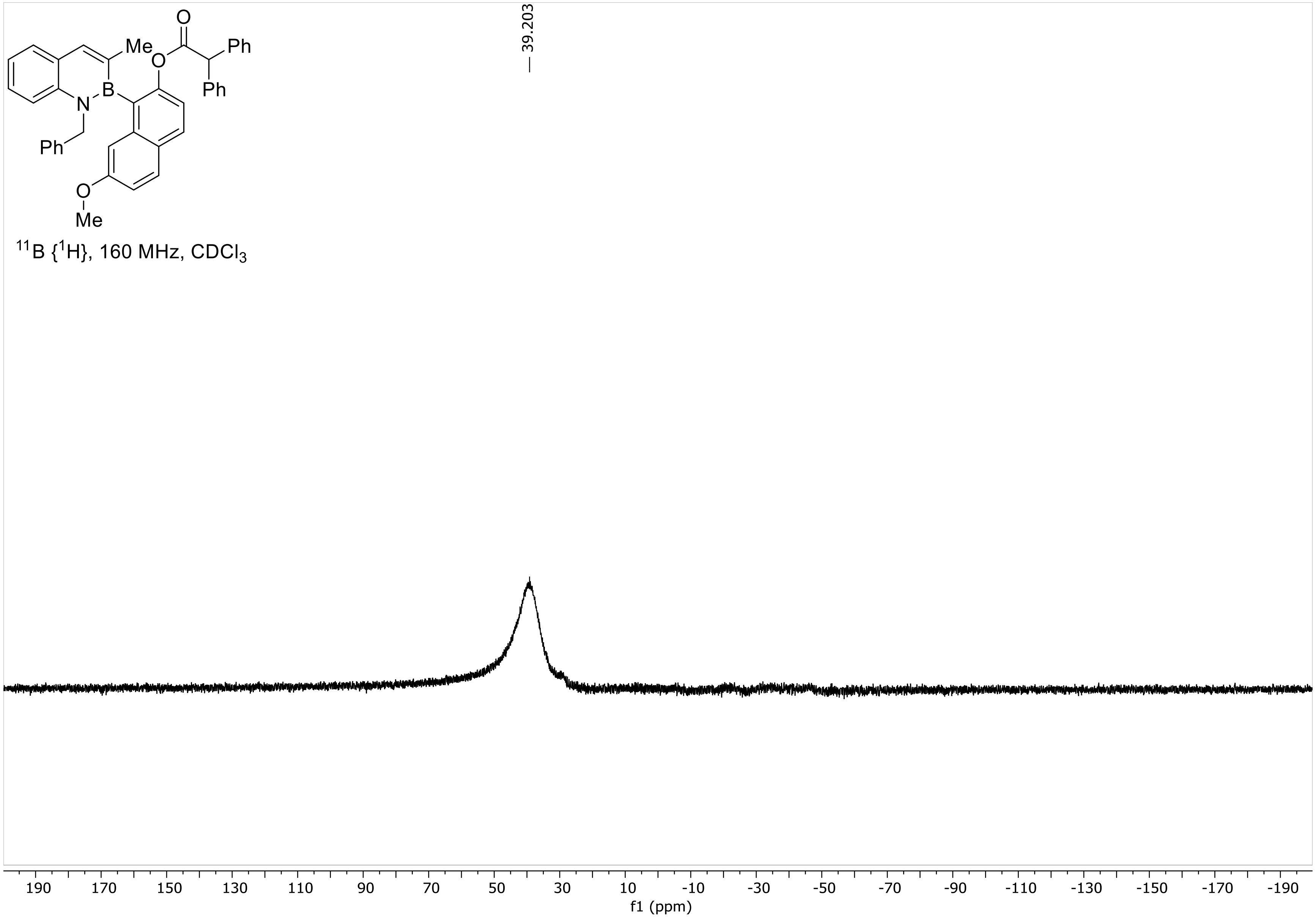

^{11}B {^{1}H}, 160 MHz, $CDCl_3$
39.203
190 170 150 130 110 90 70 50 30 10 -10 -30 -50 -70 -90 -110 -130 -150 -170 -190
f1 (ppm)

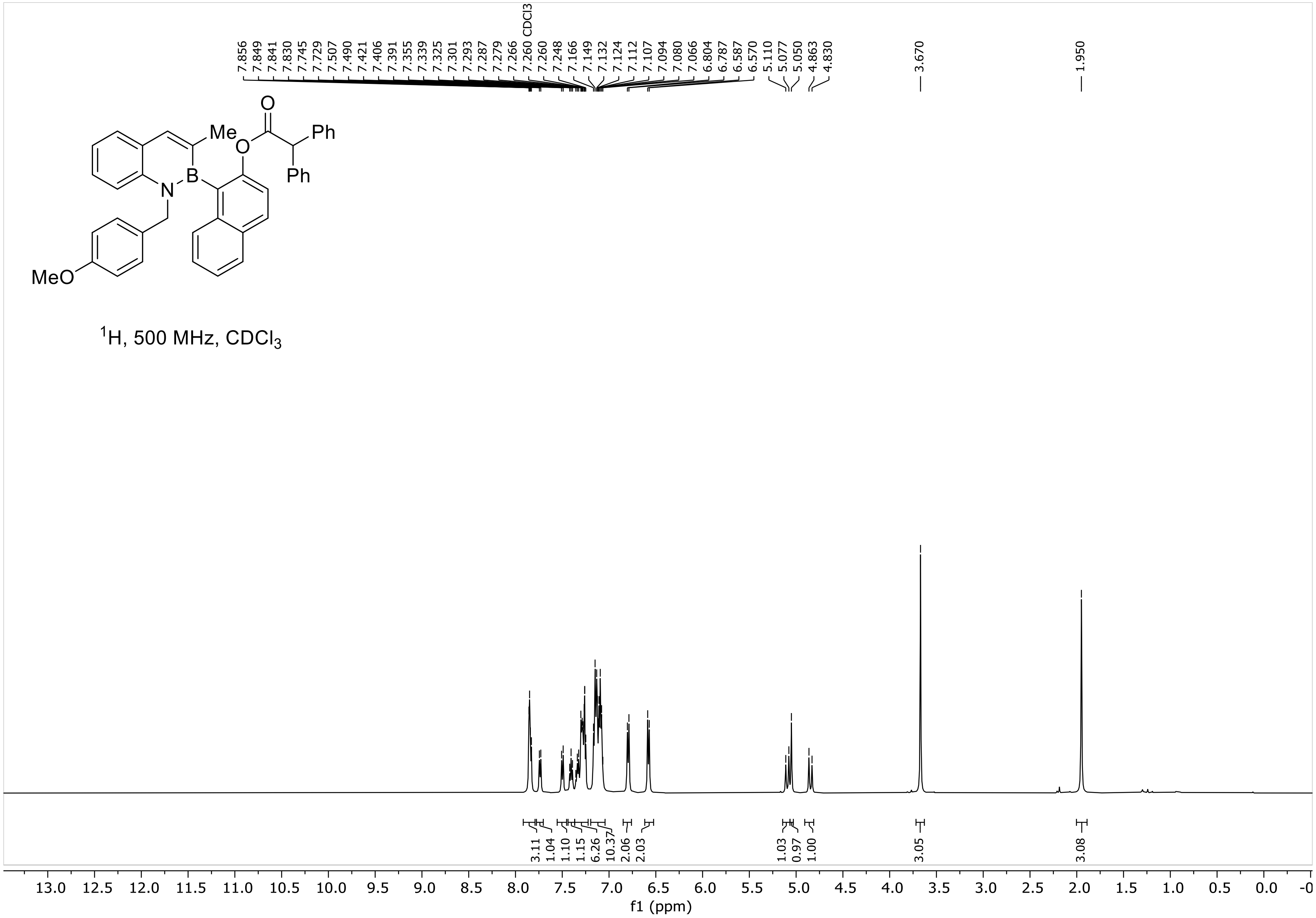
Me
O
O
Ph
Ph
N
B
MeO
¹H, 500 MHz, CDCl₃
f1 (ppm)

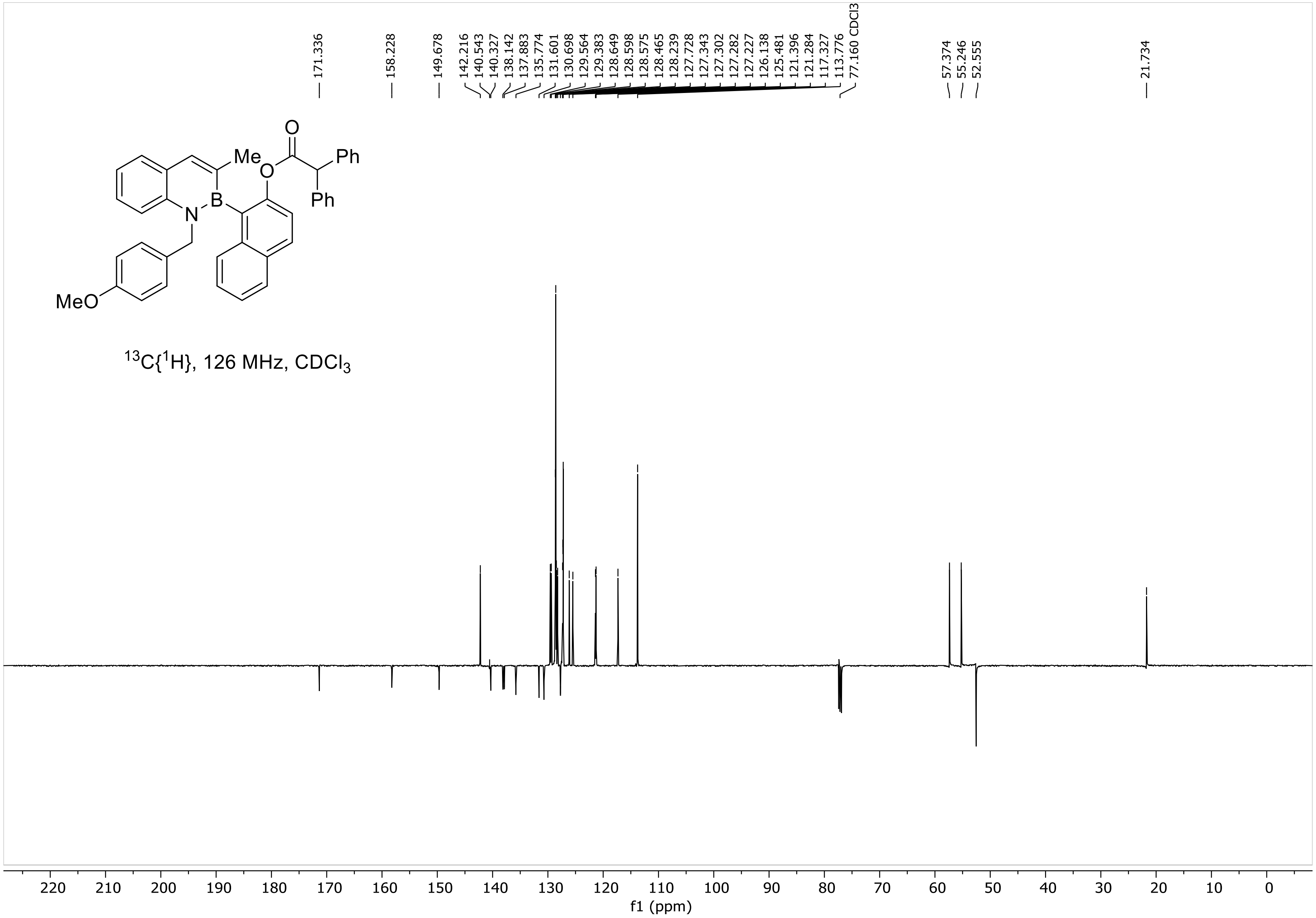

171.336
158.228
149.678
142.216
140.543
140.327
138.142
137.883
135.774
131.601
130.698
129.564
129.383
128.649
128.598
128.575
128.465
128.239
127.728
127.343
127.302
127.282
127.227
126.138
125.481
121.396
121.284
117.327
113.776
77.160 CDCl3
57.374
55.246
52.555
21.734
Me
O
O
Ph
Ph
B
N
MeO
$^{13}C\{^{1}H\}$, 126 MHz, $CDCl_3$
220 210 200 190 180 170 160 150 140 130 120 110 100 90 80 70 60 50 40 30 20 10 0
f1 (ppm)

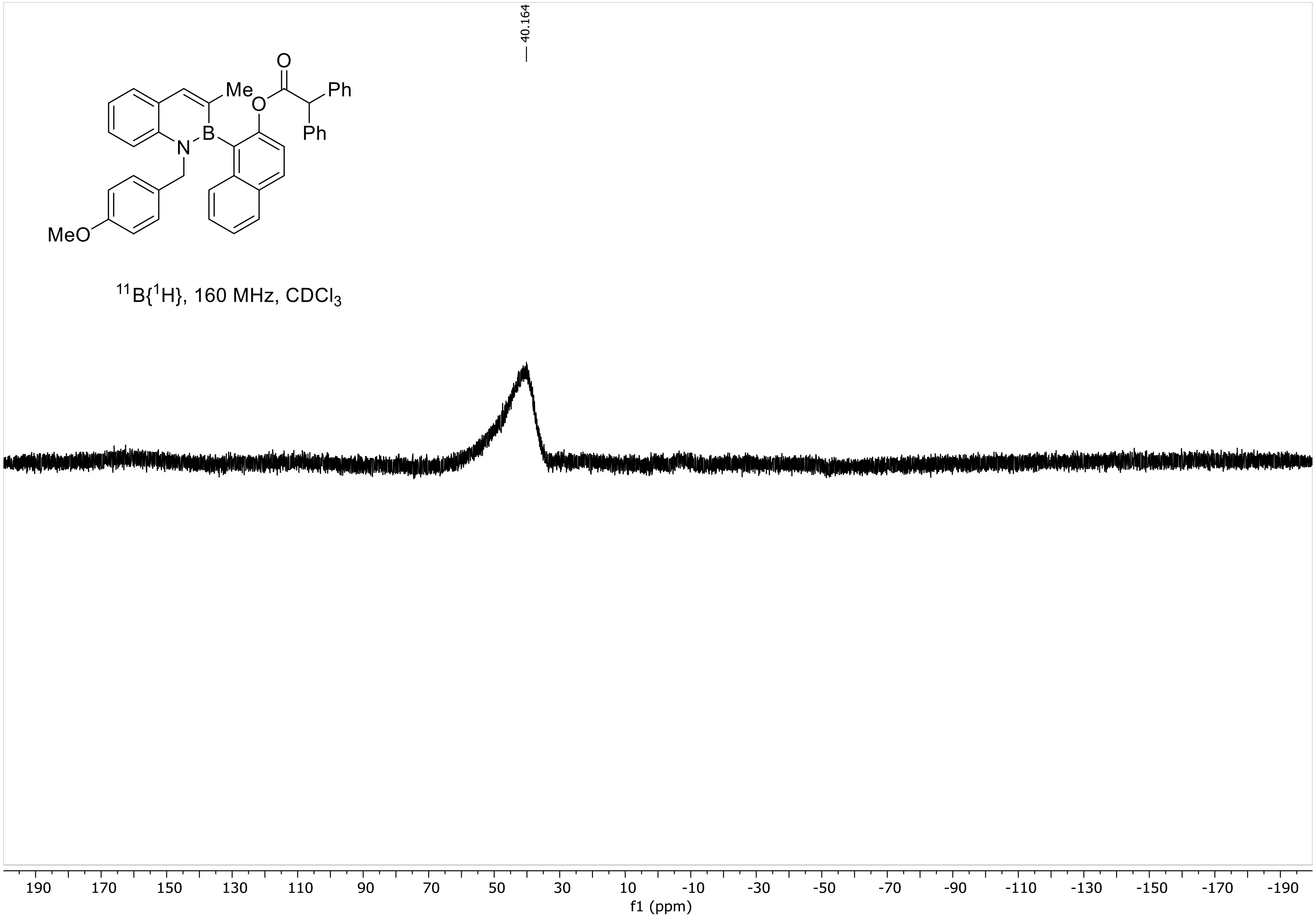
40.164
Me
O
O
Ph
Ph
B
N
MeO
11B{1H}, 160 MHz, CDCl3
190 170 150 130 110 90 70 50 30 10 -10 -30 -50 -70 -90 -110 -130 -150 -170 -190
f1 (ppm)

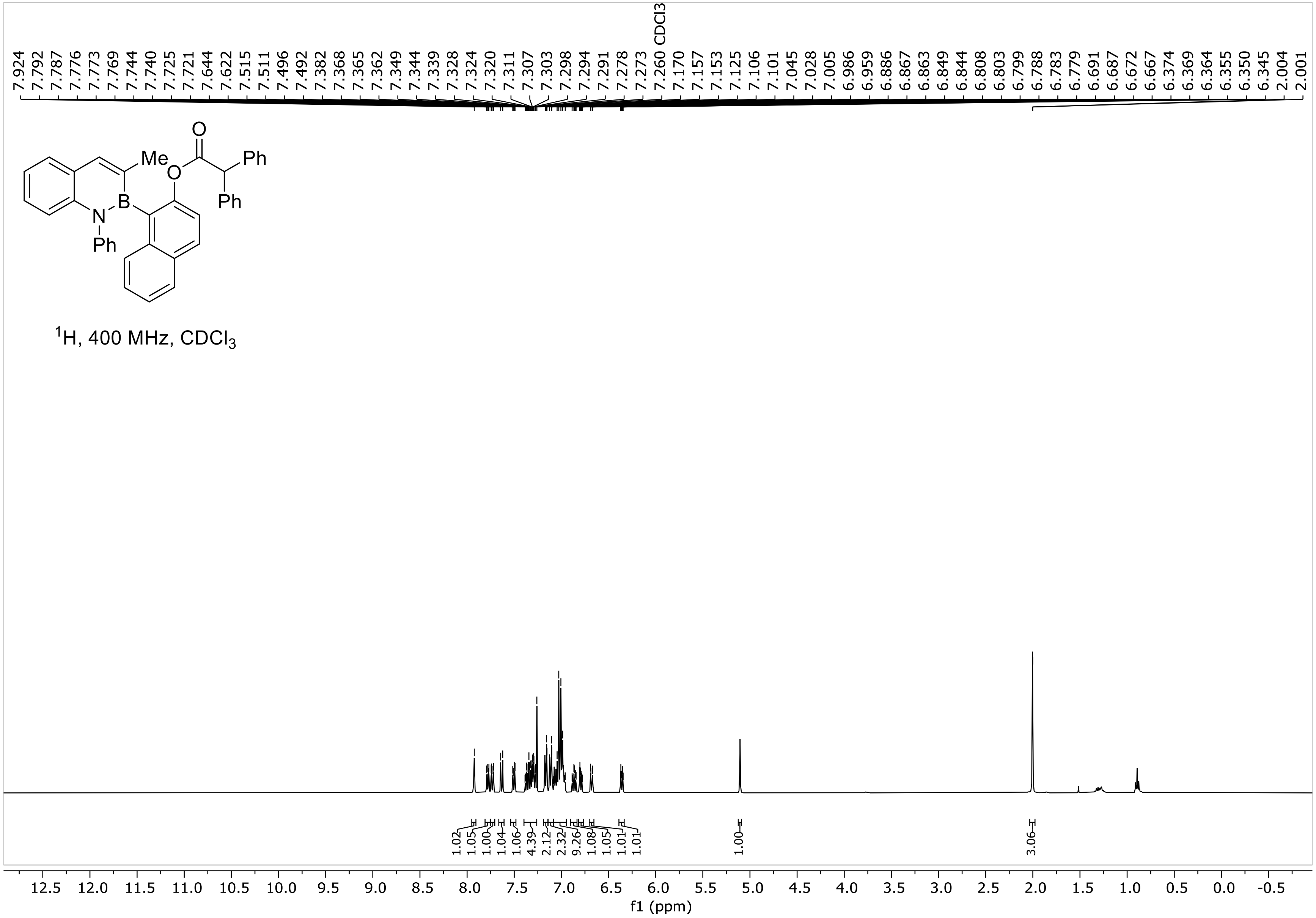

^1H, 400 MHz, $CDCl_3$

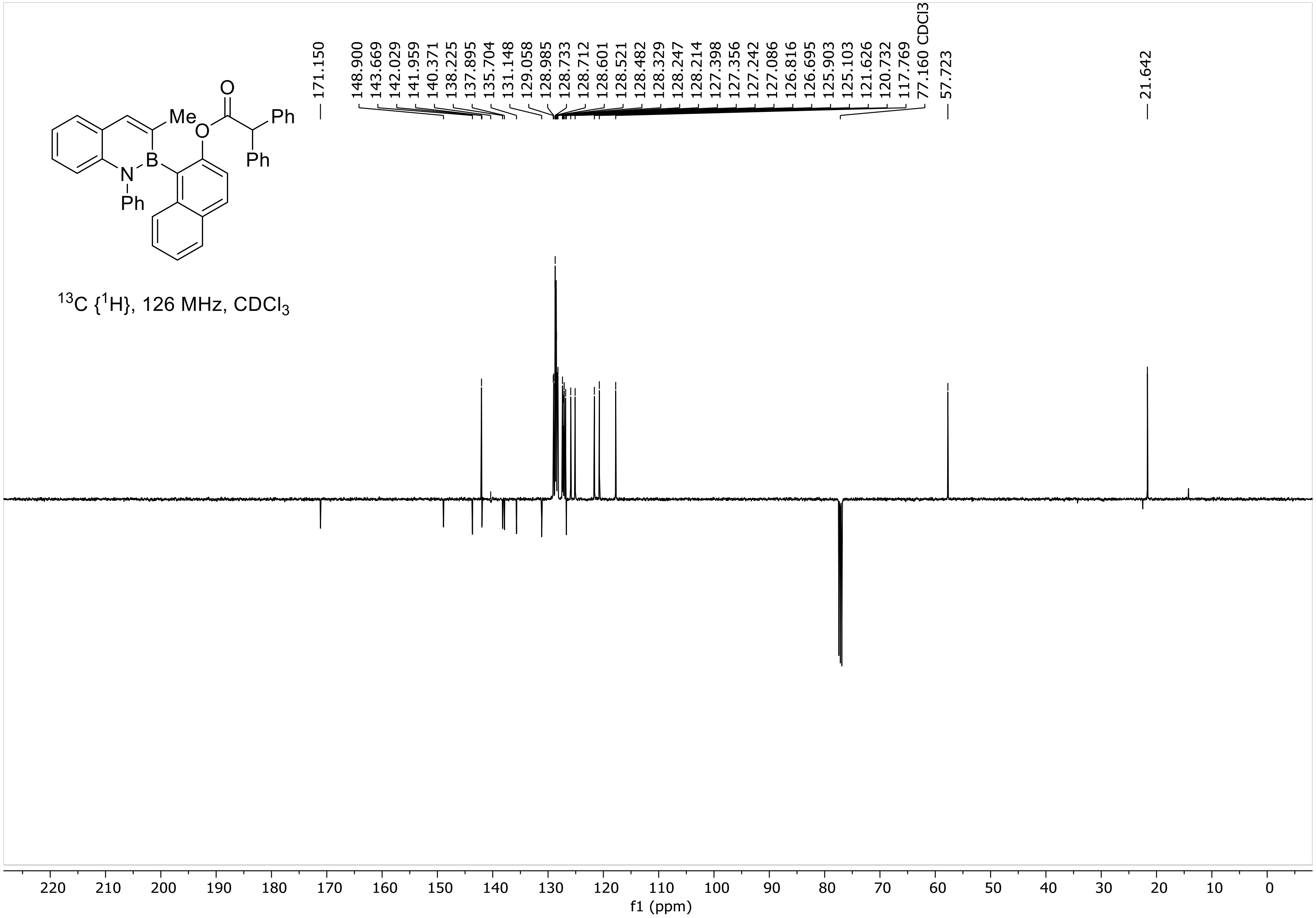

Me
O
O
Ph
Ph
B
N
Ph
13C {1H}, 126 MHz, CDCl3
171.150
148.900
143.669
142.029
141.959
140.371
138.225
137.895
135.704
131.148
129.058
128.985
128.733
128.712
128.601
128.521
128.482
128.329
128.247
128.214
127.398
127.356
127.242
127.086
126.816
126.695
125.903
125.103
121.626
120.732
117.769
77.160 CDCl3
57.723
21.642
220
210
200
190
180
170
160
150
140
130
120
110
100
90
80
70
60
50
40
30
20
10
0
f1 (ppm)

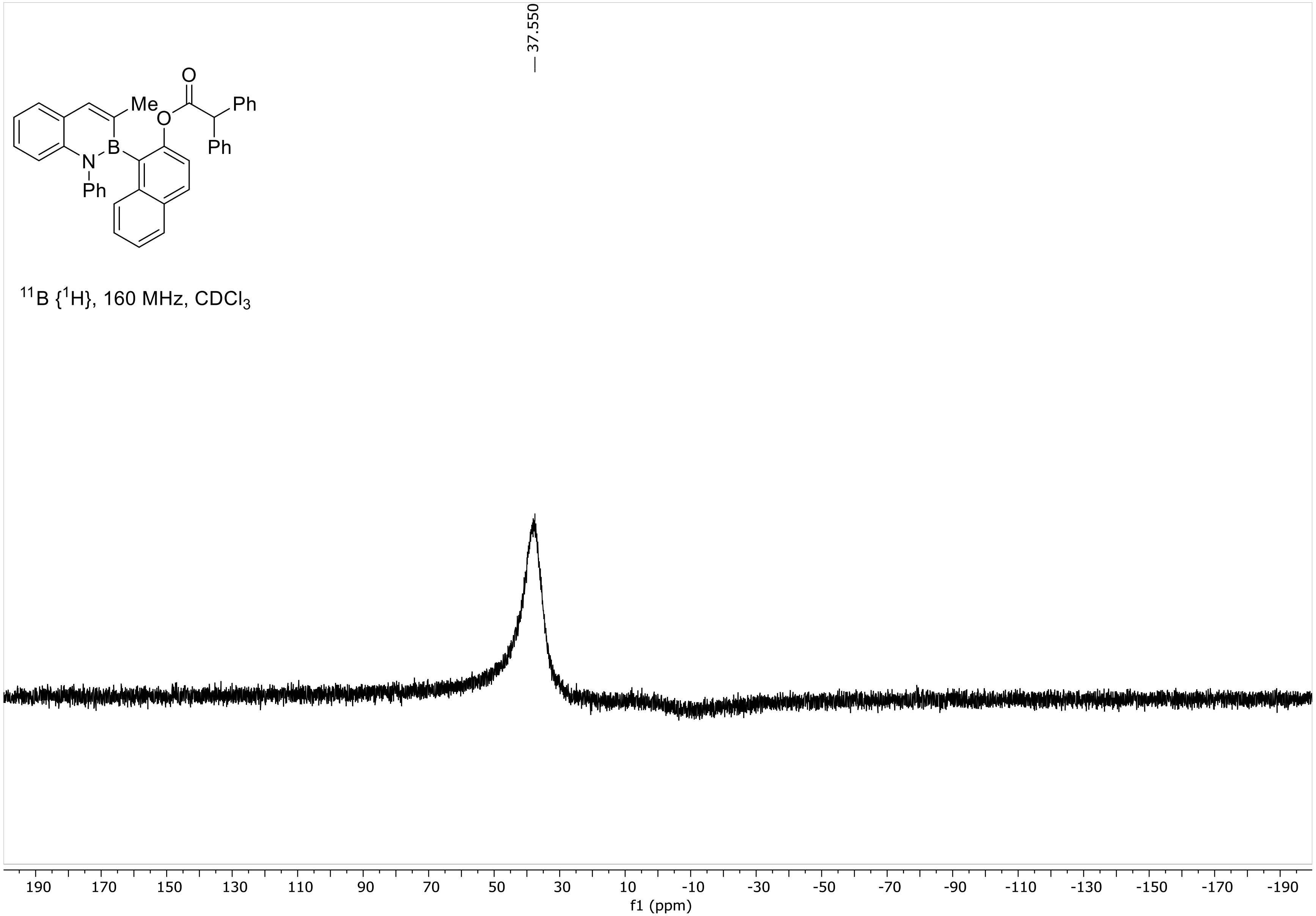
37.550
Me
O
O
Ph
Ph
N
B
Ph
^{11}B {^{1}H}, 160 MHz, $CDCl_3$
190
170
150
130
110
90
70
50
30
10
-10
-30
-50
-70
-90
-110
-130
-150
-170
-190
f1 (ppm)

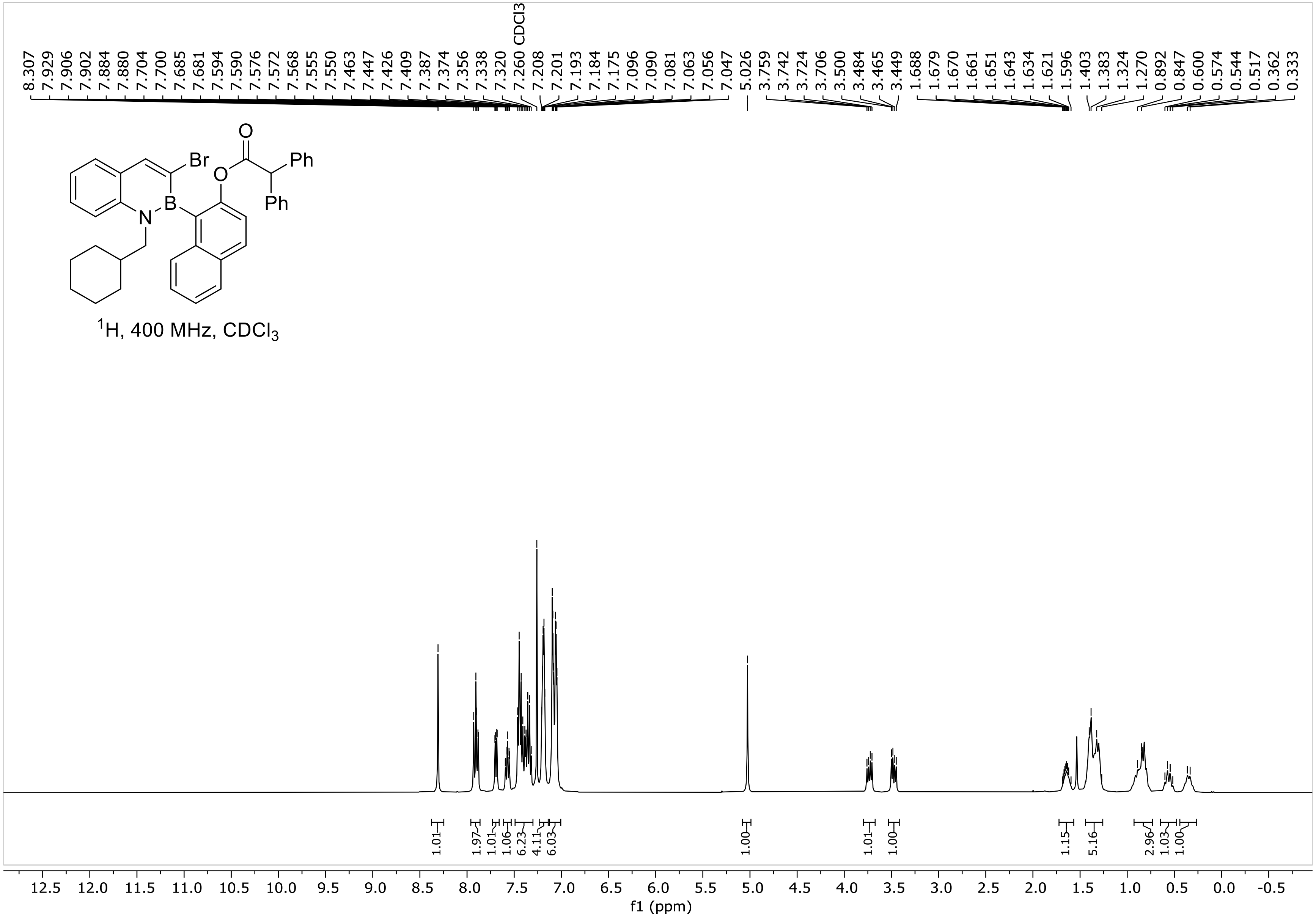

Br
O
O
Ph
Ph
B
N
^{1}H, 400 MHz, $CDCl_3$
8.307
7.929
7.906
7.902
7.884
7.880
7.704
7.700
7.685
7.681
7.594
7.590
7.576
7.572
7.568
7.555
7.550
7.463
7.447
7.426
7.409
7.387
7.374
7.356
7.338
7.320
7.260 CDCl3
7.208
7.201
7.193
7.184
7.175
7.096
7.090
7.081
7.063
7.056
7.047
5.026
3.759
3.742
3.724
3.706
3.500
3.484
3.465
3.449
1.688
1.679
1.670
1.661
1.651
1.643
1.634
1.621
1.596
1.403
1.383
1.324
1.270
0.892
0.847
0.600
0.574
0.544
0.517
0.362
0.333
1.01
1.97
1.01
1.06
6.23
4.11
6.03
1.00
1.01
1.00
1.15
5.16
2.96
1.03
1.00
12.5
12.0
11.5
11.0
10.5
10.0
9.5
9.0
8.5
8.0
7.5
7.0
6.5
6.0
5.5
5.0
4.5
4.0
3.5
3.0
2.5
2.0
1.5
1.0
0.5
0.0
-0.5
f1 (ppm)

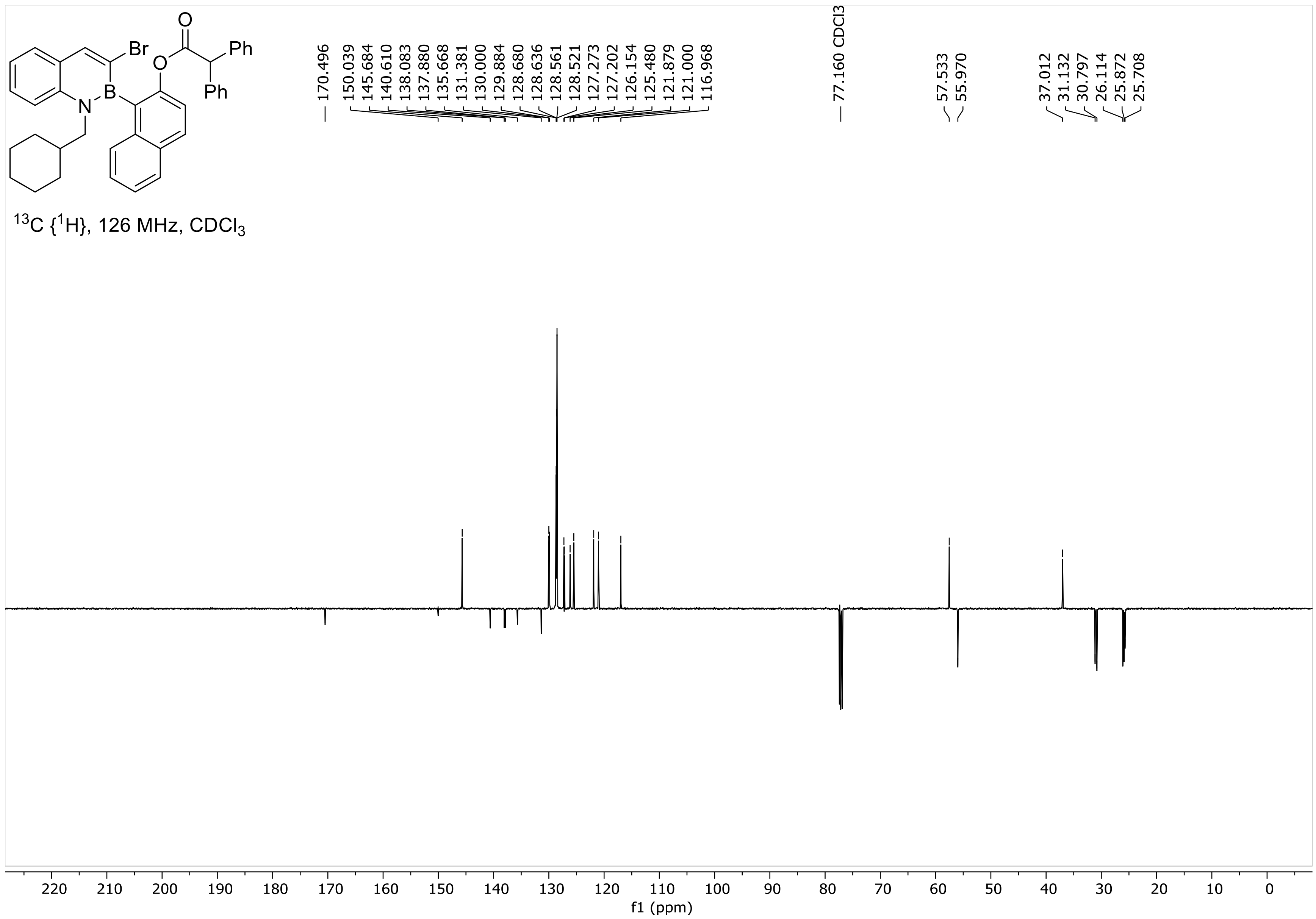
O
Br
O
Ph
Ph
N
B
13C {1H}, 126 MHz, CDCl3
170.496
150.039
145.684
140.610
138.083
137.880
135.668
131.381
130.000
129.884
128.680
128.636
128.561
128.521
127.273
127.202
126.154
125.480
121.879
121.000
116.968
77.160 CDCl3
57.533
55.970
37.012
31.132
30.797
26.114
25.872
25.708
220 210 200 190 180 170 160 150 140 130 120 110 100 90 80 70 60 50 40 30 20 10 0
f1 (ppm)

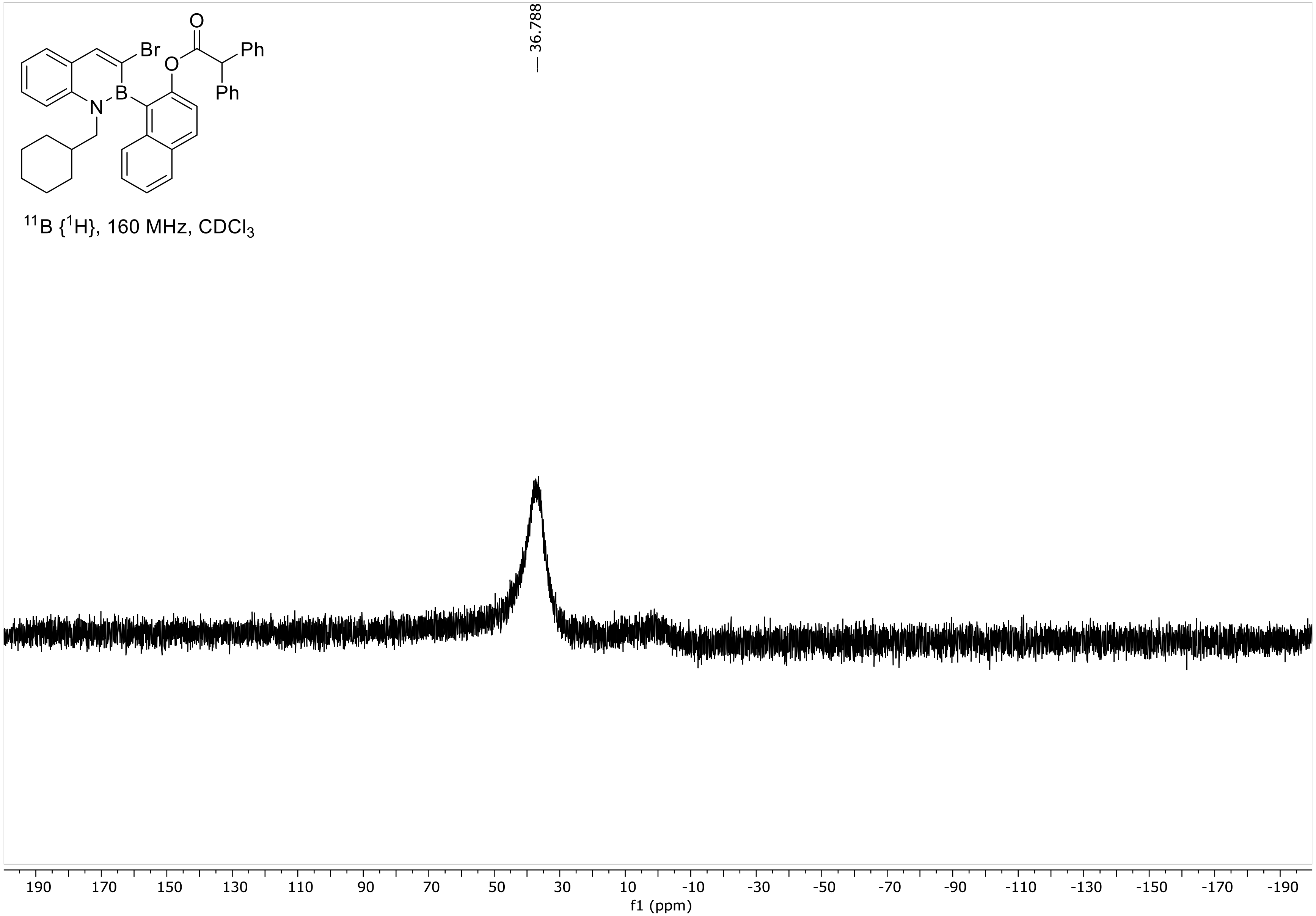

$^{11}B\ \{^{1}H\}$, 160 MHz, $CDCl_3$

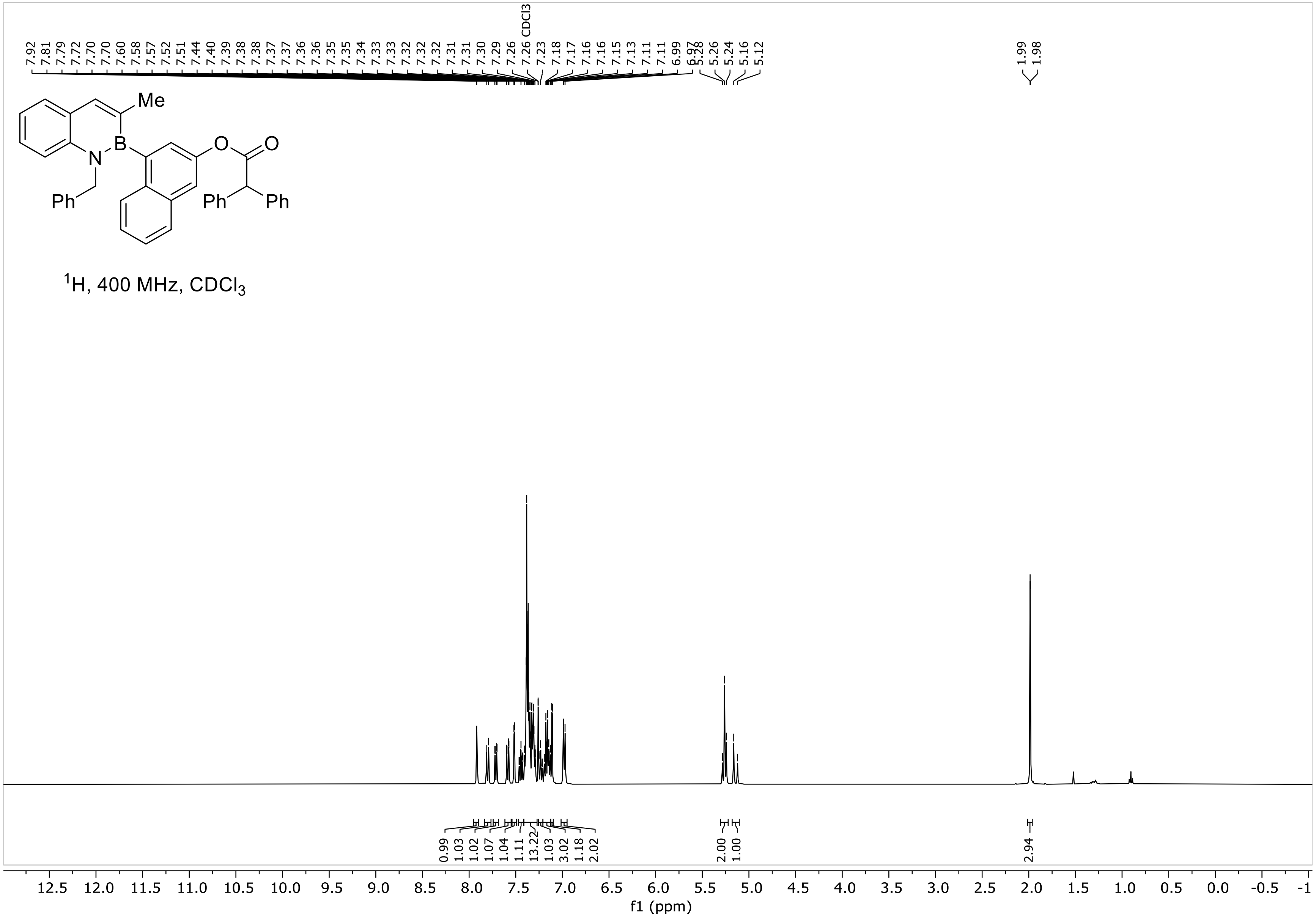
Me
B
N
O
O
Ph
Ph
Ph
¹H, 400 MHz, CDCl₃
f1 (ppm)

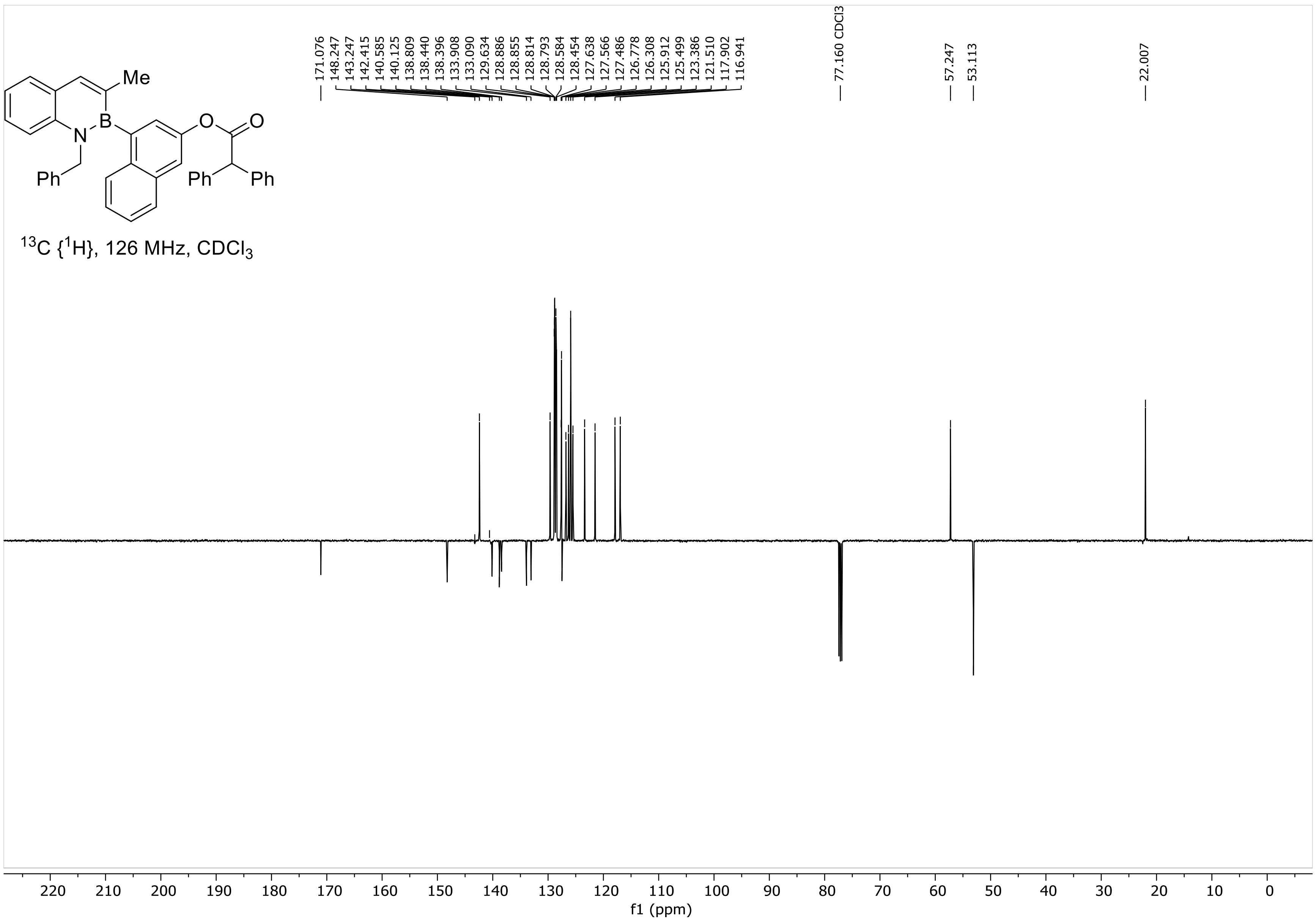
Me
B
N
O
O
Ph
Ph
Ph
13C {1H}, 126 MHz, CDCl3
171.076
148.247
143.247
142.415
140.585
140.125
138.809
138.440
138.396
133.908
133.090
129.634
128.886
128.855
128.814
128.793
128.584
128.454
127.638
127.566
127.486
126.778
126.308
125.912
125.499
123.386
121.510
117.902
116.941
77.160 CDCl3
57.247
53.113
22.007
220
210
200
190
180
170
160
150
140
130
120
110
100
90
80
70
60
50
40
30
20
10
0
f1 (ppm)

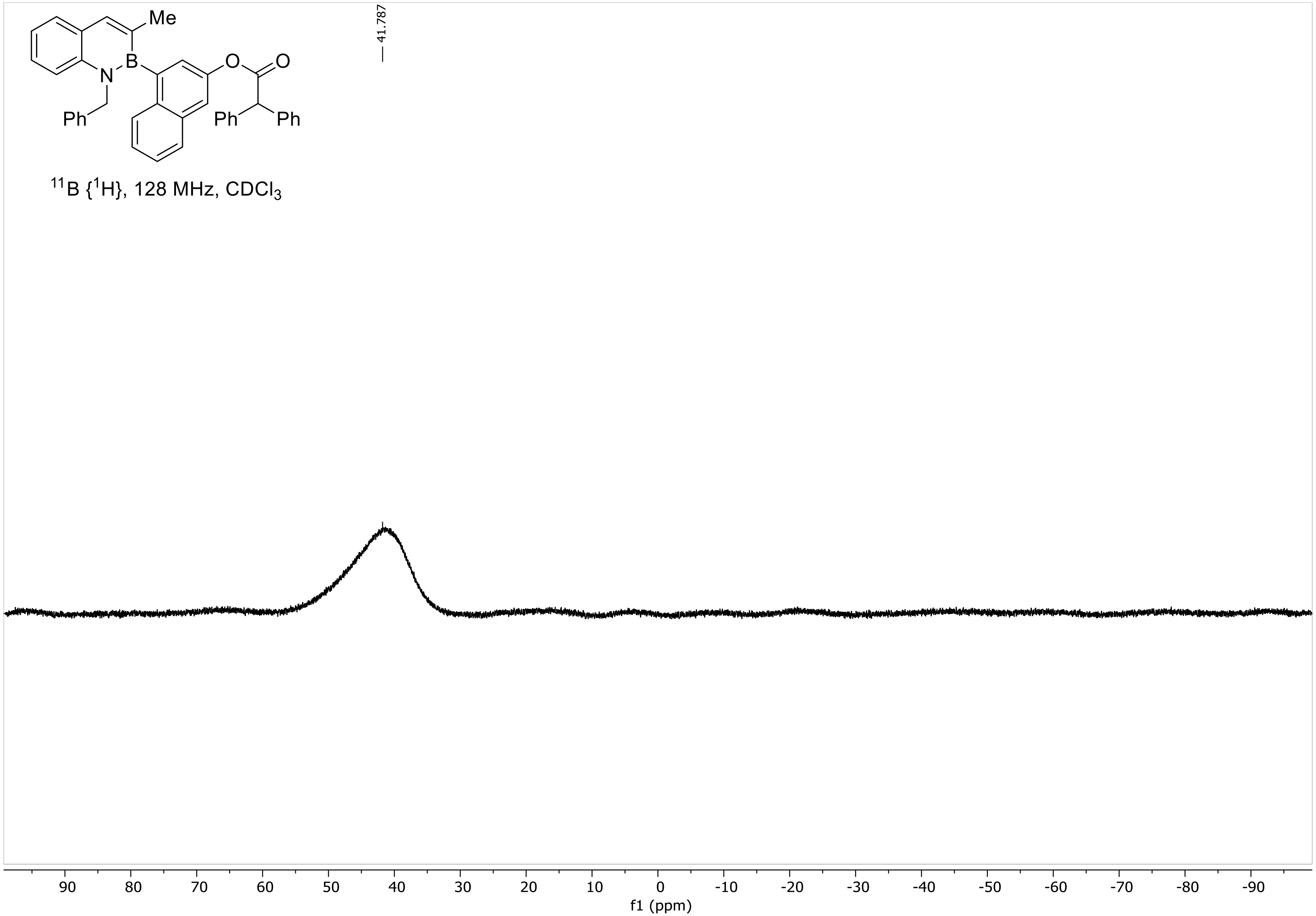
Me
B
N
O
O
Ph
Ph
Ph
11B {1H}, 128 MHz, CDCl3
41.787
90
80
70
60
50
40
30
20
10
0
-10
-20
-30
-40
-50
-60
-70
-80
-90
f1 (ppm)

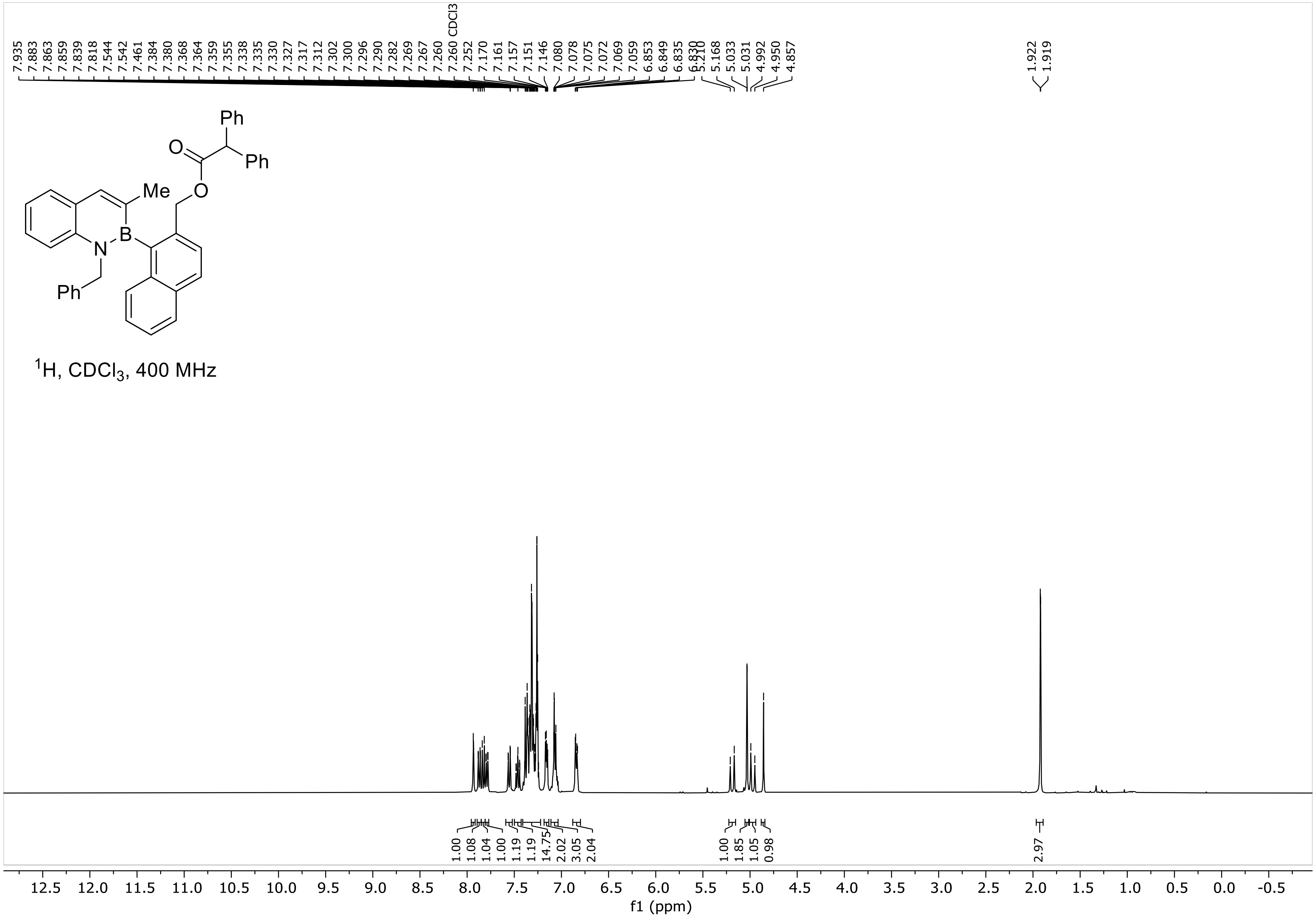

^1H, $CDCl_3$, 400 MHz

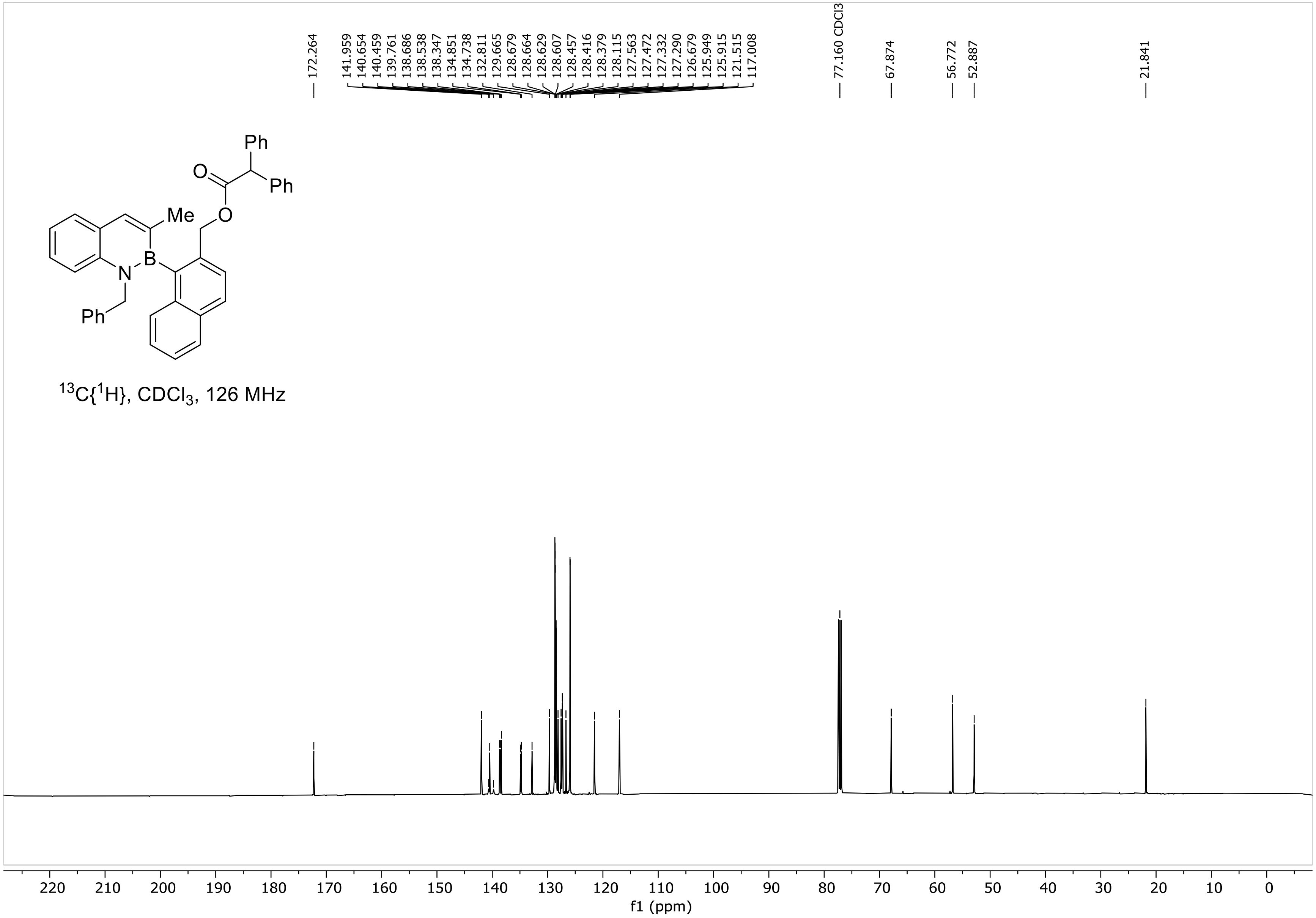

$^{13}C\{^{1}H\}$, $CDCl_3$, 126 MHz

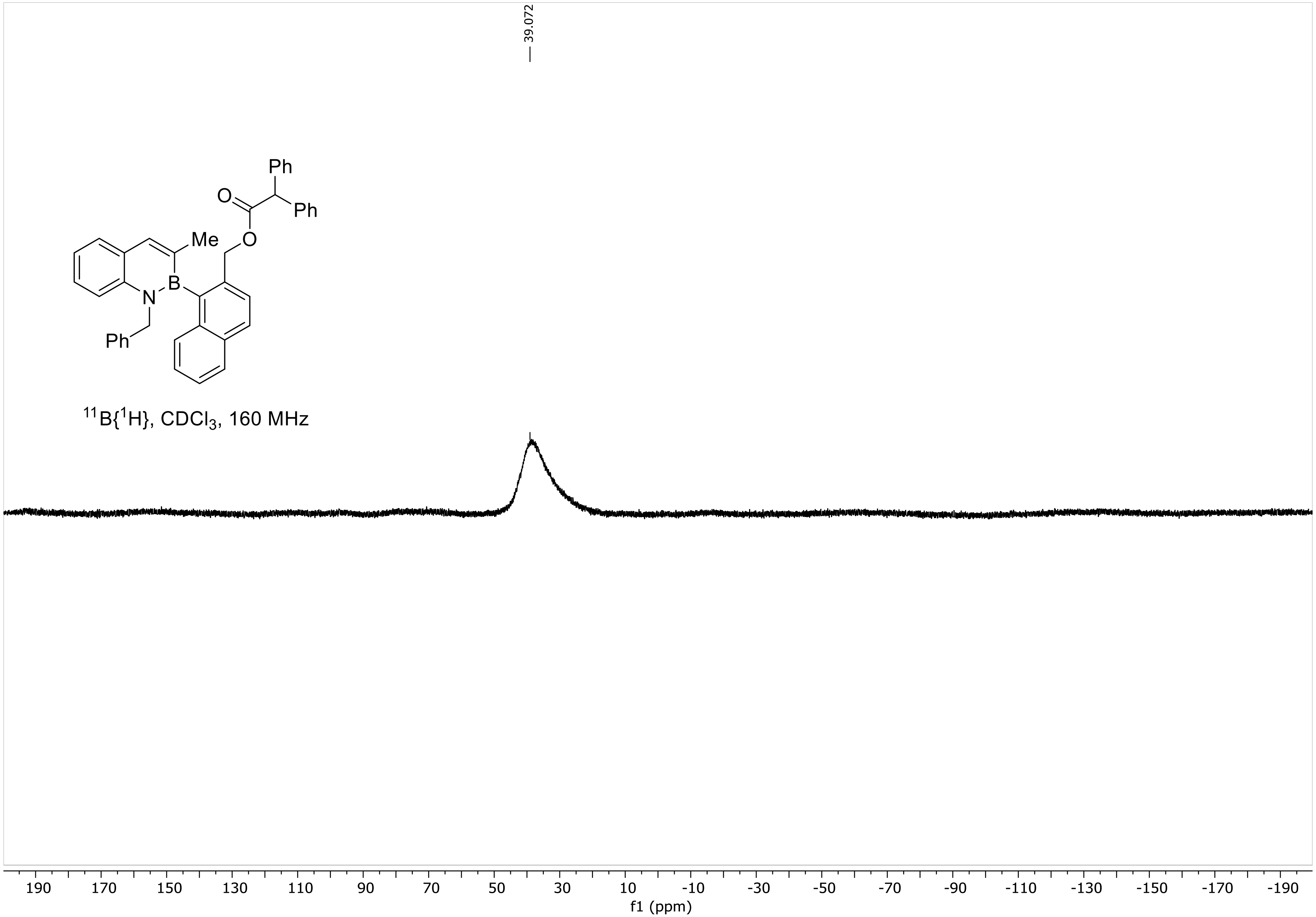
Ph
O
Ph
Me
O
B
N
Ph
11B{1H}, CDCl3, 160 MHz
39.072
190
170
150
130
110
90
70
50
30
10
-10
-30
-50
-70
-90
-110
-130
-150
-170
-190
f1 (ppm)

11. References

38. K. Yang, Y. Mao, Z. Zhang, J. Xu, H. Wang, Y. He, P. Yu and Q. Song, *Nat Commun*, 2023, **14**, 4438
56. S. Qu, M. D. Greenhalgh and A. D. Smith, *Chem. - Eur. J.*, 2019, **25**, 2816-2823.
62. J.-P. Heeb, J. Clayden, M. D. Smith and R. J. Armstrong, *Nat Protoc*, 2023, **18**, 2745-2771
63. DCXplorerMCXVII download link: https://www.cup.lmu.de/oc/trapp/tools.html (2026)
84. W. R. Scaggs and T. N. Snaddon, *Chem. - Eur. J.*, 2018, **24**, 14378-14381.
85. H. B. Kagan and J. C. Fiaud, in *Topics in Stereochemistry*, John Wiley & Sons, Ltd, 1988, DOI: 10.1002/9780470147276.ch4, pp. 249-330.
86. S. R. Wisniewski, C. L. Guenther, O. A. Argintaru and G. A. Molander, *J. Org. Chem.*, 2014, **79**, 365-378.
87. H. Zhu, A. Manchado, A. Omar Farah, A. P. McKay, D. B. Cordes, P. H.-Y. Cheong, K. Kasten and A. D. Smith, *Angew. Chem.*, 2024, **136**, e202402908.
88. R. Del Río-Rodríguez, M. T. Westwood, M. Sicignano, M. Juhl, J. A. Fernández-Salas, J. Alemán and A. D. Smith, *Chem. Commun.*, 2022, **58**, 7277-7280.
89. X. Chang, P.-L. Ma, H.-C. Chen, C.-Y. Li and P. Wang, *Angew. Chem.*, 2020, **132**, 9022-9025.
90. I. R. Hazelden, R. C. Carmona, T. Langer, P. G. Pringle and J. F. Bower, *Angew. Chem. Int. Ed.*, 2018, **57**, 5124-5128.
91. G. Chen, J. Gui, L. Li and J. Liao, *Angew. Chem. Int. Ed.*, 2011, **50**, 7681-7685.
92. M. Barbasiewicz, K. Błocki, M. Malińska and R. Pawłowski, *Dalton Trans.*, 2013, **42**, 355-358.
93. H. Albright, H. L. Vonesh and C. S. Schindler, *Org. Lett.*, 2020, **22**, 3155-3160.
94. A.-M. L. Hogan and D. F. O'Shea, *J. Am. Chem. Soc.*, 2006, **128**, 10360-10361.
95. A.-M. L. Hogan and D. F. O'Shea, *J. Org. Chem.*, 2008, **73**, 2503-2509.
96. A. D. J. Calow, D. Dailler and J. F. Bower, *J. Am. Chem. Soc.*, 2022, **144**, 11069-11074.
97. M. Weimar, R. Correa Da Costa, F.-H. Lee and M. J. Fuchter, *Org. Lett.*, 2013, **15**, 1706-1709.
98. J. Caeiro, D. Peña, A. Cobas, D. Pérez and E. Guitián, *Adv. Synth. Catal.*, 2006, **348**, 2466-2474.
99. B. Satpathi, L. Dutta and S. S. V. Ramasastry, *Org. Lett.*, 2019, **21**, 170-174.
100. D. Moser, K. Jana and C. Sparr, *Angew. Chem.*, 2023, **135**, e202309053.
101. G. A. Molander and S. R. Wisniewski, *J. Org. Chem.*, 2014, **79**, 6663-6678.
102. H. Xu, T. Du, J. Lin, F. You, Y. Shao, Q. Yang and X. Li, *Angew. Chem. Int. Ed.*, 2026, **65**, e21349.
103. T.-H. Doan, A. Chardon, N. Vanthuyne, T. N. Ramos, N. Tumanov, L. Fusaro, M. Albalat, L. Collard, J. Wouters, B. Champagne and G. Berionni, *Angew. Chem. Int. Ed.*, 2025, **64**, e202421931.

104. CrysAlisPro v1.171.44.121a. Rigaku Oxford Diffraction, Rigaku Corporation, Tokyo, Japan, 2025
105. G. M. Sheldrick, *Acta Cryst A*, 2015, **71**, 3-8.
106. G. M. Sheldrick, *Acta Cryst C*, 2015, **71**, 3-8.
107. O. V. Dolomanov, L. J. Bourhis, R. J. Gildea, J. a. K. Howard and H. Puschmann, *J Appl Cryst*, 2009, **42**, 339-341.
